# Membrane‐Permeable Nucleoside T‐1106 Diphosphate and Triphosphate Analogues as Antiviral Pronucleotides

**DOI:** 10.1002/chem.71181

**Published:** 2026-05-27

**Authors:** Chris Meier, Duan Li, Leentje Persoons, Graciela Andrei, Robert Snoeck, Dominique Schols, Lieve Naesens, Xiao Jia

**Affiliations:** ^1^ Organic Chemistry Department of Chemistry Faculty of Mathematics Informatics and Natural Sciences Universität Hamburg Hamburg Germany; ^2^ Centre for Structural Systems Biology (CSSB) Hamburg DESY Campus Hamburg Germany; ^3^ State Key Laboratory of Discovery and Utilization of Functional Components in Traditional Chinese Medicine School of Pharmaceutical Sciences Guizhou Medical University Guizhou China; ^4^ Zhongshan Institute for Drug Discovery Shanghai Institute of Materia Medica Chinese Academy of Sciences Zhongshan China; ^5^ Molecular Genetics and Therapeutics in Virology and Oncology Department of Microbiology and Immunology and Transplantation Rega Institute KU Leuven Leuven Belgium; ^6^ Molecular, Structural and Translational Virology Department of Microbiology and Immunology and Transplantation Rega Institute KU Leuven Leuven Belgium; ^7^ State Key Laboratory of Drug Research Shanghai Institute of Materia Medica Chinese Academy of Sciences Shanghai China

**Keywords:** antiviral activity, nucleoside analogue, nucleoside diphosphates, nucleoside triphosphates, prodrugs

## Abstract

The nucleobase T‐1105, the non‐fluorinated analogue of favipiravir (T‐705), inhibits viral RNA synthesis after conversion to its ribonucleoside‐5′‐triphosphate (T‐1105‐RTP) metabolite. However, this metabolic activation is rate‐limiting and inefficient. We here report the chemical synthesis and evaluation of three series of T‐1105‐ribonucleoside (T‐1106)‐Di*PP*ro‐ and Tri*PPP*ro‐nucleotide prodrugs to allow their cellular uptake. Their efficient release of the T‐1106‐diphosphate and triphosphate forms was demonstrated upon incubation in PBS, pig liver esterase, extract of human T‐lymphoblast CEM/0 cells and human plasma. This validates the successful hydrolysis and delivery of the corresponding nucleotides, via chemical or enzyme‐triggered mechanisms. Interestingly, several T‐1106‐Di*PP*ro‐prodrugs, for example, compound **24h**, and Tri*PPP*ro‐prodrugs proved superior to the parent T‐1106 nucleoside, in inhibiting influenza A and influenza B replication in MDCK cells, and of human coronavirus OC43 in HEL299 cells. This validates the utility of our pronucleotide strategy to overcome the inefficient activation of T‐1106 and increase the antiviral efficacy against influenza and coronaviruses.

## Graphical abstract

Favipiravir (T‐705) and the non‐fluorinated counterpart (T‐1106) are antiviral agents that inhibit the RNA‐dependent RNA polymerase (RdRp) of various RNA viruses. The antiviral efficacy of nucleoside analogues is strongly dependent on their intracellular activation by cellular kinases to produce their corresponding triphosphate metabolites (T‐705‐RTP and T‐1106‐TP). Herein, we report the development of three versions of T‐1106‐Di*PP*ro‐ and Tri*PPP*ro‐nucleotide prodrugs comprising two identical or different masking groups at the terminal β‐ and γ‐phosph(on)ate. Efficient release of T‐1106‐diphosphate (T‐1106‐DP) and T‐1106‐triphosphate (T‐1106‐TP) were confirmed in different medium, validating nucleotide delivery via chemical or enzyme‐triggered hydrolysis. The active T‐1106‐TP is incorporated into viral RNA by RdRp, disrupting viral replication. This strategy aims to inhibit influenza A, influenza B, and human coronaviruses, offering a promising approach to combat various viral pathogens.

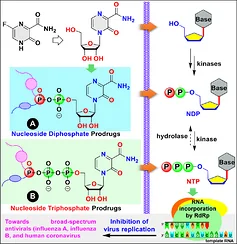

## 1 Introduction

The frequent emergence of zoonotic RNA viruses, such as avian influenza, Zika, Ebola and severe acute respiratory syndrome coronavirus 2 (SARS‐CoV‐2), is associated with significant morbidity and mortality [1, 2, 3]. Besides, seasonal influenza A (IAV) and influenza B (IBV) viruses account each year for ∼290,000 to 650,000 deaths worldwide [4]. To complement vaccines, effective antiviral drugs must at all times be available, with broad‐acting agents having a clear advantage. One prominent example is the nucleobase analogue favipiravir (T‐705 **1**; 6‐fluoro‐3‐hydroxypyrazine‐2‐carboxamide), an antiviral that is formally approved in Japan and China. In addition to IAV and IBV, the broad anti‐RNA virus activity of T‐705 encompasses other severe pathogens, such as yellow fever virus, West Nile virus, rabies virus, Rift Valley fever virus and Ebola virus [5, 6, 7, 8, 9, 10, 11, 12, 13, 14, 15].

Mechanistically, the ribonucleoside‐5′‐triphosphate form of T‐705 (T‐705‐RTP **4**) is recognized by viral RNA‐dependent RNA polymerase (RdRp) enzymes as a mimic of guanosine triphosphate (GTP) or adenosine triphosphate (ATP). Since its incorporation does not *a priori* result in chain termination, T‐705 induces accumulation of mutations in the viral genome, known as lethal virus mutagenesis. Also SARS‐CoV RdRp readily incorporates T‐705‐RTP, which escapes from being excised since it is not recognized by the coronavirus exonuclease [16].

**FIGURE 1:**
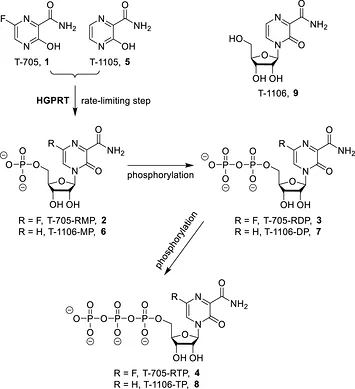
Conversion of Favipiravir (T‐705) **1** and T‐1105 **5** into their active 5′‐triphosphates.

The metabolic activation of T‐705 into T‐705‐RTP **4** starts with hypoxanthine guanine phosphoribosyl transferase (HGPRT)‐mediated formation of the ribonucleoside‐5′‐monophosphate (T‐705‐RMP **2**, Figure 1) [12, 17], which is followed by two additional phosphorylations [5, 18, 19]. However, the chemical instability of the T‐705‐ribonucleos(t)ides bearing the fluorinated heterocycle can lead to stability issues when applying T‐705 in biochemical studies and as an antiviral drug [20, 21, 22]. Furthermore, since T‐705 **1** is a very poor substrate for HGPRT, the overall conversion of T‐705 **1** into T‐705‐RTP **4** is highly inefficient, explaining the poor potency of T‐705 **1** as an antiviral drug.

To execute antiviral effect, the fluorine atom of T‐705 **1** is not required. In several studies [5, 6, 7, 8, 9, 10, 11, 12, 13, 14, 15], the non‐fluorinated analogues T‐1105 (3‐hydroxy‐pyrazine‐2‐carboxamide) **5** and its nucleoside form T‐1106 (3,4‐dihydro‐3‐oxo‐4‐*β*‐D‐ribofuranosyl‐2‐pyrazine‐carboxamide) **9** (Figure 1) exhibited antiviral activity that was similar as or even superior to that of T‐705 **1**. This aligns with reports showing that T‐1106‐triphosphate (T‐1106‐TP **8**) is efficiently formed in RNA virus‐infected cells [18] and notably more stable than T‐705‐RTP **4** in enzymatic assays.

**FIGURE 2:**
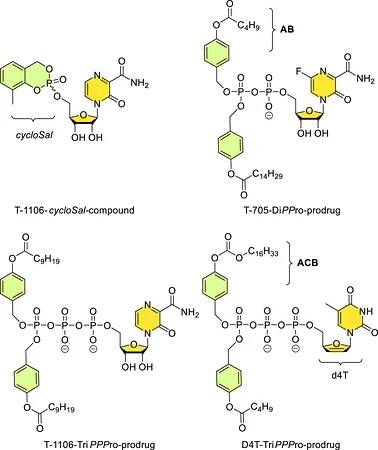
Chemical structure of representative T‐705‐, T‐1106‐ and d4T‐pronucleotides comprising AB (acyloxybenzyl), ACB (alkoxycarbonyloxybenzyl) or *cyclo*Sal (*cyclo*Saligenyl)‐prodrug entities.

Several pronucleotide strategies have been developed to circumvent the inefficient phosphorylation of antiviral nucleoside analogues and to improve the ability of nucleotide drugs to cross cell membranes. Our own group has been focusing on prodrugs of the nucleoside monophosphate (*cyclo*Sal) [23], nucleoside diphosphate (Di*PP*ro) [24, 25, 26, 27, 28, 29, 30] and nucleoside triphosphate (Tri*PPP*ro) forms [31, 32, 33, 34, 35, 36, 37, 38, 39, 40, 41, 42]. In 2018, we reported on the first synthesis of T‐705‐ and T‐1106‐*cyclo*Sal‐, Di*PP*ro‐ and Tri*PPP*ro‐compounds (Figure 2) [24]. Whereas the *cyclo*Sal‐derivatives were inactive, we found promising anti‐influenza virus activity in Madin–Darby Canine Kidney (MDCK) cells (including an HGPRT‐deficient line) for the T‐1106 di‐ and triphosphate‐prodrugs comprising two lipophilic biocleavable acyloxybenzyl (AB) groups [(AB‐C9;AB‐C9) and (AB‐C4;AB‐C14)] at the β‐ or γ‐phosphate, respectively. This provided proof‐of‐concept for successful membrane permeability and formation of the nucleotides (T‐1106‐diphosphate, T‐1106‐DP **7** or T‐1106‐TP **8**) by an enzyme‐triggered mechanism in these cells. As expected, the nucleoside T‐1106 moiety was entirely stable. In contrast, decomposition of the T‐705 nucleoside, most probably by nucleophilic aromatic substitution, occurred parallel or even faster than chemical hydrolysis of the phenyl ester within the AB‐masking moieties or of the phosphoranhydride bonds in the T‐705‐diphosphate prodrug (T‐705‐Di*PP*ro‐prodrug (AB‐C4;AB‐C14)) [24].

Additionally, we developed second generation Di*PP*ro‐ [43, 44] and Tri*PPP*ro‐pronucleotides [43, 45] which comprise one biocleavable group in addition to one non‐cleavable alkyl moiety, and offer successful intracellular delivery of nucleoside diphosphate or triphosphate derivatives. Their efficient metabolic bypass and superior antiviral activities were demonstrated in HIV‐infected cells [43, 44, 45]. Finally, our third generation Di*PP*ro‐ and Tri*PPP*ro‐compounds bear two different, non‐biocleavable and hydrolytically stable alkyl moieties. They show high chemical and enzymatic stability in different chemical and biological media such as phosphate buffered saline (PBS), pig liver esterase (PLE), and human T‐lymphoblast (CEM/0) cell extracts besides marked anti‐HIV activity in CEM/0 cells, including a thymidine kinase‐deficient cell line [43, 44, 46]. Recently, we reported on a high performance liquid chromatography (HPLC) mass spectrometry method that allowed the quantification of the formed metabolites from Tri*PPP*ro‐derivatives bearing the antitumor nucleoside analogue 5‐fluorouridine. There, we observed a rapid uptake of the pronucleotide and the efficient formation of the key metabolites [47].

Encouraged by these previous results, we here applied our three masking approaches [48] (AB/ACB, series 1 compounds) [33]; AB/alkyl (series 2 pronucleotides [37, 38]; alkyl/alkyl (series 3 prodrugs) [43]) to the nucleoside analogue T‐1106 **9**. Our aim was to study if various types of T‐1106‐Di*PP*ro‐ and Tri*PPP*ro‐nucleotides would exhibit higher potency or a broader activity spectrum than the parent nucleoside [49]. Hence, we conducted the chemical synthesis of three series of compounds, to then evaluate their stability in different media. To assess the broad antiviral potential, we performed cell‐based assays with a wide variety of RNA viruses including, among others, influenza virus, coronaviruses, flaviviruses and HIV.

## 2 Results and Discussion

### 2.1 Synthesis of T‐1106‐MP **6** and T‐1106‐DP **7**

**SCHEME 1:**
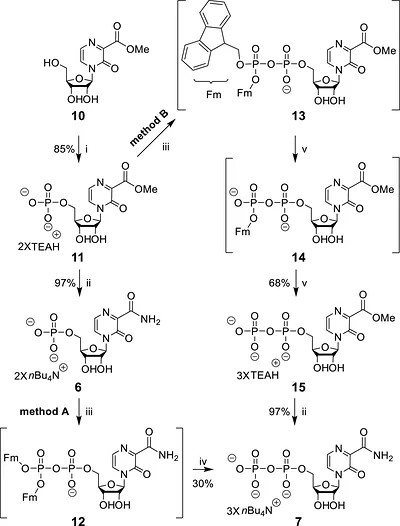
Reagents and conditions: (i) Trimethyl phosphate (TMP), POCl_3_, 0°C, 2 h; (ii) a. 7 M NH_3_ (MeOH), rt – 50°C, 24–48 h, b. Dowex 50WX8 (H^+^ form) ion exchange, 40% (Bu_4_N)_4_OH in H_2_O; (iii) (Fm;Fm)‐*H*‐phosphonate, NCS, THF, CH_3_CN, rt, 2 h; (iv) a. TEA, THF, CH_3_CN, rt, 24–48 h. b. Dowex 50WX8 (H^+^ form) ion exchange, 40% (Bu_4_N)_4_OH in H_2_O. (v) TEA, THF, CH_3_CN, rt, 24–48 h.

As starting materials for the pronucleotide synthesis, we used nucleotides T‐1106‐MP **6** (T‐1106‐monophosphate) and T‐1106‐DP **7**. Although the synthetic procedures for these two molecules have been previously reported [24, 41], a number of reaction steps were required to start from T‐1106 **9**. In order to achieve satisfactory yields and purities for T‐1106‐MP **6** and T‐1106‐DP **7**, we developed a novel method (Scheme 1). First, the ester‐bearing nucleoside monophosphate **11** (Et_3_NH^+^ form) was prepared employing a synthesis starting from the pyrazinemethylcarboxylate analogue **10** in a very good overall yield of 85%. Subsequently, the methyl ester **11** was transformed into T‐1106‐MP **6** (NH_4_^+^ form) using a methanol solution of aqueous ammonia (7 M) [49]. T‐1106‐MP **6**, in its *n*‐Bu_4_N^+^ form with a purity of 97%, was obtained as a solid through a series of steps, including Dowex 50WX8 (H^+^) ion exchange, neutralization using tetra‐*n*‐butylammonium hydroxide, and freeze‐drying.

**SCHEME 2:**
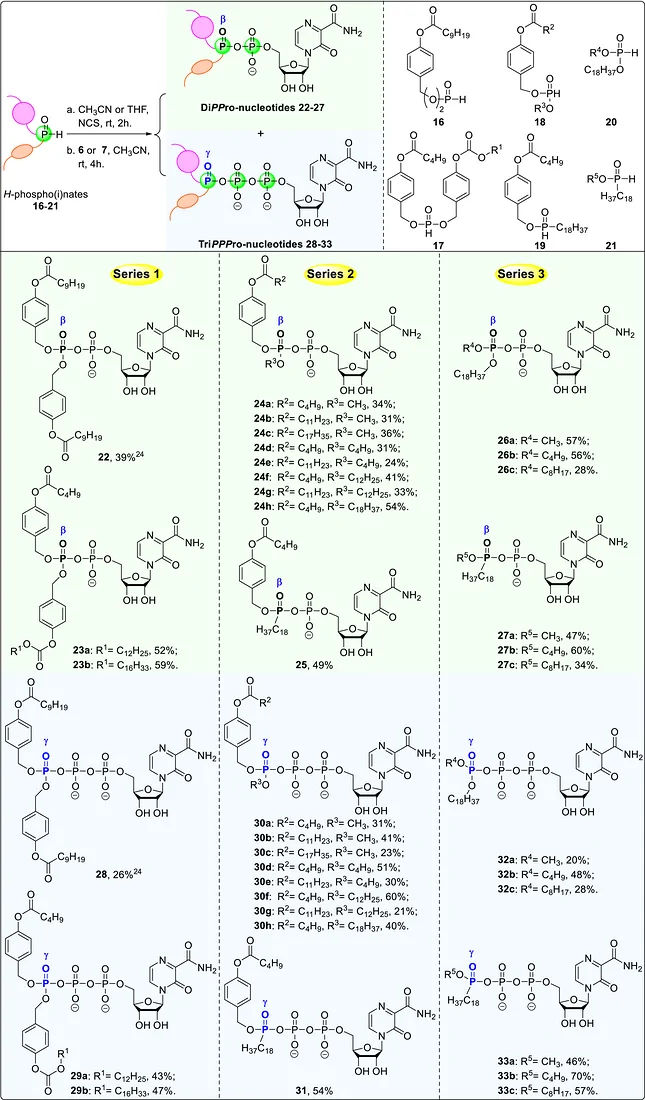
Synthesis of T‐1106‐Di*PP*ro‐ and Tri*PPP*ro‐nucleotides **22–33**.

Nucleotide **6** was used for the synthesis of T‐1106‐Di*PP*ro‐prodrugs **22–27** (Scheme 2). Commencing with T‐1106‐MP **6** (*n*‐Bu_4_N^+^ form), the synthesis of T‐1106‐DP **7** (*n*‐Bu_4_N^+^ form) was accomplished in relatively low yields (approx. 30%) using the *H*‐phosphonate protocol (method A) [24]. Consequently, nucleoside monophosphate **11** (Et_3_NH^+^ form) was converted following a standard procedure to yield bis(fluorenylmethyl)‐(Fm) protected diphosphate‐derivative **13**, which was then deprotected by two individual steps to give the nucleoside diphosphate **15** (Et_3_NH^+^ form). Accordingly, under rigorous anhydrous conditions (method B), one Fm‐group was cleaved selectively by short treatment with triethylamine (TEA, 3 equiv.) for 10 min only to form the intermediate **14**, followed by a subsequent cleavage of the second Fm‐group by long (24‐48 h) treatment with TEA (≥ 10 equiv.) to yield nucleoside diphosphate **15** (Et_3_NH^+^ form, 68%). After hydrolysis of nucleoside diphosphate **15** (Et_3_NH^+^ form) with aqueous ammonia (7 M) in methanol, T‐1106‐DP **7** (*n*‐Bu_4_N^+^ form) was successfully isolated in an almost quantitative yield (97%).

### 2.2 Synthesis of T‐1106‐Di*PP*ro‐ and T1106‐Tri*PPP*ro‐nucleotides **22–33**

Our group first reported on T‐1106‐Di*PP*ro‐ and Tri*PPP*ro‐prodrugs **22**,**28** using methods based on phosphoramidite P(III) chemistry [24]. In contrast, here the synthesis of T‐1106‐Di*PP*ro‐ and Tri*PPP*ro‐nucleotides **22–33** has been accomplished by using P(V) chemistry, which is outlined in Scheme 2. Thus, no oxidation step is needed to convert the P(III)‐intermediate into the P(V)‐product. Due to low solubility associated with T‐1106‐MP **6** and T‐1106‐DP **7**, we opted to utilize these compounds in *n*Bu_4_N^+^ form. Hence, their solubility in CH_3_CN led to satisfactory yield and purity, with overall yields ranging from 20% to 70%. In all cases, *H*‐phosphonates **16–18**,**20** or *H*‐phosphinates **19**,**21** were reacted with *N*‐chlorosuccinimide (NCS) [50] to form the corresponding phosphorochloridates and phosphonochloridates, followed by phosphorylation of T‐1106‐MP **6** (*n*‐Bu_4_N^+^ form) and T‐1106‐DP **7** (*n*‐Bu_4_N^+^ form) to yield T‐1106‐Di*PP*ro‐nucleotides **22–27** (*n*‐Bu_4_N^+^ form) and T‐1106‐Tri*PPP*ro‐nucleotides **28–33** (*n*‐Bu_4_N^+^ form), respectively. In the final step, T‐1106‐prodrugs **22–33** (NH_4_^+^ form) were obtained through a Dowex 50WX8 ion exchange, converting *n*‐Bu_4_N^+^ to NH_4_^+^, followed by freeze‐drying and the overall yields ranged between 20% and 70% (Scheme 2).

### 2.3 Synthesis of the β‐ and γ‐monoalkylated T‐1106‐compounds **40,41,47,48**

**SCHEME 3:**
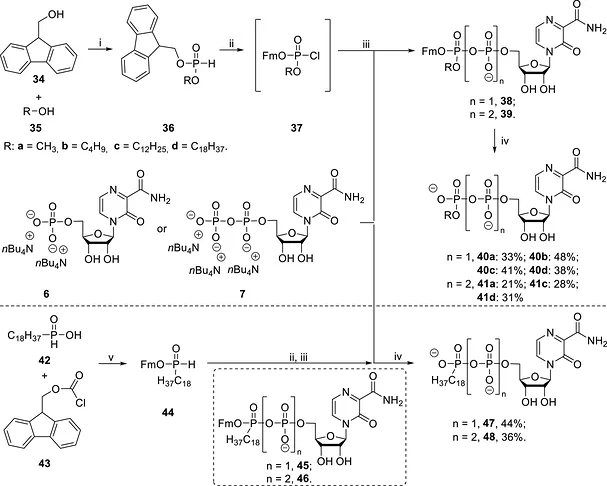
Reagents and conditions: (i) Diphenyl hydrogen phosphonate (DPP), pyridine, 0°C‐50°C, 12 h; (ii) CH_3_CN or THF, NCS, rt, 2 h; (iii) **6** (*n*‐Bu_4_N^+^) or **7** (*n*‐Bu_4_N^+^), CH_3_CN, rt, 3–4 h; iv) Et_3_N, CH_3_CN, rt, 8–12 h, Dowex 50WX8 (NH_4_^+^ form) ion exchange; v) *N*‐methylmorpholine, Et_2_O, toluene, 0°C‐rt, 4 h.

At the first step, commercially available alcohols **34** and **35** were reacted with diphenyl hydrogen phosphonate (DPP) to afford (Fm;alkyl)‐*H*‐phosphonates **36**. Starting from *H*‐phosphonates **36** [44], β‐(Fm;alkyl)‐T‐1106‐DP compounds **38** and γ‐(Fm;alkyl)‐T‐1106‐TP compounds **39** were successfully synthesized using the *H*‐phosphonate route (Scheme 3, upper section). Afterwards, selective cleavage of the Fm‐group from T‐1106‐compounds **38** and **39**) was obtained through treatment with TEA in THF or CH_3_CN, resulting in the formation of β‐ and γ‐mono‐masked compounds **40** and **41** (21‐48%). The *H*‐phosphinate protocol (Scheme 3, lower section) was similarly applied to the synthesis of β‐ or of γ‐monoalkylated compounds **47** and **48** via intermediates **45**,**46** resulting in yields of 44% and 36%, respectively.

### 2.4 Chemical and Enzymatic Hydrolysis Studies

**SCHEME 4:**
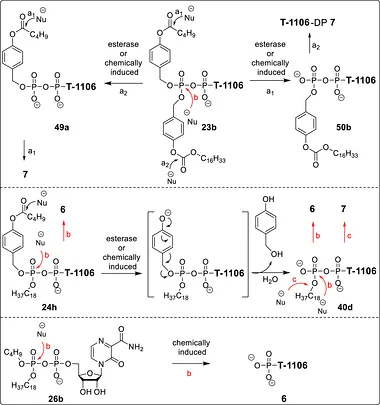
Possible hydrolysis pathways of the T‐1106‐Di*PP*ro compounds, shown for compounds **23b** (series 1), **24h** (series 2), and **26b** (series 3) as examples. Pathway description: a_1_: cleavage of the acyloxybenzyl (AB) prodrug group; a_2_: cleavage of the acyloxycarbonyloxybenzyl (ACB) prodrug group; b: cleavage of the phosphate anhydride bond; c: phosphate‐alkyl‐ester cleavage.

**SCHEME 5:**
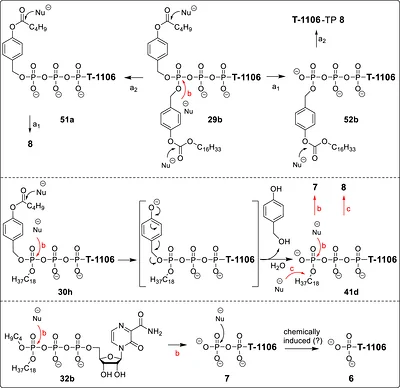
Possible hydrolysis pathways of the T‐1106‐Tri*PPP*ro compounds, shown for compounds **23b** (series 1), **24h** (series 2) and **26b** (series 3) as examples. a_1_: cleavage of the acyloxybenzyl (AB) prodrug group; a_2_: cleavage of the acyloxycarbonyloxybenzyl (ACB) prodrug group; b: cleavage of the phosphate anhydride bond; c: phosphate‐alkyl‐ester cleavage.

To verify the chemically or enzymatically triggered nucleotide release, compounds **22–33**, **40**, **41**, **47**, and **48** were incubated in PBS (25 mM, pH 7.3) or with pig liver esterase (5.6 U/mL, PLE) as a model enzyme. The hydrolysis half‐lives are compiled in Table 1. As a general guideline, the compounds should be stable for more than 15 h in PBS to allow cellular uptake. On the other hand, their esterase stability should be low to achieve efficient delivery inside the cells. Selected compounds were also incubated with cell extract of human CEM/0 T‐lymphoblasts as well as with human plasma. The hydrolysis products were identified by reverse‐phase ion‐pair high performance liquid chromatography (RP‐HPLC, UV‐detection), yielding the proposed hydrolysis pathways in Scheme 4 (for T‐1106‐DP derivatives) and Scheme 5 (for T‐1106‐TP derivatives).

**TABLE 1:** Hydrolysis half‐lives of the compounds in PBS and PLE, as well as their retention times on RP‐HPLC.

Compound		R^2^, R^4^, or R^5^	R^1^or R^3^	PBS pH = 7.3 *t*_1/2_ (h)	PLE *t*_1/2_ (h)	RP‐HPLC *t*_R_ (min)
**Series 1: two biodegradable groups**						
Di*PP*ro	**23b**	C_4_H_9_	C_16_H_33_	46	1.4	20.4
Tri*PPP*ro	**29b**	C_4_H_9_	C_16_H_33_	11	1.8	18.4
**Series 2: one biodegradable group and one non‐cleavable alkyl group**						
Di*PP*ro	**24b**	C_11_H_23_	CH_3_	32	< 0.01	15.6
	**24c**	C_17_H_35_	CH_3_	1.7	0.20	19.4
	**24e**	C_11_H_23_	C_4_H_9_	12	< 0.01	16.4
	**24f**	C_4_H_9_	C_12_H_25_	n.d.^a^	< 0.01	16.0
	**24g**	C_11_H_23_	C_12_H_25_	n.d.^a^	1.1	20.3
	**24h**	C_4_H_9_	C_18_H_37_	97	1.7	20.0
	**25**	C_4_H_9_	C_18_H_37_	>100	0.22	19.6
Tri*PPP*ro	**30b**	C_11_H_23_	CH_3_	23	0.038	14.8
	**30c**	C_17_H_35_	CH_3_	25	0.77	17.2
	**30e**	C_11_H_23_	C_4_H_9_	30	0.012	15.2
	**30f**	C_4_H_9_	C_12_H_25_	46	0.029	15.0
	**30g**	C_11_H_23_	C_12_H_25_	n.d.^a^	0.31	17.4
	**30h**	C_4_H_9_	C_18_H_37_	38	0.52	17.3
	**31**	C_4_H_9_	C_18_H_37_	54	0.64	17.1
**Series 3: two non‐cleavable alkyl groups**						
Di*PP*ro	**26b**	C_4_H_9_	C_18_H_37_	>200	>40	18.2
	**27b**	C_4_H_9_	C_18_H_37_	n.d.^a^	>40	17.9
Tri*PPP*ro	**32b**	C_4_H_9_	C_18_H_37_	>200	>40	17.0
	**33b**	C_4_H_9_	C_18_H_37_	>200	>40	16.7
**Only one non‐cleavable alkyl groups at the terminal phosphate**						
Di*PP*ro	**40a**	—	CH_3_	>500	>40	9.3
	**40d**	—	C_18_H_37_	>200	>40	15.1
	**47**	—	C_18_H_37_	63	>40	14.8
	**41a**	—	CH_3_	>500	>200	10.9
Tri*PPP*ro	**41d**	—	C_18_H_37_	>500	>40	14.8
	**48**	—	C_18_H_37_	67	>40	14.5

The hydrolysis experiments were conducted in 25 mM phosphate buffered saline (PBS, pH = 7.3), or with PLE (5.6 U/mL). The products were detected by RP18‐HPLC. *β*‐(AB‐C4;ACB‐C16)‐T‐1106‐DP **23b**; *β*‐(AB:C4‐C17;alkyl:C1‐C18)‐T‐1106‐DPs **24b**‐**h**; *β*‐(AB‐C4)‐*β*‐C‐(alkyl‐C18)‐T‐1106‐DP **25**; *β*‐(alkyl‐C4;alkyl‐C18)‐T‐1106‐DP **26b**; *β*‐(alkyl‐C4)‐*β*‐C‐(alkyl‐C18)‐T‐1106‐DP **27b**; *γ*‐(AB‐C4;ACB‐C16)‐T‐1106‐TP **29b**; *γ*‐(AB:C4‐C17;alkyl:C1‐C18)‐T‐1106‐TPs **30b**‐**h**; *γ*‐(AB‐C4)‐*γ*‐C‐(alkyl‐C18)‐T‐1106‐TP **31**; *γ*‐(alkyl‐C4;alkyl‐C18)‐T‐1106‐TP **32b**; *γ*‐(alkyl‐C4)‐*γ*‐C‐(alkyl‐C18)‐T‐1106‐TP **33b**; *β*‐(alkyl:C1‐C18)‐T‐1106‐DPs **40a**,**d**; *γ*‐(alkyl:C1‐C18)‐T‐1106‐TPs **41a**,**d**; *β*‐C‐(alkyl‐C18)‐T‐1106‐DP **47**; *γ*‐C‐(alkyl‐C18)‐T‐1106‐TP **48**.

^a^n.d.: not determined. Assay conditions are given in the Experimental Section.

### 2.5 Series 1: Di*PP*ro‐prodrugs **23a,b** and Tri*PPP*ro‐prodrug **29a,b** Bearing Two Biodegradable Masking Groups

#### 2.5.1 Hydrolysis in PBS, pH 7.3

**FIGURE 3:**
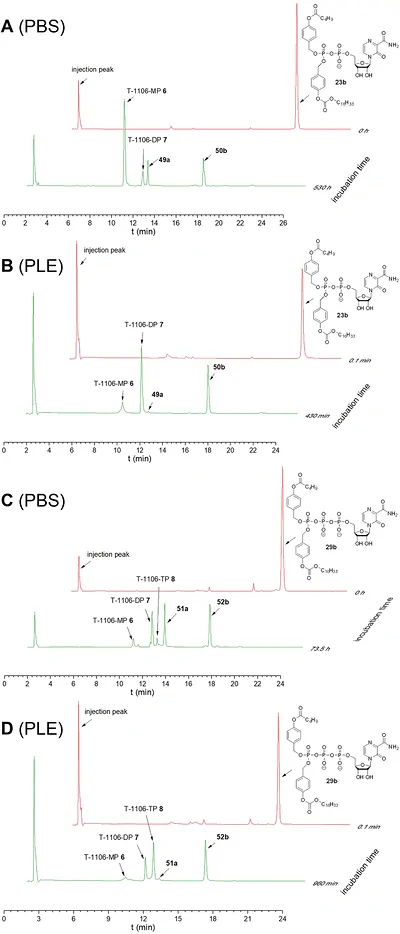
Hydrolysis of T‐1106‐prodrugs **23b** and **29b** in PBS (25 mM, pH 7.3, panel A for **23b** and panel B for **29b**) and with PLE (5.6 U/mL, panel C for **23b** and panel D for **29b**). The hydrolysis products formed after the indicated incubation time are shown in the green colored chromatographic trace, with the red colored chromatogram showing the starting compounds at time zero.

The chemical hydrolysis experiments in PBS at pH 7.3 showed that Di*PP*ro‐prodrug **23b** (AB‐C4;ACB‐C16) and Tri*PPP*ro‐prodrug **29b** (AB‐C4;ACB‐C16) can bypass two or three intracellular phosphorylation steps, respectively, by delivering T‐1106‐DP **7** or T‐1106‐TP **8** (pathways a_1_ and a_2_, Scheme 4 and 5, upper sections). This aligns with our previous results [24] for two structurally similar T‐1106 prodrugs, i.e. Di*PP*ro‐compound (AB‐C4;AB‐C14) and Tri*PPP*ro‐compound (AB‐C4;AB‐C14) (Figure 3). For comparison, the hydrolysis half‐life for the previously reported compound **22** was 30 h in PBS pH 7.3 [9, 24].

Compounds **23b** and **29b** showed a marked difference in hydrolysis time (Figure 3A,C), despite their identical masking group composition (both AB‐C4/ACB‐C16). Diphosphate prodrug **23b** still showed small but detectable amounts after 300 h, while triphosphate prodrug **29b** was already hydrolysed to very small amounts after 74 h. Surprisingly, the chemical stability of the AB‐ and ACB‐groups was almost identical, yielding virtually the same amounts of intermediates **49a** and **50b** (Figure 3A), and **51a** and **52b** (Figure 3C).

Besides T‐1106‐DP **7** or T‐1106‐TP **8**, the major hydrolysis products were T‐1106‐MP **6** for **23b** and T‐1106‐DP **7** for **29b**. Hence, besides the desired masking group cleavage, significant additional cleavage took place of the α,β‐phosphate bond in **23b** (via pathway b, Scheme 4, upper section) and the β,γ‐phosphate bond in **29b** (via pathway b, Scheme 5, upper section). This unwanted reaction under chemical hydrolysis conditions has also been observed in other Di*PP*ro‐ or Tri*PPP*ro‐compounds.

#### 2.5.2 Hydrolysis by Pig Liver Esterase (PLE)

Compared to the chemical hydrolysis (Figure 3A,C), we observed more rapid hydrolysis by PLE for Di*PP*ro‐prodrug **23b** (*t*_1/2_ = 1.4 h, 33‐fold faster) and Tri*PPP*ro‐prodrug **29b** (*t*_1/2_ = 1.84 h, 6‐fold faster), yielding the intermediates **49a** and **50b**, and **51a** and **52b**, and subsequently releasing T‐1106‐DP **7** and T‐1106‐TP **8**, respectively (Figure 3B,D). Both starting compounds were hydrolysed within the same time period. In contrast to the chemical hydrolysis, we observed substantially different PLE‐stability for the two masking groups, since the AB group was cleaved much faster than the ACB group. Interestingly, Di*PP*ro‐compound **23b** generated only traces of the nucleoside monophosphate, indicating very limited unwanted cleavage of the α,β‐phosphate bond. On the other hand, for Tri*PPP*ro‐compound **29b**, we again detected minor amounts of the nucleoside diphosphate.

### 2.6 Series 2: Di*PP*ro‐prodrugs **24** and **25**, and Tri*PPP*ro‐prodrugs **30** and **31**, Bearing One Biodegradable Group and One Non‐Cleavable Alkyl Group

#### 2.6.1 Hydrolysis in PBS at pH 7.3

**FIGURE 4:**
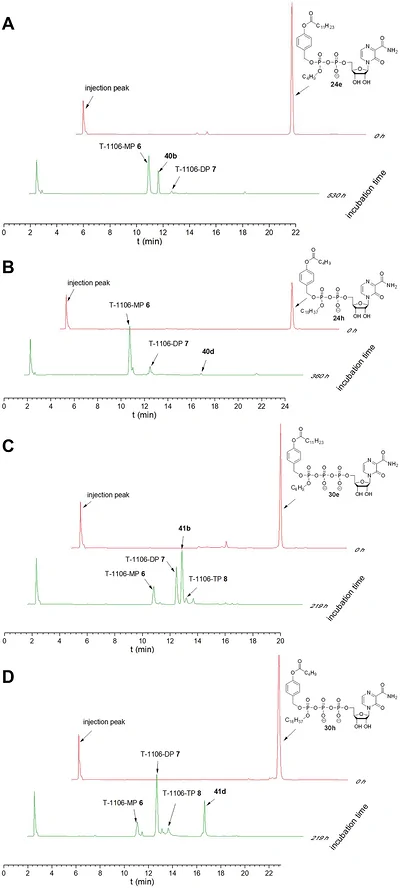
Hydrolysis of compounds **24e**, **24h**, **30e**, and **30h** in PBS (25 mM, pH 7.3). The hydrolysis products formed after the indicated incubation time are shown in the green colored chromatogram, with the red colored trace showing the starting compounds at the starting point.

We previously reported how β‐modified nucleoside diphosphate derivatives and γ‐modified nucleoside triphosphate derivatives are released from our second generation Di*PP*ro‐compounds and Tri*PPP*ro‐compounds [44]. Scheme 4 (middle section) and Scheme 5 (middle section) show the conversion scheme for Di*PP*ro‐prodrugs **24h** and Tri*PPP*ro‐prodrugs **30h**, respectively. Di*PP*ro‐prodrug **25** and Tri*PPP*ro‐compound **31** follow the same preferred hydrolysis pathways (not shown in Scheme 4, 5). Under chemical conditions in PBS at pH 7.3, these four prodrugs exhibited half‐lives between 17 and 97 h, releasing the β‐mono‐alkylated T‐1106‐DP derivatives **40** and **47** (Figures 4A,B  and S1; Supporting Information) and γ‐mono‐alkylated T‐1106‐TP derivatives **41** and **48** (Figures 4C,D  and S2; Supporting Information), as well as the nucleoside mono‐ and diphosphate forms. As expected from other studies using different nucleoside analogues, the half‐lives are considered to be sufficiently high for usage. However, some of the measured differences can not be explained, for example, **24b** and **24c** vs. **30b** and **30c** which comprise the identical combination of masking groups applied to the di‐ or the triphosphate of T‐1106, although they are still in the same range.

For example, incubation in PBS of Tri*PPP*ro‐compound **30h** (Figure 4D), generated the γ‐mono‐alkylated T‐1106‐TP compound **41d** (second preferred product), T‐1106‐DP **7** (predominant product), T‐1106‐MP **6** (minor product) and T‐1106‐TP **8** (very small amount). This validates that γ‐(alkyl)‐T‐1106‐TP **41** may follow pathway c (Scheme 5) (Figure 4C,D), which differs from our previous findings with the analogous pronucleotide of d4T‐TP [37]. In contrast, T‐1106‐TP **8** was not at all formed from γ‐alkyl*phosphonate* T‐1106‐TP prodrug **31** (Figure S2; Supporting Information). The marked influence of the terminal alkyl*phosphate* or alkyl*phosphonate* group, respectively, was also seen for Di*PP*ro‐prodrugs **24** *versus* prodrug **25**, which generated T‐1106‐MP **6** and β‐mono‐alkylated T‐1106‐DP derivatives **40** and **47** (Figures 4A (**24e**), 4B (**24h**) and S1 (**25**); Supporting Information). However, T‐1106‐DP **7** (pathway c, Scheme 4) was solely formed from the alkyl*phosphate* containing compounds **24**, and not from the alkyl*phosphonate* containing compound **25**.

When the monoalkylated T‐1106‐DP and ‐TP were hydrolysed we noticed an unexpected difference between the alkyl*phosphate* and alkyl*phosphonate* derivatives. The methyl *phosphate* ester of T‐1106‐DP **40a** proved to be stable for 500 h (Table 1). The same was observed for the C18‐ester **40d** (a small amount of an impurity was already present at the starting point; Figure S5) and the corresponding C18‐ester of the triphosphate **41d** (Figure S7). In contrast, the C18‐alkyl*phosphonate* counterparts **47** (Figure S6) and **48** (Figure S8) showed considerable formation of the nucleoside‐monophosphate or ‐diphosphate, resulting in complete hydrolysis of these compounds after 530 h. The reason for this unexpected hydrolysis is still unknown.

#### 2.6.2 Hydrolysis by PLE

**FIGURE 5:**
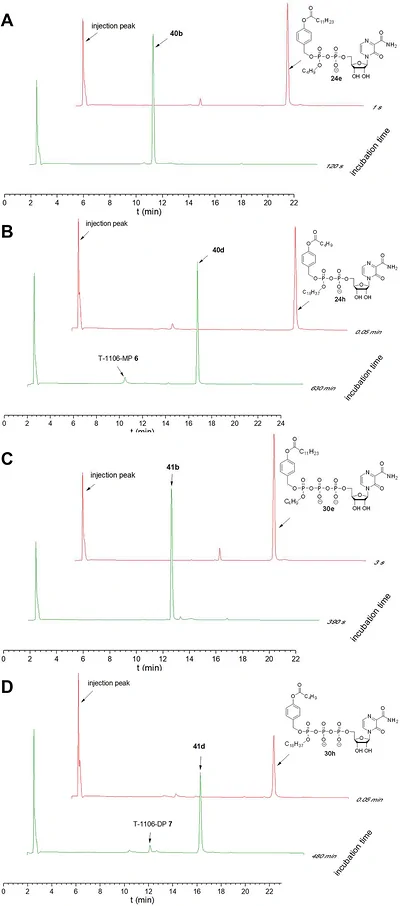
Hydrolysis of T‐1106‐compounds **24e**, **24h**, **30e,** and **30h** upon incubation with PLE (5.6 U per mL). The products formed after the indicated incubation time are shown in the green colored chromatogram, with the red colored chromatographic trace showing the starting compounds at the starting point.

When the Di*PP*ro‐compounds **24a**‐**h** and **25** and Tri*PPP*ro‐compounds **30a**‐**h** and **31** were incubated with PLE (5.6 U per mL), their hydrolysis half‐lives (t*_1/2_* = <0.01‐1.7 h, Table 1) were significantly lower than those determined in PBS buffer (e.g. 3000‐fold difference in t*_1/2_* for **24b**). During this fast hydrolysis, the four types of pronucleotides mainly formed the β‐mono‐alkylated intermediates **40a‐d** and **47** (from **24a‐d** and **25**, respectively) and γ‐mono‐alkylated intermediates **41a**,**c**,**d** and **48** (from **30** and **31**, respectively) (Figure 5A–D, respectively, and Figures S3, S4 Supporting information). The accumulation of the β/γ‐mono‐alkylated intermediates agrees with the prominent stability of these compounds towards PLE‐hydrolysis (t*_1/2_* >40 h; Table 1). This is most probably attributable to the repulsion between their two or three negative charges and the approaching nucleophile (Figures S5–S8 Supporting Information).

Next to forming the β/γ‐mono‐alkylated intermediates, compounds **24**, **25**, **30** and **31** generated very small amounts, if any, of T‐1106‐MP **6** (Figure 5B) or T‐1106‐DP **7** (Figure 5D). We saw no evidence for formation of T‐1106‐DP **7** from compound **25** (Figure S3; Supporting Information) and T‐1106‐TP **8** from compound **31** (Figure S4; Supporting Information). For example, the PLE‐mediated hydrolysis of Tri*PPP*ro‐compound **30h** (Figure 5D) showed a substantial quantity of γ‐(alkyl‐C18)‐T‐1106‐TP **41d**, marginal amounts of T‐1106‐MP **6** and T‐1106‐DP **7**, and no traces of T‐1106‐TP **8**. We assume that products **6** and **7** were not only formed from the parent pronucleotides, but also from intermediates **40** and **47**, and **41** and **48**, via a nucleophilic reaction at the β‐phosph(on)ate (pathway b, Scheme 4, middle section) or γ‐phosph(on)ate (pathway b, Scheme 5, middle section).

Compounds bearing long β‐ or γ‐alkyl‐moieties showed markedly higher stability as compared to shorter and therefore less lipophilic groups (see **24e** vs. **24h** or **30e** vs. **30h**; Figure 5). The triphosphate analogues were less stable than their diphosphate counterparts (see **24e** vs. **30e** or **24h** vs. **30h**; Figure 5).

The cleavage of compounds **24**, **25**, **30**, and **31** by PLE showed remarkable effect of the AB‐moiety (Figures 5 and S3, S4, Supporting Information), in terms of hydrolysis by pathway a_3_ in Scheme 4, and pathway a_3_ in Scheme 5. Apparently, once the esterase reaction proceeds so fast, that the concurrent and unwanted rupture of the phosphoroanhydride bond did not happen. This behaviour nicely aligns with our PLE hydrolysis results for γ‐(AB‐C4;alkyl‐C18)‐d4T‐TP [37] and γ‐(AB‐C4)‐γ‐C‐(alkyl‐C18)‐d4T‐TP, suggesting that these may be general properties [38].

#### 2.6.3 Hydrolysis in CEM/0 Cell Extracts

**FIGURE 6:**
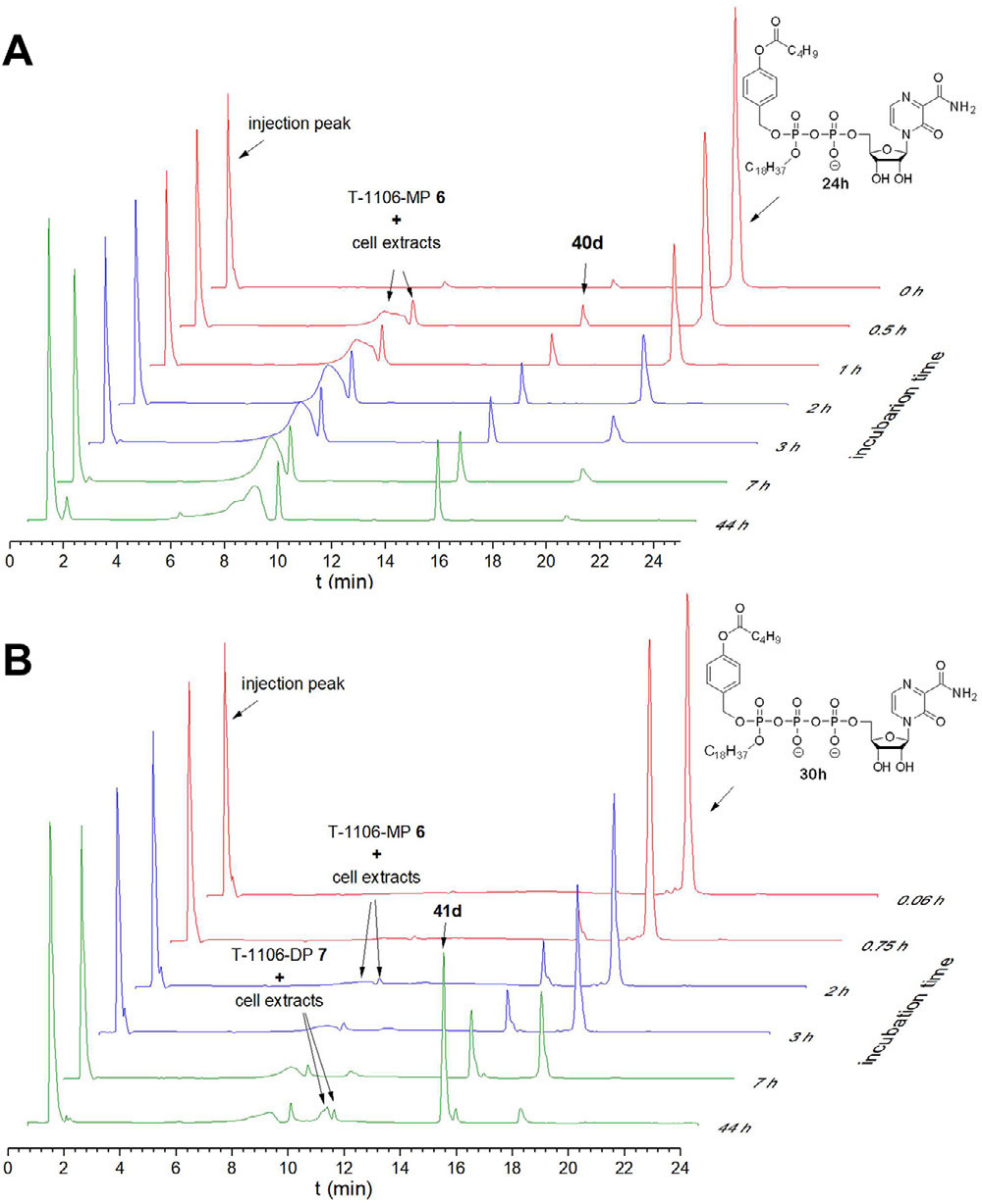
Hydrolysis of T‐1106‐Di*PP*ro‐compound **24h** and T‐1106‐Tri*PPP*ro‐compound **30h** in CEM/0 cell extract.

When we next incubated the series 2 Di*PP*ro‐compound **24h** and Tri*PPP*ro‐compound **30h** in crude extract of CEM/0 cells, the half‐lives were 0.9 and 2.5 h, respectively. The hydrolysis profile of compound **24h** (Figure 6A) shows that, after 20 h, all the starting compound had disappeared, with intermediate **40d** being the main product. However, as the half‐life in cell extract was markedly higher when compared to that in PLE (which is potentially related to different concentrations of esterases or lipases), we again detected the monophosphate product. In contrast, compound **30h** (Figure 6B) almost exclusively generated intermediate **41d**, with minor formation of T‐1106‐MP **6** and T‐1106‐DP **7** (although completing the reaction took about 44 h) and no formation of T‐1106‐TP **8**.

### 2.7 Series 3: T‐1106‐Di*PP*ro‐compounds **26** and **27** and T‐1106‐Tri*PPP*ro‐compounds **32** and **33** Bearing Two Non‐Cleavable Alkyl Groups

**FIGURE 7:**
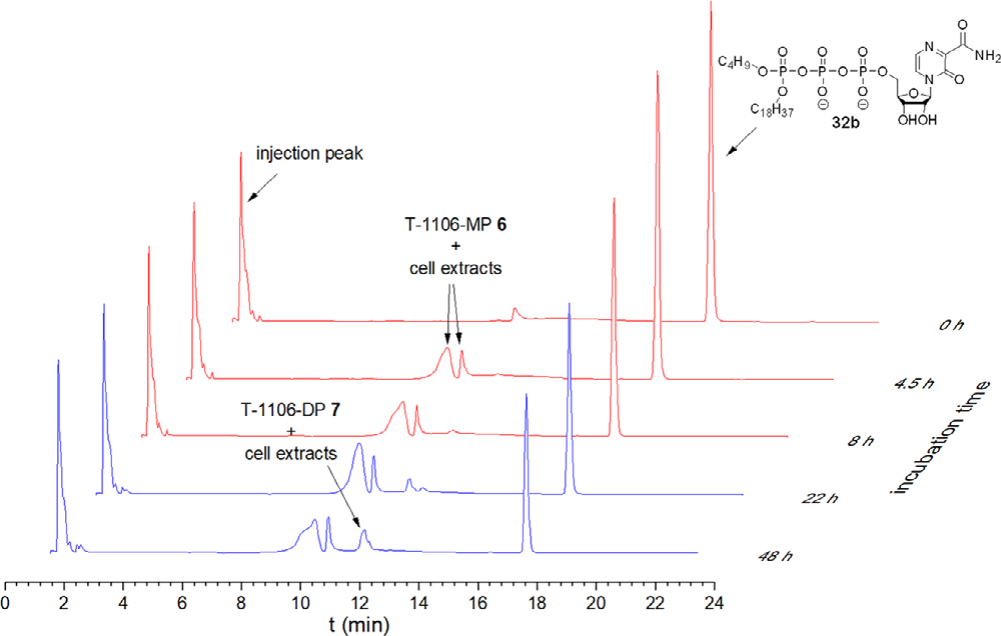
Hydrolysis of T‐1106‐Tri*PPP*ro‐compound **32b** in CEM/0 cell extracts.

Next, we conducted hydrolysis experiments with Di*PP*ro‐compounds **26** and **27**, and Tri*PPP*ro‐compounds **32** and **33**, which bear *two* non‐cleavable alkyl residues. As anticipated, the absence of (enzymatic) cleavage sites such as in the acyloxybenzyl moieties of series 1 and 2 compounds rendered these four compounds highly stable, with half‐lives >200 h in PBS and >40 h in PLE, and >10 h for compound **32b** in CEM/0 cell extract. The high stability also makes these compounds highly resistant to dephosphorylation. Notably, we also observed the formation of T‐1106‐MP **6** and T‐1106‐DP **7**, but not of T‐1106‐TP **8** or the γ‐mono‐alkylated compound **41** (Figure 7).

Thus, upon incubation in PBS, β‐(alkyl‐C4;alkyl‐C18)‐T‐1106‐DP **26b** (Figure S9; Supporting Information) and γ‐(alkyl‐C4)‐*γ*‐C‐(alkyl‐C18)‐T‐1106‐TP **33b** (Figure S10; Supporting Information) underwent chemical cleavage at the terminal β‐dialkylphosphate or γ‐dialkylphosphonate moiety. Possibly, the neutral β,γ‐phosph(on)ate moiety may enable a nucleophilic attack at the terminal phosphate group. Although these properties appear interesting from the chemistry point of view, they hinder the potential development of these pronucleotides as antivirals, due to undesirably long times to reach the active form (up to one month).

### 2.8 Hydrolysis in Human Plasma

**FIGURE 8:**
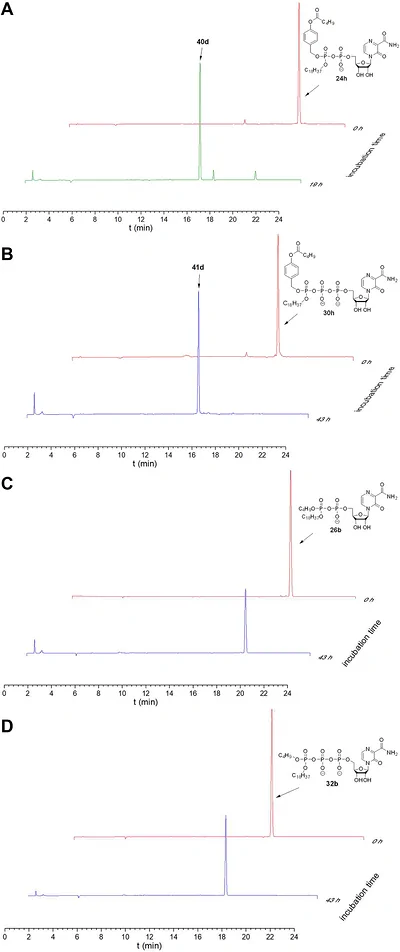
Hydrolysis of T‐1106‐Di*PP*ro‐compounds **24h** and **26b** and T‐1106‐Tri*PPP*ro‐compounds **30h** and **32b** upon incubation in human plasma.

Finally, eight Di*PP*ro‐ and Tri*PPP*ro‐compounds were incubated in human plasma. With *t*_1/2_ values between 0.14 and 3.1 h (Table 2), series 1 compounds **23a** and **29a** and series 2 compounds **24e**, **24h**, **30e**, and **30h** were markedly less stable than the series 3 compounds **26b** and **32b** (*t*_1/2_>10 h). Compounds **24** and **30h** were hydrolysed in human plasma within 19 or 43 h (Figure 8), with formation of the β/γ‐mono‐alkylated hydrolysis products **40d** and **41d** but, surprisingly, no T‐1106‐MP **6**, T‐1106‐DP **7** nor T‐1106‐TP **8**. The free nucleotides were also not formed from the dialkylated compounds **26b** and **32b** (Figure 8C,D). In these assays with human plasma, we noticed a decrease in the amount of starting material. This might be due to unspecific protein binding, which might also protect the compounds against hydrolysis.

**TABLE 2:** Hydrolysis half‐lives of T‐1106‐pronucleotides from series 1, 2, and 3 in human plasma.

Compound	Human plasma	Compound	Human plasma
	*t*_1/2_ [h]		*t*_1/2_ [h]
**23a**	0.92	**29a**	0.14
**24e**	1.25	**30e**	1.09
**24h**	3.14	**30h**	2.92
**26b**	>10	**32b**	>20

Assay conditions are given in the Experimental Section.

### 2.9 Antiviral Data

The T‐1106 pronucleotides were evaluated, in parallel with the parent nucleoside T‐1106 **9**, for antiviral activity against different viruses, specifically: influenza A virus (IAV; subtypes H1N1 and H3N2) and influenza B virus (IBV); human coronavirus (HCoV)‐229E and ‐OC43; respiratory syncytial virus (RSV); yellow fever virus (YFV); Zika virus; Sindbis virus; Semliki forest virus; HIV‐1 and ‐2; varicella zoster virus (VZV); and herpes simplex virus (HSV)‐1 and ‐2.

**FIGURE 9:**
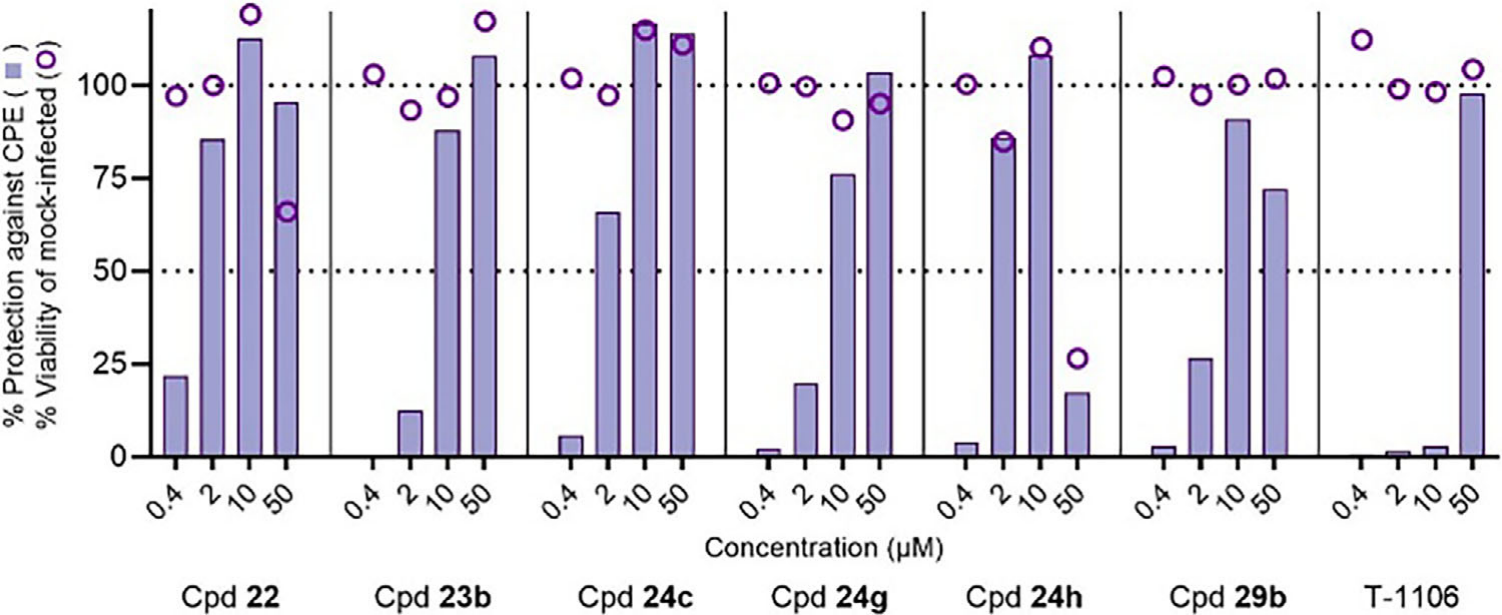
Anti‐influenza virus (H1N1) activity of six pronucleotides and parent T‐1106 **9** in MDCK cells. The bar graphs show the protection against viral CPE, and the circle symbols show the viability of mock‐infected cells. Data points are the mean values of two independent tests.

As summarized in Table 3, several of the T‐1106 pronucleotides showed nice anti‐influenza virus activity in MDCK cells, with similar potency for the three IAV/IBV strains tested. Figure 9 shows the dose‐dependent inhibition of H1N1 virus for six prodrugs and the parent nucleoside T‐1106 **9**. The EC_50_ values (i.e. average for the three strains) were ∼0.8 µM for compounds **22** and **24h**; ∼1.2 µM for compounds **25**, **40d** and **47**; 1.9‐4.0 µM for compounds **23b**, **24c**, **24g**, **28** and **30h**; and 8.1 µM for compound **29b**. The activity of these pronucleotides was markedly higher than that of the parent T‐1106 **9**, which had EC_50_ values of 23 µM (IAV) and 6.6 µM (IBV). Among the 11 active pronucleotides, 5 compounds (i.e. **22**, **23b**, **24c**, **24g**, and **29b**) had a CC_50_ value >50 µM, the highest concentration tested. Hence, the selectivity index ( = ratio of CC_50_ to EC_50_) ranged from >62 (compound **22**) to >14 (compound **23b**). In few cases also an increase in cytotoxicity was observed particularly in Hep2 cells. However, for the T‐1106 pronucleotides that did have a CC_50_ value in MDCK cells below 50 µM, there was no correlation with the anti‐influenza virus activity. For instance, compounds **26b** and **27b** were the two most toxic compounds in MDCK cells (CC_50_ = 0.6 µM), while having no anti‐influenza virus activity.

**TABLE 3:** Evaluation of the T‐1106 pronucleotides for antiviral activity and cytotoxicity.

Compound		Antiviral activity: EC_50_ ^a^ in µM					Cytotoxicity: CC_50_ ^b^ in µM			
		IAV H1N1	IAV H3N2	IBV	HCoV‐OC43	HCoV‐229E				
		in MDCK cells			in HEL299 cells		MDCK	HEL299	Hep2	VeroE6
**Series 1: two biodegradable groups**										
Di*PP*ro	**22**	0.80	1.0	0.50	>50	41	>50	>50	26	>50
	**23b**	5.7	3.9	0.80	7.6	>50	>50	>50	>50	>50
Tri*PPP*ro	**28**	2.8	3.2	3.1	7.1	>50	28	>50	28	>50
	**29b**	3.6	18	2.7	25	>50	>50	>50	>50	>50
**Series 2: one biodegradable group and one non‐cleavable alkyl group**										
Di*PP*ro	**24b**	>50	>50	>50	32	>50	24	>50	>50	>50
	**24c**	1.8	2.4	1.4	>50	48	>50	>50	>50	>50
	**24e**	>50	>50	>50	>50	>50	7.6	>50	>50	>50
	**24g**	3.6	4.4	3.9	3.9	41	>50	>50	>50	>50
	**24h**	1.0	1.0	0.70	2.9	48	10	>50	28	>50
	**25**	1.0	1.0	1.5	7.1	>50	4.3	28	5.1	23
Tri*PPP*ro	**30b**	>50	>50	>50	25	>50	25	>50	>50	>50
	**30c**	>50	>50	>50	>50	>50	6.1	33	31	30
	**30e**	>50	>50	>50	>50	>50	22	>50	>50	>50
	**30h**	>50	3.4	3.9	5.5	>50	30	>50	28	>50
	**31**	>50	>50	>50	6.5	>50	17	31	18	>50
**Series 3: two non‐cleavable alkyl groups**										
Di*PP*ro	**26b**	>50	>50	>50	4.9	>50	0.60	28	21	27
	**27b**	>50	>50	>50	9.6	>50	0.60	43	23	26
Tri*PPP*ro	**32b**	>50	>50	>50	>50	>50	4.1	>50	25	>50
	**33b**	>50	>50	>50	>50	>50	3.6	>50	26	>50
**Only one non‐cleavable alkyl group at the terminal phosphate**										
Di*PP*ro	**40d**	1.0	1.5	0.90	37	38	4.7	>50	>50	>50
	**47**	1.2	1.6	1.2	>50	>50	4.7	21	5.2	30
Tri*PPP*ro	**41d**	>50	>50	>50	15	>50	3.0	>50	27	>50
	**48**	>50	>50	>50	>50	>50	>50	>50	>50	34
Parent T‐1106^c^	**9**	23	23	6.6	>50	>50	>50	>50	>50	>50
Remdesivir		n.d.^d^	n.d.^d^	n.d.^d^	0.06	0.06	n.d.^c^	>10	>10	>10
Ribavirin		15	14	3.8	159	79	>250	>250	>250	>250

The antiviral evaluation was conducted as reported before [51, 52, 53, 54, 55, 56], and using as the highest tested concentration: 50 µM for the pronucleotides, 10 µM for remdesivir and 250 µM for ribavirin.

^a^ 50% effective concentration, that is, concentration to achieve 50% protection against virus‐induced CPE, as assessed by MTS cell viability assay;

^b^ 50% cytotoxic concentration, that is, concentration causing 50% reduction in cell viability, as assessed by MTS cell viability assay.

^c^ For T‐705 and T‐1105, our published EC_50_ values [24] against IAV in MDCK cells are 26 and 5.4 µM, respectively.

^d^ n.d., not done. Data are the mean of two independent tests. To calculate the EC_50_ and CC_50_ values from the MTS‐OD values, we assumed a semi‐log dose response and conducted interpolation between the two concentrations yielding more or less than 50% response. The detailed formulas can be found in ref [53].

Comparison of the anti‐influenza virus activities with the hydrolysis half‐lives for PBS and PLE (Table 1), revealed a trend that pronucleotides with very low (t*_1/2_* <1 h; e.g. compounds **24b** and **30e**) or very high (t*_1/2_* >40 h; e.g. compounds **26b**, **32b**, **33b**) esterase‐stability, failed to execute antiviral effect. Reciprocally, several prodrugs such as **24h** had a t*_1/2_* of ∼1.7 h for PLE and 97 h for PBS and favorable EC_50_ value. The eight prodrugs (i.e. **22**, **23b**, **24c, 24g**, **24h**, **25**, **28**, and **29b**) with the most appealing anti‐influenza virus activity all belonged either to series 1 or series 2, with **22**, **23b**, **24c**,**g**,**h** and **25** being Di*PP*ro, and **28** and **29b** being Tri*PPP*ro compounds. It appears that compounds having a retention time on the HPLC column in the range of 18 to 20 min (which is at least a qualitative measure of the lipophilicity) proved to be antivirally active. Lower lipophilicity led to inactive compounds.

Interestingly, while the parent nucleoside T‐1106 **9** was not active against Human coronavirus OC43 (HCoV‐OC43), a few pronucleotides (i.e. compounds **24g**, **24h**, **26b** and **30h**) showed an EC_50_ value below 5.5 µM. Compounds **23b**, **25**, **27b**, **28**, **31** and **41d** had an EC_50_ value between 7 and 15 µM, and **24b**, **29b**, **30b** and **40d** had weak activity (i.e. EC_50_ value between 20 and 37 µM). Neither of these pronucleotides had meaningful activity (i.e. EC_50_ below 15 µM) against Human coronavirus 229E (HCoV‐229E), the other coronavirus tested. Still, compound **24h** (a series 2 Di*PP*ro compound) had an attractive profile in combining high potency for influenza virus and HCoV‐OC43, with weak activity against HCoV‐229E.

Surprisingly, a remarkable effect was observed for compounds that comprise only one masking group at the terminal phosphate. Compounds **40d** and **47** showed some interesting antiviral activity although they have an additional charge at the diphosphate moiety as compared to the double modified prodrugs. Thus, their lipophilicity is much lower but still the compounds are active. On the other hand, no activity whatsoever was seen for the triphosphate counterparts comprising the same modification (**41d** and **48**). Though still a hypothesis, we presume that the markedly higher hydrophilicity of these compounds may prevent their cellular uptake.

Neither of the T‐1106 pronucleotides showed activity against Respiratory syncytial virus (RSV); Sindbis virus and Semliki forest virus (family: *Togaviridae*); and Yellow Fever Virus (YFV) and Zika virus (family: *Flaviviridae)* (data not shown). For YFV, the T‐1106 nucleoside was reported to have no meaningful activity in Vero cells, while it did confer strong protection in a lethal YFV hamster model [13]. Since the Di*PP*ro and Tri*PPP*ro compounds did not solve the lack of anti‐YFV activity in Vero cells, it appears that another factor than inefficient phosphorylation might be at the basis.

Since the RNA viruses were tested in different cell lines, i.e. MDCK, HEL299, Hep2 or Vero cells, we were able to more broadly assess the cytotoxicity. The compounds showing favourable anti‐influenza virus activity (see above), had no cytotoxicity (compounds **23b**, **24c**, **24g** and **29b**: CC_50_>50 µM) or weak cytotoxi‐city (compounds **22** and **24h**: CC_50_ ∼27 µM in Hep2 cells).

Finally, since the T‐1106 moiety carries a ribose sugar, neither of the T‐1106 prodrugs showed activity in cytopathic effect (CPE) assays with viruses that carry a DNA‐dependent DNA polymerase (i.e. HSV‐1, evaluated in HEL299 cells) or reverse transcriptase (i.e. HIV‐1 and HIV‐2, evaluated in CEM/0 cells). For HIV‐1 and 2 all compounds including the parent T‐1106 showed no inhibitory effect but also proved to be non‐toxic in CEM/0‐cells (Table S1; Supporting information). These compounds proved inactive in primer‐extension inhibition assays [7, 48] with HIV‐1 RT or human polymerases α, β or γ (assays performed with T‐1106‐DP **7**, T‐1106‐TP **8**, and the pronucleotides **26**, **27**, **32**, **33**, **40** and **41**, Figure S11; Supporting Information).

## 3 Conclusions

In this report, we describe the successful application of our Di*PP*ro‐ and Tri*PPP*ro‐nucleotide prodrug technology to solve the inefficient activation of the nucleobase analogue T‐1105 and its ribonucleoside T‐1106. We covered the chemical synthesis of three series of prodrugs, their chemical and metabolic pathways to release T‐1106‐diphosphate or ‐triphosphate, and their antiviral evaluation in cell culture.

Using a new method, we obtained T‐1106‐MP **6** in good yield (overall yield from the pyrazinemethylcarboxylate analogue **10**, 82%, 2 steps). Next use the (Fm;Fm)*‐H*‐phosphonate route for the synthesis of T‐1106‐DP **7** was adapted towards two deprotection steps. Then, P(V) chemistry reagents were applied to synthesize the T‐1106‐Di*PP*ro‐ and Tri*PPP*ro‐compounds **23–33** bearing different kinds of masking groups.

**SCHEME 6:**
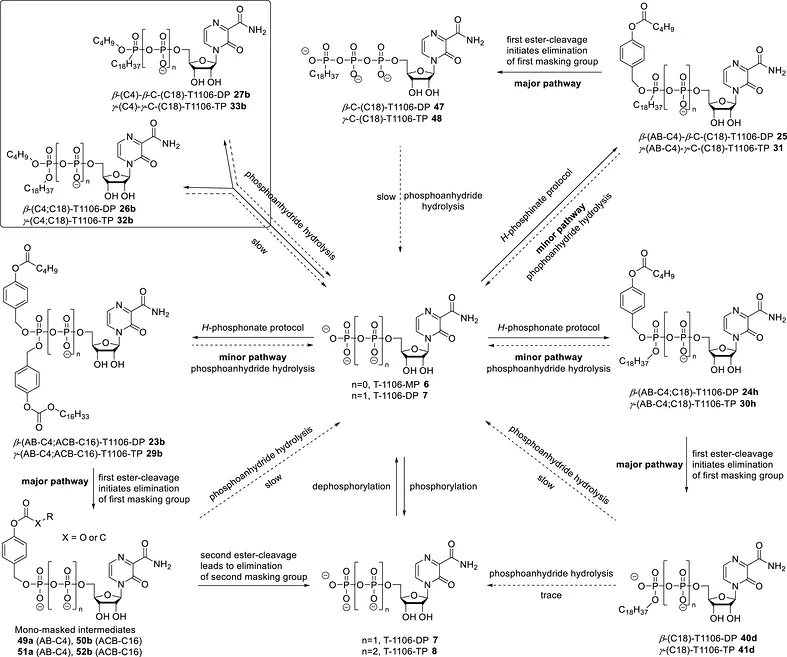
Summary of the compound's metabolic characteristics.

Our extensive analysis of the prodrugs’ hydrolysis by chemical or enzyme‐triggered pathways is summarized in Scheme 6. For series 2 (i.e. T‐1106‐Di*PP*ro‐ and Tri*PPP*ro‐compounds **24**, **25**, **30** and **31**), we observed significant hydrolysis to the β‐ and γ‐mono‐masked intermediates **40**, **41**, **47**, and **48**, which comprise one non‐cleavable alkyl group. The minor formation of T‐1106‐MP **6** (for the T‐1106‐Di*PP*ro‐compounds **24** and **25**) and T‐1106‐DP **2** (for the T‐1106‐Tri*PPP*ro‐compounds **30** and **31**) is likely attributed to chemical cleavage of the β‐ or γ‐phosph(on)ate. In contrast, very high metabolic stability was seen with the pronucleotides bearing two non‐cleavable alkyl groups ( = series 3; compounds **26**, **27**, **32**, and **33**).

Thanks to their balanced metabolic stability several of the series 1 and series 2 pronucleotides showed favourable activity against influenza virus, with markedly lower EC_50_ values than the parent nucleoside T‐1106 **9**. A few also showed activity against HCoV‐OC43. Surprisingly, in contrast to corresponding d4T‐(D)TP pronucleotides the compound series 3 had no beneficial effect on the antiviral activity profile. Hence, this study further underlines the high relevance of our Di*PP*ro‐ and Tri*PPP*ro technology to bypass the metabolic activation bottleneck of antiviral nucleoside analogues and boost their therapeutic potential. Nevertheless, applying these three pronucleotide systems to T‐1106 did not lead to a markedly broader antiviral activity spectrum. This might indicate that, apart from the enzymes of influenza and coronaviruses, the other viral polymerases addressed by our virus panel may not be adequately inhibited by T‐1106‐TP.

## Conflicts of Interest

The authors declare no conflicts of interest.

## Supporting information




**Supporting file 1**: chem71181‐sup‐0001‐SuppMat.pdf

## Data Availability

The data that support the findings of this study are available in the Supporting Information of this article.
