# Supplementary material for: Membrane‐Permeable Nucleoside T‐1106 Diphosphate and Triphosphate Analogues as Antiviral Pronucleotides
Source: Chemistry. 2026 May 27;32(34):e71181. doi: 10.1002/chem.71181 (PMC13569937; doi:10.1002/chem.71181)
Supplement: Supplementary file 1 [file CHEM-32-e71181-s001.pdf]

Supporting Information

Membrane-Permeable Nucleoside T-1106 Diphosphate and Triphosphate Analogues as Antiviral Pronucleotides

Chris Meier,*[a] Duan Li,[b] Leentje Persoons,[c] Graciela Andrei,[d] Robert Snoeck,[d] Dominique Schols[d], Lieve Naesens, [d] Xiao Jia*[a,b]

[a] Prof. Dr. C. Meier,
Organic Chemistry, Department of Chemistry, Faculty of Mathematics, Informatics and Natural Sciences, Universität Hamburg, Martin-Luther-King-Platz 6, D-20146 Hamburg, Germany
and
Centre for Structural Systems Biology (CSSB), Hamburg, DESY Campus, Notkestrasse 85, 22607 Hamburg, Germany
E-mail: chris.meier@chemie.uni-hamburg.de; https://orcid.org/0000-0002-8323-5635

[b] M. Sc. D Li,
State Key Laboratory of Discovery and Utilization of Functional Components in Traditional Chinese Medicine, School of Pharmaceutical Sciences, Guizhou Medical University, Guizhou 550025, China.
and
Zhongshan Institute for Drug Discovery, Shanghai Institute of Materia Medica, Chinese Academy of Sciences, Zhongshan, 528400, China
Prof. Dr. X. Jia
Organic Chemistry, Department of Chemistry, Faculty of Mathematics, Informatics and Natural Sciences, Universität Hamburg, Martin-Luther-King-Platz 6, D-20146 Hamburg, Germany
and
State Key Laboratory of Discovery and Utilization of Functional Components in Traditional Chinese Medicine, School of Pharmaceutical Sciences, Guizhou Medical University, Guizhou 550025, China.
and
State Key Laboratory of Drug Research, Shanghai Institute of Materia Medica, Chinese Academy of Sciences, Shanghai 201203, China
and
Zhongshan Institute for Drug Discovery, Shanghai Institute of Materia Medica, Chinese Academy of Sciences, Zhongshan, 528400, China
E-Mail: jiaxiao@simm.ac.cn

[c] L. Persoons

Molecular Genetics and Therapeutics in Virology and Oncology, Department of Microbiology and Immunology and Transplantation, Rega Institute, KU Leuven, Herestraat 49, 3000 Leuven, Belgium
Email: leentje.persoons@kuleuven.be
[d] Prof. Dr. G Andrei, Prof. Dr. R Snoeck, Prof. Dr. D. Schols, Prof. Dr. L Naesens
Laboratory of Virology and Chemotherapy, Department of Microbiology and Immunology and Transplantation, Rega Institute for Medical Research, KU Leuven, Herestraat 49, 3000 Leuven, Belgium.
E-mails: graciela.andrei@kuleuven.be, robert.snoeck@kuleuven.be, dominique.schols@kuleuven.be and lieve.naesens@kuleuven.be

Contents

Experimental Section S3

Part 1. General Procedure: Preparation of T-1106-compounds **22-33** (NH_4^+ form)........ S3

Part 2. Synthesis and Characterization. S4

Part 3. Hydrolysis in PBS at pH 7.3 or by pig liver esterase........ S24

Part 4. Hydrolysis in crude CEM/0 cell extract or in human plasma S25

Part 5. Figure S1-S10. HPLC profiles of T-1106-Di*PP*ro- and Tri*PPP*ro-compounds after incubation in PBS (pH 7.3) and PLE........ S25

Part 6. Antiviral procedures........ S29

Table S1. Antiviral data against human HIV-1/2 .... S29

Part 7. Primer-extension assays........ S30

Figure S11. Primer extension assay using HIV-RT and polymerases α,β,γ. S30

Part 8. Spectral data for new compounds S32

Reference........ S109

Experimental Section

General: All chemical reactions were carried out under nitrogen atmosphere (N_2) and anhydrous conditions. All anhydrous solvents (CH_3CN, pyridine, TEA, and THF) were purchased from Acros Organics (Extra Dry over molecular sieves). Phosphate buffer saline (PBS) was purchased from Sigma-Aldrich. Ultrapure water was produced by a Sartorius Aurium® pro (Sartopore 0.2 μm, UV). CH_3CN (HPLC grade) was purchased from VWR. All other organic solvents (ethyl acetate, petroleum ether 50-70, CH_2Cl_2, acetone, and CH_3OH) were purchased technical grade and distilled prior to use. Commercially available reagents (such as FmOH and NCS) and solvents (such as TMP, and $POCl_3$) were used without further purification. General flash column chromatography was performed with silica gel 60 M (0.04-0.063mm, Macherey-Nagel). For reversed phase automated flash chromatography, an Interchim Puriflash 430 was used in combination with Chromabond® Flash RS40 C_{18}ec. Analytical thin-layer chromatography (TLC): For thin layer chromatography Macherey-Nagel pre-coated TLC sheets Alugram® Xtra SIL G/UV254 were used. High Performance Liquid Chromatography (HPLC) measurements were carried out using a VWR-Hitachi LaChromElite HPLC system (L-2130, L-2200, L-2455) and EzChromElite software. TBAA buffer solvents: 2 mM tetra-*n*-Butylammonium acetate solution (pH 6.3). All T-1106-Di*PP*ro- and Tri*PPP*ro-compounds were analyzed for purity by NMR spectroscopy and RP-HPLC (UV). Purity of T-1106-Di*PP*ro- and Tri*PPP*ro-compounds was ≥95%. HPLC method: Nucleodur 100-5 C18ec; 0-20 min: TBAA buffer/CH_3CN gradient (5-80%); 20-30 min: buffer/CH_3CN (80%); 30-33 min: buffer/CH_3CN (80-5%); 33-38 min: buffer/CH_3CN (5%); flow: 1 mL/min. All NMR spectra were carried out using Bruker spectrometers: Bruker AMX 400 or Bruker AVIII 600. All chemical shifts (δ) were given in ppm and calibrated on solvent signals. HRMS (ESI) mass spectra were performed with a VG Analytical Finnigan ThermoQuest MAT 95 XL or an Agilent 6224 EIS-TOF spectrometer. MALDI mass spectra were recorded on an ultrafleXtreme MALDI-TOF-TOF mass spectrometer by Bruker Daltonik with 9-AA as matrix.

Part 1. General Procedure: Preparation of T-1106-compounds 22-33 (NH_4^+ form)

All reactions were performed under nitrogen (N_2) atmosphere and dry conditions. Firstly, *H*-phosphonates **16**-**18**,**20**,**36** and *H*-phosphinates **19**,**21**,**44** (0.3 mmol, 1.0 equiv.) were dissolved in CH_3CN or THF. NCS (0.3 mmol, 1.0 equiv.) was added and the mixture was stirred for 2 h at room temperature. Subsequently, the corresponding T-1106-MP **6** ($n$$Bu_4N^+$ form) or T-1106-DP **7** ($n$$Bu_4N^+$ form) (0.21-0.3 mmol, 0.7-1.0 equiv.) in 6 mL of CH_3CN was added to the mixture and then stirred for 4 h at room temperature. All volatile components were removed in vacuum. The crude product was purified by automatic RP18 flash chromatography, and followed by ion-exchange to the ammonium form with Dowex 50WX8 cation-exchange resin and a second RP18 chromatography purification step. Product-containing fractions were freeze-dried and the target T-1106-Di*PP*ro- and Tri*PPP*ro-compounds **22-33** (NH_4^+ form) were obtained as solids.

Part 2. Synthesis and Characterization.

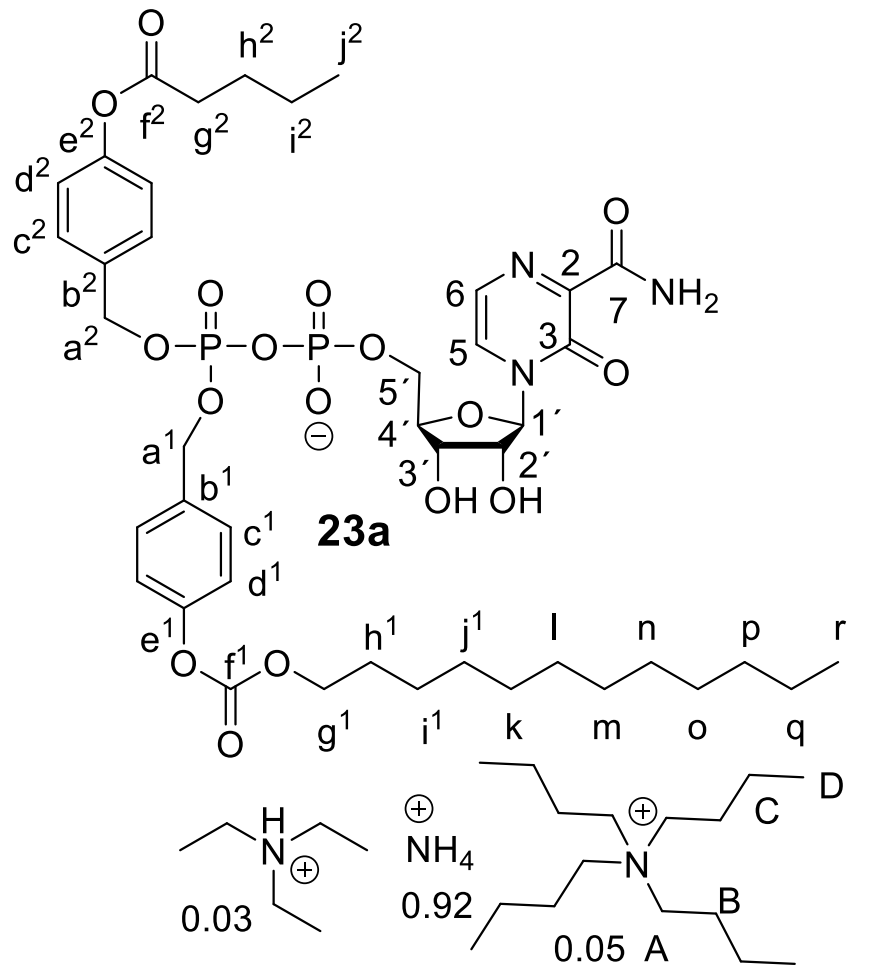

β-(AB-C4;ACB-C12)-T-1106-DP **23a**. According to general procedure with 177 mg (AB-C4;ACB-C12)-*H*-phosphonate **17a** (0.3 mmol, 1.0 equiv.), 40 mg NCS (0.3 mmol, 1.0 equiv.), and 250 mg T-1106-MP 2×*n*Bu_4N^+ salt (0.3 mmol, 1.0 equiv.) and then stirred for 4 h at room temperature. Yield: 149 mg (0.16 mmol, 52%) white solid. Purity (HPLC): > 96%. ^{1}H NMR (600 MHz, CD_3OD): δ [ppm] = 8.30 (d, $^3J_{HH}$= 4.2 Hz, 1H, H-5), 7.72 (dd, $^3J_{HH}$= 4.2 Hz, $^4J_{HH}$= 1.5 Hz, 1H, H-6), 7.44-7.36 (m, 4H, H-c^1, H-c^2), 7.16-7.11 (m, 2H, H-d^1), 7.05-7.02 (m, 2H, H-d^2), 6.05 (d, $^3J_{HH}$= 2.0 Hz, 1H, H-1´), 5.16-5.10 (m, 4H, H-a^1, H-a^2), 4.45-4.10 (m, 7H, H-2´, H-3´, H-4´, H-5´, H-g^1), 3.28-3.20 (m, 0.58H, H-A, $HN(CH_2CH_3)_3^+$), 2.57 (t, $^3J_{HH}$= 7.5 Hz, 2H, H-g^2), 1.75-1.62 (m, 4.4H, H-B, H-h^1, H-h^2), 1.48-1.37 (m, 4.4H, H-C, H-i^1, H-i^2), 1.35-1.25 (m, 16H, H-j^1, H-k, H-l, H-m, H-n, H-o, H-p, H-q), 1.02 (t, $^3J_{HH}$= 7.4 Hz, 0.6H, H-D), 1.00 (t, $^3J_{HH}$= 7.3 Hz, 3H, H-j^2), 0.90 (t, $^3J_{HH}$= 6.8 Hz, 3H, H-r). ^{13}C NMR (151 MHz, CD_3OD): δ [ppm] = 173.7 (C-f^2), 166.3 (C-7), 157.0 (C-3), 155.1 (C-f^1), 152.7 (C-e^1), 152.4 (C-e^2), 144.0 (C-2), 135.1 (C-b^1), 134.8 (C-b^2), 130.51, 130.48, 130.44, 130.3 (C-5, C-c^1, C-c^2), 125.9 (C-6), 122.9 (C-d^2), 122.3 (C-d^1), 92.4 (C-1´), 84.3 (d, $^3J_{CP}$= 9.6 Hz, C-4´), 76.7 (C-2´), 70.4, 70.3 (2 × dd, $^3J_{CP}$= 2.6 Hz, $^3J_{CP}$= 5.6 Hz, $^3J_{CP}$= 2.3 Hz, $^3J_{CP}$= 6.5 Hz, C-a^1, C-a^2), 70.0 (C-g^1), 69.7 (C-3´), 65.6 (C-5´), 59.5 (t, $^3J_{CP}$= 2.8 Hz, C-A), 34.7 (C-g^2), 33.1, 30.79, 30.77, 30.75, 30.74, 30.68, 30.63, 30.5, 30.3, 23.7 (C-j^1, C-k, C-l, C-m, C-n, C-o, C-p, C-q), 29.7 (C-h^1), 28.1 (C-h^2), 26.8 (C-i^1), 24.8 (C-B), 23.3 (C-i^2), 20.7(C-C), 14.5 (C-r), 14.1 (C-j^2), 13.9 (C-D). ^{31}P NMR (243 MHz, CD_3OD): δ [ppm] = -11.90 (d, $^2J_{pp}$= 20.3 Hz, P-*β*), -12.73 (d, $^2J_{pp}$= 18.3 Hz, P-*α*). HRMS (ESI$^-$, m/z): $C_{42}H_{60}N_3O_{17}P_2$, [M-H]$^-$ 938.3247; found 938.3354.

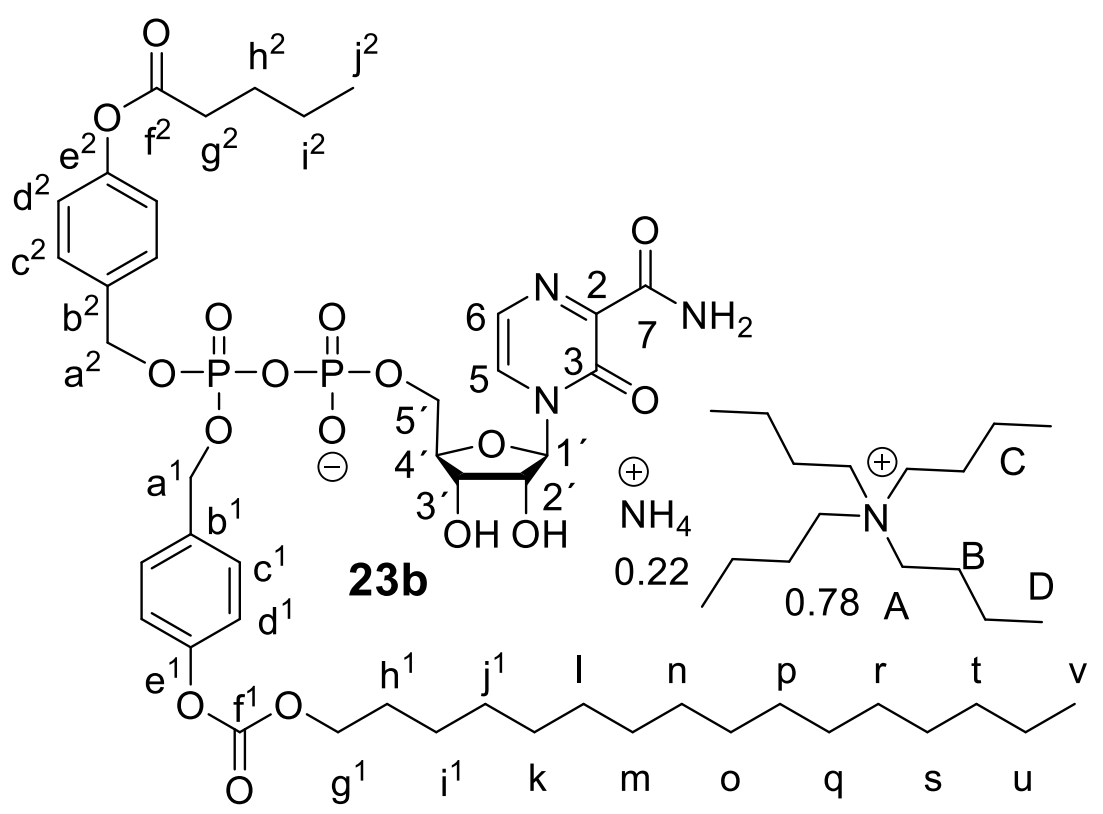

β-(AB-C4;ACB-C16)-T-1106-DP **23b**. According to general procedure with 194 mg (AB-C4;ACB-C16)-*H*-phosphonate **17b** (0.3 mmol, 1.0 equiv.), 40 mg NCS (0.3 mmol, 1.0 equiv.), and 250 mg T-1106-MP 2×*n*Bu_4N^+ salt (0.3 mmol, 1.0 equiv.) and then stirred for 4 h at room temperature. Yield: 210 mg (0.18 mmol, 59%) white solid. Purity (HPLC): > 98%. ^{1}H NMR (600 MHz, CD_3OD): δ [ppm] = 8.30 (d, $^3J_{HH}$= 4.2 Hz, 1H, H-5), 7.79 (dd, $^3J_{HH}$= 4.2 Hz, $^4J_{HH}$= 1.5 Hz, 1H, H-6), 7.44-7.36 (m, 4H, H-c^1, H-c^2), 7.16-7.10 (m, 2H, H-d^1), 7.05-7.02 (m, 2H, H-d^2), 6.05 (d, $^3J_{HH}$= 2.0 Hz, 1H, H-1´), 5.20-5.10 (m, 4H, H-a^1, H-a^2), 4.45-4.10 (m, 7H, H-2´, H-3´, H-4´, H-5´, H-g^1), 3.28-3.20 (m, 6.24H, H-A), 2.57 (t, $^3J_{HH}$= 7.5 Hz, 2H, H-h^2), 1.75-1.62 (m, 10.24H, H-B, H-h^1, H-h^2), 1.48-1.39 (m, 10.24H, H-C, H-i^1, H-i^2), 1.35-1.25 (m, 24H, H-j^1, H-k, H-l, H-m, H-n, H-o, H-p, H-q, H-r, H-s, H-t, H-u), 1.02 (t, $^3J_{HH}$= 7.3 Hz, 9.36H, H-D), 0.98 (t, $^3J_{HH}$= 7.3 Hz, 3H, H-j^2), 0.90 (t, $^3J_{HH}$= 6.4 Hz, 3H, H-v). ^{13}C NMR (151 MHz, CD_3OD): δ [ppm] = 173.7 (C-f^2), 166.3 (C-7), 157.0 (C-3), 155.1 (C-f^1), 152.7 (C-e^1), 152.4 (C-e^2), 144.0 (C-2), 135.1 (C-b^1), 134.8 (C-b^2), 130.51, 130.48, 130.44, 130.3 (C-5, C-c^1, C-c^2), 125.8

(C-6), 122.9 (C-d^2), 122.3 (C-d^1), 92.4 (C-1´), 84.3 (d, $^3J_{CP}$= 9.6 Hz, C-4´), 76.7 (C-2´), 70.4, 70.3 (2 × dd , $^3J_{CP}$= 2.4 Hz, $^3J_{CP}$= 5.8 Hz, $^3J_{CP}$= 2.3 Hz, $^3J_{CP}$= 5.6 Hz, C-a^1, C-a^2), 70.0 (C-g^1), 69.7 (C-3´), 65.6 (d, $^3J_{CP}$= 5.6 Hz, C-5´), 59.5 (t, $^3J_{CP}$= 2.8 Hz, C-A), 34.7 (C-g^2), 33.1, 30.76, 30.69, 30.64, 30.5, 30.3, 23.7 (C-j^1, C-k, C-l, C-m, C-n, C-o, C-p, C-q, C-r, C-s, C-t, C-u), 29.7 (C-h^1), 28.1 (C-h^2), 26.8 (C-i^1), 24.8 (C-B), 23.3 (C-i^2), 20.7(C-C), 14.4 (C-r), 14.1 (C-j^2), 13.9 (C-D). ^{31}P NMR (243 MHz, CD_3OD): δ [ppm] = -11.88 (d, $^2J_{pp}$= 20.5 Hz, P-β), -12.73 (d, $^2J_{pp}$= 18.3 Hz, P-α). HRMS (ESI$^-$, m/z): $C_{46}H_{68}N_3O_{17}P_2$, [M-H]$^-$ 994.3873; found 994.3878.

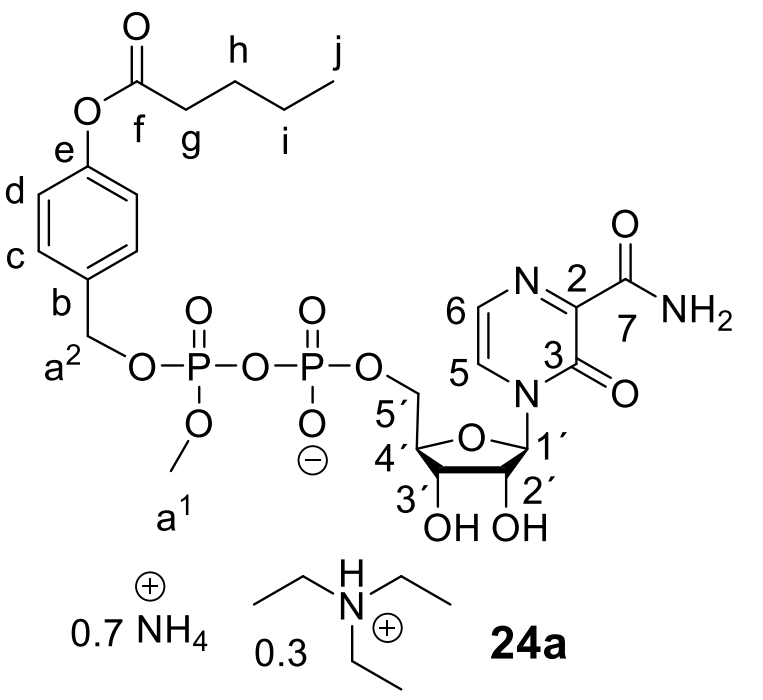

24a

β-(AB-C4;alkyl-C1)-T-1106-DP **24a**. According to general procedure with 86 mg (AB-C4;alkyl-C1)-*H*-phosphonate **18a** (0.3 mmol, 1.0 equiv.), 40 mg NCS (0.3 mmol, 1.0 equiv.), and 250 mg T-1106-MP 2×nBu$_4$N$^+$ salt (0.3 mmol, 1.0 equiv.) and then stirred for 4 h at room temperature. Yield: 69 mg (0.1 mmol, 34%) white solid. Purity (HPLC): > 96%. ^{1}H NMR (400 MHz, CD_3OD): δ [ppm] = 8.32 (dd, $^3J_{HH}$= 4.1 Hz, $^3J_{HH}$= 2.0 Hz, 1H, H-5), 7.74 (t, $^3J_{HH}$= 4.1 Hz, 1H, H-6), 7.48-7.43 (m, 2H, H-c), 7.10-7.04 (m, 2H, H-d), 6.07 (d, $^4J_{HH}$= 1.7 Hz, 1H, H-1´), 5.18 (d, $^3J_{HH}$= 8.1 Hz, 2H, H-a^2), 4.44-4.14 (m, 5H, H-2´, H-3´, H-4´,H-5´), 3.81 (dd, $^2J_{HH}$= 11.6 Hz, $^4J_{HH}$= 1.4 Hz, 3H, H-a^1), 3.04 (m, 1.8H, $HN(CH_2CH_3)_3^+$), 2.57 (t, $^3J_{HH}$= 7.4 Hz, 2H, H-g), 1.75-1.65 (m, 2H, H-h), 1.50-1.40 (m, 2H, H-i), 1.34-1.26 (m, 2.7H, $HN(CH_2CH_3)_3^+$), 0.98 (t, $^3J_{HH}$= 7.4 Hz, 2H, H-j). ^{13}C-NMR (101 MHz, CD_3OD): δ [ppm] = 173.7 (C-f), 166.3 (C-7), 157.0 (C-3), 152.3 (C-e), 144.0 (C-2), 134.9 (d, $^3J_{CP}$= 7.1 Hz, C-b), 130.40 (C-d), 130.36 (C-5), 125.8 (C-6), 122.9 (C-c), 92.4 (C-1´), 84.3 (d, $^3J_{CP}$= 9.0 Hz, C-4´), 76.7 (C-2´), 70.3 (C-a^2), 69.7 (C-3´), 65.6 (d, $^3J_{CP}$= 2.5 Hz, C-5´), 55.5 (d, $^3J_{CP}$= 6.0 Hz, C-a^1), 43.5 ($HN(CH_2CH_3)_3^+$), 34.7 (C-g), 28.0 (C-h), 23.2 (C-i), 14.1 (C-j), 11.6, 9.2 ($HN(CH_2CH_3)_3^+$). ^{31}P NMR (162 MHz, CD_3OD): δ [ppm] = -11.41 (dd, $^2J_{pp}$= 19.6 Hz, $^3J_{pp}$= 7.8 Hz, P-β), -11.95 (d, $^2J_{pp}$= 19.5 Hz, P-α). HRMS (ESI$^-$, m/z): calculated for $C_{23}H_{31}N_3O_{14}P_2$, [M-H]$^-$ 634.1208; found 634.1209.

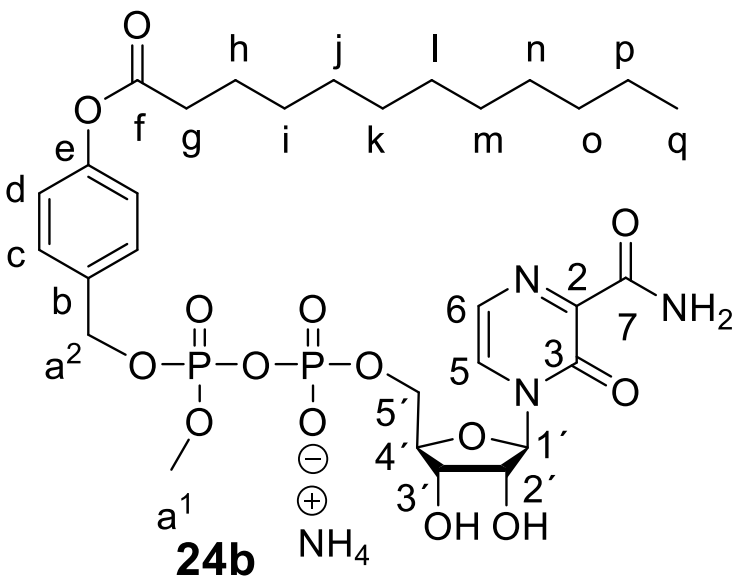

24b

β-(AB-C11;alkyl-C1)-T-1106-DP **24b**. According to general procedure with 115 mg (AB-C11;alkyl-C1)-*H*-phosphonate **18b** (0.3 mmol, 1.0 equiv.), 40 mg NCS (0.3 mmol, 1.0 equiv.), and 250 mg T-1106-MP 2×nBu$_4$N$^+$ salt (0.3 mmol, 1.0 equiv.) and then stirred for 4 h at room temperature. Yield: 70 mg (0.09 mmol, 31%) white solid. Confirmed purity: > 98%. ^{1}H NMR (600 MHz, CD_3OD): δ [ppm] = 8.35-8.30 (m, 1H, H-5), 7.80-7.70 (m, 1H, H-6), 7.48-7.44 (m, 2H, H-c), 7.10-7.03 (m, 2H, H-d), 6.07 (d, $^4J_{HH}$= 1.9 Hz, 1H, H-1´), 5.18 (d, $^3J_{HH}$= 8.1 Hz, 2H, H-a^2), 4.44-4.14 (m, 5H, H-2´, H-3´, H-4´,H-5´), 3.81 (dd, $^2J_{HH}$= 11.6 Hz, $^4J_{HH}$= 1.9 Hz, 3H, H-a^1), 2.56 (t, $^3J_{HH}$= 7.3 Hz, 2H, H-g), 1.75-1.68 (m, 2H, H-h), 1.47-1.40 (m, 2H, H-i), 1.38-1.25 (m, 14H, H-j, H-k, H-l, H-m, H-n, H-o, H-p), 0.90 (t, $^3J_{HH}$= 6.9 Hz, 2H, H-j). ^{13}C-NMR (151 MHz, CD_3OD): δ [ppm] = 173.7 (C-f), 166.3 (C-7), 157.1 (C-3), 152.4 (C-e), 144.0 (C-2), 134.9 (d, $^3J_{CP}$= 6.9 Hz, C-b), 130.40 (C-d), 130.36 (C-5), 125.8 (C-6), 122.9 (C-c), 92.4 (C-1´), 84.3 (d, $^3J_{CP}$= 9.1 Hz, C-4´), 76.7 (C-2´), 70.3 (C-a^2), 69.7 (C-3´), 65.6 (t, $^3J_{CP}$= 5.7 Hz, C-5´), 55.5 (d, $^3J_{CP}$= 6.2 Hz, C-a^1), 35.0 (C-g), 33.1, 30.7, 30.6, 30.5, 30.4, 30.2, 23.7 (C-i, C-j, C-k, C-l, C-m, C-n, C-o, C-p), 25.9 (C-h), 14.4 (C-q). ^{31}P NMR (243 MHz, CD_3OD): δ [ppm] = -11.41 (dd, $^2J_{pp}$= 20.6 Hz, $^3J_{pp}$=

8.8 Hz, P-β), -11.95 (d, $^{2}J_{pp}$= 17.7 Hz, P-α). HRMS (ESI$^-$, m/z): calculated for $C_{30}H_{45}N_3O_{14}P_2$, [M-H]$^-$ 732.2304; found 732.2299.

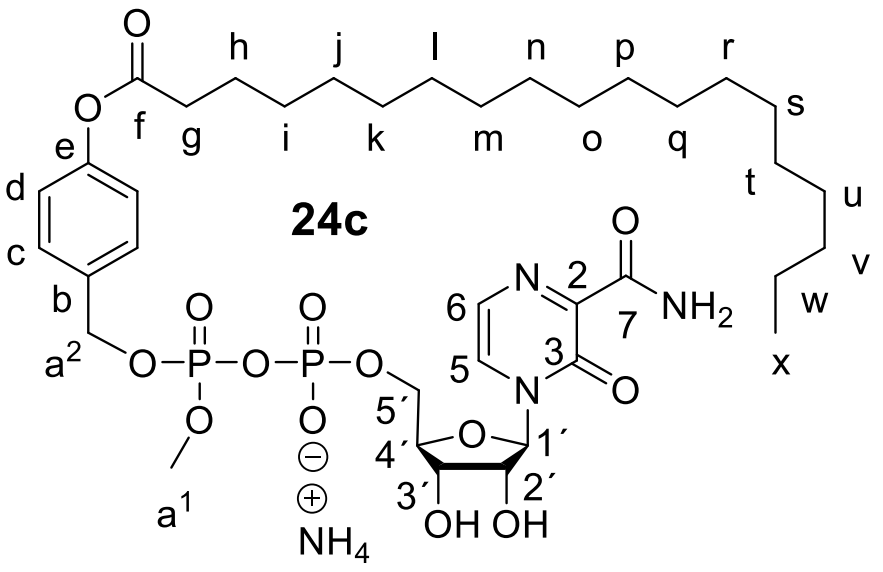

β-(AB-C17;alkyl-C1)-T-1106-DP **24c**. According to general procedure with 145 mg (AB-C17;alkyl-C1)-*H*-phosphonate **18c** (0.3 mmol, 1.0 equiv.), 40 mg NCS (0.3 mmol, 1.0 equiv.), and 250 mg T-1106-MP 2×*n*Bu_4N^+ salt (0.3 mmol, 1.0 equiv.) and then stirred for 4 h at room temperature. Yield: 90 mg (0.11 mmol, 36%) white solid. Purity (HPLC): > 95%. ^{1}H NMR (400 MHz, CD_3OD): δ [ppm] = 8.32 (dd, $^{3}J_{HH}$= 4.1 Hz, $^{3}J_{HH}$= 2.2 Hz, 1H, H-5), 7.74 (t, $^{3}J_{HH}$= 4.4 Hz, 1H, H-6), 7.50-7.44 (m, 2H, H-c), 7.10-7.03 (m, 2H, H-d), 6.07 (d, $^{4}J_{HH}$= 1.9 Hz, 1H, H-1´), 5.18 (d, $^{3}J_{HH}$= 8.0 Hz, 2H, H-a^2), 4.44-4.14 (m, 5H, H-2´, H-3´, H-4´,H-5´), 3.81 (dd, $^{2}J_{HH}$= 11.6 Hz, $^{4}J_{HH}$= 1.4 Hz, 3H, H-a^1), 2.56 (t, $^{3}J_{HH}$= 7.4 Hz, 2H, H-g), 1.74-1.68 (m, 2H, H-h), 1.38-1.25 (m, 28H, H-i, H-j, H-k, H-l, H-m, H-n, H-o, H-p, H-q, H-r, H-s, H-t, H-u, H-v), 0.90 (t, $^{3}J_{HH}$= 6.9 Hz, 2H, H-w). ^{13}C-NMR (101 MHz, CD_3OD): δ [ppm] = 173.7 (C-f), 157.5 (C-3), 152.4 (C-e), 144.0 (C-2), 134.9 (C-b), 130.40 (C-d), 130.36 (C-5), 125.8 (C-6), 122.9 (C-c), 92.4 (C-1´), 84.3 (C-4´), 76.7 (C-2´), 70.3 (C-a^2), 69.7 (C-3´), 65.6 (C-5´), 55.5 (C-a^1), 35.0 (C-g), 33.1, 30.76, 30.71, 30.6, 30.5, 30.4, 30.2, 23.7 (C-i, C-j, C-k, C-l, C-m, C-n, C-o, C-p, C-q, C-r, C-s, C-t, C-u, C-v), 25.9 (C-h), 14.4 (C-w). ^{31}P NMR (162 MHz, CD_3OD): δ [ppm] = -11.6 -- -11.3 (m, P-β), -11.95 (d, $^{2}J_{pp}$= 19.6 Hz, P-α). HRMS (ESI$^-$, m/z): calculated for $C_{36}H_{57}N_3O_{14}P_2$, [M-H]$^-$ 816.3243; found 816.3247.

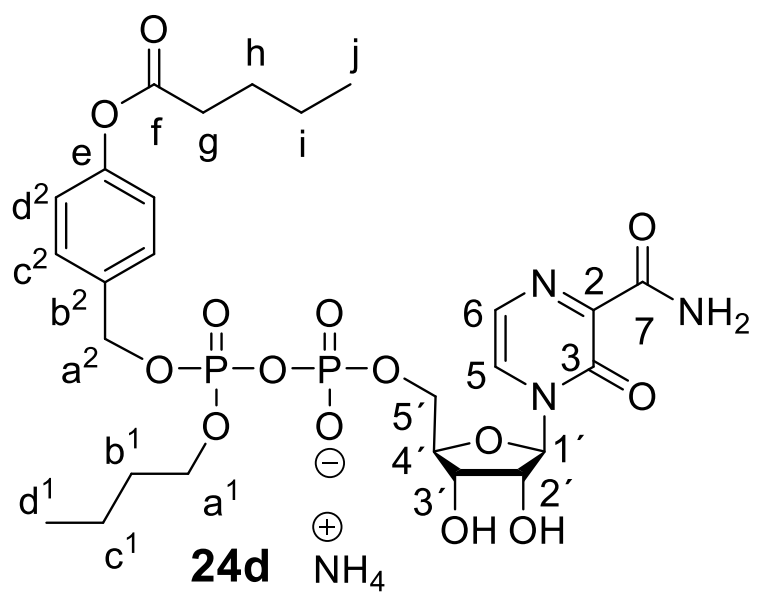

β-(AB-C4;alkyl-C4)-T-1106-DP **24d**. According to general procedure with 99 mg (AB-C4;alkyl-C4)-*H*-phosphonate **18d** (0.3 mmol, 1.0 equiv.), 40 mg NCS (0.3 mmol, 1.0 equiv.), and 250 mg T-1106-MP 2×*n*Bu_4N^+ salt (0.3 mmol, 1.0 equiv.) and then stirred for 4 h at room temperature. Yield: 65 mg (0.09 mmol, 31%) white solid. Purity (HPLC): > 96%. ^{1}H NMR (600 MHz, CD_3OD): δ [ppm] = 8.33 (d, $^{3}J_{HH}$= 4.2 Hz, 1H, H-5), 7.75 (t, $^{3}J_{HH}$= 3.4 Hz, 1H, H-6), 7.50-7.44 (m, 2H, H-c^2), 7.10-7.03 (m, 2H, H-d^2), 6.07 (d, $^{4}J_{HH}$= 1.9 Hz, 1H, H-1´), 5.20-5.16 (m, 2H, H-a^2), 4.44-4.14 (m, 5H, H-2´, H-3´, H-4´,H-5´), 4.13-4.07 (m, 2H, H-a^1), 2.57 (t, $^{3}J_{HH}$= 7.4 Hz, 2H, H-g), 1.74-1.68 (m, 2H, H-h), 1.67-1.60 (m, 2H, H-b^1), 1.48-1.41 (m, 2H, H-i), 1.41-1.34 (m, 2H, H-c^1), 0.98 (t, $^{3}J_{HH}$= 7.4 Hz, 2H, H-j), 0.90 (dt, $^{3}J_{HH}$= 7.4 Hz, $^{3}J_{HH}$= 2.7 Hz, 2H, H-d^1). ^{13}C-NMR (151 MHz, CD_3OD): δ [ppm] = 173.7 (C-f), 166.3 (C-7), 157.0 (C-3), 152.4 (C-e), 144.0 (C-2), 135.0 (d, $^{3}J_{CP}$= 7.0 Hz, C-b^2), 130.3 (d, $^{3}J_{CP}$= 4.8 Hz, C-d^2), 129.6 (C-5), 125.9 (C-6), 122.9 (C-c^2), 92.4 (C-1´), 84.3 (d, $^{3}J_{CP}$= 9.3 Hz, C-4´), 76.7 (C-2´), 70.2 (dd, $^{3}J_{CP}$= 5.6 Hz, $^{3}J_{CP}$= 2.3 Hz, C-a^2), 69.7 (d, $^{3}J_{CP}$= 3.9 Hz, C-a^1), 69.5 (d, $^{3}J_{CP}$= 6.2 Hz, C-3´), 65.5 (dd, $^{3}J_{CP}$= 5.1 Hz, $^{3}J_{CP}$= 2.3 Hz, C-5´), 34.7 (C-g^2), 33.3 (d, $^{3}J_{CP}$ = 7.3 Hz, C-b^1), 28.0 (C-h), 19.7 (C-c^1), 23.2 (C-i), 14.1 (C-j), 13.9 (C-d^1). ^{31}P NMR (243 MHz, CD_3OD): δ [ppm] = -11.98 (d, $^{2}J_{pp}$= 18.2 Hz, P-β), -12.57 (dd, $^{2}J_{pp}$= 20.5 Hz, $^{3}J_{pp}$= 5.9 Hz, P-α). HRMS (ESI$^-$, m/z): calculated for $C_{26}H_{37}N_3O_{14}P_2$, [M-H]$^-$ 676.1678; found 676.1671.

β-(AB-C11;alkyl-C4)-T-1106-DP **24e**. According to general procedure with 128 mg (AB-C11;alkyl-C4)-*H*-phosphonate **18e** (0.3 mmol, 1.0 equiv.), 40 mg NCS (0.3 mmol, 1.0 equiv.), and 250 mg T-1106-

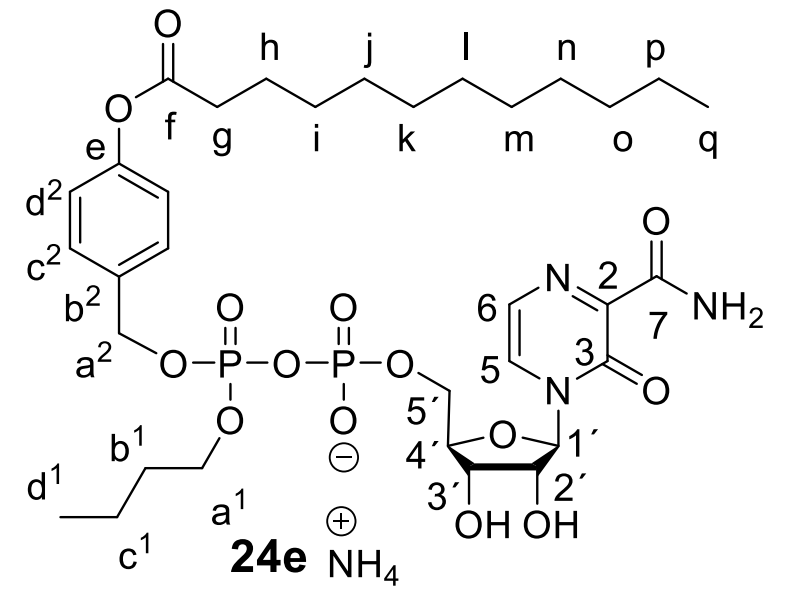

24e

MP 2×nBu$_4$N$^+$ salt (0.3 mmol, 1.0 equiv.) and then stirred for 4 h at room temperature. Yield: 57 mg (0.07 mmol, 24%) white solid. Purity (HPLC): > 97%. ^{1}H NMR (400 MHz, CD$_3$OD): δ [ppm] = 8.33 (d, $^3J_{HH}$= 4.0 Hz, 1H, H-5), 7.75 (d, $^3J_{HH}$= 3.4 Hz, 1H, H-6), 7.52-7.44 (m, 2H, H-c^2), 7.14-7.02 (m, 2H, H-d^2), 6.07 (d, $^4J_{HH}$= 1.6 Hz, 1H, H-1′), 5.18 (d, $^3J_{HH}$= 8.0 Hz, 2H, H-a^2), 4.44-4.07 (m, 7H, H-2′, H-3′, H-4′, H-5′, H-a^1), 2.61-2.52 (m, 2H, H-g), 1.75-1.67 (m, 2H, H-h), 1.66-1.56 (m, 2H, H-b^1), 1.46-1.26 (m, 18H, H-c^1, H-i, H-j, H-k, H-l, H-m, H-n, H-o, H-p), 0.94-0.86 (m, 6H, H-d^1, H-q). ^{13}C-NMR (101 MHz, CD$_3$OD): δ [ppm] = 173.7 (C-f), 166.3 (C-7), 157.0 (C-3), 152.4 (C-e), 144.1 (C-2), 135.0 (C-b^2), 130.38, 130.34 (C-d^2, C-5), 125.9 (C-6), 122.9 (C-c^2), 92.4 (C-1′), 84.4 (C-4′), 76.7 (C-2′), 70.2 (C-a^2), 69.7 (C-a^1), 69.5 (C-3′), 65.5 (C-5′), 35.0 (C-g^2), 33.3 (d, $^3J_{CP}$ = 7.1 Hz, C-b^1), 33.1, 30.7, 30.6, 30.5, 30.4, 30.1, 23.7 (C-i, C-j, C-k, C-l, C-m, C-n, C-o, C-p), 25.9 (C-h), 19.7 (C-c^1), 14.4 (C-q), 13.9 (C-d^1). ^{31}P NMR (162 MHz, CD$_3$OD): δ [ppm] = -11.98 -- -11.78 (m, 1P, P-β), -12.62 (d, $^2J_{pp}$= 20.2 Hz, P-α). HRMS (ESI$^-$, m/z): $C_{33}H_{51}N_3O_{14}P_2$, [M-H]$^-$ 774.2773; found 774.2813.

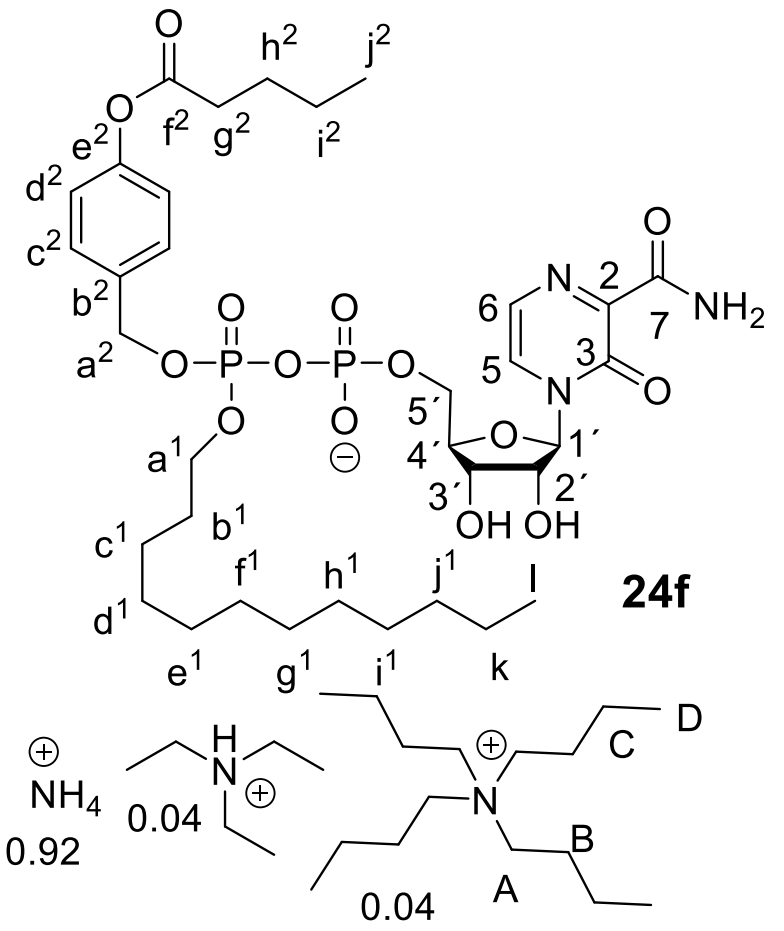

24f

β-(AB-C4;alkyl-C12)-T-1106-DP **24f**. According to general procedure with 132 mg (AB-C4;alkyl-C12)-*H*-phosphonate **18f** (0.3 mmol, 1.0 equiv.), 40 mg NCS (0.3 mmol, 1.0 equiv.), and 250 mg T-1106-MP 2×nBu$_4$N$^+$ salt (0.3 mmol, 1.0 equiv.) and then stirred for 4 h at room temperature. Yield: 101 mg (0.12 mmol, 41%) white solid. Purity (HPLC): > 98%. ^{1}H NMR (600 MHz, CD$_3$OD): δ [ppm] = 8.37-8.33 (m, 1H, H-5), 7.77-7.72 (m, 1H, H-6), 7.48-7.44 (m, 2H, H-c^2), 7.10-7.03 (m, 2H, H-d^2), 6.07 (d, $^4J_{HH}$= 1.9 Hz, 1H, H-1′), 5.18 (dd, $^3J_{HH}$= 8.1 Hz, $^3J_{HH}$= 2.4 Hz, 2H, H-a^2), 4.44-4.14 (m, 5H, H-2′, H-3′, H-4′,H-5′), 4.13-4.07 (m, 2H, H-a^1), 3.26-3.22 (m, 0.32H, H-A), 3.21-3.18 (m, 0.24H, HN(CH$_2$CH$_3$)$_3^+$), 2.57 (t, $^3J_{HH}$= 7.4 Hz, 2H, H-g^2), 1.75-1.68 (m, 2H, H-h^2), 1.67-1.60 (m, 2H, H-b^1), 1.48-1.40 (m, 2.32H, H-B, H-i^2), 1.35-1.25 (m, 18.36H, H-C, H-c^1, H-d^1, H-e^1, H-f^1, H-g^1, H-h^1, H-i^1, H-j^1, H-k, HN(CH$_2$CH$_3$)$_3^+$), 1.02 (t, $^3J_{HH}$= 7.3 Hz, 2H, H-D), 0.98 (t, $^3J_{HH}$= 7.4 Hz, 2H, H-j^2), 0.90 (t, $^3J_{HH}$= 6.9 Hz, 2H, H-l). ^{13}C-NMR (151 MHz, CD$_3$OD): δ [ppm] = 173.6 (C-f^2), 166.3 (C-7), 157.1 (C-3), 152.4 (C-e^2), 144.0 (C-2), 135.0 (d, $^3J_{CP}$= 7.0 Hz, C-b^2), 130.38 (C-d^2), 130.35 (C-5), 125.9 (C-6), 122.9 (C-c^2), 92.4 (C-1′), 84.4 (C-4′), 76.7 (C-2′), 70.3 (dd, $^3J_{CP}$= 5.7 Hz, $^4J_{CP}$= 1.7 Hz, C-a^2), 69.8 (d, $^3J_{CP}$= 6.2 Hz, C-a^1), 69.6 (d, $^3J_{CP}$= 5.2 Hz, C-3′), 65.5 (t, $^3J_{CP}$= 5.6 Hz, C-5′), 34.7 (C-g^2), 33.1, 30.80, 30.78, 30.73, 30.67, 30.5, 30.3, 23.7 (C-d^1, C-e^1, C-f^1, C-g^1, C-h^1, C-i^1, C-j^1, C-k), 31.2 (d, $^3J_{CP}$ = 6.9 Hz, C-b^1), 28.1 (C-h^2), 26.5 (C-c^1), 24.8 (C-B), 23.2 (C-i^2), 20.7 (C-C), 14.4 (C-l), 14.1 (C-j^2), 13.9 (C-D). ^{31}P NMR (243 MHz, CD$_3$OD): δ [ppm] = -11.97 (d, $^2J_{pp}$= 20.5 Hz, P-β), -12.66 -- -12.45 (m, P-α). HRMS (ESI$^-$, m/z): $C_{34}H_{53}N_3O_{14}P_2$, [M-H]$^-$ 788.2930; found 788.2888.

β-(AB-C11;alkyl-C12)-T-1106-DP **24g**. According to general procedure with 162 mg (AB-C11;alkyl-C12)-*H*-phosphonate **18g** (0.3 mmol, 1.0 equiv.), 40 mg NCS (0.3 mmol, 1.0 equiv.), and 250 mg T-1106-MP 2×nBu$_4$N$^+$ salt (0.3 mmol, 1.0 equiv.) and then stirred for 4 h at room temperature. Yield: 90 mg (0.1 mmol, 33%) white solid. Purity (HPLC): > 97%. ^{1}H NMR (600 MHz, CD$_3$OD): δ [ppm] = 8.37-

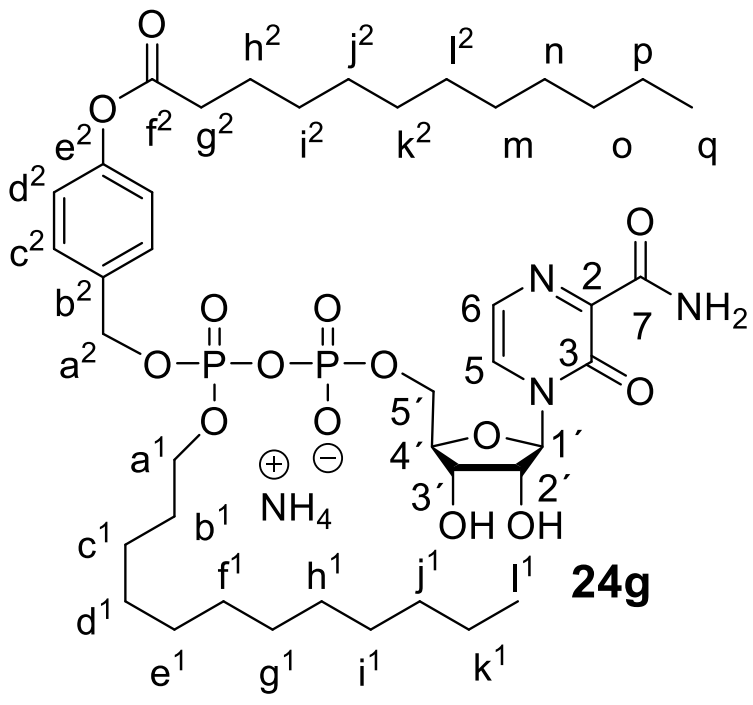

24g

8.33 (m, 1H, H-5), 7.79-7.74 (m, 1H, H-6), 7.50-7.45 (m, 2H, H-c^2), 7.10-7.03 (m, 2H, H-d^2), 6.07 (d, $^4J_{HH}$= 1.9 Hz, 1H, H-1´), 5.18 (dd, $^3J_{HH}$= 8.1 Hz, $^3J_{HH}$= 2.8 Hz, 2H, H-a^2), 4.44-4.14 (m, 5H, H-2´, H-3´, H-4´,H-5´), 4.13-4.06 (m, 2H, H-a^1), 2.56 (t, $^3J_{HH}$= 7.8 Hz, 2H, H-g^2), 1.75-1.68 (m, 2H, H-h^2), 1.65-1.58 (m, 2H, H-b^1), 1.48-1.25 (m, 36H, H-c^1, H-d^1, H-e^1, H-f^1, H-g^1, H-h^1, H-i^1, H-i^2, H-j^1, H-j^2, H-k^1, H-k^2, H-l^1, H-l^2, H-m, H-n, H-o, H-p), 0.92-0.88 (m, 6H, H-l^1, H-q). ^{13}C-NMR (151 MHz, CD_3OD): δ [ppm] = 173.6 (C-f^2), 166.3 (C-7), 157.0 (C-3), 152.4 (C-e^2), 144.0 (C-2), 135.0 (d, $^3J_{CP}$= 7.0 Hz, C-b^2), 130.40 (C-d^2), 130.37 (C-5), 125.9 (C-6), 122.9 (C-c^2), 92.4 (C-1´), 84.3 (d, $^3J_{CP}$= 9.6 Hz, C-4´), 76.7 (C-2´), 70.2 (dd, $^3J_{CP}$= 5.7 Hz, $^4J_{CP}$= 1.6 Hz, C-a^2), 69.8 (d, $^3J_{CP}$= 6.6 Hz, C-a^1), 69.6 (d, $^3J_{CP}$= 4.0 Hz, C-3´), 65.5 (t, $^3J_{CP}$= 5.6 Hz, C-5´), 35.0 (C-g^2), 33.10, 33.07, 30.81, 30.79, 30.73, 30.68, 30.61, 30.51, 30.47, 30.4, 30.3, 30.2, 23.75, 23.74 (C-d^1, C-e^1, C-f^1, C-g^1, C-h^1, C-i^1, C-i^2, C-j^1, C-j^2, C-k^1, C-k^2, C-l^2, C-m, C-n, C-o, C-p), 31.2 (d, $^3J_{CP}$ = 7.1 Hz, C-b^1), 26.5 (C-c^1), 25.9 (C-h^2), 14.46, 14.44 (C-l^1, C-q). ^{31}P NMR (243 MHz, CD_3OD): δ [ppm] = -11.97 (d, $^2J_{pp}$= 17.7 Hz, P-β), -12.55 (d, $^2J_{pp}$= 20.5 Hz, P-α). HRMS (ESI$^-$, m/z): $C_{41}H_{67}N_3O_{14}P_2$, $[M-H]^-$ 886.4026; found 886.4043.

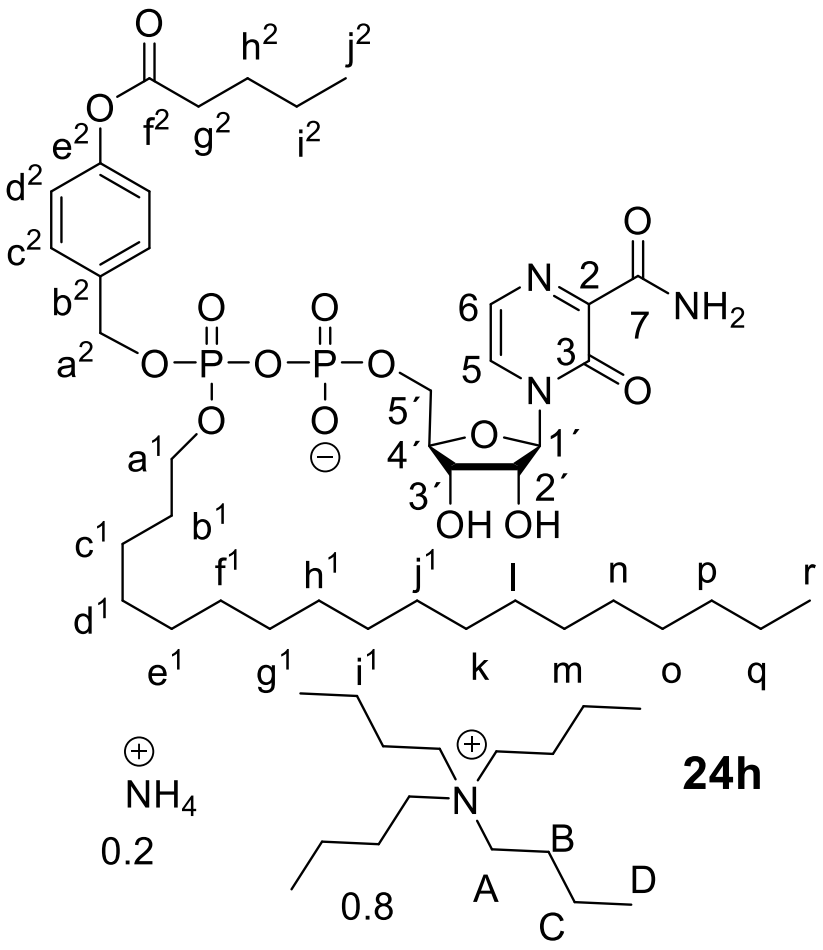

24h

β-(AB-C4;alkyl-C18)-T-1106-DP **24h**. According to general procedure with 157 mg (AB-C4;alkyl-C18)-*H*-phosphonate **18h** (0.3 mmol, 1.0 equiv.), 40 mg NCS (0.3 mmol, 1.0 equiv.), and 250 mg T-1106-MP 2×$n$$Bu_4N^+$ salt (0.3 mmol, 1.0 equiv.) and then stirred for 4 h at room temperature. Yield: 173 mg (0.16 mmol, 54%) white solid. Purity (HPLC): > 99%. ^{1}H NMR (400 MHz, CD_3OD): δ [ppm] = 8.37-8.33 (m, 1H, H-5), 7.76 (d, $^3J_{HH}$= 2.4 Hz, 1H, H-6), 7.50-7.44 (m, 2H, H-c^2), 7.10-7.03 (m, 2H, H-d^2), 6.07 (d, $^3J_{HH}$= 2.1 Hz, 1H, H-1´), 5.18 (dd, $^3J_{HH}$= 8.3 Hz, $^3J_{HH}$= 2.4 Hz, 2H, H-a^2), 4.45-4.14 (m, 5H, H-2´, H-3´, H-4´,H-5´), 4.13-4.07 (m, 2H, H-a^1), 3.26-3.22 (m, 6.4H, H-A), 2.57 (t, $^3J_{HH}$= 7.4 Hz, 2H, H-g^2), 1.75-1.60 (m, 10.4H, H-b^1, H-B, H-h^2), 1.48-1.38 (m, 8.4H, H-C, H-i^2), 1.35-1.25 (m, 30H, H-c^1, H-d^1, H-e^1, H-f^1, H-g^1, H-h^1, H-i^1, H-j^1, H-k, H-l, H-m, H-n, H-o, H-p, H-q), 1.02 (t, $^3J_{HH}$= 7.3 Hz, 9.6H, H-D), 0.98 (t, $^3J_{HH}$= 7.4 Hz, 2H, H-j^2), 0.90 (t, $^3J_{HH}$= 7.0 Hz, 2H, H-r). ^{13}C-NMR (101 MHz, CD_3OD): δ [ppm] = 173.6 (C-f^2), 166.2 (C-7), 157.0 (C-3), 152.4 (d, $^3J_{CP}$= 2.2 Hz, C-e^2), 144.0 (C-2), 135.0 (d, $^3J_{CP}$= 7.3 Hz, C-b^2), 130.38 (C-d^2), 130.35 (C-5), 125.9 (C-6), 122.9 (C-c^2), 92.3 (C-1´), 84.3 (d, $^3J_{CP}$= 9.5 Hz, C-4´), 76.7 (C-2´), 70.3 (dd, $^3J_{CP}$= 5.7 Hz, $^4J_{CP}$= 1.7 Hz, C-a^2), 69.8 (d, $^3J_{CP}$= 6.2 Hz, C-a^1), 69.6 (d, $^3J_{CP}$= 5.2 Hz, C-3´), 65.5 (t, $^3J_{CP}$= 5.6 Hz, C-5´), 59.5 (t, $^3J_{CP}$= 2.8 Hz, C-A), 34.7 (C-g^2), 33.1, 30.80, 30.76, 30.72, 30.67, 30.5, 30.3, 23.7 (C-d^1, C-e^1, C-f^1, C-g^1, C-h^1, C-i^1, C-j^1, C-k, C-l, C-m, C-n, C-o, C-p, C-q), 31.2 (d, $^3J_{CP}$ = 6.9 Hz, C-b^1), 28.1 (C-h^2), 26.5 (C-c^1), 24.8 (C-B), 23.2 (C-i^2), 20.7 (t, $^4J_{CP}$= 1.4 Hz, C-C), 14.4 (C-r), 14.1 (C-j^2), 13.9 (C-D). ^{31}P NMR (162 MHz, CD_3OD): δ [ppm] = -11.97 (d, $^2J_{pp}$= 20.4 Hz, P-β), -12.57 (dd, $^2J_{pp}$= 20.4 Hz, $^3J_{pp}$= 5.5 Hz, P-α). HRMS (ESI$^-$, m/z): $C_{40}H_{65}N_3O_{14}P_2$, $[M-H]^-$ 872.3869; found 872.3870.

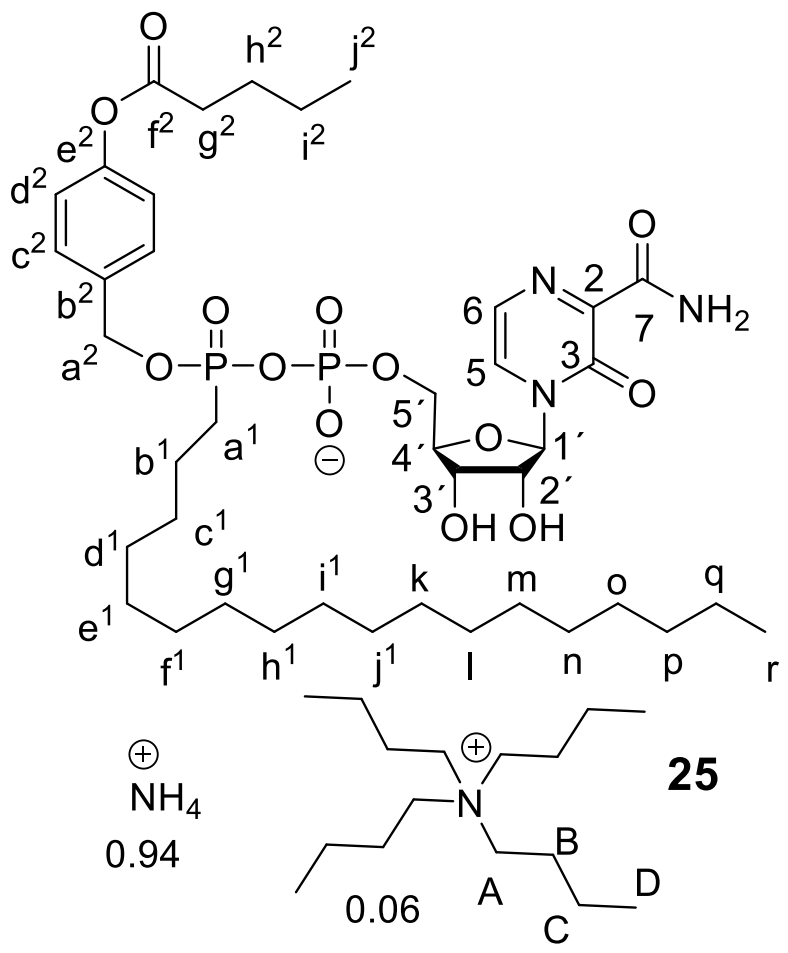

β-(AB-C4)-*β*-(alkyl-C18)-T-1106-DP **25**. According to general procedure with 153 mg (AB-C4;alkyl-C18)-*H*-phosphinate **19** (0.3 mmol, 1.0 equiv.), 40 mg NCS (0.3 mmol, 1.0 equiv.), and 250 mg T-1106-MP 2×*n*Bu_4N^+ salt (0.3 mmol, 1.0 equiv.) and then stirred for 4 h at room temperature. Yield: 131 mg (0.15 mmol, 49%) white solid. Purity (HPLC): > 99%. ^{1}H NMR (600 MHz, CD_3OD): δ [ppm] = 8.34 (t, $^3J_{HH}$= 4.4 Hz, 1H, H-5), 7.75 (dd, $^3J_{HH}$= 7.8 Hz, $^3J_{HH}$= 4.4 Hz, 1H, H-6), 7.50-7.44 (m, 2H, H-c^2), 7.10-7.03 (m, 2H, H-d^2), 6.07-6.05 (m, 1H, H-1´), 5.18 (d, $^3J_{HH}$= 7.8 Hz, 2H, H-a^2), 4.45-4.14 (m, 5H, H-2´, H-3´, H-4´,H-5´), 3.26-3.22 (m, 0.48H, H-A), 2.57 (t, $^3J_{HH}$= 7.4 Hz, 2H, H-g^2), 2.05-1.94 (m, 2H, H-a^1), 1.75-1.67 (m, 2H, H-h^2), 1.67-1.55 (m, 2.48H, H-b^1, H-B), 1.48-1.40 (m, 2.48H, H-C, H-i^2), 1.40-1.33 (m, 82H, H-c^1), 1.32-1.25 (m, 28H, H-d^1, H-e^1, H-f^1, H-g^1, H-h^1, H-i^1, H-j^1, H-k, H-l, H-m, H-n, H-o, H-p, H-q), 1.02 (t, $^3J_{HH}$= 7.3 Hz, 0.64H, H-D), 0.98 (t, $^3J_{HH}$= 7.4 Hz, 2H, H-j^2), 0.90 (t, $^3J_{HH}$= 7.0 Hz, 2H, H-r). ^{13}C-NMR (151 MHz, CD_3OD): δ [ppm] = 173.6 (d, $^3J_{CP}$= 2.2 Hz, C-f^2), 166.2 (C-7), 157.0 (C-3), 152.2 (C-e^2), 144.0 (C-2), 135.0 (dd, $^3J_{CP}$= 6.8 Hz, $^3J_{CP}$= 3.9 Hz, C-b^2), 130.33 (C-d^2), 130.27 (d, $^3J_{CP}$= 2.8 Hz, C-5), 125.8 (C-6), 122.9 (C-c^2), 92.4 (C-1´), 84.3 (d, $^3J_{CP}$= 8.5 Hz, C-4´), 76.7 (d, $^3J_{CP}$= 2.8 Hz, C-2´), 69.6 (d, $^3J_{CP}$= 5.2 Hz, C-3´), 69.8 (d, $^3J_{CP}$= 6.2 Hz, C-a^2), 65.5 (dd, $^3J_{CP}$= 9.8 Hz, $^3J_{CP}$= 5.6 Hz, C-5´), 59.5 (t, $^3J_{CP}$= 2.8 Hz, C-A), 34.7 (C-g^2), 33.1, 31.5, 31.4, 30.79, 30.75, 30.72, 30.51, 30.47, 30.2, 23.7 (C-c^1, C-d^1, C-e^1, C-f^1, C-g^1, C-h^1, C-i^1, C-j^1, C-k, C-l, C-m, C-n, C-o, C-p, C-q), 28.0 (C-h^2), 27.6, 26.7 (C-a^1), 24.8 (C-B), 23.3 (d, $^3J_{CP}$= 5.2 Hz, C-b^1), 23.2 (C-i^2), 20.7 (C-C), 14.4 (C-r), 14.1 (C-j^2), 13.9 (C-D). ^{31}P NMR (243 MHz, CD_3OD): δ [ppm] = -11.97 (dd, $^2J_{pp}$= 26.4 Hz, $^3J_{pp}$= 5.8 Hz, P-*β*), -12.57 (dd, $^2J_{pp}$= 23.8 Hz, $^3J_{pp}$= 6.1 Hz, P-*α*). HRMS (ESI$^-$, m/z): $C_{40}H_{65}N_3O_{13}P_2$, $[M-H]^-$ 856.3920; found 856.3890.

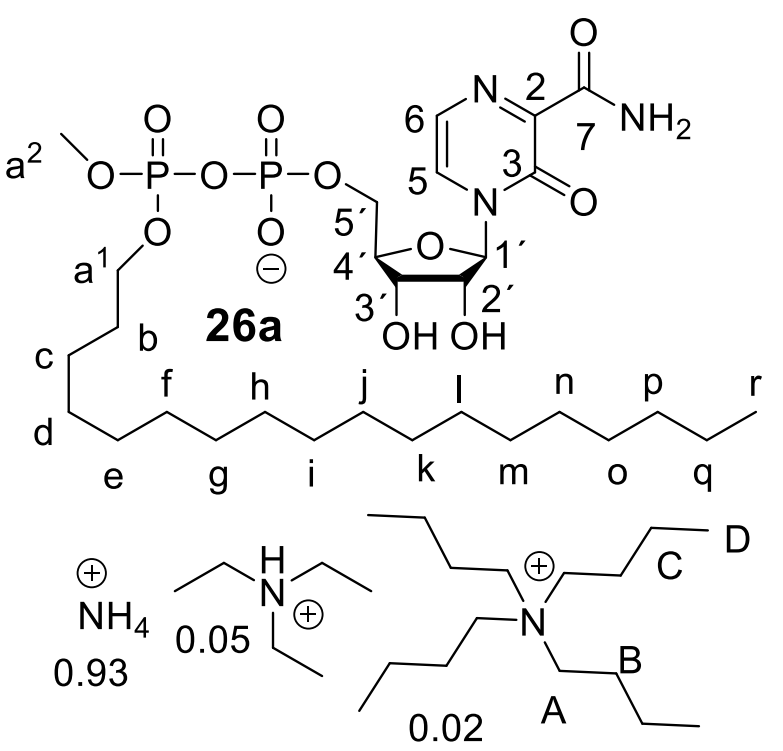

β-(Alkyl-C1;alkyl-C18)-T-1106-DP **26a**. According to general procedure with 105 mg (alkyl-C1;alkyl-C18)-*H*-phosphonate **20a** (0.3 mmol, 1.0 equiv.), 40 mg NCS (0.3 mmol, 1.0 equiv.), and 250 mg T-1106-MP 2×*n*Bu_4N^+ salt (0.3 mmol, 1.0 equiv.) and then stirred for 4 h at room temperature. Yield: 124 mg (0.17 mmol, 57%) white solid. Purity (HPLC): > 99%. ^{1}H NMR (600 MHz, CD_3OD): δ [ppm] = 8.39 (s, 1H, H-5), 7.79 (s, 1H, H-6), 6.09 (d, $^3J_{HH}$= 2.1 Hz, 1H, H-1´), 4.45-4.16 (m, 5H, H-2´, H-3´, H-4´, H-5´), 4.18-4.15 (m, 2H, H-a^1), 3.85 (dd, $^3J_{HH}$=11.6 Hz, $^4J_{HH}$=1.8 Hz, 3H, H-a^2), 3.26-3.22 (m, 0.16H, H-A), 3.22-3.15 (m, 0.30H, $HN(CH_2CH_3)_3^+$), 1.73-1.66 (m, 2.16H, H-b, H-B), 1.43-1.36 (m, 2.16H, H-c, H-C), 1.35-1.26 (m, 28.45H, H-d, H-e, H-f, H-g, H-h, H-i, H-j, H-k, H-l, H-m, H-n, H-o, H-p, H-q, $HN(CH_2CH_3)_3^+$), 1.02 (t, $^3J_{HH}$= 7.3 Hz, 0.24H, H-D), 0.90 (t, $^3J_{HH}$= 6.9 Hz, 3H, H-r). ^{13}C-NMR (151 MHz, CD_3OD): δ [ppm] = 166.3 (C-7), 157.1 (C-3), 143.8 (C-2), 130.8 (C-5), 126.1 (C-6), 92.3 (C-1´), 84.5 (d, $^3J_{CP}$= 9.7 Hz, C-4´), 76.9 (C-2´), 69.8 (d, $^3J_{CP}$= 6.0 Hz, C-a^1), 69.4 (C-3´), 65.0 (d, $^3J_{CP}$= 6.2 Hz, C-5´), 55.5 (d, $^3J_{CP}$= 6.8 Hz, C-a^2), 33.1, 30.78, 30.75, 30.73, 30.69, 30.5, 23.7 (C-d, C-e, C-f, C-g, C-h, C-i, C-j, C-k, C-l, C-m, C-n, C-o, C-p, C-q), 31.3 (d, $^3J_{CP}$ = 6.9 Hz, C-b), 26.6 (C-c), 14.4 (C-r). ^{31}P NMR (243 MHz, CD_3OD):

δ [ppm] = -11.5 -- -11.0 (m, 1P, P-β), -12.03 (d, $^2J_{pp}$= 20.6 Hz, P-α). HRMS (ESI$^-$, m/z): $C_{29}H_{53}N_3O_{12}P_2$, [M-H]$^-$ 696.3032; found 696.3025.

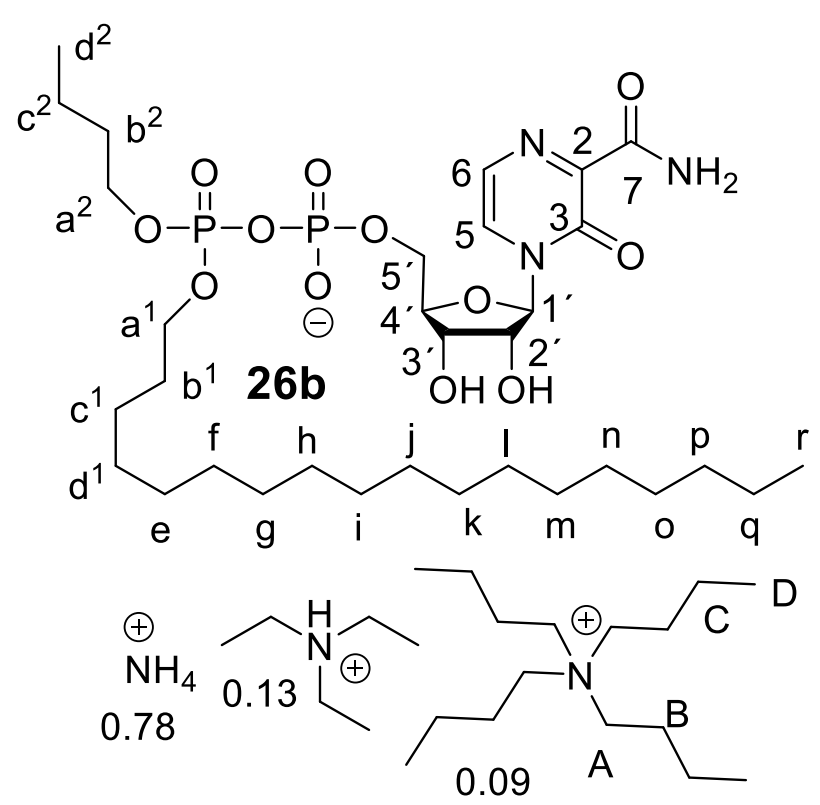

β-(Alkyl-C4;alkyl-C18)-T-1106-DP **26b**. According to general procedure with 117 mg (alkyl-C4;alkyl-C18)-*H*-phosphonate **20b** (0.3 mmol, 1.0 equiv.), 40 mg NCS (0.3 mmol, 1.0 equiv.), and 250 mg T-1106-MP 2×*n*Bu_4N^+ salt (0.3 mmol, 1.0 equiv.) and then stirred for 4 h at room temperature. Yield: 132 mg (0.17 mmol, 56%) white solid. Purity (HPLC): > 99%. ^{1}H NMR (600 MHz, CD_3OD): δ [ppm] = 8.39 (s, 1H, H-5), 7.80 (s, 1H, H-6), 6.10 (d, $^3J_{HH}$= 2.1 Hz, 1H, H-1´), 4.45-4.18 (m, 5H, H-2´, H-3´, H-4´, H-5´), 4.18-4.10 (m, 4H, H-a^1, H-a^2), 3.26-3.22 (m, 1.5H, H-A, $HN(CH_2CH_3)_3^+$), 1.73-1.66 (m, 4.72H, H-b^1, H-b^2, H-B), 1.43-1.36 (m, 4.72H, H-c^1, H-c^2, H-C), 1.35-1.26 (m, 29.2H, H-d^1, H-e, H-f, H-g, H-h, H-i, H-j, H-k, H-l, H-m, H-n, H-o, H-p, H-q, $HN(CH_2CH_3)_3^+$), 1.03 (t, $^3J_{HH}$= 7.3 Hz, 1.08H, H-D), 0.95 (dt, $^3J_{HH}$= 7.3 Hz, $^3J_{HH}$= 4.1 Hz, 3H, H-d^1), 0.90 (t, $^3J_{HH}$= 6.9 Hz, 3H, H-r). ^{13}C-NMR (151 MHz, CD_3OD): δ [ppm] = 166.3 (C-7), 157.1 (C-3), 144.0 (C-2), 130.4 (C-5), 125.9 (C-6), 92.3 (C-1´), 84.4 (d, $^3J_{CP}$= 9.7 Hz, C-4´), 76.7 (C-2´), 69.7, 69.7 (C-a^1, C-a^2), 69.3 (d, $^3J_{CP}$= 6.4 Hz, C-3´), 65.5 (d, $^3J_{CP}$= 5.2 Hz, C-5´), 59.5 (C-A), 47.8 ($HN(CH_2CH_3)_3^+$), 33.3 (d, $^3J_{CP}$= 7.0 Hz, C-b^2), 33.1, 30.78, 30.75, 30.71, 30.68, 30.5, 30.3, 23.7 (C-d, C-e, C-f, C-g, C-h, C-i, C-j, C-k, C-l, C-m, C-n, C-o, C-p, C-q), 31.3 (d, $^3J_{CP}$ = 6.9 Hz, C-b^1), 26.6 (C-c^1), 24.8 (C-B), 20.7 (C-C), 19.8 (C-c^2), 14.4 (C-r), 13.95 (C-d^1), 13.94 (C-D), 9.2 ($HN(CH_2CH_3)_3^+$). ^{31}P NMR (243 MHz, CD_3OD): δ [ppm] = -12.06 (d, $^2J_{pp}$= 20.5 Hz, P-β), -12.45 (d, $^2J_{pp}$= 20.5 Hz, P-α). HRMS (ESI$^-$, m/z): $C_{32}H_{59}N_3O_{12}P_2$, [M-H]$^-$ 738.3501; found 738.3565.

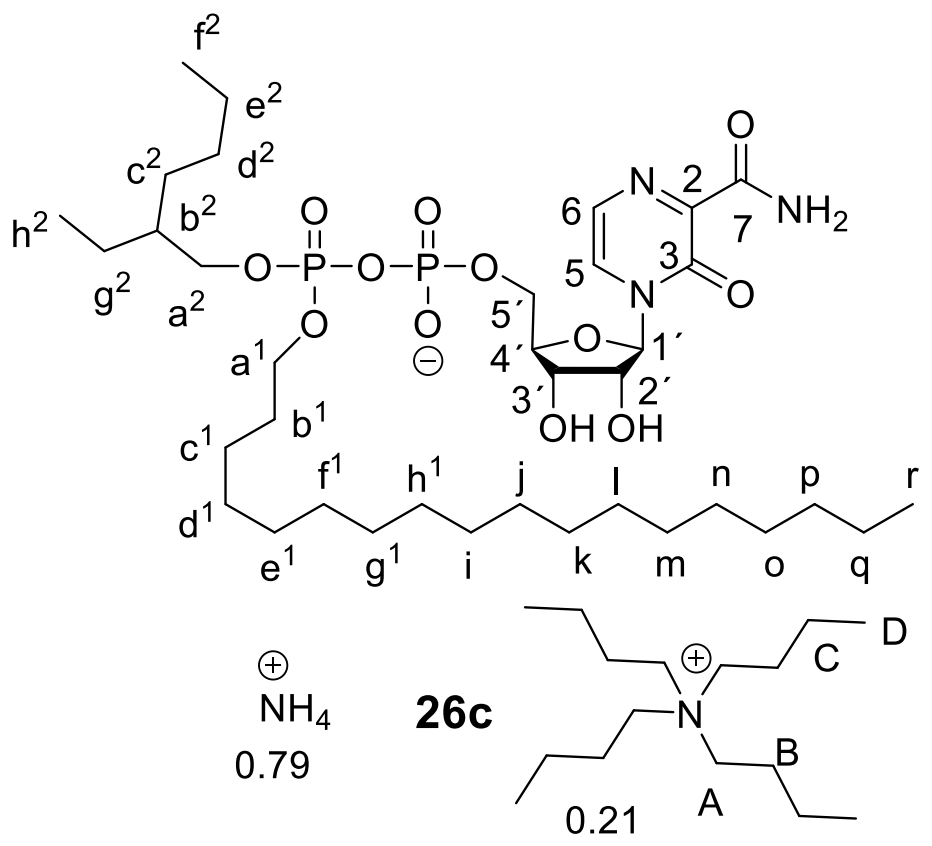

β-(Alkyl-C8;alkyl-C18)-T-1106-DP **26c**. According to general procedure with 134 mg (alkyl-C8;alkyl-C18)-*H*-phosphonate **20c** (0.3 mmol, 1.0 equiv.), 40 mg NCS (0.3 mmol, 1.0 equiv.), and 250 mg T-1106-MP 2×*n*Bu_4N^+ salt (0.3 mmol, 1.0 equiv.) and then stirred for 4 h at room temperature. Yield: 72 mg (0.08 mmol, 28%) white solid. Purity (HPLC): > 96%. ^{1}H NMR (400 MHz, CD_3OD): δ [ppm] = 8.39 (d, $^3J_{HH}$= 3.1 Hz, 1H, H-5), 7.80 (s, 1H, H-6), 6.10 (d, $^3J_{HH}$= 2.1 Hz, 1H, H-1´), 4.45-4.18 (m, 5H, H-2´, H-3´, H-4´, H-5´), 4.18-4.05 (m, 4H, H-a^1, H-a^2), 3.26-3.22 (m, 1.68H, H-A), 1.73-1.66 (m, 3.68H, H-b^1, H-B), 1.65-1.56 (m, 1.0H, H-b^2), 1.43-1.26 (m, 39.7H, H-c^1, H-c^2, H-C, H-d^1, H-d^2, H-e^1, H-e^2, H-f^1, H-g^1, H-g^2, H-h^1, H-i, H-j, H-k, H-l, H-m, H-n, H-o, H-p, H-q), 1.03 (t, $^3J_{HH}$= 7.3 Hz, 2.52H, H-D), 0.95-0.88 (m, 9H, H-h^2, H-f^2, H-r). ^{13}C-NMR (101 MHz, CD_3OD): δ [ppm] = 166.2 (C-7), 157.0 (C-3), 142.1 (C-2), 130.4 (C-5), 125.9 (C-6), 92.3 (C-1´), 84.4 (C-4´), 76.8 (C-2´), 71.6 (d, $^3J_{CP}$= 6.9 Hz, C-a^2), 69.8, 69.7 (C-a^1, C-3´), 65.5 (C-5´), 59.5 (C-A), 41.4 (d, $^3J_{CP}$= 7.7 Hz, C-b^2), 33.1, 31.1, 30.78, 30.69, 30.5, 30.3, 30.0, 24.4, 24.1, 23.7 (C-c^2, C-d^1, C-d^2, C-e^1, C-e^2, C-f^1, C-g^1, C-g^2, C-h^1, C-i, C-j, C-k, C-l, C-m, C-n, C-o, C-p, C-q), 31.3 (d, $^3J_{CP}$ = 6.9 Hz, C-b^1), 26.6 (C-c^1), 24.8 (C-B), 20.7 (C-C), 14.4 (C-r), 13.9 (C-f^2), 13.94

(C-D), 11.3 (C-h^2). ^{31}P NMR (162 MHz, CD_3OD): δ [ppm] = -12.06 (d, $^2J_{pp}$= 19.6 Hz, P-β), -12.45 (d, $^2J_{pp}$= 19.6 Hz, P-α). HRMS (ESI$^-$, m/z): $C_{36}H_{67}N_3O_{12}P_2$, [M-H]$^-$ 794.4127; found 794.4082.

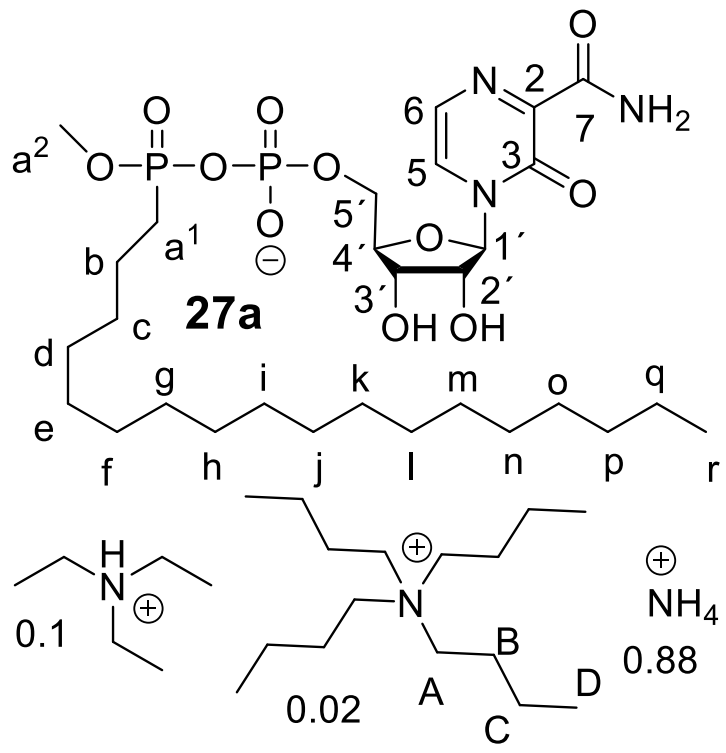

β-(Alkyl-C1)-β-(alkyl-C18)-T-1106-DP **27a**. According to general procedure with 100 mg (alkyl-C1;alkyl-C18)-*H*-phosphinate **21a** (0.3 mmol, 1.0 equiv.), 40 mg NCS (0.3 mmol, 1.0 equiv.), and 250 mg T-1106-MP 2×nBu$_4$N$^+$ salt (0.3 mmol, 1.0 equiv.) and then stirred for 4 h at room temperature. Yield: 100 mg (0.14 mmol, 47%) white solid. Purity (HPLC): > 96%. ^{1}H NMR (600 MHz, CD_3OD): δ [ppm] = 8.39 (s, 1H, H-5), 7.79 (s, 1H, H-6), 6.09 (d, $^4J_{HH}$= 1.5 Hz, 1H, H-1´), 4.45-4.16 (m, 5H, H-2´, H-3´, H-4´, H-5´), 3.83 (d, $^3J_{HH}$=11.3 Hz, 3H, H-a^2), 3.28-3.15 (m, 0.76H, H-A, $HN(CH_2CH_3)_3^+$), 2.05-1.92 (m, 2H, H-a^1), 1.71-1.56 (m, 2.16H, H-b, H-B), 1.45-1.36 (m, 2.16H, H-c, H-C), 1.35-1.26 (m, 28.9H, H-d, H-e, H-f, H-g, H-h, H-i, H-j, H-k, H-l, H-m, H-n, H-o, H-p, H-q, $HN(CH_2CH_3)_3^+$), 1.02 (t, $^3J_{HH}$= 7.3 Hz, 0.24H, H-D), 0.90 (t, $^3J_{HH}$= 6.6 Hz, 3H, H-r). ^{13}C-NMR (151 MHz, CD_3OD): δ [ppm] = 166.2 (C-7), 157.1 (C-3), 144.1 (C-2), 130.4 (C-5), 125.8 (C-6), 92.4 (C-1´), 84.3 (d, $^3J_{CP}$= 9.0 Hz, C-4´), 76.7 (C-2´), 69.7 (C-3´), 65.3 (d, $^3J_{CP}$= 5.6 Hz, C-5´), 53.2 (dd, $^3J_{CP}$= 7.3 Hz, $^4J_{CP}$= 1.1 Hz, C-a^2), 47.8 ($HN(CH_2CH_3)_3^+$), 33.1, 31.65, 31.64, 30.78, 30.75, 30.73, 30.54, 30.47, 30.3, 23.7 (C-d, C-e, C-f, C-g, C-h, C-i, C-j, C-k, C-l, C-m, C-n, C-o, C-p, C-q), 27.1, 26.1 (C-a^1), 24.8 (C-B), 23.3 (d, $^3J_{CP}$ = 5.5 H$_Z$, C-b), 20.7 (C-C), 14.4 (C-r), 13.9 (C-D), 9.2 ($HN(CH_2CH_3)_3^+$). ^{31}P NMR (243 MHz, CD_3OD): δ [ppm] = 25.52 (d, $^2J_{pp}$= 23.8 Hz, P-β), -11.62 (d, $^2J_{pp}$= 24.4 Hz, P-α). HRMS (ESI$^-$, m/z): $C_{29}H_{53}N_3O_{11}P_2$, [M-H]$^-$ 680.3083; found 680.3012.

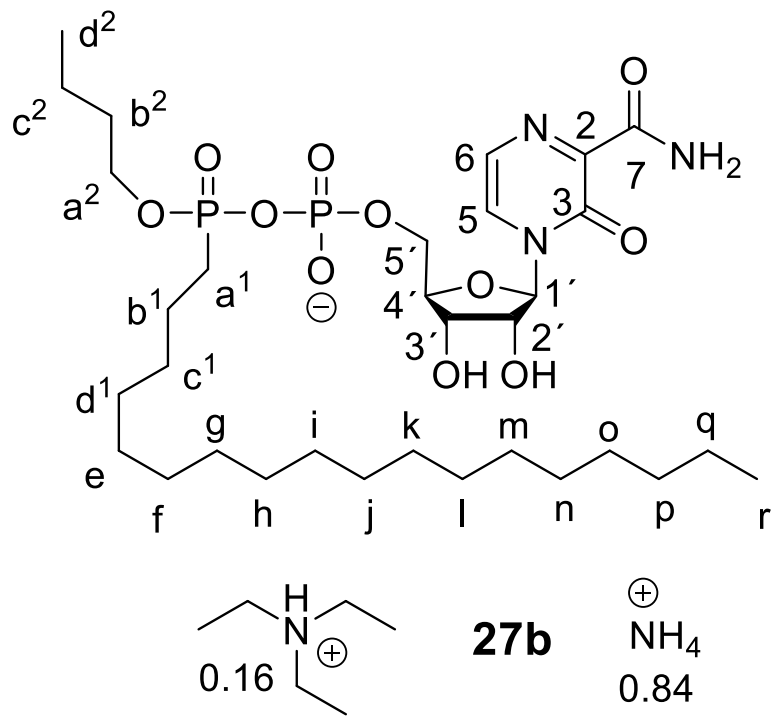

β-(Alkyl-C4)-β-(alkyl-C18)-T-1106-DP **27b**. According to general procedure with 112 mg (alkyl-C4;alkyl-C18)-*H*-phosphinate **21b** (0.3 mmol, 1.0 equiv.), 40 mg NCS (0.3 mmol, 1.0 equiv.), and 250 mg T-1106-MP 2×nBu$_4$N$^+$ salt (0.3 mmol, 1.0 equiv.) and then stirred for 4 h at room temperature. Yield: 136 mg (0.18 mmol, 60%) white solid. Purity (HPLC): > 99%. ^{1}H NMR (600 MHz, CD_3OD): δ [ppm] = 8.40 (s, 1H, H-5), 7.80 (s, 1H, H-6), 6.09 (d, $^3J_{HH}$= 2.0 Hz, 1H, H-1´), 4.45-4.10 (m, 7H, H-2´, H-3´, H-4´, H-5´, H-a^2), 3.21 (q, $^3J_{HH}$= 7.3 Hz, 0.96H, $HN(CH_2CH_3)_3^+$), 2.05-1.94 (m, 2H, H-a^1), 1.70-1.62 (m, 4H, H-b^1, H-b^2), 1.45-1.36 (m, 4H, H-c^1, H-c^2), 1.35-1.26 (m, 29.4H, H-d^1, H-e, H-f, H-g, H-h, H-i, H-j, H-k, H-l, H-m, H-n, H-o, H-p, H-q, $HN(CH_2CH_3)_3^+$), 0.94 (dt, $^3J_{HH}$= 7.6 Hz, $^4J_{HH}$= 1.6 Hz, 3H, H-d^2), 0.90 (t, $^3J_{HH}$= 6.6 Hz, 3H, H-r). ^{13}C-NMR (151 MHz, CD_3OD): δ [ppm] = 166.2 (C-7), 157.0 (C-3), 144.0 (C-2), 130.4 (C-5), 125.8 (C-6), 92.4 (C-1´), 84.3 (d, $^3J_{CP}$= 9.0 Hz, C-4´), 76.7 (C-2´), 69.6 (d, $^3J_{CP}$= 6.3 Hz, C-3´), 67.0 (dd, $^3J_{CP}$= 7.3 Hz, $^3J_{CP}$= 2.2 Hz, C-a^2), 65.3 (t, $^3J_{CP}$= 5.9 Hz, C-5´), 47.8 ($HN(CH_2CH_3)_3^+$), 33.5 (d, $^3J_{CP}$= 6.7 Hz, C-b^2), 33.1, 31.59, 31.58, 31.48, 31.47, 30.77, 30.75, 30.71, 30.52, 30.46, 30.2, 23.7 (C-c^1, C-d^1, C-e, C-f, C-g, C-h, C-i, C-j, C-k, C-l, C-m, C-n, C-o, C-p, C-q), 27.4, 26.4 (C-a^1), 23.4 (d, $^3J_{CP}$ = 5.6 H$_Z$, C-b^1), 19.8 (C-c^2), 14.4 (C-r), 14.0 (C-d^2), 9.2 ($HN(CH_2CH_3)_3^+$). ^{31}P NMR (243 MHz, CD_3OD): δ [ppm] = 24.12 (dd, $^2J_{pp}$= 26.4 Hz, $^3J_{pp}$= 5.7 Hz, P-β), -11.96 (dd, $^2J_{pp}$= 24.4 Hz, $^3J_{pp}$= 5.7 Hz, P-α). HRMS (ESI$^-$, m/z): $C_{32}H_{59}N_3O_{11}P_2$, [M-H]$^-$ 722.3552; found 722.3500.

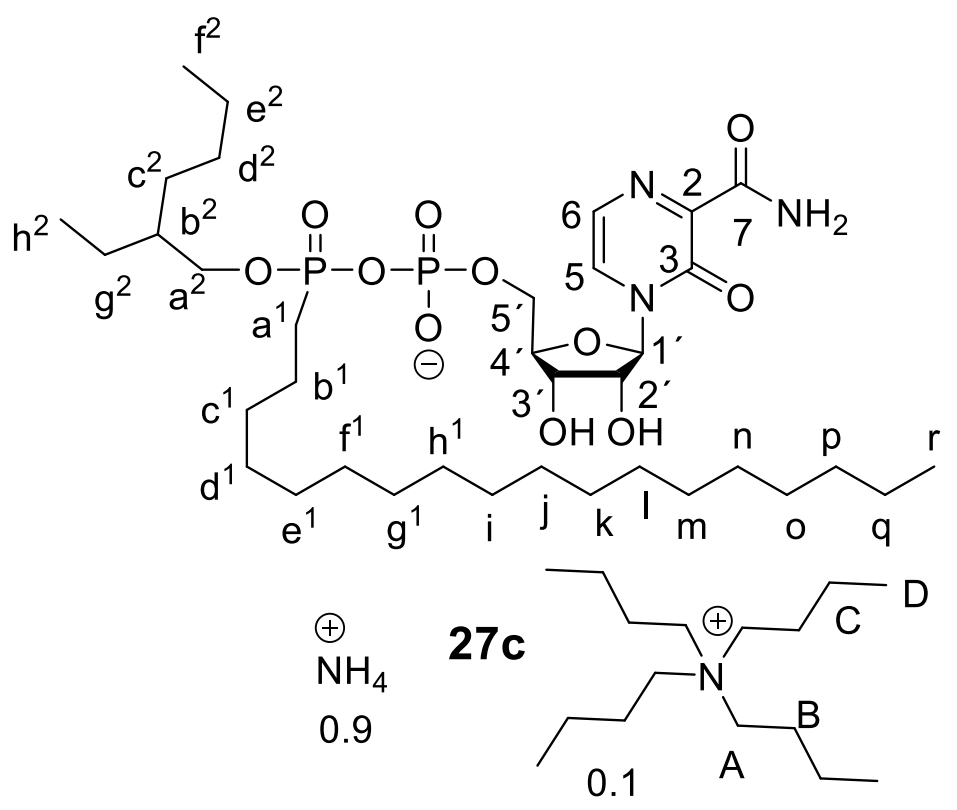

β-(Alkyl-C8)-*β*-(alkyl-C18)-T-1106-DP **27c**. According to general procedure with 129 mg (alkyl-C8;alkyl-C18)-*H*-phosphinate **21c** (0.3 mmol, 1.0 equiv.), 40 mg NCS (0.3 mmol, 1.0 equiv.), and 250 mg T-1106-MP 2×*n*Bu_4N^+ salt (0.3 mmol, 1.0 equiv.) and then stirred for 4 h at room temperature. Yield: 84 mg (0.1 mmol, 34%) white solid. Purity (HPLC): > 96%. ^{1}H NMR (400 MHz, CD_3OD): δ [ppm] = 8.4a (s, 1H, H-5), 7.8a (s, 1H, H-6), 6.09 (d, $^4J_{HH}$= 1.8 Hz, 1H, H-1´), 4.45-4.10 (m, 7H, H-2´, H-3´, H-4´, H-5´, H-a^2), 3.28-3.20 (m, 0.8H, H-D), 2.05-1.94 (m, 2H, H-a^1), 1.70-1.62 (m, 2.8 H, H-b^1, H-B), 1.60-1.54 (m, 1H, H-b^2), 1.45-1.26 (m, 38.8H, H-C, H-c^1, H-c^2, H-d^1, H-d^2, H-e^1, H-e^2, H-f^1, H-g^1, H-g^2, H-h^1, H-i, H-j, H-k, H-l, H-m, H-n, H-o, H-p, H-q), 1.02 (t, $^3J_{HH}$= 7.2 Hz, 3H, H-D), 0.95-0.88 (m, 9H, H-h^2, H-f^2, H-r). ^{13}C-NMR (101 MHz, CD_3OD): δ [ppm] = 166.2 (C-7), 157.0 (C-3), 144.0 (C-2), 130.4 (C-5), 125.8 (d, $^3J_{CP}$= 4.3 Hz, C-6), 92.4 (C-1´), 84.4 (dd, $^3J_{CP}$= 9.0 Hz, $^3J_{CP}$= 5.4 Hz, C-4´), 76.7 (d, $^4J_{CP}$= 1.8 Hz, C-2´), 69.7 (d, $^3J_{CP}$= 6.3 Hz, C-3´), 69.1 (dd, $^3J_{CP}$= 8.0 Hz, $^3J_{CP}$= 3.5 Hz, C-a^2), 65.5 (dd, $^3J_{CP}$= 9.6 Hz, $^3J_{CP}$= 6.2 Hz, C-5´), 59.5 (t, $^3J_{CP}$= 2.8 Hz, C-A), 41.4 (dd, $^3J_{CP}$= 7.0 Hz, $^4J_{CP}$= 1.4 Hz, C-b^2), 33.1, 31.6, 31.4, 31.18, 31.14, 30.78, 30.71, 30.49, 30.47, 30.2, 30.1, 30.0, 24.45, 24.41, 24.1, 23.7 (C-c^1, C-c^2, C-d^1, C-d^2, C-e^1, C-e^2, C-f^1, C-g^1, C-g^2, C-h^1, C-i, C-j, C-k, C-l, C-m, C-n, C-o, C-p, C-q), 27.6, 26.2 (C-a^1), 24.8 (C-B), 23.5 (d, $^3J_{CP}$ = 5.6 Hz, C-b^1), 20.7 (C-C), 14.45, 14.43 (C-r, C-f^2), 13.9 (C-D), 11.3 (d, $^3J_{CP}$= 5.7 Hz, C-h^2). ^{31}P NMR (162 MHz, CD_3OD): δ [ppm] = 24.3-24.0 (m, 1P, P-*β*), -11.95 (dd, $^2J_{pp}$= 24.4 Hz, $^3J_{pp}$= 5.7 Hz, P-*α*). HRMS (ESI$^-$, m/z): $C_{36}H_{67}N_3O_{11}P_2$, [M-H]$^-$ 778.4178; found 778.4193.

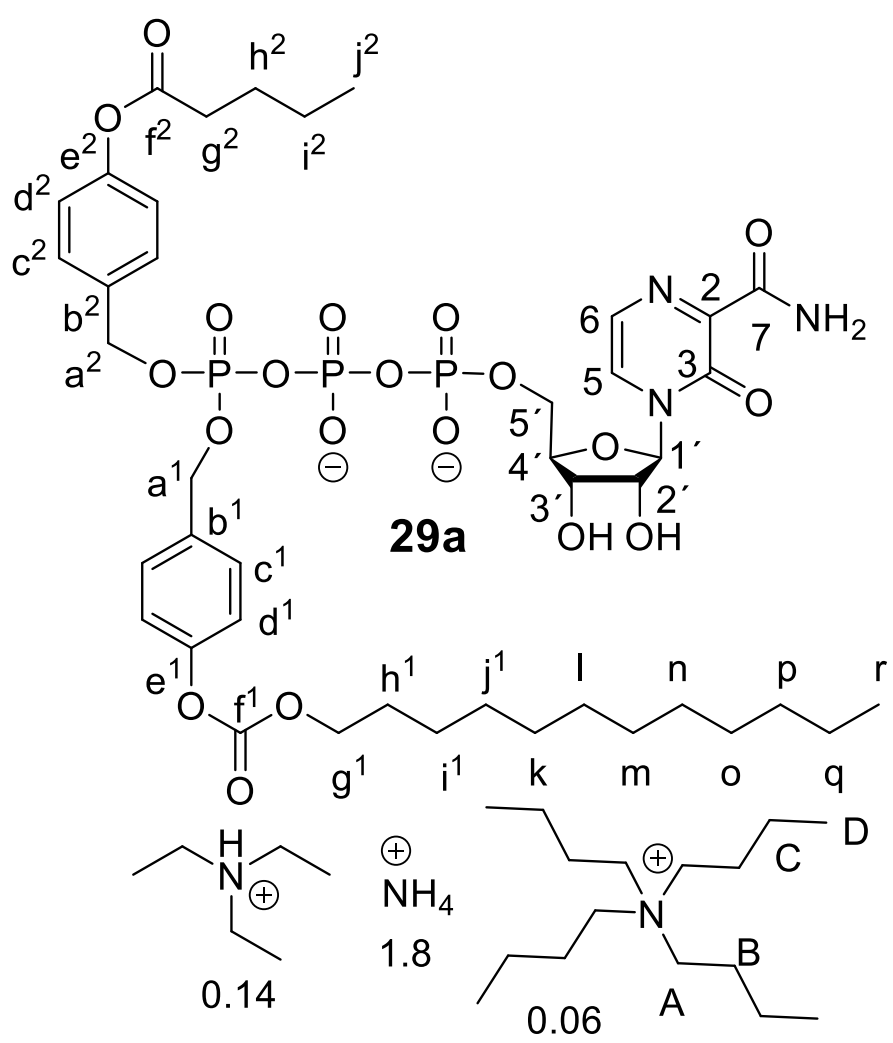

γ-(AB-C4;ACB-C12)-T-1106-TP **29a**. According to general procedure with 177 mg (AB-C4;ACB-C12)-*H*-phosphonate **17a** (0.3 mmol, 1.0 equiv.), 40 mg NCS (0.3 mmol, 1.0 equiv.), and 346 mg T-1106-DP 3×*n*Bu_4N^+ salt (0.3 mmol, 1.0 equiv.) and then stirred for 4 h at room temperature. Yield: 139 mg (0.13 mmol, 43%) white solid. Purity (HPLC): > 96%. ^{1}H NMR (600 MHz, CD_3OD): δ [ppm] = 8.44 (d, $^3J_{HH}$= 4.2 Hz, 1H, H-5), 7.80 (d, $^3J_{HH}$= 4.2 Hz, 1H, H-6), 7.44-7.36 (m, 4H, H-c^1, H-c^2), 7.16-7.11 (m, 2H, H-d^1), 7.05-7.02 (m, 2H, H-d^2), 6.04 (d, $^3J_{HH}$= 2.0 Hz, 1H, H-1´), 5.16-5.10 (m, 4H, H-a^1, H-a^2), 4.45-4.16 (m, 7H, H-2´, H-3´, H-4´, H-5´, H-g^1), 3.28-3.20 (m, 1.32H, H-A, $HN(CH_2CH_3)_3^+$), 2.57 (t, $^3J_{HH}$= 7.5 Hz, 2H, H-h^2), 1.75-1.65 (m, 4.48H, H-B, H-h^1, H-h^2), 1.48-1.37 (m, 4.48H, H-C, H-i^1, H-i^2), 1.35-1.25 (m, 17.23H, H-j^1, H-k, H-l, H-m, H-n, H-o, H-p, H-q), 1.02 (t, $^3J_{HH}$= 7.4 Hz, 0.72H, H-D), 0.99 (t, $^3J_{HH}$= 7.3 Hz, 3H, H-j^2), 0.90 (t, $^3J_{HH}$= 6.8 Hz, 3H, H-r). ^{13}C NMR (151 MHz, CD_3OD): δ [ppm] = 173.7 (C-f^2), 166.3 (C-7), 157.9 (C-3), 155.1 (C-f^1), 152.7, 152.4 (C-e^1, C-e^2), 143.7 (C-2), 135.1 (C-b^1), 133.8 (C-b^2), 130.55, 130.47, 130.44 (C-5, C-c^1, C-c^2), 126.1 (C-6), 122.8 (C-d^2), 122.3 (C-d^1), 92.3 (C-1´), 84.1 (C-4´), 76.9 (C-2´), 70.4, 70.3 (C-a^1, C-a^2), 70.0 (C-g^1), 69.4 (C-3´), 65.0 (C-5´), 34.8

(C-g[2]), 33.1, 30.76, 30.69, 30.63, 30.5, 30.3, 23.7 (C-j[1], C-k, C-l, C-m, C-n, C-o, C-p, C-q), 29.7 (C-h[1]), 28.1 (C-h[2]), 26.8 (C-i[1]), 23.3 (C-i[2]), 14.5 (C-r), 14.1 (C-j[2]), 13.9 (C-D). ^{31}P NMR (243 MHz, CD_3OD): δ [ppm] = -11.58 (d, $^2J_{pp}$= 20.5 Hz, P-α), -13.18 (d, $^2J_{pp}$= 17.6, P-γ), -23.61 (t, $^3J_{pp}$= 19.8 Hz, P-β). HRMS (ESI$^-$, m/z): $C_{42}H_{60}N_3O_{20}P_3$, [M-H]$^-$ 1018.2910; found 1018.2906.

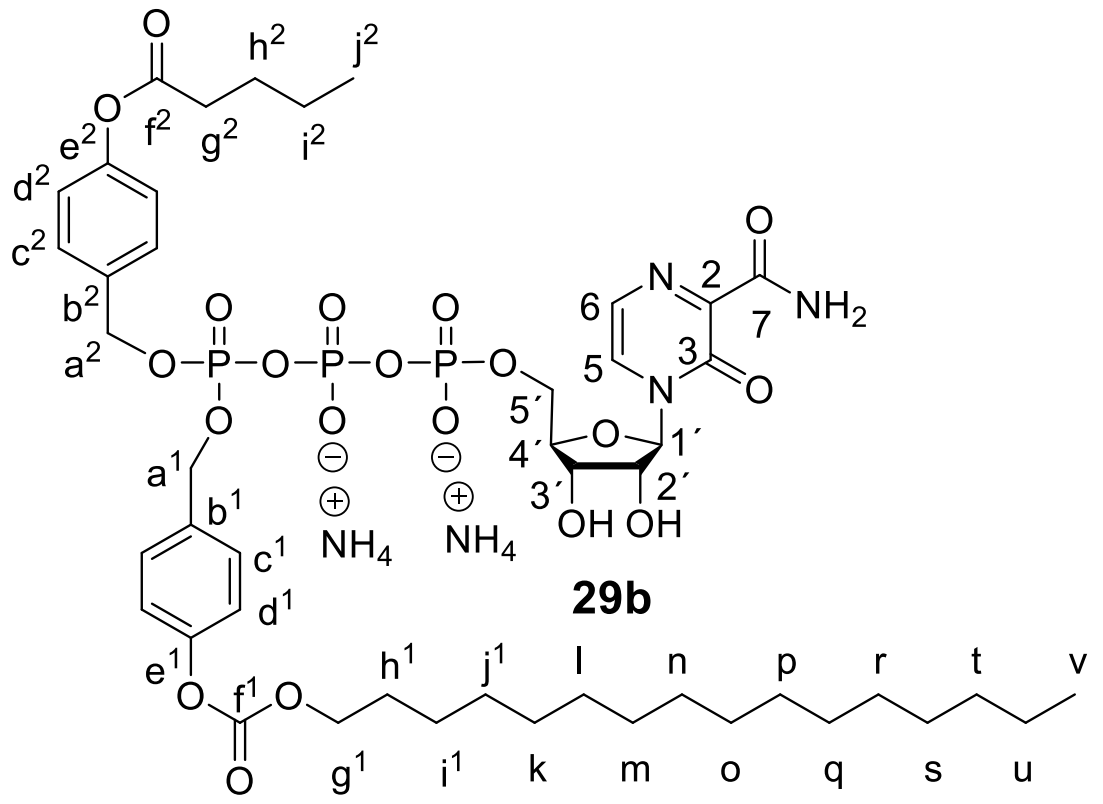

γ-(AB-C4;ACB-C16)-T-1106-TP **29b**. According to general procedure with 194 mg (AB-C4;ACB-C16)-*H*-phosphonate **17b** (0.3 mmol, 1.0 equiv.), 40 mg NCS (0.3 mmol, 1.0 equiv.), and 346 mg T-1106-DP 3×nBu$_4$N$^+$ salt (0.3 mmol, 1.0 equiv.) and then stirred for 4 h at room temperature. Yield: 156 mg (0.14 mmol, 47%) white solid. Purity (HPLC): > 96%. ^{1}H NMR (400 MHz, CD_3OD): δ [ppm] = 8.42 (d, $^3J_{HH}$= 4.2 Hz, 1H, H-5), 7.79 (d, $^3J_{HH}$= 4.2 Hz, 1H, H-6), 7.44-7.36 (m, 4H, H-c[1], H-c[2]), 7.16-7.10 (m, 2H, H-d[1]), 7.05-7.02 (m, 2H, H-d[2]), 6.05 (d, $^3J_{HH}$= 2.0 Hz, 1H, H-1´), 5.20-5.10 (m, 4H, H-a[1], H-a[2]), 4.45-4.10 (m, 7H, H-2´, H-3´, H-4´, H-5´, H-g[1]), 2.58 (t, $^3J_{HH}$= 7.5 Hz, 2H, H-h[2]), 1.75-1.65 (m, 4H, H-h[1], H-h[2]), 1.48-1.39 (m, 4H, H-i[1], H-i[2]), 1.35-1.25 (m, 24H, H-j[1], H-k, H-l, H-m, H-n, H-o, H-p, H-q, H-r, H-s, H-t, H-u), 0.99 (t, $^3J_{HH}$= 7.3 Hz, 3H, H-j[2]), 0.90 (t, $^3J_{HH}$= 6.4 Hz, 3H, H-v). ^{13}C NMR (101 MHz, CD_3OD): δ [ppm] = 173.7 (C-f[2]), 166.3 (C-7), 157.0 (C-3), 155.1 (C-f[1]), 152.7 (C-e[1]), 152.4 (C-e[2]), 144.0 (C-2), 135.1 (C-b[1]), 134.8 (d, $^3J_{CP}$= 9.8 Hz, C-b[2]), 130.51, 130.48, 130.44, 130.3 (C-5, C-c[1], C-c[2]), 125.9 (C-6), 122.9 (C-d[2]), 122.3 (C-d[1]), 92.4 (C-1´), 84.3 (d, $^3J_{CP}$= 9.6 Hz, C-4´), 76.7 (C-2´), 70.4, 70.2 (2 × dd , $^3J_{CP}$= 2.4 Hz, $^3J_{CP}$= 5.8 Hz, $^3J_{CP}$= 2.3 Hz, $^3J_{CP}$= 5.6 Hz, C-a[1], C-a[2]), 70.0 (C-g[1]), 69.7 (C-3´), 65.5 (dd, $^3J_{CP}$= 5.6 Hz, $^4J_{CP}$= 1.8 Hz, C-5´), 59.5 (t, $^3J_{CP}$= 2.8 Hz, C-A), 34.7 (C-g[2]), 33.1, 30.79, 30.78, 30.76, 30.74, 30.68, 30.63, 30.5, 30.3, 23.7 (C-j[1], C-k, C-l, C-m, C-n, C-o, C-p, C-q, C-r, C-s, C-t, C-u), 29.7 (C-h[1]), 28.1 (C-h[2]), 26.8 (C-i[1]), 24.8 (C-B), 23.3 (C-i[2]), 20.7 (C-C), 14.4 (C-r), 14.1 (C-j[2]), 13.9 (C-D). ^{31}P NMR (162 MHz, CD_3OD): δ [ppm] = -11.68 (d, $^2J_{pp}$= 18.0 Hz, P-α), -13.16 (d, $^2J_{pp}$= 18.0, P-γ), -23.60 (t, $^3J_{pp}$= 19.6 Hz, P-β). HRMS (ESI$^-$, m/z): $C_{46}H_{68}N_3O_{20}P_3$, [M-H]$^-$ 1074.3536; found 1074.3521.

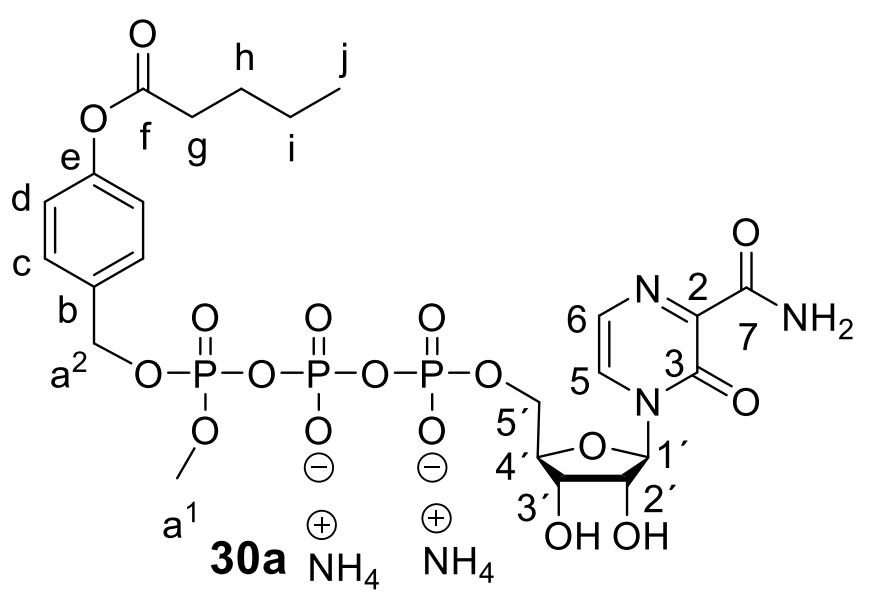

γ-(AB-C4;alkyl-C1)-T-1106-TP **30a**. According to general procedure with 86 mg (AB-C4;alkyl-C1)-*H*-phosphonate **18a** (0.3 mmol, 1.0 equiv.), 40 mg NCS (0.3 mmol, 1.0 equiv.), and 346 mg T-1106-DP 3×nBu$_4$N$^+$ salt (0.3 mmol, 1.0 equiv.) and then stirred for 4 h at room temperature. Yield: 70 mg (0.09 mmol, 31%) white solid. Purity (HPLC): > 96%. ^{1}H NMR (400 MHz, CD_3OD): δ [ppm] = 8.45 (d, $^3J_{HH}$= 4.1 Hz, 1H, H-5), 7.80 (d, $^3J_{HH}$= 4.1 Hz, 1H, H-6), 7.48-7.45 (m, 2H, H-c), 7.10-7.04 (m, 2H, H-d), 6.08-6.04 (m, 1H, H-1´), 5.18 (dd, $^3J_{HH}$= 8.0 Hz, $^3J_{HH}$= 2.1 Hz, 2H, H-a[2]), 4.46-4.16 (m, 5H, H-2´, H-3´, H-4´, H-5´), 3.81 (dd, $^2J_{HH}$= 11.8 Hz, $^3J_{HH}$= 2.0 Hz, 3H, H-a[1]), 2.57 (t, $^3J_{HH}$= 7.4 Hz, 2H, H-g), 1.75-1.66 (m, 2H, H-h), 1.50-1.40 (m, 2H, H-i), 0.98 (t, $^3J_{HH}$= 7.3 Hz, 3H, H-j). ^{13}C-NMR (101 MHz, CD_3OD): δ [ppm] = 173.7 (C-f), 166.4 (C-7), 157.1 (C-3), 152.3 (C-e), 143.8 (C-2), 135.0 (d, $^3J_{CP}$= 7.6 Hz, C-b), 130.7 (C-d), 130.4 (C-5), 126.0 (C-6), 122.8 (C-c), 92.4 (C-1´), 84.5 (d,

$^3J_{CP}$= 9.9 Hz, C-4´), 76.9 (C-2´), 70.2 (d, $^3J_{CP}$= 5.5 Hz, C-a^2), 69.3 (d, $^3J_{CP}$= 3.2 Hz, C-3´), 65.0 (d, $^3J_{CP}$= 4.5 Hz, C-5´), 55.5 (d, $^3J_{CP}$= 6.7 Hz, C-a^1), 34.7 (C-g), 28.1 (C-h), 23.2 (C-i), 14.1 (C-j). ^{31}P NMR (162 MHz, CD_3OD): δ [ppm] = -11.50 -- -12.10 (m, 2P, P-α, P-γ), -23.72 (d, $^2J_{pp}$= 17.5 Hz, P-β). HRMS (ESI$^-$, m/z): $C_{23}H_{32}N_3O_{17}P_3$, [M-H]$^-$ 714.0872; found 714.0885.

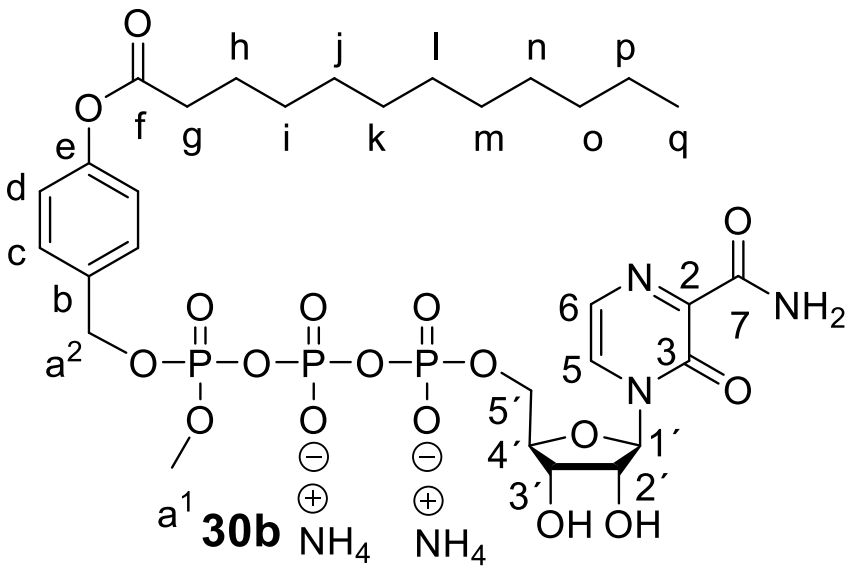

γ-(AB-C11;alkyl-C1)-T-1106-TP **30b**. According to general procedure with 115 mg (AB-C11;alkyl-C1)-*H*-phosphonate **18b** (0.3 mmol, 1.0 equiv.), 40 mg NCS (0.3 mmol, 1.0 equiv.), and 346 mg T-1106-DP 3×nBu$_4$N$^+$ salt (0.3 mmol, 1.0 equiv.) and then stirred for 4 h at room temperature. Yield: 104 mg (0.12 mmol, 41%) white solid. Purity (HPLC): > 96%. ^{1}H NMR (400 MHz, CD_3OD): δ [ppm] = 8.43 (d, $^3J_{HH}$= 4.2 Hz, 1H, H-5), 7.80 (d, $^3J_{HH}$= 4.2 Hz, 1H, H-6), 7.48-7.44 (m, 2H, H-c), 7.10-7.03 (m, 2H, H-d), 6.06 (d, $^4J_{HH}$= 1.0 Hz, 1H, H-1´), 5.18 (dd, $^3J_{HH}$= 7.8 Hz, $^4J_{HH}$= 1.8 Hz, 2H, H-a^2), 4.44-4.16 (m, 5H, H-2´, H-3´, H-4´, H-5´), 3.81 (dd, $^2J_{HH}$= 11.6 Hz, $^4J_{HH}$= 1.9 Hz, 3H, H-a^1), 2.60-2.54 (m, 2H, H-g), 1.75-1.68 (m, 2H, H-h), 1.46-1.25 (m, 16H, H-i, H-j, H-k, H-l, H-m, H-n, H-o, H-p), 0.90 (t, $^3J_{HH}$= 6.9 Hz, 3H, H-j). ^{13}C-NMR (101 MHz, CD_3OD): δ [ppm] = 173.8 (C-f), 166.3 (C-7), 157.1 (C-3), 152.3 (C-e), 143.8 (C-2), 135.1 (d, $^3J_{CP}$= 7.6 Hz, C-b), 130.7 (d, $^2J_{CP}$= 24.6 Hz, C-d), 130.35 (C-5), 126.0 (C-6), 122.8 (C-c), 92.4 (C-1´), 84.5 (d, $^3J_{CP}$= 9.1 Hz, C-4´), 76.8 (C-2´), 70.3 (d, $^3J_{CP}$= 5.7 Hz, C-a^2), 69.5 (d, $^3J_{CP}$= 2.7 Hz, C-3´), 65.0 (t, $^3J_{CP}$= 4.7 Hz, C-5´), 55.6 (d, $^3J_{CP}$= 6.3 Hz, C-a^1), 35.0 (C-g), 33.1, 30.7, 30.6, 30.5, 30.4, 30.2, 23.7 (C-i, C-j, C-k, C-l, C-m, C-n, C-o, C-p), 25.9 (C-h), 14.4 (C-q). ^{31}P NMR (162 MHz, CD_3OD): δ [ppm] = -11.50 -- -12.10 (m, 2P, P-α, P-γ), -23.71 (d, $^2J_{pp}$= 18.5 Hz, P-β). HRMS (ESI$^-$, m/z): $C_{30}H_{46}N_3O_{17}P_3$, [M-H]$^-$ 812.1967; found 812.1972.

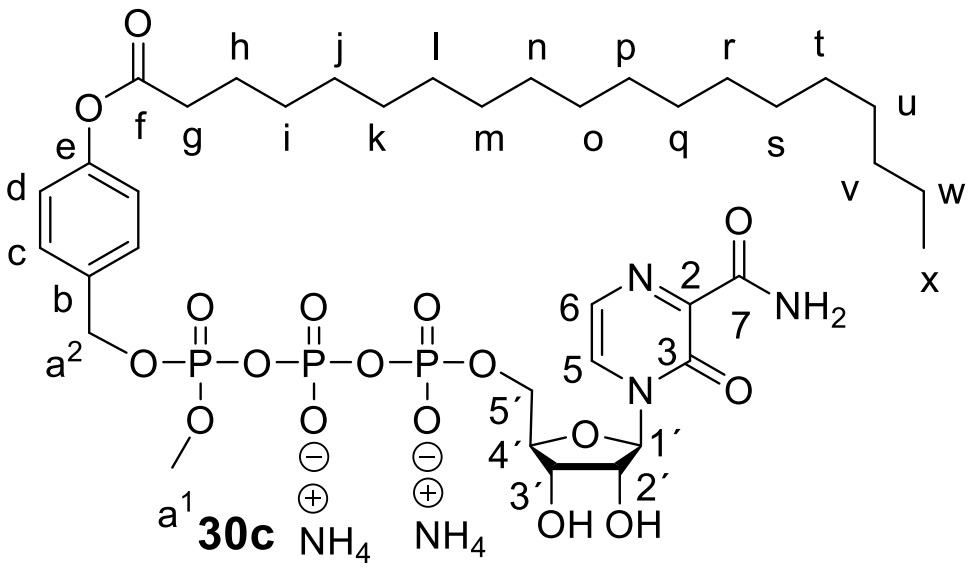

γ-(AB-C17;alkyl-C1)-T-1106-TP **30c**. According to general procedure with 145 mg (AB-C17;alkyl-C1)-*H*-phosphonate **18c** (0.3 mmol, 1.0 equiv.), 40 mg NCS (0.3 mmol, 1.0 equiv.), and 346 mg T-1106-DP 3×nBu$_4$N$^+$ salt (0.3 mmol, 1.0 equiv.) and then stirred for 4 h at room temperature. Yield: 66 mg (0.07 mmol, 23%) white solid. Purity (HPLC): > 95%. ^{1}H NMR (600 MHz, CD_3OD): δ [ppm] = 8.48 (d, $^3J_{HH}$= 4.1 Hz, 1H, H-5), 7.83 (t, $^3J_{HH}$= 4.1 Hz, 1H, H-6), 7.52-7.45 (m, 2H, H-c), 7.12-7.05 (m, 2H, H-d), 6.08 (d, $^4J_{HH}$= 1.7 Hz, 1H, H-1´), 5.25-5.18 (m, 2H, H-a^2), 4.44-4.16 (m, 5H, H-2´, H-3´, H-4´, H-5´), 3.81 (dd, $^2J_{HH}$= 11.6 Hz, $^4J_{HH}$= 1.6 Hz, 3H, H-a^1), 2.59 (t, $^3J_{HH}$= 7.5 Hz, 2H, H-g), 1.78-1.70 (m, 2H, H-h), 1.38-1.25 (m, 30H, H-i, H-j, H-k, H-l, H-m, H-n, H-o, H-p, H-q, H-r, H-s, H-t, H-u, H-v, H-w), 0.92 (t, $^3J_{HH}$= 6.9 Hz, 3H, H-x). ^{13}C-NMR (151 MHz, CD_3OD): δ [ppm] = 173.8 (C-f), 157.1 (C-3), 152.3 (C-e), 143.7 (C-2), 135.1 (C-b), 130.8 (C-d), 130.4 (C-5), 126.1 (C-6), 122.8 (C-c), 92.3 (C-1´), 84.6 (C-4´), 76.8 (C-2´), 70.2 (C-a^2), 69.4 (C-3´), 65.0 (C-5´), 55.5 (d, $^3J_{CP}$= 6.2 Hz, C-a^1), 35.0 (C-g), 33.1, 30.77, 30.75, 30.72, 30.6, 30.5, 30.4, 30.2, 23.7 (C-i, C-j, C-k, C-l, C-m, C-n, C-o, C-p, C-q, C-r, C-s, C-t, C-u, C-v, C-w), 26.0 (C-h), 14.4 (C-x). ^{31}P NMR (243 MHz, CD_3OD): δ [ppm] = -11.50 -- -12.10 (m, 2P, P-α, P-γ), -23.71 (d, $^2J_{pp}$= 18.5 Hz, P-β). HRMS (ESI$^-$, m/z): $C_{37}H_{58}N_3O_{17}P_3$, [M-H]$^-$ 896.2906; found 896.2931.

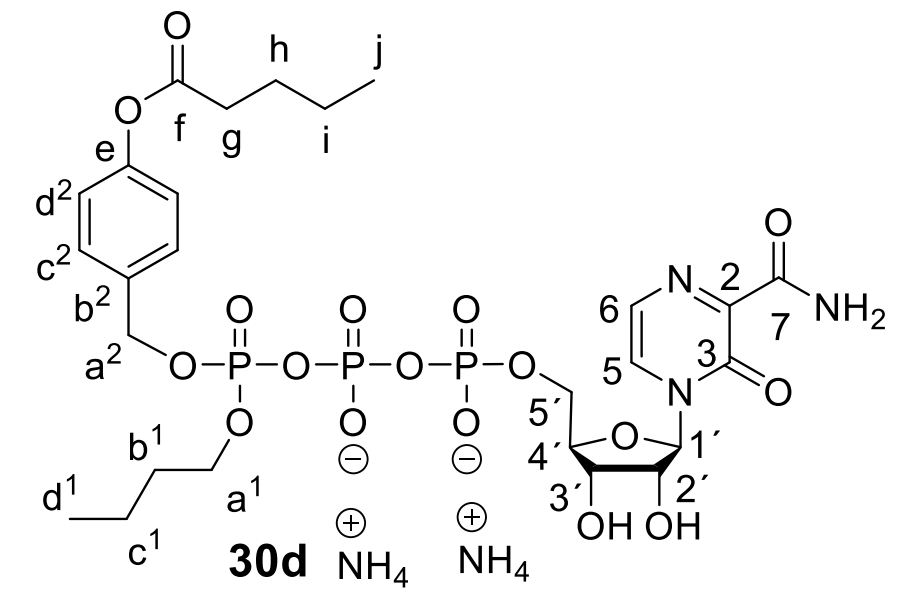

γ-(AB-C4;alkyl-C4)-T-1106-TP **30d**. According to general procedure with 99 mg (AB-C4;alkyl-C4)-*H*-phosphonate **18d** (0.3 mmol, 1.0 equiv.), 40 mg NCS (0.3 mmol, 1.0 equiv.), and 346 mg T-1106-DP 3×*n*Bu_4N^+ salt (0.3 mmol, 1.0 equiv.) and then stirred for 4 h at room temperature. Yield: 65 mg (0.15 mmol, 51%) white solid. Purity (HPLC): > 96%. ^{1}H NMR (600 MHz, CD_3OD): δ [ppm] = 8.46 (d, $^3J_{HH}$= 4.2 Hz, 1H, H-5), 7.75 (dd, $^3J_{HH}$= 4.2 Hz, $^4J_{HH}$= 1.0 Hz, 1H, H-6), 7.50-7.44 (m, 2H, H-c^2), 7.10-7.03 (m, 2H, H-d^2), 6.06 (d, $^4J_{HH}$= 1.9 Hz, 1H, H-1´), 5.20-5.16 (m, 2H, H-a^2), 4.44-4.14 (m, 5H, H-2´, H-3´, H-4´, H-5´), 4.15-4.08 (m, 2H, H-a^1), 2.58 (t, $^3J_{HH}$= 7.4 Hz, 2H, H-g), 1.74-1.66 (m, 2H, H-h), 1.66-1.60 (m, 2H, H-b^1), 1.48-1.41 (m, 2H, H-i), 1.41-1.32 (m, 2H, H-c^1), 0.98 (t, $^3J_{HH}$= 7.4 Hz, 3H, H-j), 0.90 (dt, $^3J_{HH}$= 7.4 Hz, $^4J_{HH}$= 1.3 Hz, 3H, H-d^1). ^{13}C-NMR (151 MHz, CD_3OD): δ [ppm] = 173.7 (C-f), 166.3 (C-7), 157.1 (C-3), 152.3 (C-e), 143.7 (C-2), 135.2 (C-b^2), 130.7 (C-d), 130.5 (C-d^2), 130.3 (C-5), 126.1 (C-6), 122.8 (C-c^2), 92.4 (C-1´), 84.6 (d, $^3J_{CP}$= 9.7 Hz, C-4´), 76.9 (C-2´), 70.2 (d, $^3J_{CP}$= 5.6 Hz, C-a^2), 69.5 (d, $^3J_{CP}$= 6.2 Hz, C-a^1), 69.4 (d, $^3J_{CP}$= 3.9 Hz, C-3´), 65.0 (dd, $^3J_{CP}$= 5.1 Hz, $^3J_{CP}$= 2.3 Hz, C-5´), 34.7 (C-g^2), 33.3 (d, $^3J_{CP}$ = 7.3 Hz, C-b^1), 28.1 (C-h), 23.2 (C-i), 19.7 (C-c^1), 14.1 (C-j), 13.9 (C-d^1). ^{31}P NMR (243 MHz, CD_3OD): δ [ppm] = -11.69 (d, $^2J_{pp}$= 18.2 Hz, P-*γ*), -12.57 (d, $^2J_{pp}$= 17.5 Hz, P-*α*), -23.69 (d, $^2J_{pp}$= 17.7 Hz, P-*β*). HRMS (ESI$^-$, m/z): $C_{26}H_{38}N_3O_{17}P_3$, [M-H]$^-$ 756.1341; found 756.1349.

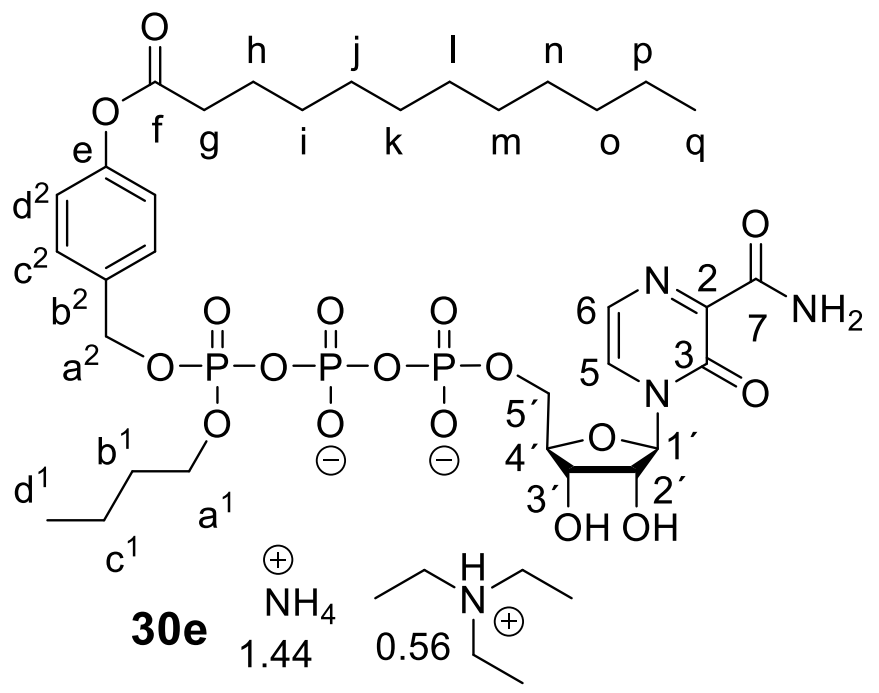

γ-(AB-C11;alkyl-C4)-T-1106-TP **30e**. According to general procedure with 128 mg (AB-C11;alkyl-C4)-*H*-phosphonate **18e** (0.3 mmol, 1.0 equiv.), 40 mg NCS (0.3 mmol, 1.0 equiv.), and 346 mg T-1106-DP 3×*n*Bu_4N^+ salt (0.3 mmol, 1.0 equiv.) and then stirred for 4 h at room temperature. Yield: 80 mg (0.09 mmol, 30%) white solid. Purity (HPLC): > 97%. ^{1}H NMR (400 MHz, CD_3OD): δ [ppm] = 8.47 (d, $^3J_{HH}$= 4.1 Hz, 1H, H-5), 7.82 (dd, $^3J_{HH}$= 4.2 Hz, $^4J_{HH}$= 1.4 Hz, 1H, H-6), 7.52-7.44 (m, 2H, H-c^2), 7.14-7.02 (m, 2H, H-d^2), 6.07 (d, $^4J_{HH}$= 1.9 Hz, 1H, H-1´), 5.19 (dd, $^3J_{HH}$= 8.0 Hz, $^4J_{HH}$= 1.9 Hz, 2H, H-a^2), 4.44-4.07 (m, 7H, H-2´, H-3´, H-4´, H-5´, H-a^1), 3.20 (t, $^3J_{HH}$= 7.5 Hz, 3.24H, $HN(CH_2CH_3)_3^+$), 2.57 (t, $^3J_{HH}$= 7.4 Hz, 2H, H-g), 1.75-1.67 (m, 2H, H-h), 1.66-1.56 (m, 2H, H-b^1), 1.46-1.26 (m, 22.8H, H-c^1, H-i, H-j, H-k, H-l, H-m, H-n, H-o, H-p, $HN(CH_2CH_3)_3^+$), 0.94-0.86 (m, 6H, H-d^1, H-q). ^{13}C-NMR (101 MHz, CD_3OD): δ [ppm] = 173.7 (C-f), 166.2 (C-7), 157.1 (C-3), 151.5 (C-e), 143.8 (C-2), 135.1 (C-b^2), 130.5, 130.3 (C-d^2, C-5), 126.1 (C-6), 122.8 (C-c^2), 92.3 (C-1´), 86.3 (C-4´), 76.9 (C-2´), 70.2 (C-a^2), 69.5 (C-a^1), 69.4 (C-3´), 65.0 (C-5´), 35.0 (C-g^2), 33.3 (d, $^3J_{CP}$ = 7.1 Hz, C-b^1), 33.1, 30.7, 30.6, 30.5, 30.4, 30.2, 23.7 (C-i, C-j, C-k, C-l, C-m, C-n, C-o, C-p), 25.9 (C-h), 19.7 (C-c^1), 14.4 (C-q), 13.9 (C-d^1). ^{31}P NMR (162 MHz, CD_3OD): δ [ppm] = -11.76 (d, $^2J_{pp}$= 20.2 Hz, P-*γ*), -13.10 (d, $^2J_{pp}$= 17.5 Hz, P-*α*), -23.69 (d, $^2J_{pp}$= 18.6 Hz, P-*β*). HRMS (ESI$^-$, m/z): $C_{33}H_{52}N_3O_{17}P_3$, [M-H]$^-$ 854.2437; found 854.2428.

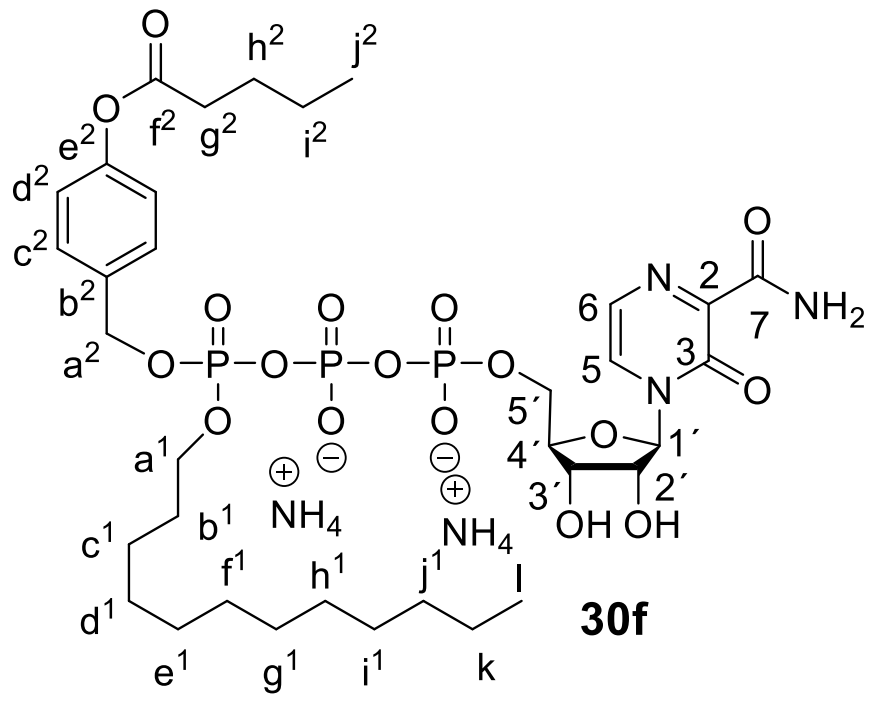

30f

γ-(AB-C4;alkyl-C12)-T-1106-TP **30f**. According to general procedure with 132 mg (AB-C4;alkyl-C12)-*H*-phosphonate **18f** (0.3 mmol, 1.0 equiv.), 40 mg NCS (0.3 mmol, 1.0 equiv.), and 346 mg T-1106-DP 3×nBu$_4$N$^+$ salt (0.3 mmol, 1.0 equiv.) and then stirred for 4 h at room temperature. Yield: 163 mg (0.18 mmol, 60%) white solid. Purity (HPLC): > 98%. ^{1}H NMR (600 MHz, CD$_3$OD): δ [ppm] = 8.45 (dd, $^3J_{HH}$= 4.1 Hz, $^4J_{HH}$= 1.4 Hz, 1H, H-5), 7.82 (t, $^3J_{HH}$= 4.0 Hz, 1H, H-6), 7.50-7.44 (m, 2H, H-c^2), 7.10-7.03 (m, 2H, H-d^2), 6.06 (s, 1H, H-1′), 5.23-5.18 (m, 2H, H-a^2), 4.44-4.16 (m, 5H, H-2′, H-3′, H-4′, H-5′), 4.13-4.07 (m, 2H, H-a^1), 2.60-2.55 (m, 2H, H-g^2), 1.75-1.68 (m, 2H, H-h^2), 1.65-1.58 (m, 2H, H-b^1), 1.48-1.40 (m, 2H, H-i^2), 1.35-1.25 (m, 18H, H-c^1, H-d^1, H-e^1, H-f^1, H-g^1, H-h^1, H-i^1, H-j^1, H-k), 0.98 (t, $^3J_{HH}$= 7.4 Hz, 2H, H-j^2), 0.90 (t, $^3J_{HH}$= 6.9 Hz, 3H, H-l). ^{13}C-NMR (151 MHz, CD$_3$OD): δ [ppm] = 173.7 (C-f^2), 166.3 (C-7), 157.1 (C-3), 152.3 (C-e^2), 143.7 (C-2), 135.1 (d, $^3J_{CP}$= 7.8 Hz, C-b^2), 130.7 (d, $^3J_{CP}$= 7.8 Hz, C-d^2), 130.3 (C-5), 126.1 (C-6), 122.8 (C-c^2), 92.3 (C-1′), 84.6 (C-4′), 76.9 (C-2′), 70.2 (d, $^3J_{CP}$= 4.7 Hz, C-a^2), 69.8 (d, $^3J_{CP}$= 6.2 Hz, C-a^1), 69.4 (d, $^3J_{CP}$= 3.6 Hz, C-3′), 64.9 (C-5′), 34.8 (C-g^2), 33.1, 30.81, 30.78, 30.73, 30.68, 30.5, 30.3, 23.7 (C-d^1, C-e^1, C-f^1, C-g^1, C-h^1, C-i^1, C-j^1, C-k), 31.2 (d, $^3J_{CP}$ = 6.9 Hz, C-b^1), 28.1 (C-h^2), 26.5 (C-c^1), 23.2 (C-i^2), 14.4 (C-l), 14.1 (C-j^2). ^{31}P NMR (243 MHz, CD$_3$OD): δ [ppm] = -11.64 (d, $^2J_{pp}$= 17.9 Hz, P-γ), -13.97 (d, $^2J_{pp}$= 17.5 Hz, P-α), -23.59 (d, $^2J_{pp}$= 17.6 Hz, P-β). HRMS (ESI$^-$, m/z): $C_{34}H_{54}N_3O_{17}P_3$, [M-H]$^-$ 868.2593; found 868.2592.

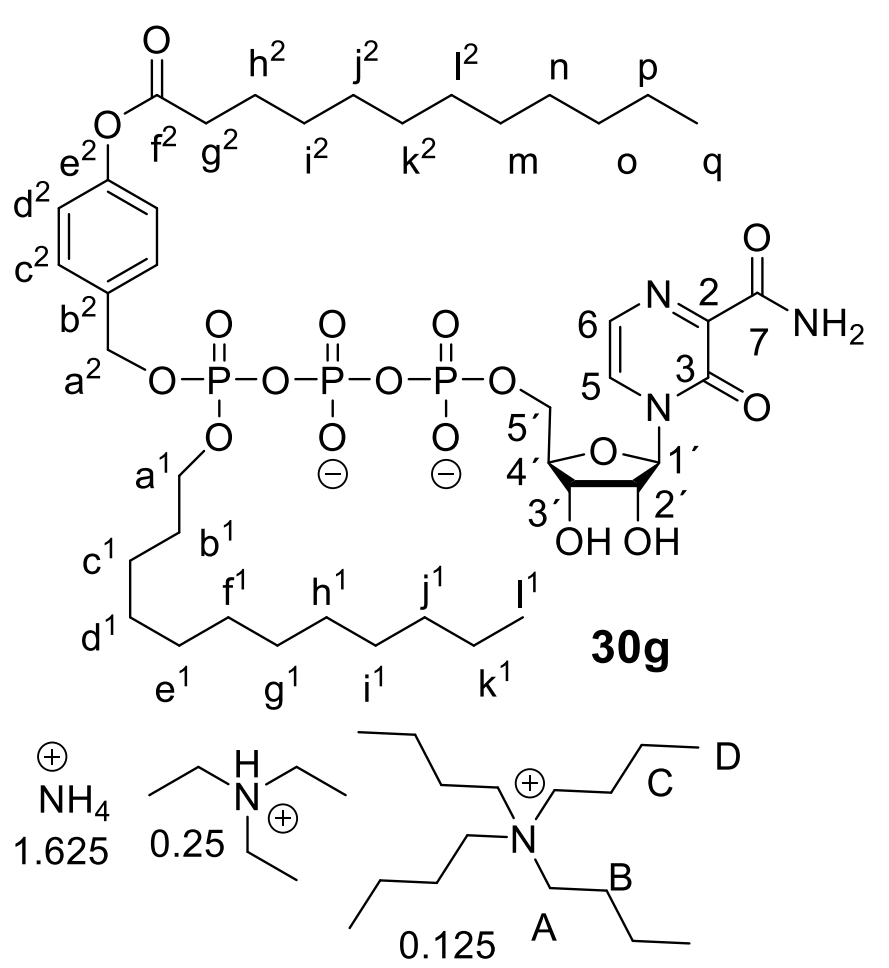

30g

γ-(AB-C11;alkyl-C12)-T-1106-TP **30g**. According to general procedure with 162 mg (AB-C11;alkyl-C12)-*H*-phosphonate **18g** (0.3 mmol, 1.0 equiv.), 40 mg NCS (0.3 mmol, 1.0 equiv.), and 346 mg T-1106-DP 3×nBu$_4$N$^+$ salt (0.3 mmol, 1.0 equiv.) and then stirred for 4 h at room temperature. Yield: 63 mg (0.06 mmol, 21%) white solid. Purity (HPLC): > 95%. ^{1}H NMR (600 MHz, CD$_3$OD): δ [ppm] = 8.46 (dd, $^3J_{HH}$= 4.1 Hz, $^4J_{HH}$= 1.8 Hz, 1H, H-5), 7.82 (d, $^3J_{HH}$= 4.1 Hz, 1H, H-6), 7.50-7.45 (m, 2H, H-c^2), 7.10-7.03 (m, 2H, H-d^2), 6.07 (d, $^4J_{HH}$= 1.9 Hz, 1H, H-1′), 5.23-5.16 (m, 2H, H-a^2), 4.44-4.14 (m, 5H, H-2′, H-3′, H-4′, H-5′), 4.13-4.06 (m, 2H, H-a^1), 3.25-3.31 (m, 0.8H, H-A), 3.19 (q, $^3J_{HH}$= 7.6 Hz, 1.2H, HN(CH$_2$CH$_3$)$_3^+$), 2.58-2.54 (m, 2H, H-g^2), 1.75-1.56 (m, 4.8H, H-h^2, H-B, H-b^1), 1.48-1.25 (m, 39.2H, H-C, H-c^1, H-d^1, H-e^1, H-f^1, H-g^1, H-h^1, H-i^1, H-i^2, H-j^1, H-j^2, H-k^1, H-k^2, H-l^1, H-l^2, H-m, H-n, H-o, H-p, HN(CH$_2$CH$_3$)$_3^+$), 1.02 (t, $^3J_{HH}$= 7.3 Hz, 1.5H,), 0.92-0.88 (m, 6H, H-l^1, H-q). ^{13}C-NMR (151 MHz, CD$_3$OD): δ [ppm] = 173.6 (C-f^2), 166.3 (C-7), 157.1 (C-3), 152.3 (C-e^2), 144.5 (C-2), 135.2 (C-b^2), 130.7 (d, $^3J_{CP}$= 7.8 Hz, C-d^2), 130.35 (C-5), 126.1 (C-6), 122.8 (C-c^2), 92.3 (C-1′), 84.6 (C-4′), 76.9 (C-2′), 70.2 (d, $^3J_{CP}$= 5.9 Hz, C-a^2), 69.8 (d, $^3J_{CP}$= 4.9 Hz, C-a^1), 69.6 (d, $^3J_{CP}$= 2.4 Hz, C-3′), 65.0 (C-5′), 35.0 (C-g^2), 33.10, 33.07, 30.79, 30.73, 30.68, 30.60, 30.51, 30.47, 30.40, 30.3, 30.2, 23.75, 23.73 (C-d^1, C-e^1, C-f^1, C-g^1, C-h^1, C-i^1, C-i^2, C-j^1, C-j^2, C-k^1, C-k^2, C-l^2, C-m, C-n, C-o, C-p), 31.2 (d, $^3J_{CP}$ = 7.1 Hz, C-b^1), 26.5 (C-c^1), 26.0 (C-h^2), 14.45, 14.44 (C-l^1, C-q). ^{31}P NMR (243 MHz, CD$_3$OD): δ [ppm] = -11.75

(d, $^2J_{pp}$= 19.3 Hz, P-γ), -13.05 (d, $^2J_{pp}$= 17.5 Hz, P-α), -23.79 (d, $^2J_{pp}$= 18.6 Hz, P-β). HRMS (ESI$^-$, m/z): $C_{41}H_{68}N_3O_{17}P_3$, [M-H]$^-$ 966.3689; found 966.3684.

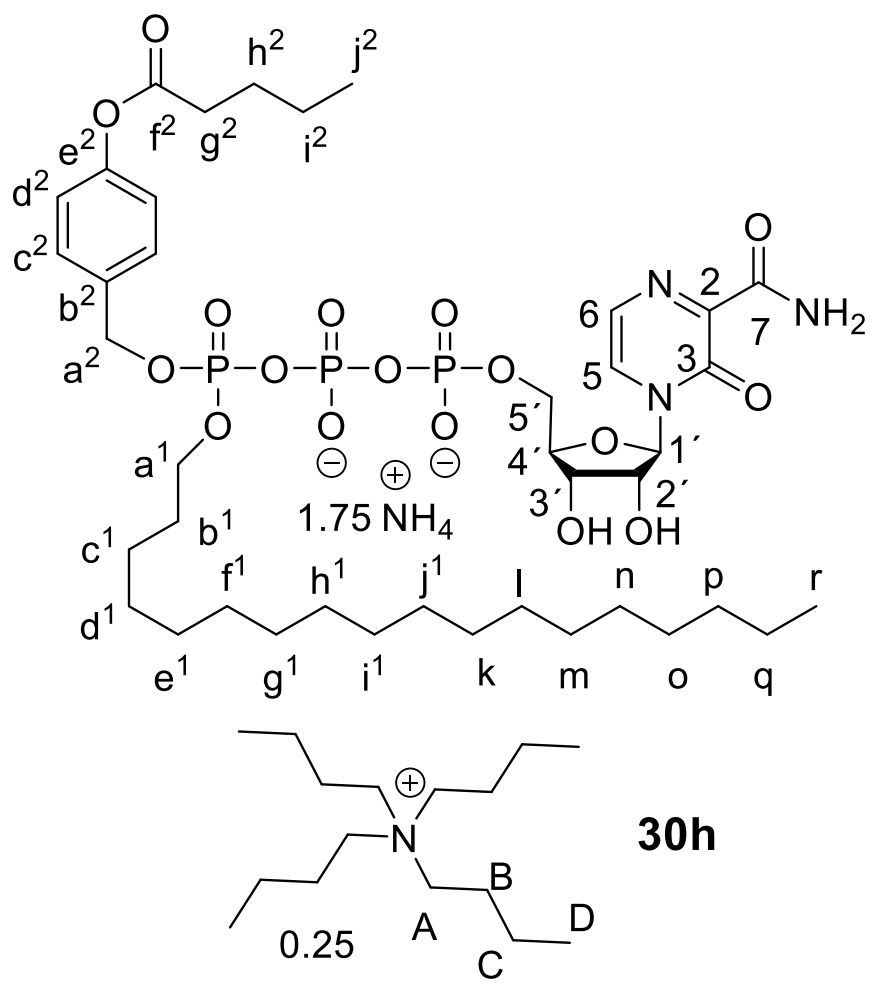

30h

γ-(AB-C4;alkyl-C18)-T-1106-TP **30h**. According to general procedure with 157 mg (AB-C4;alkyl-C18)-*H*-phosphonate **18h** (0.3 mmol, 1.0 equiv.), 40 mg NCS (0.3 mmol, 1.0 equiv.), and 346 mg T-1106-DP 3×nBu$_4$N$^+$ salt (0.3 mmol, 1.0 equiv.) and then stirred for 4 h at room temperature. Yield: 118 mg (0.12 mmol, 40%) white solid. Purity (HPLC): > 99%. ^{1}H NMR (400 MHz, CD_3OD): δ [ppm] = 8.36 (d, $^3J_{HH}$= 3.5 Hz, 1H, H-5), 7.78 (d, $^3J_{HH}$= 3.5 Hz, 1H, H-6), 7.52-7.45 (m, 2H, H-c^2), 7.12-7.05 (m, 2H, H-d^2), 6.08 (d, $^4J_{HH}$= 1.8 Hz, 1H, H-1´), 5.24-5.18 (m, 2H, H-a^2), 4.45-4.14 (m, 5H, H-2´, H-3´, H-4´, H-5´), 4.14-4.07 (m, 2H, H-a^1), 3.28-3.18 (m, 2H, H-A), 2.59 (t, $^3J_{HH}$= 7.4 Hz, 2H, H-g^2), 1.78-1.60 (m, 6H, H-b^1, H-B, H-h^2), 1.50-1.40 (m, 6H, H-C, H-c^1, H-i^2), 1.33-1.27 (m, 28H, H-d^1, H-e^1, H-f^1, H-g^1, H-h^1, H-i^1, H-j^1, H-k, H-l, H-m, H-n, H-o, H-p, H-q), 1.04 (t, $^3J_{HH}$= 7.4 Hz, 3H, H-D), 1.00 (t, $^3J_{HH}$= 7.4 Hz, 3H, H-j^2), 0.92 (t, $^3J_{HH}$= 6.7 Hz, 2H, H-r). ^{13}C-NMR (101 MHz, CD_3OD): δ [ppm] = 173.6 (C-f^2), 166.2 (C-7), 157.0 (C-3), 152.4 (C-e^2), 144.0 (C-2), 135.0 (d, $^3J_{CP}$= 7.3 Hz, C-b^2), 130.36, 130.34 (C-d^2, C-5), 125.9 (C-6), 122.9 (C-c^2), 92.4 (C-1´), 84.4 (d, $^3J_{CP}$= 9.9 Hz, C-4´), 76.7 (C-2´), 70.2 (d, $^3J_{CP}$= 5.2 Hz, C-a^2), 69.8 (d, $^3J_{CP}$= 6.2 Hz, C-a^1), 69.6 (d, $^3J_{CP}$= 3.2 Hz, C-3´), 65.5 (d, $^3J_{CP}$= 5.8 Hz, C-5´), 59.5 (t, $^3J_{CP}$= 2.8 Hz, C-A), 34.7 (C-g^2), 33.1, 30.79, 30.75, 30.72, 30.66, 30.5, 30.3, 23.7 (C-d^1, C-e^1, C-f^1, C-g^1, C-h^1, C-i^1, C-j^1, C-k, C-l, C-m, C-n, C-o, C-p, C-q), 31.2 (d, $^3J_{CP}$ = 7.2 H$_Z$, C-b^1), 28.1 (C-h^2), 26.5 (C-c^1), 24.8 (C-B), 23.2 (C-i^2), 20.7 (t, $^4J_{CP}$= 1.4 Hz, C-C), 14.4 (C-r), 14.1 (C-j^2), 13.9 (C-D). ^{31}P NMR (162 MHz, CD_3OD): δ [ppm] = -11.23 (d, $^2J_{pp}$=20.5 Hz, P-γ), -12.17 (d, $^2J_{pp}$=20.5 Hz, P-α), -23.71 (t, $^2J_{pp}$= 19.5 Hz, P-β). HRMS (ESI$^-$, m/z): $C_{40}H_{65}N_3O_{17}P_3$, [M-H]$^-$ 952.3532; found 952.3485.

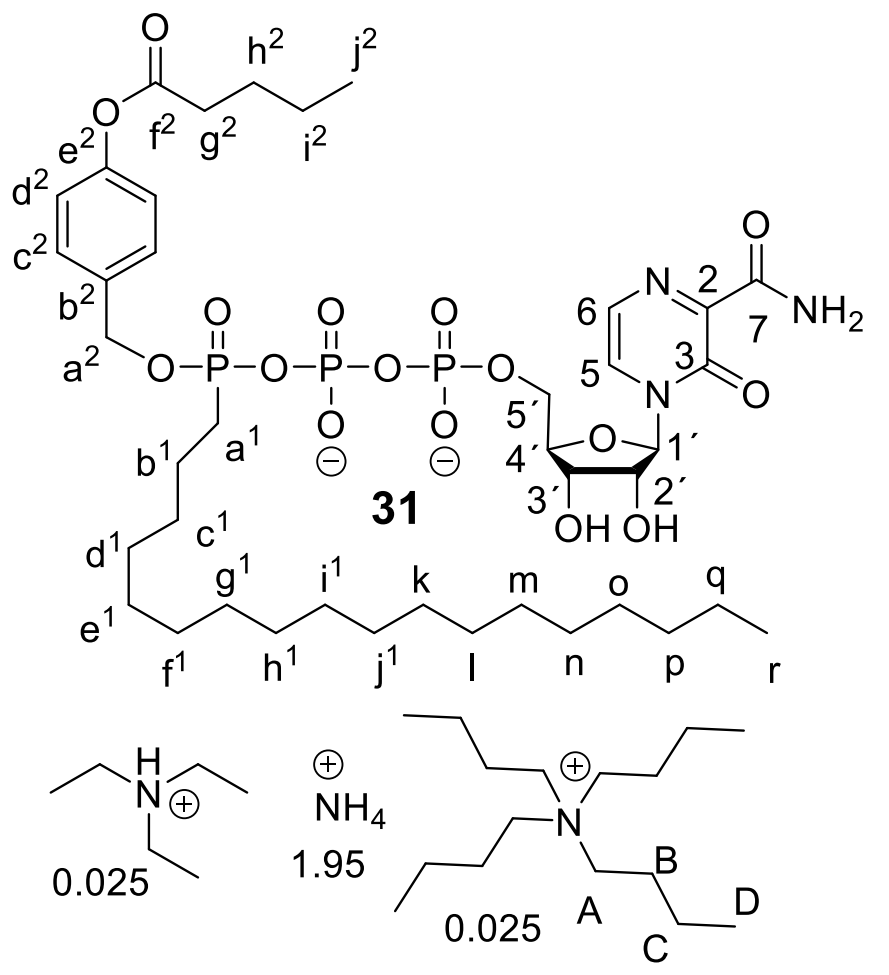

31

γ-(AB-C4)-γ-(alkyl-C18)-T-1106-TP **31**. According to general procedure with 153 mg (AB-C4;alkyl-C18)-*H*-phosphinate **19** (0.3 mmol, 1.0 equiv.), 40 mg NCS (0.3 mmol, 1.0 equiv.), and 346 mg T-1106-DP 3×nBu$_4$N$^+$ salt (0.3 mmol, 1.0 equiv.) and then stirred for 4 h at room temperature. Yield: 157 mg (0.16 mmol, 54%) white solid. Purity (HPLC): > 99%. ^{1}H NMR (600 MHz, CD_3OD): δ [ppm] = 8.45 (d, $^3J_{HH}$= 4.1 Hz, 1H, H-5), 7.80 (d, $^3J_{HH}$= 4.1 Hz, 1H, H-6), 7.50-7.44 (m, 2H, H-c^2), 7.08-7.03 (m, 2H, H-d^2), 6.07-6.05 (m, 1H, H-1´), 5.25-5.15 (m, 2H, H-a^2), 4.45-4.17 (m, 5H, H-2´, H-3´, H-4´, H-5´), 3.26-3.22 (m, 0.2H, H-A), 3.19 (q, $^3J_{HH}$= 7.6 Hz, 0.15H, $HN(CH_2CH_3)_3^+$), 2.57 (t, $^3J_{HH}$= 7.4 Hz, 2H, H-g^2), 2.05-1.94 (m, 2H, H-a^1), 1.75-1.67 (m, 2H, H-h^2), 1.67-1.55 (m, 2.4H, H-b^1, H-B), 1.48-1.40 (m, 2.4H, H-C, H-i^2), 1.32-1.25 (m, 30H, H-c^1, H-d^1, H-e^1, H-f^1, H-g^1, H-h^1, H-i^1, H-j^1, H-k, H-l, H-m, H-n, H-o, H-p, H-q), 1.02 (t, $^3J_{HH}$= 7.3 Hz, 0.3H, H-D), 0.98 (t, $^3J_{HH}$=

7.4 Hz, 3H, H-j[2]), 0.90 (t, $^3J_{HH}$= 7.0 Hz, 3H, H-r). ^{13}C-NMR (151 MHz, CD_3OD): δ [ppm] = 173.7 (C-f[2]), 166.3 (C-7), 157.0 (C-3), 152.2 (C-e[2]), 143.7 (C-2), 135.5 (d, $^3J_{CP}$= 6.9 Hz, C-b[2]), 130.7 (C-d[2]), 130.3 (d, $^3J_{CP}$= 2.5 Hz, C-5), 126.0 (C-6), 122.8 (C-c[2]), 92.4 (C-1′), 84.5 (d, $^3J_{CP}$= 9.5 Hz, C-4′), 76.8 (C-2′), 69.4 (C-3′), 67.9 (t, $^3J_{CP}$= 6.1 Hz, C-a[2]), 65.0 (C-5′), 43.2 ($HN(CH_2CH_3)_3^+$), 34.7 (C-g[2]), 33.1, 31.57, 31.46, 30.80, 30.75, 30.73, 30.55, 30.47, 30.2, 23.7 (C-c[1], C-d[1], C-e[1], C-f[1], C-g[1], C-h[1], C-i[1], C-j[1], C-k, C-l, C-m, C-n, C-o, C-p, C-q), 28.1 (C-h[2]), 27.5, 26.6 (C-a[1]), 24.8 (C-B), 23.3 (C-b[1]), 23.2 (C-i[2]), 20.7 (C-C), 14.4 (C-r), 14.1 (C-j[2]), 13.9 (C-D), 11.6 ($HN(CH_2CH_3)_3^+$). ^{31}P NMR (243 MHz, CD_3OD): δ [ppm] = 24.46 (d, $^2J_{pp}$= 23.5, P-γ), -11.61 (d, $^2J_{pp}$= 17.6 Hz, P-α), -23.50 (dd, $^2J_{pp}$= 23.5 Hz, $^3J_{pp}$= 17.6 Hz, P-β). HRMS (ESI$^-$, m/z): $C_{40}H_{66}N_3O_{16}P_3$, $[M-H]^-$ 936.3583; found 936.3582.

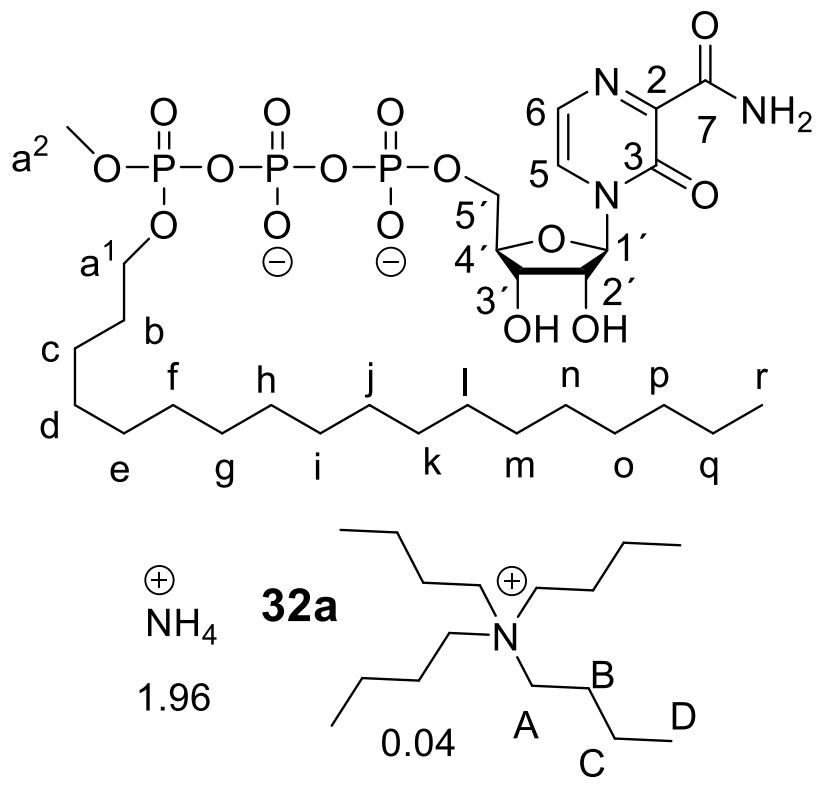

γ-(Alkyl-C1;alkyl-C18)-T-1106-TP **32a**. According to general procedure with 105 mg (alkyl-C1;alkyl-C18)-*H*-phosphonate **20a** (0.3 mmol, 1.0 equiv.), 40 mg NCS (0.3 mmol, 1.0 equiv.), and 346 mg T-1106-DP 3×nBu$_4$N$^+$ salt (0.3 mmol, 1.0 equiv.) and then stirred for 4 h at room temperature. Yield: 49 mg (0.06 mmol, 20%) white solid. Purity (HPLC): > 96%. ^{1}H NMR (600 MHz, CD_3OD): δ [ppm] = 8.47 (d, $^3J_{HH}$= 4.2 Hz, 1H, H-5), 7.79 (dd, $^3J_{HH}$= 4.2 Hz, $^4J_{HH}$= 1.0 Hz, 1H, H-6), 6.09 (d, $^3J_{HH}$= 2.1 Hz, 1H, H-1′), 4.45-4.21 (m, 5H, H-2′, H-3′, H-4′, H-5′), 4.18-4.15 (m, 2H, H-a[1]), 3.85 (dd, $^3J_{HH}$=11.6 Hz, $^3J_{HH}$=3.1 Hz, 3H, H-a[2]), 3.26-3.22 (m, 0.32H, H-A), 1.73-1.66 (m, 2.32H, H-b, H-B), 1.43-1.36 (m, 2.32H, H-c, H-C), 1.35-1.26 (m, 28H, H-d, H-e, H-f, H-g, H-h, H-i, H-j, H-k, H-l, H-m, H-n, H-o, H-p, H-q), 1.03 (t, $^3J_{HH}$= 7.4 Hz, 0.48H, H-D), 0.90 (t, $^3J_{HH}$= 7.0 Hz, 3H, H-r). ^{13}C-NMR (151 MHz, CD_3OD): δ [ppm] = 166.4 (C-7), 157.1 (C-3), 143.8 (C-2), 130.8 (C-5), 126.1 (C-6), 92.4 (C-1′), 84.6 (d, $^3J_{CP}$= 9.7 Hz, C-4′), 76.9 (C-2′), 69.8 (d, $^3J_{CP}$= 6.0 Hz, C-a[1]), 69.4 (C-3′), 65.0 (d, $^3J_{CP}$= 6.2 Hz, C-5′), 55.5 (d, $^3J_{CP}$= 6.1 Hz, C-a[2]), 33.1, 30.79, 30.75, 30.73, 30.69, 30.5, 30.3, 23.7 (C-d, C-e, C-f, C-g, C-h, C-i, C-j, C-k, C-l, C-m, C-n, C-o, C-p, C-q), 31.3 (d, $^3J_{CP}$ = 7.4 H$_Z$, C-b), 26.6 (C-c), 14.4 (C-r). ^{31}P NMR (243 MHz, CD_3OD): δ [ppm] = -12.0 -- -11.5 (m, 2P, P-α, P-γ), -23.81 (t, $^2J_{pp}$= 17.6 Hz, P-β). HRMS (ESI$^-$, m/z): $C_{29}H_{54}N_3O_{15}P_3$, $[M-H]^-$ 776.2695; found 776.2692.

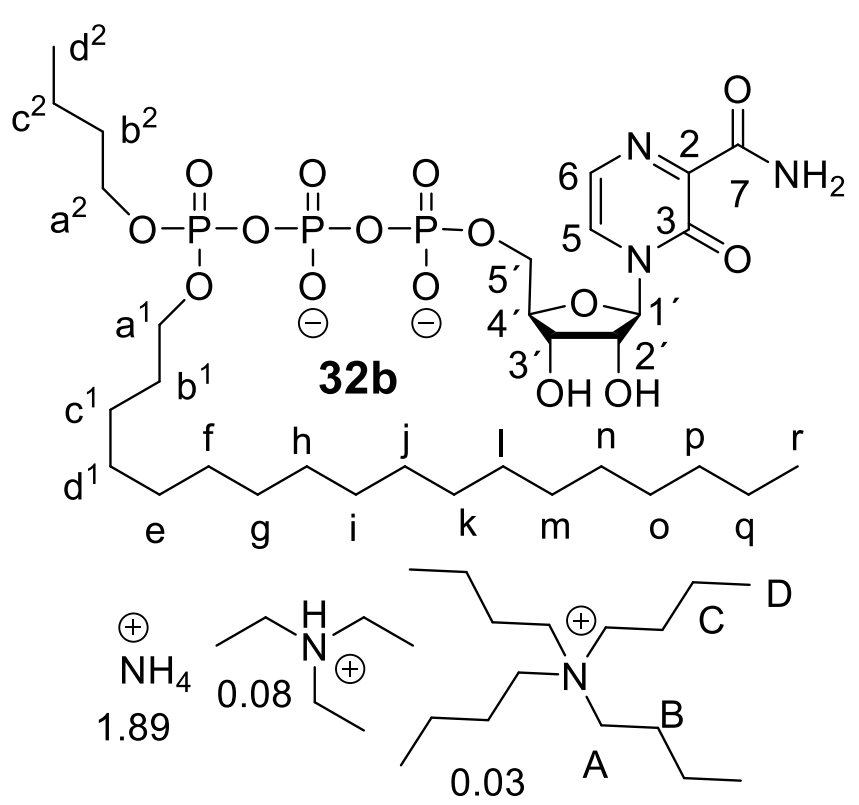

γ-(Alkyl-C4;alkyl-C18)-T-1106-TP **32b**. According to general procedure with 117 mg (alkyl-C4;alkyl-C18)-*H*-phosphonate **20b** (0.3 mmol, 1.0 equiv.), 40 mg NCS (0.3 mmol, 1.0 equiv.), and 346 mg T-1106-DP 3×nBu$_4$N$^+$ salt (0.3 mmol, 1.0 equiv.) and then stirred for 4 h at room temperature. Yield: 123 mg (0.14 mmol, 48%) white solid. Purity (HPLC): > 99%. ^{1}H NMR (600 MHz, CD_3OD): δ [ppm] = 8.49 (d, $^3J_{HH}$= 4.1 Hz, 1H, H-5), 7.84 (d, $^3J_{HH}$= 4.1 Hz, 1H, H-6), 6.08 (d, $^3J_{HH}$= 2.1 Hz, 1H, H-1′), 4.45-4.20 (m, 5H, H-2′, H-3′, H-4′, H-5′), 4.20-4.10 (m, 4H, H-a[1], H-a[2]), 3.26-3.22 (m, 0.72H, H-A, $HN(CH_2CH_3)_3^+$), 1.73-1.62 (m, 4.24H, H-b[1], H-b[2], H-B), 1.46-1.36 (m, 4.24H, H-c[1], H-c[2], H-C), 1.35-1.20 (m, 28.72H, H-d[1], H-e, H-f, H-g, H-h, H-i, H-j, H-k, H-l, H-m, H-n, H-o, H-p, H-q, $HN(CH_2CH_3)_3^+$), 1.03 (t, $^3J_{HH}$= 7.3 Hz, 0.36H, H-D), 0.94 (dt, $^3J_{HH}$= 7.4

Hz, $^{3}J_{HH}$= 2.7 Hz, 3H, H-d^{1}), 0.90 (t, $^{3}J_{HH}$= 6.9 Hz, 3H, H-r). ^{13}C-NMR (151 MHz, CD_3OD): δ [ppm] = 166.3 (C-7), 157.1 (C-3), 143.8 (C-2), 130.8 (C-5), 126.1 (C-6), 92.3 (C-1´), 84.6 (d, $^{3}J_{CP}$= 9.7 Hz, C-4´), 76.9 (C-2´), 69.72, 69.67 (C-a^{1}, C-a^{2}), 69.3 (d, $^{3}J_{CP}$= 6.7 Hz, C-3´), 64.9 (d, $^{3}J_{CP}$= 4.5 Hz, C-5´), 33.3 (d, $^{3}J_{CP}$= 7.6 Hz, C-b^{2}), 33.1, 30.79, 30.76, 30.73, 30.71, 30.5, 30.3, 23.7 (C-d, C-e, C-f, C-g, C-h, C-i, C-j, C-k, C-l, C-m, C-n, C-o, C-p, C-q), 31.3 (d, $^{3}J_{CP}$ = 7.3 Hz, C-b^{1}), 26.6 (C-c^{1}), 19.8 (C-c^{2}), 14.4 (C-r), 14.0 (C-d^{1}). ^{31}P NMR (243 MHz, CD_3OD): δ [ppm] = -11.65 (d, $^{2}J_{pp}$= 20.5 Hz, P-α), -12.76 (d, $^{2}J_{pp}$= 17.7, P-γ), -23.57 (d, $^{3}J_{pp}$= 17.7 Hz, P-β). HRMS (ESI$^-$, m/z): $C_{32}H_{60}N_3O_{15}P_3$, [M-H]$^-$ 818.3165; found 818.2932.

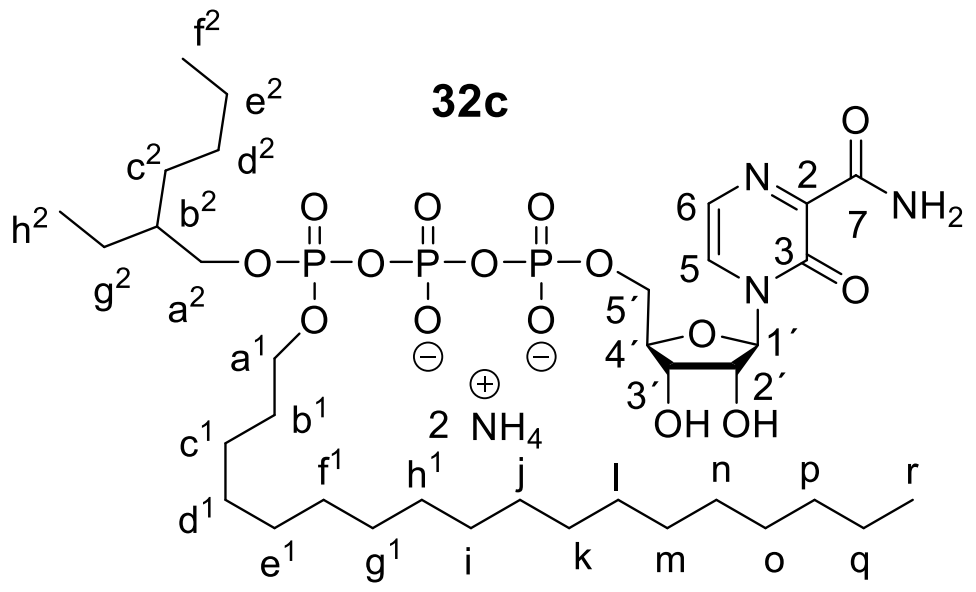

γ-(Alkyl-C8;alkyl-C18)-T-1106-TP **32c**. According to general procedure with 134 mg (alkyl-C8;alkyl-C18)-*H*-phosphonate **20c** (0.3 mmol, 1.0 equiv.), 40 mg NCS (0.3 mmol, 1.0 equiv.), and 346 mg T-1106-DP 3×*n*Bu_4N^+ salt (0.3 mmol, 1.0 equiv.) and then stirred for 4 h at room temperature. Yield: 76 mg (0.08 mmol, 28%) white solid. Purity (HPLC): > 96%. ^{1}H NMR (400 MHz, CD_3OD): δ [ppm] = 8.40 (d, $^{3}J_{HH}$= 4.1 Hz, 1H, H-5), 7.88 (d, $^{3}J_{HH}$= 4.1 Hz, 1H, H-6), 6.10-6.06 (m, 1H, H-1´), 4.45-4.18 (m, 5H, H-2´, H-3´, H-4´, H-5´), 4.18-4.05 (m, 4H, H-a^{1}, H-a^{2}), 1.73-1.66 (m, 2H, H-b^{1}), 1.65-1.56 (m, 1H, H-b^{2}), 1.43-1.26 (m, 38H, H-c^{1}, H-c^{2}, H-d^{1}, H-d^{2}, H-e^{1}, H-e^{2}, H-f^{1}, H-g^{1}, H-g^{2}, H-h^{1}, H-i, H-j, H-k, H-l, H-m, H-n, H-o, H-p, H-q), 0.95-0.88 (m, 9H, H-h^{2}, H-f^{2}, H-r). ^{13}C-NMR (101 MHz, CD_3OD): δ [ppm] = 166.3 (C-7), 157.1 (C-3), 142.1 (C-2), 130.5 (C-5), 125.9 (C-6), 92.3 (C-1´), 84.4 (C-4´), 76.8 (C-2´), 71.1 (C-a^{2}), 69.8 (C-a^{1}), 69.7 (d, $^{3}J_{CP}$= 5.9 Hz, C-3´), 65.5 (C-5´), 59.5 (C-A), 33.3 (d, $^{3}J_{CP}$= 7.6 Hz, C-b^{2}), 33.1, 30.8, 30.71, 30.68, 30.5, 30.3, 23.7 (C-c^{2}, C-d^{1}, C-d^{2}, C-e^{1}, C-e^{2}, C-f^{1}, C-g^{1}, C-g^{2}, C-h^{1}, C-i, C-j, C-k, C-l, C-m, C-n, C-o, C-p, C-q), 31.3 (d, $^{3}J_{CP}$ = 6.4 Hz, C-b^{1}), 26.6 (C-c^{1}), 24.8 (C-B), 20.7 (C-C), 14.4 (C-r), 13.95 (C-f^{2}, C-D), 9.2 (C-h^{2}). ^{31}P NMR (162 MHz, CD_3OD): δ [ppm] = -13.16 (d, $^{2}J_{pp}$= 17.7 Hz, P-α), -14.12 (d, $^{2}J_{pp}$= 15.7, P-γ), -25.08 (d, $^{3}J_{pp}$= 16.7 Hz, P-β). HRMS (ESI$^-$, m/z): $C_{36}H_{68}N_3O_{15}P_3$, [M-H]$^-$ 874.3791; found 874.3784.

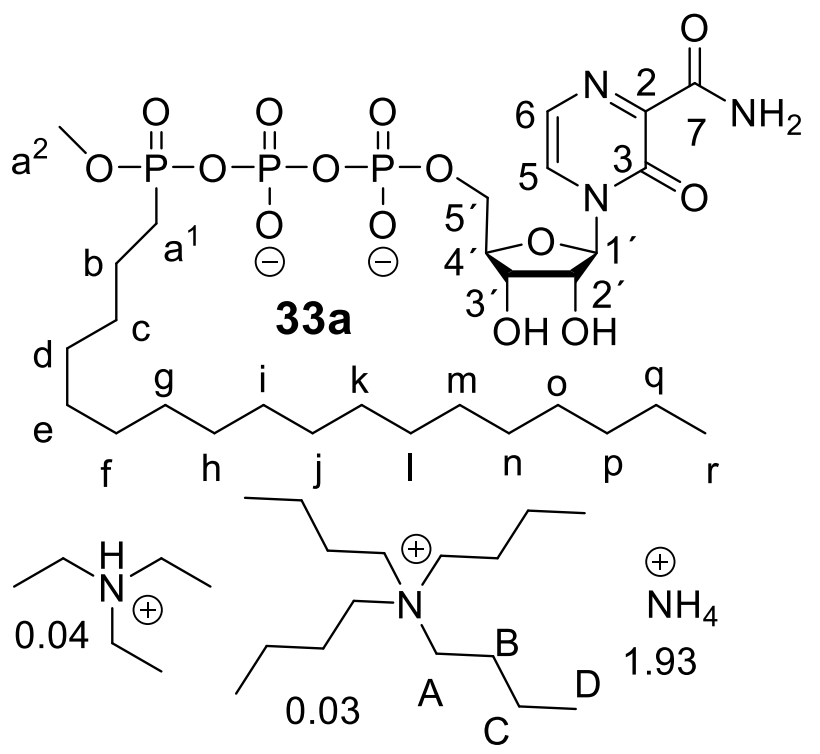

γ-(Alkyl-C1)-γ-(alkyl-C18)-T-1106-TP**33a**. According to general procedure with 100 mg (alkyl-C1;alkyl-C18)-*H*-phosphinate **21a** (0.3 mmol, 1.0 equiv.), 40 mg NCS (0.3 mmol, 1.0 equiv.), and 346 mg T-1106-DP 3×*n*Bu_4N^+ salt (0.3 mmol, 1.0 equiv.) and then stirred for 4 h at room temperature. Yield: 111 mg (0.14 mmol, 46%) white solid. Purity (HPLC): > 96%. ^{1}H NMR (600 MHz, CD_3OD): δ [ppm] = 8.48 (d, $^{3}J_{HH}$= 4.1 Hz, 1H, H-5), 7.83 (d, $^{3}J_{HH}$= 4.1 Hz, 1H, H-6), 6.08 (d, $^{3}J_{HH}$= 2.0 Hz, 1H, H-1´), 4.45-4.18 (m, 5H, H-2´, H-3´, H-4´, H-5´), 3.83 (dd, $^{2}J_{HH}$=11.3 Hz, $^{3}J_{HH}$=3.7 Hz, 3H, H-a^{2}), 3.28-3.15 (m, 0.48H, H-A, $HN(CH_2CH_3)_3^+$), 2.05-1.98 (m, 2H, H-a^{1}), 1.68-1.60 (m, 2.24H, H-b, H-B), 1.45-1.36 (m, 2.24H, H-c, H-C), 1.35-1.26 (m, 28.4H, H-d, H-e, H-f, H-g, H-h, H-i, H-j, H-k, H-l, H-m, H-n, H-o, H-p, H-q, $HN(CH_2CH_3)_3^+$), 1.02 (t, $^{3}J_{HH}$= 7.3 Hz, 0.36H, H-D), 0.90 (t, $^{3}J_{HH}$= 6.9 Hz, 3H, H-r). ^{13}C-NMR (151 MHz, CD_3OD): δ [ppm] = 166.4 (C-7), 157.1 (C-3), 143.8 (C-2), 130.8 (C-5), 126.1 (C-6), 92.3 (C-1´), 84.6 (d, $^{3}J_{CP}$= 9.0 Hz, C-4´), 76.9 (C-2´), 69.4 (C-3´), 64.9 (d, $^{3}J_{CP}$= 6.3 Hz, C-5´), 53.2 (d, $^{3}J_{CP}$= 6.5 Hz,

C-a^2), 33.1, 31.70, 31.59, 30.79, 30.75, 30.6, 30.5, 30.3, 23.7 (C-d, C-e, C-f, C-g, C-h, C-i, C-j, C-k, C-l, C-m, C-n, C-o, C-p, C-q), 27.0, 26.1 (C-a^1), 23.2 (d, $^3J_{CP}$ = 5.1 Hz, C-b), 14.4 (C-r). ^{31}P NMR (243 MHz, CD$_3$OD): δ [ppm] = 25.55 (d, $^2J_{pp}$= 25.5, $^3J_{pp}$= 3.9, P-γ), -10.71 (d, $^2J_{pp}$= 19.6 Hz, P-α), -22.58 (dd, $^2J_{pp}$= 25.5, $^2J_{pp}$= 19.6 Hz, P-β). HRMS (ESI$^-$, m/z): $C_{29}H_{54}N_3O_{14}P_3$, [M-H]$^-$ 760.2746; found 760.2766.

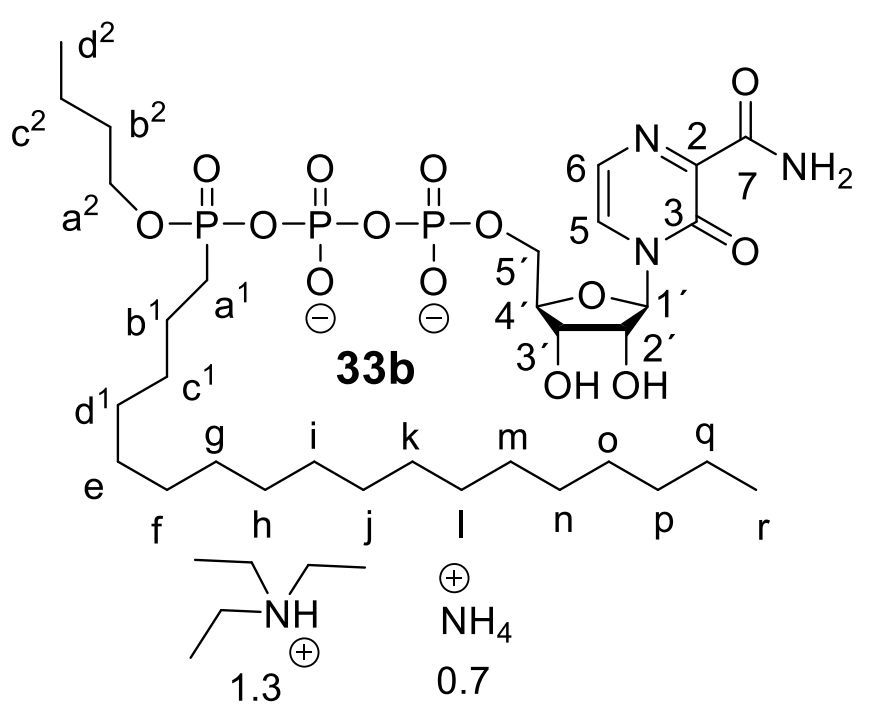

γ-(Alkyl-C4)-γ-(alkyl-C18)-T-1106-TP **33b**. According to general procedure with 112 mg (alkyl-C4;alkyl-C18)-*H*-phosphinate **21b** (0.3 mmol, 1.0 equiv.), 40 mg NCS (0.3 mmol, 1.0 equiv.), and 346 mg T-1106-DP 3×nBu$_4$N$^+$ salt (0.3 mmol, 1.0 equiv.) and then stirred for 4 h at room temperature. Yield: 199 mg (0.21 mmol, 70%) white solid. Purity (HPLC): > 99%. ^{1}H NMR (600 MHz, CD$_3$OD): δ [ppm] = 8.43 (d, $^3J_{HH}$= 4.2 Hz, 1H, H-5), 7.82 (d, $^3J_{HH}$= 4.2 Hz, 1H, H-6), 6.08 (d, $^3J_{HH}$= 2.0 Hz, 1H, H-1´), 4.45-4.10 (m, 7H, H-2´, H-3´, H-4´, H-5´, H-a^2), 3.21 (q, $^3J_{HH}$= 7.3 Hz, 7.8H, HN(CH$_2$CH$_3$)$_3^+$), 2.05-1.98 (m, 2H, H-a^1), 1.70-1.60 (m, 4H, H-b^1, H-b^2), 1.45-1.36 (m, 4H, H-c^1, H-c^2), 1.35-1.26 (m, 40H, H-d^1, H-e, H-f, H-g, H-h, H-i, H-j, H-k, H-l, H-m, H-n, H-o, H-p, H-q, HN(CH$_2$CH$_3$)$_3^+$), 0.94 (dt, $^3J_{HH}$= 7.6 Hz, $^2J_{HH}$= 3.4 Hz, 3H, H-d^2), 0.90 (t, $^3J_{HH}$= 7.0 Hz, 3H, H-r). ^{13}C-NMR (151 MHz, CD$_3$OD): δ [ppm] = 166.3 (C-7), 157.0 (C-3), 143.9 (C-2), 130.6 (C-5), 125.9 (C-6), 92.4 (C-1´), 84.4 (d, $^3J_{CP}$= 9.1 Hz, C-4´), 76.7 (C-2´), 69.4 (C-3´), 67.0 (d, $^3J_{CP}$= 7.4 Hz, C-a^2), 65.4 (d, $^3J_{CP}$= 5.4 Hz, C-5´), 47.6 (HN(CH$_2$CH$_3$)$_3^+$), 33.5 (d, $^3J_{CP}$= 6.9 Hz, C-b^2), 33.1, 31.62, 31.51, 30.78, 30.75, 30.72, 30.57, 30.46, 30.3, 23.7 (C-c^1, C-d^1, C-e, C-f, C-g, C-h, C-i, C-j, C-k, C-l, C-m, C-n, C-o, C-p, C-q), 27.4, 26.4 (C-a^1), 23.3 (d, $^3J_{CP}$ = 5.1 Hz, C-b^1), 19.8 (C-c^2), 14.4 (C-r), 14.0 (C-d^2), 9.2 (HN(CH$_2$CH$_3$)$_3^+$). ^{31}P NMR (243 MHz, CD$_3$OD): δ [ppm] = 24.07 (d, $^2J_{pp}$= 23.5, $^3J_{pp}$= 3.9, P-γ), -12.18 (d, $^2J_{pp}$= 17.6 Hz, P-α), -24.42 (t, $^2J_{pp}$= 20.6, P-β). HRMS (ESI$^-$, m/z): $C_{32}H_{60}N_3O_{14}P_3$, [M-H]$^-$ 802.3215; found 802.3262.

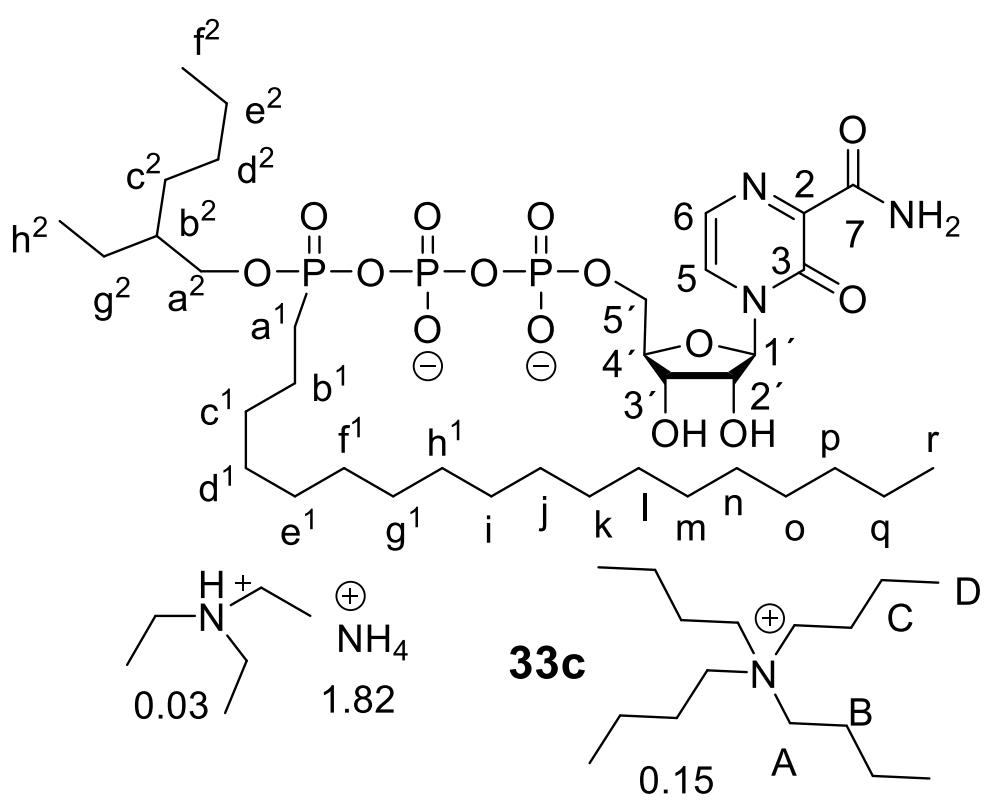

γ-(Alkyl-C8)-γ-(alkyl-C18)-T-1106-TP **33c**. According to general procedure with 129 mg (alkyl-C8;alkyl-C18)-*H*-phosphinate **21c** (0.3 mmol, 1.0 equiv.), 40 mg NCS (0.3 mmol, 1.0 equiv.), and 346 mg T-1106-DP 3×nBu$_4$N$^+$ salt (0.3 mmol, 1.0 equiv.) and then stirred for 4 h at room temperature. Yield: 159 mg (0.17 mmol, 57%) white solid. Purity (HPLC): > 98%. ^{1}H NMR (600 MHz, CD$_3$OD): δ [ppm] = 8.46 (d, $^3J_{HH}$= 4.2 Hz, 1H, H-5), 7.82 (dd, $^3J_{HH}$= 4.2 Hz, $^4J_{HH}$= 1.2 Hz, 1H, H-6), 6.07 (d, $^3J_{HH}$= 2.0 Hz, 1H, H-1´), 4.45-4.10 (m, 7H, H-2´, H-3´, H-4´, H-5´, H-a^2), 3.28-3.20 (m, 1.38H, H-A, HN(CH$_2$CH$_3$)$_3^+$), 2.05-1.94 (m, 2H, H-a^1), 1.70-1.62 (m, 3.2 H, H-b^1, H-B), 1.57-1.52 (m, 1H, H-b^2), 1.45-1.26 (m, 40.4H, H-C, H-c^1, H-c^2, H-d^1, H-d^2, H-e^1, H-e^2, H-f^1, H-g^1, H-g^2, H-h^1, H-i, H-j, H-k, H-l, H-m, H-n, H-o, H-p, H-q, HN(CH$_2$CH$_3$)$_3^+$), 1.02 (t, $^3J_{HH}$= 7.2 Hz, 1.8H, H-D), 0.92-0.87 (m, 9H, H-h^2, H-f^2, H-r). ^{13}C-NMR (151 MHz, CD$_3$OD): δ [ppm] = 166.3 (C-7), 157.1 (C-3), 143.8 (C-2), 130.7 (C-5), 126.0 (C-6), 92.4 (C-1´), 84.5 (d, $^3J_{CP}$= 9.6 Hz, C-4´), 76.9 (C-2´), 69.4 (C-3´), 69.1 (dd, $^2J_{CP}$= 16.4 Hz, $^3J_{CP}$= 7.9 Hz, C-a^2), 65.0 (d, $^3J_{CP}$= 5.2 Hz, C-5´), 59.5 (t, $^3J_{CP}$= 2.8 Hz, C-A), 47.6, 43.2 (HN(CH$_2$CH$_3$)$_3^+$),

41.4 (d, $^3J_{CP}$= 7.4 Hz, C-b^2), 33.1, 31.6, 31.5, 31.15, 31.14, 31.11, 30.77, 30.75, 30.72, 30.52, 30.46, 30.3, 30.1, 30.00, 29.99, 24.45, 24.41, 24.35, 24.1, 23.7 (C-c^1, C-c^2, C-d^1, C-d^2, C-e^1, C-e^2, C-f^1, C-g^1, C-g^2, C-h^1, C-i, C-j, C-k, C-l, C-m, C-n, C-o, C-p, C-q), 27.3, 26.4 (C-a^1), 24.8 (C-B), 23.4 (d, $^3J_{CP}$ = 5.6 Hz, C-b^1), 20.7 (C-C), 14.4 (C-r, C-f^2), 13.9 (C-D), 11.4 (d, $^3J_{CP}$= 5.7 Hz, C-h^2), 9.2 ($HN(CH_2CH_3)_3^+$). ^{31}P NMR (243 MHz, CD_3OD): δ [ppm] = 24.17 (dd, $^2J_{pp}$= 23.3, $^3J_{pp}$= 5.8, P-*γ*), -11.74 (d, $^2J_{pp}$= 17.8 Hz, P-*α*), -23.62 (t, $^2J_{pp}$= 20.7, P-*β*). HRMS (ESI$^-$, m/z): $C_{36}H_{68}N_3O_{14}P_3$, [M-H]$^-$ 858.3841; found 858.3822.

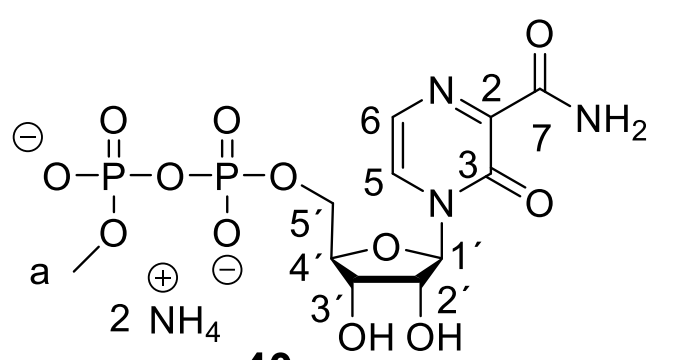

β-(Alkyl-C1)-T-1106-DP **40a**. According to general procedure with 82 mg (Fm;alkyl-C1)-*H*-phosphonate **36a** (0.3 mmol, 1.0 equiv.), 40 mg NCS (0.3 mmol, 1.0 equiv.), and 250 mg T-1106-MP 2×*n*Bu_4N^+ salt (0.3 mmol, 1.0 equiv.) and then stirred for 4 h at room temperature. Subsequently, 0.42 mL TEA (3.0 mmol, 10.0 equiv.) was added and stirred for 8 h. Yield: 48 mg (0.1 mmol, 33%) white solid. Purity (HPLC): > 98%. ^{1}H NMR (600 MHz, CD_3OD): δ [ppm] = 8.48 (d, $^3J_{HH}$= 4.2 Hz, 1H, H-5), 7.82 (d, $^3J_{HH}$= 4.2 Hz, 1H, H-6), 6.08 (d, $^3J_{HH}$= 2.0 Hz, 1H, H-1´), 4.45-4.18 (m, 5H, H-2´, H-3´, H-4´, H-5´), 3.67 (d, $^2J_{HH}$= 11.4 Hz, 2H, H-a). ^{13}C-NMR (151 MHz, CD_3OD): δ [ppm] = 166.4 (C-7), 157.1 (C-3), 143.8 (C-2), 130.8 (C-5), 126.0 (C-6), 92.4 (C-1´), 84.6 (d, $^3J_{CP}$= 9.6 Hz, C-4´), 76.9 (C-2´), 69.4 (C-3´), 64.7 (C-5´), 53.66, 53.62 (C-a, C-4´). ^{31}P NMR (243 MHz, CD_3OD): δ [ppm] = -9.60 (d, $^2J_{pp}$= 21.7 Hz, P-*β*), -11.46 (d, $^2J_{pp}$= 21.7 Hz, P-*α*). HRMS (ESI$^-$, m/z): $C_{11}H_{17}N_3O_{12}P_2$, [M-H]$^-$ 444.0215; found 444.0240.

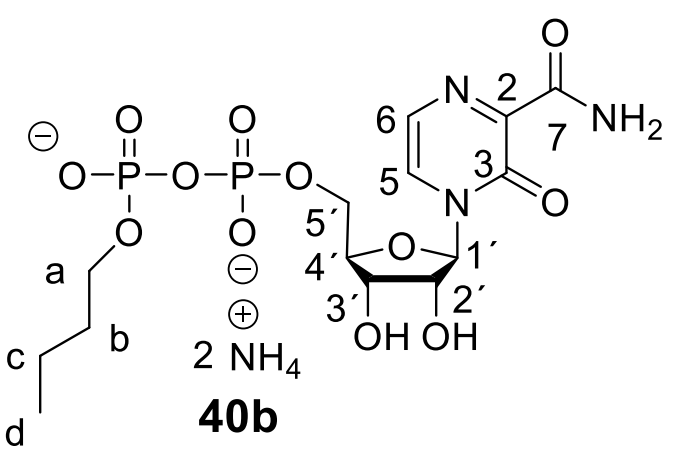

β-(Alkyl-C4)-T-1106-DP **40b**. According to general procedure with 95 mg (Fm;alkyl-C4)-*H*-phosphonate **36b** (0.3 mmol, 1.0 equiv.), 40 mg NCS (0.3 mmol, 1.0 equiv.), and 250 mg T-1106-MP 2×*n*Bu_4N^+ salt (0.3 mmol, 1.0 equiv.) and then stirred for 4 h at room temperature. Subsequently, 0.42 mL TEA (3.0 mmol, 10.0 equiv.) was added and stirred for 8 h. Yield: 75 mg (0.14 mmol, 48%) white solid. Purity (HPLC): > 96%. ^{1}H NMR (600 MHz, CD_3OD): δ [ppm] = 8.49 (d, $^3J_{HH}$= 4.2 Hz, 1H, H-5), 7.82 (d, $^3J_{HH}$= 4.2 Hz, 1H, H-6), 6.07 (d, $^3J_{HH}$= 2.0 Hz, 1H, H-1´), 4.45-4.17 (m, 5H, H-2´, H-3´, H-4´, H-5´), 3.97 (q, $^3J_{HH}$= 6.6 Hz, 2H, H-a), 1.66-1.56 (m, 2H, H-b), 1.45-1.35 (m, 2H, H-c), 0.90 (t, $^3J_{HH}$= 7.4 Hz, 3H, H-d). ^{13}C-NMR (151 MHz, CD_3OD): δ [ppm] = 166.4 (C-7), 157.1 (C-3), 143.7 (C-2), 130.8 (C-5), 126.0 (C-6), 92.4 (C-1´), 84.5 (d, $^3J_{CP}$= 9.3 Hz, C-4´), 76.9 (C-2´), 69.3 (C-a), 66.9 (d, $^3J_{CP}$= 6.2 Hz, C-3´), 64.6 (d, $^3J_{CP}$= 5.1 Hz, C-5´), 33.3 (d, $^3J_{CP}$= 8.4 Hz, C-b), 20.0 (C-c), 14.2 (C-d). ^{31}P NMR (243 MHz, CD_3OD): δ [ppm] = -10.76 (d, $^2J_{pp}$= 21.6 Hz, P-*β*), -11.40 (d, $^2J_{pp}$= 21.6 Hz, P-*α*). HRMS (ESI$^-$, m/z): $C_{14}H_{23}N_3O_{12}P_2$, [M-H]$^-$ 486.0684; found 486.0683.

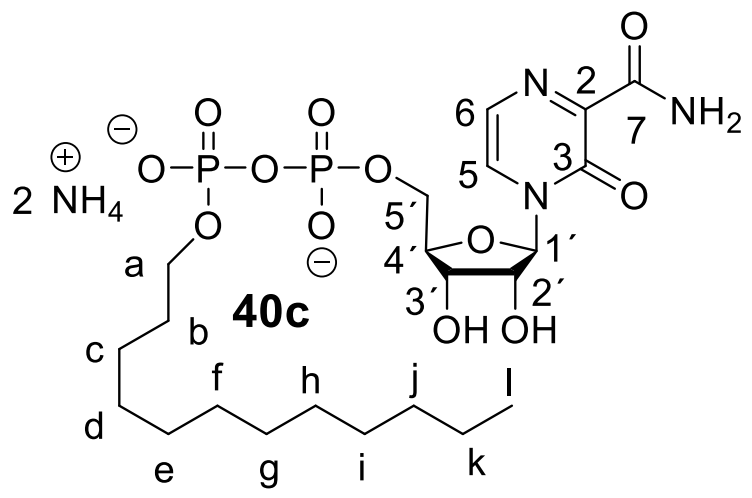

β-(Alkyl-C12)-T-1106-DP **40c**. According to general procedure with 129 mg (Fm;alkyl-C12)-*H*-phosphonate **36c** (0.3 mmol, 1.0 equiv.), 40 mg NCS (0.3 mmol, 1.0 equiv.), and 250 mg T-1106-MP 2×*n*Bu_4N^+ salt (0.3 mmol, 1.0 equiv.) and then stirred for 4 h at room temperature. Subsequently, 0.42 mL TEA (3.0 mmol, 10.0 equiv.) was added and stirred for 8 h. Yield: 78 mg (0.12 mmol, 41%) white solid. Purity (HPLC): > 97%. ^{1}H NMR (400 MHz, CD_3OD): δ [ppm] = 8.49 (d, $^3J_{HH}$= 4.2 Hz, 1H, H-5), 7.84 (d, $^3J_{HH}$= 4.2 Hz, 1H, H-6), 6.07 (d, $^3J_{HH}$= 2.0

Hz, 1H, H-1´), 4.45-4.18 (m, 5H, H-2´, H-3´, H-4´, H-5´), 3.98 (q, $^{3}J_{HH}$= 6.6 Hz, 2H, H-a), 1.68-1.58 (m, 2H, H-b), 1.38-1.25 (m, 18H, H-c, H-d, H-e, H-f, H-g, H-h, H-i, H-j, H-k), 0.90 (t, $^{3}J_{HH}$= 6.8 Hz, 3H, H-l). ^{13}C-NMR (101 MHz, CD_3OD): δ [ppm] = 166.3 (C-7), 157.1 (C-3), 143.8 (C-2), 130.8 (C-5), 126.1 (C-6), 92.4 (C-1´), 84.5 (d, $^{3}J_{CP}$= 9.3 Hz, C-4´), 76.9 (C-2´), 69.3 (C-a), 67.4 (d, $^{3}J_{CP}$= 6.0 Hz, C-3´), 64.7 (d, $^{3}J_{CP}$= 5.9 Hz, C-5´), 33.1, 30.81, 30.79, 30.76, 30.6, 30.5, 23.7 (C-d, C-e, C-f, C-g, C-h, C-i, C-j, C-k), 31.8 (d, $^{3}J_{CP}$ = 8.0 Hz, C-b), 26.9 (C-c), 14.4 (C-l). ^{31}P NMR (162 MHz, CD_3OD): δ [ppm] = -10.7o (d, $^{2}J_{pp}$= 21.6 Hz, P-β), -11.37 (d, $^{2}J_{pp}$= 21.6 Hz, P-α). HRMS (ESI$^-$, m/z): $C_{22}H_{39}N_3O_{12}P_2$, [M-H]$^-$ 598.1936; found 598.1935.

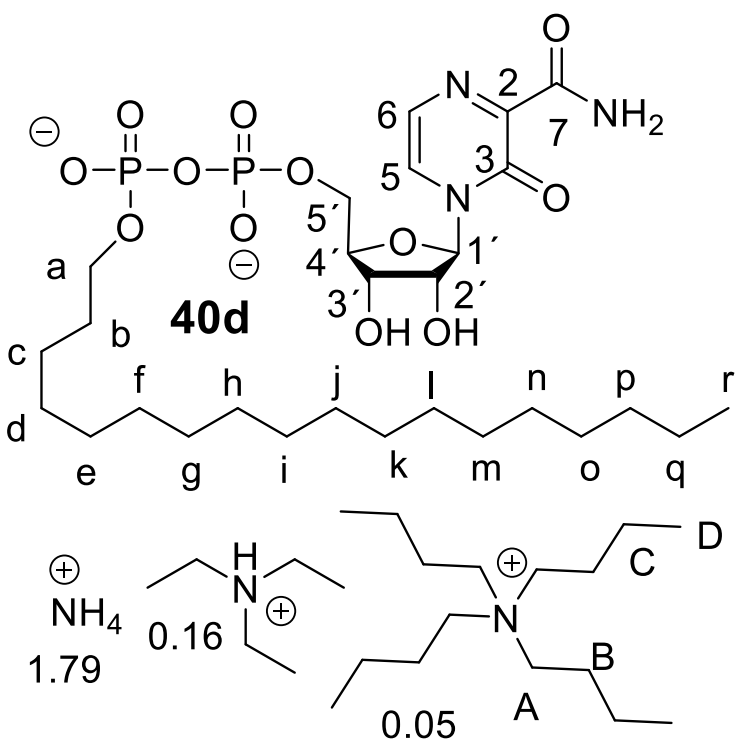

β-(Alkyl-C18)-T-1106-DP **40d**. According to general procedure with 154 mg (Fm;alkyl-C18)-*H*-phosphonate **36d** (0.3 mmol, 1.0 equiv.), 40 mg NCS (0.3 mmol, 1.0 equiv.), and 250 mg T-1106-MP 2×nBu$_4$N$^+$ salt (0.3 mmol, 1.0 equiv.) and then stirred for 4 h at room temperature. Subsequently, 0.42 mL TEA (3.0 mmol, 10.0 equiv.) was added and stirred for 12 h. Yield: 85 mg (0.11 mmol, 38%) white solid. Purity (HPLC): > 95%. ^{1}H NMR (400 MHz, CD_3OD): δ [ppm] = 8.34 (d, $^{3}J_{HH}$= 4.4 Hz, 1H, H-5), 7.75 (d, $^{3}J_{HH}$= 4.4 Hz, 1H, H-6), 6.07-6.05 (m, 1H, H-1´), 4.45-4.14 (m, 5H, H-2´, H-3´, H-4´, H-5´), 3.99 (q, $^{3}J_{HH}$= 6.4 Hz, 2H, H-a^{1}), 3.26-3.22 (m, 0.40H, H-A), 3.23-3.15 (m, 1.08H, HN(CH_2CH_3)$_3^+$), 1.68-1.58 (m, 2,4H, H-b, H-B), 1.48-1.40 (m, 32.0H, H-C, H-c, H-d, H-e, H-f, H-g, H-h, H-i, H-j, H-k, H-l, H-m, H-n, H-o, H-p, H-q, HN(CH_2CH_3)$_3^+$), 1.02 (t, $^{3}J_{HH}$= 7.3 Hz, 0.60H, H-D), 0.90 (t, $^{3}J_{HH}$= 6.8 Hz, 3H, H-r). ^{13}C-NMR (101 MHz, CD_3OD): δ [ppm] = 166.3 (C-7), 157.1 (C-3), 143.8 (C-2), 130.8 (C-5), 126.1 (C-6), 92.4 (C-1´), 84.5 (d, $^{3}J_{CP}$= 9.3 Hz, C-4´), 76.9 (C-2´), 69.3 (C-a), 67.4 (d, $^{3}J_{CP}$= 6.0 Hz, C-3´), 64.7 (d, $^{3}J_{CP}$= 5.9 Hz, C-5´), 47.6 (HN(CH_2CH_3)$_3^+$), 33.1, 30.79, 30.75, 30.6, 30.5, 23.7 (C-d, C-e, C-f, C-g, C-h, C-i, C-j, C-k, C-l, C-m, C-n, C-o, C-p, C-q), 31.8 (d, $^{3}J_{CP}$ = 7.6 Hz, C-b), 26.9 (C-c), 14.4 (C-r), 9.1 (HN(CH_2CH_3)$_3^+$). ^{31}P NMR (162 MHz, CD_3OD): δ [ppm] = -10.76 (d, $^{2}J_{pp}$= 21.6 Hz, P-β), -11.40 (d, $^{2}J_{pp}$= 21.6 Hz, P-α). HRMS (ESI$^-$, m/z): $C_{28}H_{51}N_3O_{12}P_2$, [M-H]$^-$ 682.2875; found 682.2880.

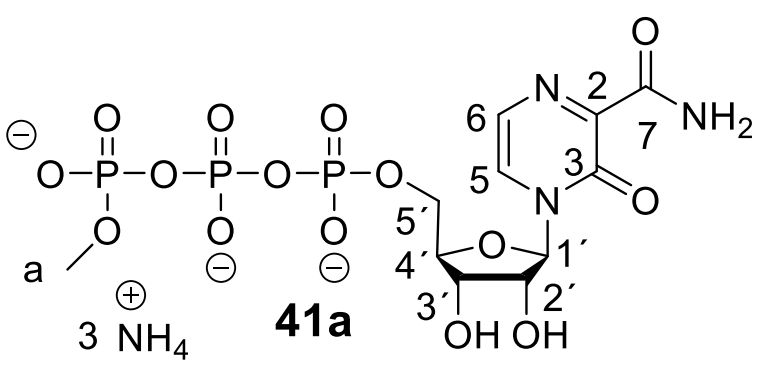

γ-(Alkyl-C1)-T-1106-TP **41a**. According to general procedure with 82 mg (Fm;alkyl-C1)-*H*-phosphonate **36a** (0.3 mmol, 1.0 equiv.), 40 mg NCS (0.3 mmol, 1.0 equiv.), and 346 mg T-1106-DP 3×nBu$_4$N$^+$ salt (0.3 mmol, 1.0 equiv.) and then stirred for 4 h at room temperature. Subsequently, 0.42 mL TEA (3.0 mmol, 10.0 equiv.) was added and stirred for 8 h. Yield: 36 mg (0.06 mmol, 21%) white solid. Purity (HPLC): > 96%. ^{1}H NMR (400 MHz, D_2O): δ [ppm] = 8.39 (d, $^{3}J_{HH}$= 4.2 Hz, 1H, H-5), 7.86 (d, $^{3}J_{HH}$= 4.2 Hz, 1H, H-6), 6.15 (d, $^{4}J_{HH}$= 1.4 Hz, 1H, H-1´), 4.48-4.28 (m, 5H, H-2´, H-3´, H-4´, H-5´), 3.67 (d, $^{2}J_{HH}$= 11.4 Hz, 2H, H-a). ^{13}C-NMR (101 MHz, D_2O): δ [ppm] = 166.0 (C-7), 155.9 (C-3), 142.4 (C-2), 129.4 (C-5), 125.4 (C-6), 90.7 (C-1´), 82.7 (d, $^{3}J_{CP}$= 9.2 Hz, C-4´), 74.9 (C-2´), 68.0 (C-3´), 63.7 (C-5´), 53.44,53.38 (C-a, C-4´). ^{31}P NMR (162 MHz, D_2O): δ [ppm] = -9.76 (d, $^{2}J_{pp}$= 19.5 Hz, P-α), -11.61 (d, $^{2}J_{pp}$= 19.5, P-γ), -23.23 (d, $^{3}J_{pp}$= 19.6 Hz, P-β). HRMS (ESI$^-$, m/z): $C_{11}H_{18}N_3O_{15}P_3$, [M-H]$^-$ 523.9878; found 523.9909.

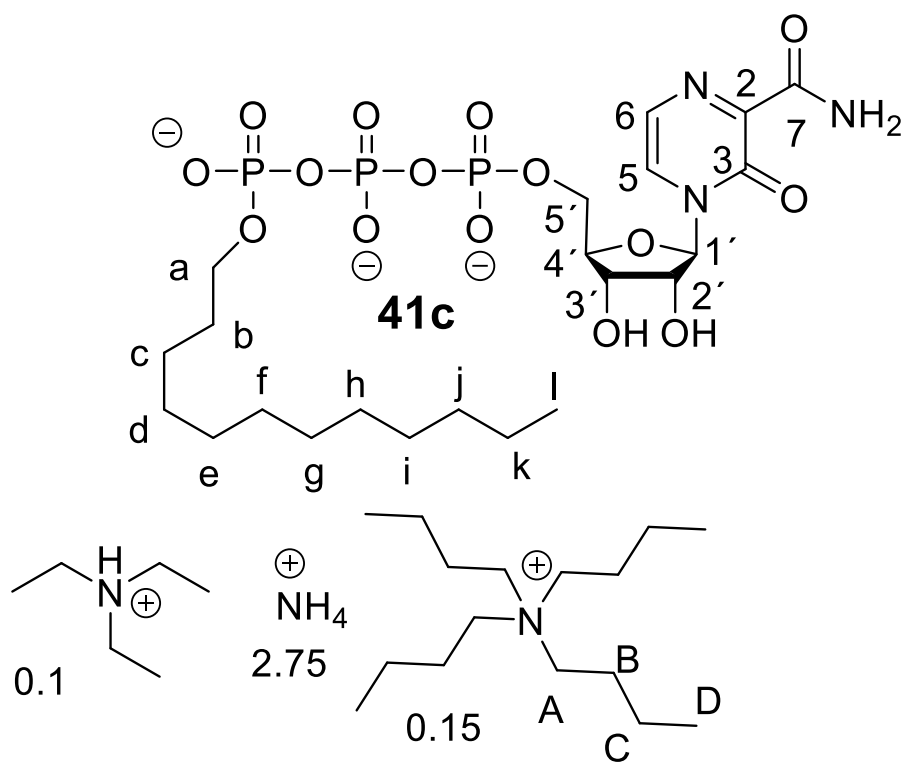

γ-(Alkyl-C12)-T-1106-TP **41c**. According to general procedure with 129 mg (Fm;alkyl-C12)-*H*-phosphonate **36c** (0.3 mmol, 1.0 equiv.), 40 mg NCS (0.3 mmol, 1.0 equiv.), and 346 mg T-1106-DP 3×*n*Bu_4N^+ salt (0.3 mmol, 1.0 equiv.) and then stirred for 4 h at room temperature. Subsequently, 0.42 mL TEA (3.0 mmol, 10.0 equiv.) was added and stirred for 8 h. Yield: 67 mg (0.08 mmol, 28%) white solid. Purity (HPLC): > 96%. ^{1}H NMR (600 MHz, CD_3OD): δ [ppm] = 8.50 (d, $^3J_{HH}$= 4.2 Hz, 1H, H-5), 7.82 (d, $^3J_{HH}$= 4.2 Hz, 1H, H-6), 6.08 (d, $^3J_{HH}$= 2.0 Hz, 1H, H-1´), 4.45-4.18 (m, 5H, H-2´, H-3´, H-4´, H-5´), 3.97 (q, $^3J_{HH}$= 6.8 Hz, 2H, H-a), 3.27-3.22 (m, 1.2H, H-A), 3.19 (q, $^3J_{HH}$= 7.3 Hz, 0.6H, $HN(CH_2CH_3)_3^+$), 1.68-1.62 (m, 3.2H, H-b, H-B), 1.44-1.38 (m, 3.2H, H-c, H-C), 1.34-1.25 (m, 16.9H, H-d, H-e, H-f, H-g, H-h, H-i, H-j, H-k), 1.03 (t, $^3J_{HH}$= 7.3 Hz, 0.3H, H-D), 0.90 (t, $^3J_{HH}$= 6.8 Hz, 3H, H-l). ^{13}C-NMR (151 MHz, CD_3OD): δ [ppm] = 166.3 (C-7), 157.1 (C-3), 143.7 (C-2), 130.8 (C-5), 126.0 (C-6), 92.4 (C-1´), 84.5 (C-4´), 76.9 (C-2´), 69.2 (C-a), 67.4 (d, $^3J_{CP}$= 6.0 Hz, C-3´), 64.8 (C-5´), 59.5 (C-A), 33.1, 30.82, 30.79, 30.77, 30.6, 30.5, 23.7 (C-d, C-e, C-f, C-g, C-h, C-i, C-j, C-k), 31.8 (d, $^3J_{CP}$ = 8.0 Hz, C-b), 26.9 (C-c), 24.8 (C-B), 20.7 (C-C), 14.4 (C-l), 13.9 (C-D). ^{31}P NMR (243 MHz, CD_3OD): δ [ppm] = -10.83 (d, $^2J_{pp}$= 17.7 Hz, P-*α*), -11.46 (d, $^2J_{pp}$= 20.5, P-*γ*), -22.51 (d, $^3J_{pp}$= 17.9 Hz, P-*β*). HRMS (ESI$^-$, m/z): $C_{22}H_{40}N_3O_{15}P_3$, $[M-H]^-$ 678.1600; found 678.1395.

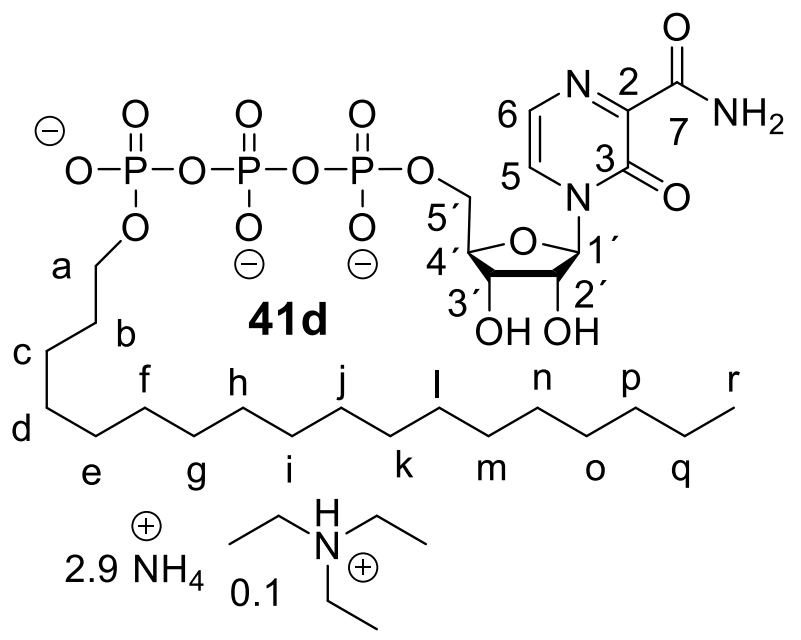

γ-(Alkyl-C18)-T-1106-TP **41d**. According to general procedure with 154 mg (Fm;alkyl-C18)-*H*-phosphonate **36d** (0.3 mmol, 1.0 equiv.), 40 mg NCS (0.3 mmol, 1.0 equiv.), and 346 mg T-1106-DP 3×*n*Bu_4N^+ salt (0.3 mmol, 1.0 equiv.) and then stirred for 4 h at room temperature. Subsequently, 0.42 mL TEA (3.0 mmol, 10.0 equiv.) was added and stirred for 12 h. Yield: 76 mg (0.09 mmol, 31%) white solid. Purity (HPLC): > 99%. ^{1}H NMR (600 MHz, CD_3OD): δ [ppm] = 8.48 (d, $^3J_{HH}$= 4.2 Hz, 1H, H-5), 7.83 (d, $^3J_{HH}$= 4.2 Hz, 1H, H-6), 6.08 (d, $^3J_{HH}$= 2.1 Hz, 1H, H-1´), 4.45-4.18 (m, 5H, H-2´, H-3´, H-4´, H-5´), 3.98 (q, $^3J_{HH}$= 6.6 Hz, 2H, H-a), 3.20 (q, $^3J_{HH}$= 7.4 Hz, 0.6H, $HN(CH_2CH_3)_3^+$), 1.66-1.58 (m, 2H, H-b), 1.48-1.40 (m, 30.9H, H-c, H-d, H-e, H-f, H-g, H-h, H-i, H-j, H-k, H-l, H-m, H-n, H-o, H-p, H-q, $HN(CH_2CH_3)_3^+$), 0.90 (t, $^3J_{HH}$= 6.9 Hz, 3H, H-r). ^{13}C-NMR (151 MHz, CD_3OD): δ [ppm] = 166.4 (C-7), 157.1 (C-3), 143.8 (C-2), 130.8 (C-5), 126.0 (C-6), 92.4 (C-1´), 84.5 (d, $^3J_{CP}$= 8.6 Hz, C-4´), 76.9 (C-2´), 69.2 (C-a), 67.6 (d, $^3J_{CP}$= 6.1 Hz, C-3´), 64.9 (C-5´), 47.6 ($HN(CH_2CH_3)_3^+$), 33.1, 30.79, 30.75, 30.6, 30.5, 23.7 (C-d, C-e, C-f, C-g, C-h, C-i, C-j, C-k, C-l, C-m, C-n, C-o, C-p, C-q), 31.8 (d, $^3J_{CP}$ = 7.9 Hz, C-b), 26.9 (C-c), 14.4 (C-r), 9.3 ($HN(CH_2CH_3)_3^+$). ^{31}P NMR (243 MHz, CD_3OD): δ [ppm] = -10.96 (d, $^2J_{pp}$= 20.2 Hz, P-*α*), -11.53 (d, $^2J_{pp}$= 17.5, P-*γ*), -22.75 (d, $^3J_{pp}$= 19.2 Hz, P-*β*). HRMS (ESI$^-$, m/z): $C_{28}H_{52}N_3O_{15}P_3$, $[M-H]^-$ 762.2539; found 762.2451.

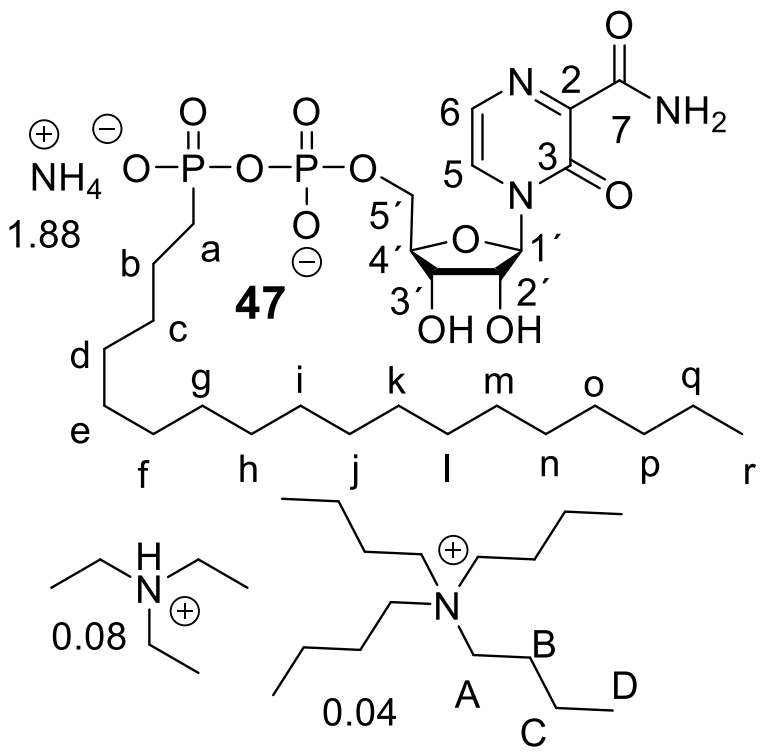

β-C-(Alkyl-C18)-T-1106-DP **47**. According to general procedure with 149 mg (Fm;alkyl-C18)-*H*-phosphinate **44** (0.3 mmol, 1.0 equiv.), 40 mg NCS (0.3 mmol, 1.0 equiv.), and 250 mg T-1106-MP 2×*n*Bu_4N^+ salt (0.3 mmol, 1.0 equiv.) and then stirred for 4 h at room temperature. Subsequently, 0.42 mL TEA (3.0 mmol, 10.0 equiv.) was added and stirred for 12 h. Yield: 94 mg (0.13 mmol, 44%) white solid. Purity (HPLC): > 99%. ^{1}H NMR (400 MHz, CD_3OD): δ [ppm] = 8.48 (d, $^{3}J_{HH}$= 4.2 Hz, 1H, H-5), 7.83 (d, $^{3}J_{HH}$= 4.2 Hz, 1H, H-6), 6.07 (d, $^{3}J_{HH}$= 2.0 Hz, 1H, H-1´), 4.45-4.14 (m, 5H, H-2´, H-3´, H-4´, H-5´), 3.26-3.22 (m, 0.32H, H-A), 3.22-3.15 (m, 0.48H, $HN(CH_2CH_3)_3^+$), 1.85-1.76 (m, 2H, H-a), 1.68-1.62 (m, 2.3H, H-b, H-B), 1.44-1.26 (m, 31H, H-C, H-c, H-d, H-e, H-f, H-g, H-h, H-i, H-j, H-k, H-l, H-m, H-n, H-o, H-p, H-q, $HN(CH_2CH_3)_3^+$), 1.02 (t, $^{3}J_{HH}$= 7.3 Hz, 0.48H, H-D), 0.90 (t, $^{3}J_{HH}$= 6.6 Hz, 3H, H-r). ^{13}C-NMR (101 MHz, CD_3OD): δ [ppm] = 166.2 (C-7), 157.1 (C-3), 143.9 (C-2), 130.7 (C-5), 126.0 (C-6), 92.4 (C-1´), 84.6 (C-4´), 76.9 (C-2´), 69.3 (C-3´), 64.6 (C-5´), 33.1, 32.3, 30.1, 30.79, 30.74, 30.68, 30.5, 23.7 (C-c, C-d, C-e, C-f, C-g, C-h, C-i, C-j, C-k, C-l, C-m, C-n, C-o, C-p, C-q), 30.1, 28.7 (C-a^{1}), 24.4 (C-B), 14.4 (C-r). ^{31}P NMR (162 MHz, CD_3OD): δ [ppm] = 24.84 (dd, $^{2}J_{pp}$= 25.8 Hz, $^{3}J_{pp}$= 4.0 Hz, P-*β*), -11.62 (dd, $^{2}J_{pp}$= 25.4 Hz, $^{3}J_{pp}$= 3.6 Hz, P-*α*). HRMS (ESI$^-$, m/z): $C_{28}H_{51}N_3O_{11}P_2$, $[M-H]^-$ 666.2926; found 666.2898.

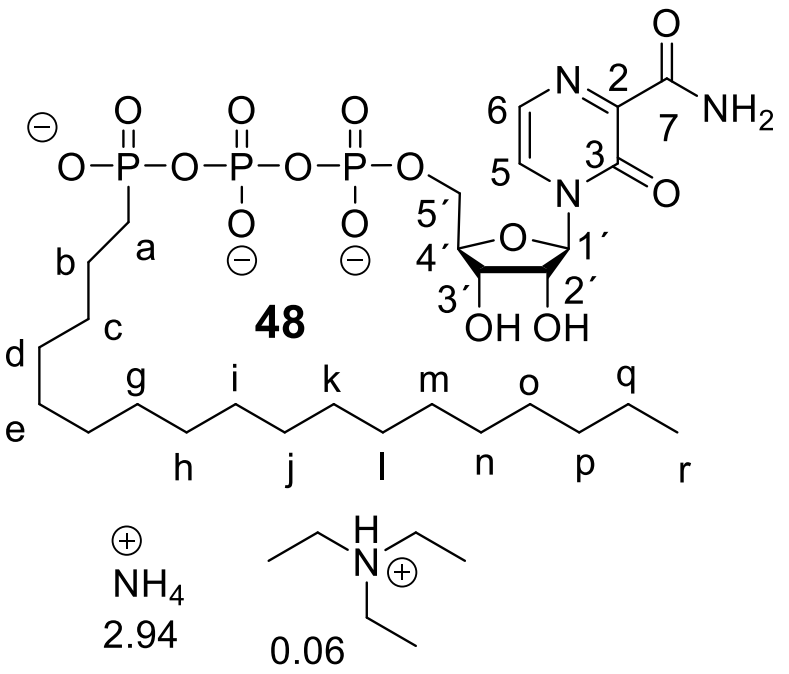

γ-C-(Alkyl-C18)-T-1106-TP **48**. According to general procedure with 149 mg (Fm;alkyl-C18)-*H*-phosphinate **44** (0.3 mmol, 1.0 equiv.), 40 mg NCS (0.3 mmol, 1.0 equiv.), and 346 mg T-1106-DP 3×*n*Bu_4N^+ salt (0.3 mmol, 1.0 equiv.) and then stirred for 4 h at room temperature. Subsequently, 0.42 mL TEA (3.0 mmol, 10.0 equiv.) was added and stirred for 12 h. Yield: 83 mg (0.11 mmol, 36%) white solid. Purity (HPLC): > 95%. ^{1}H NMR (600 MHz, CD_3OD): δ [ppm] = 8.48 (d, $^{3}J_{HH}$= 4.2 Hz, 1H, H-5), 7.82 (d, $^{3}J_{HH}$= 4.2 Hz, 1H, H-6), 6.08 (d, $^{3}J_{HH}$= 2.0 Hz, 1H, H-1´), 4.45-4.18 (m, 5H, H-2´, H-3´, H-4´, H-5´), 3.22-3.15 (m, 0.36H, $HN(CH_2CH_3)_3^+$), 1.86-1.80 (m, 2H, H-a), 1.66-1.60 (m, 2H, H-b), 1.42-1.35 (m, 2H, H-c), 1.34-1.26 (m, 28.54H, H-d, H-e, H-f, H-g, H-h, H-i, H-j, H-k, H-l, H-m, H-n, H-o, H-p, H-q, $HN(CH_2CH_3)_3^+$), 0.90 (t, $^{3}J_{HH}$= 6.9 Hz, 3H, H-r). ^{13}C-NMR (151 MHz, CD_3OD): δ [ppm] = 166.4 (C-7), 157.1 (C-3), 143.8 (C-2), 130.7 (C-5), 126.0 (C-6), 92.4 (C-1´), 84.6 (d, $^{3}J_{CP}$= 9.0 Hz, C-4´), 76.9 (C-2´), 69.3 (C-3´), 65.0 (C-5´), 33.1, 30.81, 30.79, 30.75, 30.69, 30.5, 23.7 (C-c, C-d, C-e, C-f, C-g, C-h, C-i, C-j, C-k, C-l, C-m, C-n, C-o, C-p, C-q), 32.1 (d, $^{2}J_{CP}$ = 18.2 Hz, C-b), 29.5, 28.6 (C-a^{1}), 24.4 (d, $^{3}J_{CP}$= 5.0 Hz, C-B), 20.0 (C-C), 14.4 (C-r). ^{31}P NMR (243 MHz, CD_3OD): δ [ppm] = 20.96 (d, $^{2}J_{pp}$= 26.5, P-*γ*), -11.58 (d, $^{2}J_{pp}$= 20.6 Hz, P-*α*), -23.61 (t, $^{2}J_{pp}$= 24.9 Hz, P-*β*). HRMS (ESI$^-$, m/z): $C_{28}H_{52}N_3O_{14}P_3$, $[M-H]^-$ 746.2589; found 746.2439.

Part 3. Hydrolysis in PBS at pH 7.3 or by pig liver esterase

Starting from 50 mM stocks of compounds **22**-**33** in 100% DMSO, 0.9 mM working solutions were prepared by mixing 22 µL stock, 378 µL water, 200 µL DMSO and 600 µL PBS (phosphate buffered saline) at pH 7.3. The tubes were incubated in a thermomixer at 37 °C and, at different time points, 40 µL was taken out and frozen (- 30 °C), until analysis by ion-pairing RP-HPLC (Method 1).

Pig liver esterase (PLE) was purchased from Sigma-Aldrich and diluted in PBS to 100 units/mL. The working solutions of compounds **22-33** were prepared in three steps by combining: (i) 10 µL stock (50 mM in DMSO) with 32 µL DMSO and 42 µL water; (ii)125 µL DMSO and 833 µL PBS buffer; and (iii) 62 µL of diluted PLE. The tubes were incubated at 37 °C and, at different time points, 75 µL was taken out, mixed with 79.6 µL MeOH to stop the enzymatic reaction, and frozen until HPLC analysis (Method 1). The half-lives ($t_{1/2}$) after hydrolysis in PBS of PLE were calculated using Origin software.

Part 4. Hydrolysis in crude CEM/0 cell extract or in human plasma

CEM/0 cells were grown in RPMI 1640-based cell culture medium to a final density of ~3 x 10^6 cells/mL. Cells were centrifuged for 10 min at 1,250 rpm at 4 °C, washed twice with cold PBS, and the pellet was resuspended at 10^8 cells/mL and sonicated (Hielscher Ultrasound Techn., 100% amplitude, 3·times for 10 s) to destroy cell integrity. After centrifugating the cell lysate at 10,000 rpm to remove cell debris, the supernatant was frozen in aliquots at -80 °C.

To prepare the working solutions of compounds **22-33** at 0.85 mM, we combined 21 µL stock (50 mM in DMSO), 154 µL DMSO and 175 µL water, then divided the mix over different aliquots of 20 µL to next add 50 µL CEM/0 cell extract in each aliquot. After incubation at 37 °C for different time periods, the reaction was stopped by adding 150 µL MeOH and keeping the samples on ice for 5 min. After centrifugation at 14,000 rpm for 22 min, the supernatants were filtered (Chromafil RC-20/15 MS, 0.2 µm) and stored in liquid nitrogen, until analysis by HPLC (Method 1).

For the experiments with human plasma, we obtained whole blood samples anticoagulated with 0.106 M citrate. After centrifugation (3,000 x g for 10 min at RT), the supernatant was frozen in aliquots at -80 °C. The working solutions of compounds **22-33** at 0.85 mM were prepared by mixing 21 µL stock (50 mM in DMSO), 154 µL DMSO and 175 µL water; dividing these mixes in 10-µL aliquots; and adding 25 µL human plasma to each aliquot. The incubation, protein precipitation and sample filtration were conducted as above. For HPLC analysis, we used Method 2. The half-lives ($t_{1/2}$) were again calculated using Origin software.

Part 5. Figure S1-S10. HPLC profiles of T-1106-Di*PP*ro- and Tri*PPP*ro-compounds after incubation in PBS (pH 7.3) and PLE.

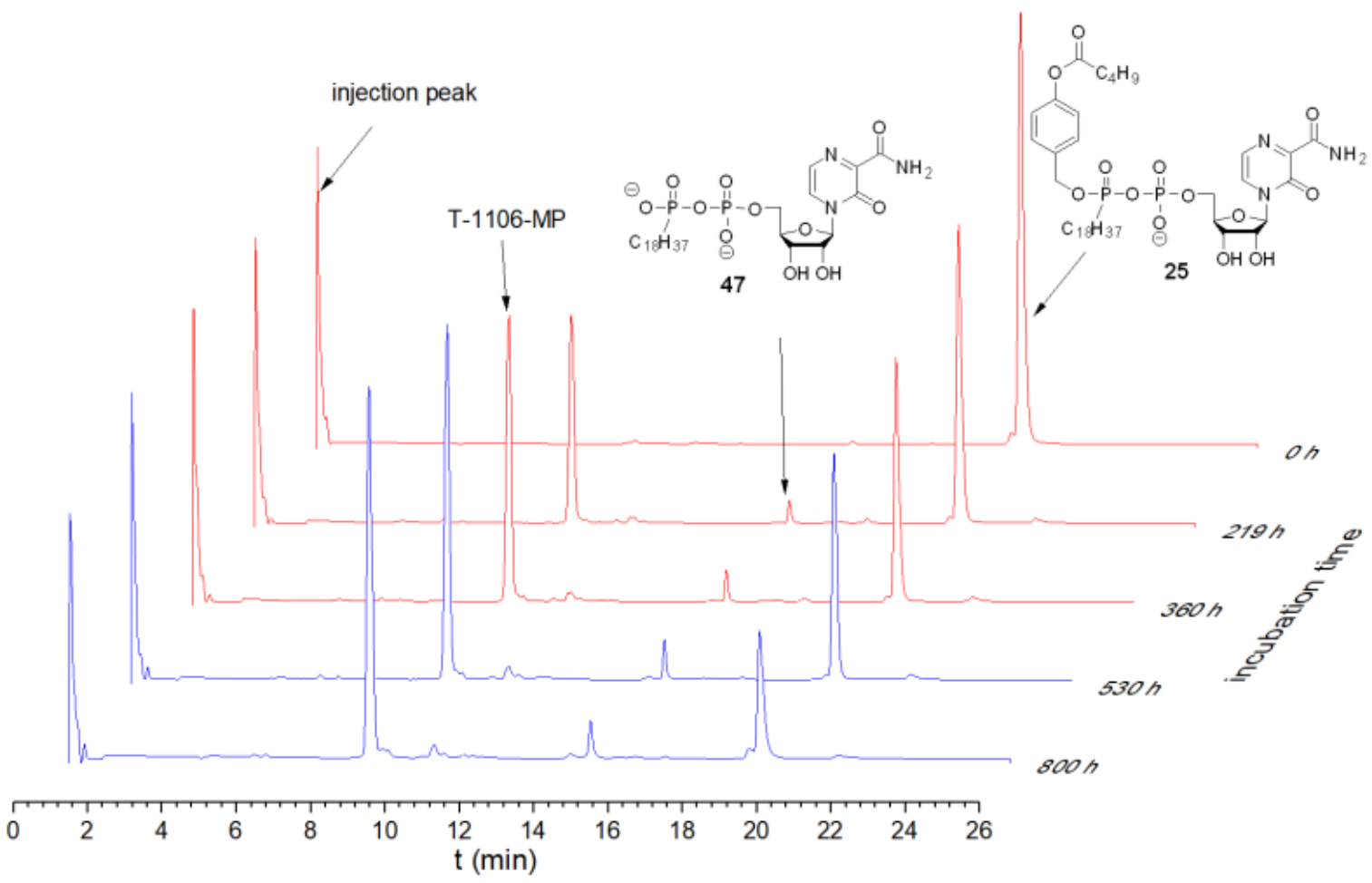

Figure S1. HPLC profile of T-1106-Di*PP*ro-compound **25** after incubation in PBS.

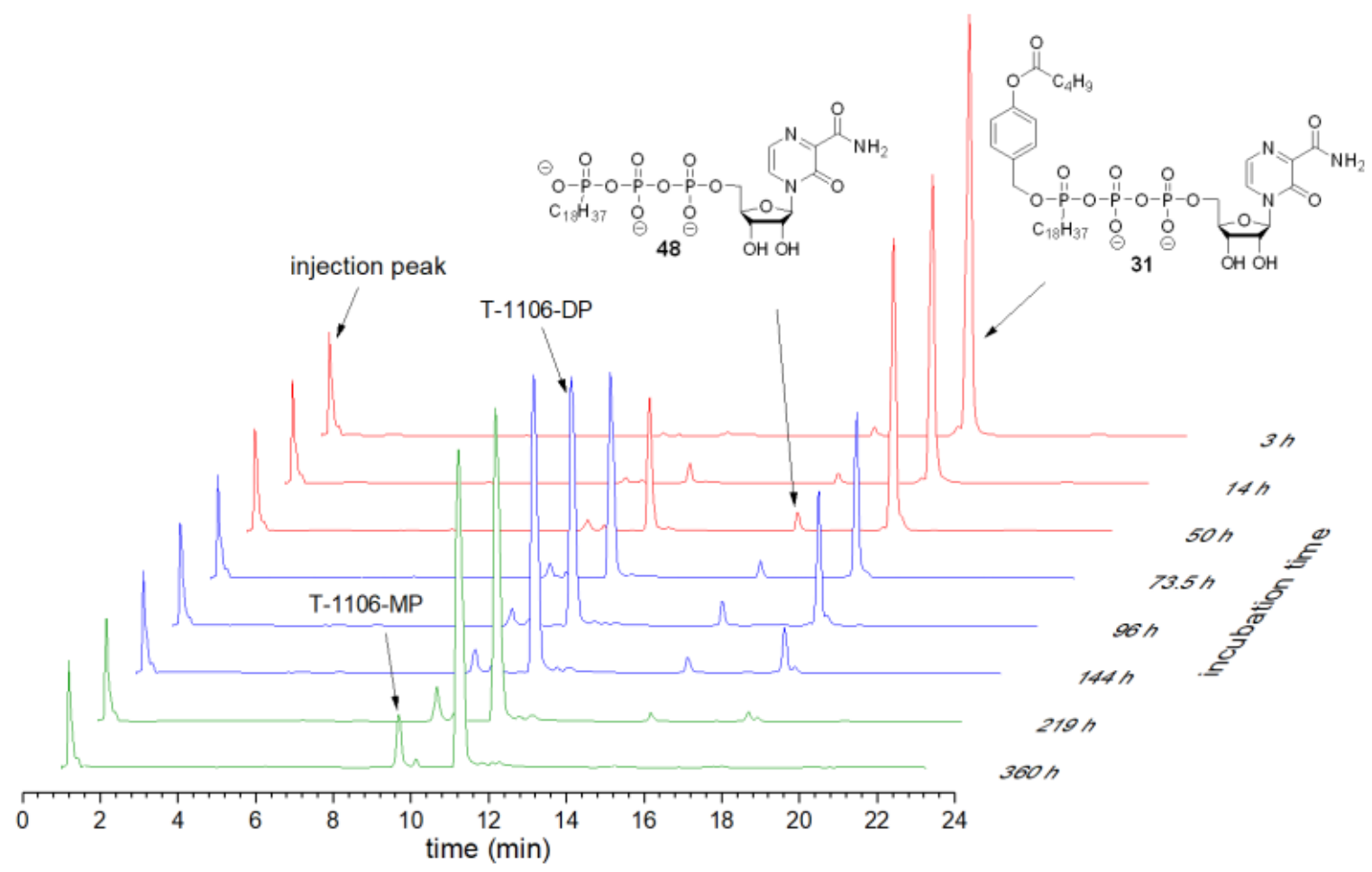

Figure S2. HPLC profile of T-1106-Tri*PP*Pro-compound **31** after incubation in PBS.

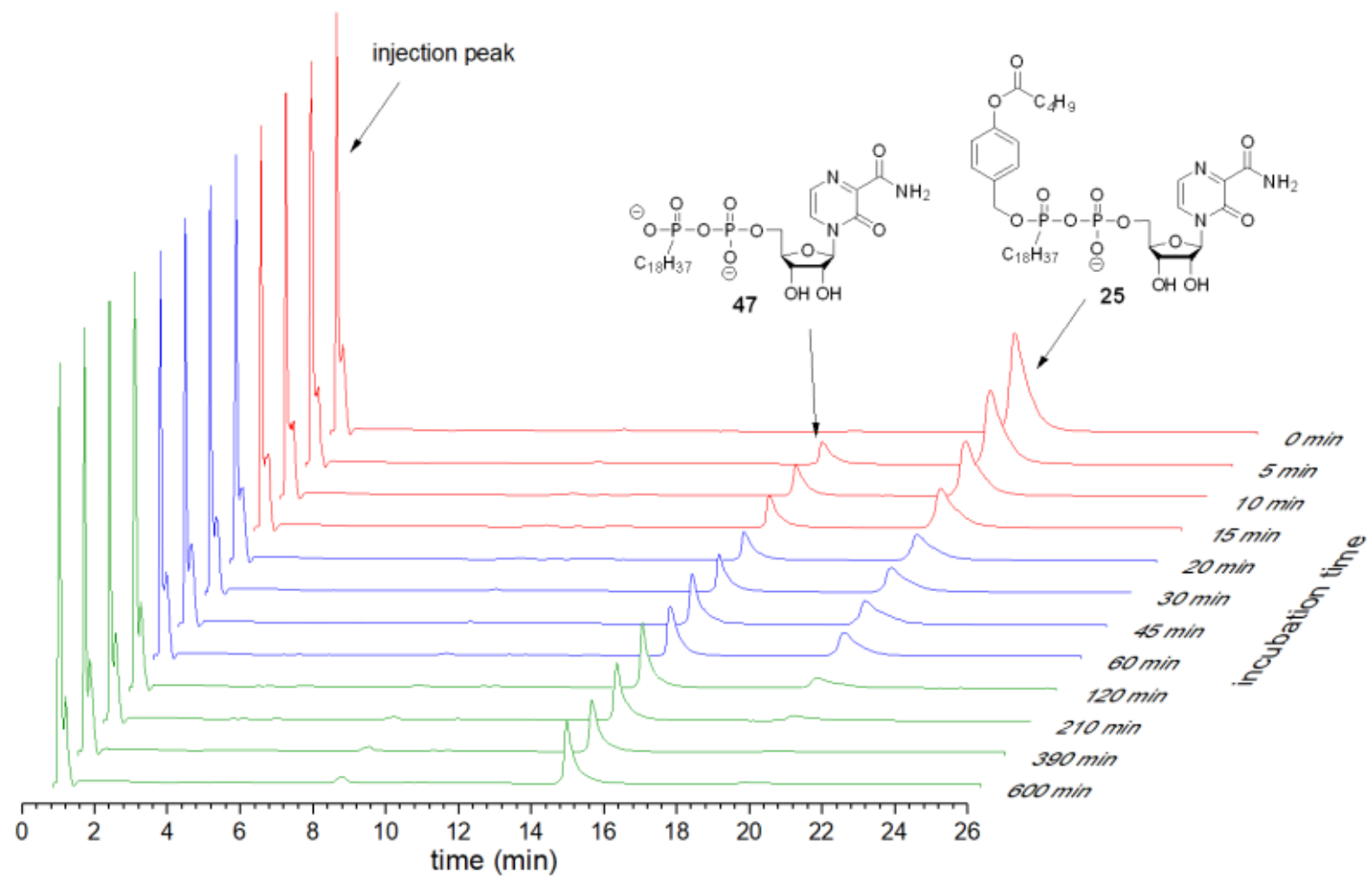

Figure S3. HPLC profile of T-1106-Di*PP*Pro-compound **25** after incubation with PLE.

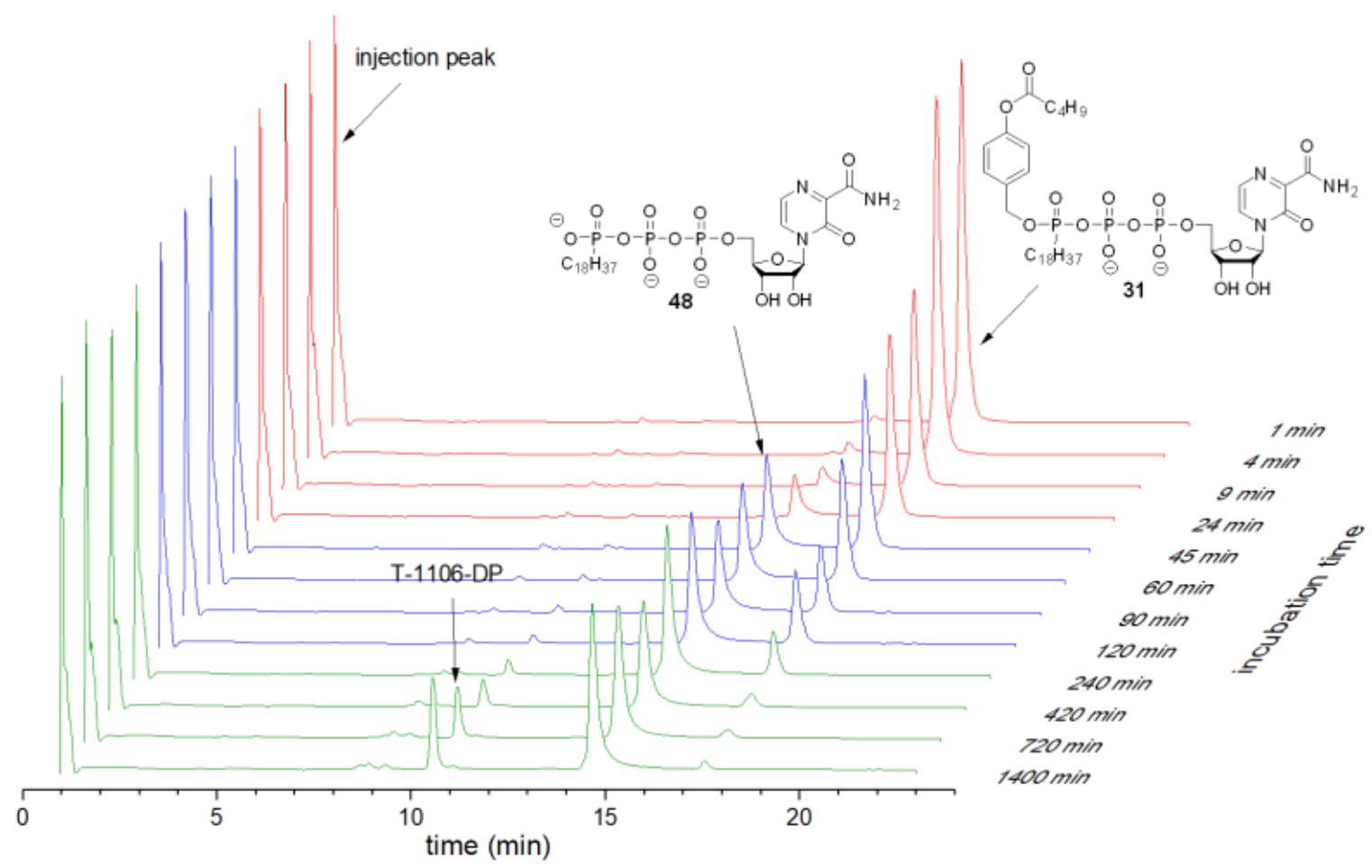

Figure S4. HPLC profile of T-1106-Tri*PP*Pro-compound **31** after incubation with PLE.

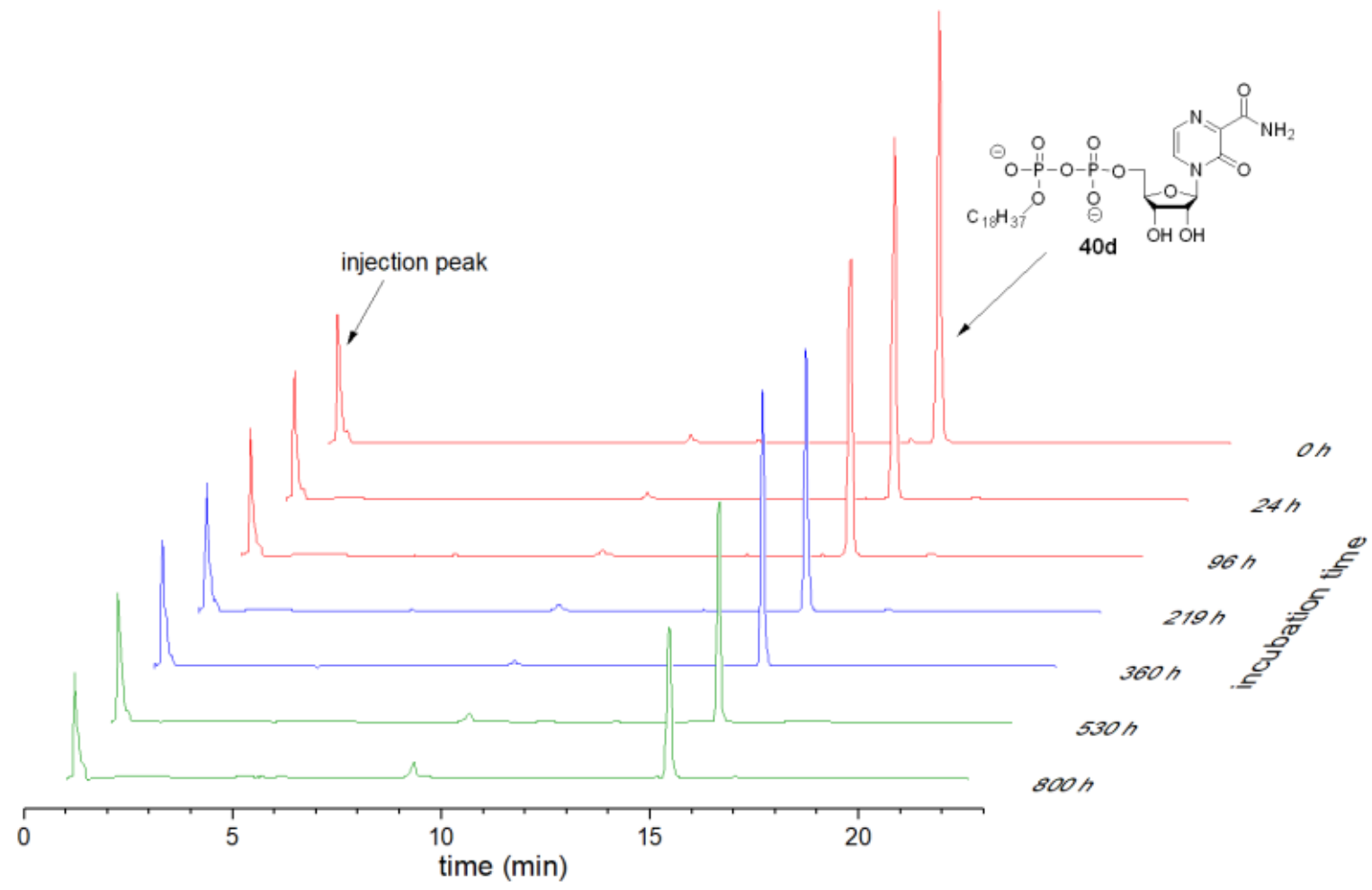

Figure S5. HPLC profile of T-1106-Di*PP*ro-compound **40d** after incubation in PBS.

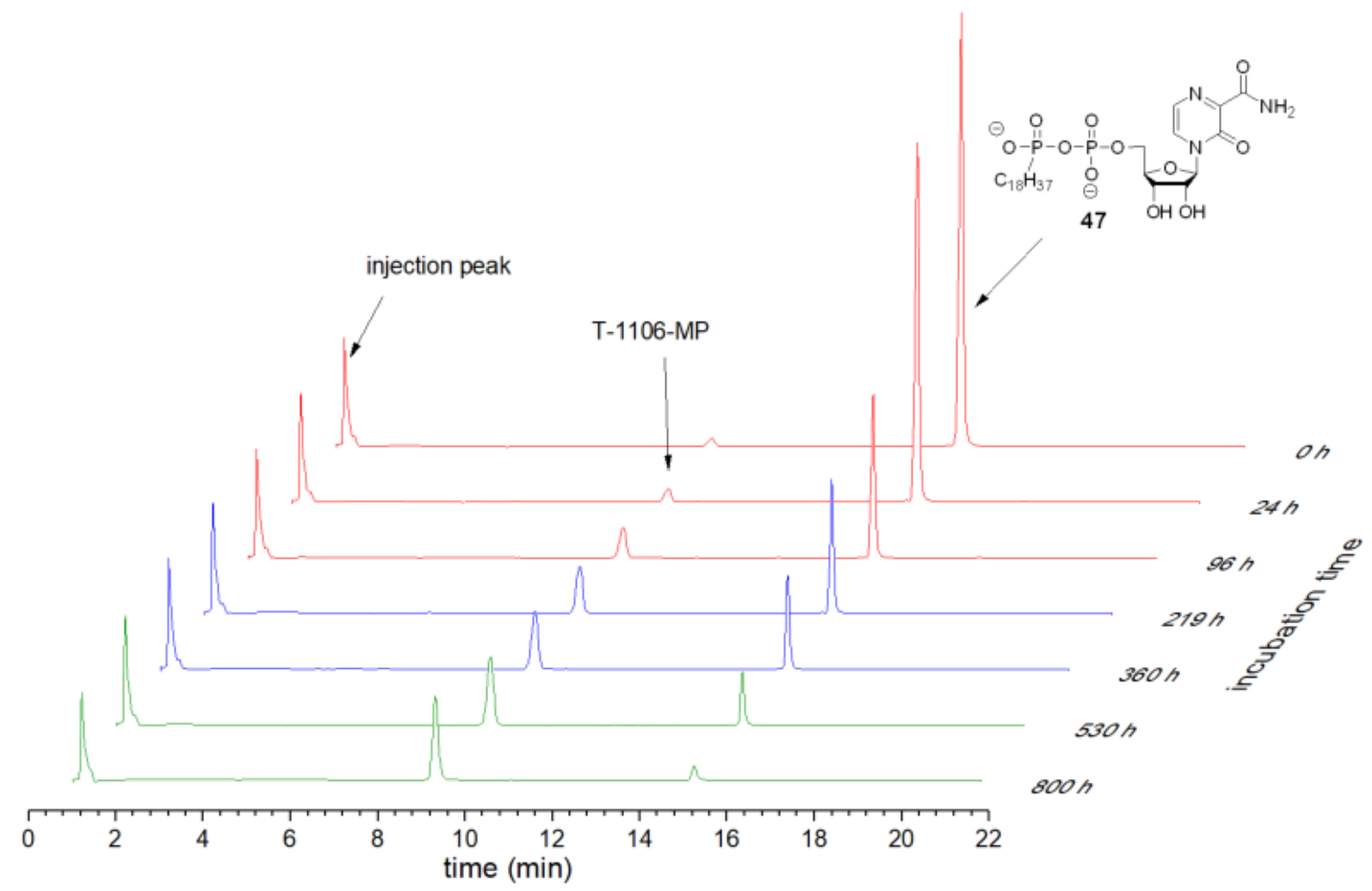

Figure S6. HPLC profile of T-1106-Di*PP*ro-compound **47** after incubation in PBS.

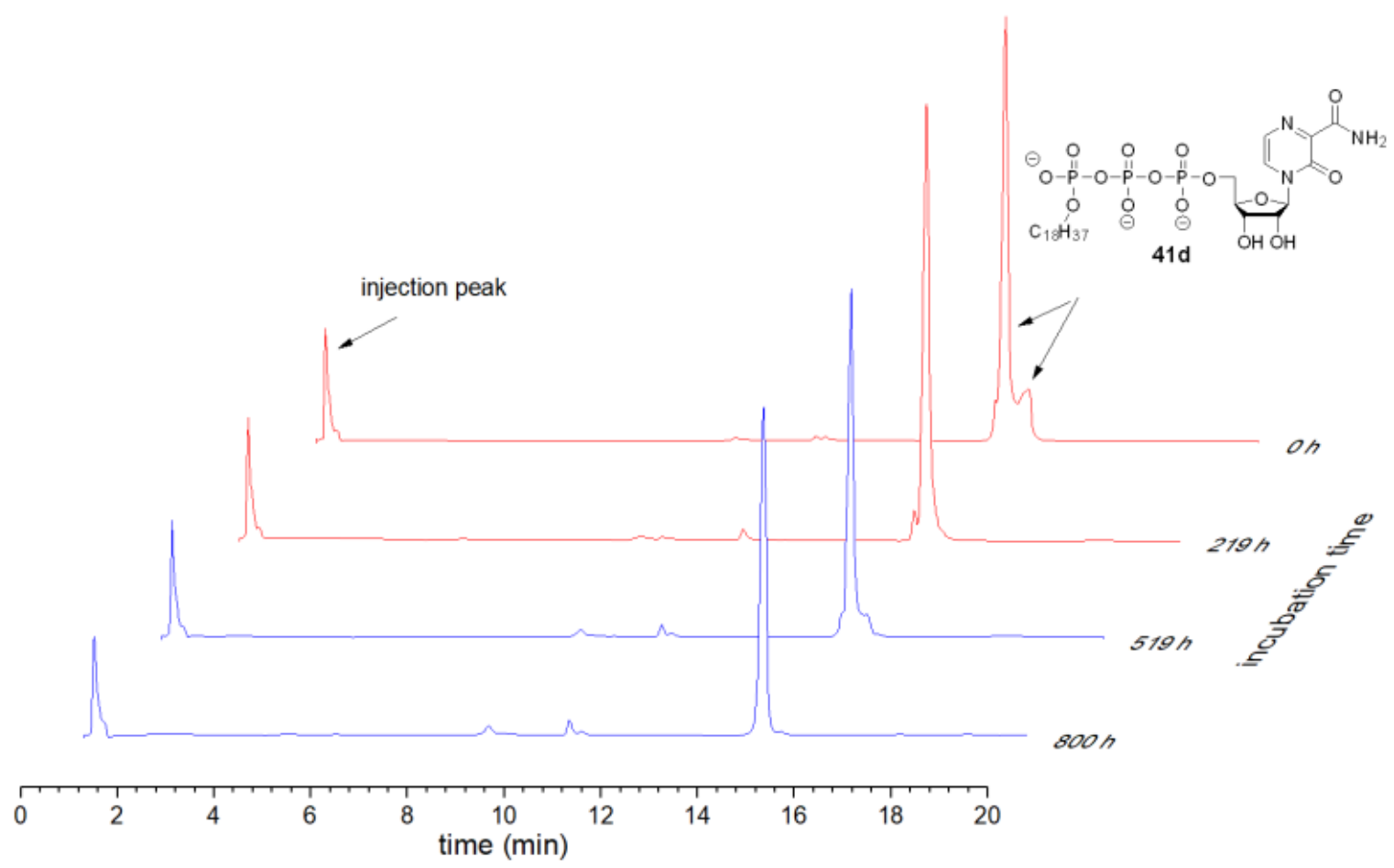

Figure S7. HPLC profile of T-1106-Tri*PPP*ro-compound **41d** after incubation in PBS.

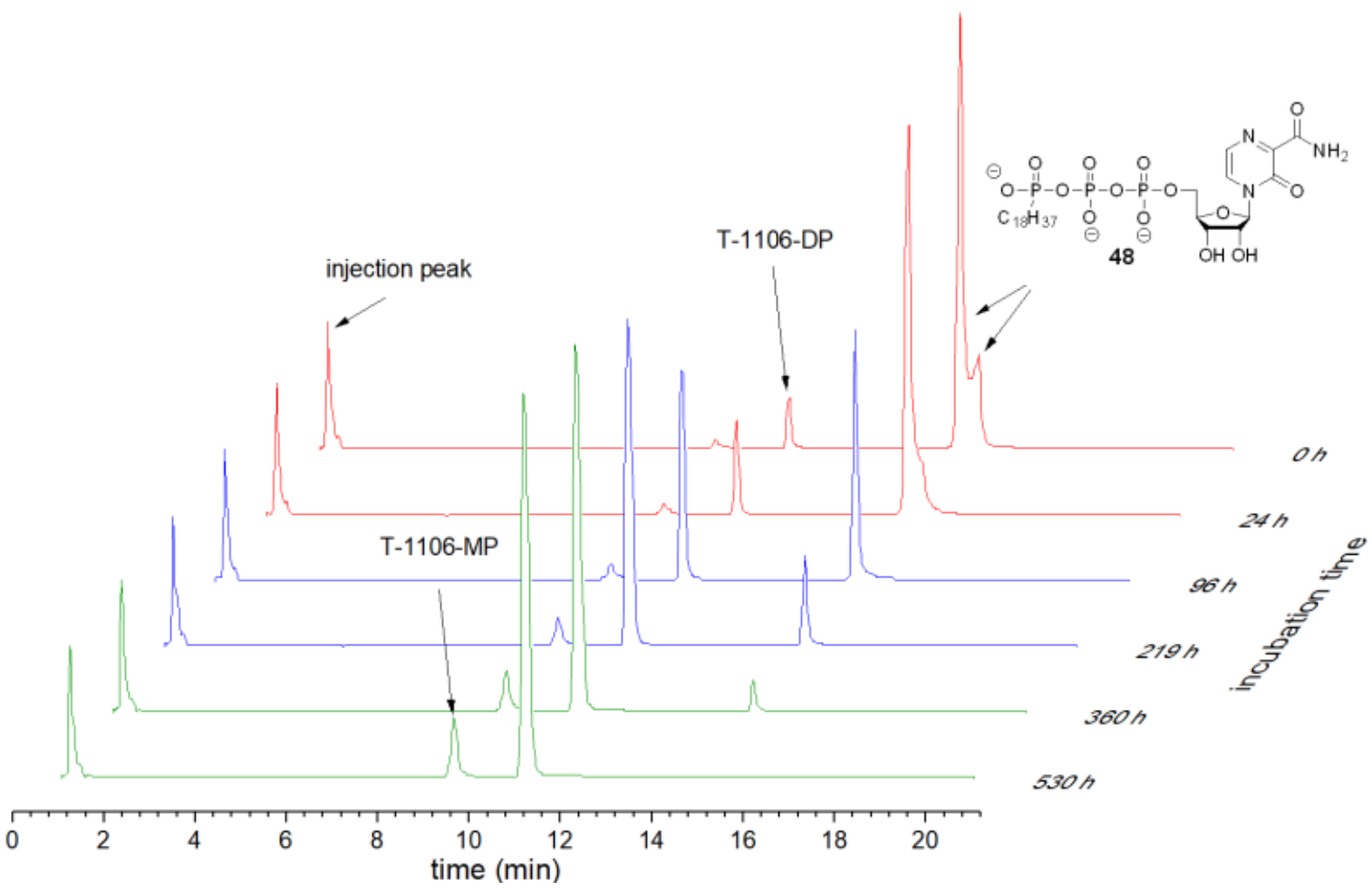

Figure S8. HPLC profile of T-1106-Tri*PP*Pro-compound **48** after incubation in PBS.

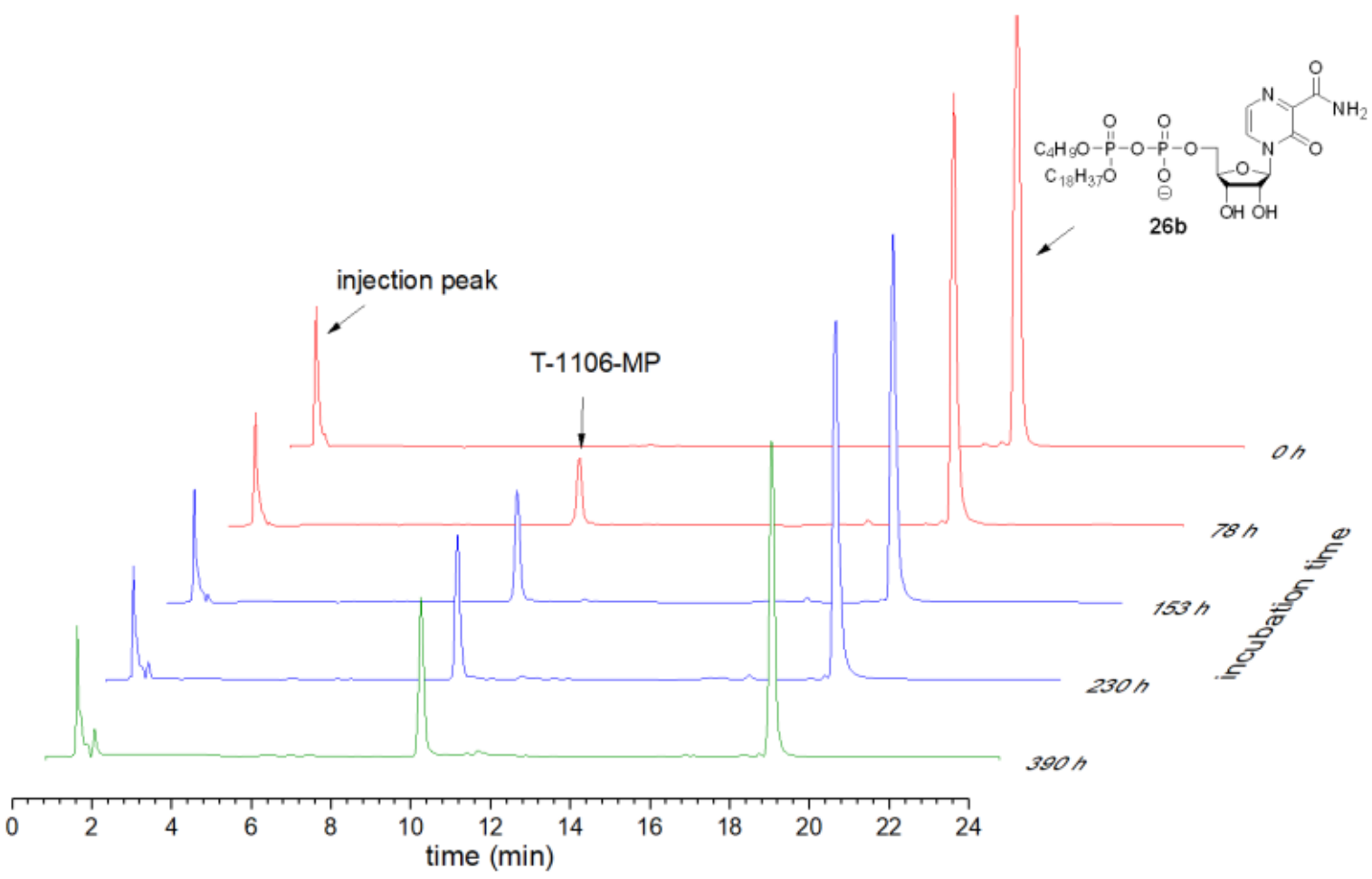

Figure S9. HPLC profile of T-1106-Di*PP*ro-compound **26b** after incubation in PBS.

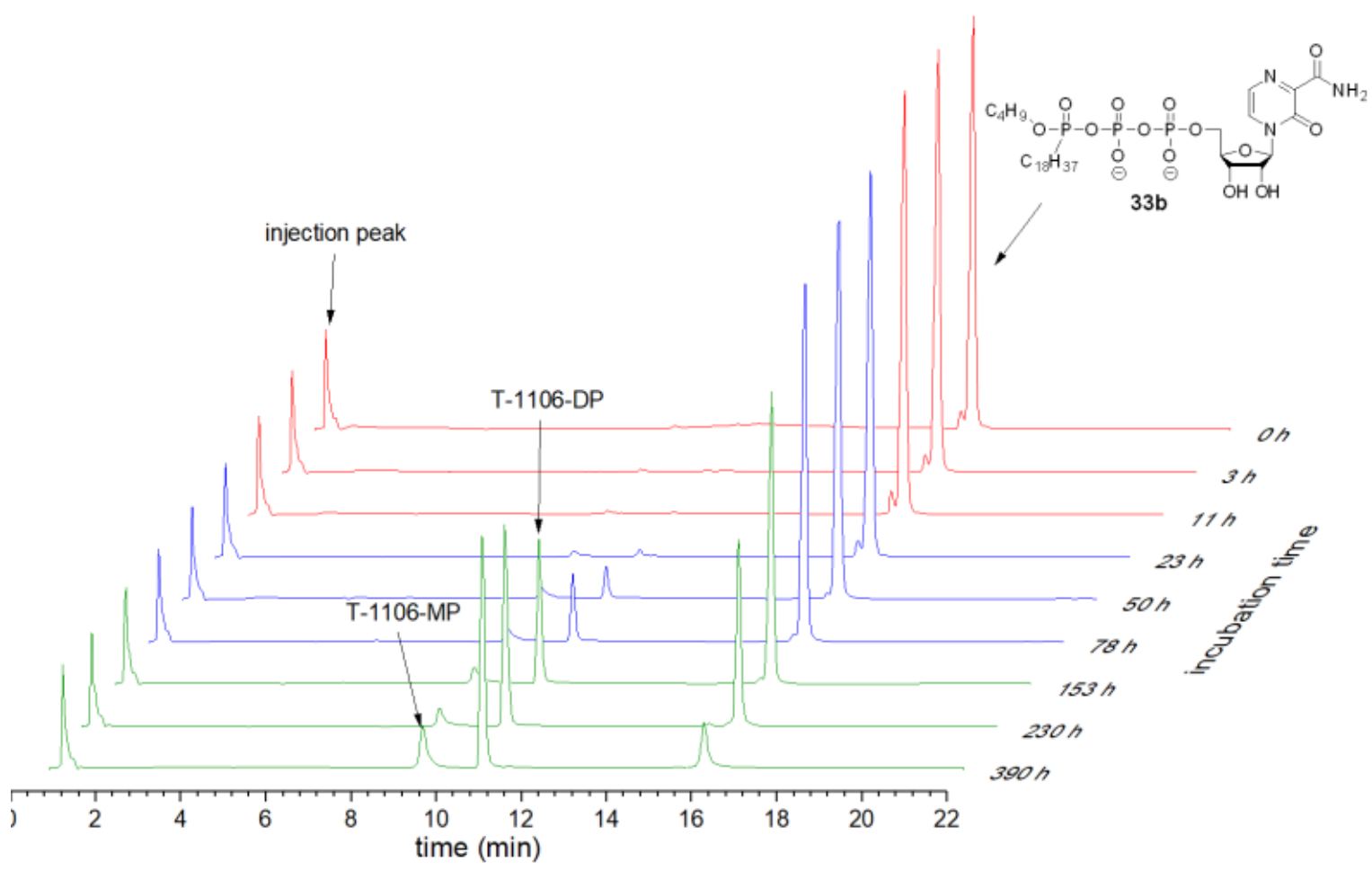

Figure S10. HPLC profile of T-1106-Tri*PP*Pro-compound **33b** after incubation in PBS.

Part 6. Antiviral procedures

We used three influenza virus strains: A/H3N2 (A/HK/7/87), and A/H1N1 (A/Ned/378/05) and B/Ned/537/05, both donated by R. Fouchier, Rotterdam, The Netherlands. Antiviral activity was assessed in Madin Darby canine kidney cells (MDCK; RRID CVCL_0422; kind gift from M. Matrosovich, Marburg, Germany), using established cytopathic effect (CPE) reduction assays.[1] Briefly, low-density cell cultures were infected with virus at 100 $CCID_{50}$ (50% cell culture infective dose) per well, immediately followed by addition of the test compounds. Mock-infected wells were included to assess cytotoxicity. After three days incubation at 35 °C, the CPE was scored by microscopy, then quantified by the colorimetric MTS cell viability assay.

For coronavirus,[2] we used human embryonic lung fibroblast HEL299 cells (RRID CVCL_2480; ATCC No. CCL-137). Confluent cultures were infected with HCoV-229E or HCoV-OC43 at 100 $CCID_{50}$ (50% cell culture infective dose) per well and treated with the compounds. After five days incubation at 35 °C, the CPE scoring and MTS assay were conducted as above. To convert the MTS-absorbance values into % antiviral effect or % cytotoxicity, and calculate the compounds' EC_{50} and CC_{50} values, we used the formulas published elsewhere.[3]

The panel of other RNA viruses and corresponding cell lines included; respiratory syncytial virus (RSV-A strain Long), tested in human Hep2 cells (RRID CVCL_1906; ATCC No. CCL-23); and Sindbis virus, Semliki forest virus, yellow fever virus (vaccine strain 17D) and Zika virus, tested in African Green monkey kidney VeroE6 cells (CVCL_0574; ATCC No. CRL-1586). Evaluation against varicella zoster virus (VZV); herpes simplex virus (HSV)-1 and -2; and HIV-1/2 (CEM/0 cells; CVCL_0207; ATCC No. CCL-1199, was conducted as reported before.[4-7]

Table S1. Antiviral data against human HIV-1/2 in CEM cells

Comp.	HIV-1 (HE)	HIV-2 (ROD)	Toxicity	Selectivity index (SI[c])	CEM/TK⁻ HIV-2 (ROD)	Toxicity	Selectivity index (SI[c])
	EC_{50}[a] [µM]	EC_{50}[a] [µM]	CC_{50}[b] [µM]		EC_{50}[a] [µM]	CC_{50}[d] [µM]	
**22**	>39.6±0	>39.6±0	39.6	<1	>44.7±0	44.7	<1
**23b**	24.3±0	24.3±0	24.3	1	>53.2±0	53.2	<1
**24b**	>100±0	>100±0	>100	>1	>100±0	>100	>1
**24c**	>50.0±0	>50.0±0	50.0	<1	>41.9±0	41.9	<1
**24e**	>100±0	>100±0	>100	>1	>20.0±0	20.00	<1
**24g**	>61.8±0	>61.8±0	61.8	<1	>100±0	>100	>1
**24h**	>44.5±0	>44.5±0	44.5	<1	>16.7±0	16.7	<1
**25**	>25.3±0	>25.3±0	25.3	<1	>16.4±0	16.4	<1
**26b**	>39.7±0	>39.7±0	39.7	<1	>20.0±0	20.0	<1
**27b**	>39.2±0	>39.2±0	39.2	<1	>51.8±0	51.8	<1
**28**	>20.0±0	>20.0±0	20.0	<1	>30.0±0	30.0	<1
**29b**	>45.3±0	>45.3±0	45.3	<1	38.2±17.2	>100	>2
**30b**	>52.5±0	>52.5±0	52.5	<1	>80.5±0	80.5	<1
**30c**	>33.3±0	>33.3±0	33.3	<1	>20.0±0	20.0	<1
**30e**	>44.3±0	>100±0	>100	>1	>70.3±0	70.3	<1
**30g**	>100±0	>100±0	>100	>1	>100±0	>100	>1
**30h**	>13.6±0	>13.6±0	13.6	<1	>20.0±0	20.0	<1
**31**	>27.7±0	>27.7±0	27.7	<1	>20.0±0	20.0	<1
**32b**	>43.0±0	>43.0±0	43.0	<1	>20.0±0	20.0	<1

**33b**	>43.4±0	>43.4±0	43.4	<1	>20.0±0	20.0	<1
**40d**	>100±0	>100±0	>100	>1	>100±0	>100	>1
**41d**	>100±0	>100±0	>100	>1	>50.0±0	50.0	<1
**47**	>37.5±0	>37.5±0	37.5	<1	>20.0±0	20.0	<1
**48**	>16.3±0	>16.3±0	16.3	<1	>30.0±0	30.0	<1
**T-1106**	>100±0	>100±0	>100	>1	>100±0	>100	>1

[a]Antiviral activity determined in $CD4^+$ T-lymphocytes: 50% effective concentration; values are the mean ±SD of n=2-3 independent experiments. [b]Cytotoxicity: 50% cytostatic concentration or compound concentration required to inhibit $CD4^+$ T-cell (CEM) proliferation by 50%; values are the mean ±SD of n=2-3 independent experiments. [c]Selectivity index (SI) was calculated as the ratio of the CC_{50} [μM] and the EC_{50} [μM], HIV-2 (ROD) in CEM cells. [d]Cytotoxicity: 50% cytostatic concentration or compound concentration required to inhibit $CD4^+$ T-cell (CEM/TK^-) proliferation by 50%; values are the mean ± SD of n = 2−3 independent experiments. [e]Selectivity index (SI) was calculated as the ratio of the CC_{50} [μM] and the EC_{50} [μM], HIV-2 (ROD) in CEM/TK^- cells.

Part 7. Primer-extension assays

Human polymerases α,β,γ and HIV-RT were obtained from Chimerx and Roboklon. The fluorescent labeled primer and template were bought from Metabion. Gel size was adjusted to the electrophoresis chamber (450mm×200mm×1.0mm)

Primer sequence for the Cy3-fluorescent labeled primer extension experiment:

25nt Primer-sequence: 5´-Cy3-CGTTG GTCCT GAAGG AGGAT AGGTT-3´

30nt Template-sequence: 3´-GCAAC CAGGA CTTCC TCCTA TCCAA AGACA-5´

Human DNA polymerase α assay conditions: 60 mM Tris-HCl (pH 8), 5 mM $Mg(OAc)_2$, 100 mM KCl, 1.0 mM dithiothreiol, 0.1 mM spermine, 0.01% (w/v) bovine serum albumin, 250 μM dNTPs, 2U human polymerase α, 0.20 μM DNA hybrid, primer-template-hybrid 0.32 mM in reaction volume of 10 μL, incubated at 37 °C for 1 h, 80 °C for 7 min. Primer extension assays: 50 mA, 45 w for 3 h.

HIV-RT assay reaction conditions: 50 mM Tris-HCl (pH 8.6), 10 mM $MgCl_2$, 40 mM KCl, 250 μM dNTPs, HIV-RT (6 U), 0.20 μM DNA hybrid, primer-template-hybrid 0.32 mM in reaction volume of 10 μL, incubated at 37 °C for 30 min, 80 °C for 7 min. Primer extension assays: 50 mA, 45 w for 4 h.

Human DNA polymerase α assay conditions: 60mM Tris-HCl (pH 8), 5 mM $Mg(OAc)_2$, 100 mM KCl, 1.0 mM dithiothreiol, 0.1 mM spermine, 0.01% (w/v) bovine serum albumin, 250 μM dNTPs, 2 U human polymerase α, 0.20 μM DNA hybrid, primer-template-hybrid 0.32 mM in reaction volume of 10 μL, incubated at 37 °C for 1 h, 80 °C for 7 min. Primer extension assays: 50 mA, 45 w for 3 h.

Human DNA polymerase β assay conditions: 50mM Tris-HCl (pH 8.7), 10 mM $Mg(OAc)_2$, 100 mM KCl, 1.0 mM dithiothreitol, 0.1 mM spermine, 0.01% (w/v) bovine serum albumin, 15% glycerol, 250 μM dNTPs, 2 U human DNA polymerase β, 0.20 μM DNA hybrid, primer-template-hybrid 0.32 mM in reaction volume of 10 μL, incubated at 37 °C for 1 h, 80 °C for 7 min. Primer extension assays: 50 mA, 45 w for 3 h.

Human polymerase γ assay conditions: 60 mM Tris-HCl (pH 8), 5 mM $Mg(OAc)_2$, 1.0 mM dithiothreiol, 0.1mM spermine, 0.01% (w/v) bovine serum albumin, 250 μM dNTPs, 2 U human polymerase γ, 0.5 μM $MnCl_2$, 0.20 μM DNA hybrid, primer-template-hybrid 0.32 mM in reaction volume of 10 μL, incubated at 37 °C for 2 h, 80 °C for 7 min. Primer extension assays: 50 mA, 45 w for 3 h.

Figure S11. Primer extension assay using HIV-RT and polymerases α,β,γ.

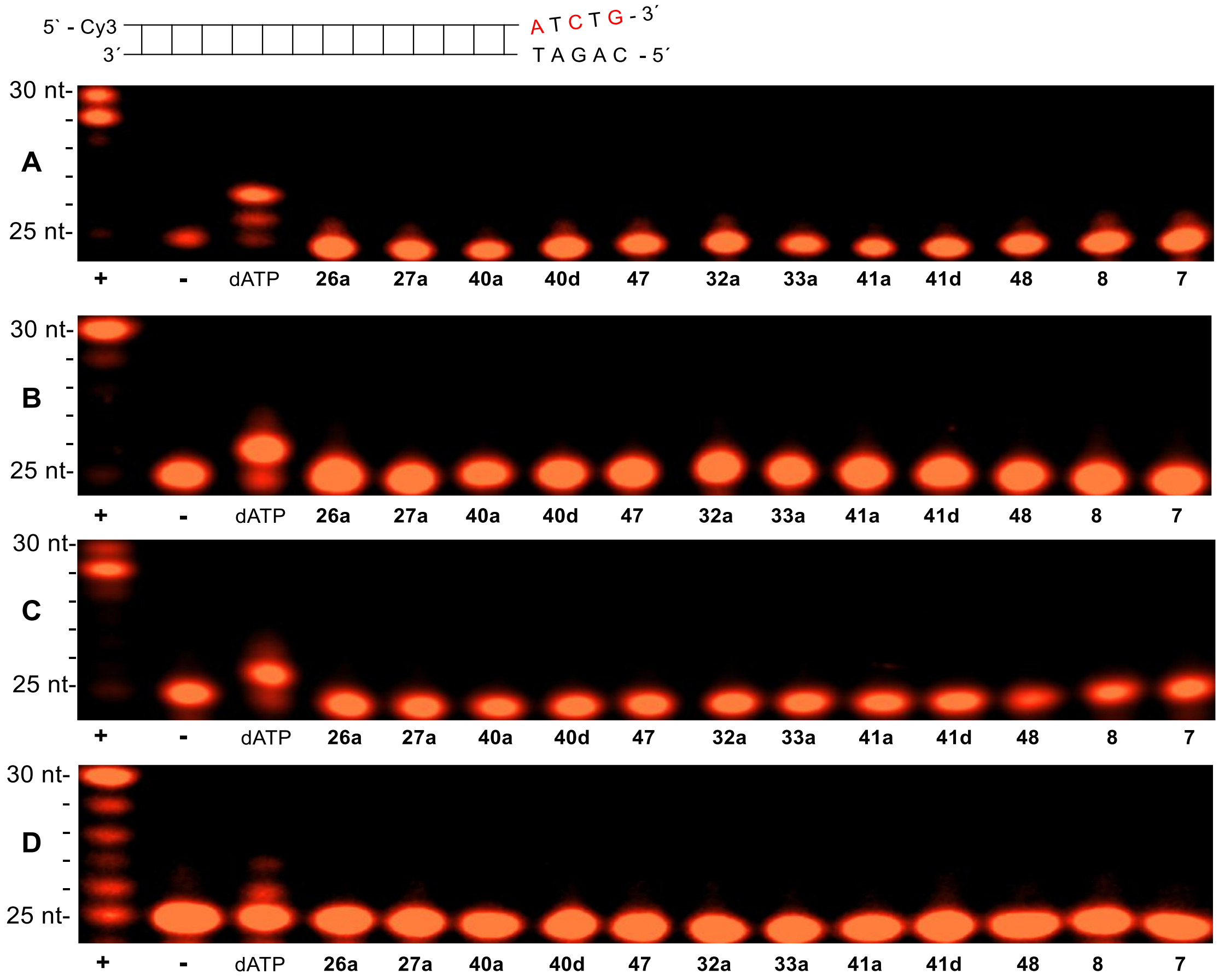

Figure S11. Primer extension assay using HIV-RT (**A**, 30 min, 6U) and polymerases *α* (**B**, 60 min, 2U), *β* (**C**, 60 min, 2U), and *γ* (**D**, 120 min, 2U). Lane 1: (+) dATP, dCTP, dGTP and TTP with HIV-RT (**A**) or polymerases *α* (**B**) or *β* (**C**) or *γ* (**D**). Lane 2: (-) dATP, dCTP, dGTP and TTP without HIV-RT (**A**) or polymerases *α* (**B**) or *β* (**C**) or *γ* (**D**). Lane 3: dATP. Lane 4: *β*-(C1;C18)-T-1106-DP **26a**. Lane 5: *β*-(C1;C18)-phosphonate-T-1106-MP **27a**. Lane 6: *β*-(C1)-T-1106-DP **40a**. Lane 7: *β*-(C18)-T-1106-DP **40d**. Lane 8: *β*-C-(C18)-T-1106-DP **47**. Lane 9: *γ*-(C1;C18)-T-1106-TP **32a**. Lane 10: *γ*-(C1;C18)-phosphonate-T-1106-DP **33a**. Lane 11: *γ*-(C1)-T-1106-TP **41a**. Lane 12: *γ*-(C18)-T-1106-TP **41d**. Lane 13: *γ*-C-(C18)-T-1106-TP **48**. Lane 14: T-1106-TP **8**. Lane 14: T-1106-DP **7**.

Part 8. Spectral data for new compounds

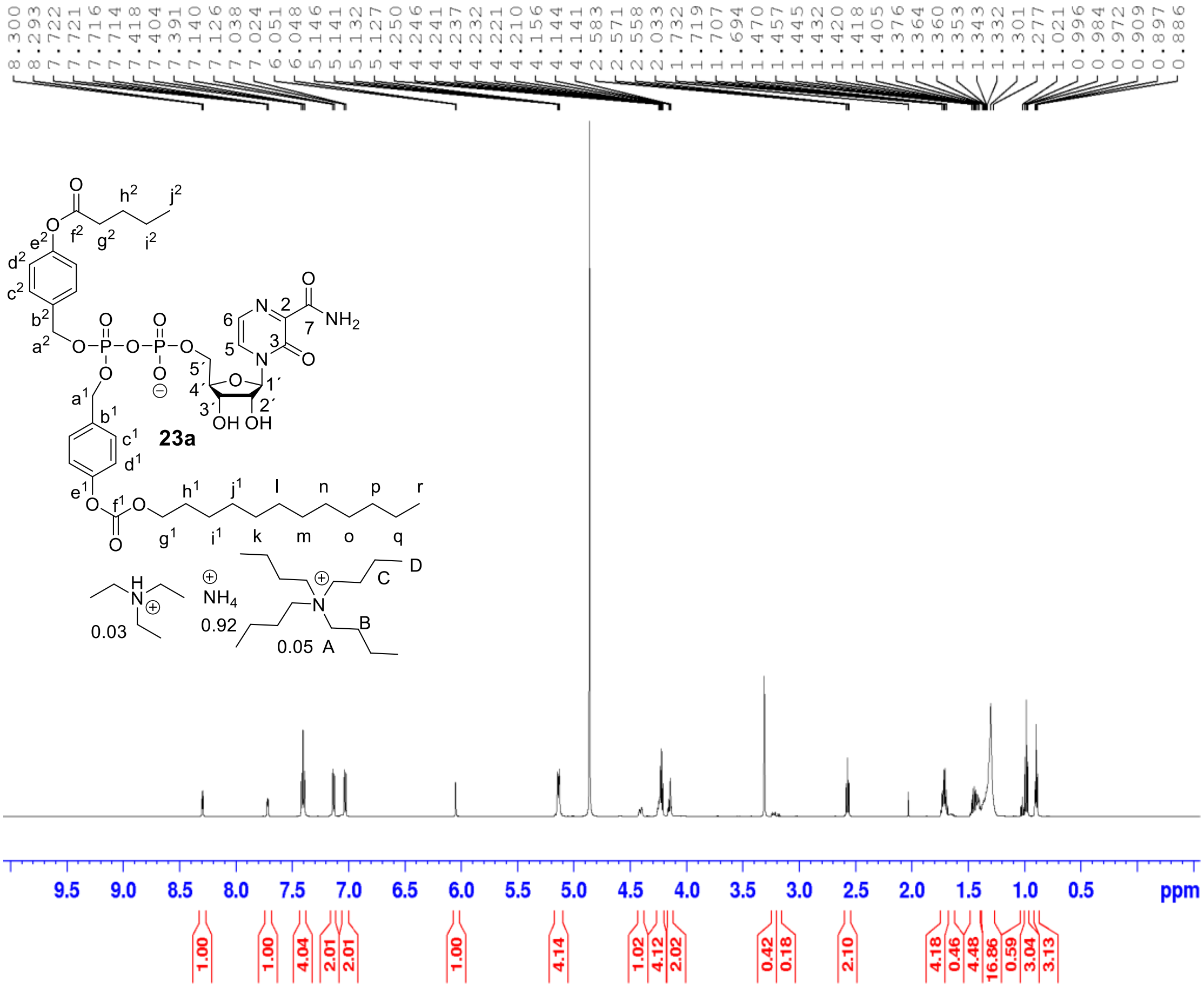

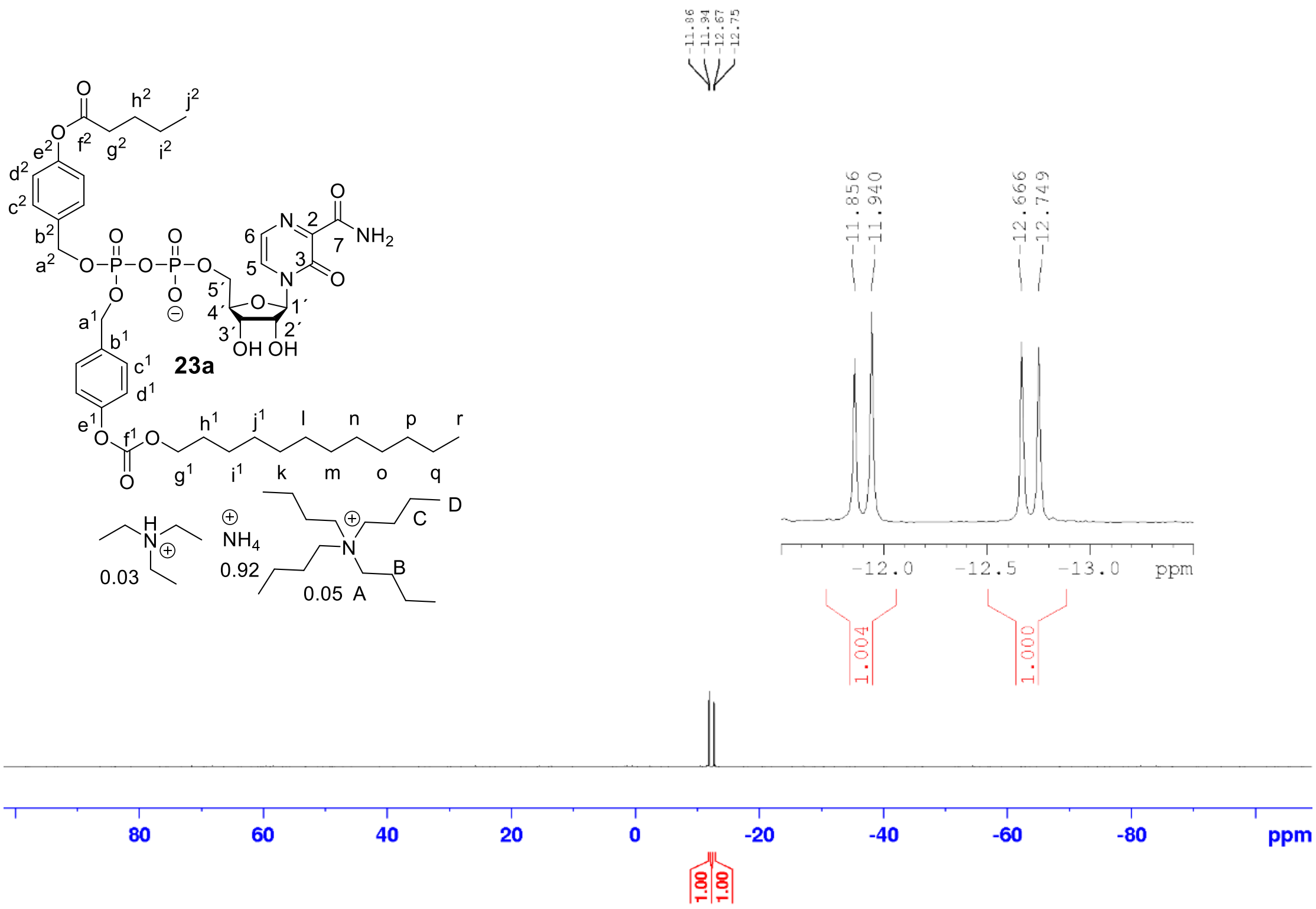

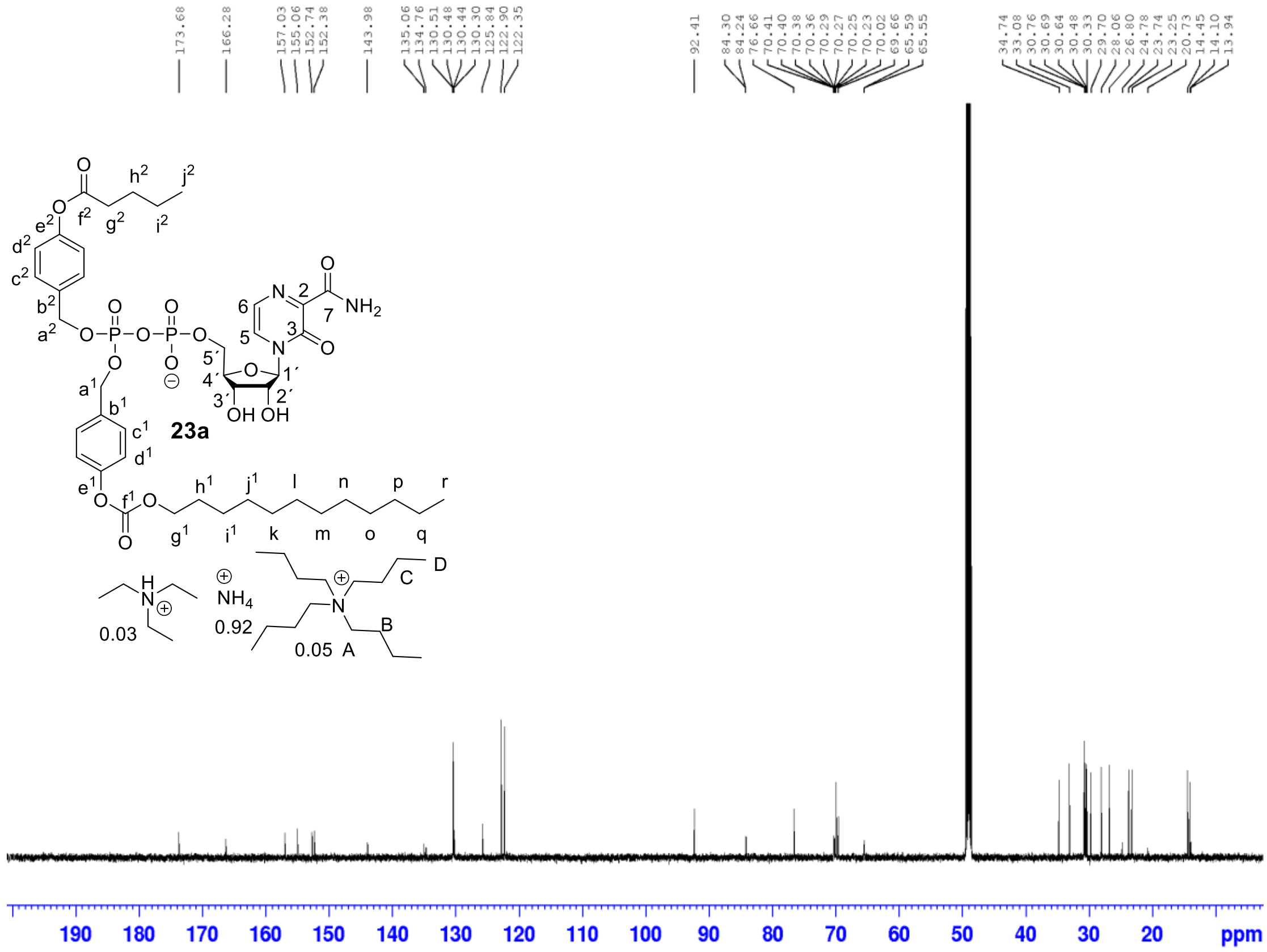
173.68
166.28
157.03
155.06
152.74
152.38
143.98
135.06
134.76
130.51
130.48
130.44
130.30
125.84
122.90
122.35
92.41
84.30
84.24
76.66
70.41
70.40
70.38
70.36
70.29
70.27
70.25
70.23
70.02
69.66
65.59
65.55
34.74
33.08
30.76
30.69
30.64
30.48
30.33
29.70
28.06
26.80
24.78
23.74
23.25
20.73
14.45
14.10
13.94
23a
0.03
NH_4
0.92
0.05 A
B
C
D
ppm

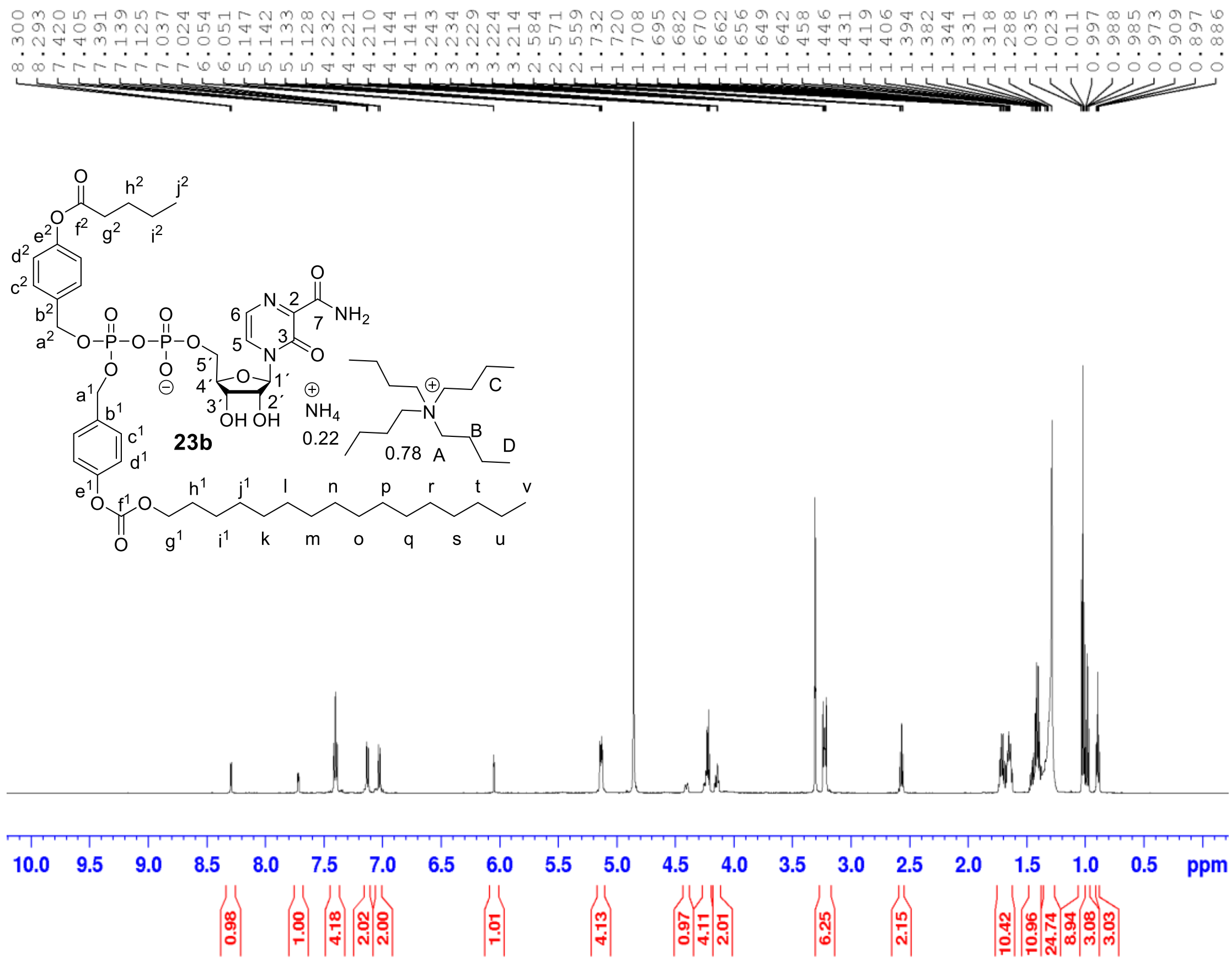
8.300
8.293
7.420
7.405
7.391
7.139
7.125
7.037
7.024
6.054
6.051
5.147
5.142
5.133
5.128
4.232
4.221
4.210
4.144
4.141
3.243
3.234
3.229
3.224
3.214
2.584
2.571
2.559
1.732
1.720
1.708
1.695
1.682
1.670
1.662
1.656
1.649
1.642
1.458
1.446
1.431
1.419
1.406
1.394
1.382
1.344
1.331
1.318
1.288
1.035
1.023
1.011
0.997
0.988
0.985
0.973
0.909
0.897
0.886
23b
NH_4
0.22
0.78 A
B
C
D
ppm
0.98
1.00
4.18
2.02
2.00
1.01
4.13
0.97
4.11
2.01
6.25
2.15
10.42
10.96
24.74
8.94
3.08
3.03

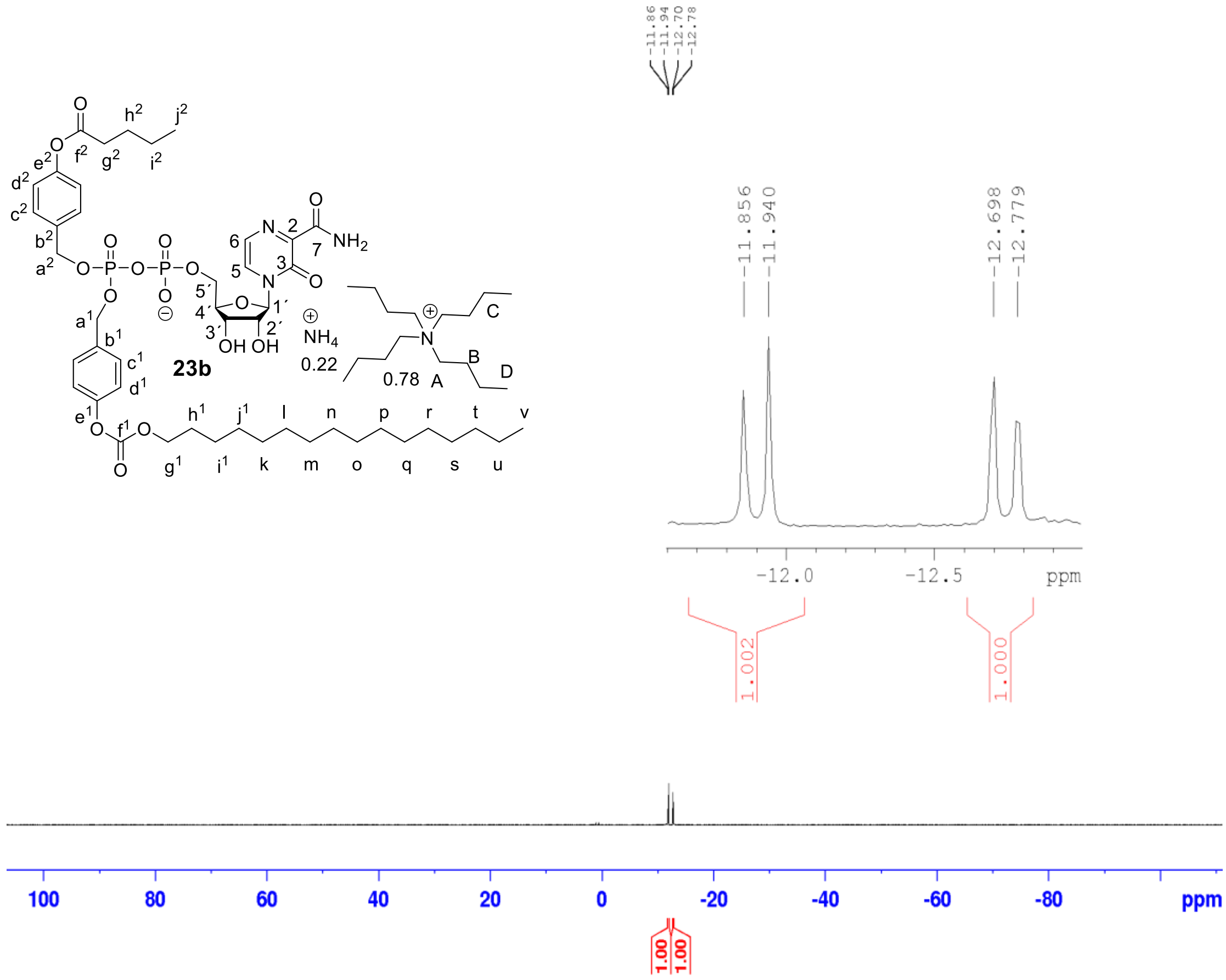
23b
0.22
0.78
NH4
-11.86
-11.94
-12.70
-12.78
-11.856
-11.940
-12.698
-12.779
-12.0
-12.5
ppm
1.002
1.000
100
80
60
40
20
0
-20
-40
-60
-80
ppm
1.00
1.00

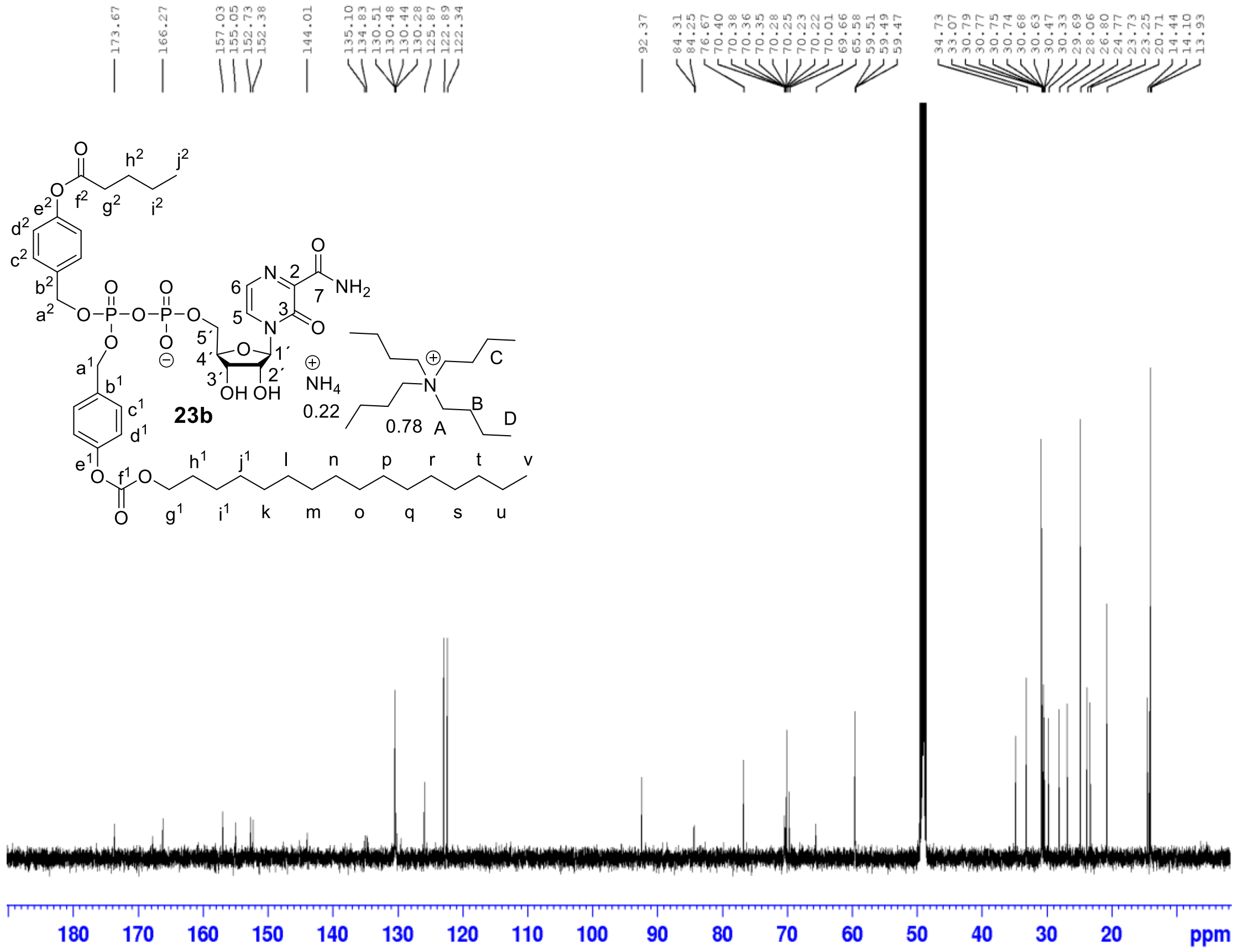
23b
0.22
0.78
NH4
173.67
166.27
157.03
155.05
152.73
152.38
144.01
135.10
134.83
130.51
130.48
130.44
130.28
125.87
122.89
122.34
92.37
84.31
84.25
76.67
70.40
70.38
70.36
70.35
70.28
70.25
70.23
70.22
70.01
69.66
65.58
59.51
59.49
59.47
34.73
33.07
30.79
30.77
30.75
30.74
30.68
30.63
30.47
30.33
29.69
28.06
26.80
24.77
23.73
23.25
20.71
14.44
14.10
13.93
180
170
160
150
140
130
120
110
100
90
80
70
60
50
40
30
20
ppm

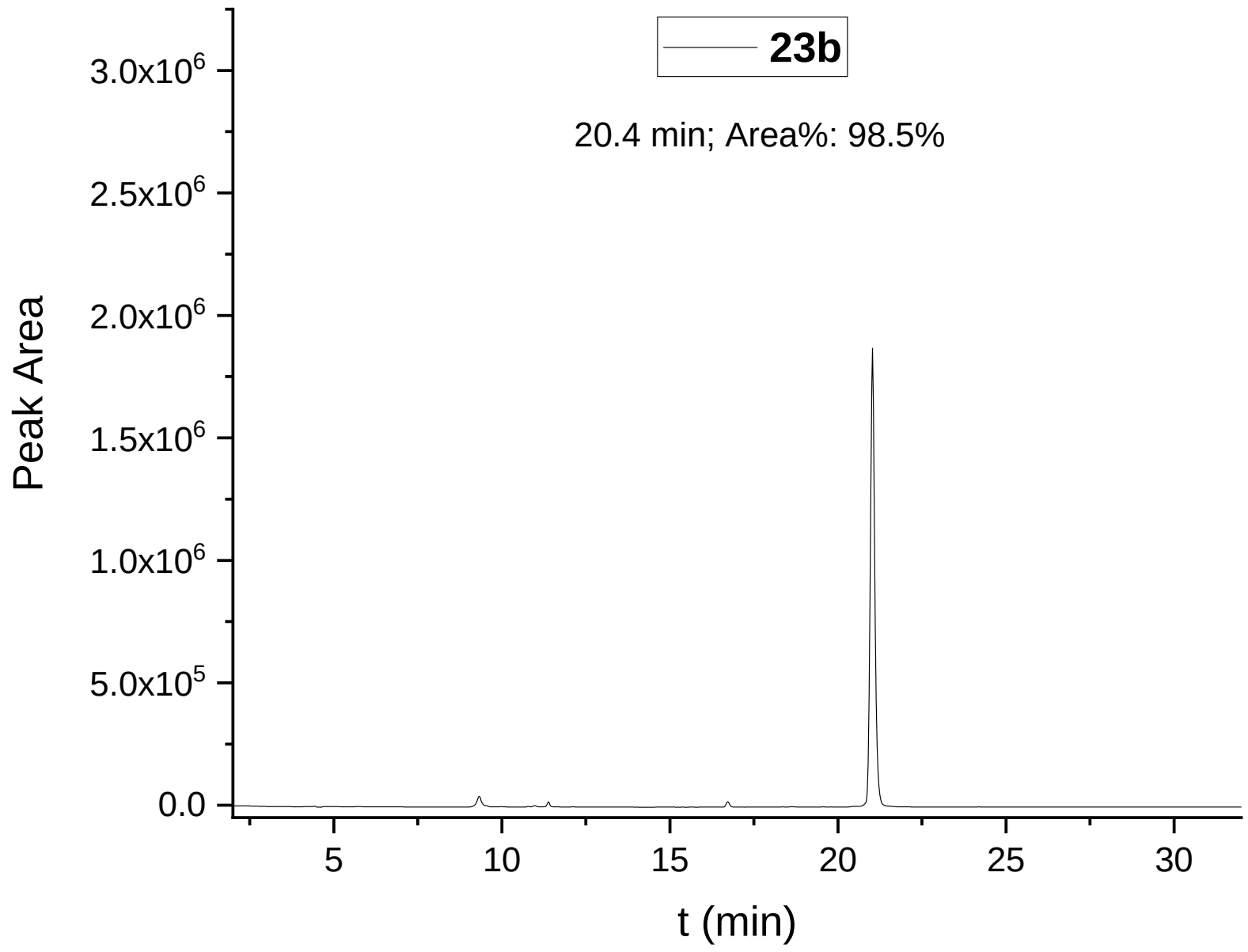
23b
20.4 min; Area%: 98.5%
Peak Area
t (min)

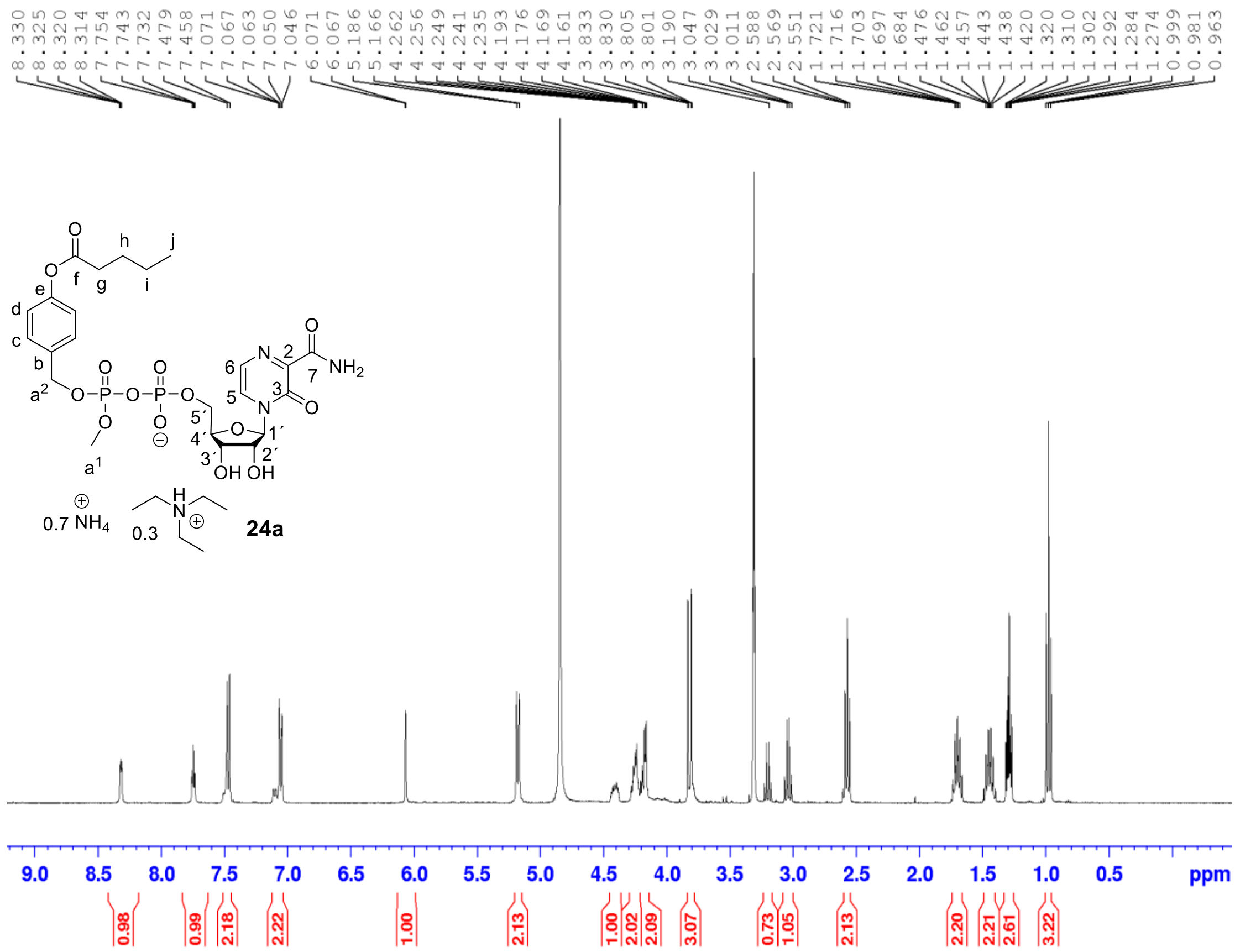
0.7 NH4
0.3
24a
ppm

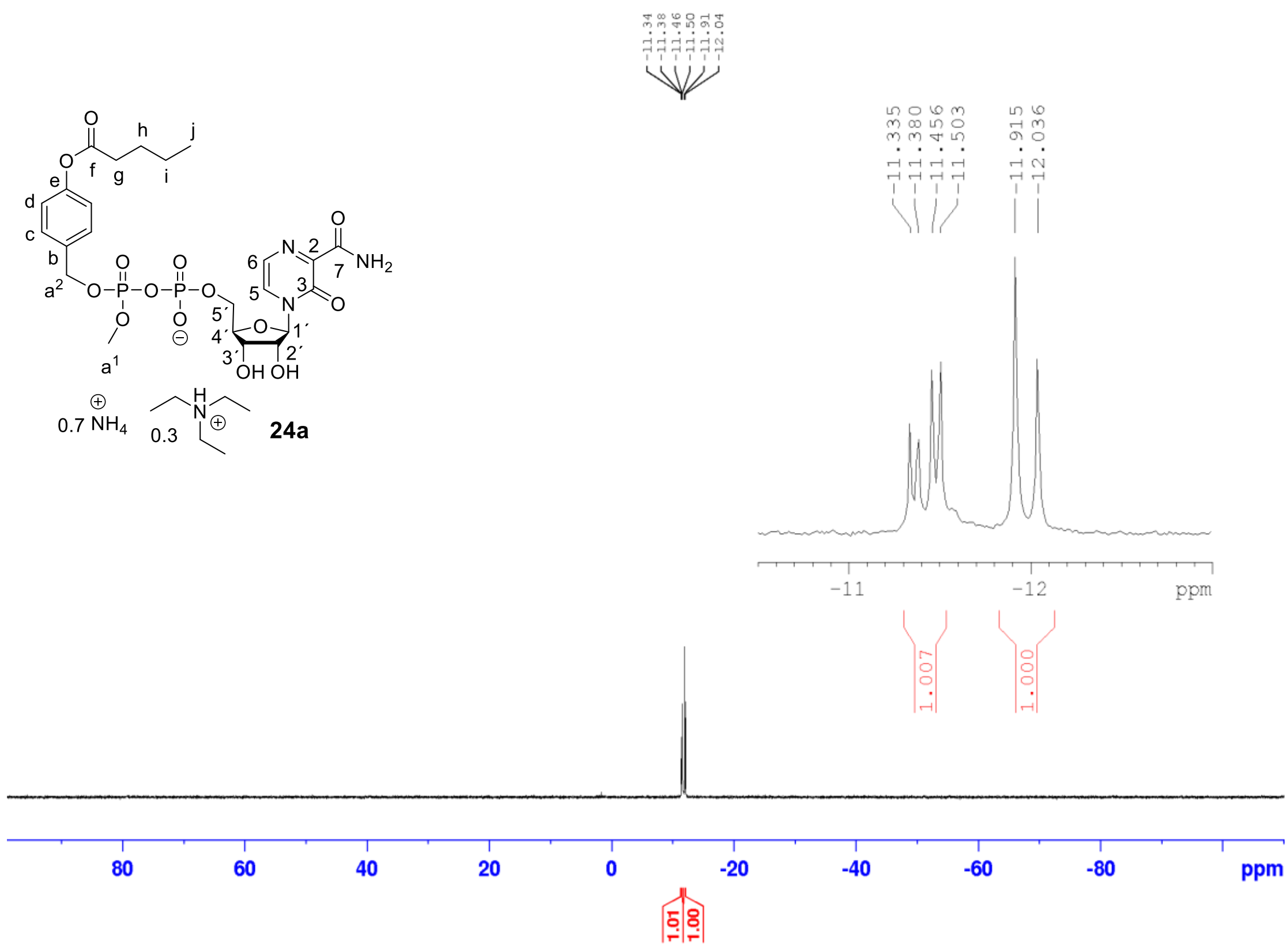
24a
0.7 NH4
0.3
-11.34
-11.38
-11.46
-11.50
-11.91
-12.04
-11.335
-11.380
-11.456
-11.503
-11.915
-12.036
1.007
1.000
1.01
1.00
ppm

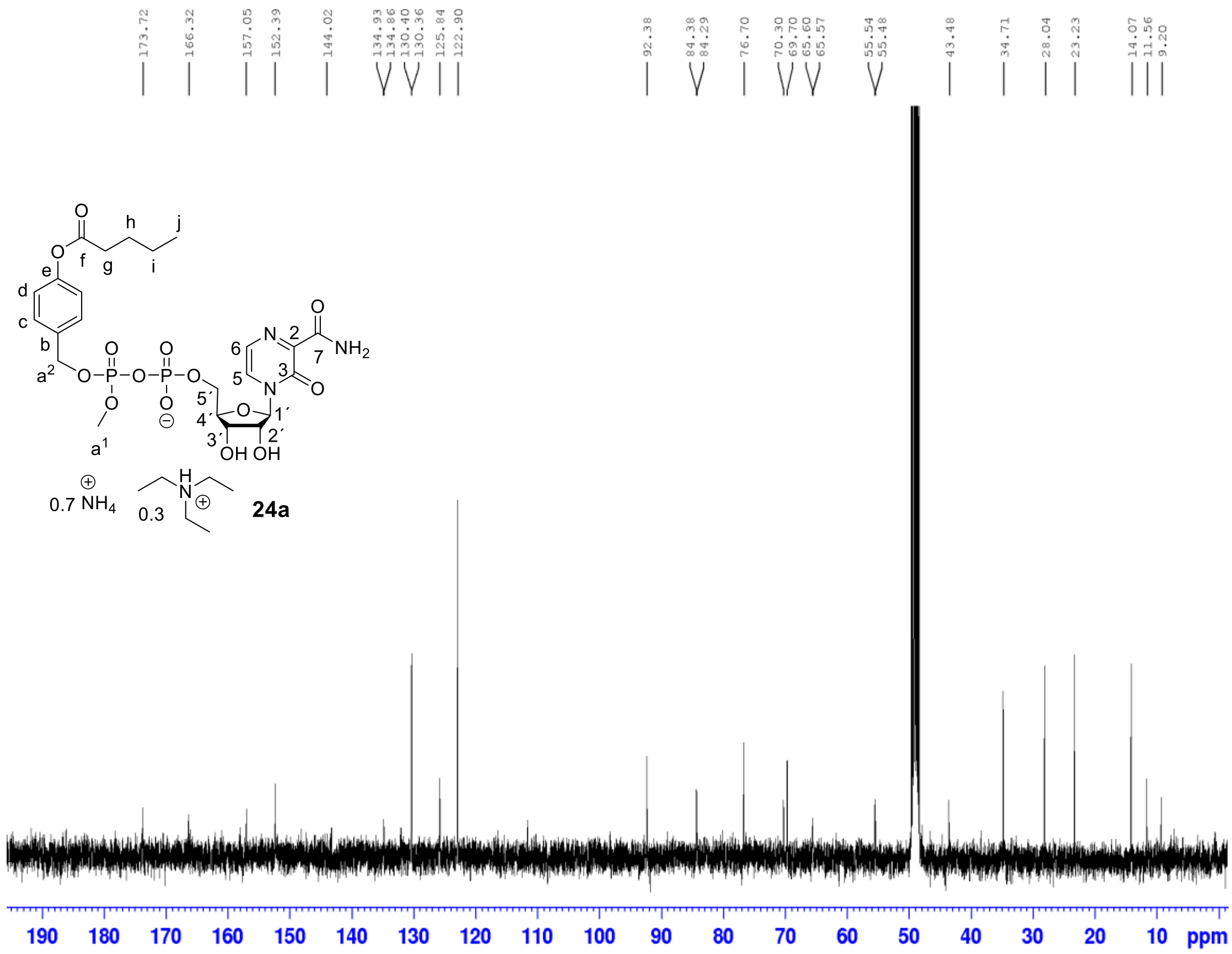
24a
0.7 NH4
0.3
173.72
166.32
157.05
152.39
144.02
134.93
134.86
130.40
130.36
125.84
122.90
92.38
84.38
84.29
76.70
70.30
69.70
65.60
65.57
55.54
55.48
43.48
34.71
28.04
23.23
14.07
11.56
9.20
ppm

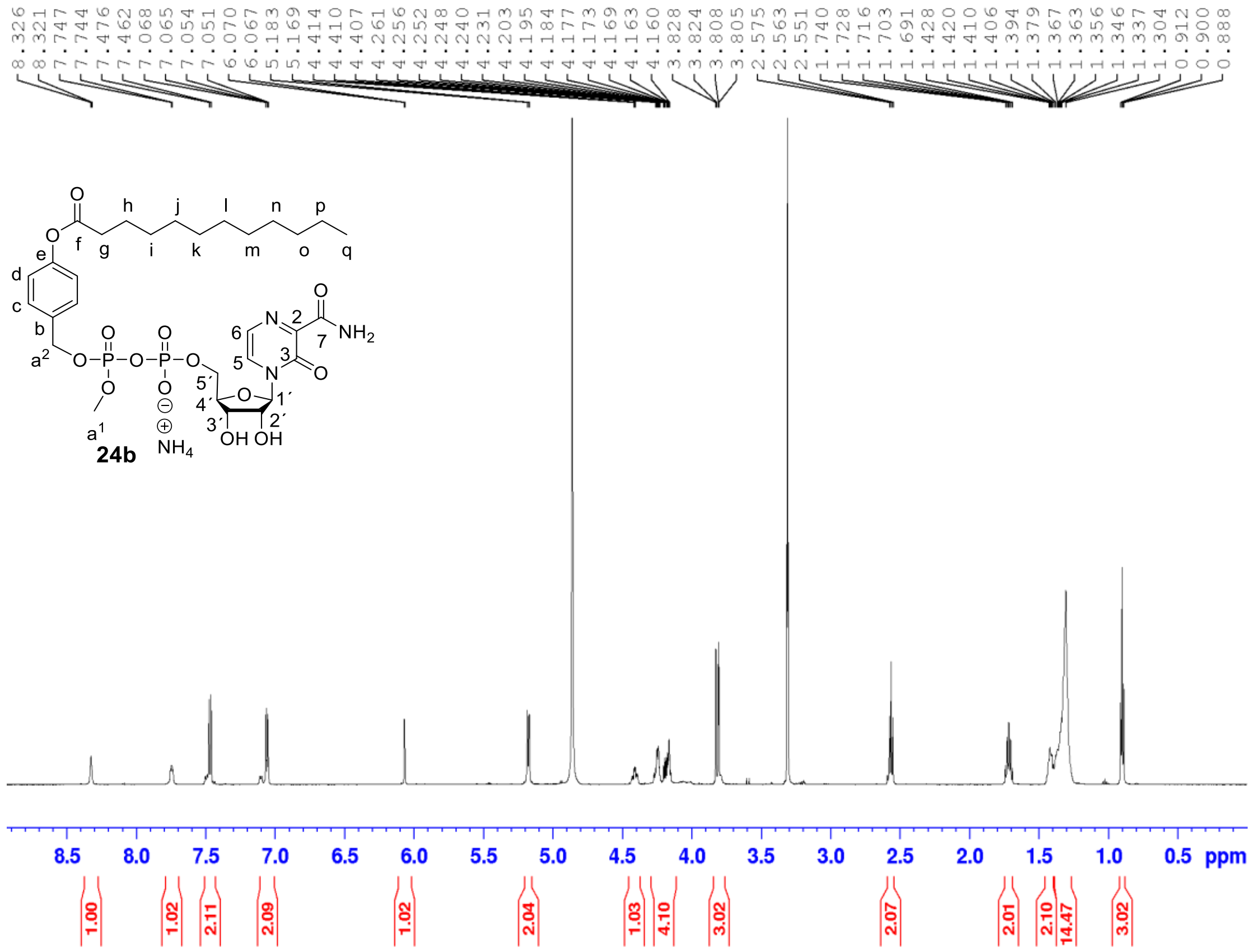
24b
8.5 8.0 7.5 7.0 6.5 6.0 5.5 5.0 4.5 4.0 3.5 3.0 2.5 2.0 1.5 1.0 0.5 ppm
1.00 1.02 2.11 2.09 1.02 2.04 1.03 4.10 3.02 2.07 2.01 2.10 14.47 3.02

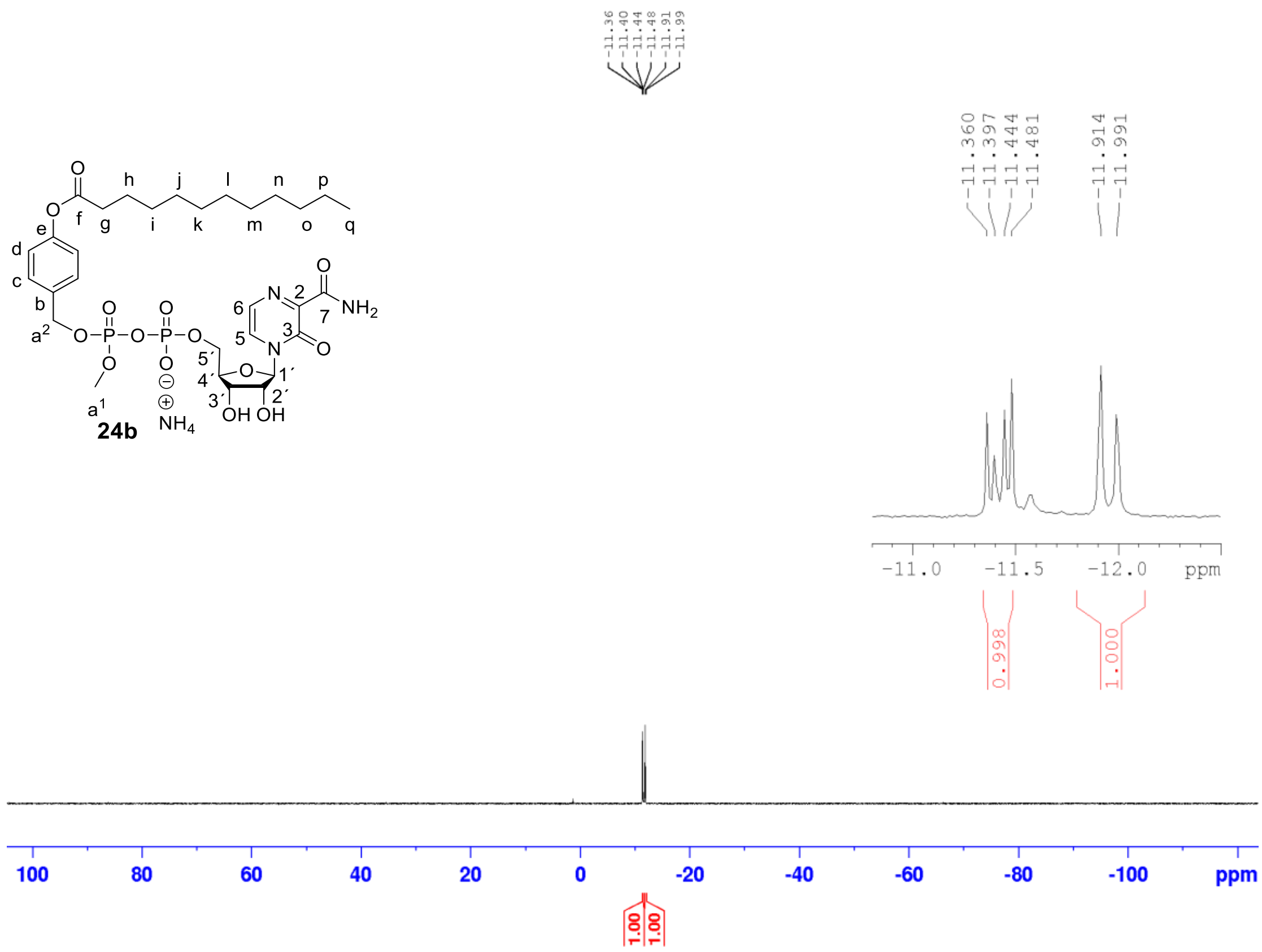
24b
-11.0 -11.5 -12.0 ppm
0.998 1.000
100 80 60 40 20 0 -20 -40 -60 -80 -100 ppm
1.00 1.00

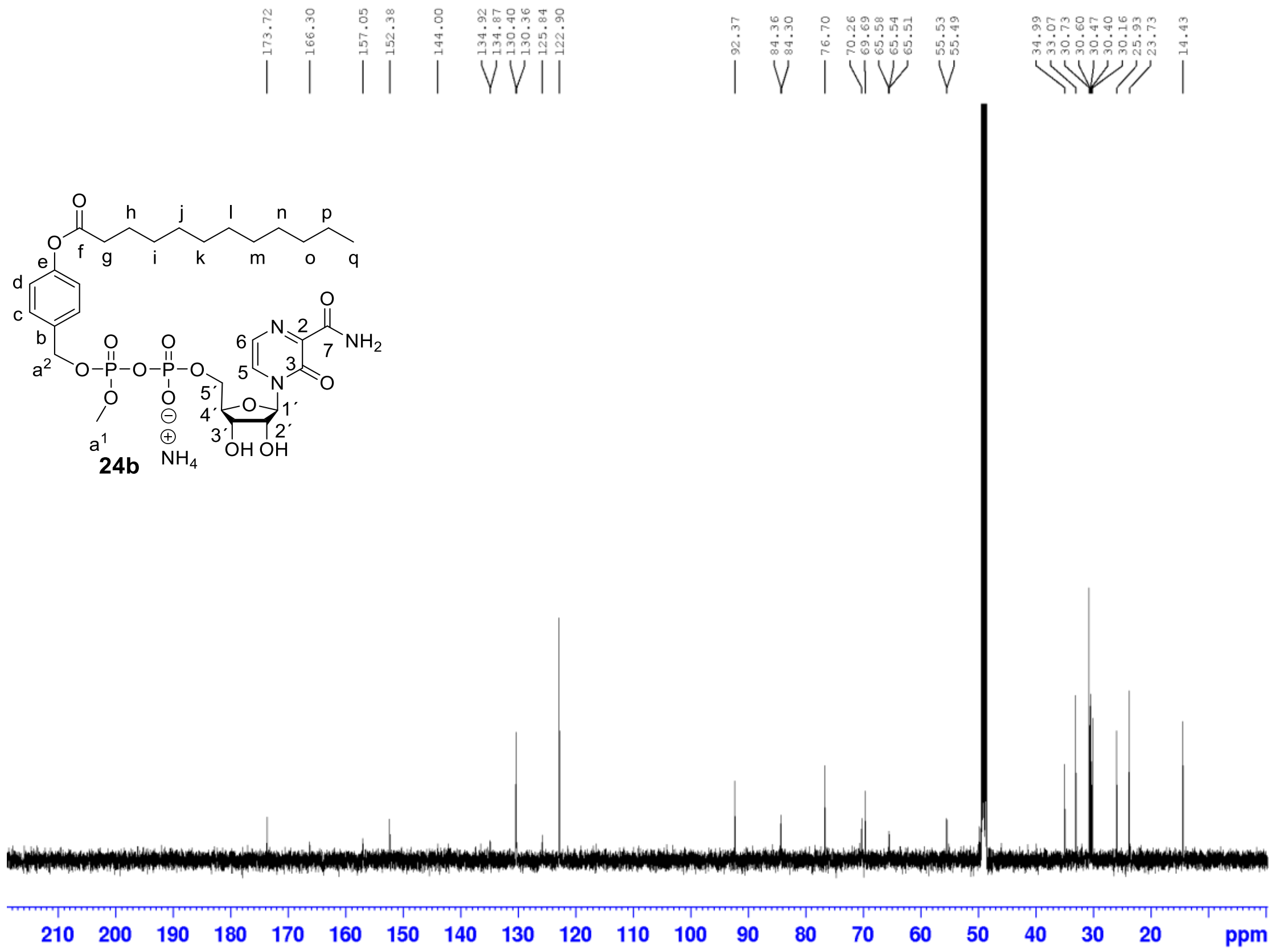
173.72
166.30
157.05
152.38
144.00
134.92
134.87
130.40
130.36
125.84
122.90
92.37
84.36
84.30
76.70
70.26
69.69
65.58
65.54
65.51
55.53
55.49
34.99
33.07
30.73
30.60
30.47
30.40
30.16
25.93
23.73
14.43
24b
210 200 190 180 170 160 150 140 130 120 110 100 90 80 70 60 50 40 30 20 ppm

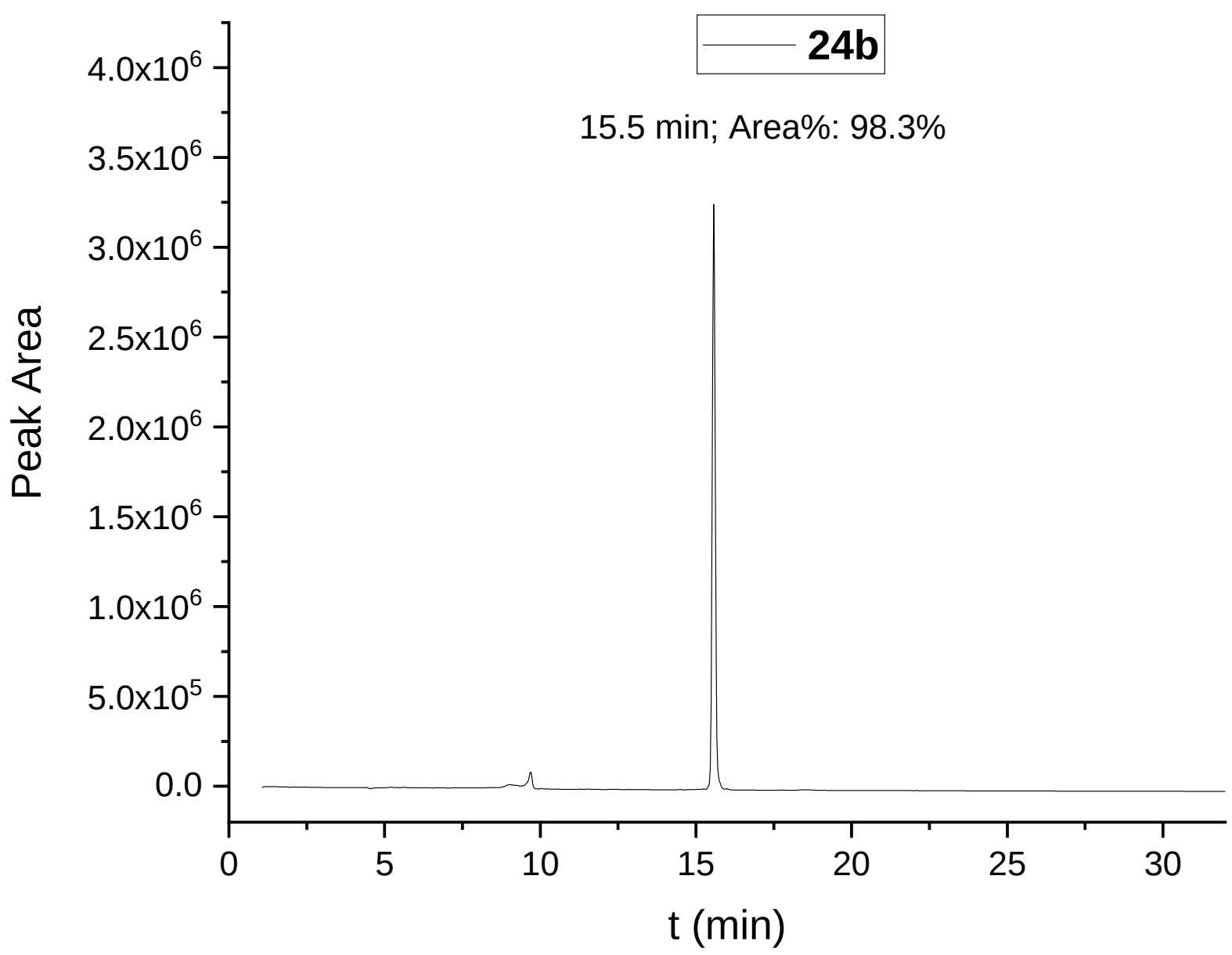
24b
15.5 min; Area%: 98.3%
Peak Area
t (min)

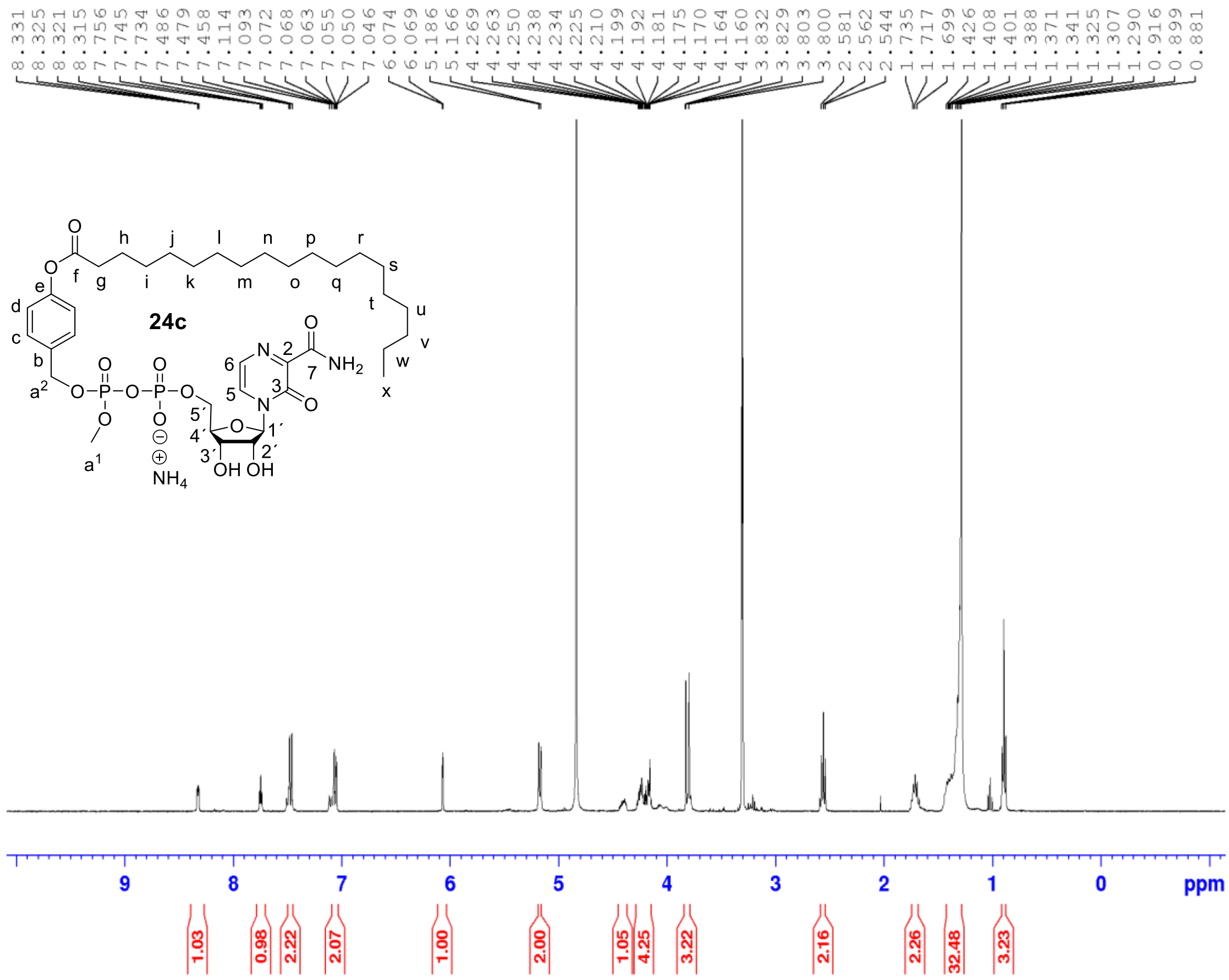

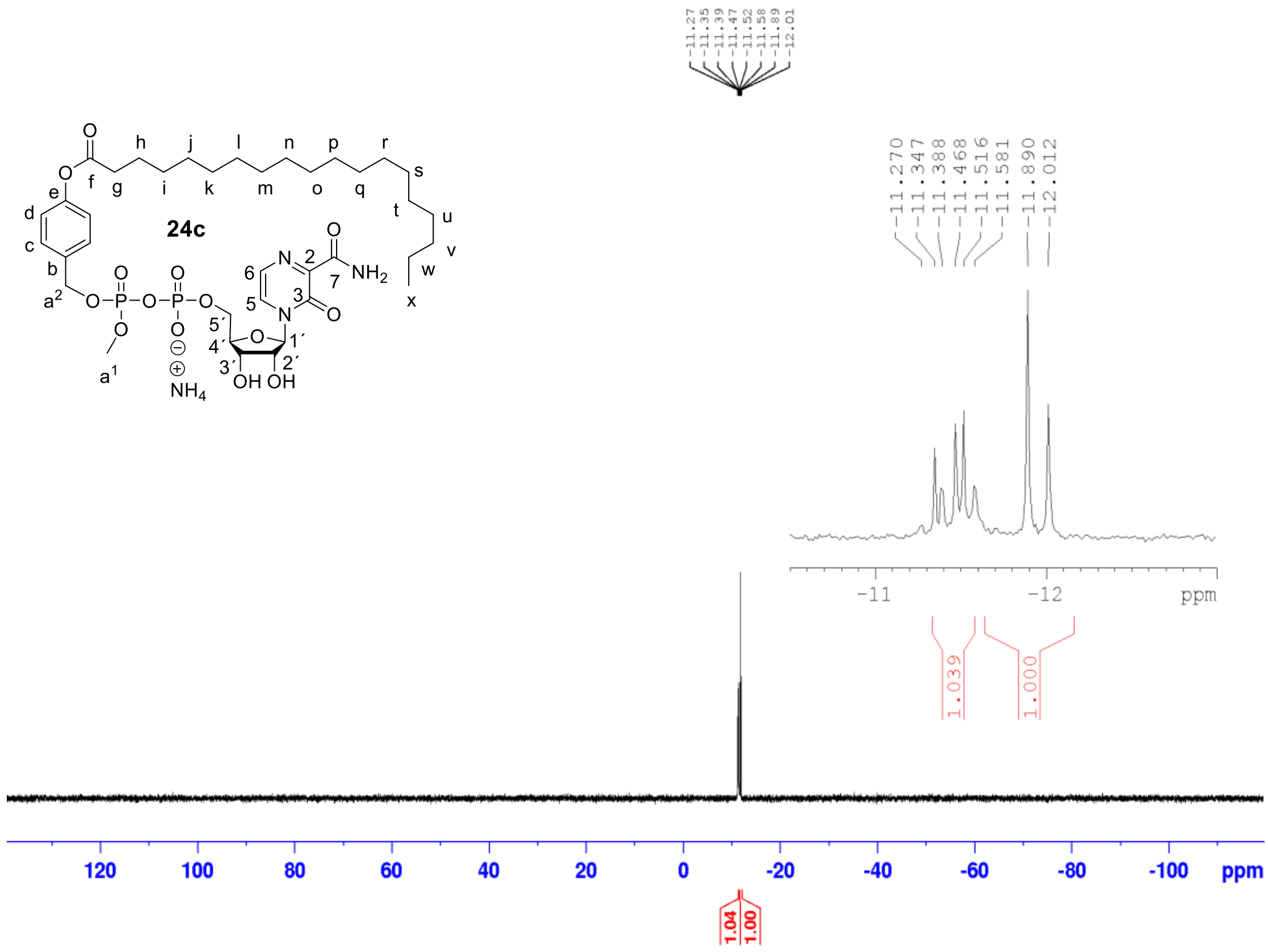

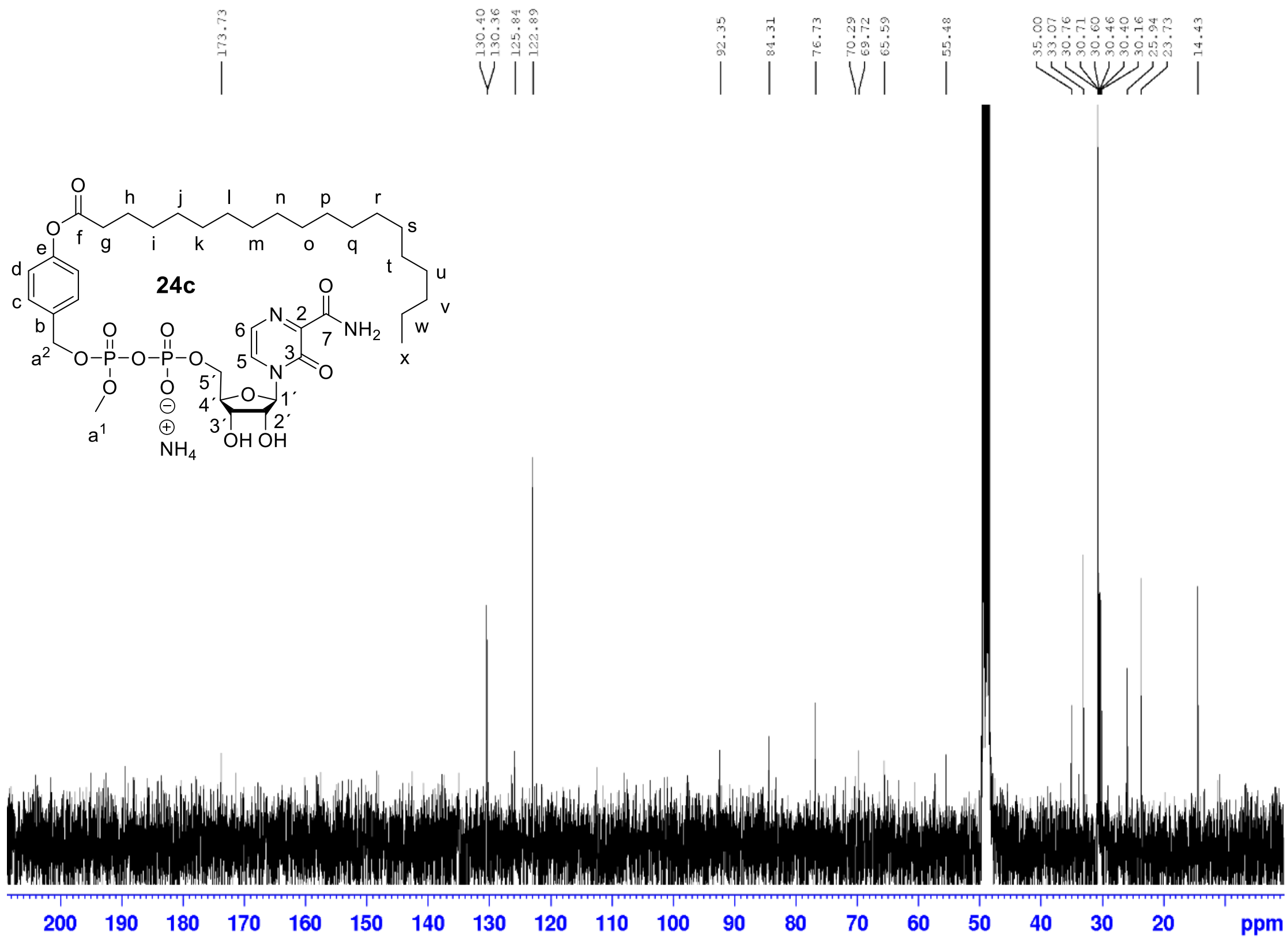
173.73
130.40
130.36
125.84
122.89
92.35
84.31
76.73
70.29
69.72
65.59
55.48
35.00
33.07
30.76
30.71
30.60
30.46
30.40
30.16
25.94
23.73
14.43
24c
ppm

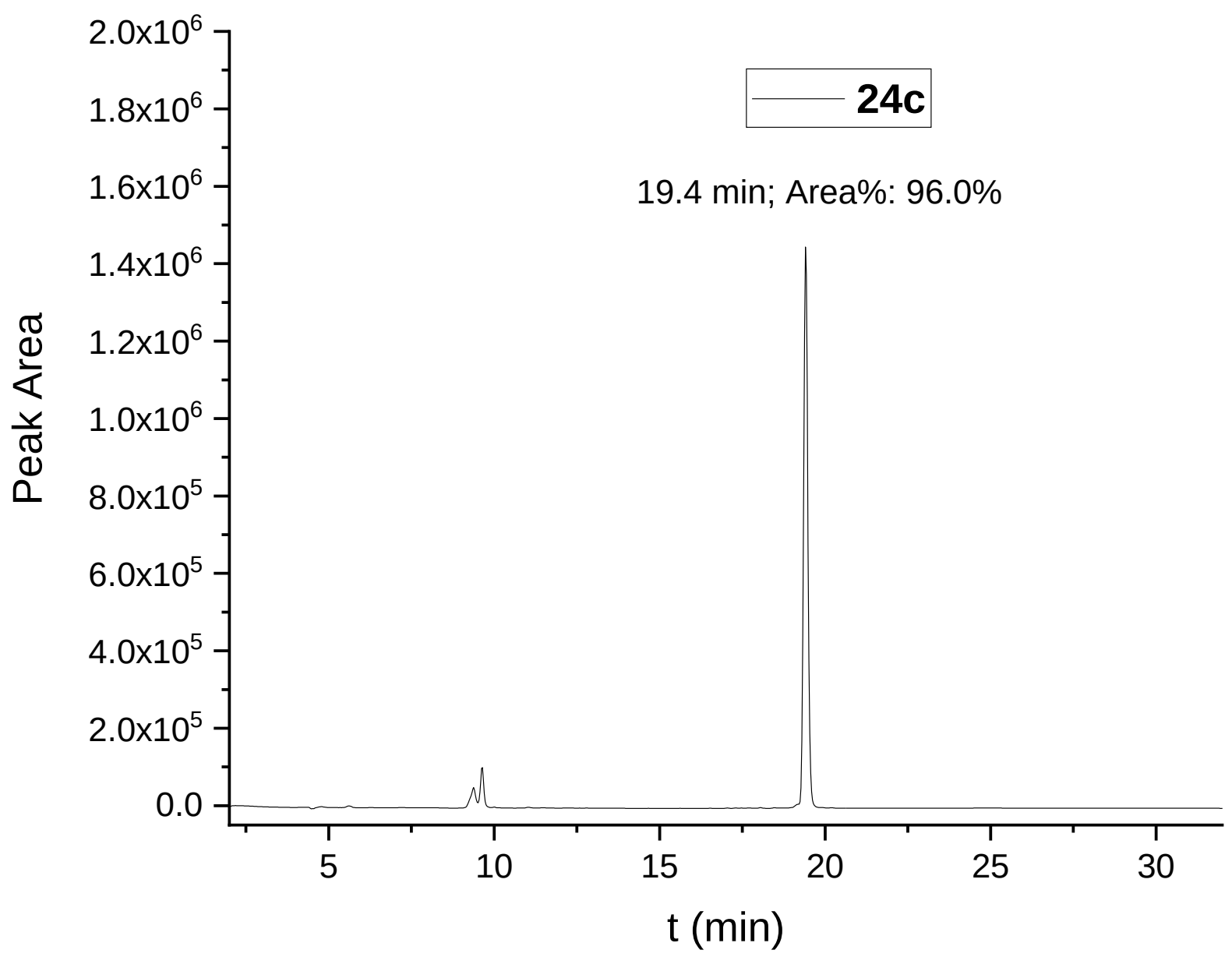
24c
19.4 min; Area%: 96.0%
Peak Area
t (min)

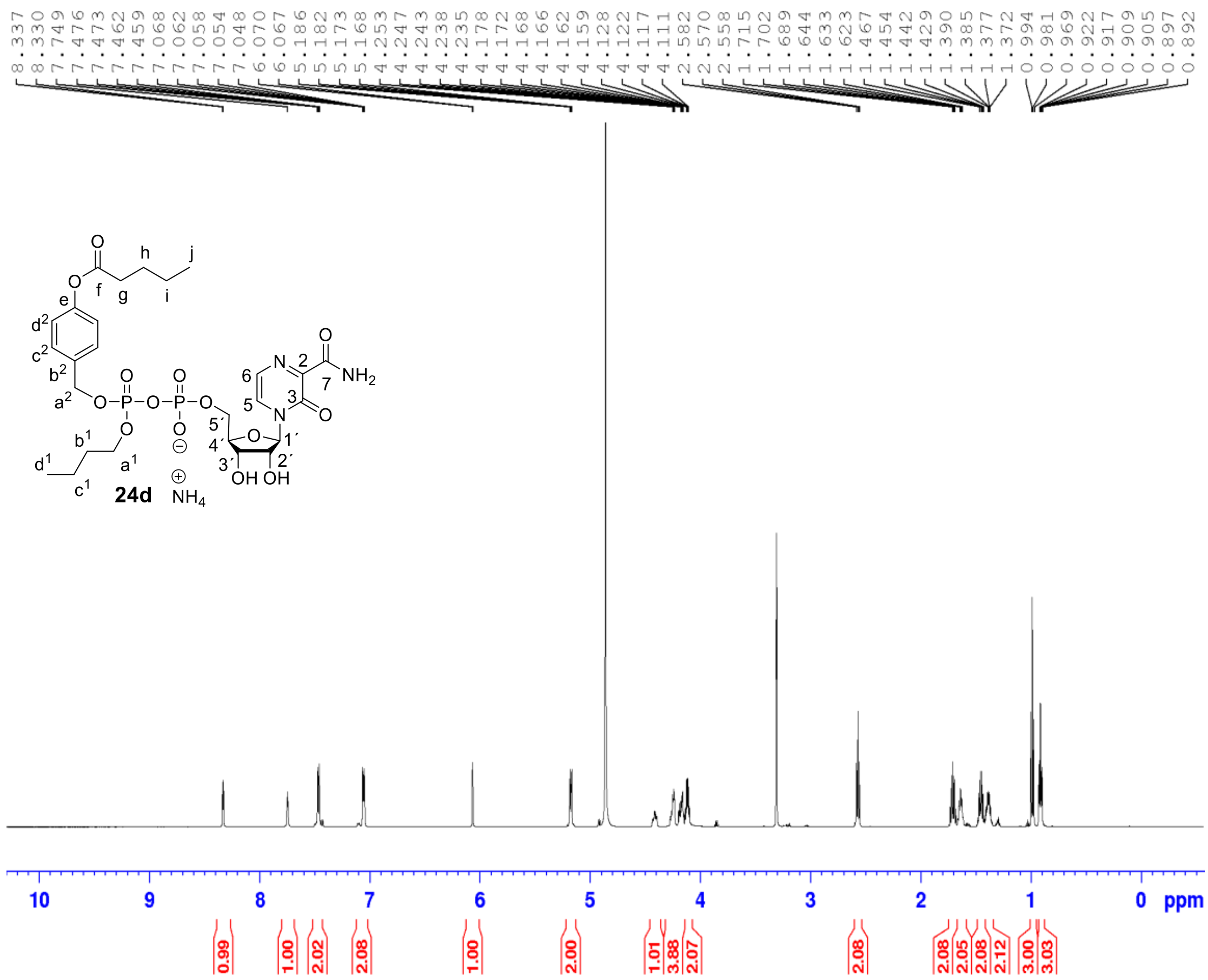
8.337 8.330 7.749 7.476 7.473 7.462 7.459 7.068 7.062 7.058 7.054 7.048 6.070 6.067 5.186 5.182 5.173 5.168 4.253 4.247 4.243 4.238 4.235 4.178 4.172 4.168 4.166 4.162 4.159 4.128 4.122 4.117 4.111 2.582 2.570 2.558 1.715 1.702 1.689 1.644 1.633 1.623 1.467 1.454 1.442 1.429 1.390 1.385 1.377 1.372 0.994 0.981 0.969 0.922 0.917 0.909 0.905 0.897 0.892
24d
NH4
10 9 8 7 6 5 4 3 2 1 0 ppm
0.99 1.00 2.02 2.08 1.00 2.00 1.01 3.88 2.07 2.08 2.08 2.05 2.08 2.12 3.00 3.03

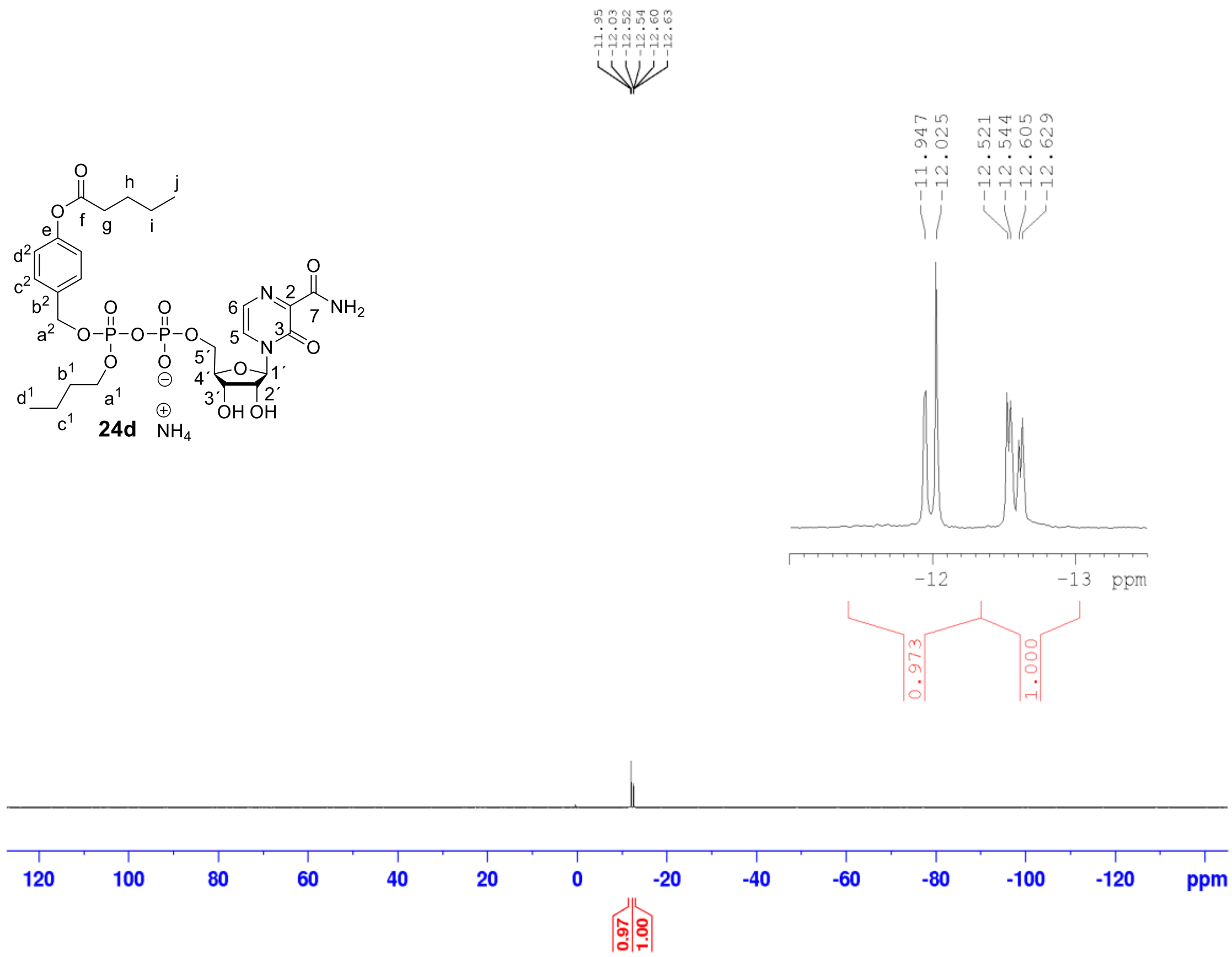
-11.95 -12.03 -12.52 -12.54 -12.60 -12.63
-11.947 -12.025 -12.521 -12.544 -12.605 -12.629
24d
NH4
-12 -13 ppm
0.973 1.000
120 100 80 60 40 20 0 -20 -40 -60 -80 -100 -120 ppm
0.97 1.00

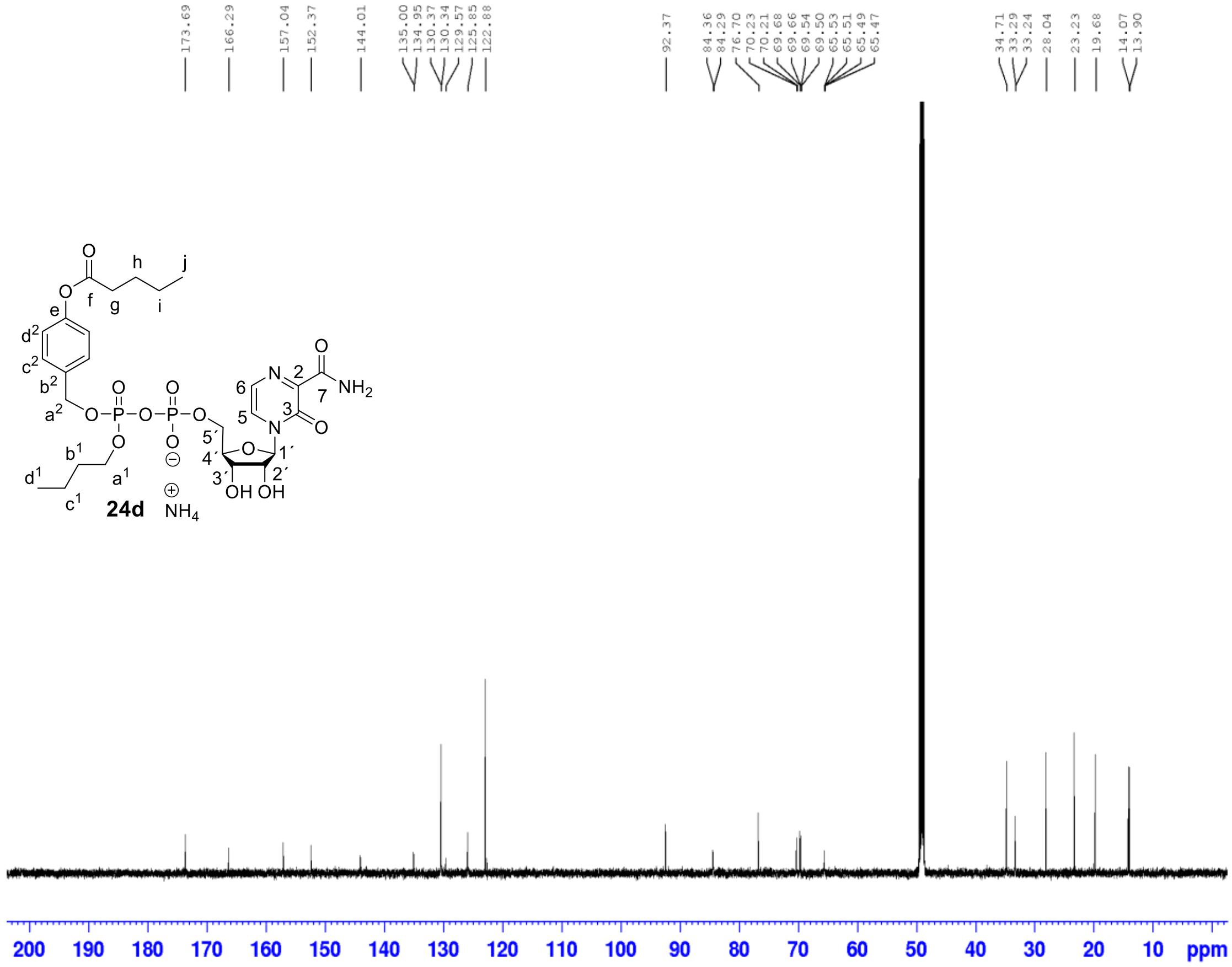

24d
173.69
166.29
157.04
152.37
144.01
135.00
134.95
130.37
130.34
129.57
125.85
122.88
92.37
84.36
84.29
76.70
70.23
70.21
69.68
69.66
69.54
69.50
65.53
65.51
65.49
65.47
34.71
33.29
33.24
28.04
23.23
19.68
14.07
13.90
200 190 180 170 160 150 140 130 120 110 100 90 80 70 60 50 40 30 20 10 ppm

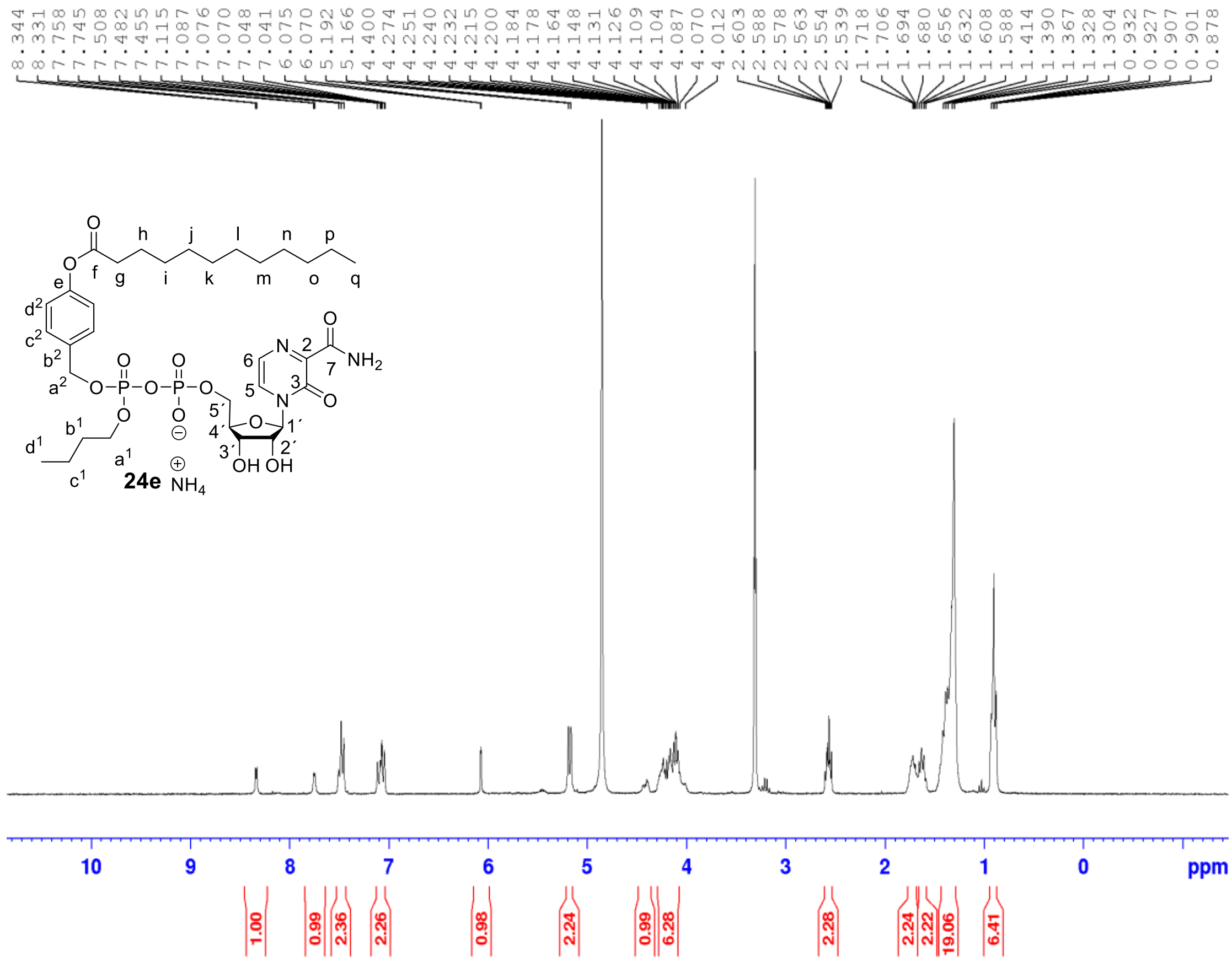

24e
8.344
8.331
7.758
7.745
7.508
7.482
7.455
7.115
7.087
7.076
7.070
7.048
7.041
6.075
6.070
5.192
5.166
4.400
4.274
4.251
4.240
4.232
4.215
4.200
4.184
4.178
4.164
4.148
4.131
4.126
4.109
4.104
4.087
4.070
4.012
2.603
2.588
2.578
2.563
2.554
2.539
1.718
1.706
1.694
1.680
1.656
1.632
1.608
1.588
1.414
1.390
1.367
1.328
1.304
0.932
0.927
0.907
0.901
0.878
10 9 8 7 6 5 4 3 2 1 0 ppm
1.00
0.99
2.36
2.26
0.98
2.24
0.99
6.28
2.28
2.24
2.22
19.06
6.41

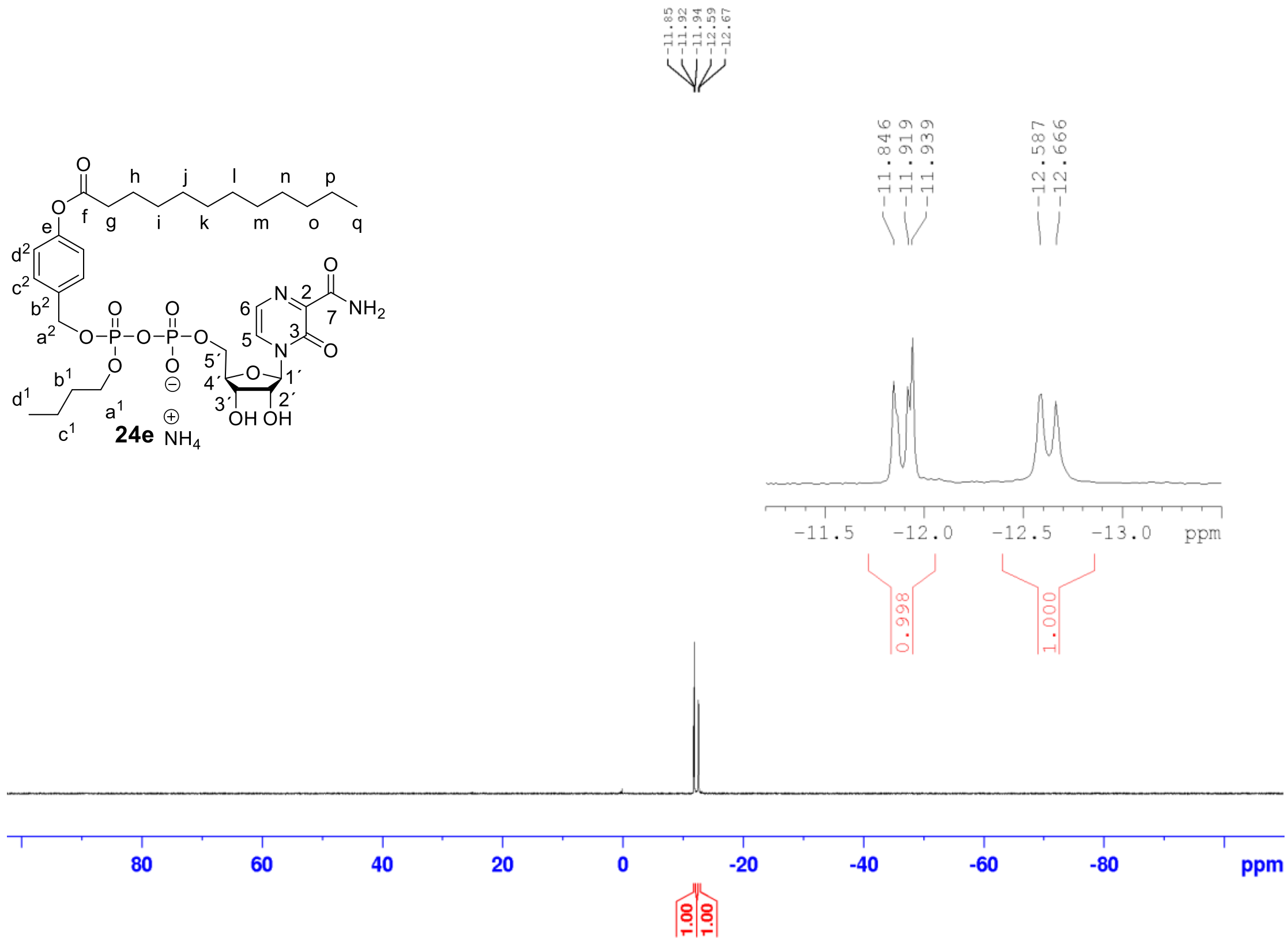
24e
-11.85
-11.92
-11.94
-12.59
-12.67
-11.846
-11.919
-11.939
-12.587
-12.666
0.998
1.000
ppm
1.00
1.00

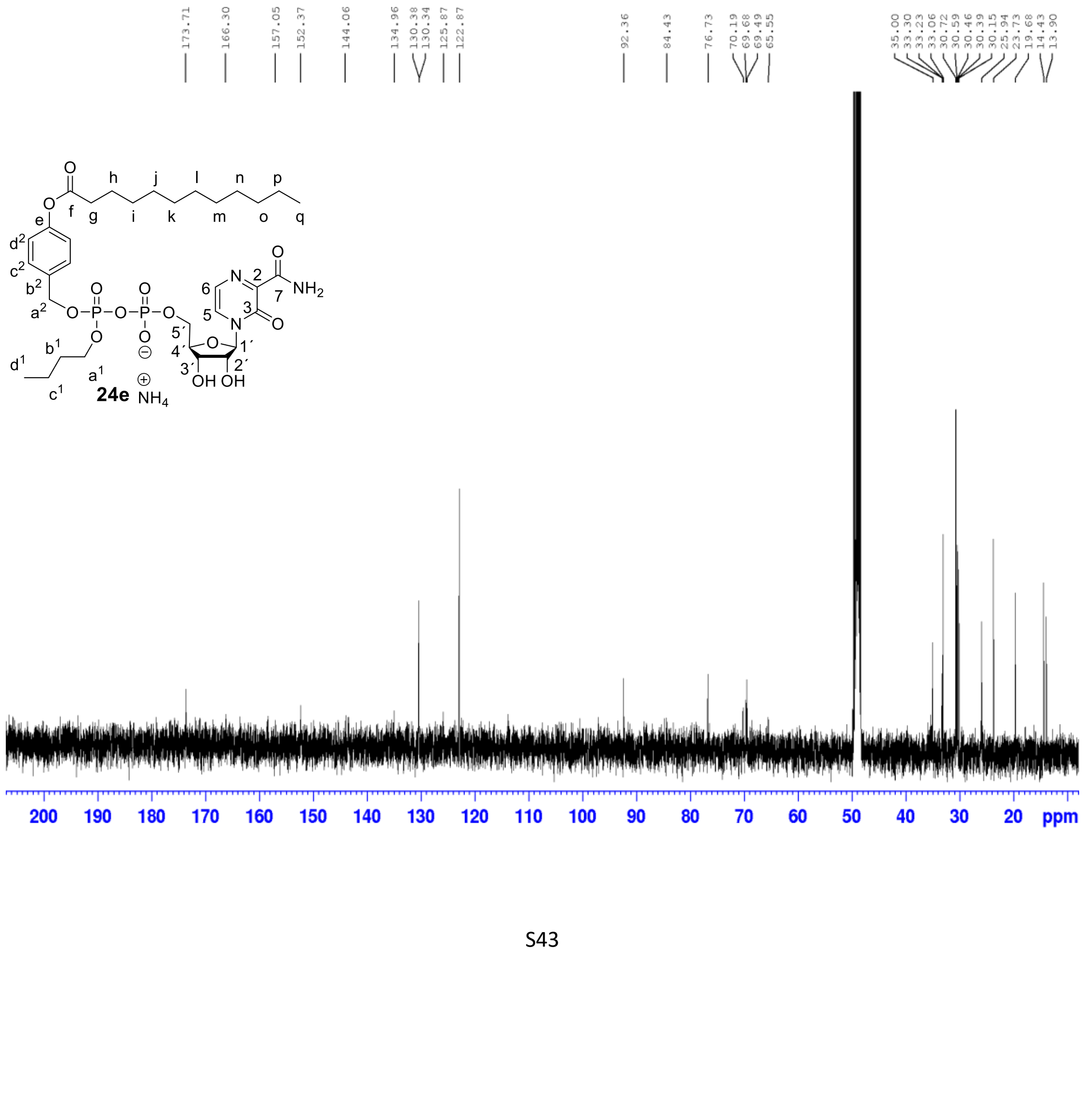
24e
173.71
166.30
157.05
152.37
144.06
134.96
130.38
130.34
125.87
122.87
92.36
84.43
76.73
70.19
69.68
69.49
65.55
35.00
33.30
33.23
33.06
30.72
30.59
30.46
30.39
30.15
25.94
23.73
19.68
14.43
13.90
ppm

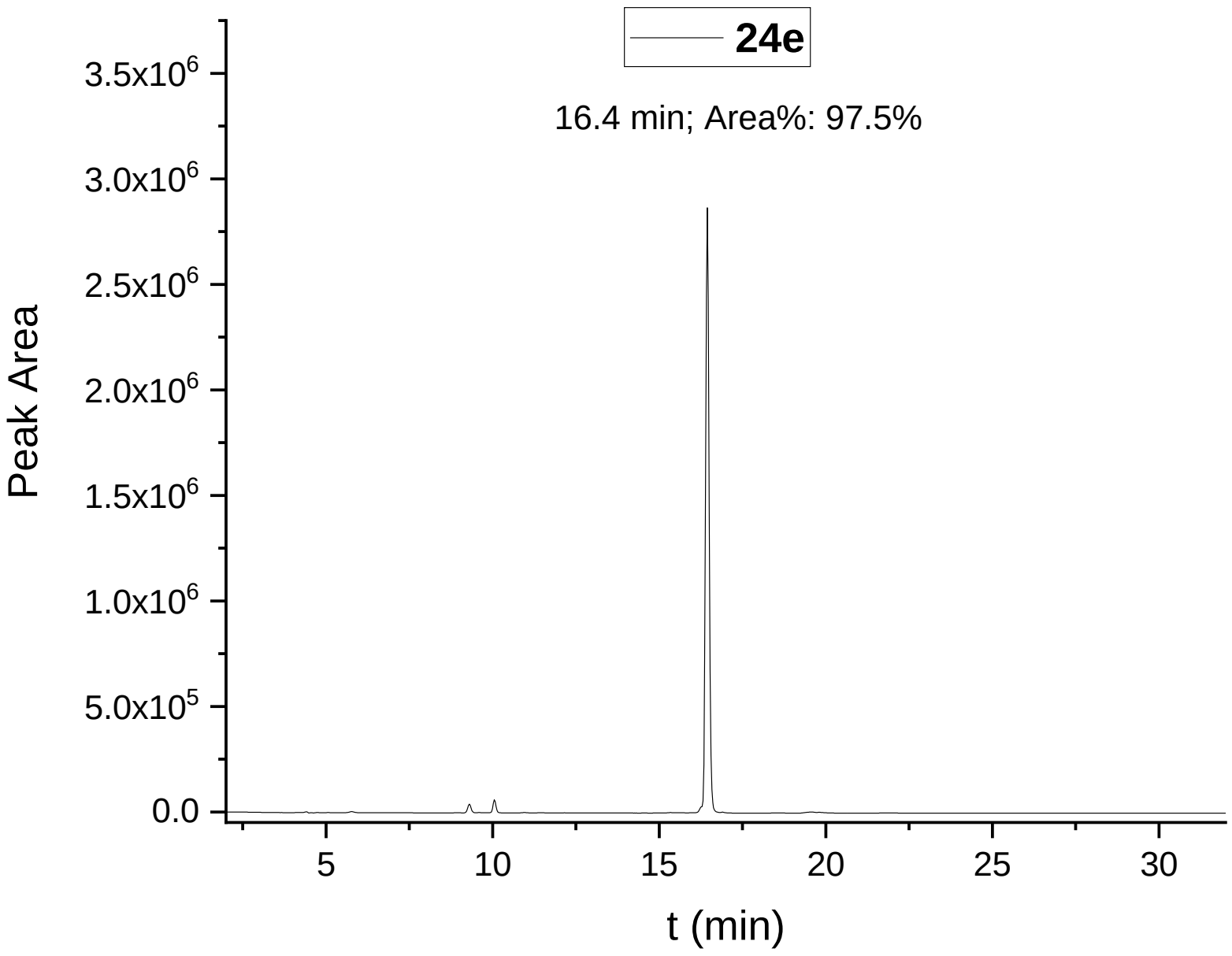
24e
16.4 min; Area%: 97.5%
Peak Area
t (min)

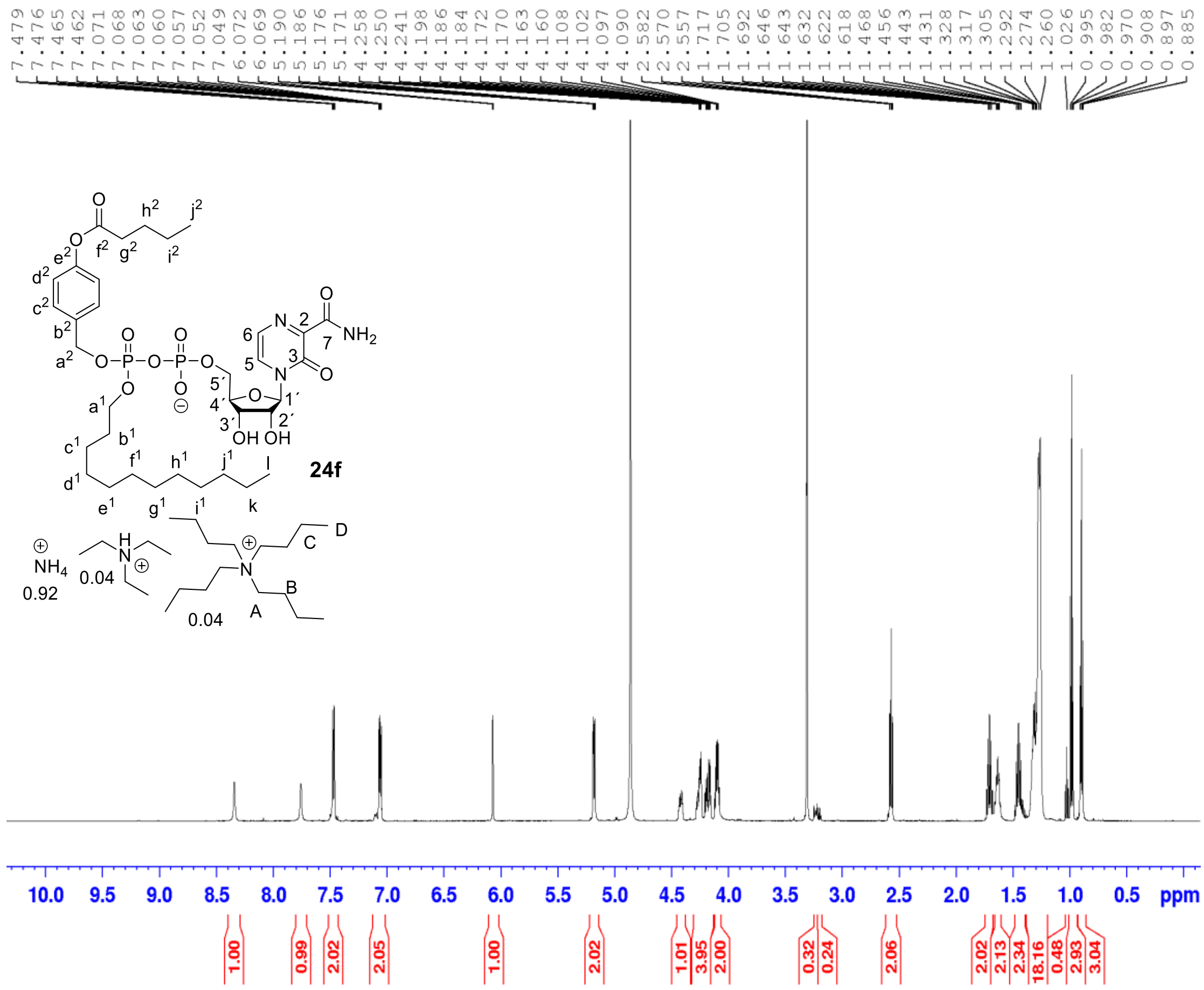
24f
NH4
0.92
0.04
0.04
A
B
C
D
ppm

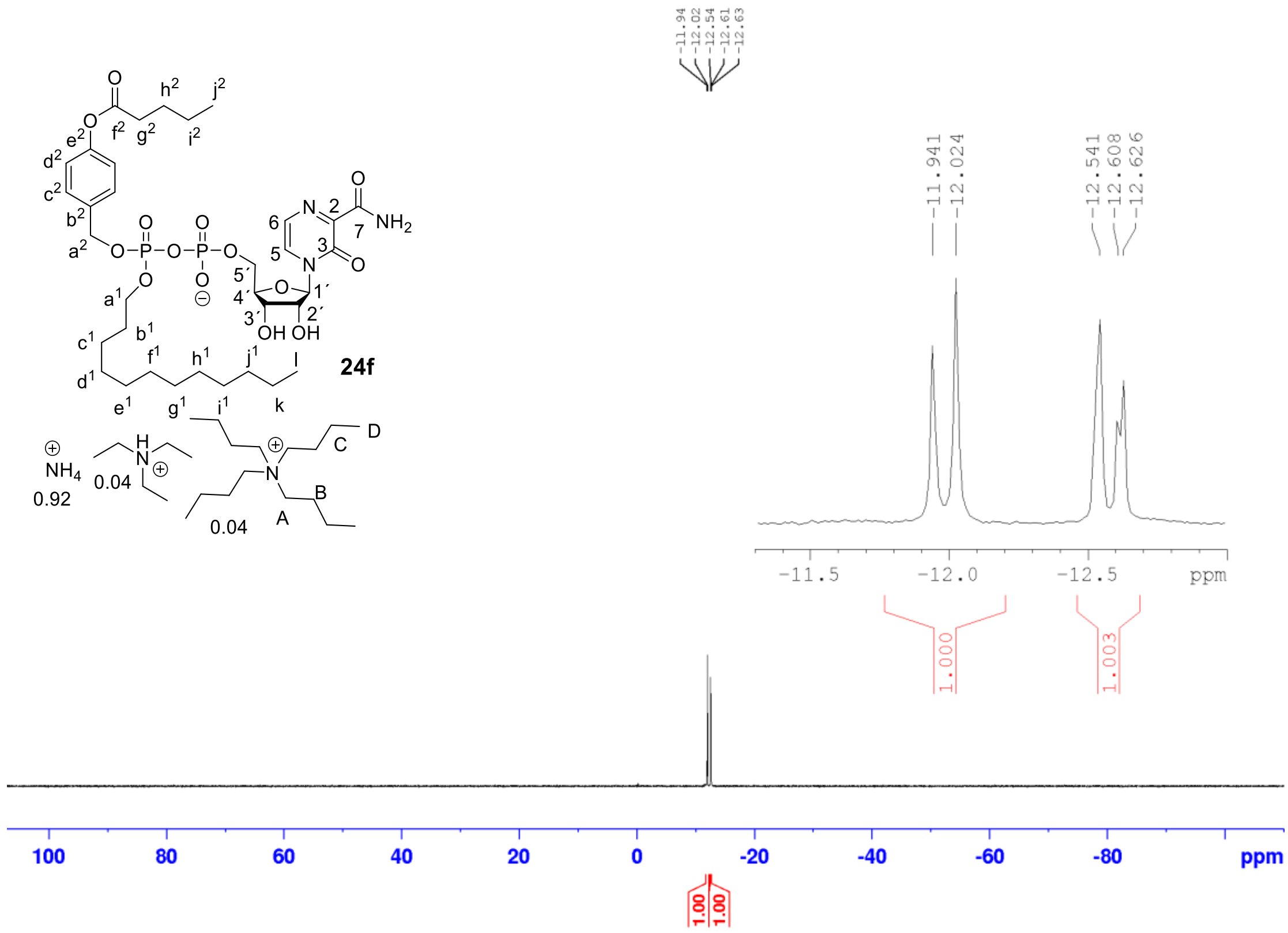
24f
0.92
0.04
0.04
-11.94
-12.02
-12.54
-12.61
-12.63
-11.941
-12.024
-12.541
-12.608
-12.626
1.000
1.003
1.00
1.00
ppm

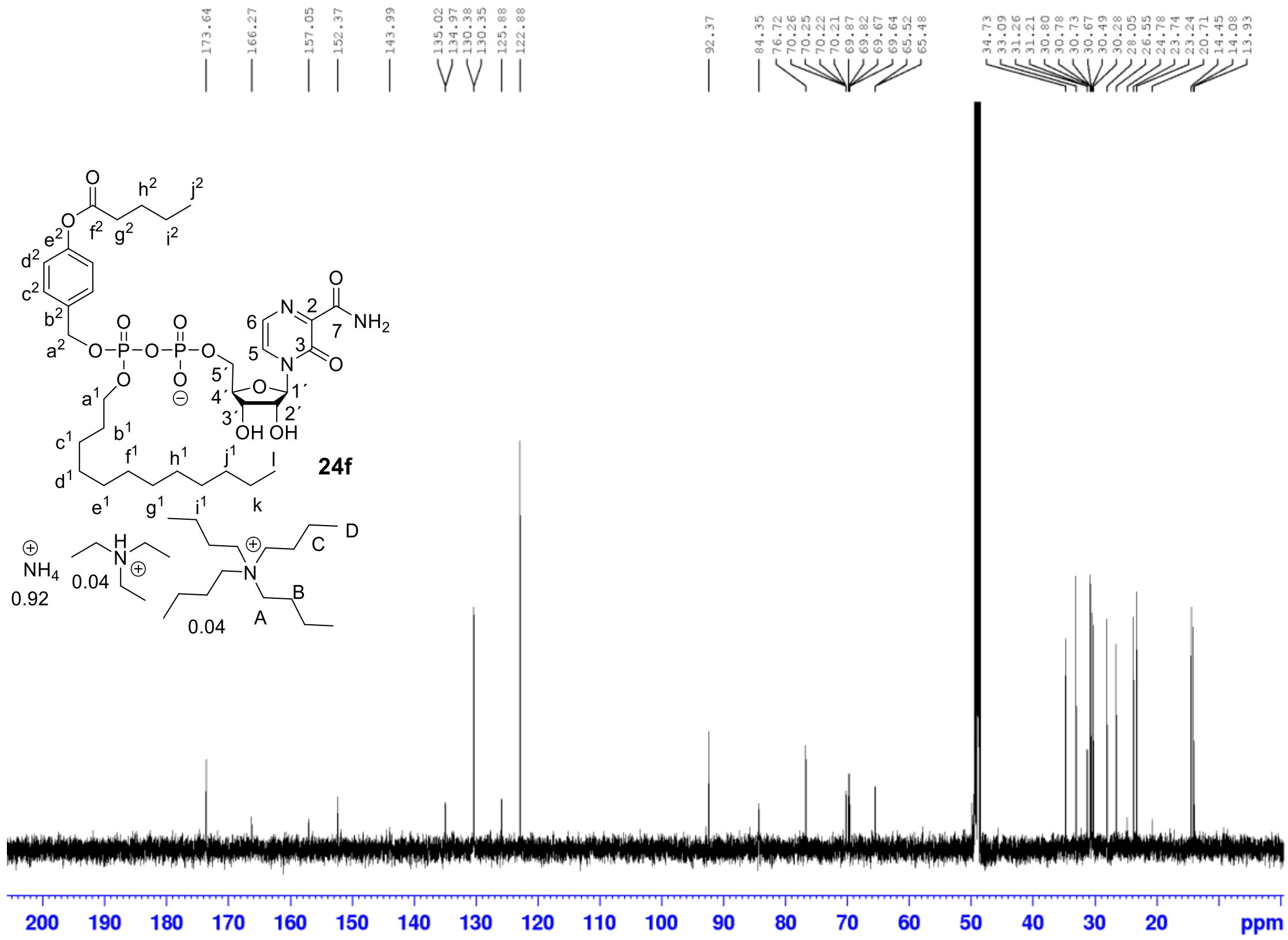
24f
173.64
166.27
157.05
152.37
143.99
135.02
134.97
130.38
130.35
125.88
122.88
92.37
84.35
76.72
70.26
70.25
70.22
70.21
69.87
69.82
69.67
69.64
65.52
65.48
34.73
33.09
31.26
31.21
30.80
30.78
30.73
30.67
30.49
30.28
28.05
26.55
24.78
23.74
23.24
20.71
14.45
14.08
13.93
ppm

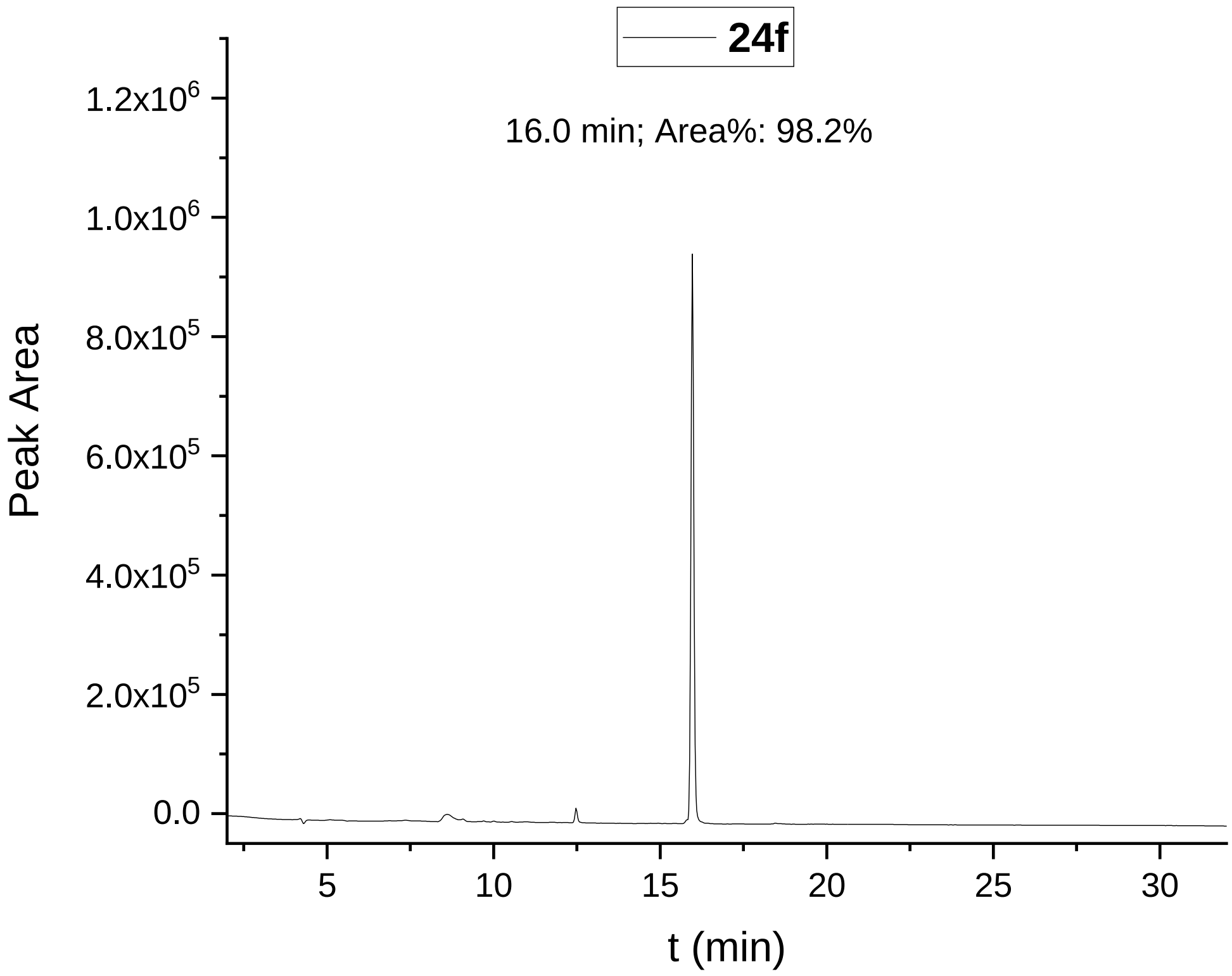
24f
16.0 min; Area%: 98.2%
Peak Area
1.2x10^6
1.0x10^6
8.0x10^5
6.0x10^5
4.0x10^5
2.0x10^5
0.0
5
10
15
20
25
30
t (min)

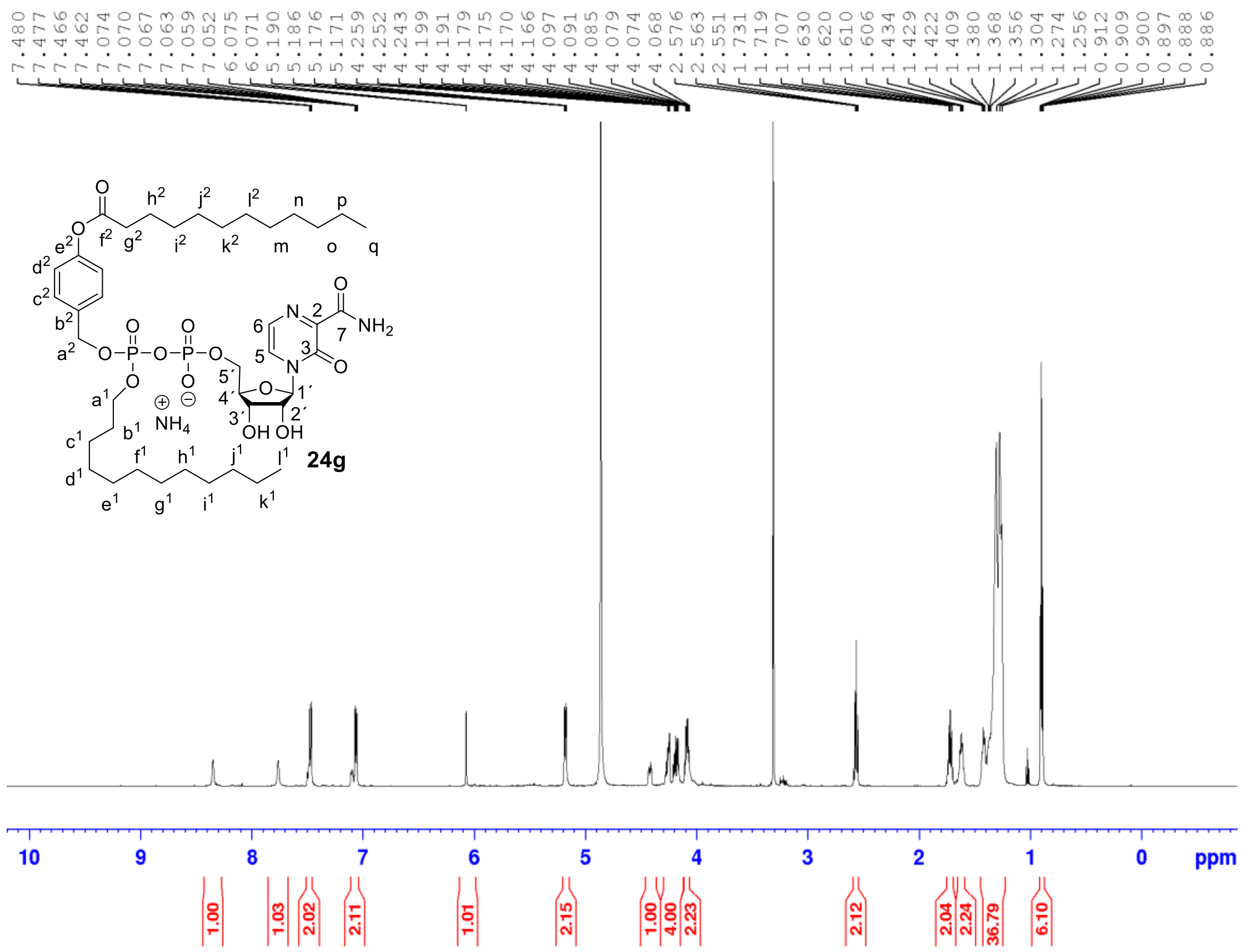
7.480 7.477 7.466 7.462 7.074 7.070 7.067 7.063 7.059 7.052 6.075 6.071 5.190 5.186 5.176 5.171 4.259 4.252 4.243 4.199 4.191 4.179 4.175 4.170 4.166 4.097 4.091 4.085 4.079 4.074 4.068 2.576 2.563 2.551 1.731 1.719 1.707 1.630 1.620 1.610 1.606 1.434 1.429 1.422 1.409 1.380 1.368 1.356 1.304 1.274 1.256 0.912 0.909 0.900 0.897 0.888 0.886
24g
10
9
8
7
6
5
4
3
2
1
0
ppm
1.00
1.03
2.02
2.11
1.01
2.15
1.00
4.00
2.23
2.12
2.04
2.24
36.79
6.10

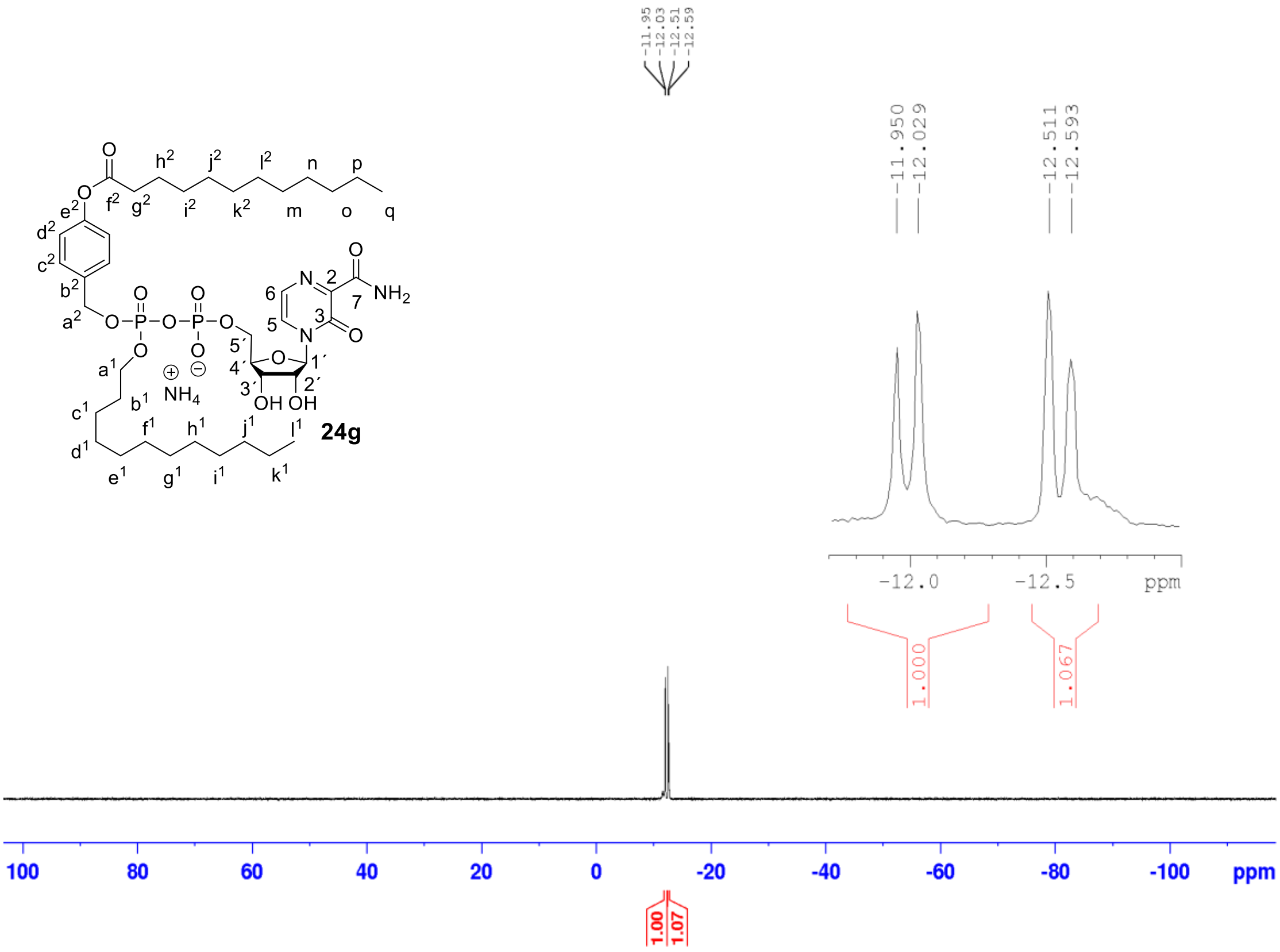
-11.95
-12.03
-12.51
-12.59
-11.950
-12.029
-12.511
-12.593
-12.0
-12.5
ppm
1.000
1.067
24g
100
80
60
40
20
0
-20
-40
-60
-80
-100
ppm
1.00
1.07

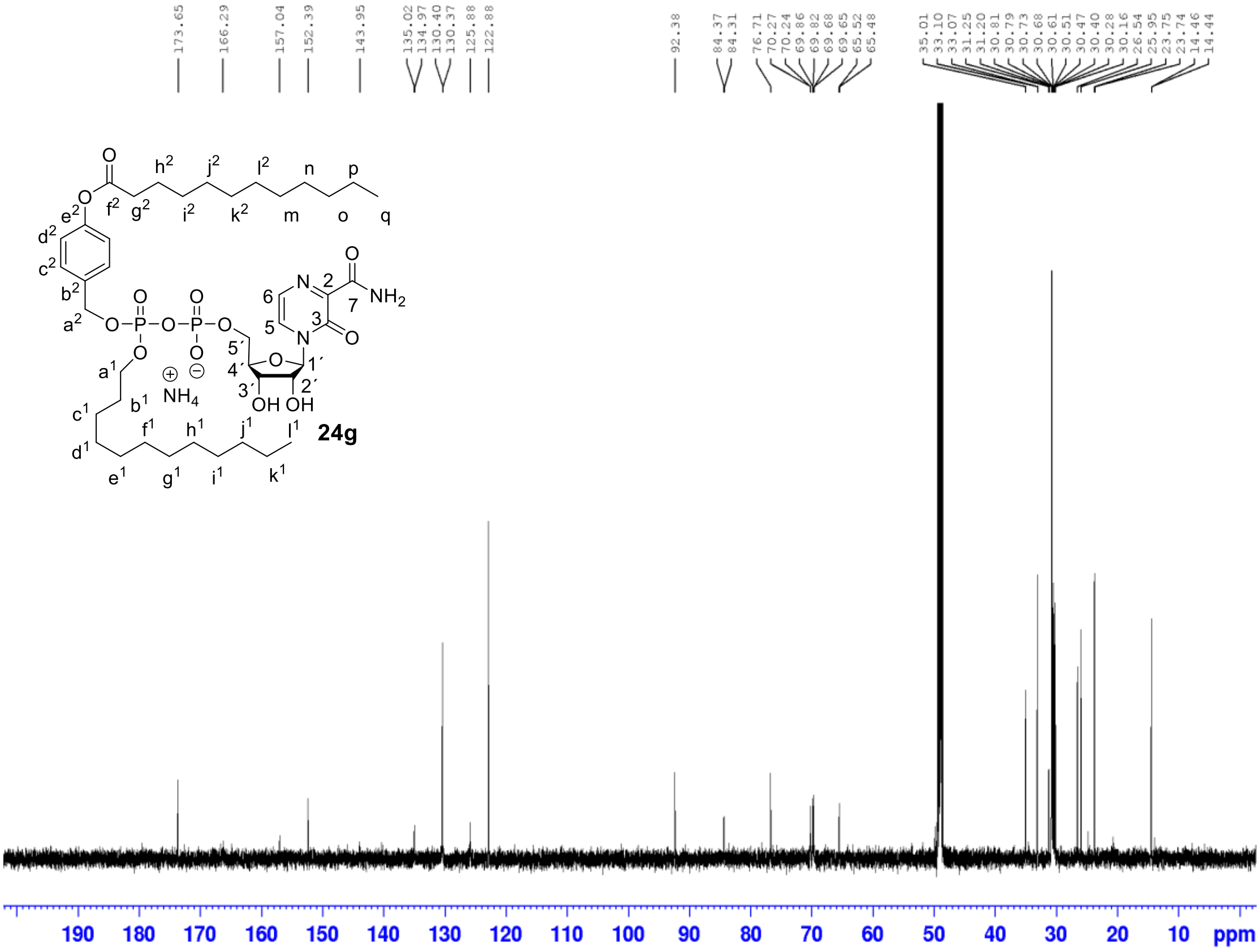
173.65
166.29
157.04
152.39
143.95
135.02
134.97
130.40
130.37
125.88
122.88
92.38
84.37
84.31
76.71
70.27
70.24
69.86
69.82
69.68
69.65
65.52
65.48
35.01
33.10
33.07
31.25
31.20
30.81
30.79
30.73
30.68
30.61
30.51
30.47
30.40
30.28
30.16
26.54
25.95
23.75
23.74
14.46
14.44
24g
190
180
170
160
150
140
130
120
110
100
90
80
70
60
50
40
30
20
10
ppm

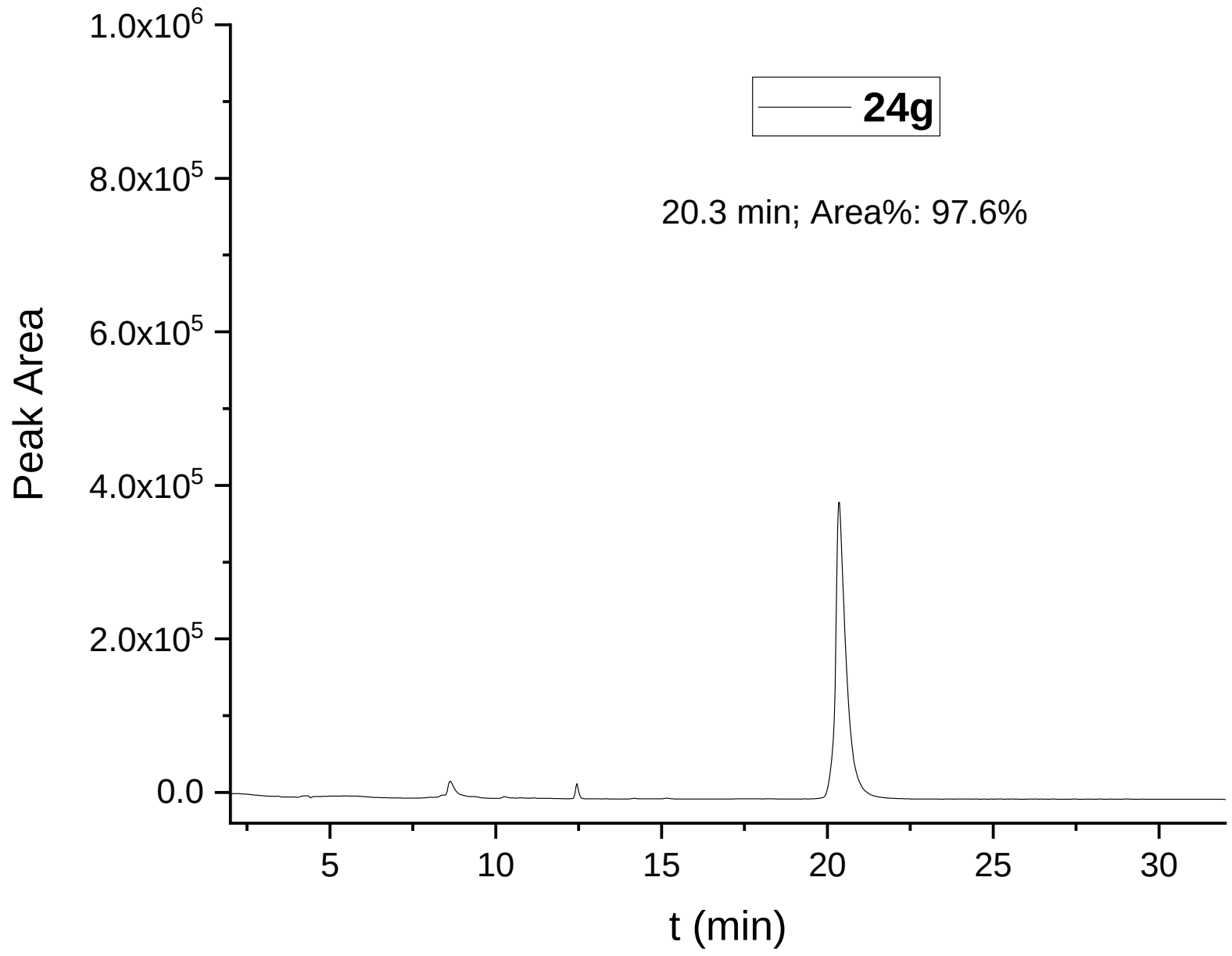
24g
20.3 min; Area%: 97.6%
Peak Area
1.0x10^6
8.0x10^5
6.0x10^5
4.0x10^5
2.0x10^5
0.0
5
10
15
20
25
30
t (min)

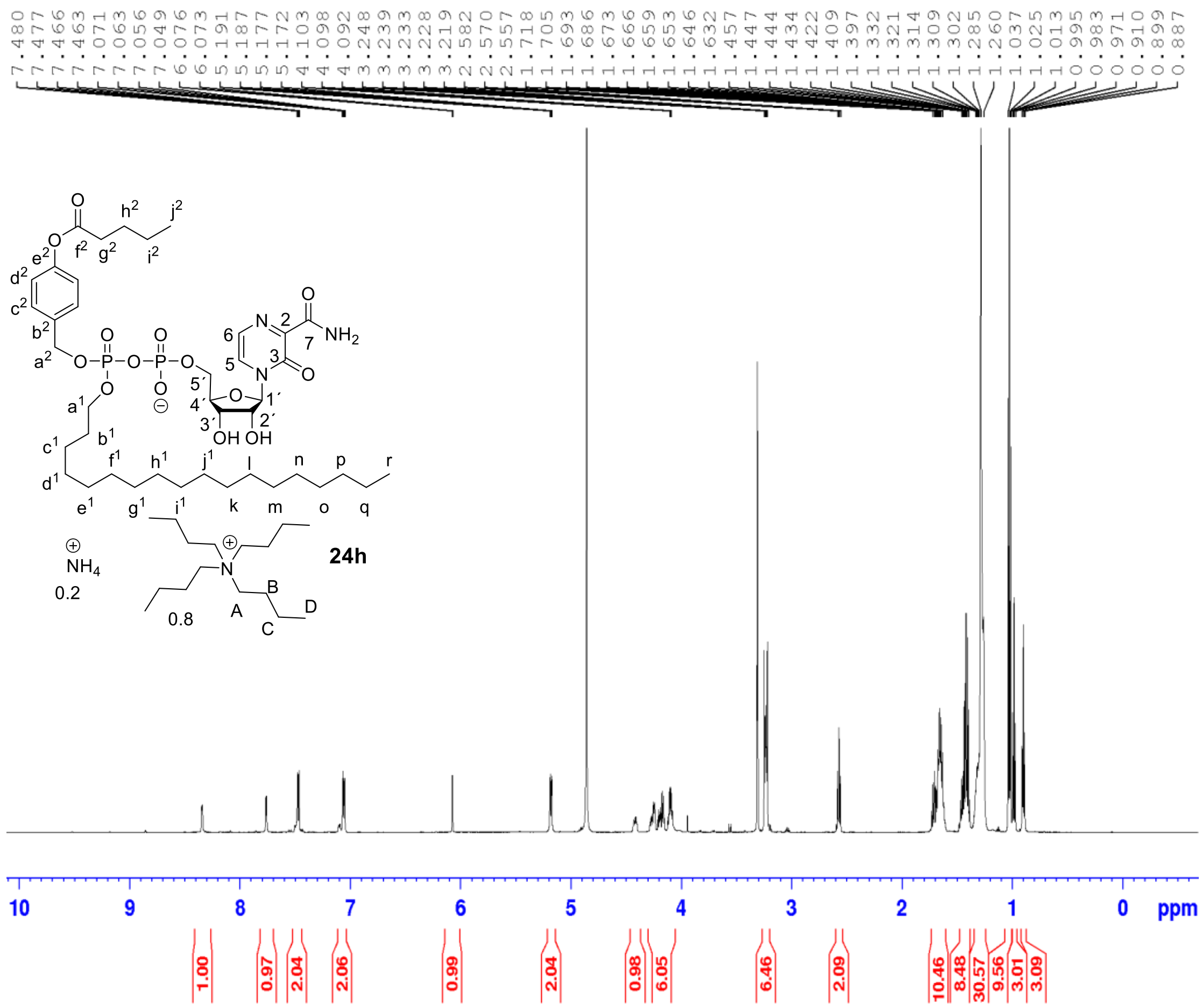
7.480 7.477 7.466 7.463 7.071 7.063 7.056 7.049 6.076 6.073 5.191 5.187 5.177 5.172 4.103 4.098 4.092 3.248 3.239 3.233 3.228 3.219 2.582 2.570 2.557 1.718 1.705 1.693 1.686 1.673 1.666 1.659 1.653 1.646 1.632 1.457 1.447 1.444 1.434 1.422 1.409 1.397 1.332 1.321 1.314 1.309 1.302 1.285 1.260 1.037 1.025 1.013 0.995 0.983 0.971 0.910 0.899 0.887
24h
NH4
0.2
0.8
10
9
8
7
6
5
4
3
2
1
0
ppm
1.00
0.97
2.04
2.06
0.99
2.04
0.98
6.05
6.46
2.09
10.46
8.48
30.57
9.56
3.01
3.09

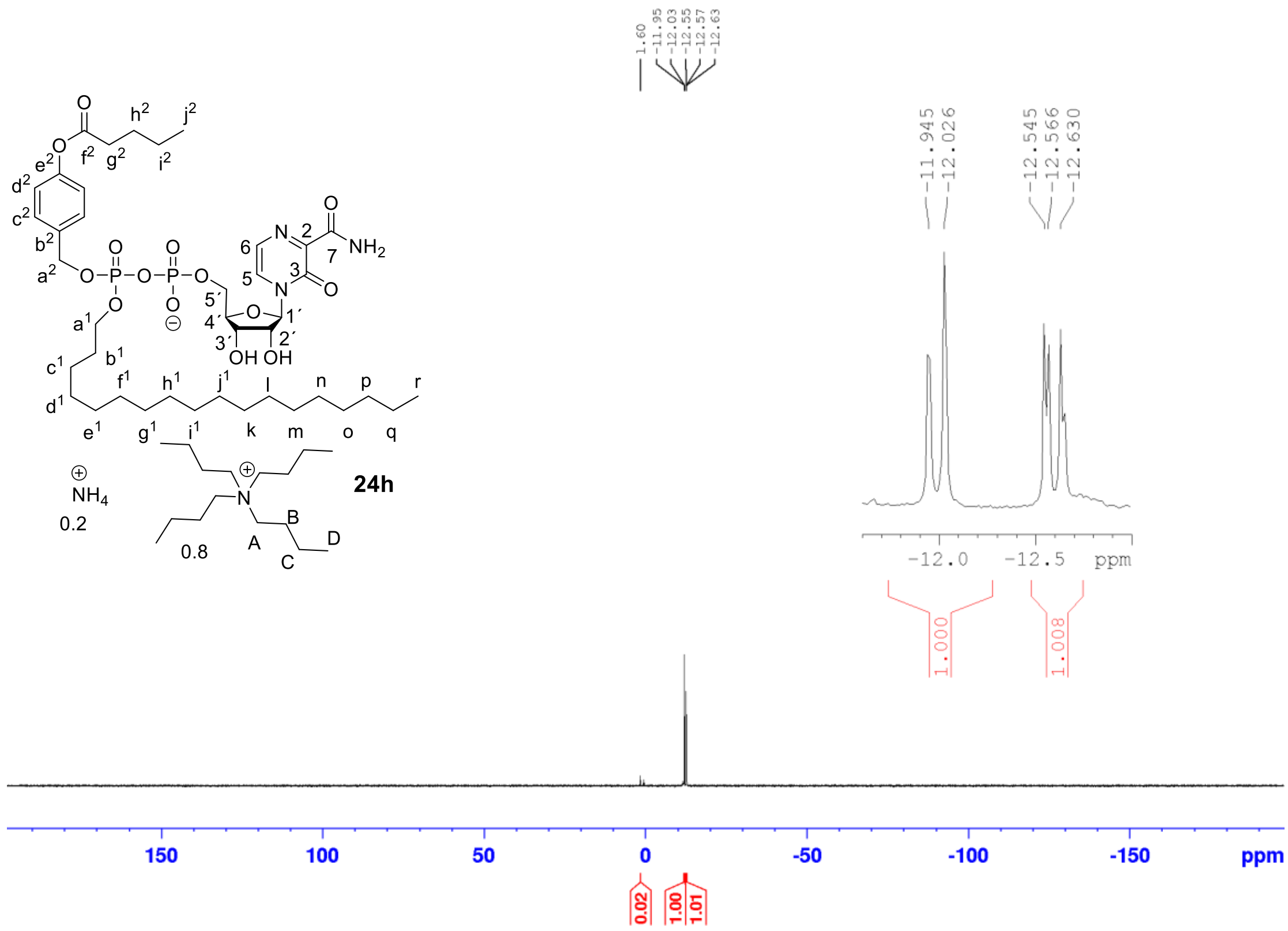

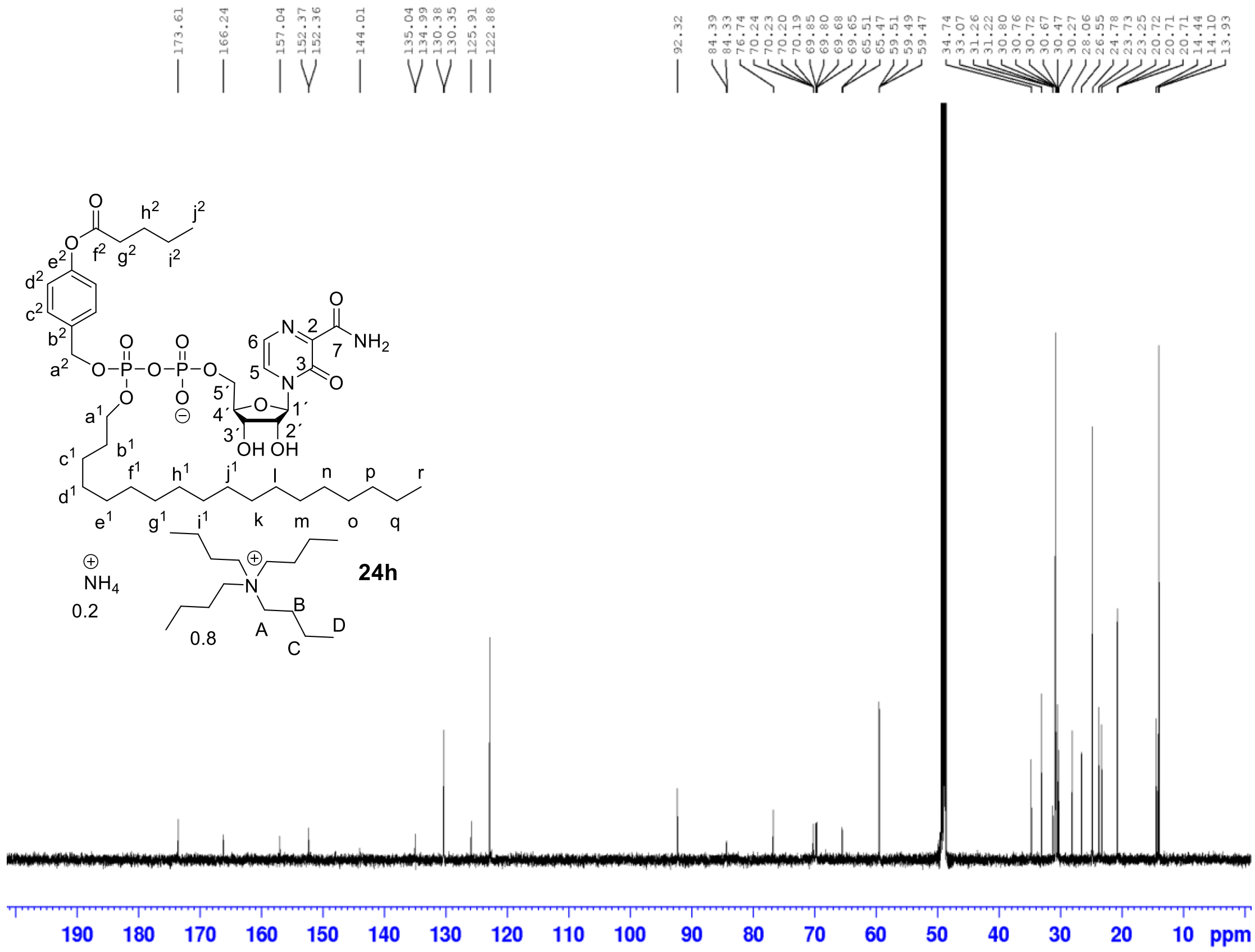

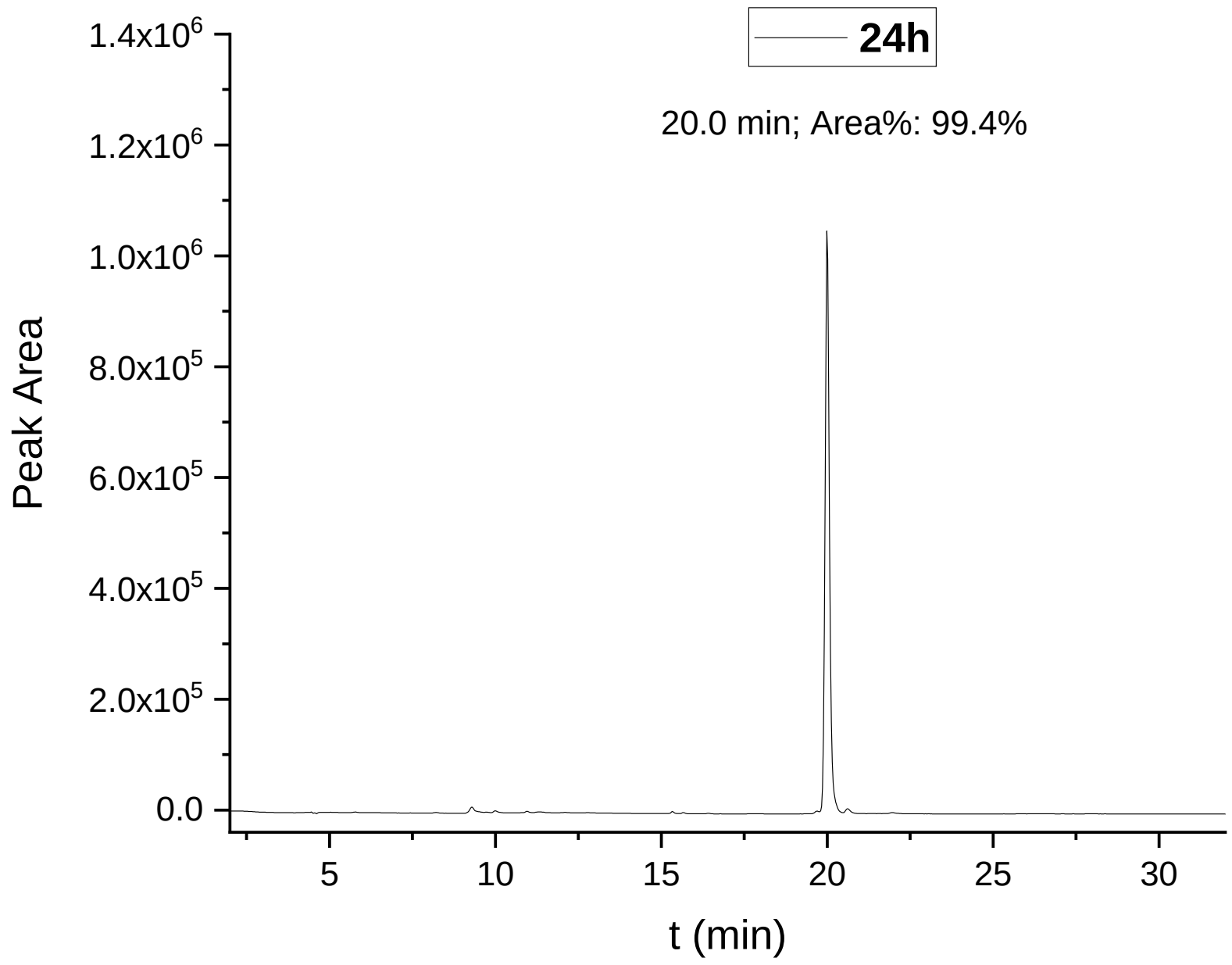

24h
20.0 min; Area%: 99.4%
Peak Area
1.4x10^6
1.2x10^6
1.0x10^6
8.0x10^5
6.0x10^5
4.0x10^5
2.0x10^5
0.0
5
10
15
20
25
30
t (min)

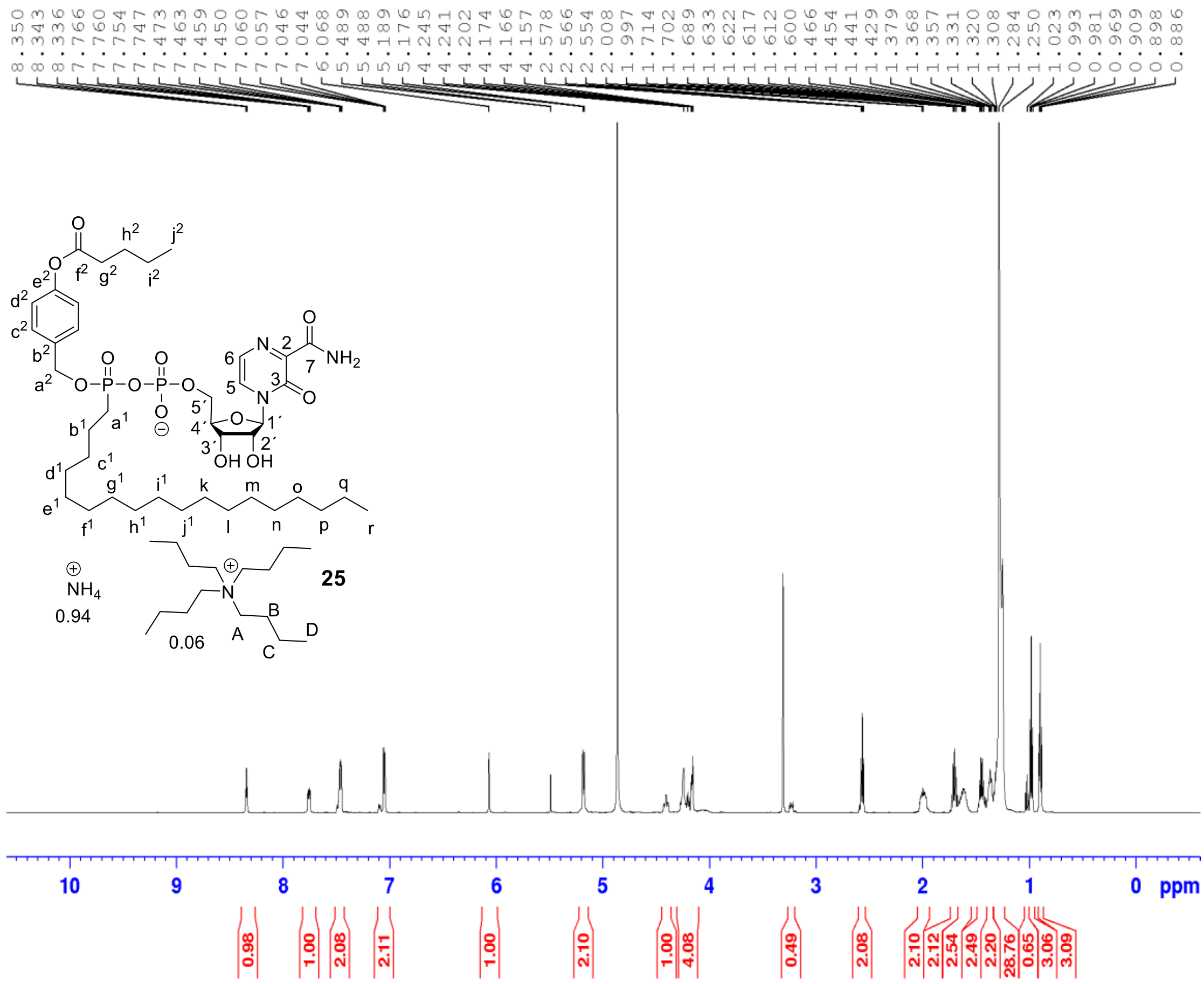

8.350
8.343
8.336
7.766
7.760
7.754
7.747
7.473
7.463
7.459
7.450
7.060
7.057
7.046
7.044
6.068
5.489
5.189
5.176
4.245
4.241
4.202
4.174
4.166
4.157
2.578
2.566
2.554
2.008
1.997
1.714
1.702
1.689
1.633
1.622
1.617
1.612
1.600
1.466
1.454
1.441
1.429
1.379
1.368
1.357
1.331
1.320
1.308
1.284
1.250
1.023
0.993
0.981
0.969
0.909
0.898
0.886
NH2
OH OH
NH4
0.94
0.06
25
10
9
8
7
6
5
4
3
2
1
0
ppm
0.98
1.00
2.08
2.11
1.00
2.10
1.00
4.08
0.49
2.08
2.10
2.12
2.54
2.49
2.20
28.76
0.65
3.06
3.09

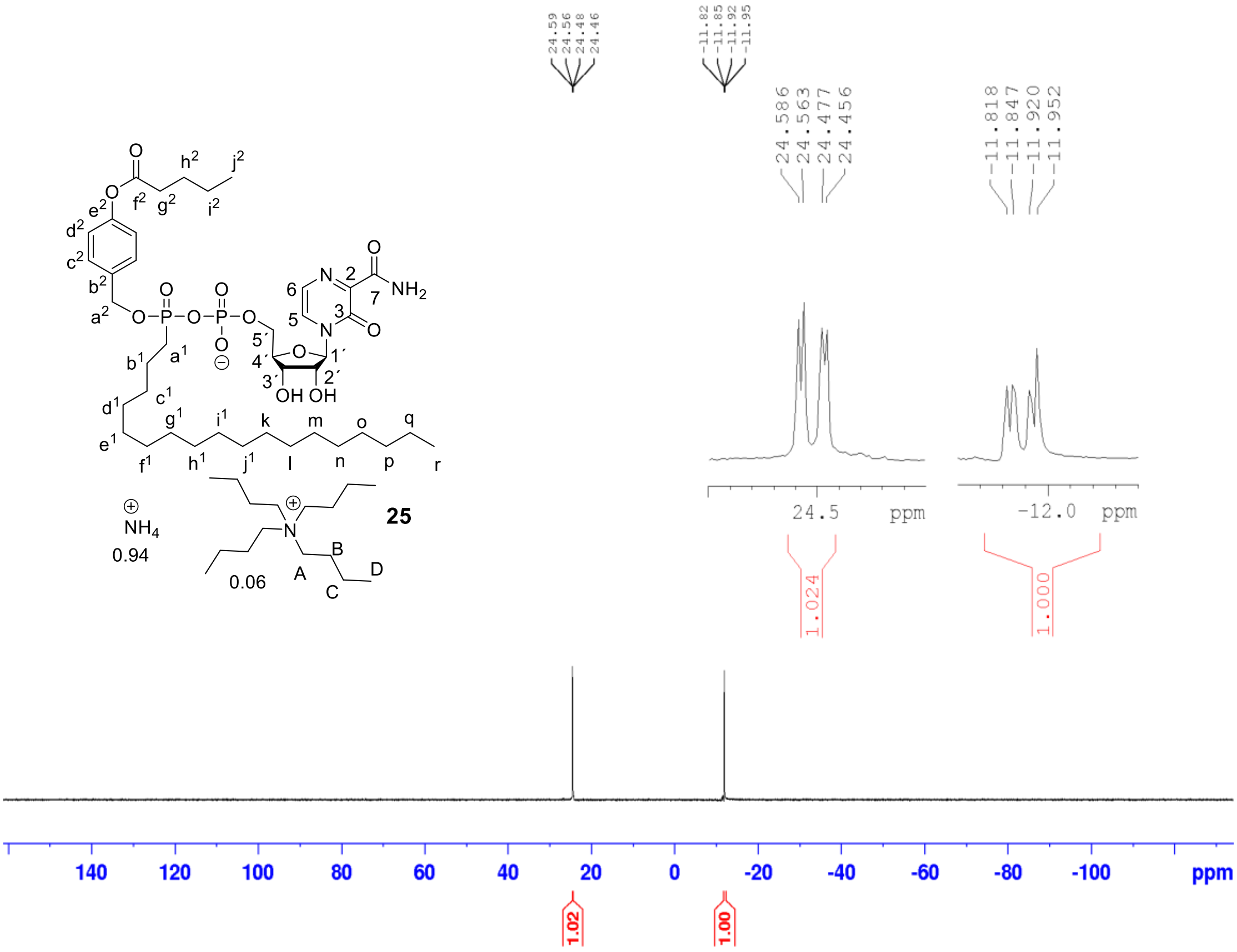
24.59
24.56
24.48
24.46
-11.82
-11.85
-11.92
-11.95
24.586
24.563
24.477
24.456
-11.818
-11.847
-11.920
-11.952
24.5 ppm
-12.0 ppm
1.024
1.000
25
0.94
0.06
1.02
1.00
ppm

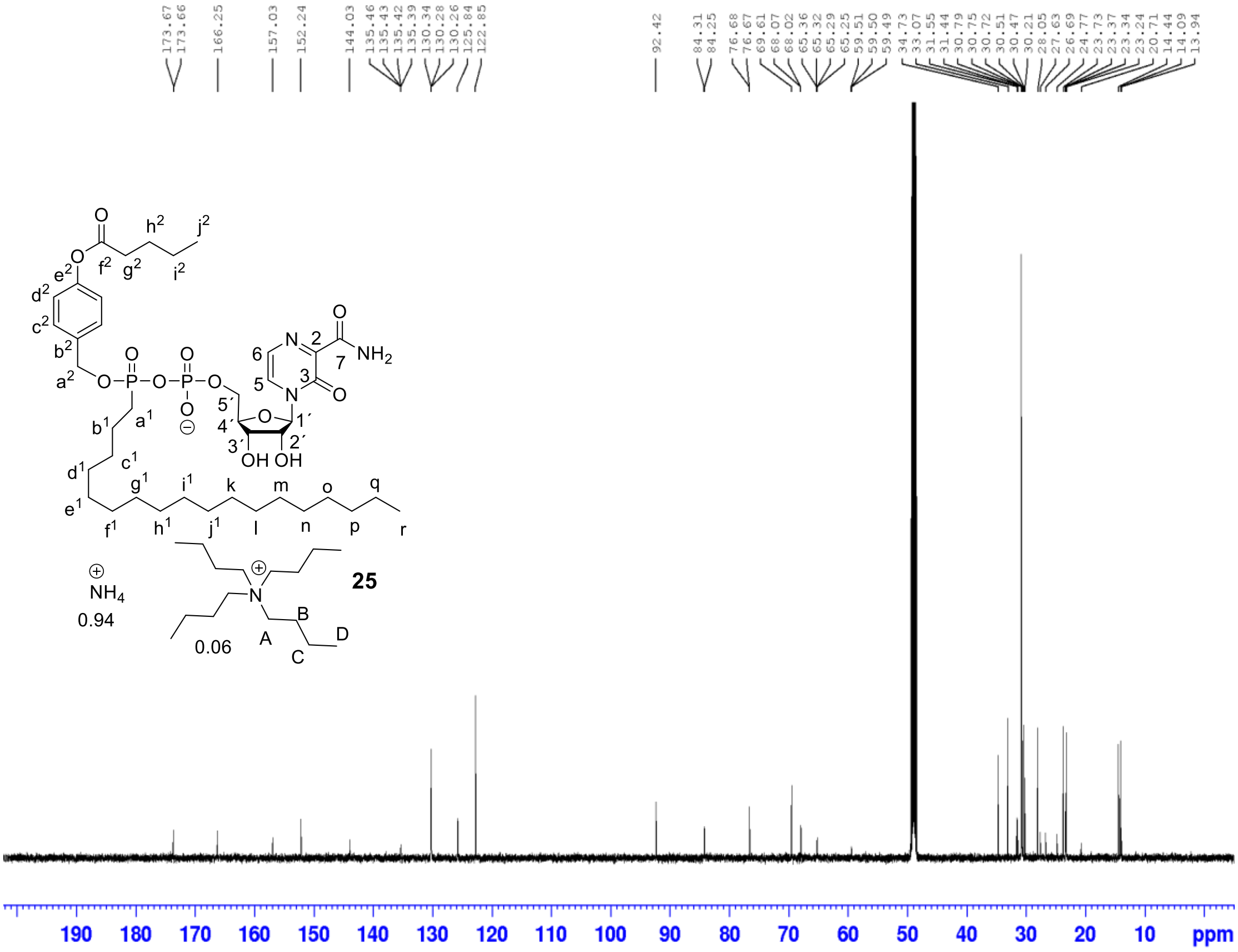
173.67
173.66
166.25
157.03
152.24
144.03
135.46
135.43
135.42
135.39
130.34
130.28
130.26
125.84
122.85
92.42
84.31
84.25
76.68
76.67
69.61
68.07
68.02
65.36
65.32
65.29
65.25
59.51
59.50
59.49
34.73
33.07
31.55
31.44
30.79
30.75
30.72
30.51
30.47
30.21
28.05
27.63
26.69
24.77
23.73
23.37
23.34
23.24
20.71
14.44
14.09
13.94
25
0.94
0.06
ppm

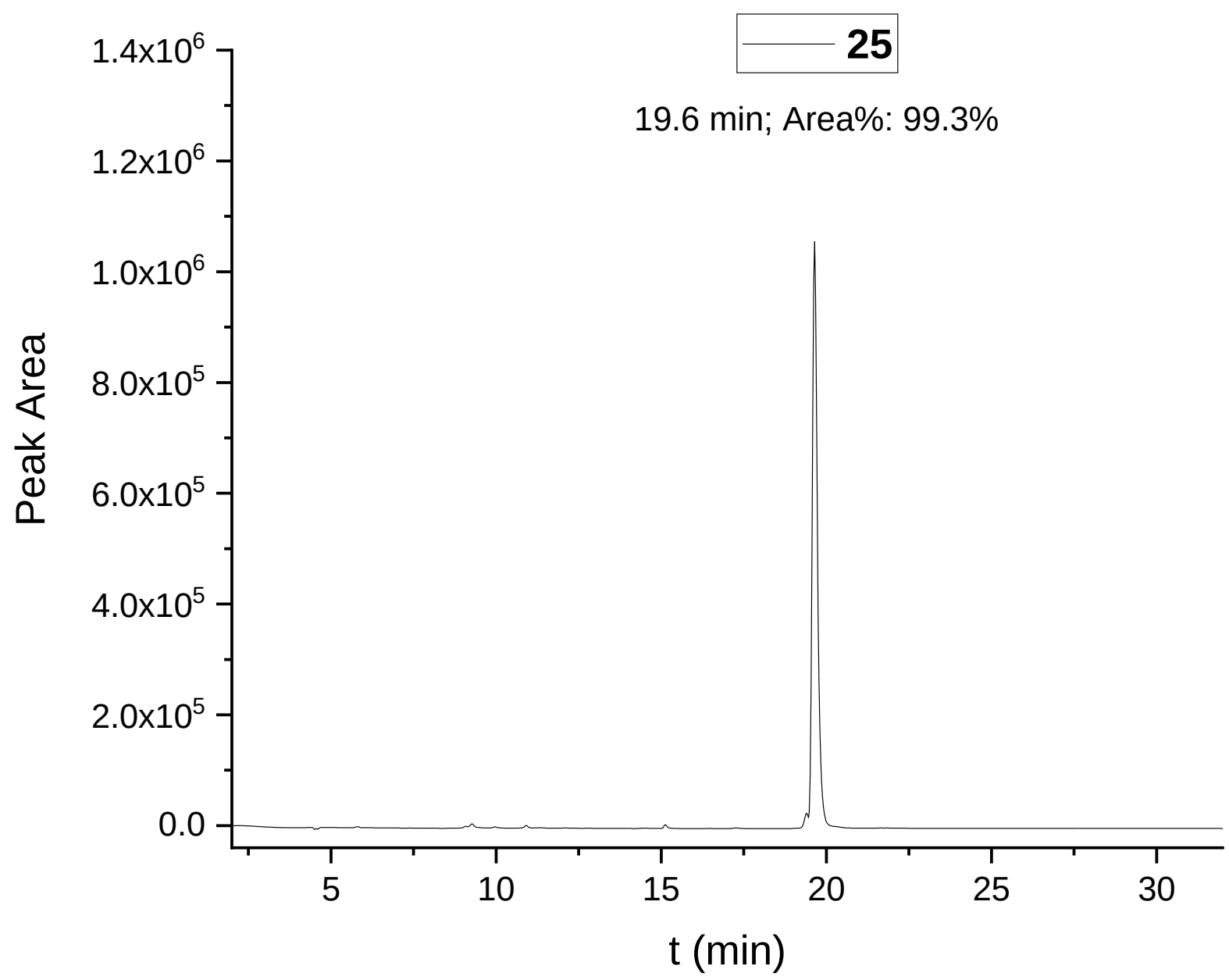
25
19.6 min; Area%: 99.3%
Peak Area
t (min)

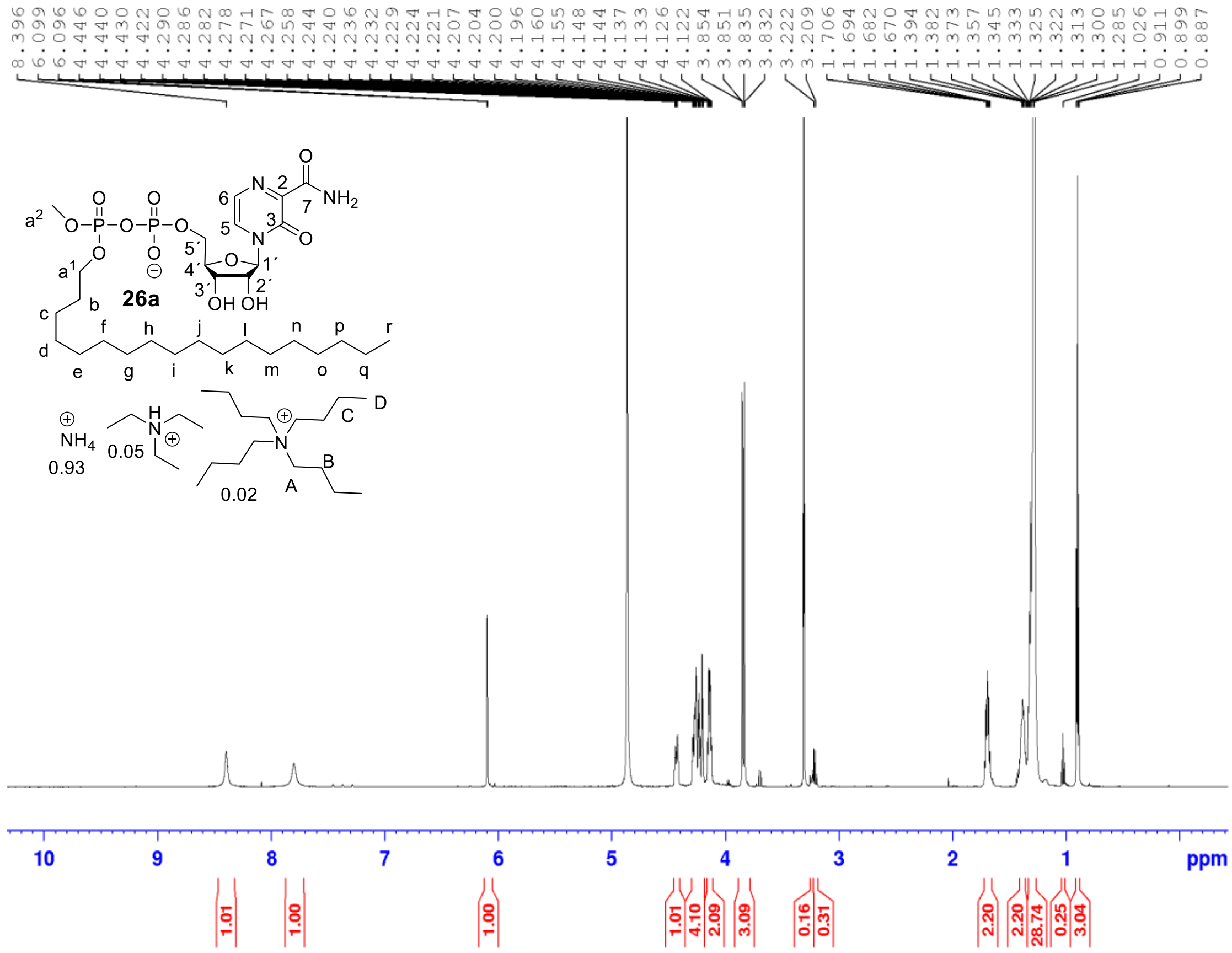
26a
NH4 0.93
0.05
0.02
ppm

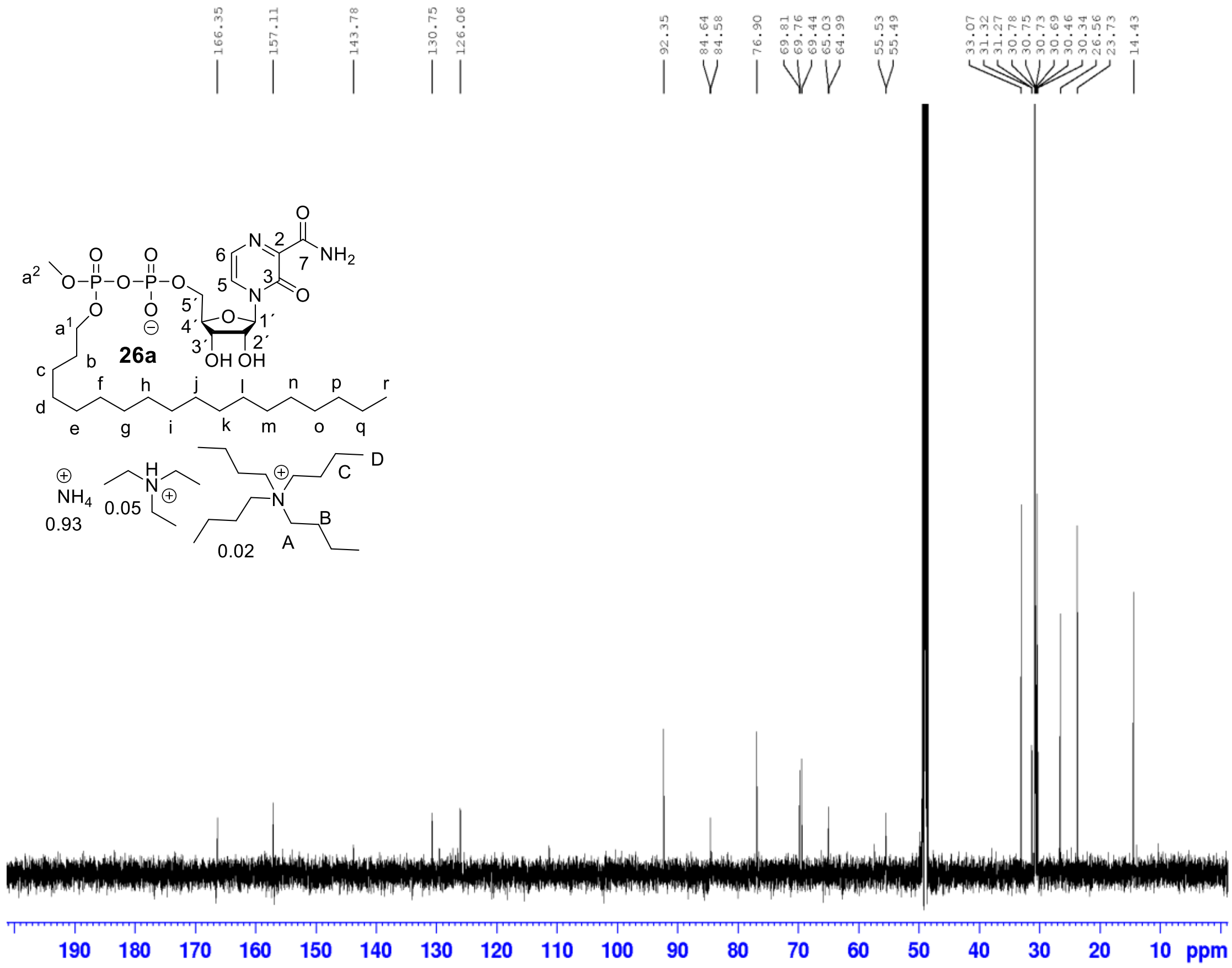
166.35
157.11
143.78
130.75
126.06
92.35
84.64
84.58
76.90
69.81
69.76
69.44
65.03
64.99
55.53
55.49
33.07
31.32
31.27
30.78
30.75
30.73
30.69
30.46
30.34
26.56
23.73
14.43
26a
0.93
0.05
0.02
ppm

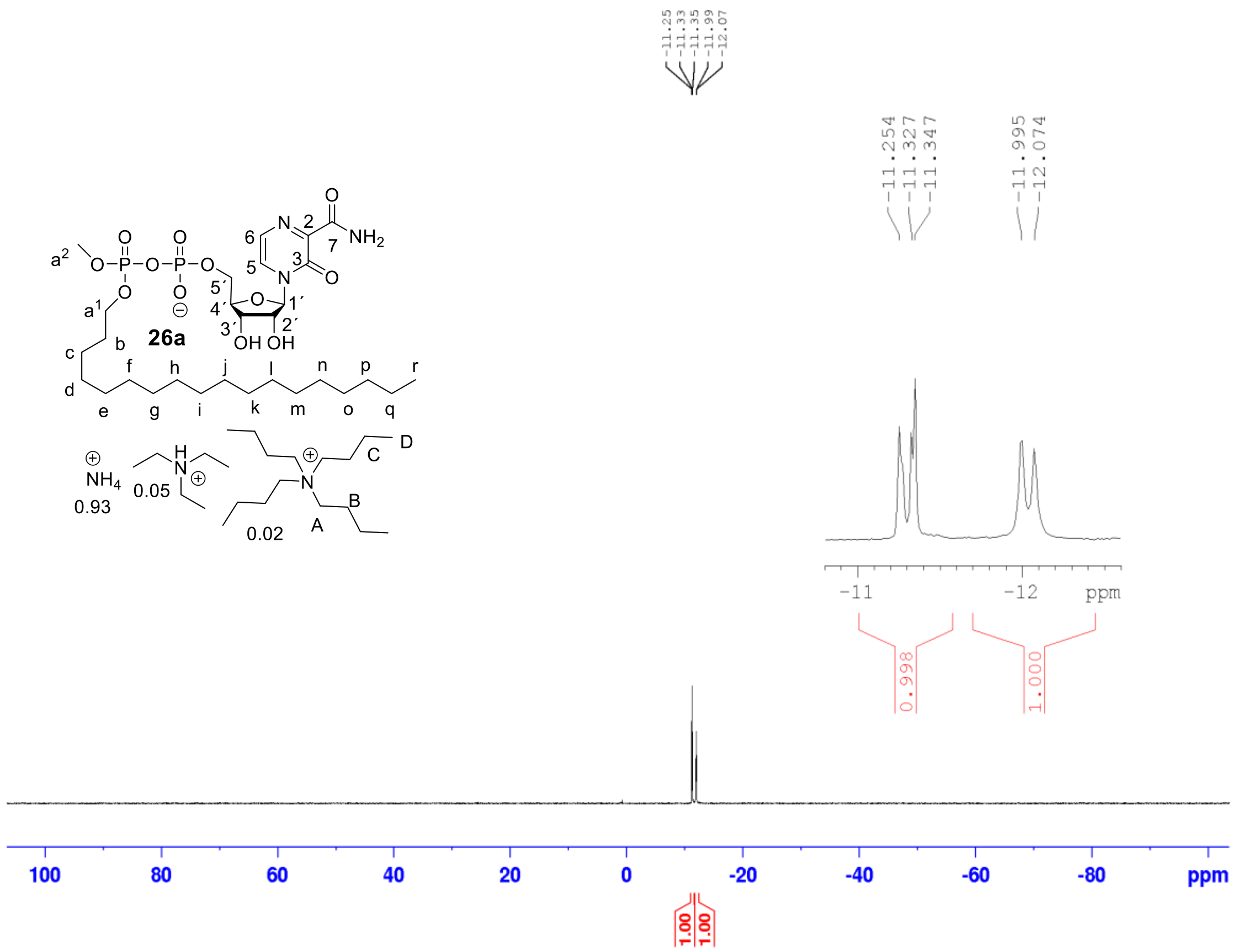
-11.25
-11.33
-11.35
-11.99
-12.07
-11.254
-11.327
-11.347
-11.995
-12.074
0.998
1.000
26a
0.93
0.05
0.02
1.00
1.00
ppm

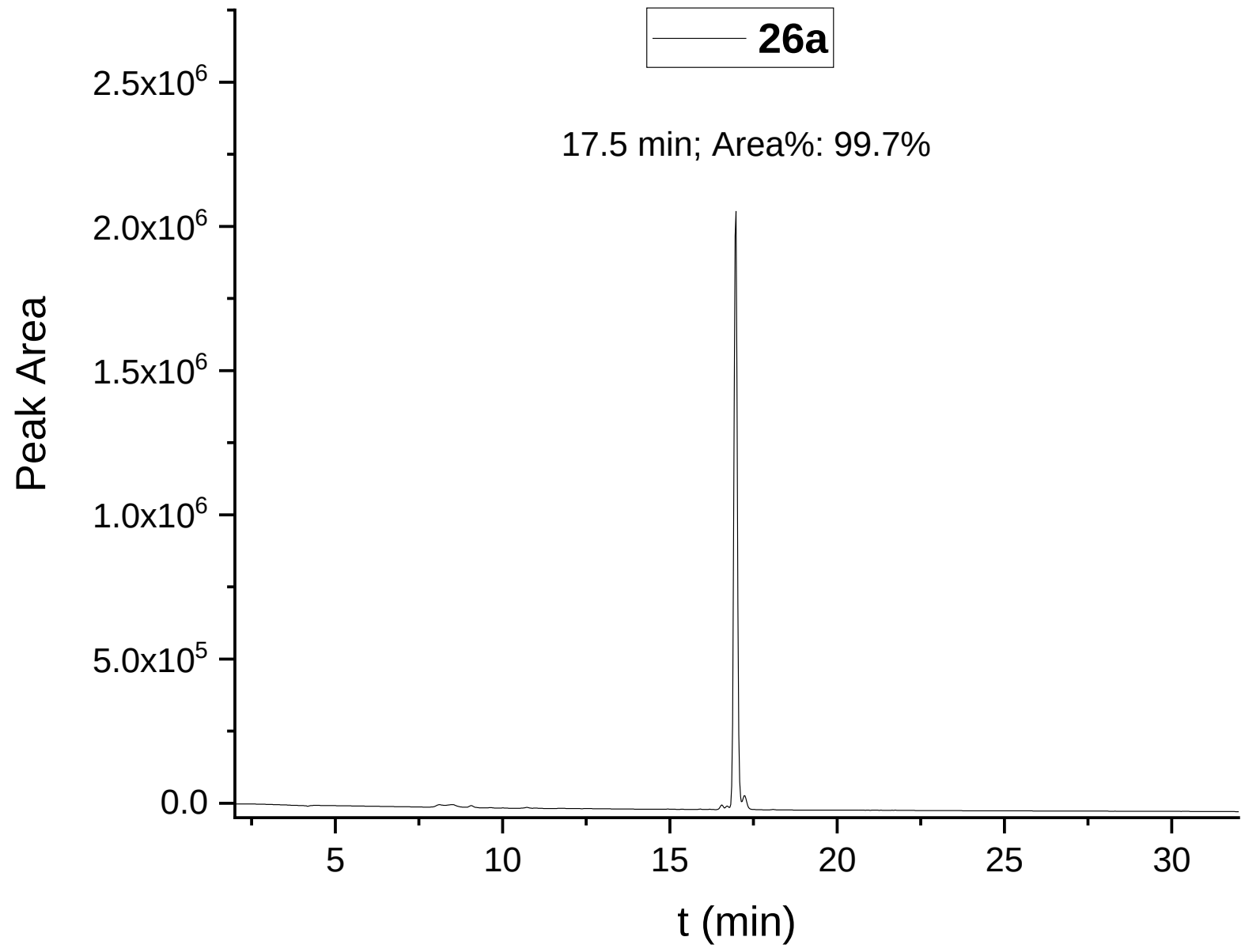

26a
17.5 min; Area%: 99.7%
Peak Area
t (min)
2.5x10^6
2.0x10^6
1.5x10^6
1.0x10^6
5.0x10^5
0.0
5
10
15
20
25
30

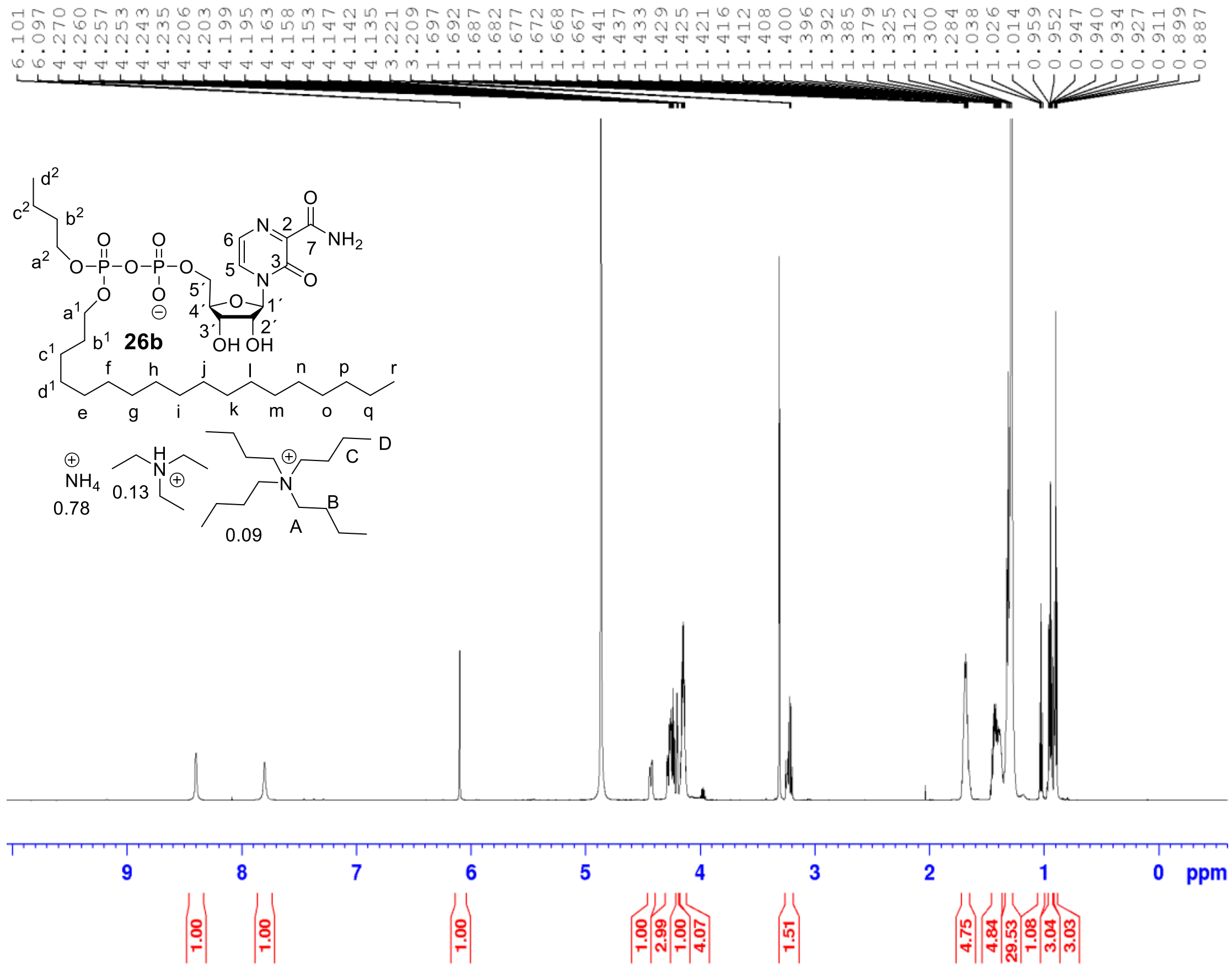

6.101 6.097 4.270 4.260 4.257 4.253 4.243 4.235 4.206 4.203 4.199 4.195 4.163 4.158 4.153 4.147 4.142 4.135 3.221 3.209 1.697 1.692 1.687 1.682 1.677 1.672 1.668 1.667 1.441 1.437 1.433 1.429 1.425 1.421 1.416 1.412 1.408 1.400 1.396 1.392 1.385 1.379 1.325 1.312 1.300 1.284 1.038 1.026 1.014 0.959 0.952 0.947 0.940 0.934 0.927 0.911 0.899 0.887
26b
NH2
OH OH
NH4
0.78
0.13
0.09
A
B
C
D
9
8
7
6
5
4
3
2
1
0
ppm
1.00
1.00
1.00
1.00
2.99
1.00
4.07
1.51
4.75
4.84
29.53
1.08
3.04
3.03

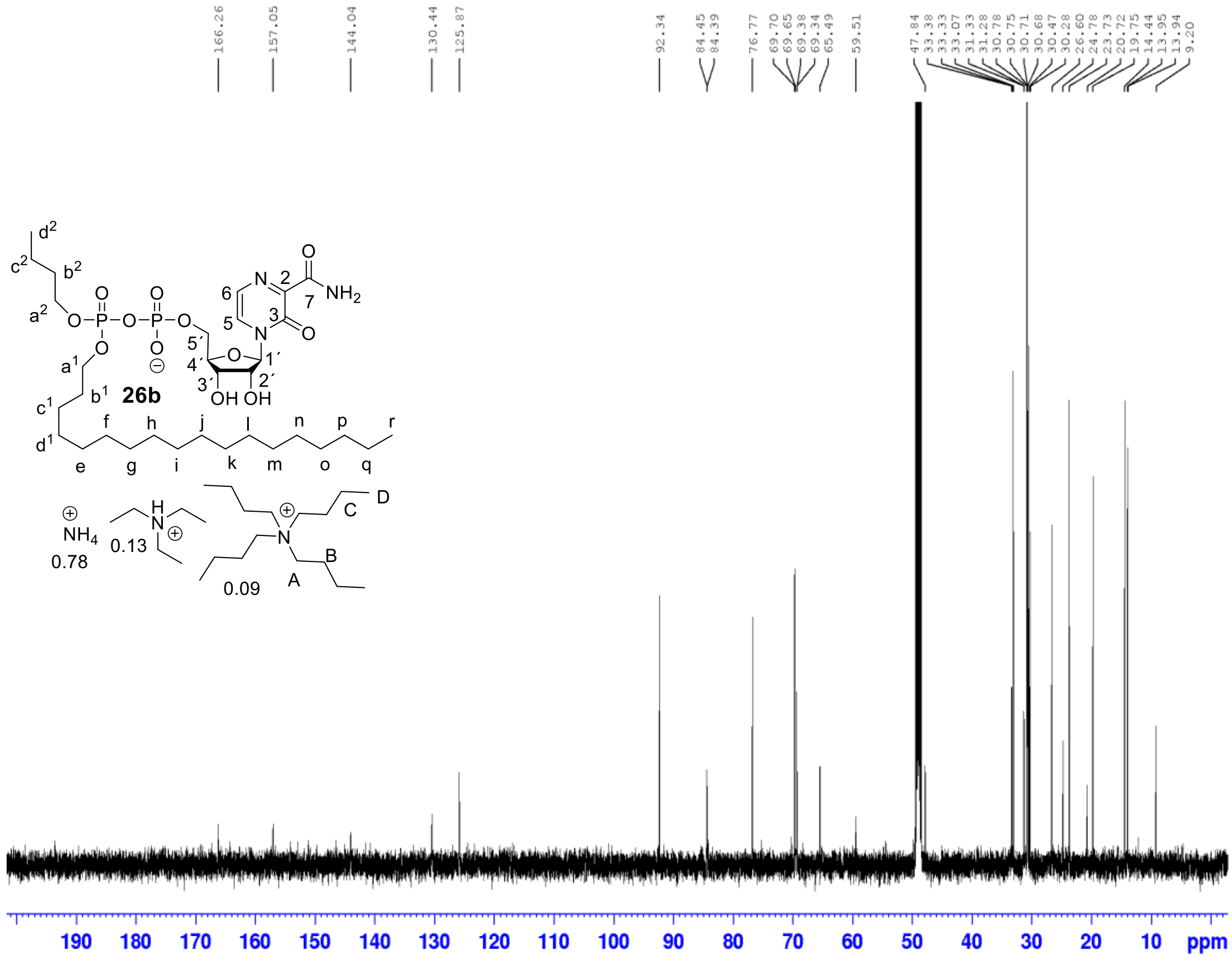
166.26
157.05
144.04
130.44
125.87
92.34
84.45
84.39
76.77
69.70
69.65
69.38
69.34
65.49
59.51
47.84
33.38
33.33
33.07
31.33
31.28
30.78
30.75
30.71
30.68
30.47
30.28
26.60
24.78
23.73
20.72
19.75
14.44
13.95
13.94
9.20
26b
0.78
0.13
0.09
ppm

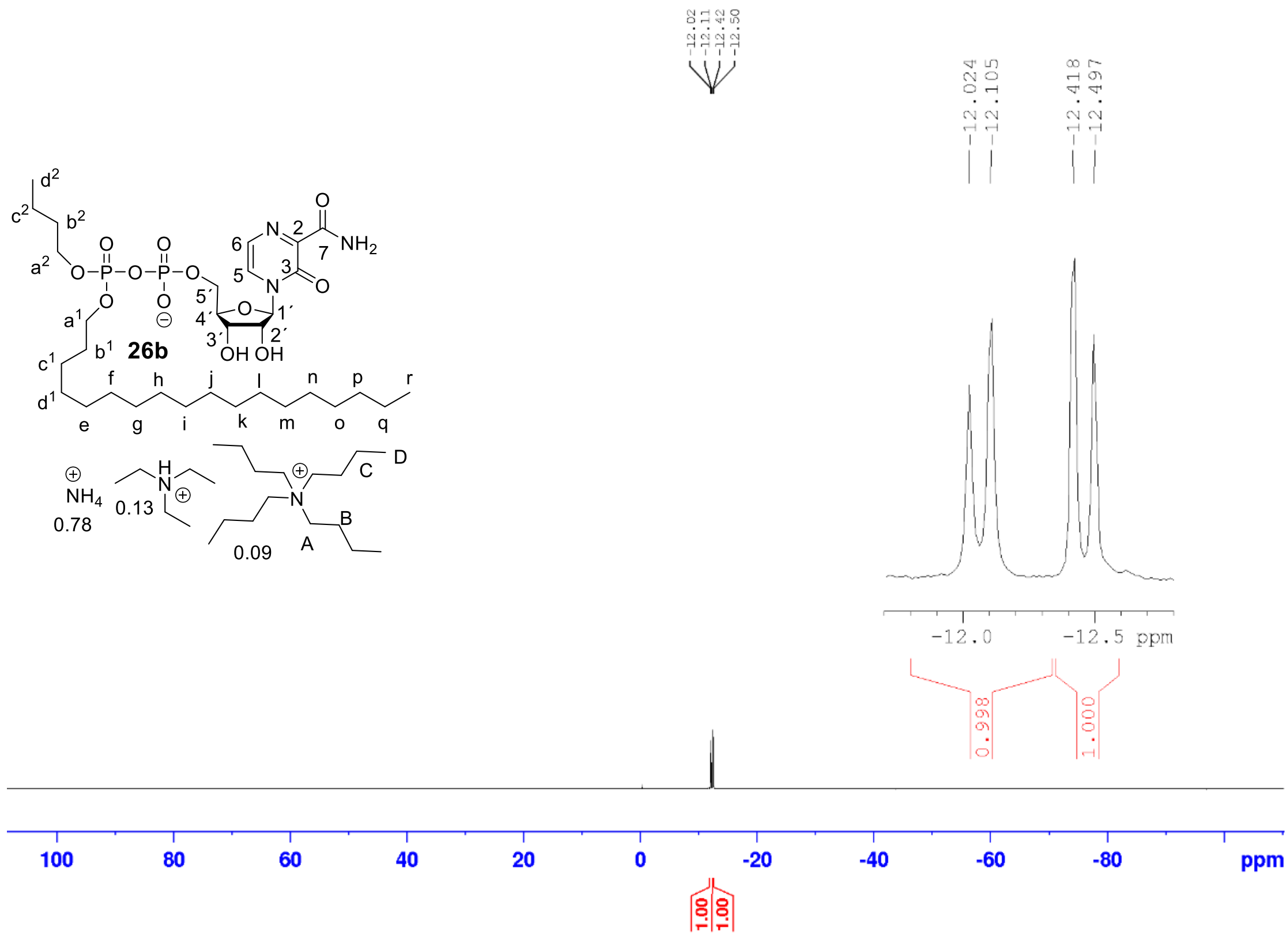
-12.02
-12.11
-12.42
-12.50
-12.024
-12.105
-12.418
-12.497
26b
0.78
0.13
0.09
0.998
1.000
1.00
1.00
ppm

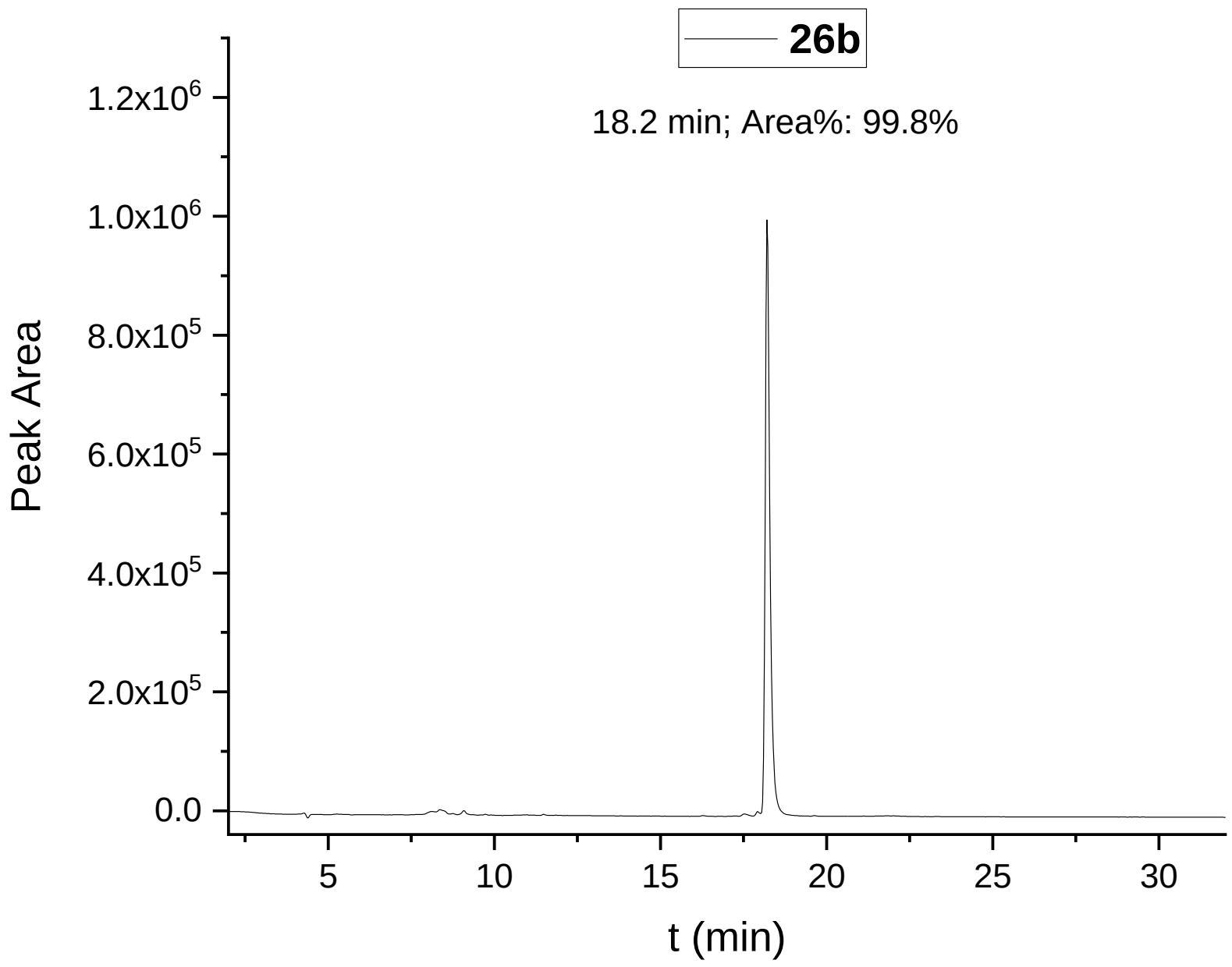

26b
18.2 min; Area%: 99.8%
Peak Area
t (min)

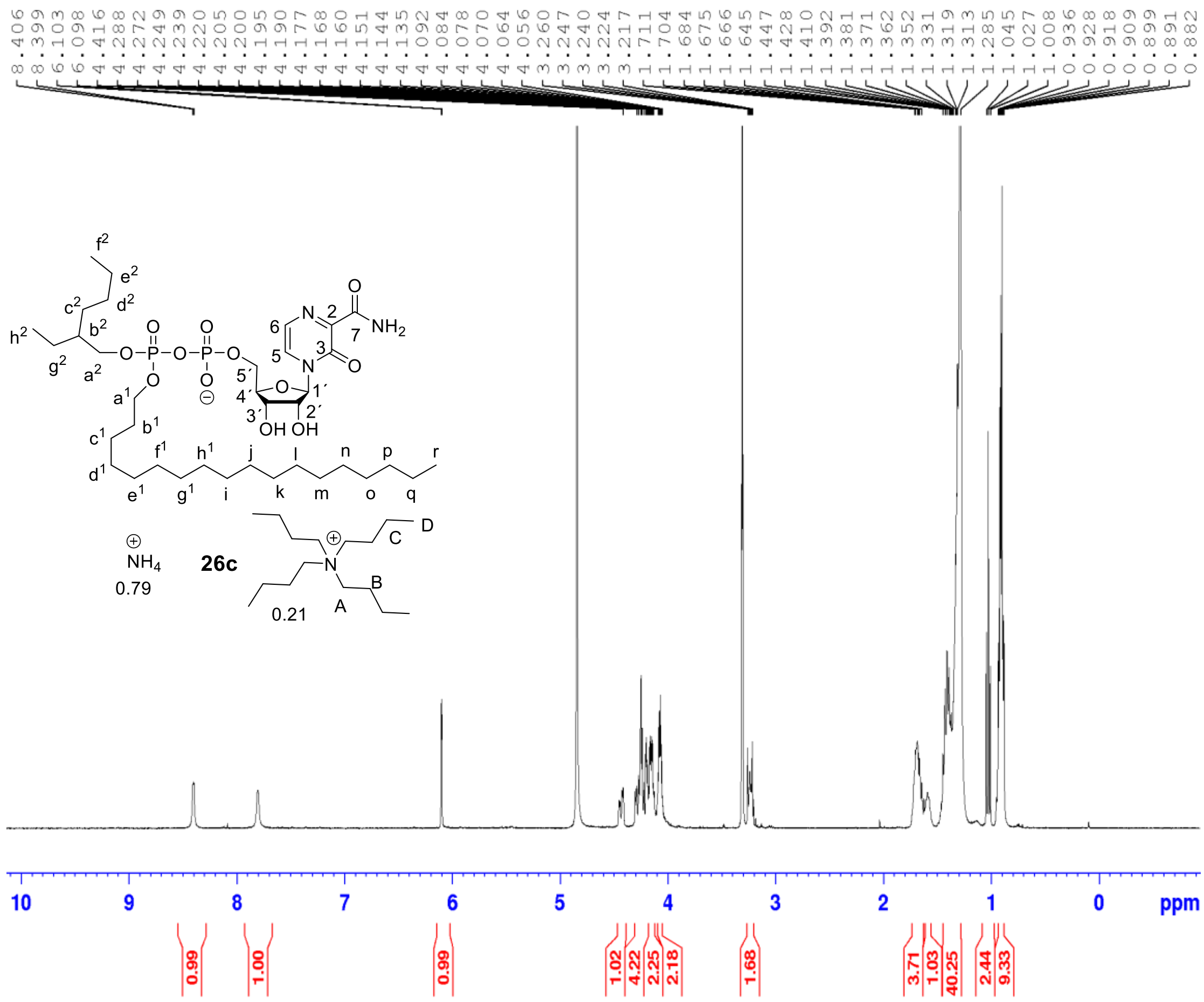

8.406
8.399
6.103
6.098
4.416
4.288
4.272
4.249
4.239
4.220
4.205
4.200
4.195
4.190
4.177
4.168
4.160
4.151
4.144
4.135
4.092
4.084
4.078
4.070
4.064
4.056
3.260
3.247
3.240
3.224
3.217
1.711
1.704
1.684
1.675
1.666
1.645
1.447
1.428
1.410
1.392
1.381
1.371
1.362
1.352
1.331
1.319
1.313
1.285
1.045
1.027
1.008
0.936
0.928
0.918
0.909
0.899
0.891
0.882
NH4
0.79
26c
0.21
ppm
0.99
1.00
0.99
1.02
4.22
2.25
2.18
1.68
3.71
1.03
40.25
2.44
9.33

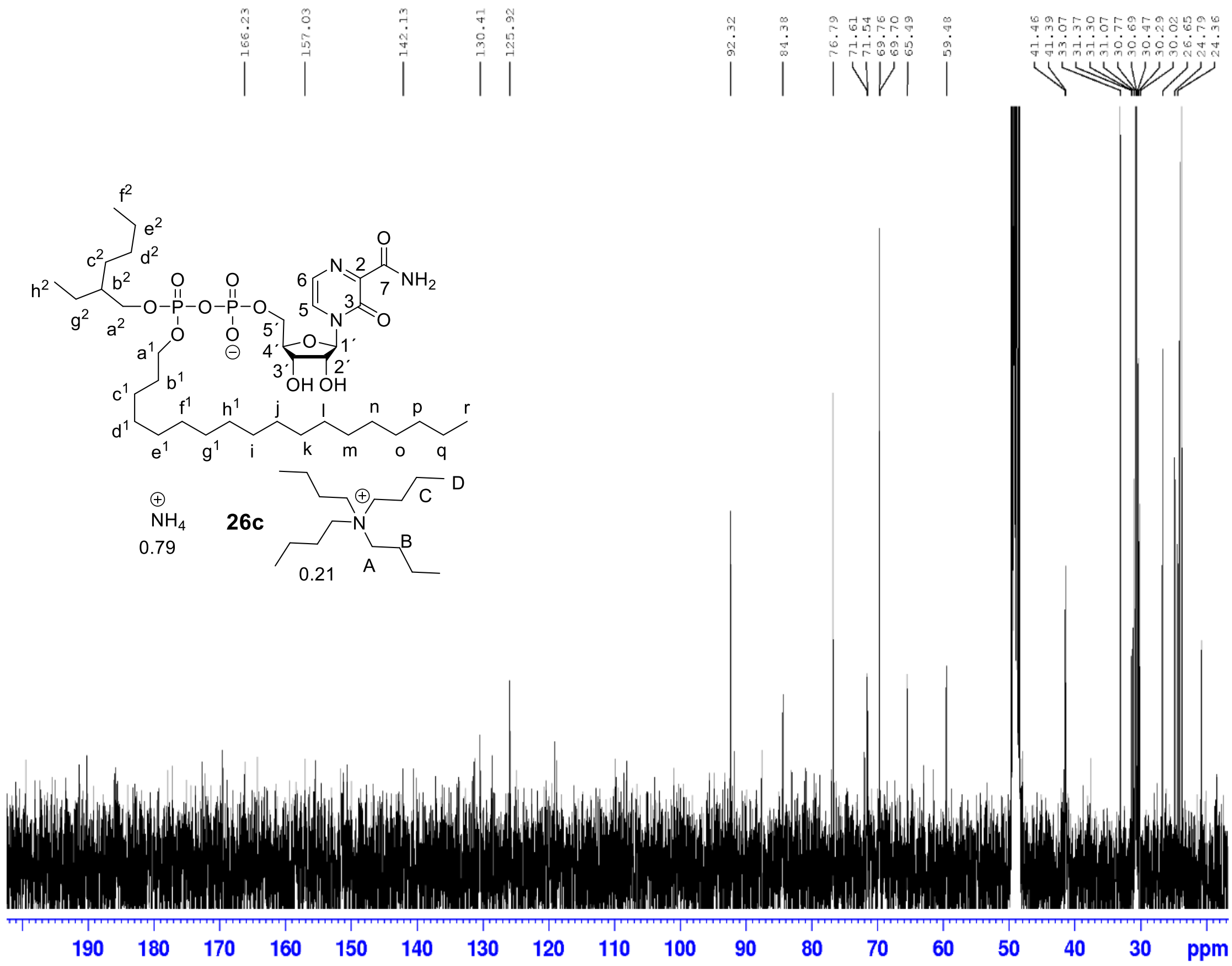
166.23
157.03
142.13
130.41
125.92
92.32
84.38
76.79
71.61
71.54
69.76
69.70
65.49
59.48
41.46
41.39
33.07
31.37
31.30
31.07
30.77
30.69
30.47
30.29
30.02
26.65
24.79
24.36
26c
NH4
0.79
0.21
190 180 170 160 150 140 130 120 110 100 90 80 70 60 50 40 30 ppm

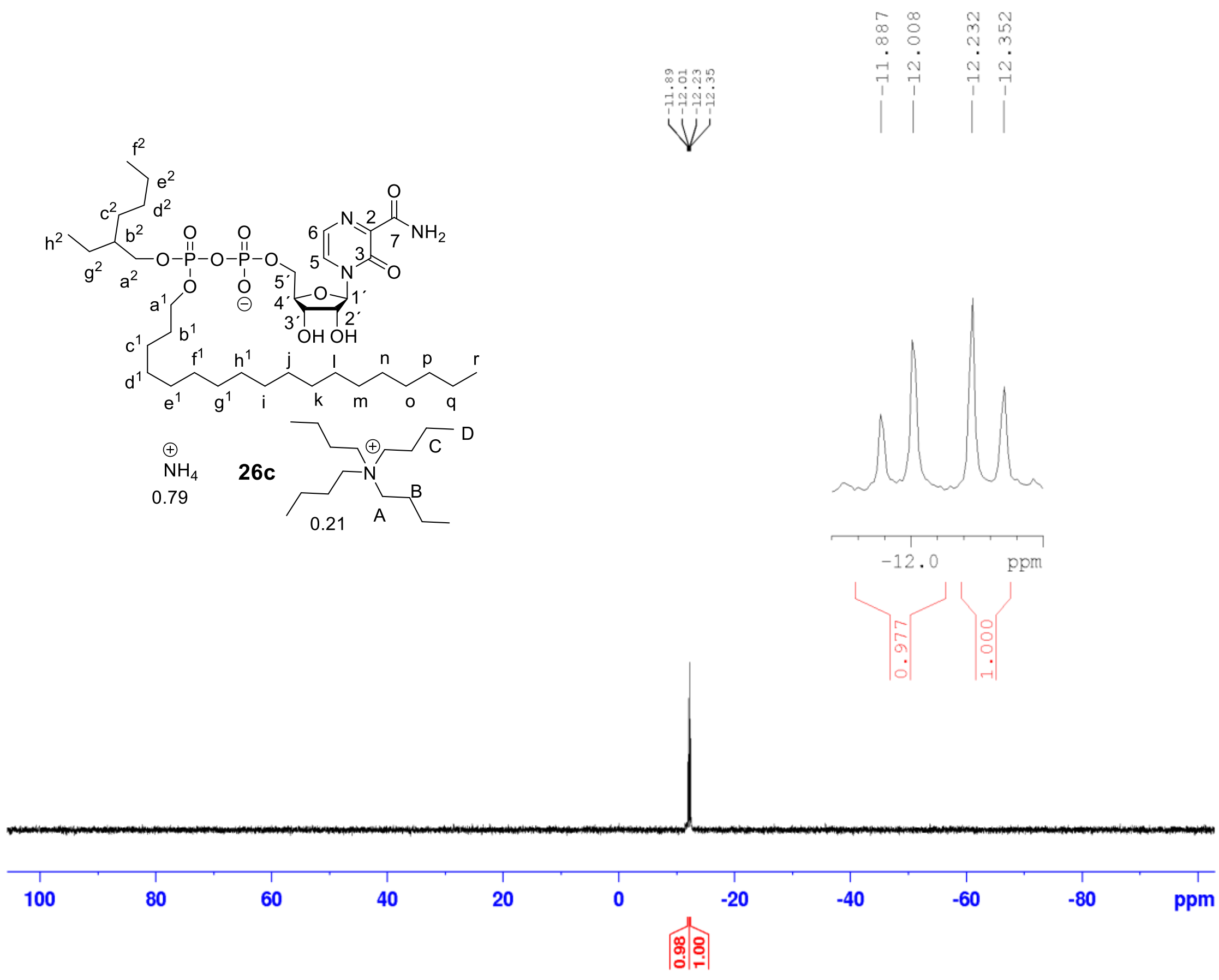
-11.89
-12.01
-12.23
-12.35
-11.887
-12.008
-12.232
-12.352
26c
NH4
0.79
0.21
-12.0 ppm
0.977
1.000
100 80 60 40 20 0 -20 -40 -60 -80 ppm
0.98
1.00

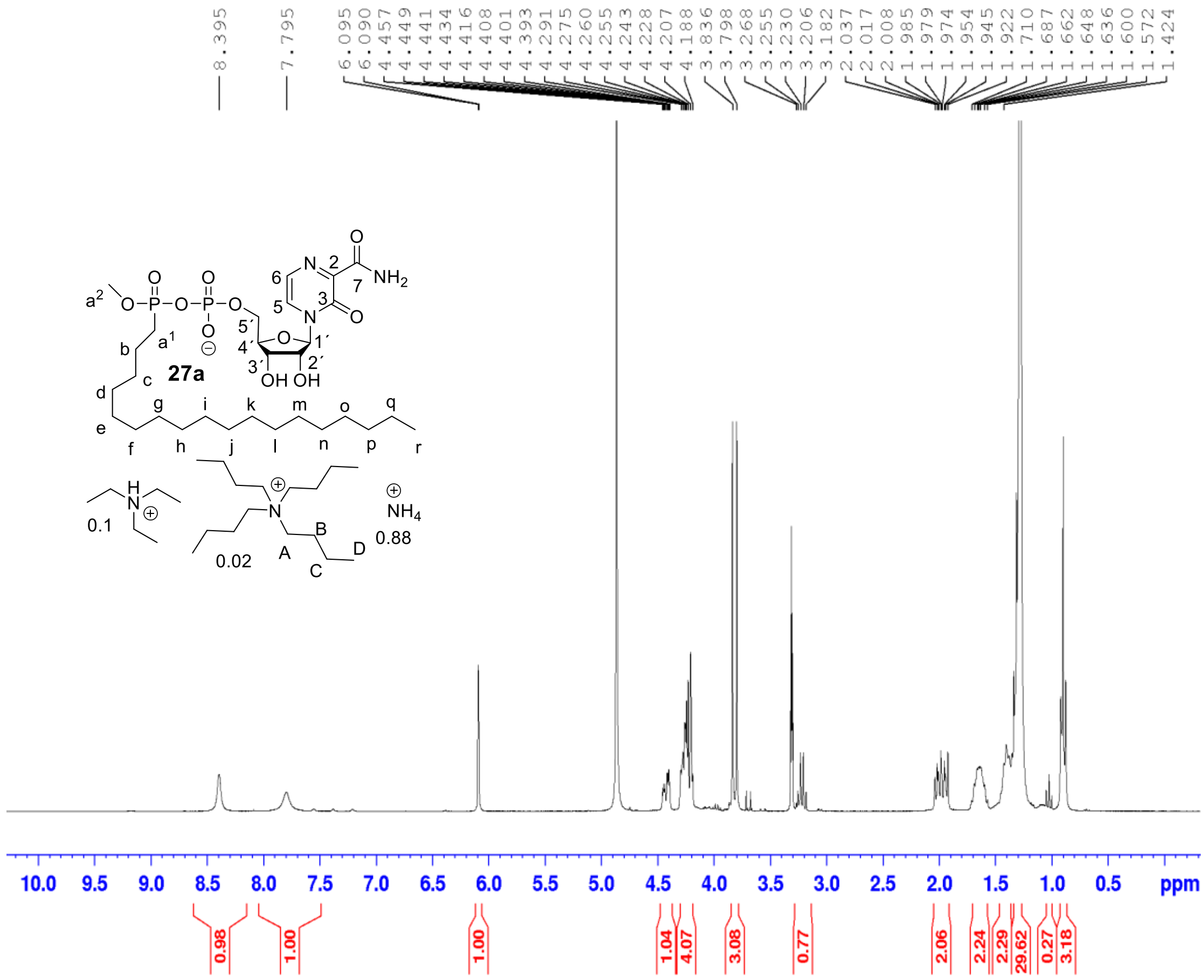
8.395
7.795
6.095
6.090
4.457
4.449
4.441
4.434
4.416
4.408
4.401
4.393
4.291
4.275
4.260
4.255
4.243
4.228
4.207
4.188
3.836
3.798
3.268
3.255
3.230
3.206
3.182
2.037
2.017
2.008
1.985
1.979
1.974
1.954
1.945
1.922
1.710
1.687
1.662
1.648
1.636
1.600
1.572
1.424
27a
0.1
0.02
0.88
10.0 9.5 9.0 8.5 8.0 7.5 7.0 6.5 6.0 5.5 5.0 4.5 4.0 3.5 3.0 2.5 2.0 1.5 1.0 0.5 ppm
0.98
1.00
1.00
1.04
4.07
3.08
0.77
2.06
2.24
2.29
29.62
0.27
3.18

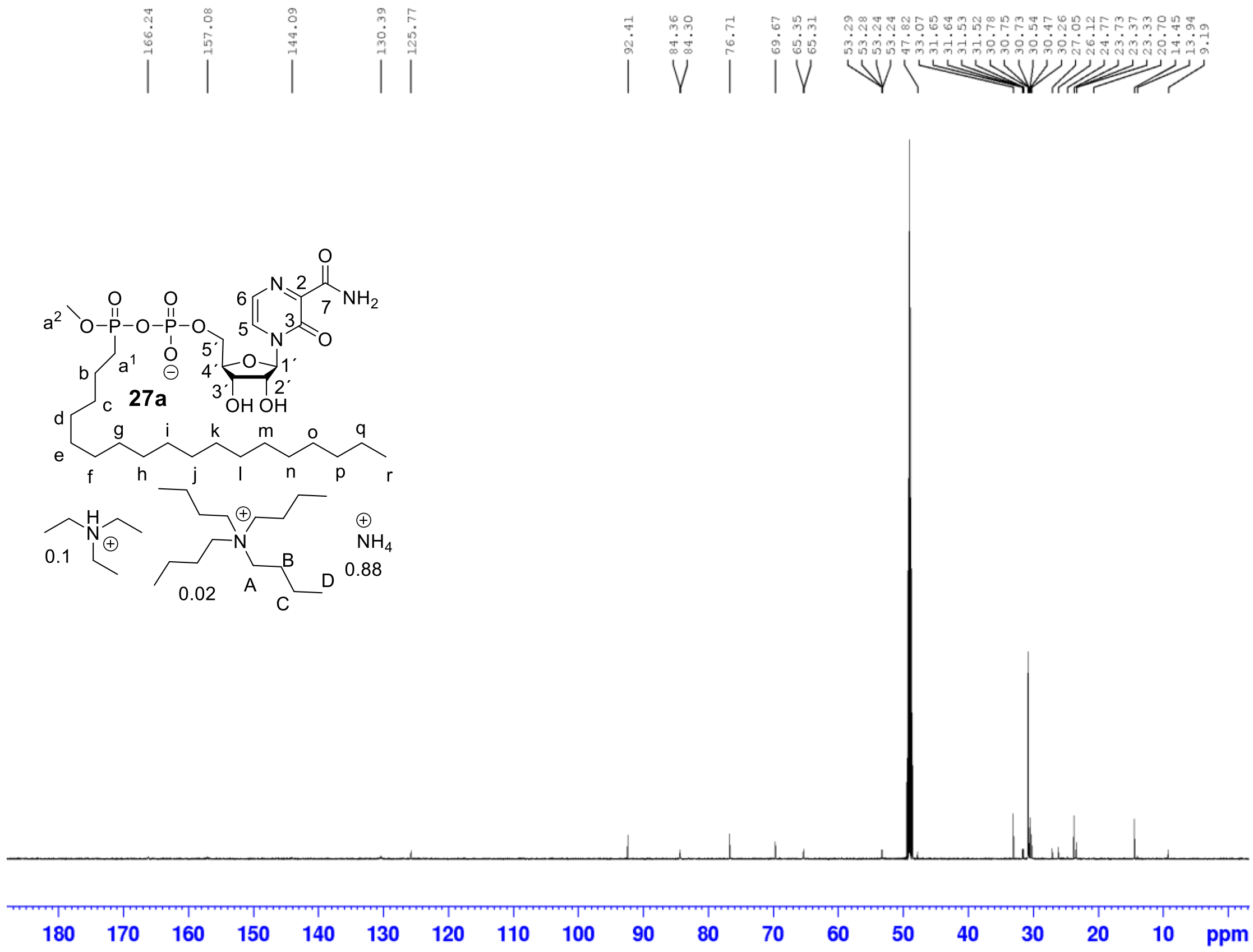
166.24
157.08
144.09
130.39
125.77
92.41
84.36
84.30
76.71
69.67
65.35
65.31
53.29
53.28
53.24
53.24
47.82
33.07
31.65
31.64
31.53
31.52
30.78
30.75
30.73
30.54
30.47
30.26
27.05
26.12
24.77
23.73
23.37
23.33
20.70
14.45
13.94
9.19
27a
0.1
0.02
0.88
180 170 160 150 140 130 120 110 100 90 80 70 60 50 40 30 20 10 ppm

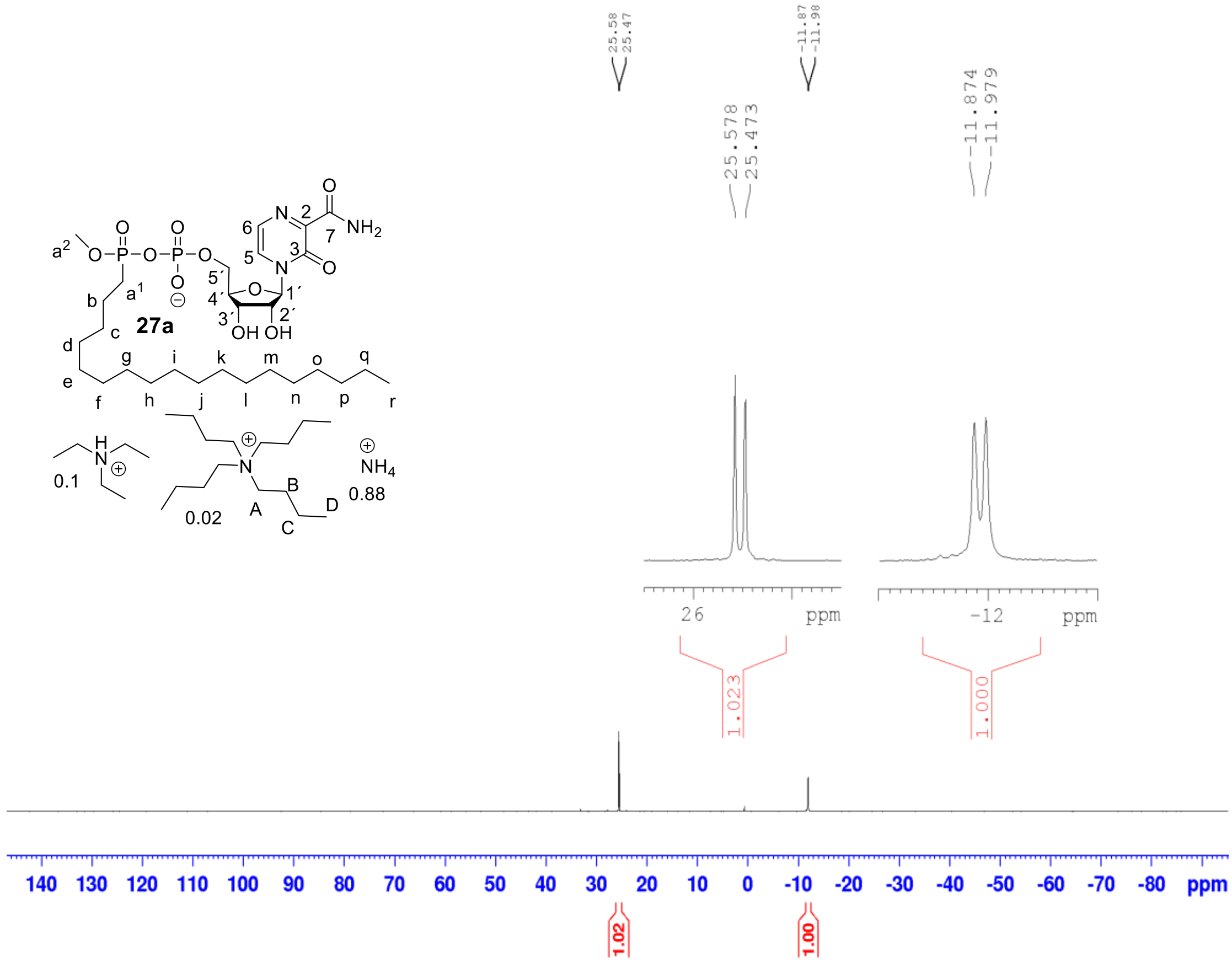
27a
0.1
0.02
0.88
25.58
25.47
-11.87
-11.98
25.578
25.473
-11.874
-11.979
26
-12
ppm
1.023
1.000
140 130 120 110 100 90 80 70 60 50 40 30 20 10 0 -10 -20 -30 -40 -50 -60 -70 -80 ppm
1.02
1.00

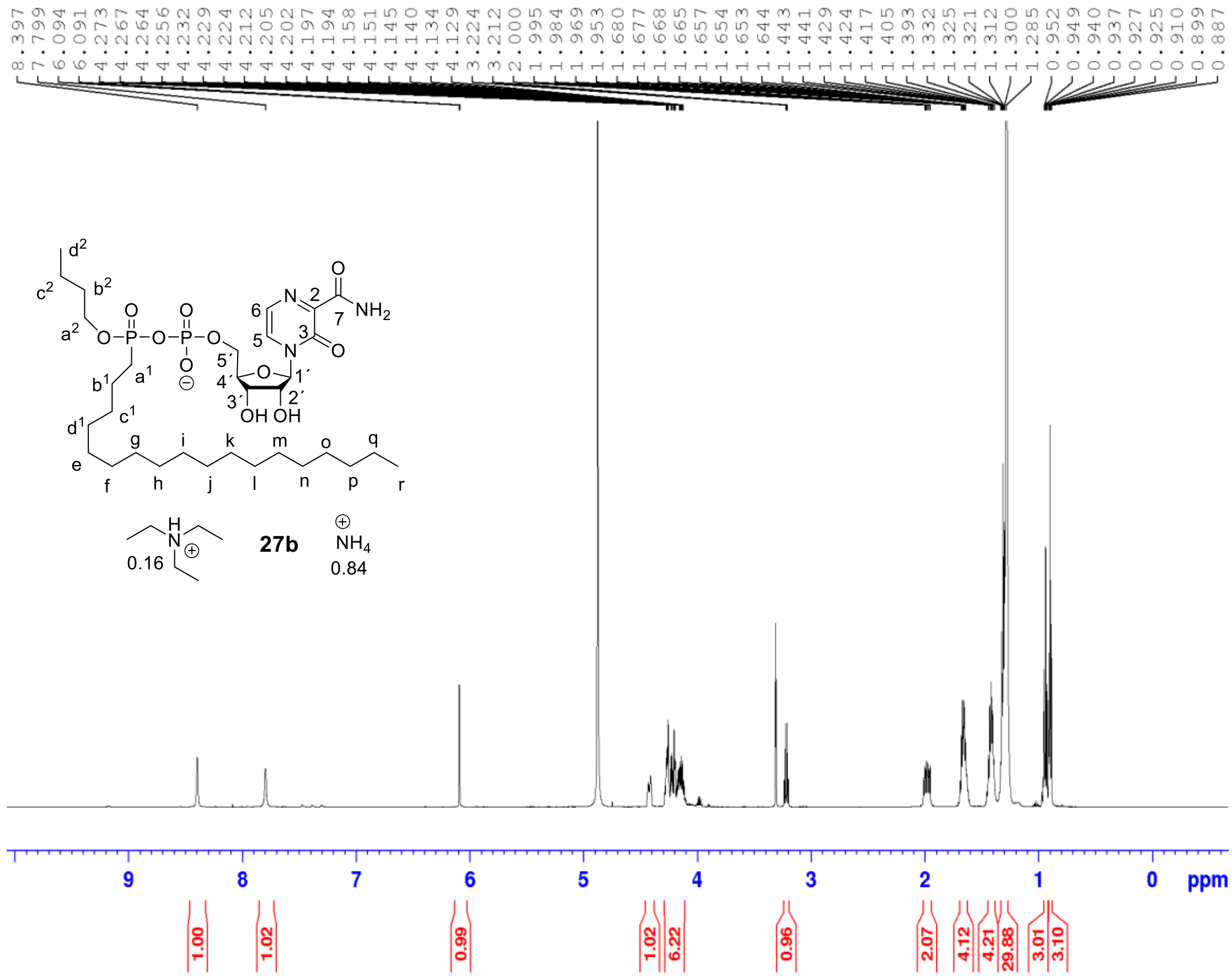
27b
0.16
0.84
8.397
7.799
6.094
6.091
4.273
4.267
4.264
4.256
4.232
4.229
4.224
4.222
4.212
4.205
4.202
4.197
4.194
4.158
4.151
4.145
4.140
4.134
4.129
3.224
3.212
2.000
1.995
1.984
1.969
1.953
1.680
1.677
1.668
1.665
1.657
1.654
1.653
1.644
1.443
1.441
1.429
1.424
1.417
1.405
1.393
1.332
1.325
1.321
1.312
1.300
1.285
0.952
0.949
0.940
0.937
0.927
0.925
0.910
0.899
0.887
9 8 7 6 5 4 3 2 1 0 ppm
1.00
1.02
0.99
1.02
6.22
0.96
2.07
4.12
4.21
29.88
3.01
3.10

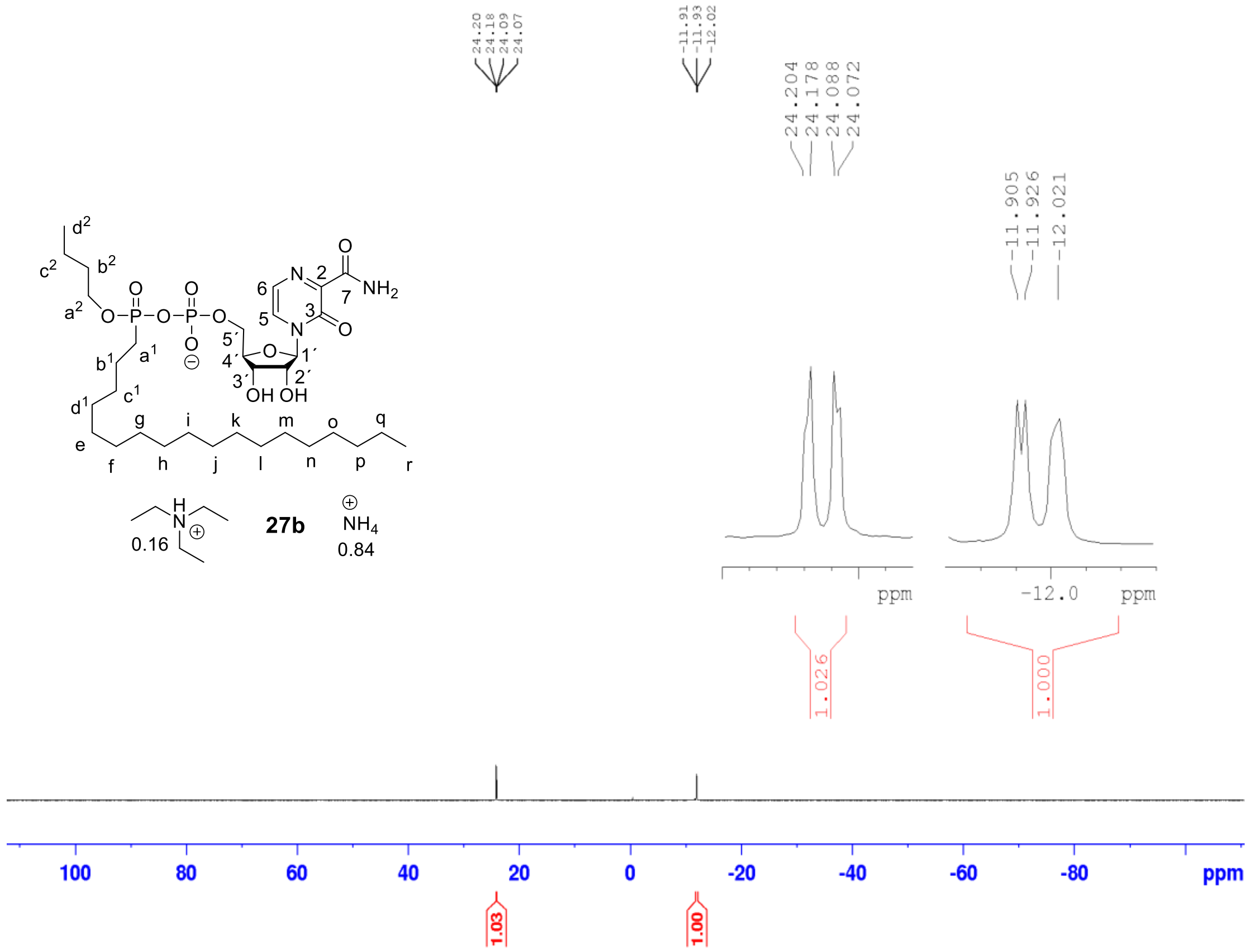

24.20
24.18
24.09
24.07
-11.91
-11.93
-12.02
24.204
24.178
24.088
24.072
-11.905
-11.926
-12.021
ppm
-12.0
ppm
1.026
1.000
27b
0.16
0.84
100 80 60 40 20 0 -20 -40 -60 -80 ppm
1.03
1.00

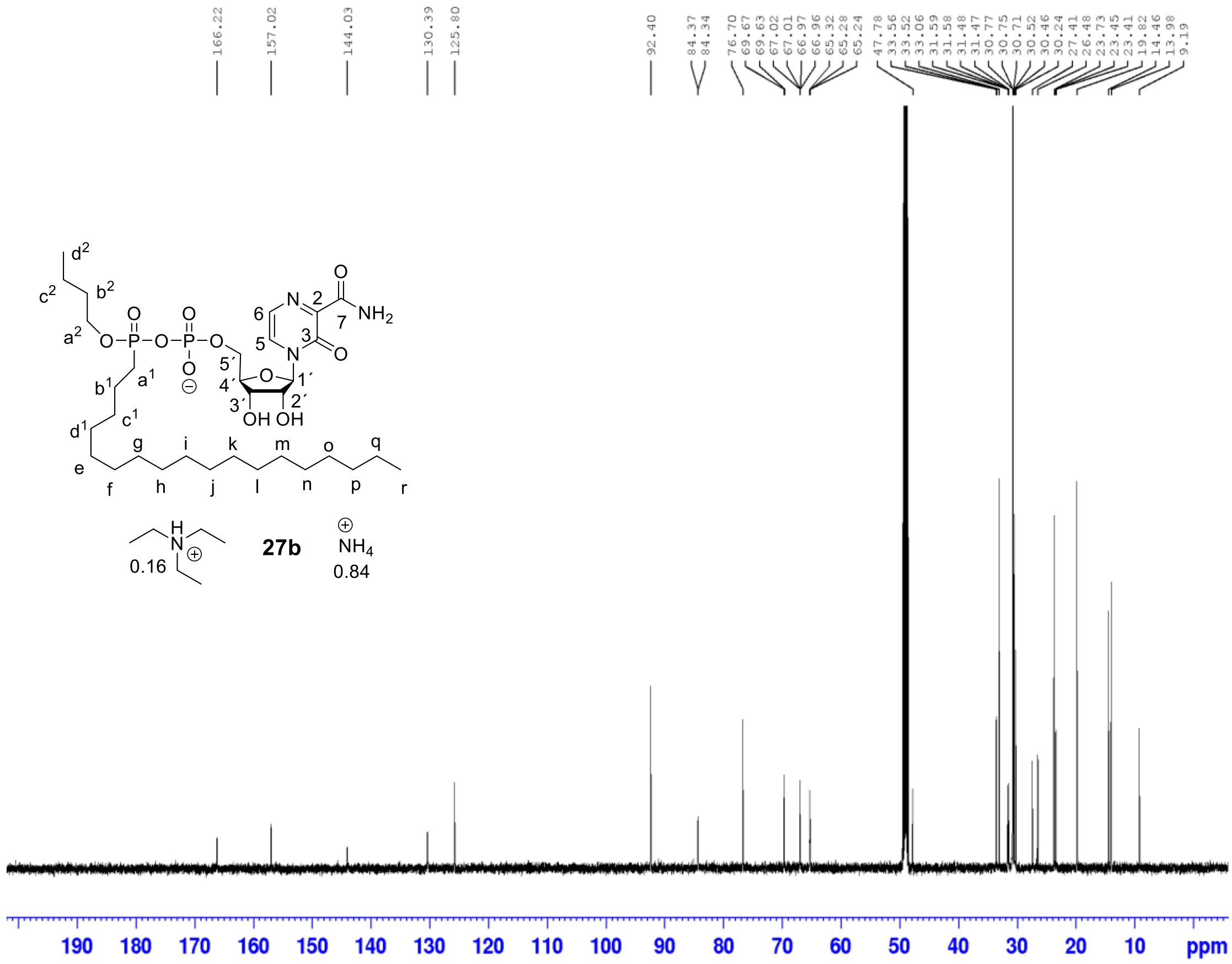

166.22
157.02
144.03
130.39
125.80
92.40
84.37
84.34
76.70
69.67
69.63
67.02
67.01
66.97
66.96
65.32
65.28
65.24
47.78
33.56
33.52
33.06
31.59
31.58
31.48
31.47
30.77
30.75
30.71
30.52
30.46
30.24
27.41
26.48
23.73
23.45
23.41
19.82
14.46
13.98
9.19
27b
0.16
0.84
190 180 170 160 150 140 130 120 110 100 90 80 70 60 50 40 30 20 10 ppm

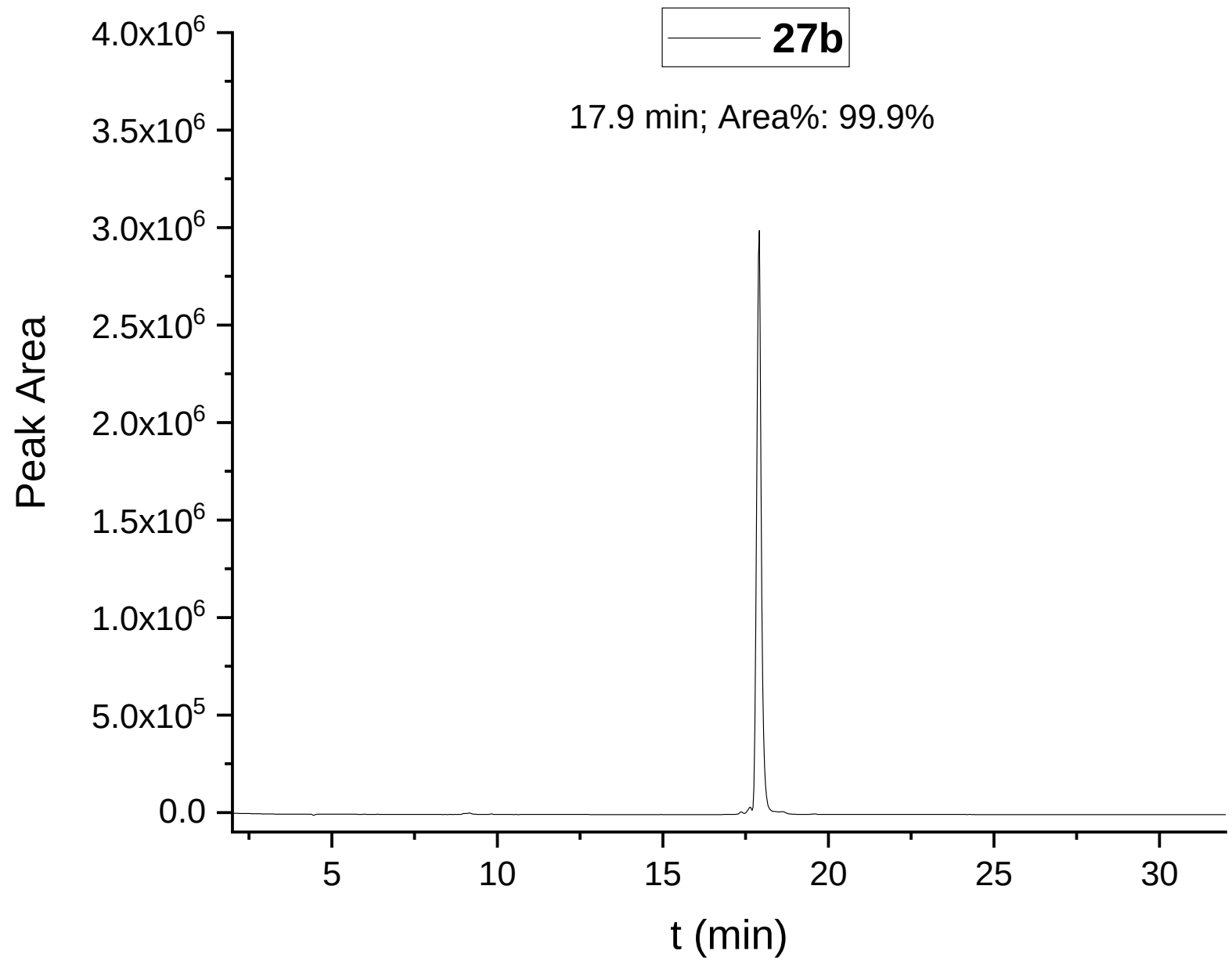

27b
17.9 min; Area%: 99.9%
Peak Area
t (min)

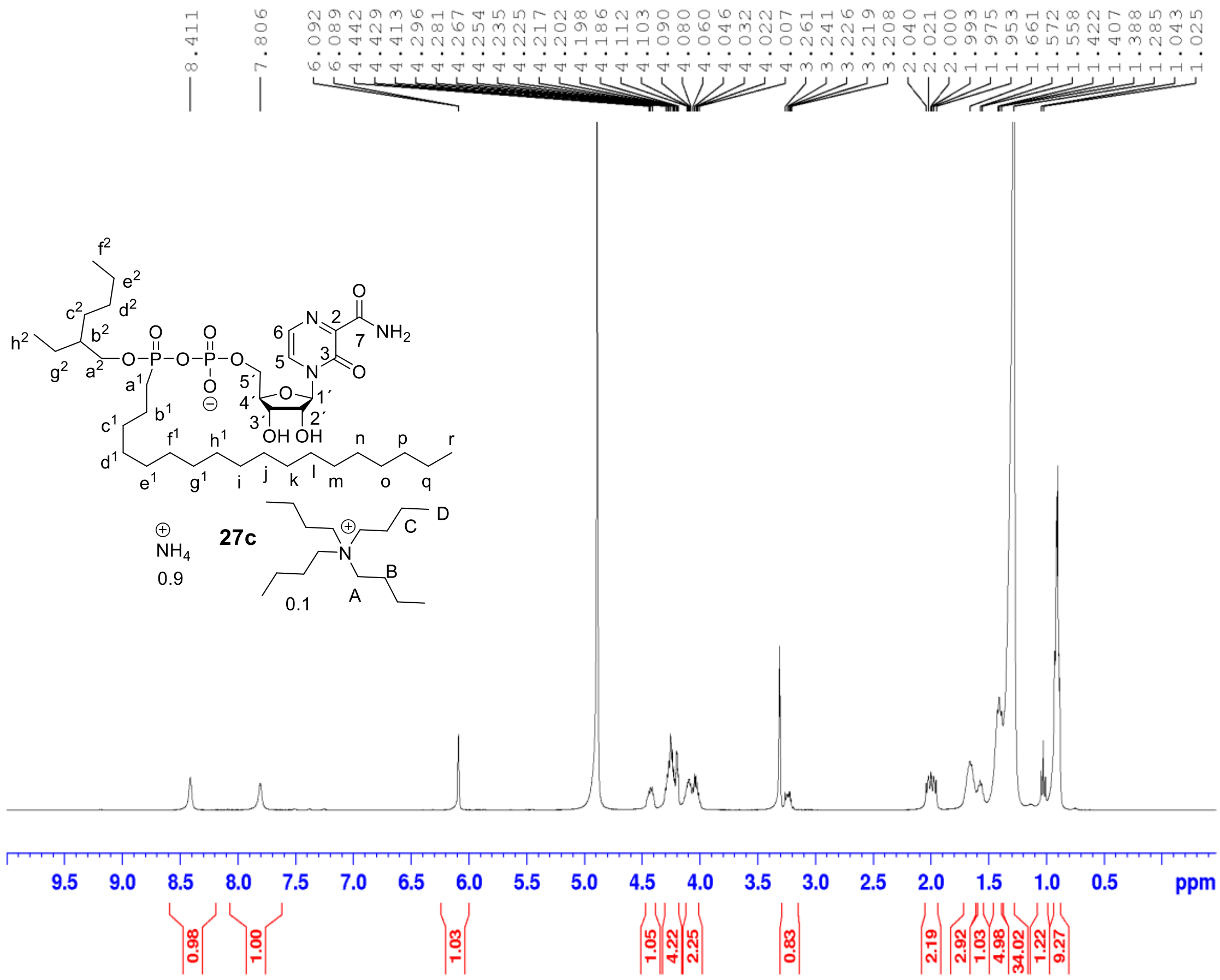

27c
0.9
0.1
ppm

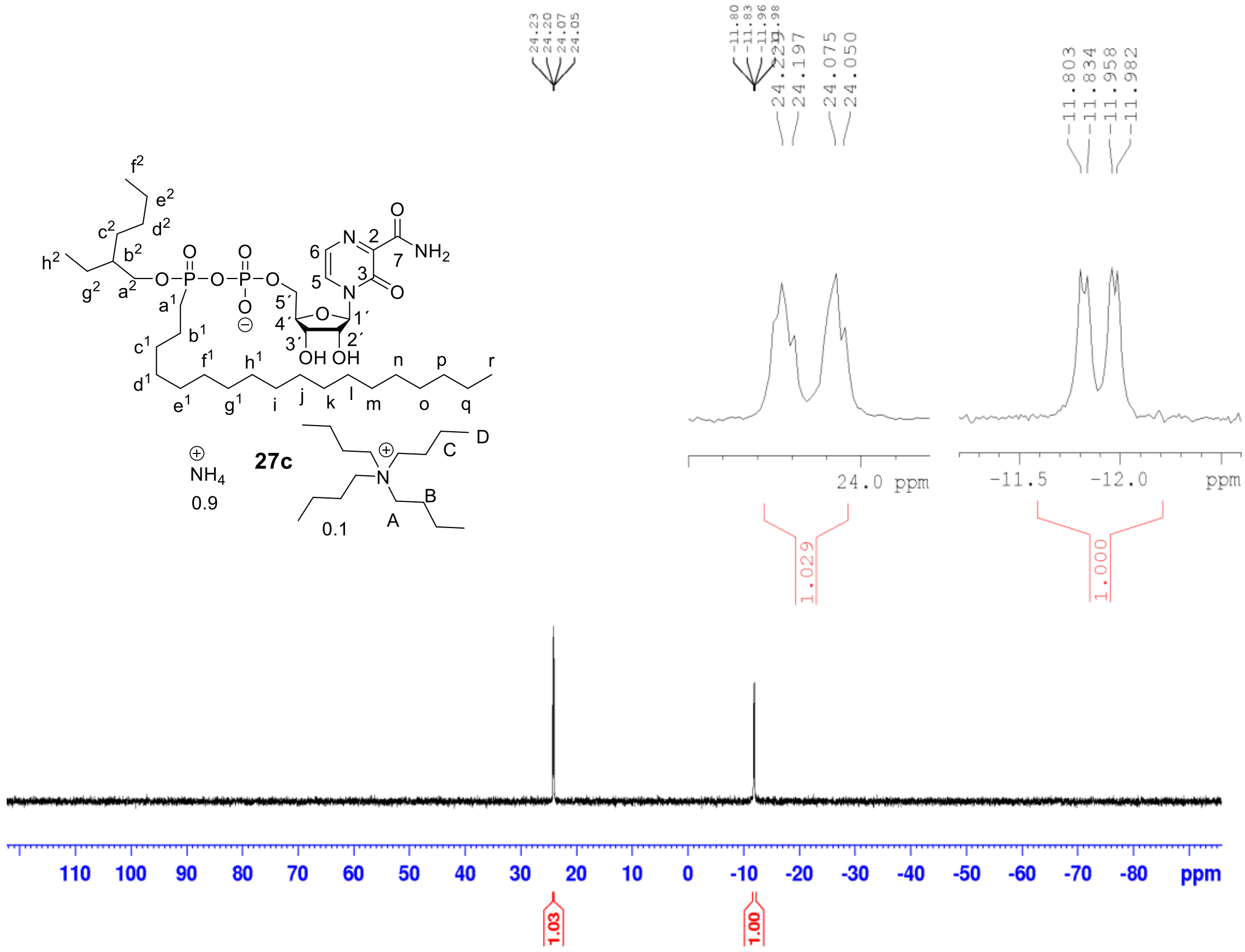

27c
NH4
0.9
0.1
A
B
C
D
24.229
24.197
24.075
24.050
-11.803
-11.834
-11.958
-11.982
24.0 ppm
-11.5
-12.0
ppm
1.029
1.000
110 100 90 80 70 60 50 40 30 20 10 0 -10 -20 -30 -40 -50 -60 -70 -80 ppm
1.03
1.00

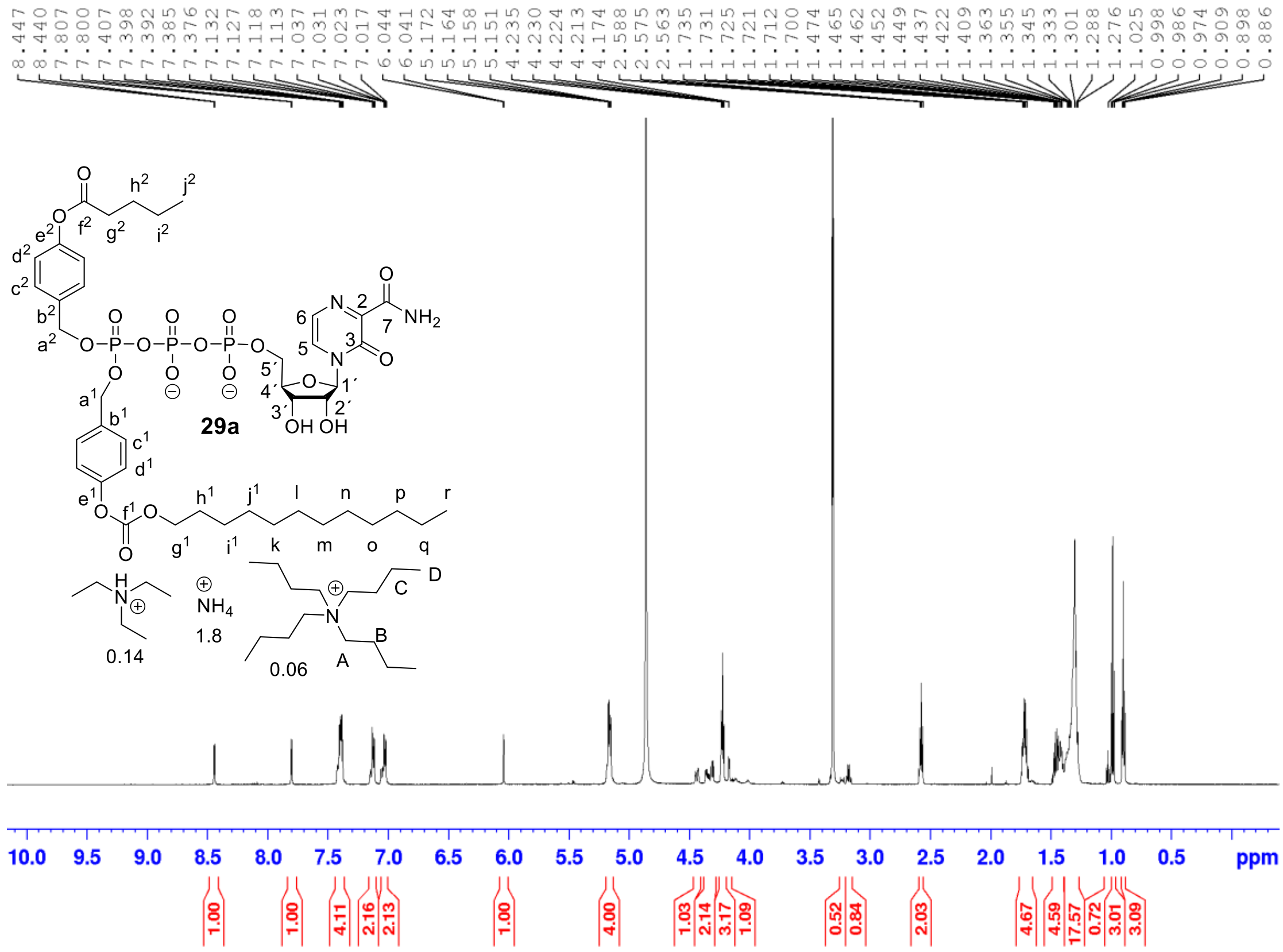

8.447 8.440 7.807 7.800 7.407 7.398 7.392 7.385 7.376 7.132 7.127 7.118 7.113 7.037 7.031 7.023 7.017 6.044 6.041 5.172 5.164 5.158 5.151 4.235 4.230 4.224 4.213 4.174 2.588 2.575 2.563 1.735 1.731 1.725 1.721 1.712 1.700 1.474 1.465 1.462 1.452 1.449 1.437 1.422 1.409 1.363 1.355 1.345 1.333 1.301 1.288 1.276 1.025 0.998 0.986 0.974 0.909 0.898 0.886
29a
0.14
NH4
1.8
0.06
A
B
C
D
10.0 9.5 9.0 8.5 8.0 7.5 7.0 6.5 6.0 5.5 5.0 4.5 4.0 3.5 3.0 2.5 2.0 1.5 1.0 0.5 ppm
1.00 1.00 4.11 2.16 2.13 1.00 4.00 1.03 2.14 3.17 1.09 0.52 0.84 2.03 4.67 4.59 17.57 0.72 3.01 3.09

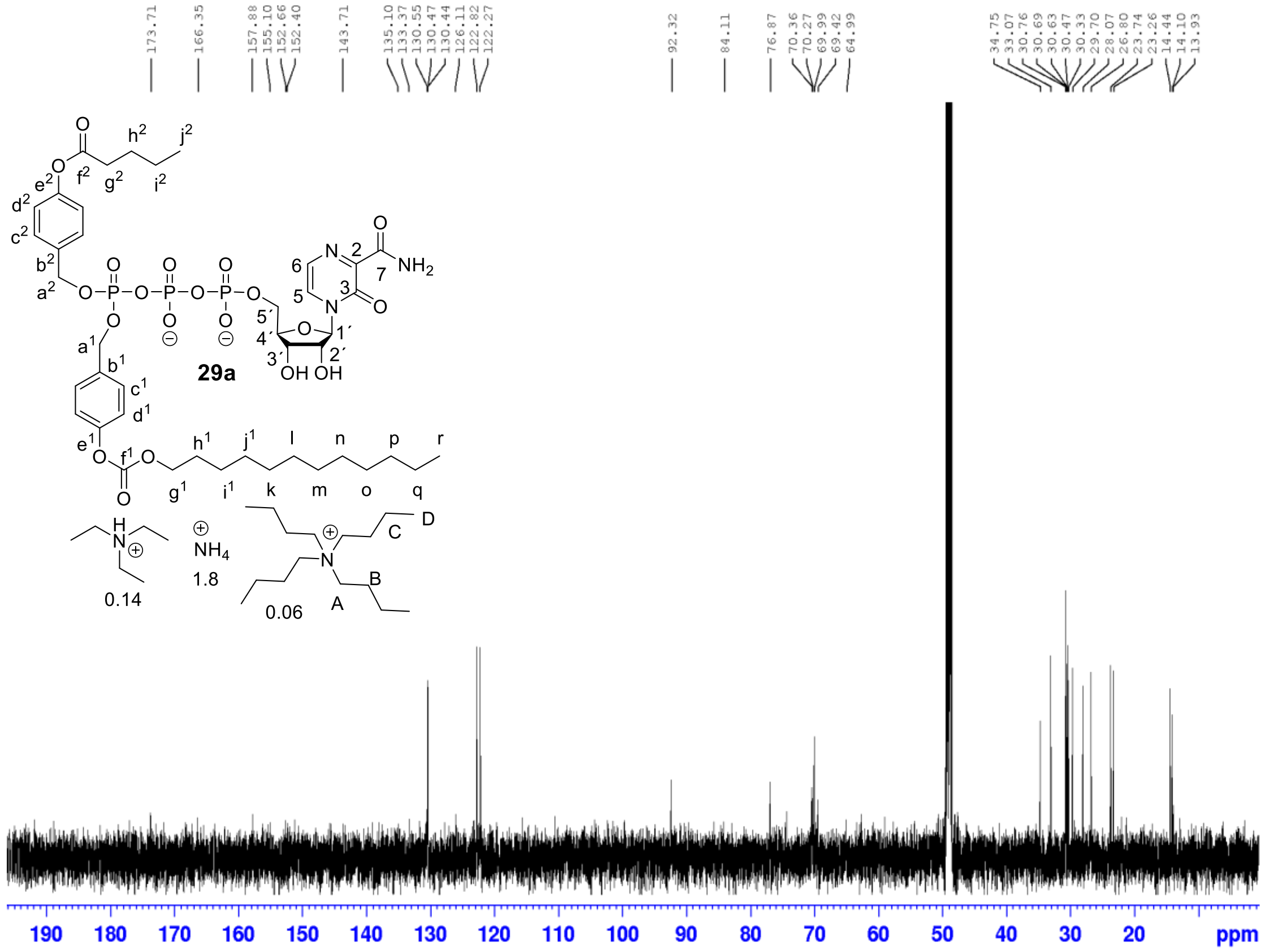

173.71
166.35
157.88
155.10
152.66
152.40
143.71
135.10
133.37
130.55
130.47
130.44
126.11
122.82
122.27
92.32
84.11
76.87
70.36
70.27
69.99
69.42
64.99
34.75
33.07
30.76
30.69
30.63
30.47
30.33
29.70
28.07
26.80
23.74
23.26
14.44
14.10
13.93
29a
NH2
OH OH
0.14
NH4
1.8
0.06
ppm

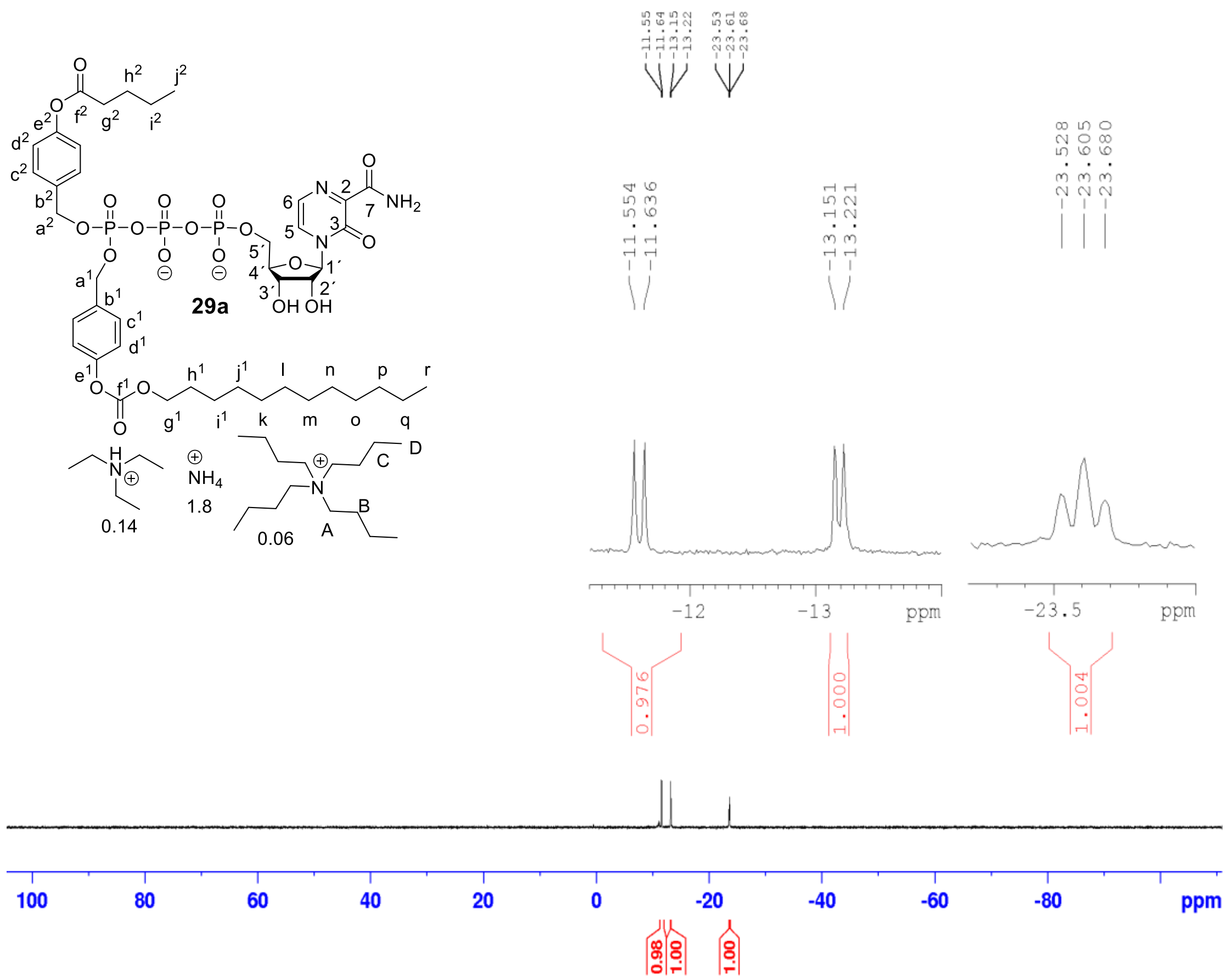

-11.55
-11.64
-13.15
-13.22
-23.53
-23.61
-23.68
-11.554
-11.636
-13.151
-13.221
-23.528
-23.605
-23.680
29a
NH2
OH OH
0.14
NH4
1.8
0.06
-12
-13
ppm
-23.5
ppm
0.976
1.000
1.004
ppm
0.98
1.00
1.00

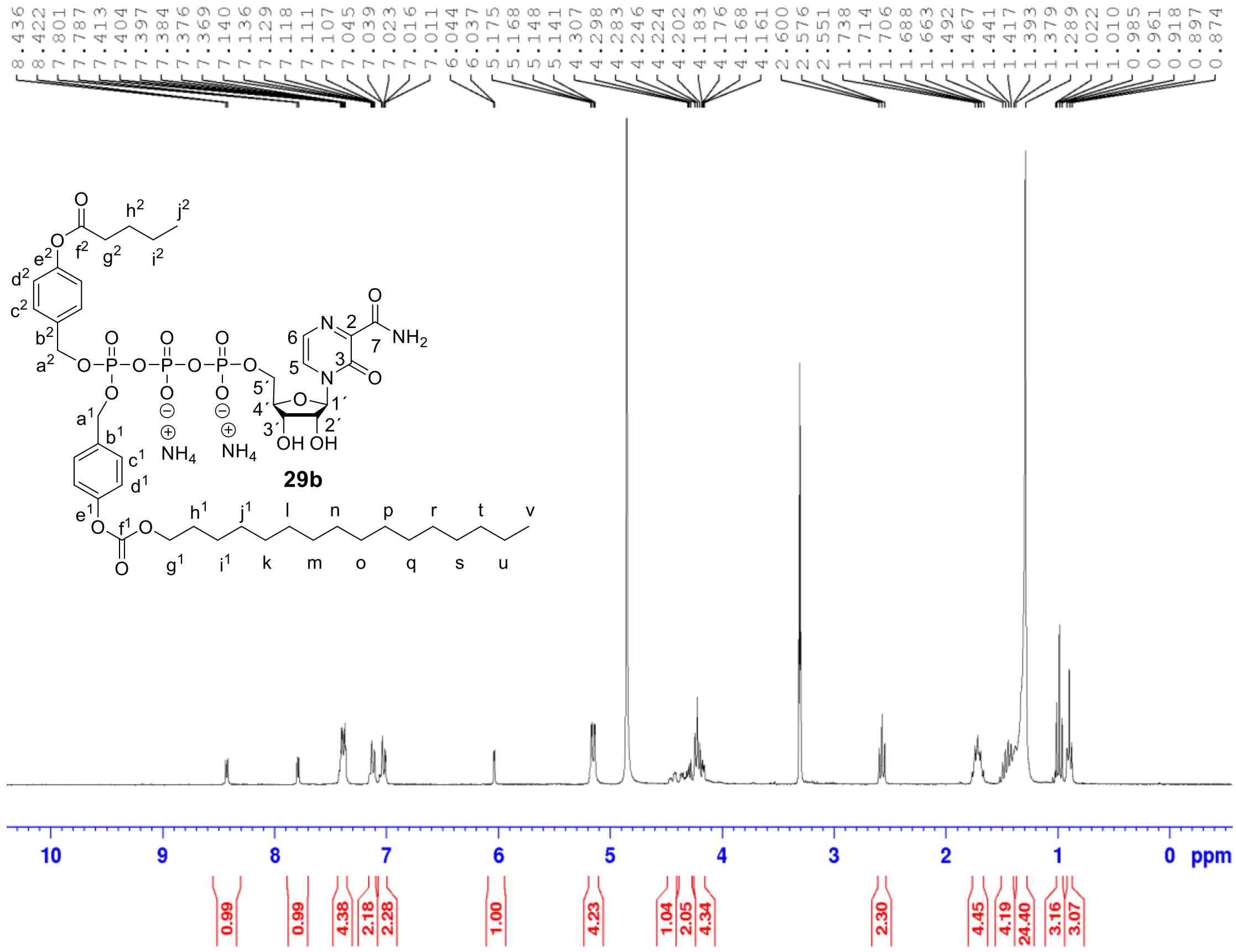
8.436 8.422 7.801 7.787 7.413 7.404 7.397 7.384 7.376 7.369 7.140 7.136 7.129 7.118 7.111 7.107 7.045 7.039 7.023 7.016 7.011 6.044 6.037 5.175 5.168 5.148 5.141 4.307 4.298 4.283 4.246 4.224 4.202 4.183 4.176 4.168 4.161 2.600 2.576 2.551 1.738 1.714 1.706 1.688 1.663 1.492 1.467 1.441 1.417 1.393 1.379 1.289 1.022 1.010 0.985 0.961 0.918 0.897 0.874
29b
10 9 8 7 6 5 4 3 2 1 0 ppm
0.99 0.99 4.38 2.18 2.28 1.00 4.23 1.04 2.05 4.34 2.30 4.45 4.19 24.40 3.16 3.07

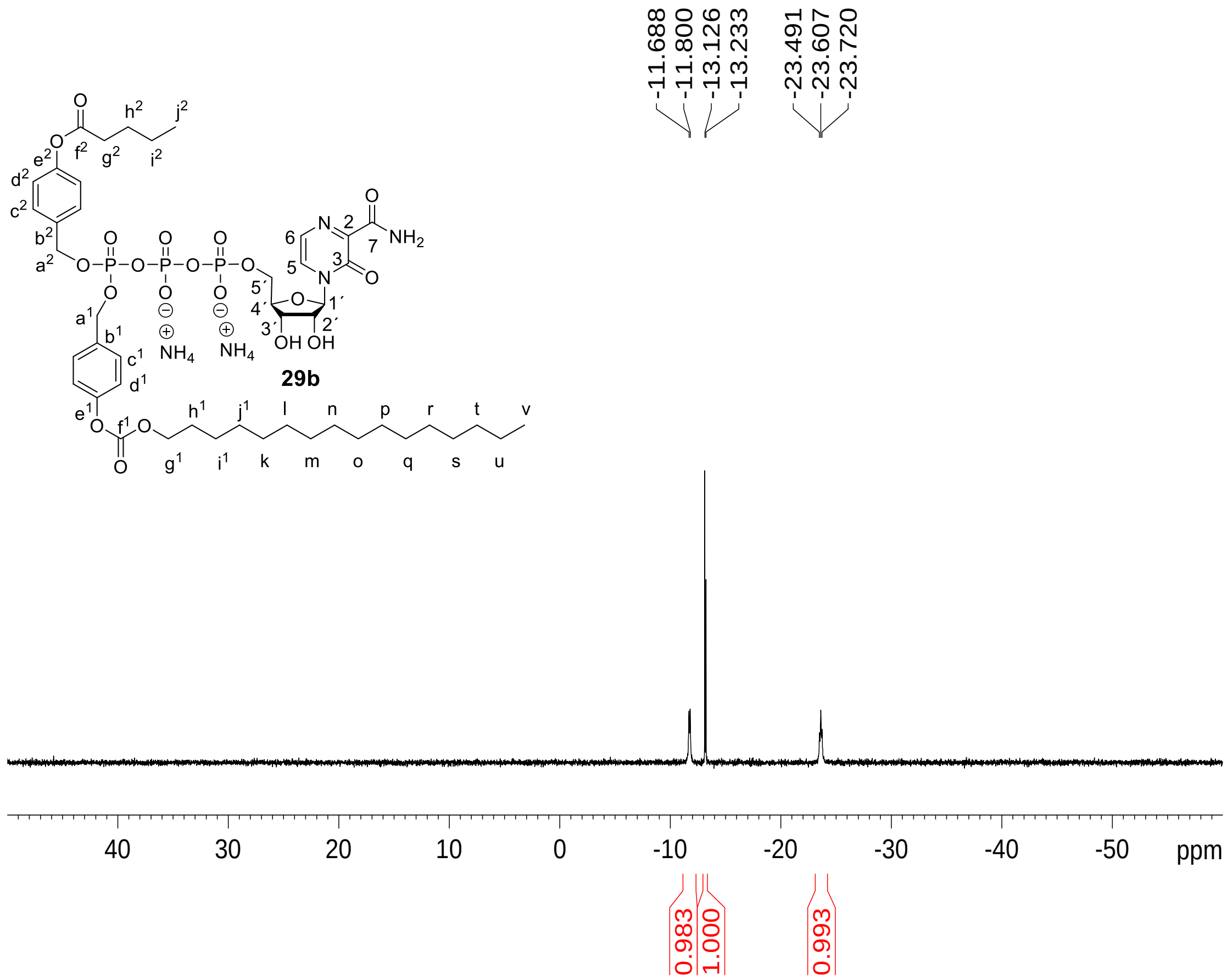
-11.688 -11.800 -13.126 -13.233 -23.491 -23.607 -23.720
29b
40 30 20 10 0 -10 -20 -30 -40 -50 ppm
0.983 1.000 0.993

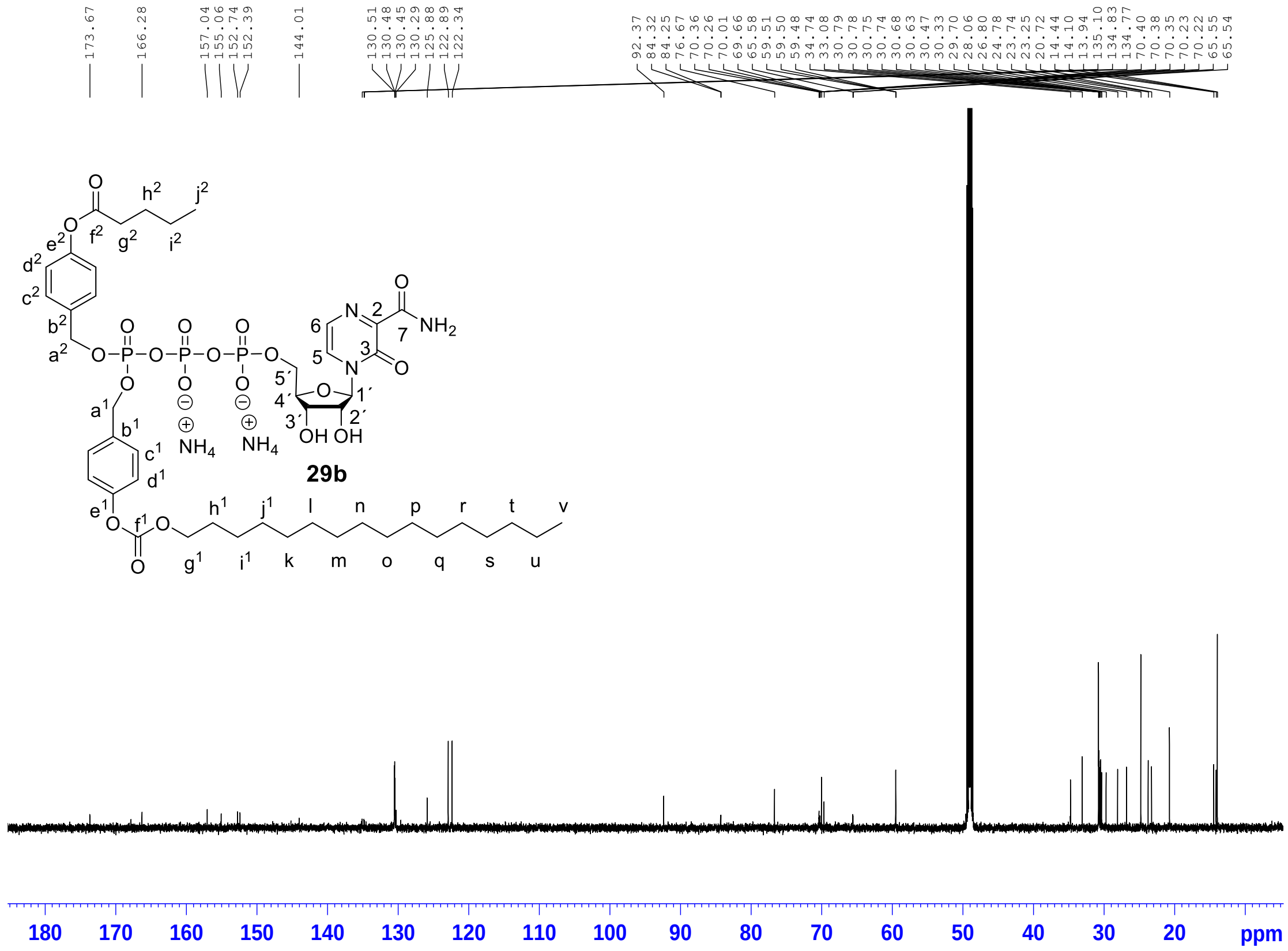
173.67
166.28
157.04
155.06
152.74
152.39
144.01
130.51
130.48
130.45
130.29
125.88
122.89
122.34
92.37
84.32
84.25
76.67
70.36
70.26
70.01
69.66
65.58
59.51
59.50
59.48
34.74
33.08
30.79
30.78
30.75
30.74
30.68
30.63
30.47
30.33
29.70
28.06
26.80
24.78
23.74
23.25
20.72
14.44
14.10
13.94
135.10
134.83
134.77
70.40
70.38
70.35
70.23
70.22
65.55
65.54
29b
180
170
160
150
140
130
120
110
100
90
80
70
60
50
40
30
20
ppm

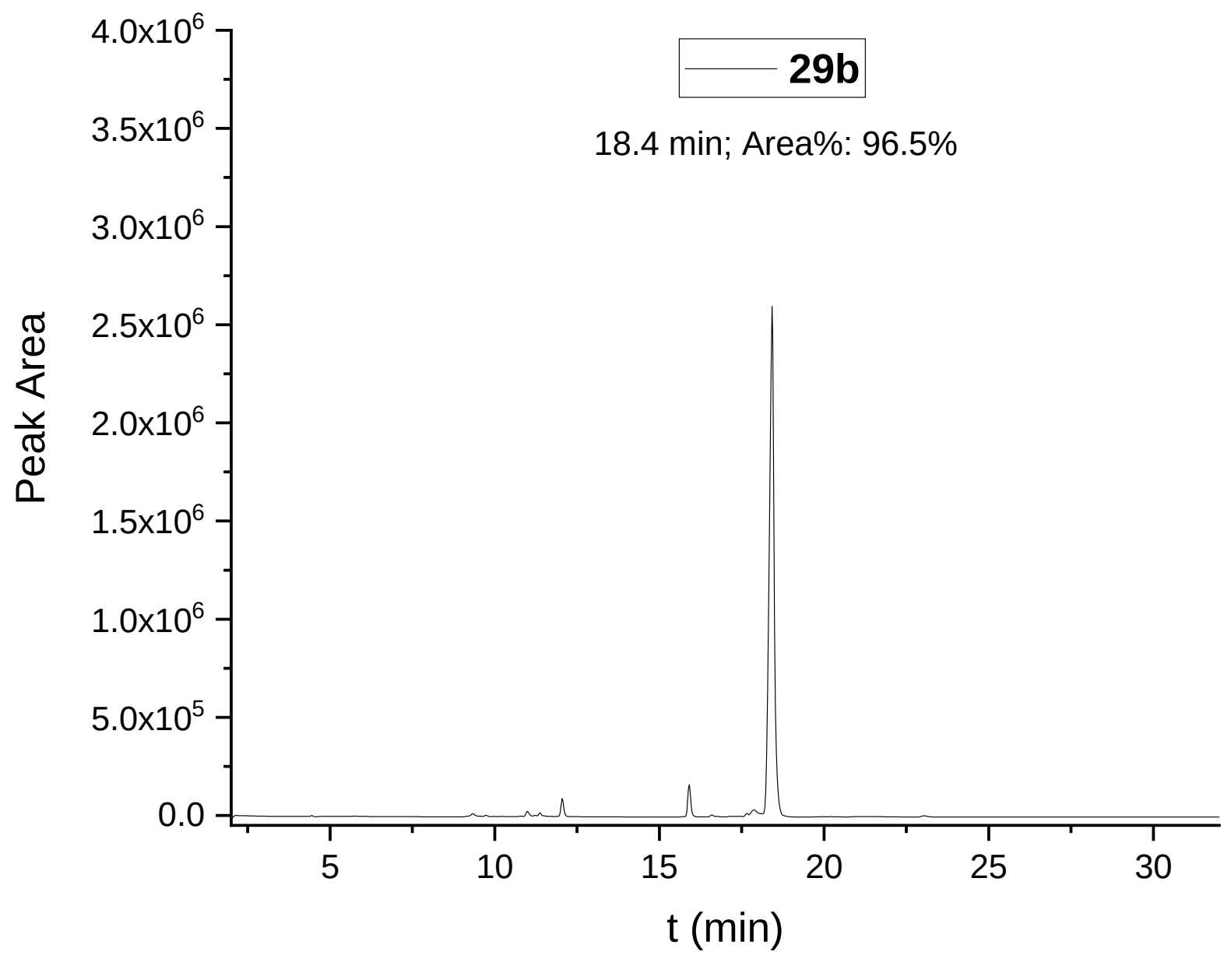
29b
18.4 min; Area%: 96.5%
Peak Area
t (min)
4.0x10^6
3.5x10^6
3.0x10^6
2.5x10^6
2.0x10^6
1.5x10^6
1.0x10^6
5.0x10^5
0.0
5
10
15
20
25
30

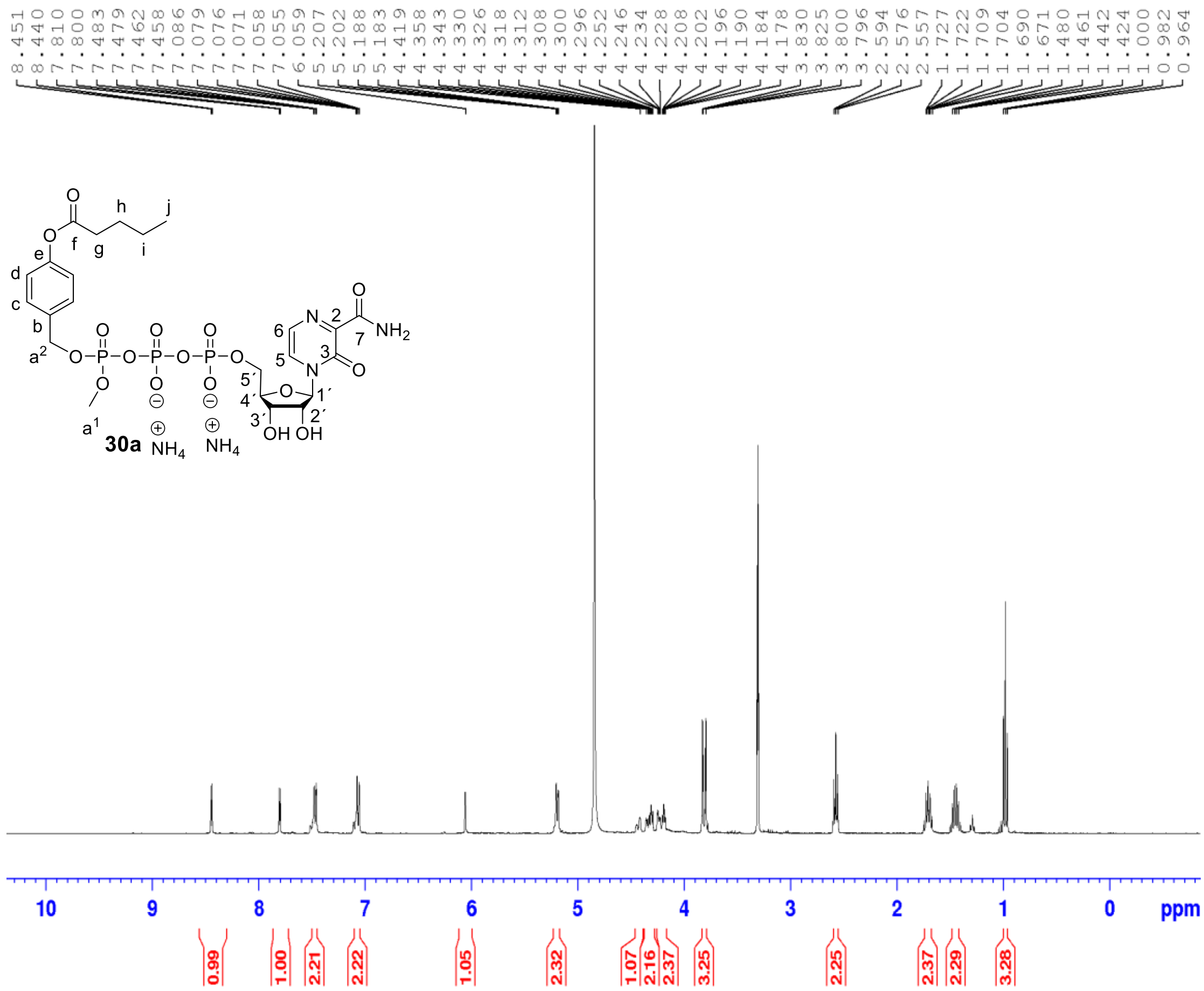
8.451 8.440 7.810 7.800 7.483 7.479 7.462 7.458 7.086 7.079 7.076 7.071 7.058 7.055 6.059 5.207 5.202 5.188 5.183 4.419 4.358 4.343 4.330 4.326 4.318 4.312 4.308 4.300 4.296 4.252 4.246 4.234 4.228 4.208 4.202 4.196 4.190 4.184 4.178 3.830 3.825 3.800 3.796 2.594 2.576 2.557 1.727 1.722 1.709 1.704 1.690 1.671 1.480 1.461 1.442 1.424 1.000 0.982 0.964
30a
ppm
0.99 1.00 2.21 2.22 1.05 2.32 1.07 2.16 2.37 3.25 2.25 2.37 2.29 3.28

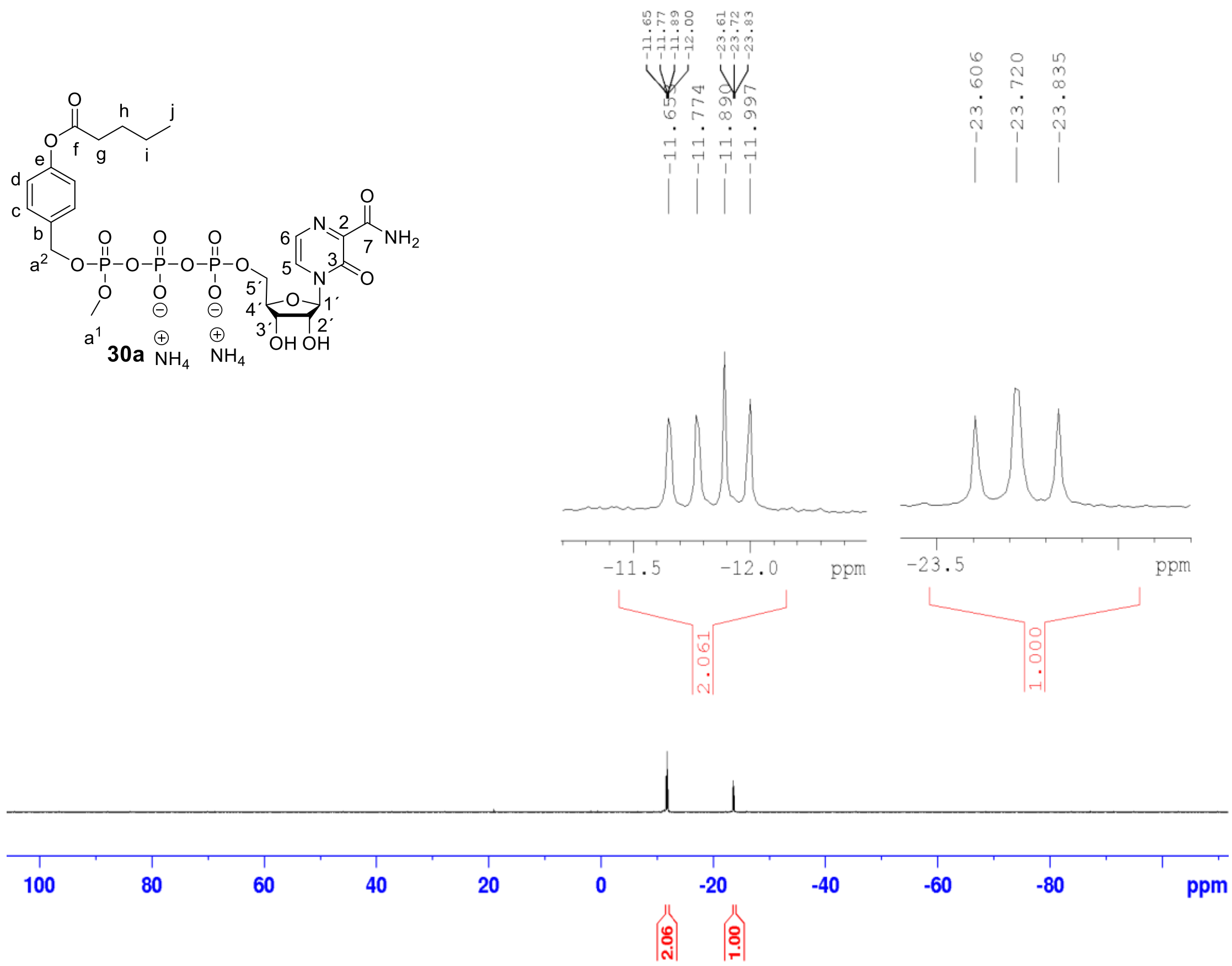
-11.65 -11.77 -11.89 -12.00
-23.61 -23.72 -23.83
-11.65 -11.774 -11.890 -11.997
-23.606 -23.720 -23.835
30a
-11.5 -12.0 ppm
-23.5 ppm
2.061 1.000
ppm
2.06 1.00

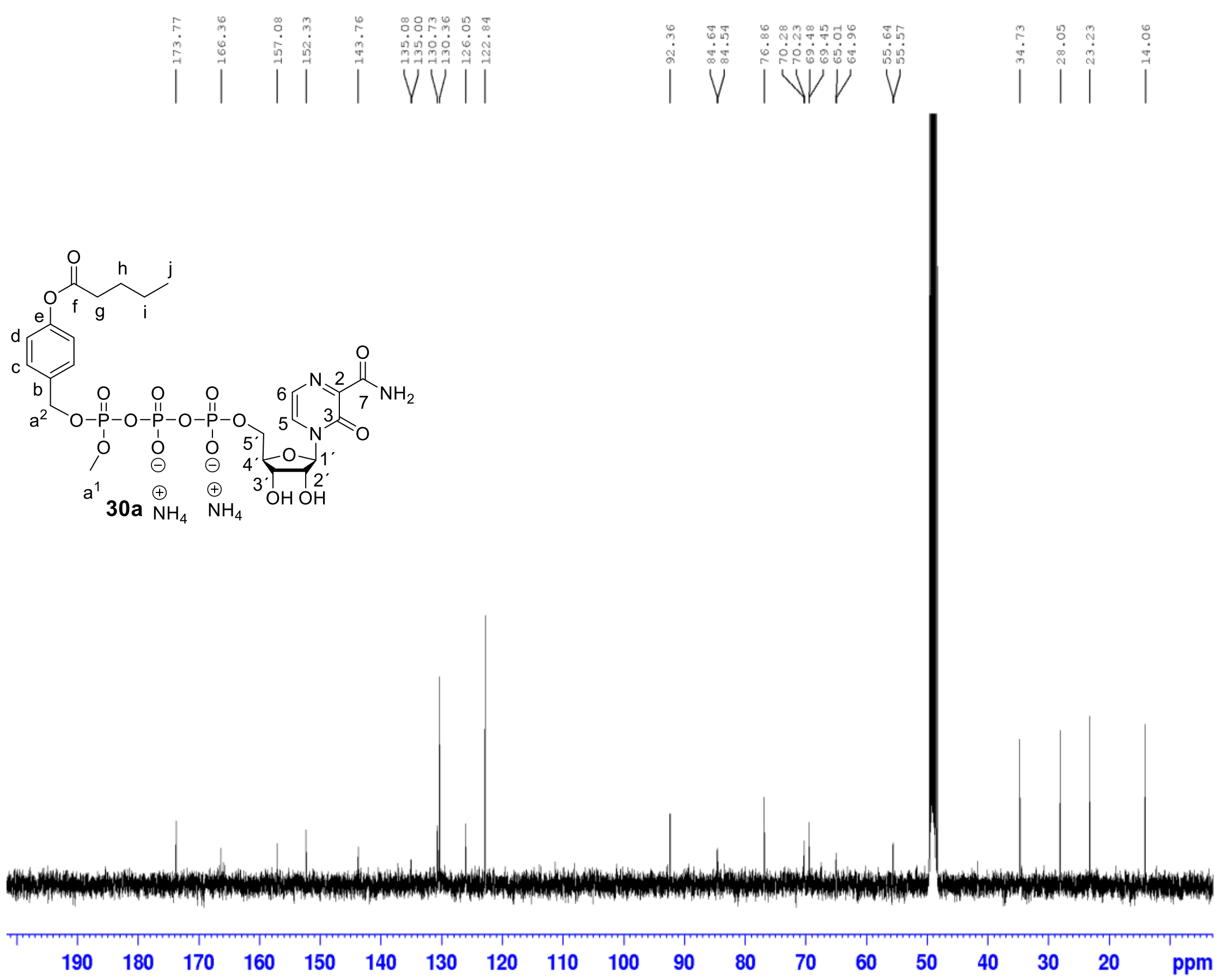

173.77
166.36
157.08
152.33
143.76
135.08
135.00
130.73
130.36
126.05
122.84
92.36
84.64
84.54
76.86
70.28
70.23
69.48
69.45
65.01
64.96
55.64
55.57
34.73
28.05
23.23
14.06
30a
ppm

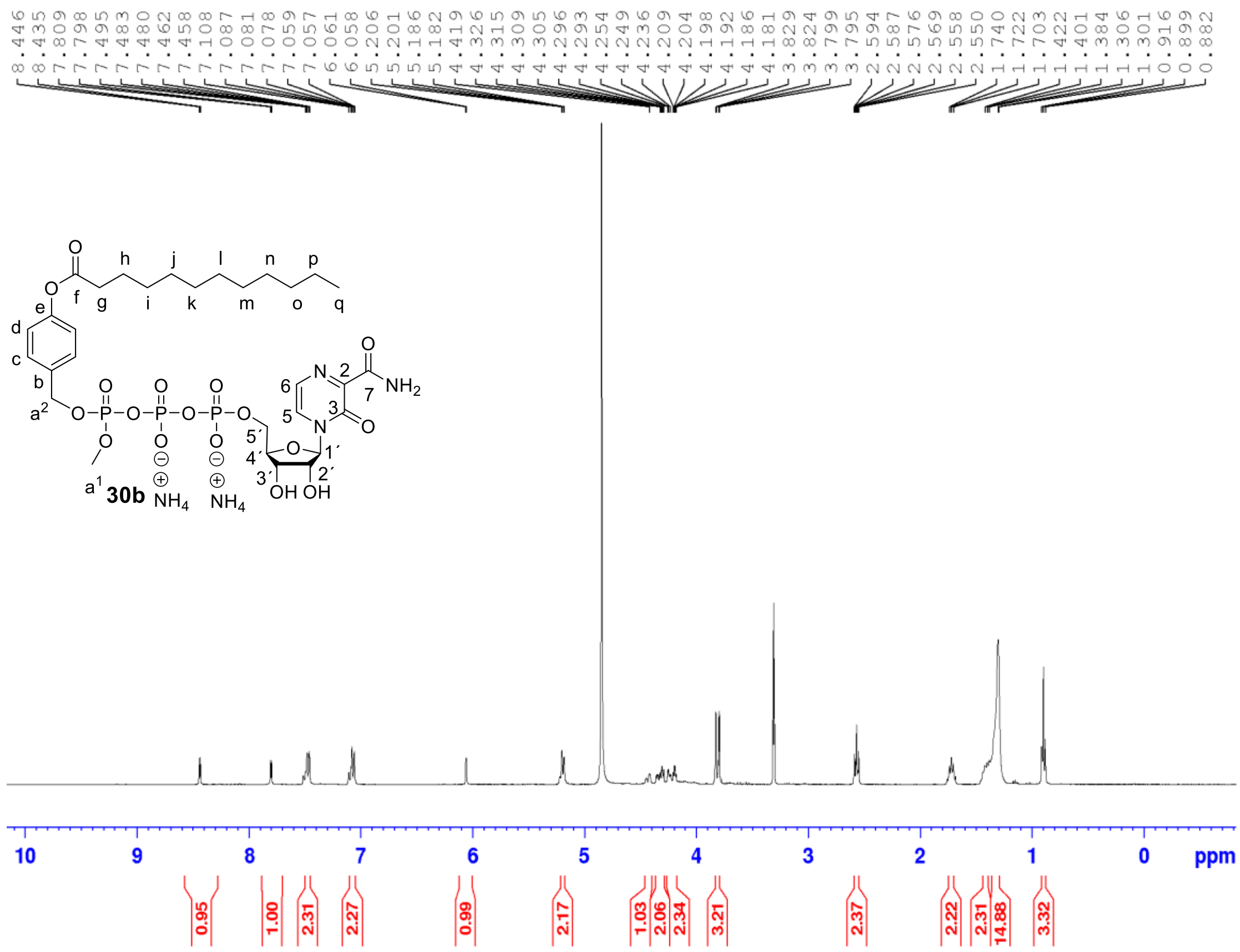

8.446
8.435
7.809
7.798
7.495
7.483
7.480
7.462
7.458
7.108
7.087
7.081
7.078
7.059
7.057
6.061
6.058
5.206
5.201
5.186
5.182
4.419
4.326
4.315
4.309
4.305
4.296
4.293
4.254
4.249
4.236
4.209
4.204
4.198
4.192
4.186
4.181
3.829
3.824
3.799
3.795
2.594
2.587
2.576
2.569
2.558
2.550
1.740
1.722
1.703
1.422
1.401
1.384
1.306
1.301
0.916
0.899
0.882
30b
ppm
0.95
1.00
2.31
2.27
0.99
2.17
1.03
2.06
2.34
3.21
2.37
2.22
2.31
14.88
3.32

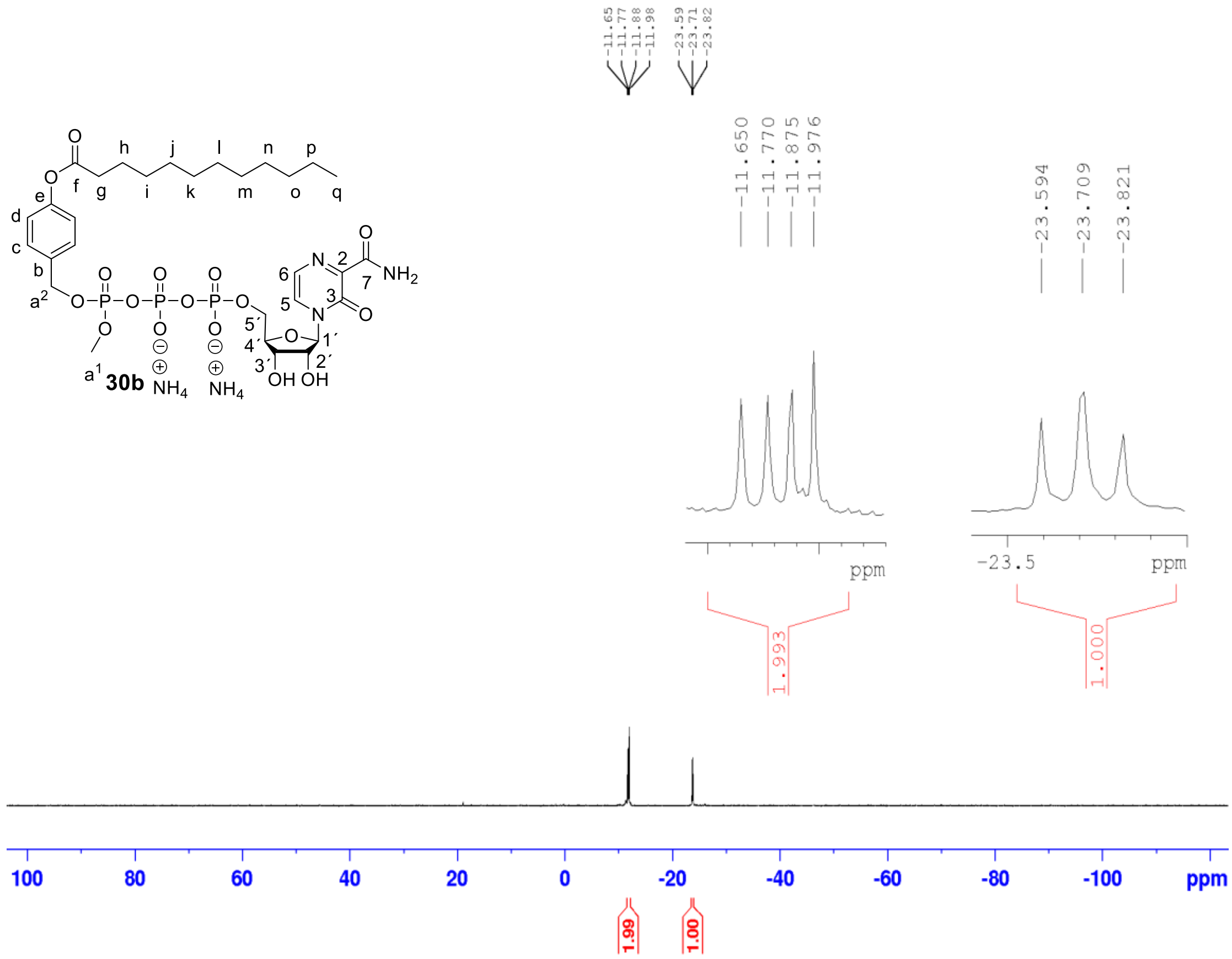

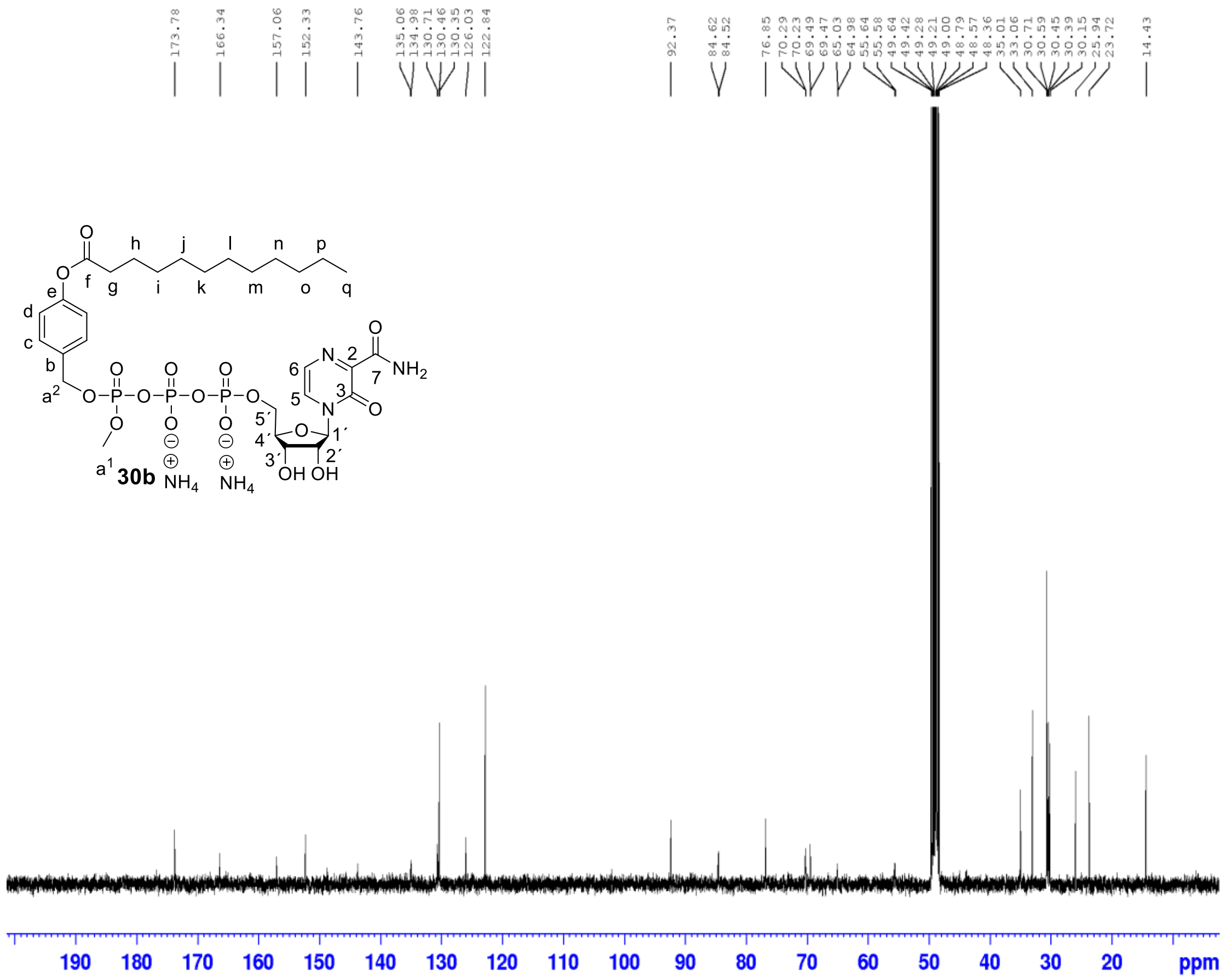

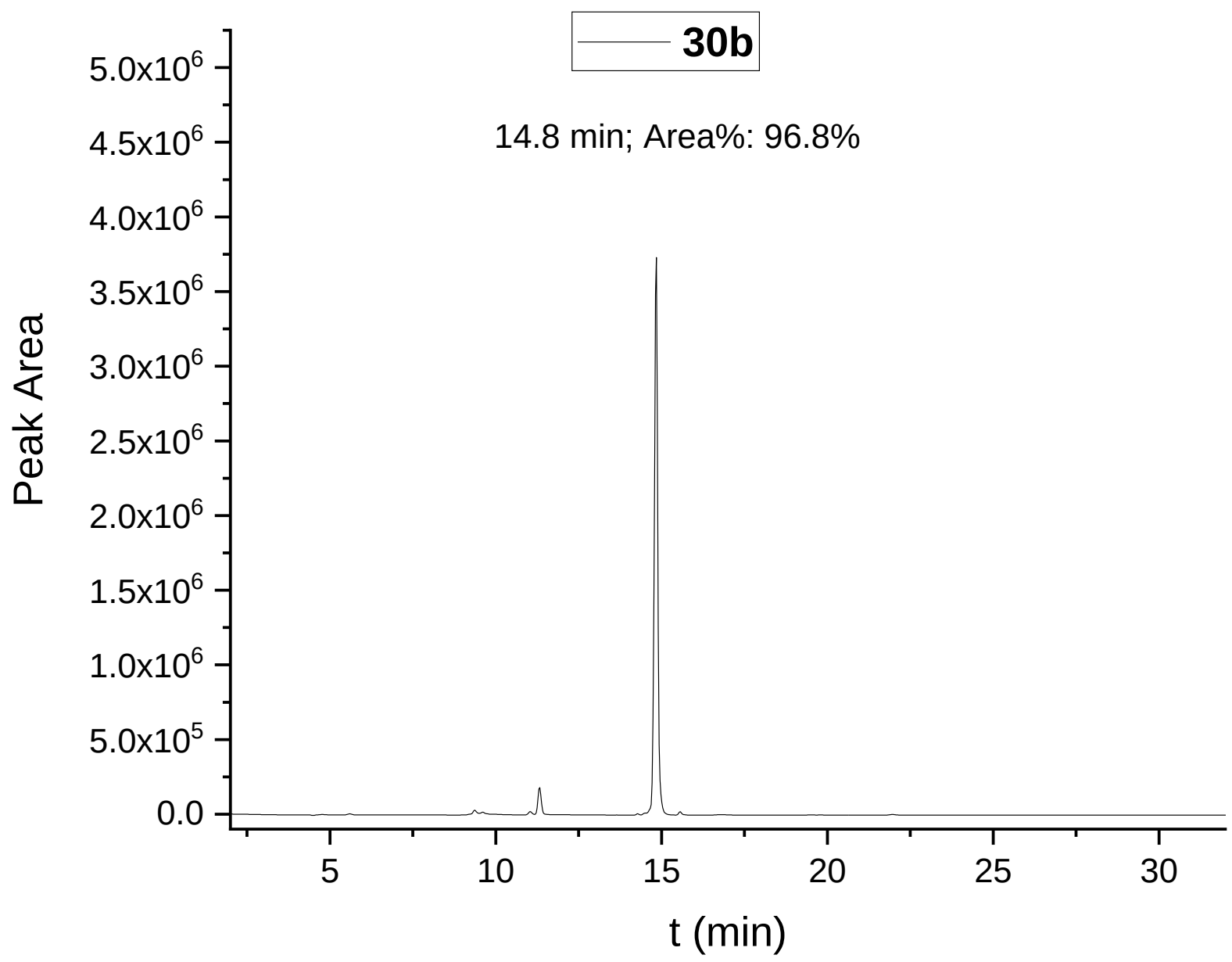

30b
14.8 min; Area%: 96.8%
Peak Area
t (min)

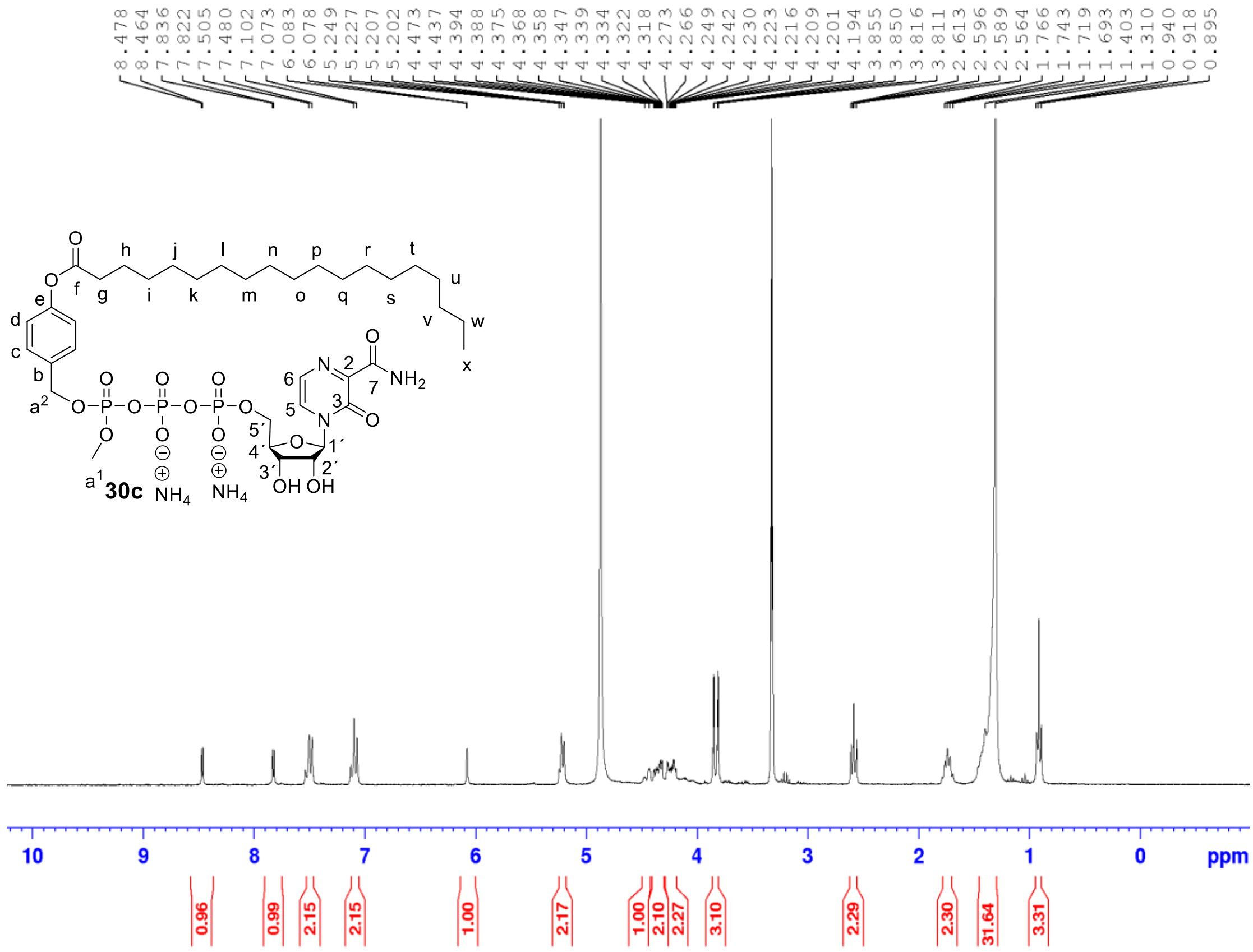

30c
ppm

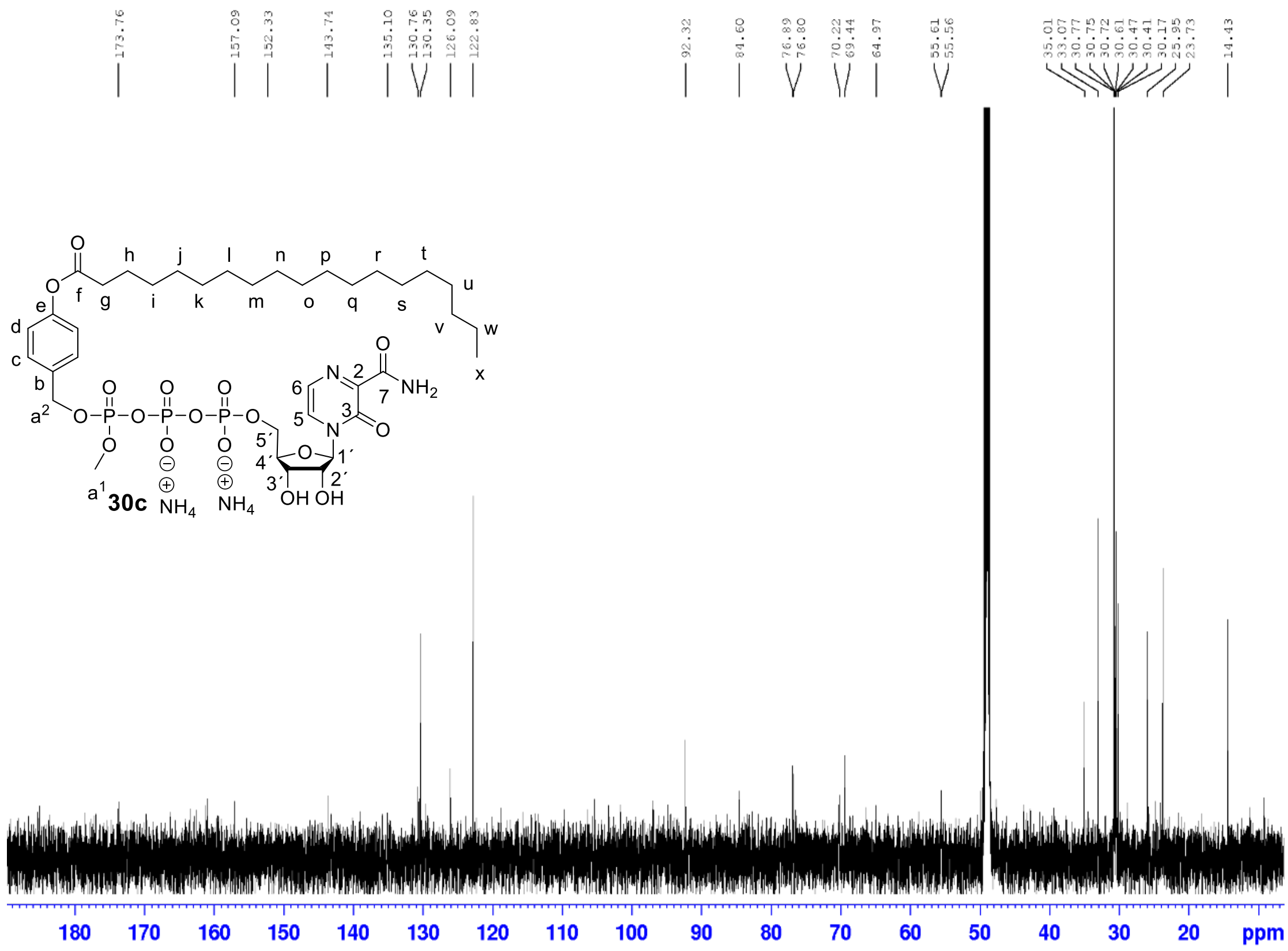
173.76
157.09
152.33
143.74
135.10
130.76
130.35
126.09
122.83
92.32
84.60
76.89
76.80
70.22
69.44
64.97
55.61
55.56
35.01
33.07
30.77
30.75
30.72
30.61
30.47
30.41
30.17
25.95
23.73
14.43
30c
ppm

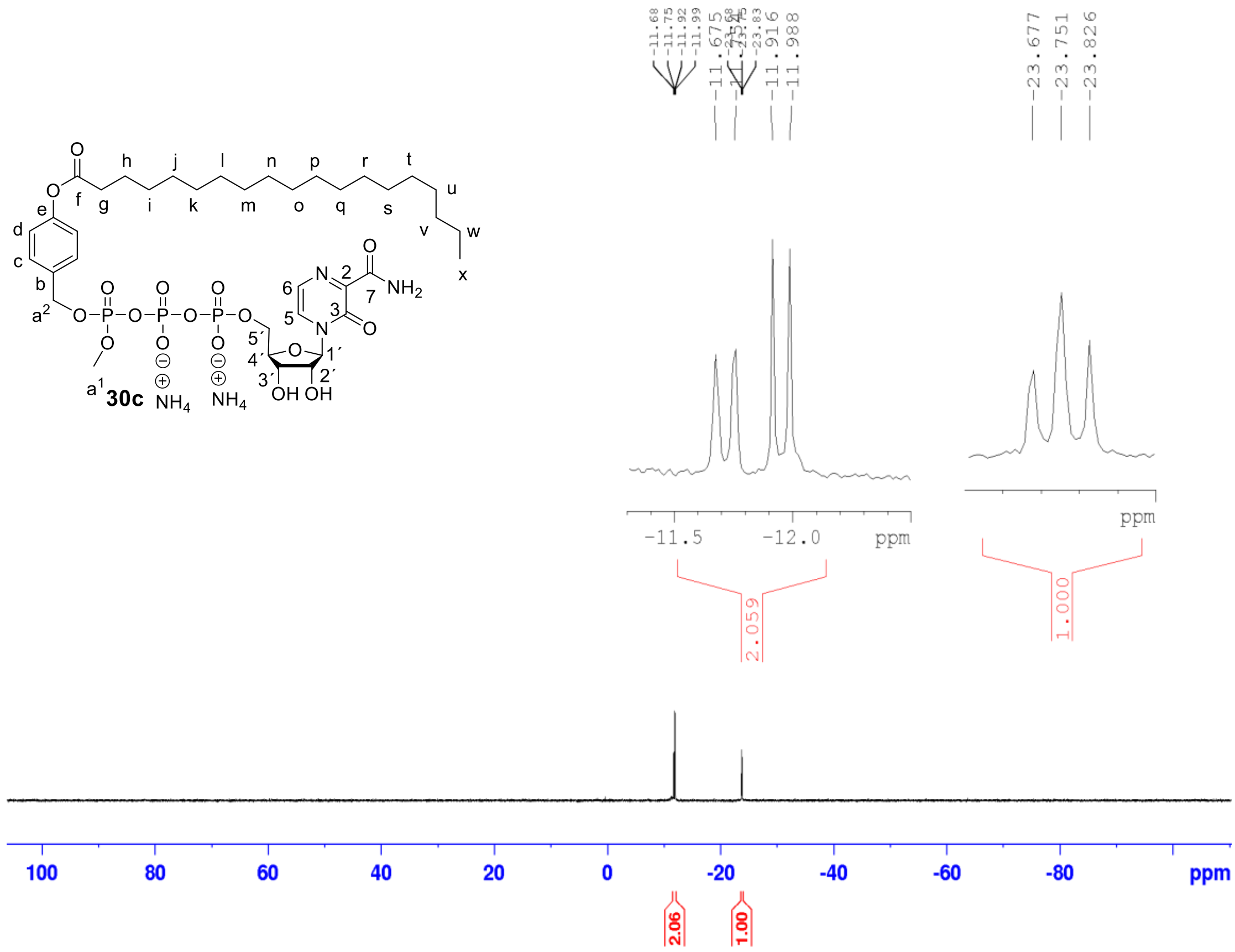
-11.68
-11.75
-11.92
-11.99
-11.675
-11.751
-11.916
-11.988
-23.68
-23.75
-23.83
-23.677
-23.751
-23.826
30c
2.059
1.000
2.06
1.00
ppm

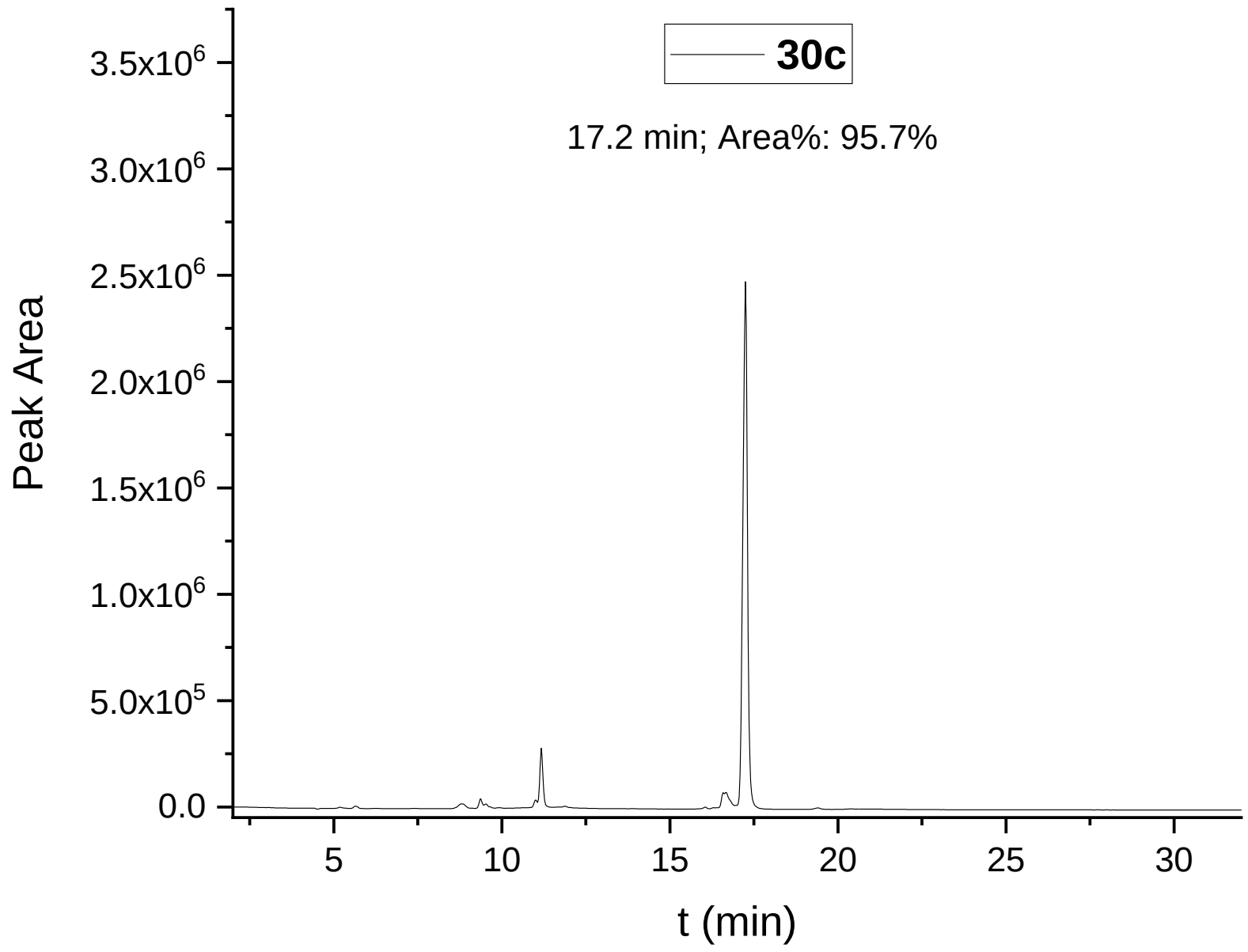
30c
17.2 min; Area%: 95.7%
Peak Area
t (min)

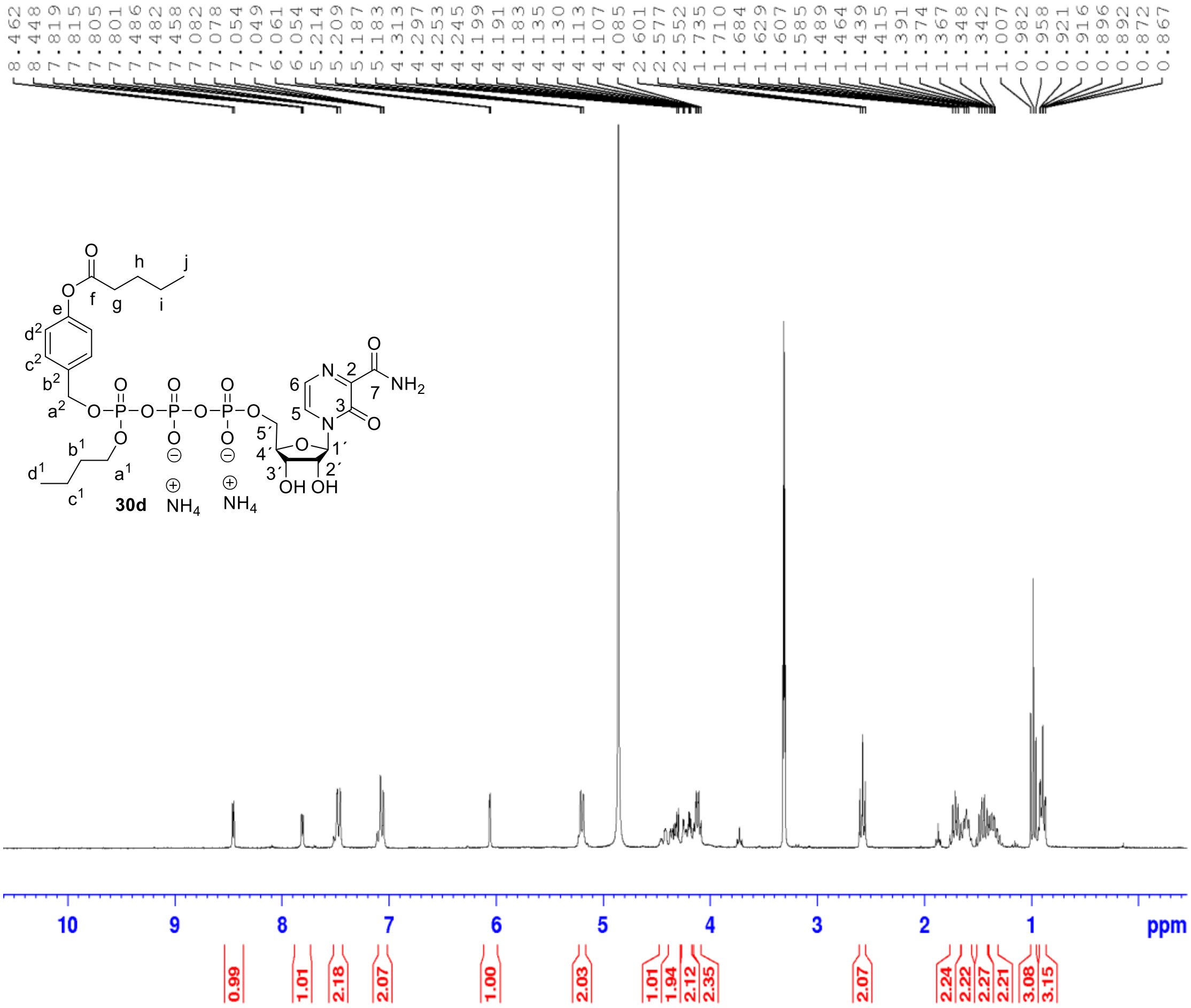
30d
ppm

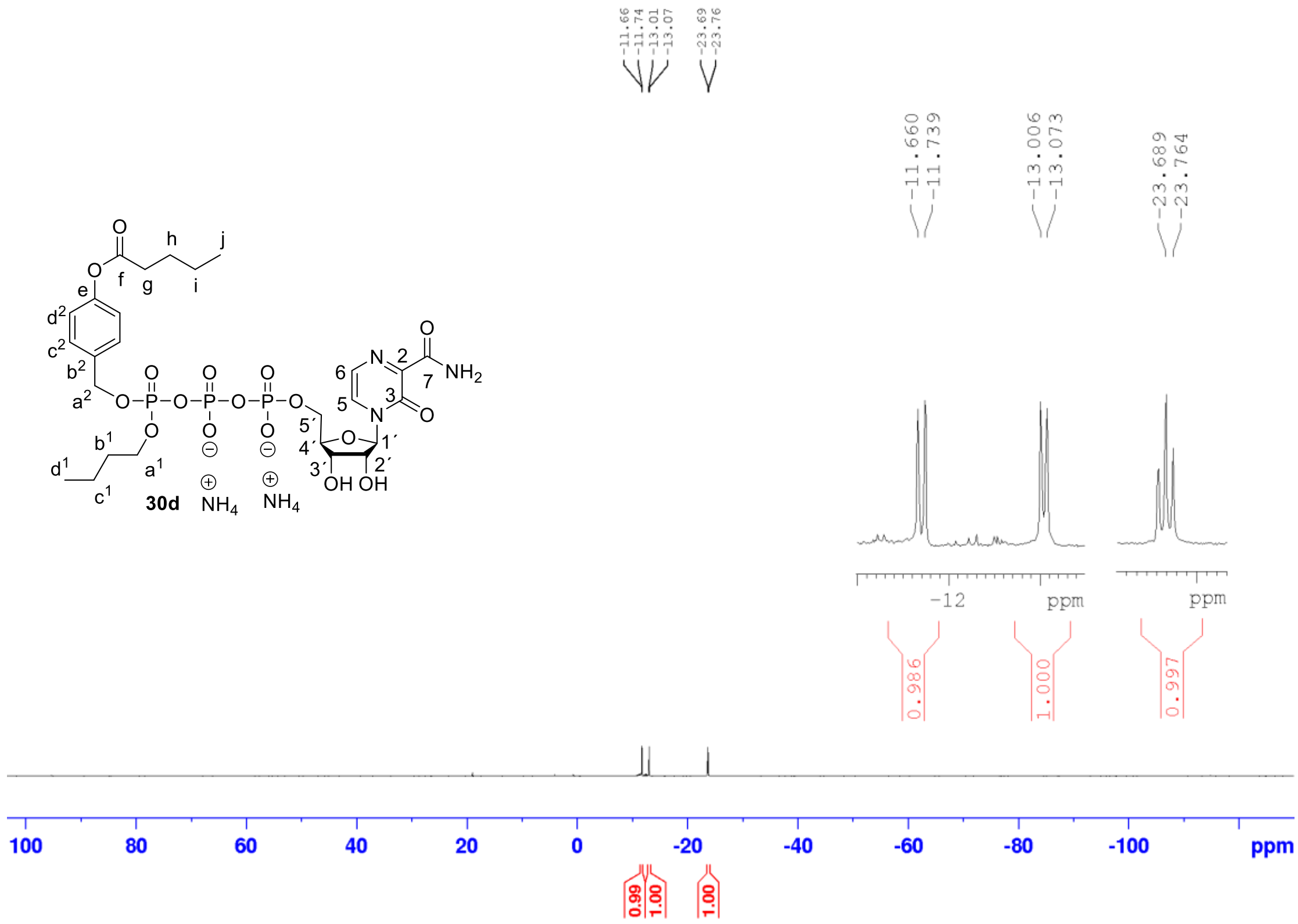
-11.66
-11.74
-13.01
-13.07
-23.69
-23.76
-11.660
-11.739
-13.006
-13.073
-23.689
-23.764
30d
-12
ppm
ppm
0.986
1.000
0.997
100 80 60 40 20 0 -20 -40 -60 -80 -100 ppm
0.99
1.00
1.00

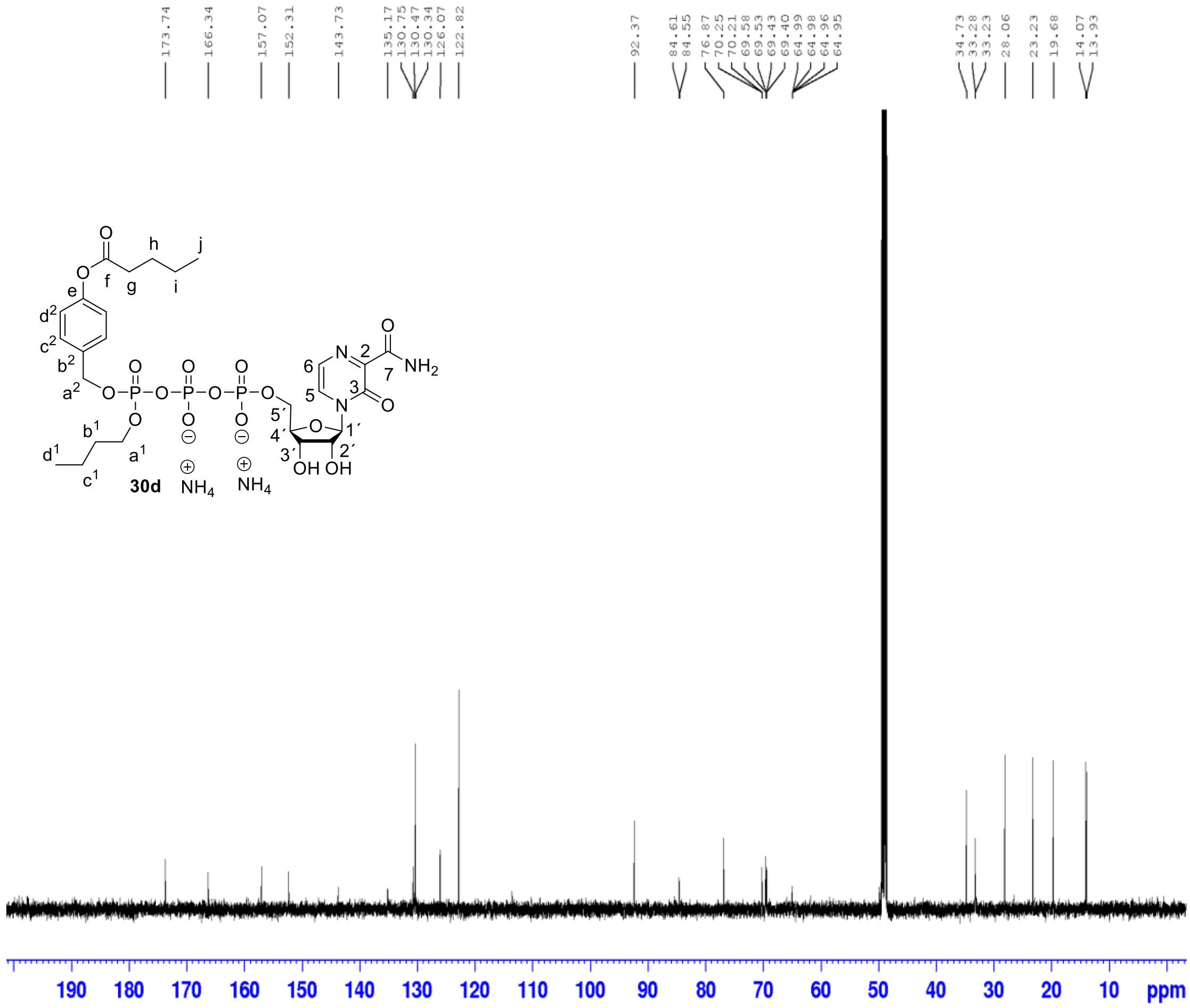
173.74
166.34
157.07
152.31
143.73
135.17
130.75
130.47
130.34
126.07
122.82
92.37
84.61
84.55
76.87
70.25
70.21
69.58
69.53
69.43
69.40
64.99
64.98
64.96
64.95
34.73
33.28
33.23
28.06
23.23
19.68
14.07
13.93
30d
190 180 170 160 150 140 130 120 110 100 90 80 70 60 50 40 30 20 10 ppm

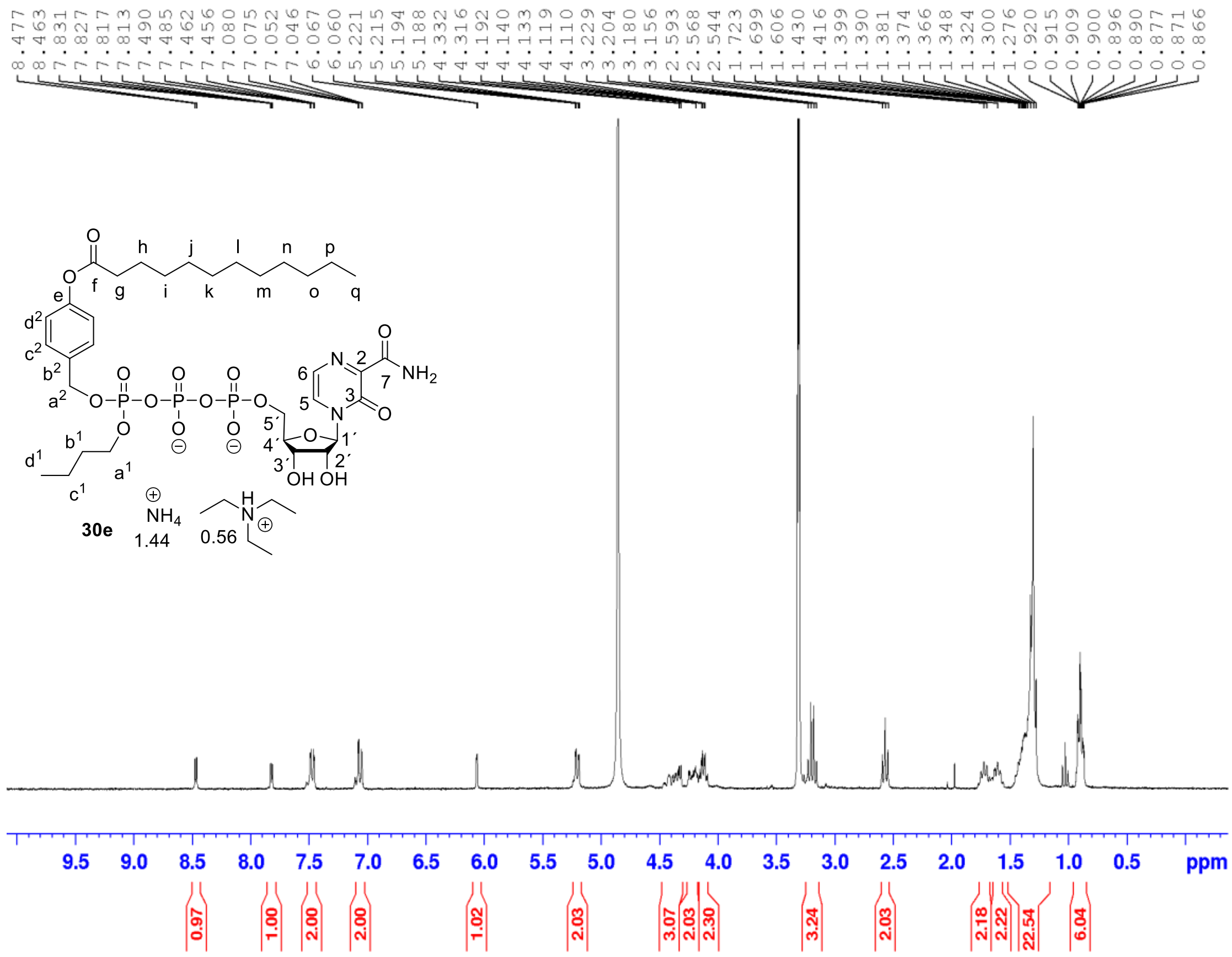
8.477 8.463 7.831 7.827 7.817 7.813 7.490 7.485 7.462 7.456 7.080 7.075 7.052 7.046 6.067 6.060 5.221 5.215 5.194 5.188 4.332 4.316 4.192 4.140 4.133 4.119 4.110 3.229 3.204 3.180 3.156 2.593 2.568 2.544 1.723 1.699 1.606 1.430 1.416 1.399 1.390 1.381 1.374 1.366 1.348 1.324 1.300 1.276 0.920 0.915 0.909 0.900 0.896 0.890 0.877 0.871 0.866
30e
NH4
1.44
0.56
9.5 9.0 8.5 8.0 7.5 7.0 6.5 6.0 5.5 5.0 4.5 4.0 3.5 3.0 2.5 2.0 1.5 1.0 0.5 ppm
0.97 1.00 2.00 2.00 1.02 2.03 3.07 2.03 2.30 3.24 2.03 2.18 2.22 22.54 6.04

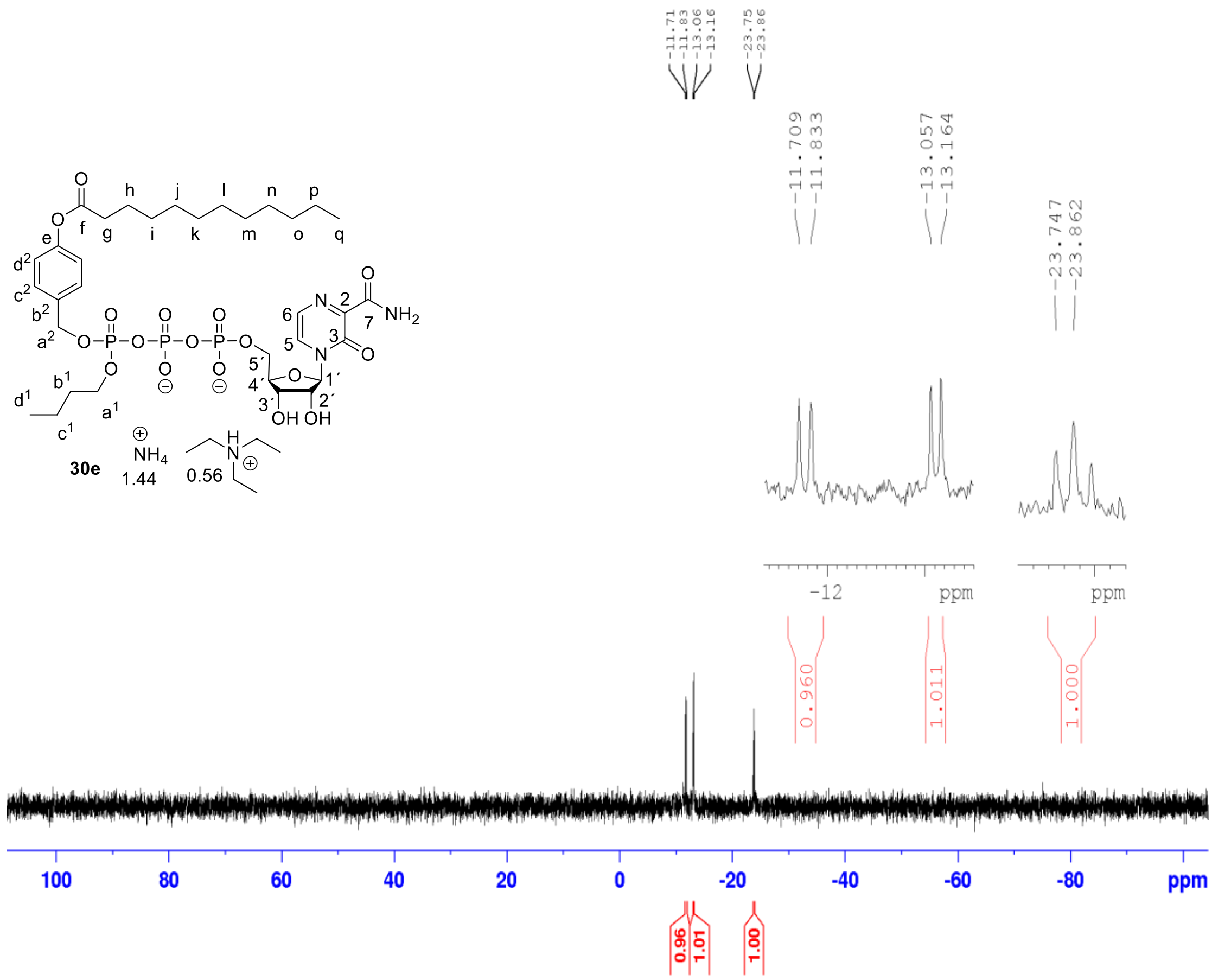
-11.71 -11.83 -13.06 -13.16 -23.75 -23.86
-11.709 -11.833
-13.057 -13.164
-23.747 -23.862
30e
NH4
1.44
0.56
-12 ppm
ppm
0.960 1.011 1.000
100 80 60 40 20 0 -20 -40 -60 -80 ppm
0.96 1.01 1.00

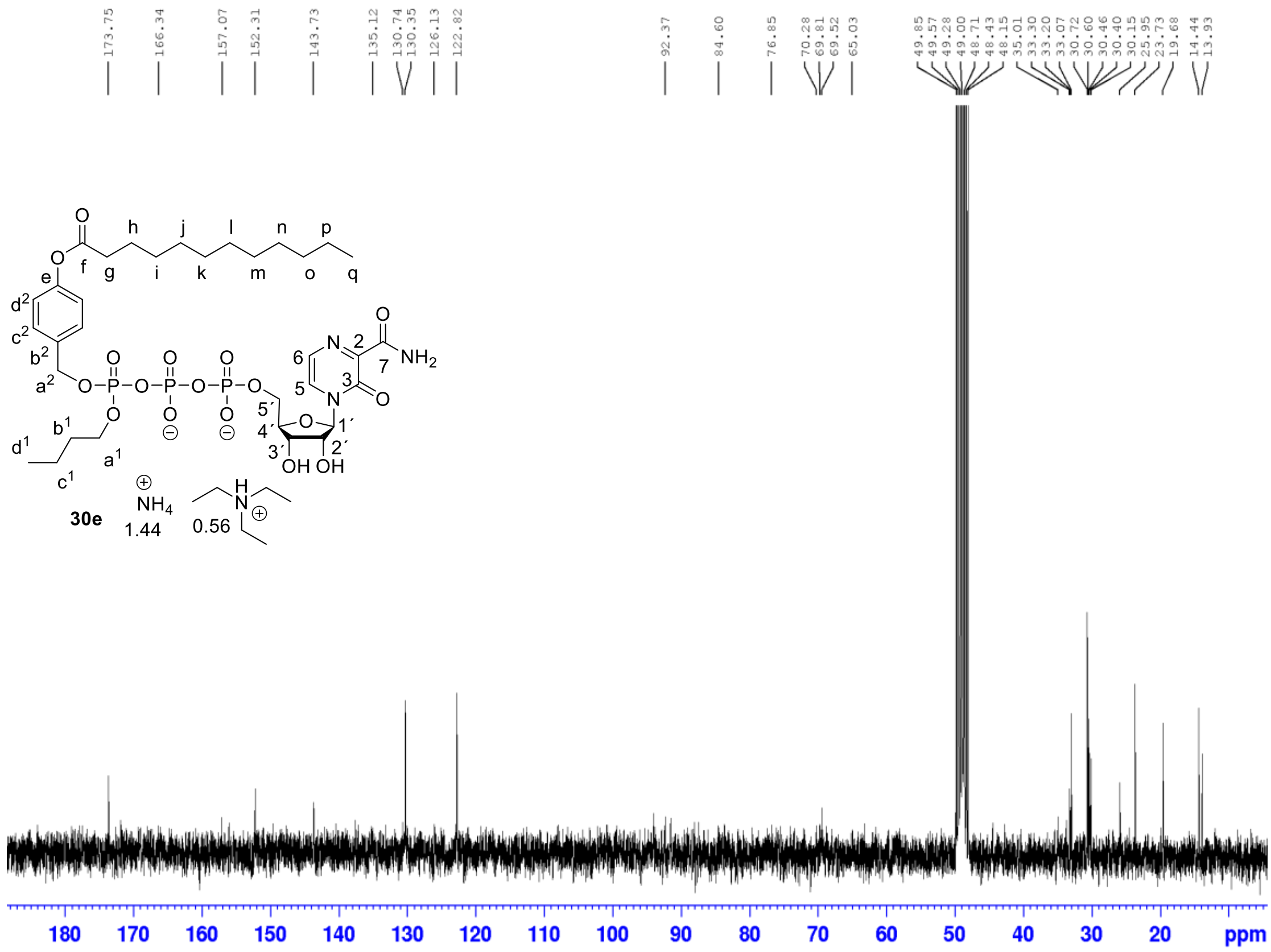
173.75
166.34
157.07
152.31
143.73
135.12
130.74
130.35
126.13
122.82
92.37
84.60
76.85
70.28
69.81
69.52
65.03
49.85
49.57
49.28
49.00
48.71
48.43
48.15
35.01
33.30
33.20
33.07
30.72
30.60
30.46
30.40
30.15
25.95
23.73
19.68
14.44
13.93
30e
1.44
0.56
180
170
160
150
140
130
120
110
100
90
80
70
60
50
40
30
20
ppm

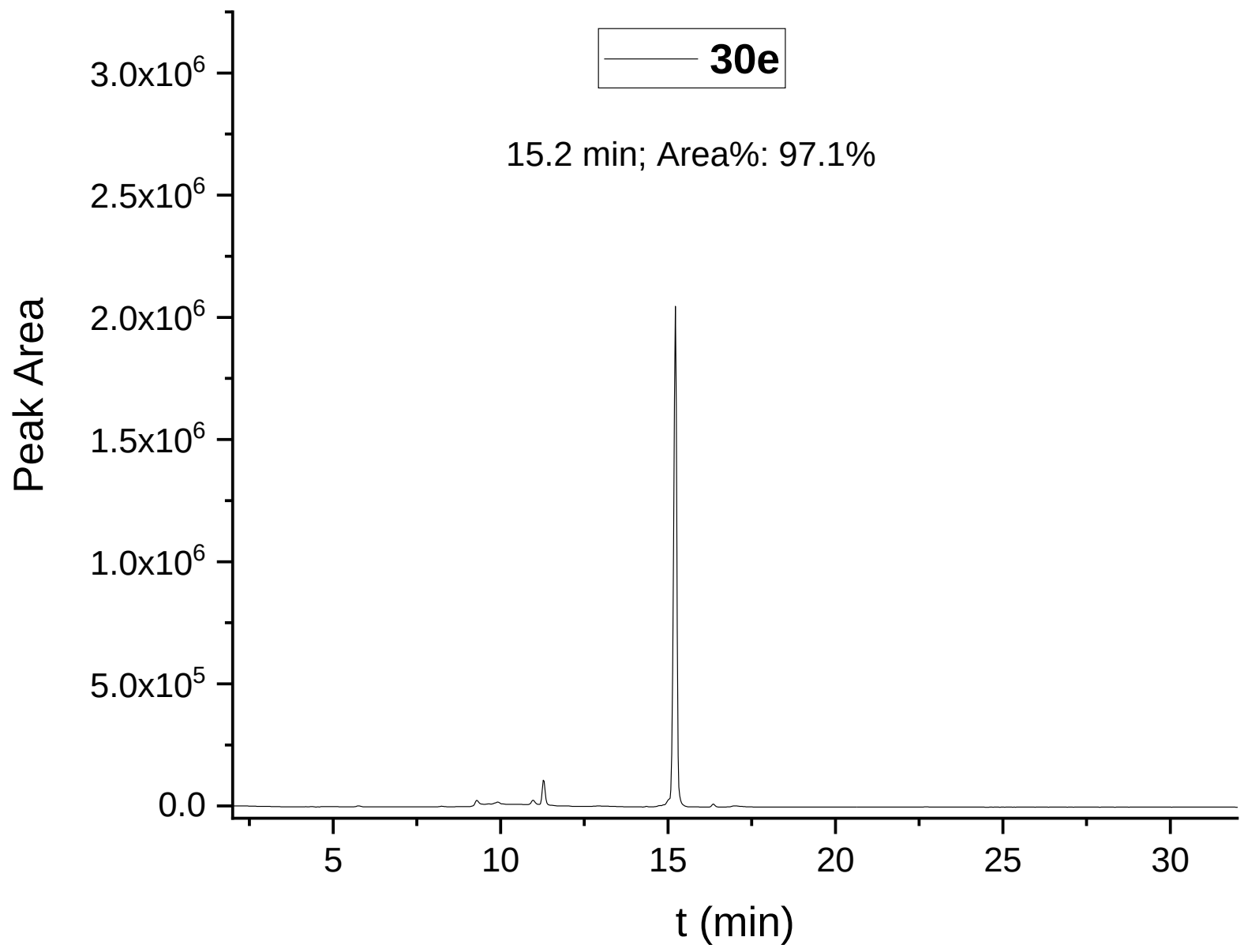
30e
15.2 min; Area%: 97.1%
Peak Area
t (min)

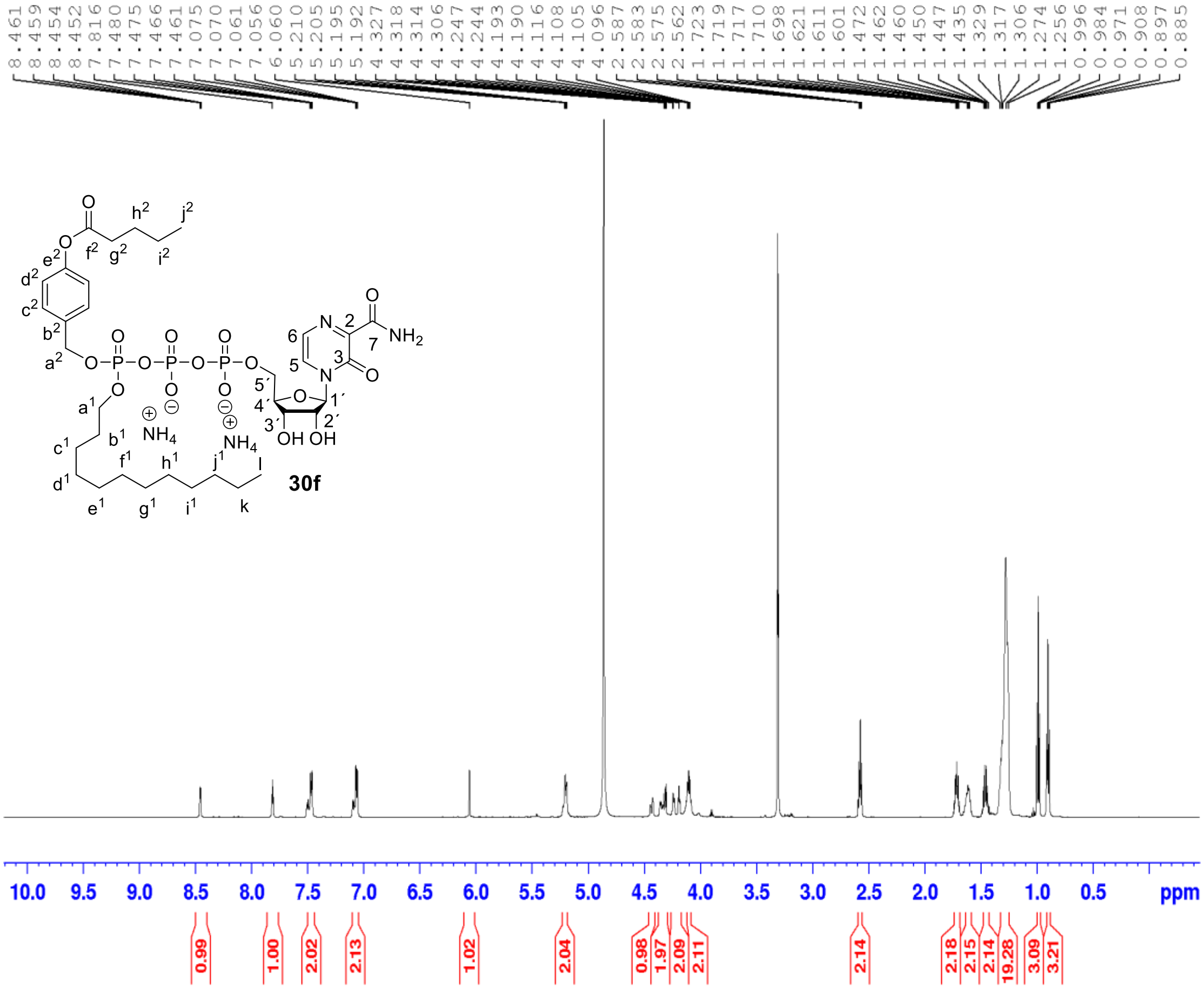
8.461 8.459 8.454 8.452 7.816 7.480 7.475 7.466 7.461 7.075 7.070 7.061 7.056 6.060 5.210 5.205 5.195 5.192 4.327 4.318 4.314 4.306 4.247 4.244 4.193 4.190 4.116 4.108 4.105 4.096 2.587 2.583 2.575 2.562 1.723 1.719 1.717 1.710 1.698 1.621 1.611 1.601 1.472 1.462 1.460 1.450 1.447 1.435 1.329 1.317 1.306 1.274 1.256 0.996 0.984 0.971 0.908 0.897 0.885
30f
ppm
0.99 1.00 2.02 2.13 1.02 2.04 0.98 1.97 2.09 2.11 2.14 2.18 2.15 2.14 19.28 3.09 3.21

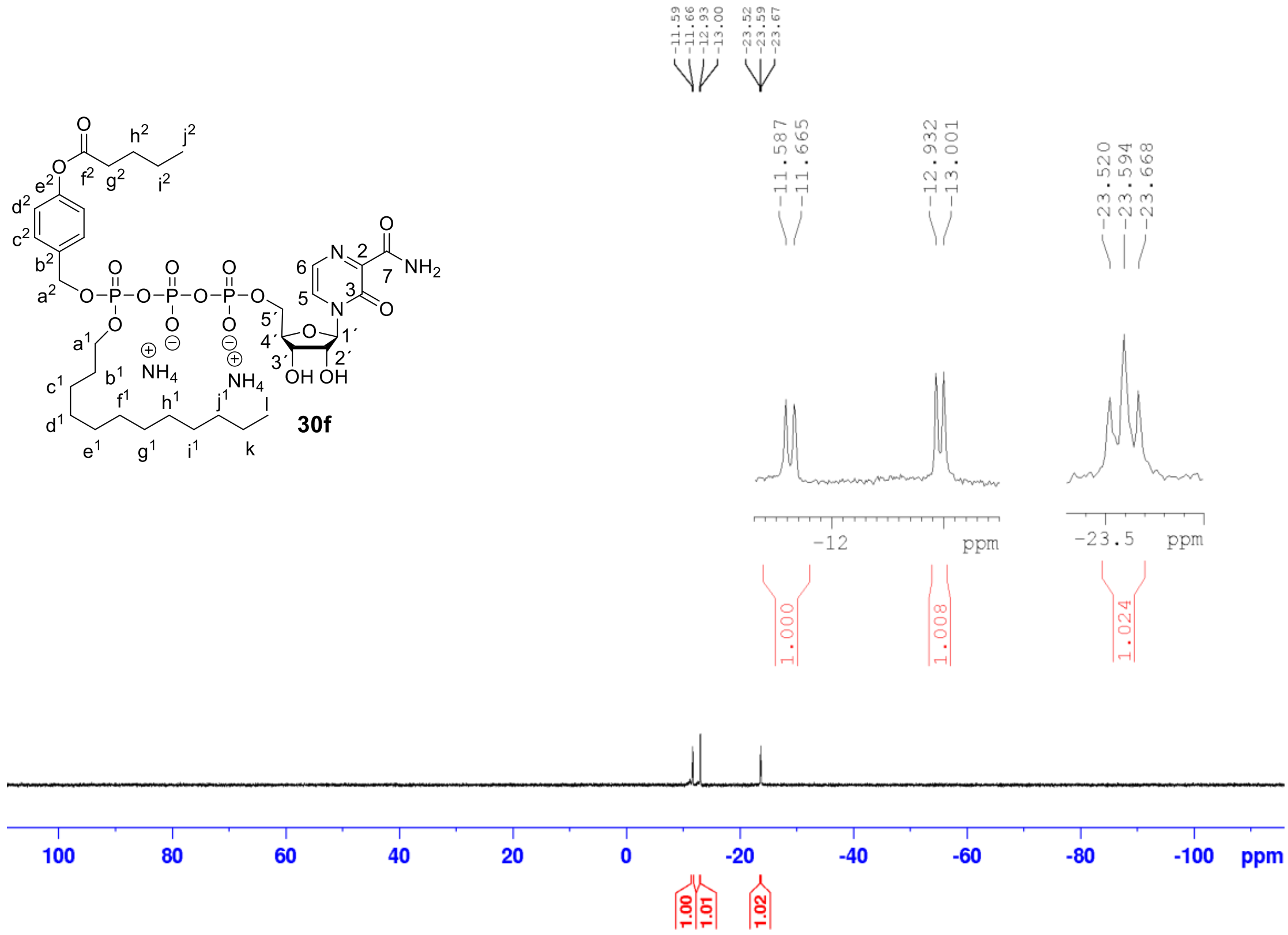
-11.59 -11.66 -12.93 -13.00 -23.52 -23.59 -23.67
-11.587 -11.665 -12.932 -13.001 -23.520 -23.594 -23.668
30f
ppm
1.000 1.008 1.024
1.00 1.01 1.02

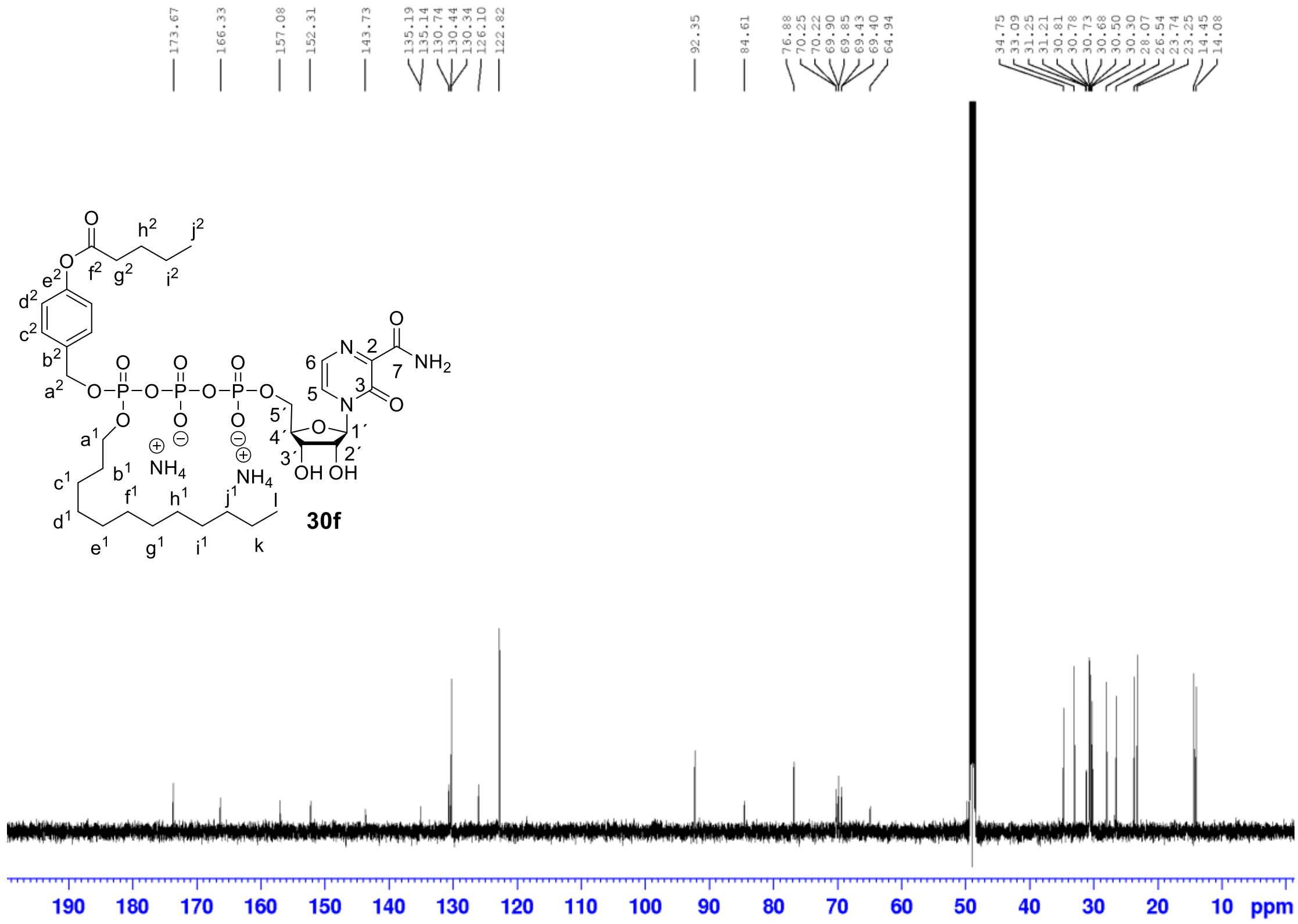
30f
190 180 170 160 150 140 130 120 110 100 90 80 70 60 50 40 30 20 10 ppm

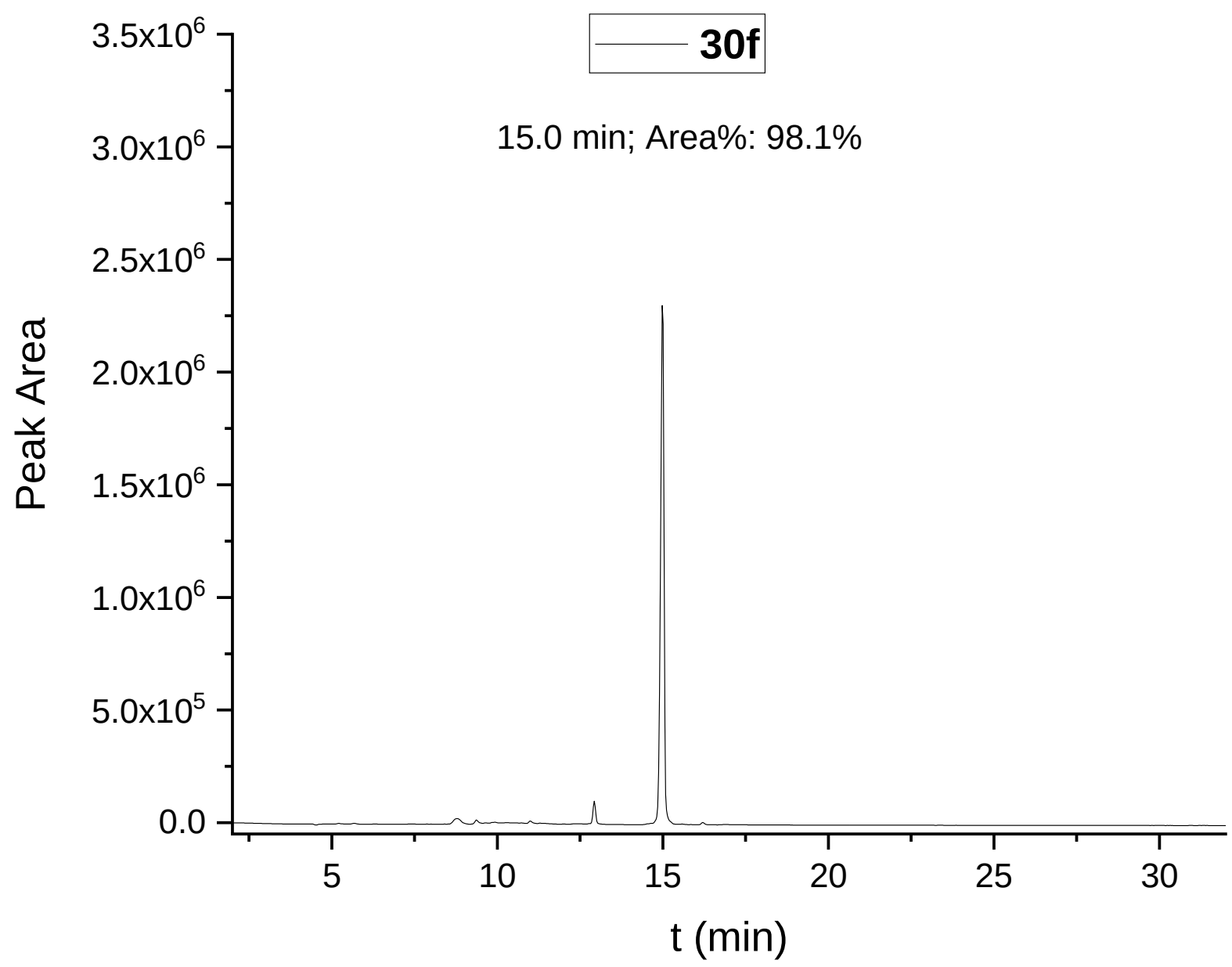
30f
15.0 min; Area%: 98.1%
Peak Area
t (min)

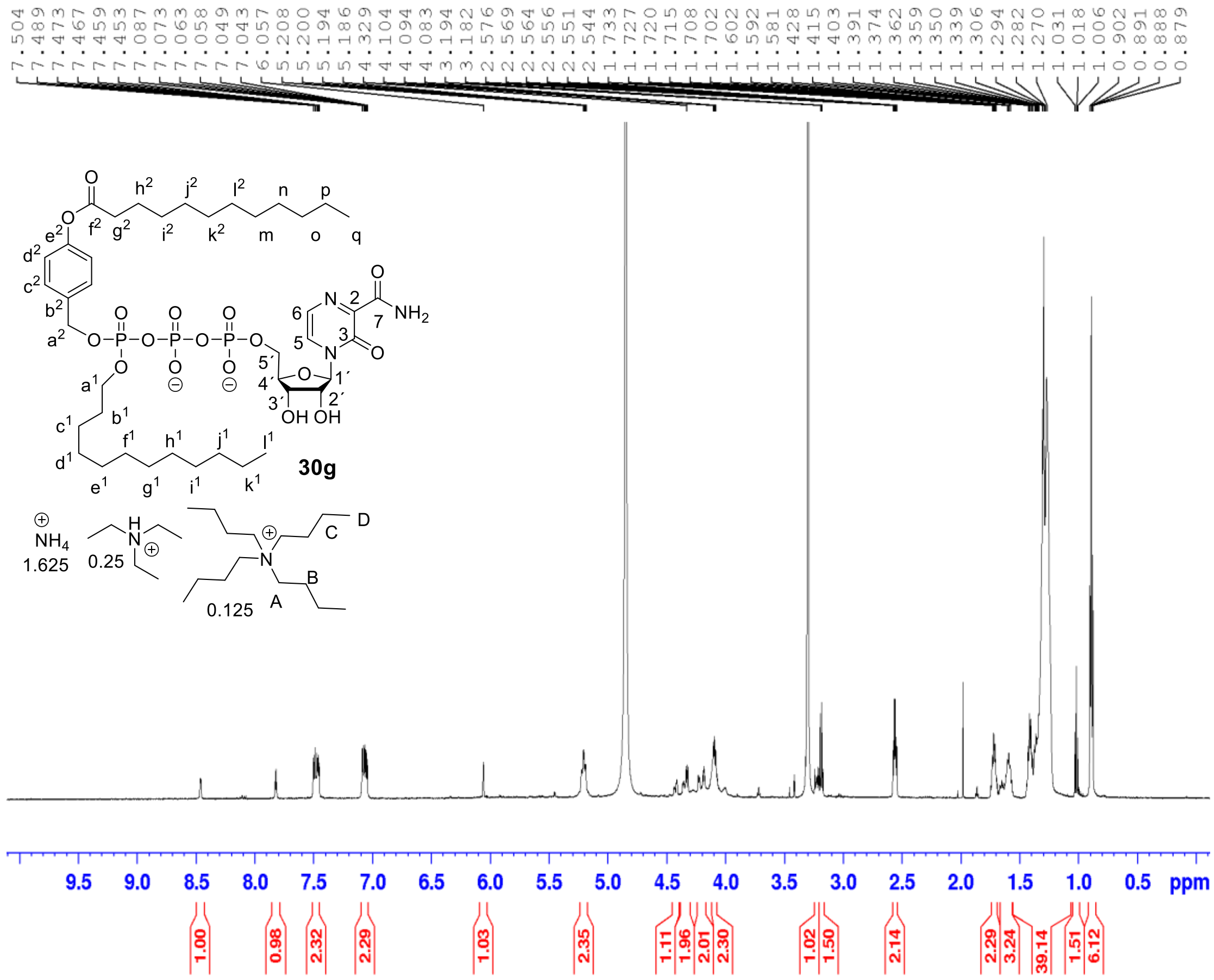
7.504 7.489 7.473 7.467 7.459 7.453 7.087 7.073 7.063 7.058 7.049 7.043 6.057 5.208 5.200 5.194 5.186 4.329 4.104 4.094 4.083 3.194 3.182 2.576 2.569 2.564 2.556 2.551 2.544 1.733 1.727 1.720 1.715 1.708 1.702 1.602 1.592 1.581 1.428 1.415 1.403 1.391 1.374 1.362 1.359 1.350 1.339 1.306 1.294 1.282 1.270 1.031 1.018 1.006 0.902 0.891 0.888 0.879
30g
NH4
1.625
0.25
0.125
A
B
C
D
9.5 9.0 8.5 8.0 7.5 7.0 6.5 6.0 5.5 5.0 4.5 4.0 3.5 3.0 2.5 2.0 1.5 1.0 0.5 ppm
1.00 0.98 2.32 2.29 1.03 2.35 1.11 1.96 2.01 2.30 1.02 1.50 2.14 2.29 3.24 39.14 1.51 6.12

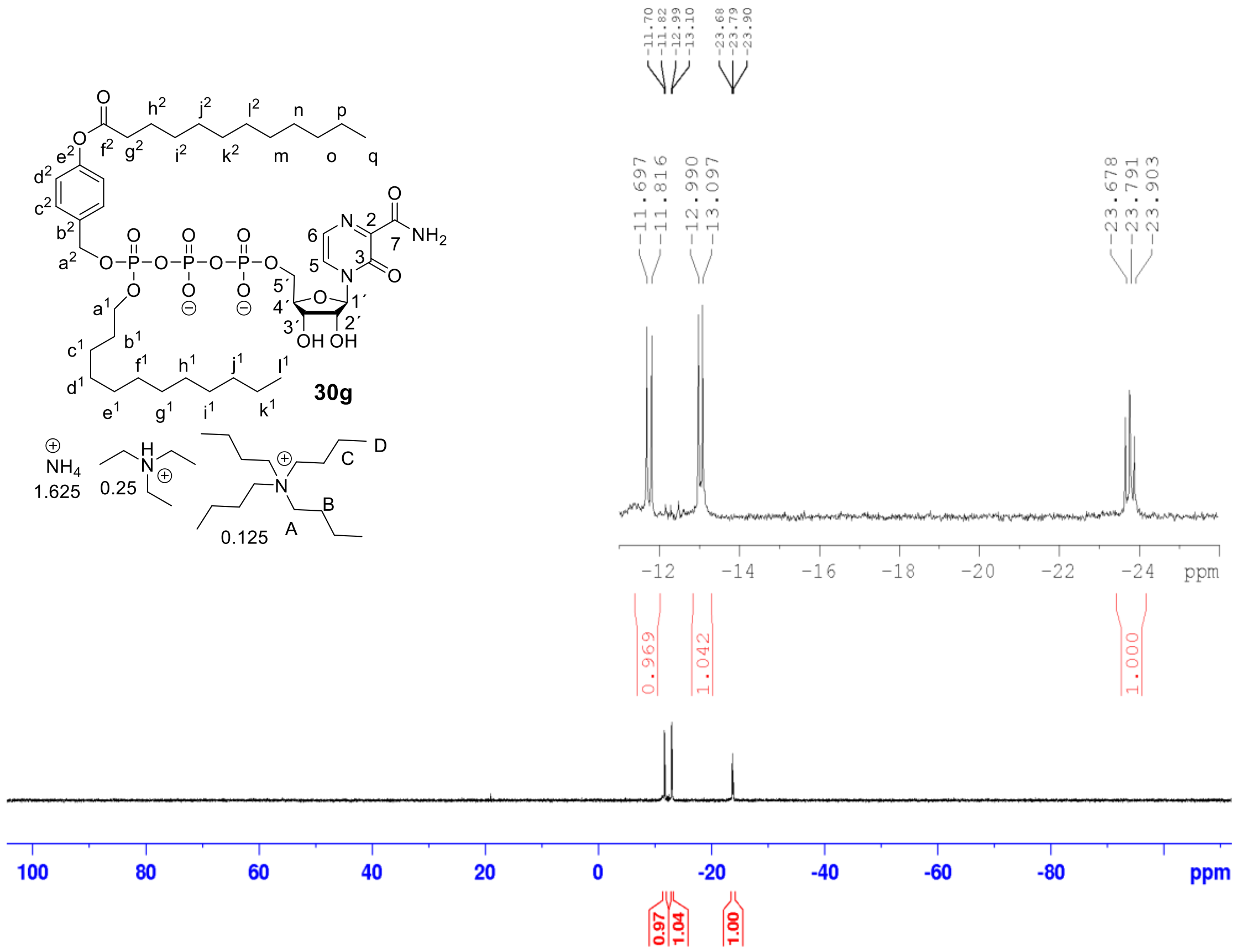
-11.70 -11.82 -12.99 -13.10 -23.68 -23.79 -23.90
-11.697 -11.816 -12.990 -13.097 -23.678 -23.791 -23.903
30g
NH4
1.625
0.25
0.125
A
B
C
D
-12 -14 -16 -18 -20 -22 -24 ppm
0.969 1.042 1.000
100 80 60 40 20 0 -20 -40 -60 -80 ppm
0.97 1.04 1.00

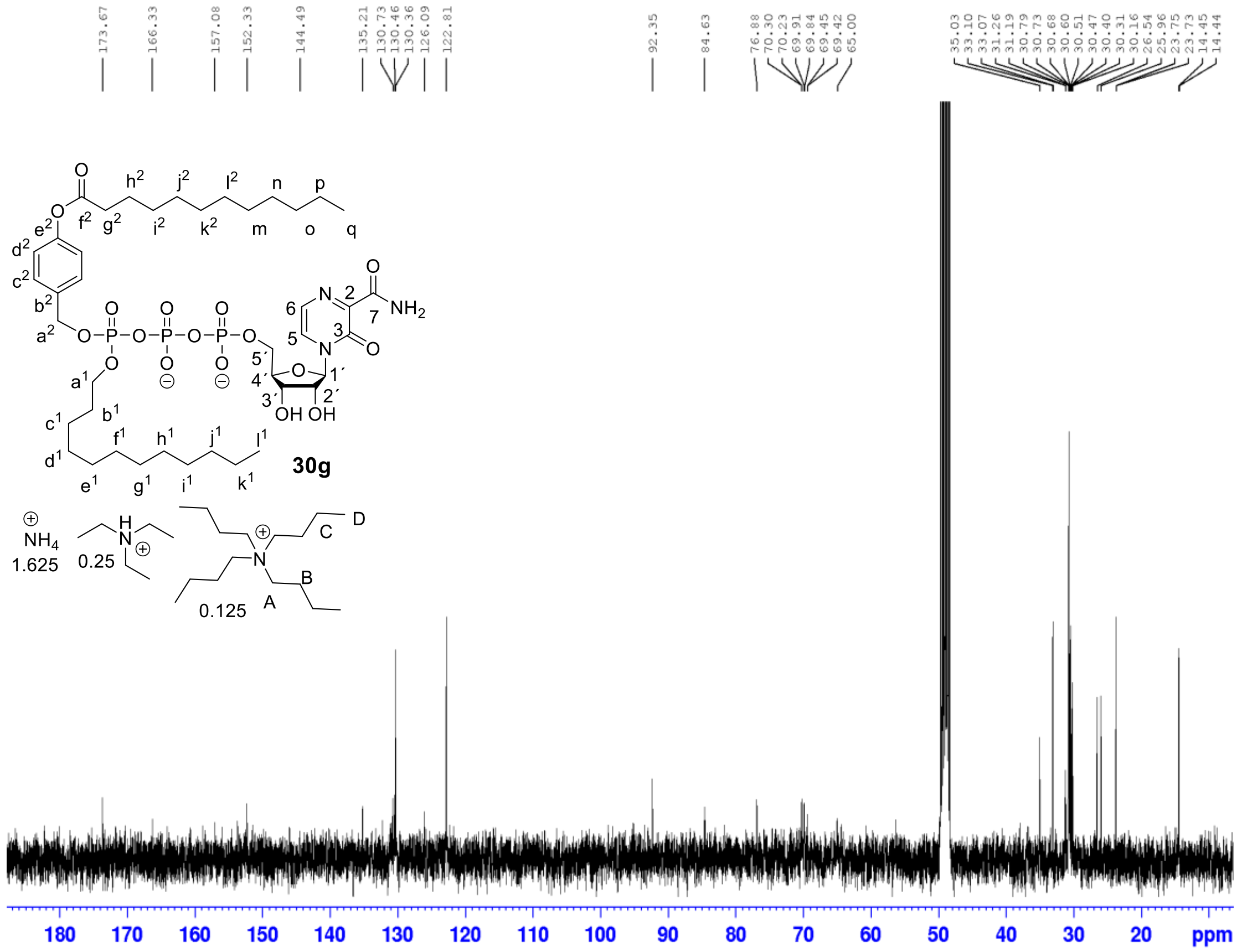
173.67
166.33
157.08
152.33
144.49
135.21
130.73
130.46
130.36
126.09
122.81
92.35
84.63
76.88
70.30
70.23
69.91
69.84
69.45
69.42
65.00
35.03
33.10
33.07
31.26
31.19
30.79
30.73
30.68
30.60
30.51
30.47
30.40
30.31
30.16
26.54
25.96
23.75
23.73
14.45
14.44
30g
1.625
0.25
0.125
180
170
160
150
140
130
120
110
100
90
80
70
60
50
40
30
20
ppm

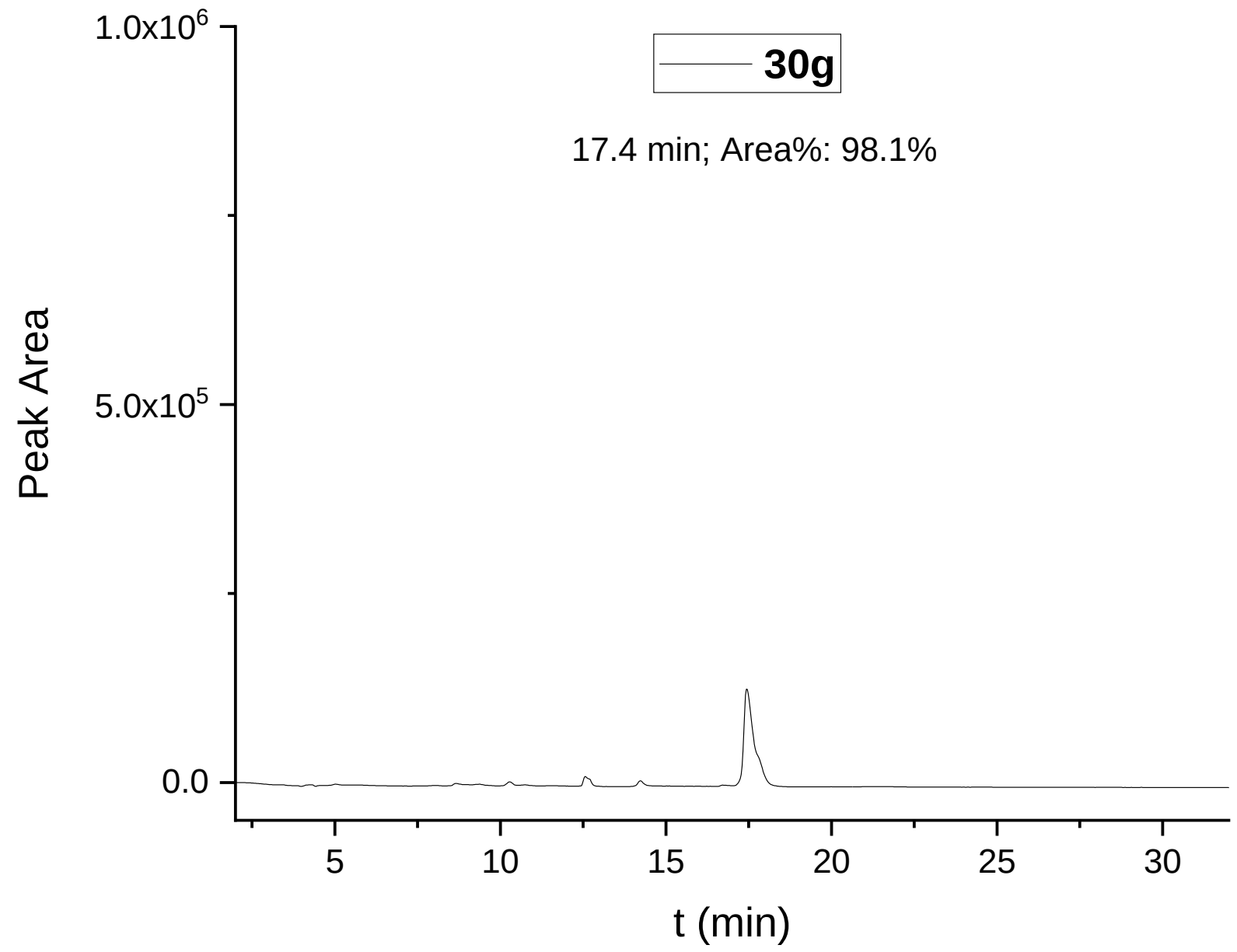
30g
17.4 min; Area%: 98.1%
Peak Area
1.0x10^6
5.0x10^5
0.0
5
10
15
20
25
30
t (min)

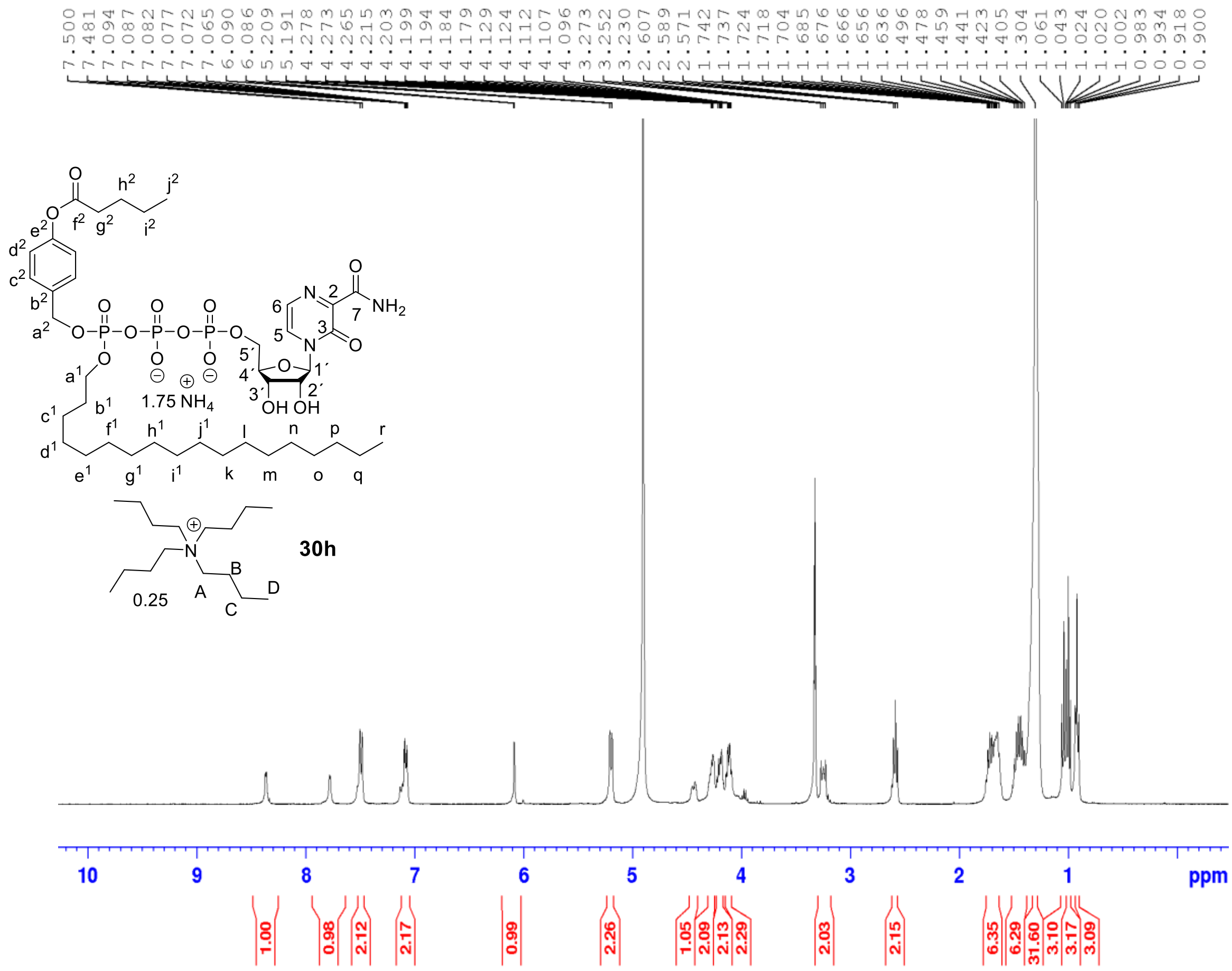

30h
1.75 NH4
0.25
ppm

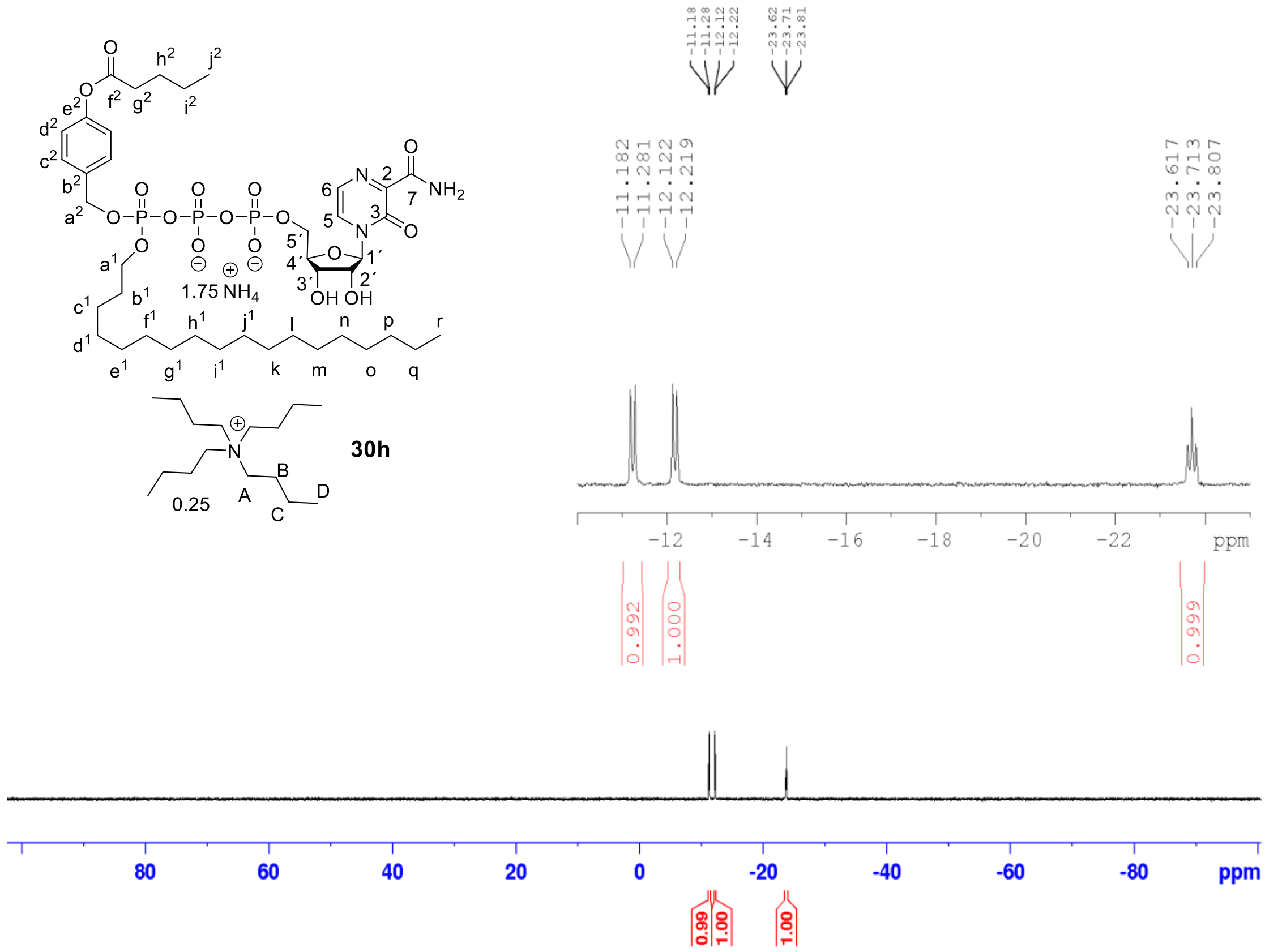

30h
1.75 NH4
0.25
ppm

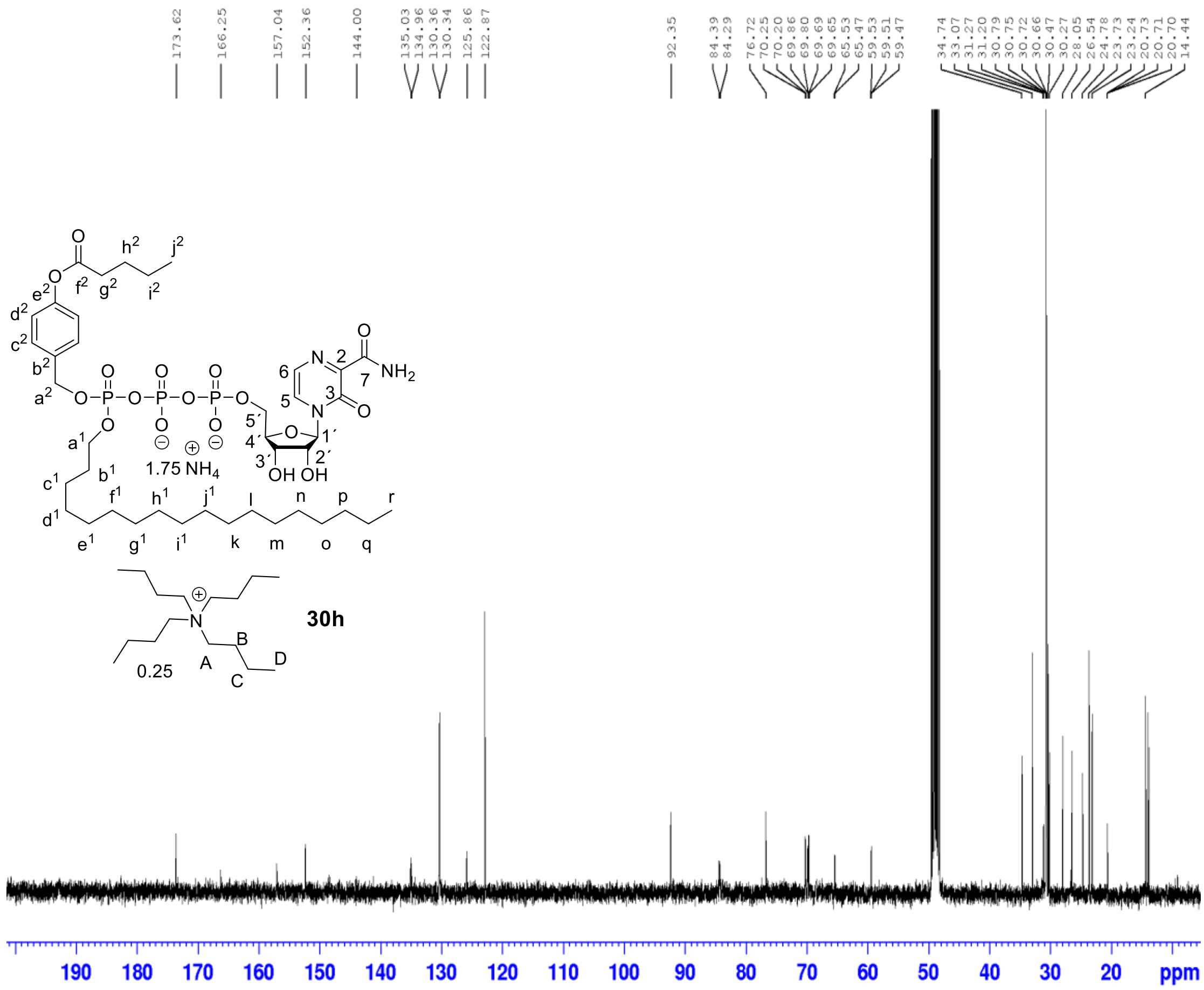
173.62
166.25
157.04
152.36
144.00
135.03
134.96
130.36
130.34
125.86
122.87
92.35
84.39
84.29
76.72
70.25
70.20
69.86
69.80
69.69
69.65
65.53
65.47
59.53
59.51
59.47
34.74
33.07
31.27
31.20
30.79
30.75
30.72
30.66
30.47
30.27
28.05
26.54
24.78
23.73
23.24
20.73
20.71
20.70
14.44
1.75 NH4
0.25
30h
190
180
170
160
150
140
130
120
110
100
90
80
70
60
50
40
30
20
ppm

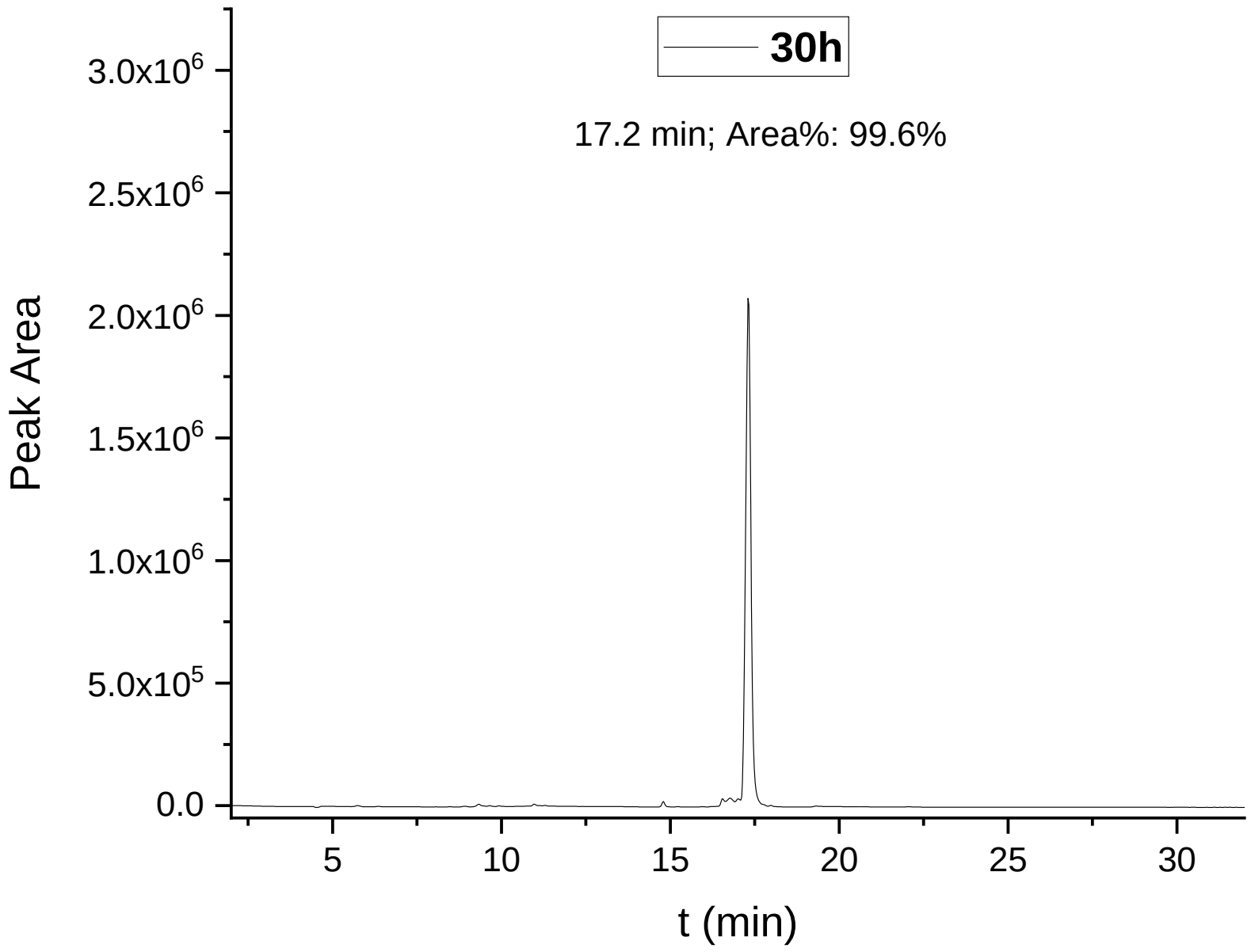
30h
17.2 min; Area%: 99.6%
Peak Area
3.0x10^6
2.5x10^6
2.0x10^6
1.5x10^6
1.0x10^6
5.0x10^5
0.0
5
10
15
20
25
30
t (min)

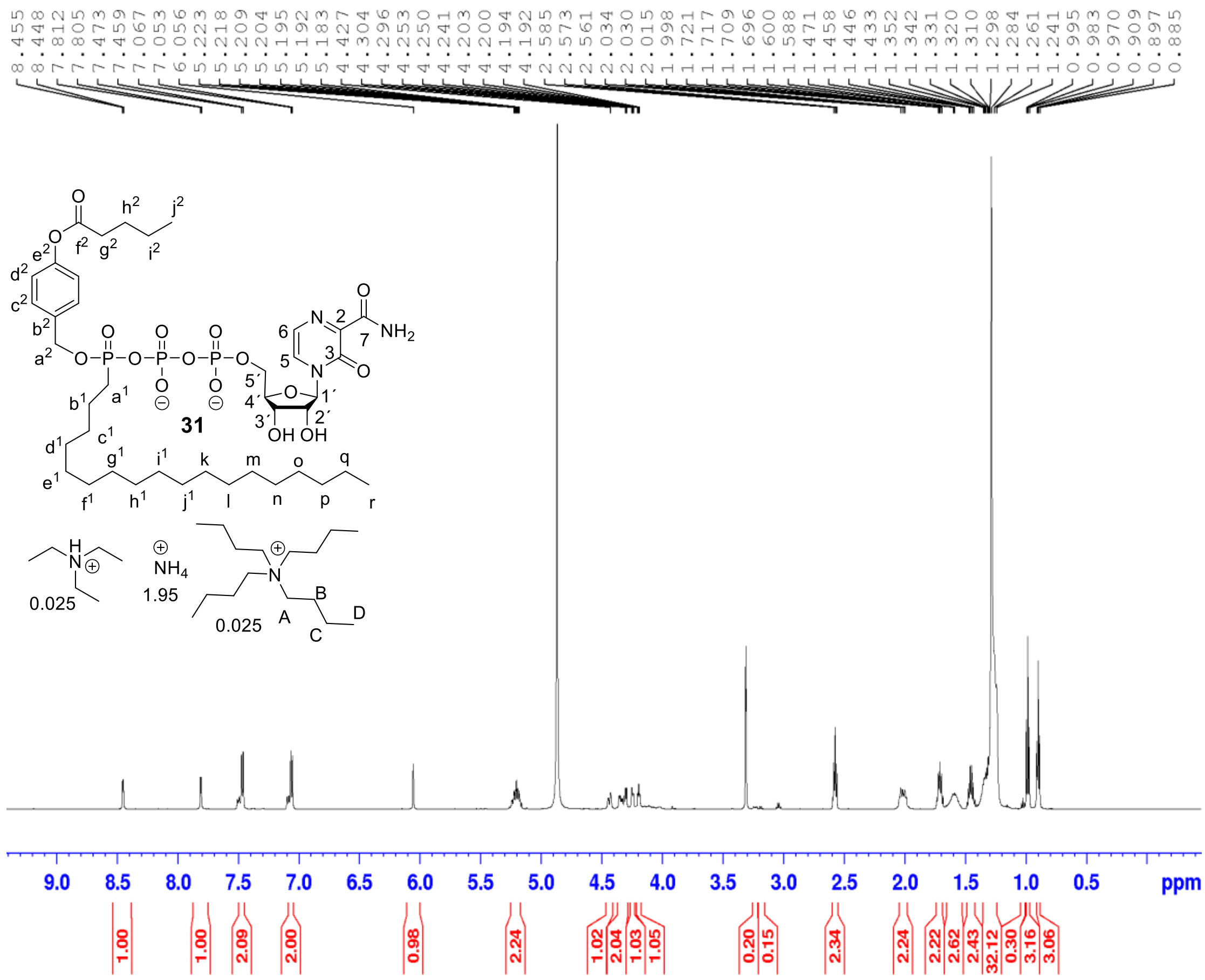

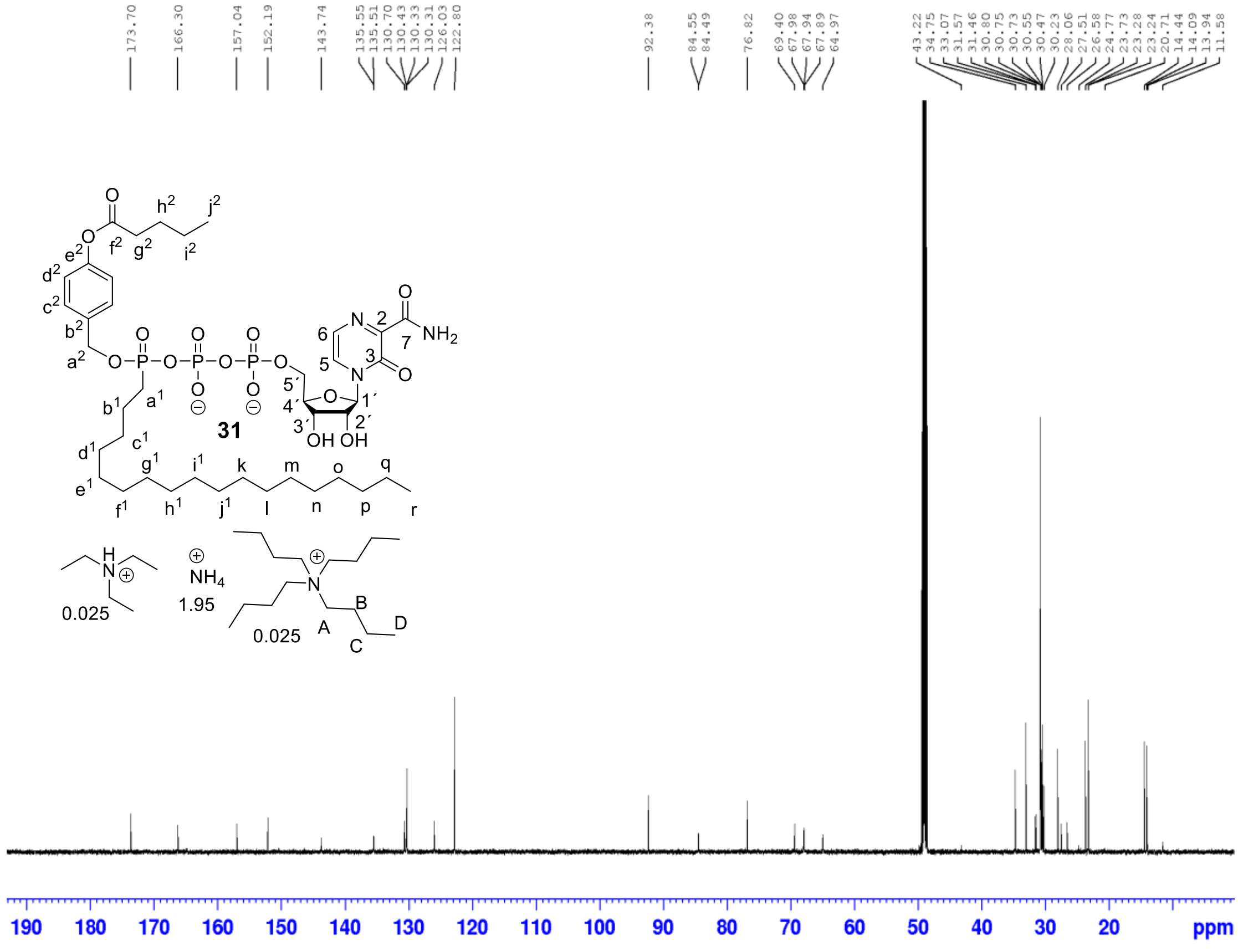

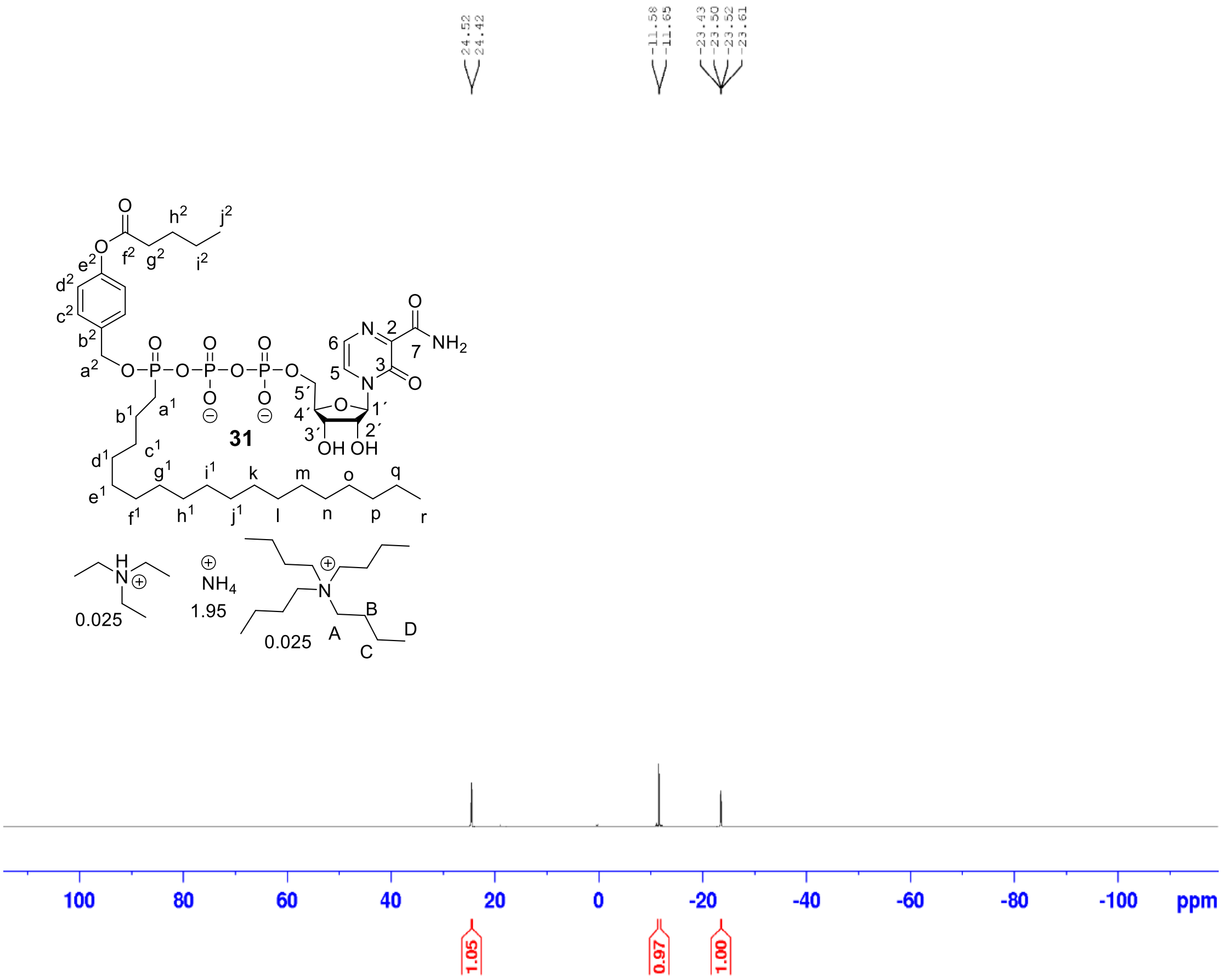
24.52
24.42
-11.58
-11.65
-23.43
-23.50
-23.52
-23.61
31
0.025
1.95
0.025
100
80
60
40
20
0
-20
-40
-60
-80
-100
ppm
1.05
0.97
1.00

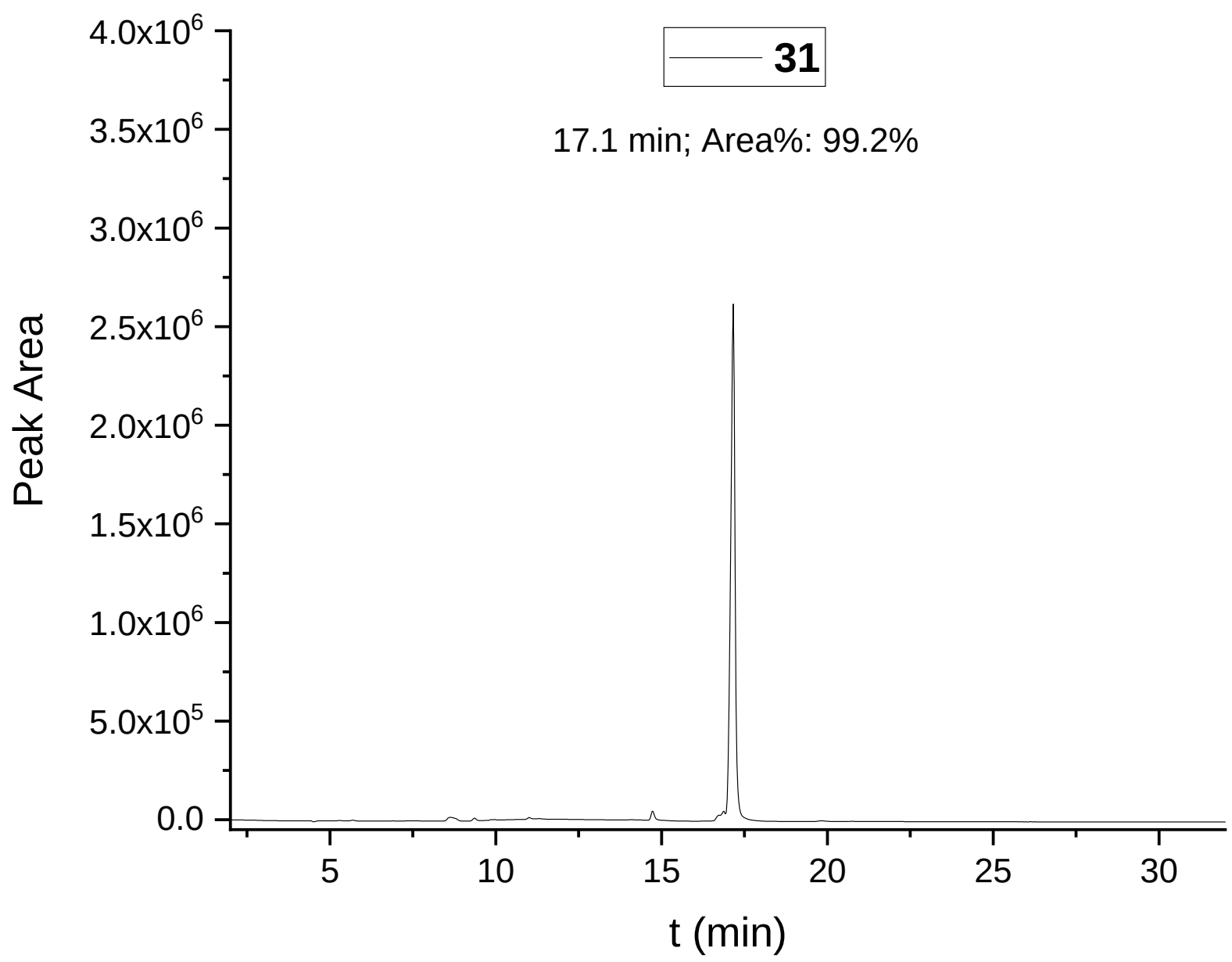
31
17.1 min; Area%: 99.2%
Peak Area
t (min)

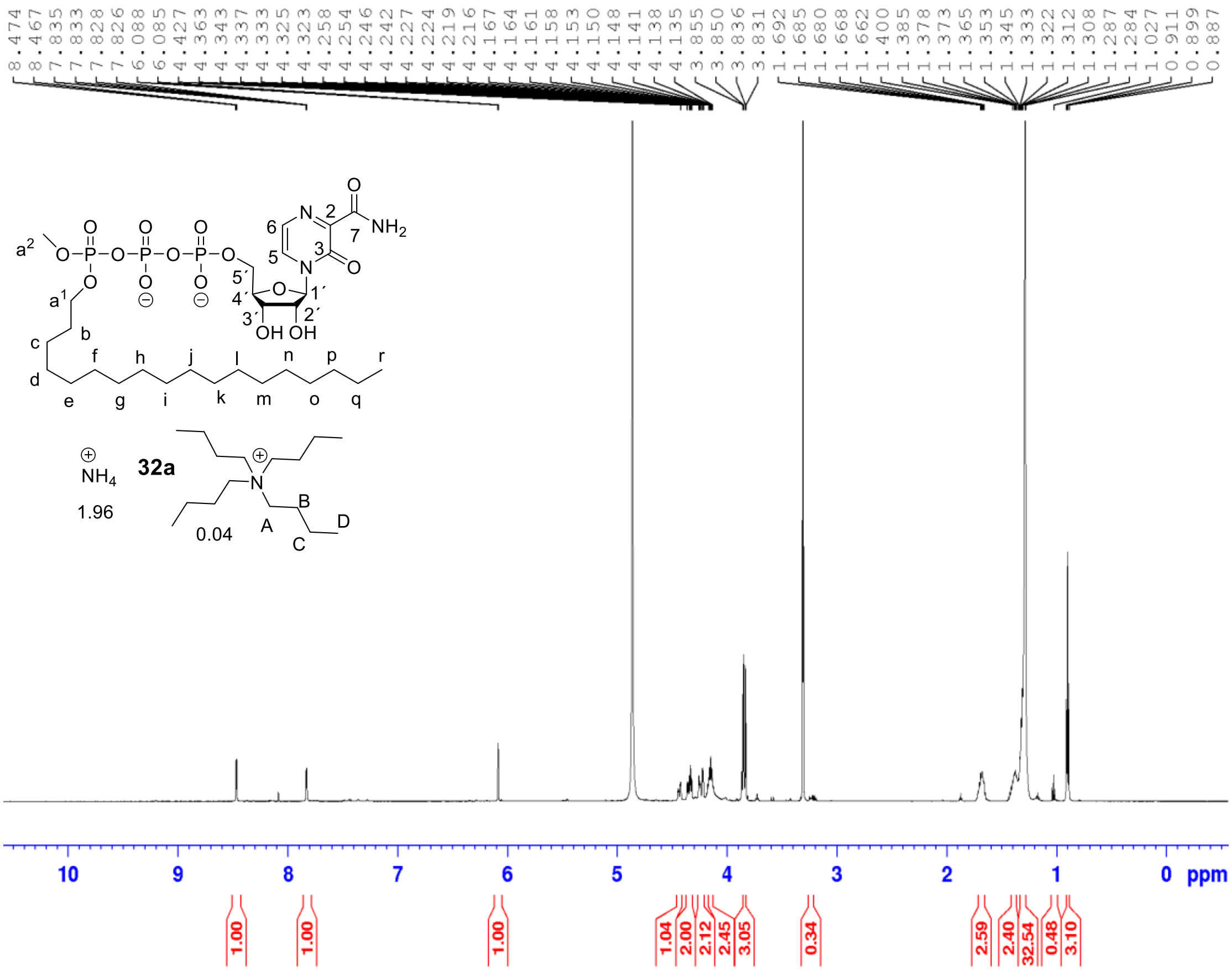
8.474 8.467 7.835 7.833 7.828 7.826 6.088 6.085 4.427 4.363 4.343 4.337 4.333 4.325 4.323 4.258 4.254 4.246 4.242 4.227 4.224 4.219 4.216 4.167 4.164 4.161 4.158 4.153 4.150 4.148 4.141 4.138 4.135 3.855 3.850 3.836 3.831 1.692 1.685 1.680 1.668 1.662 1.400 1.385 1.378 1.373 1.365 1.353 1.345 1.333 1.322 1.312 1.308 1.287 1.284 1.027 0.911 0.899 0.887
32a
NH4
1.96
0.04
1.00 1.00 1.00 1.04 2.00 2.12 2.45 3.05 0.34 2.59 2.40 32.54 0.48 3.10
10 9 8 7 6 5 4 3 2 1 0 ppm

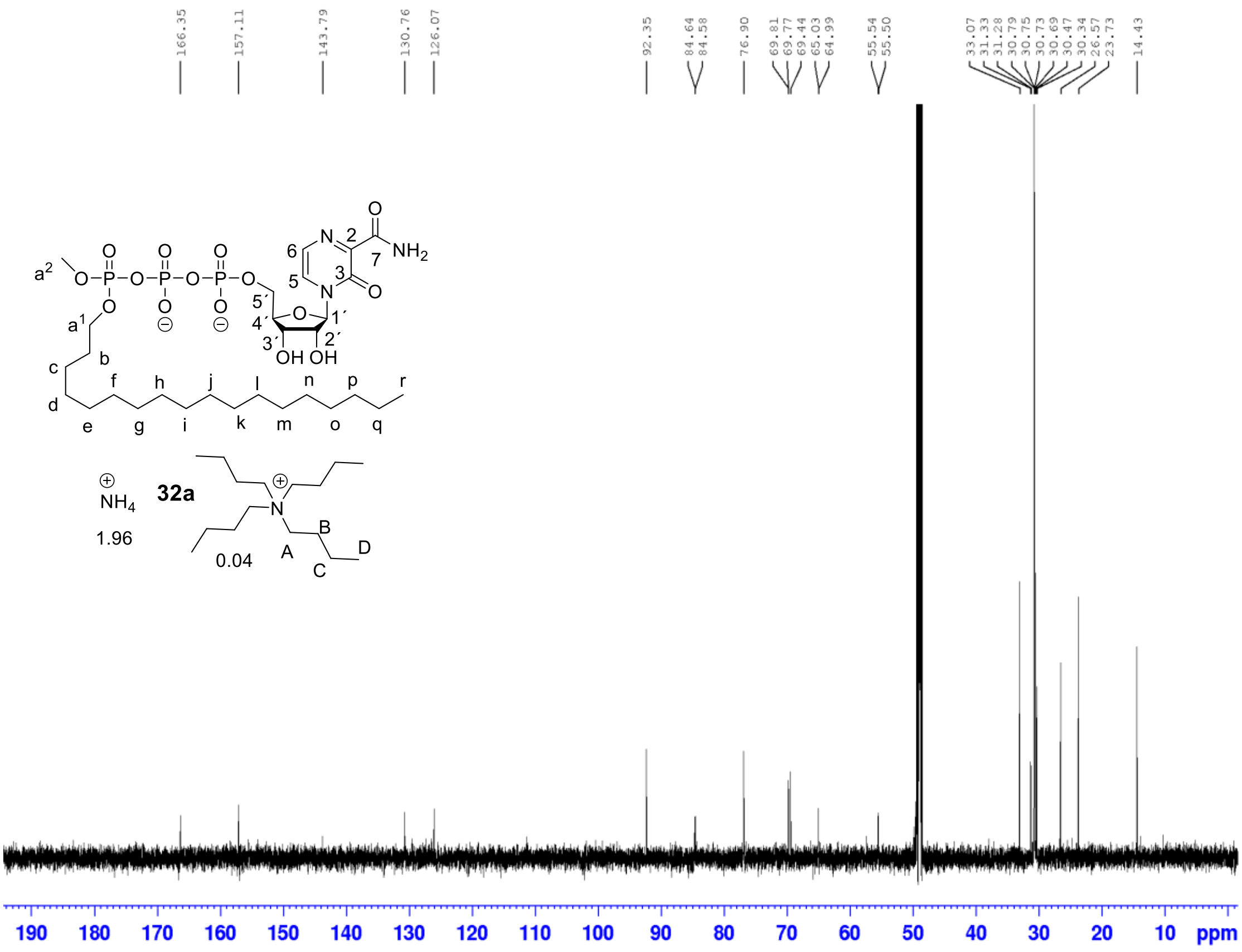
166.35 157.11 143.79 130.76 126.07 92.35 84.64 84.58 76.90 69.81 69.77 69.44 65.03 64.99 55.54 55.50 33.07 31.33 31.28 30.79 30.75 30.73 30.69 30.47 30.34 26.57 23.73 14.43
32a
NH4
1.96
0.04
190 180 170 160 150 140 130 120 110 100 90 80 70 60 50 40 30 20 10 ppm

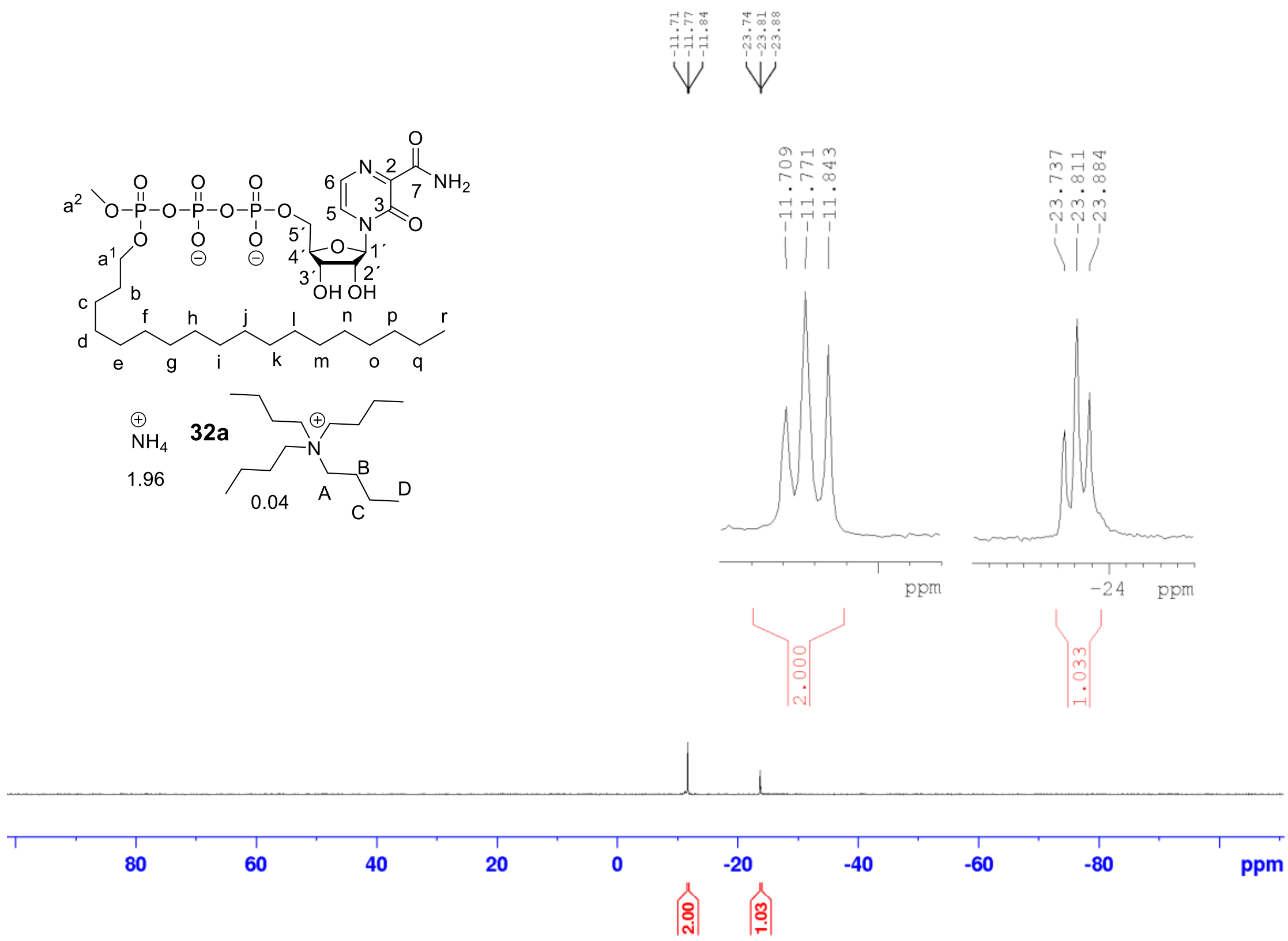
-11.709
-11.771
-11.843
-23.737
-23.811
-23.884
32a
NH4
1.96
0.04
ppm
-24
2.000
1.033
80
60
40
20
0
-20
-40
-60
-80
2.00
1.03

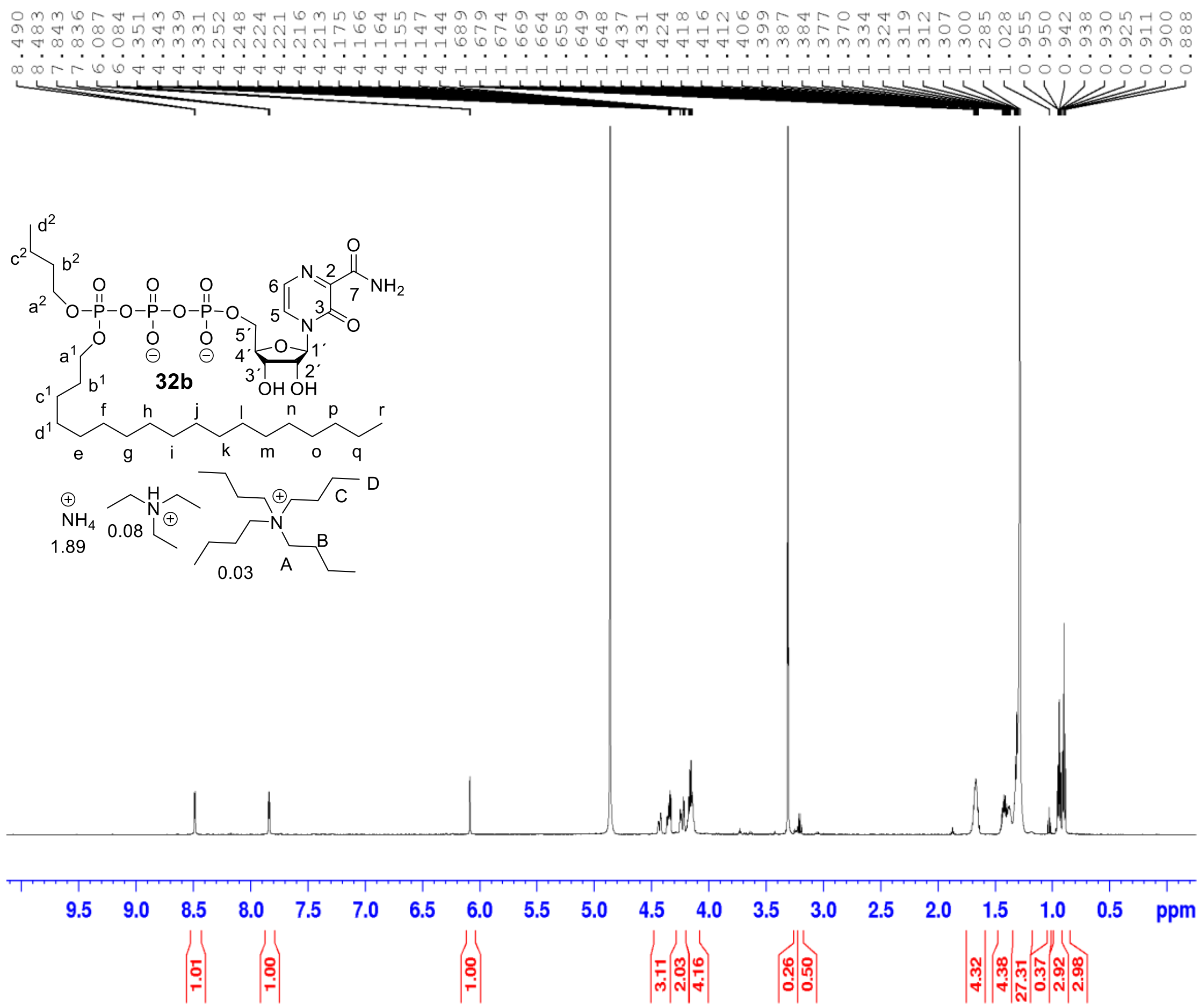
32b
NH4
1.89
0.08
0.03
9.5
9.0
8.5
8.0
7.5
7.0
6.5
6.0
5.5
5.0
4.5
4.0
3.5
3.0
2.5
2.0
1.5
1.0
0.5
ppm
1.01
1.00
1.00
3.11
2.03
4.16
0.26
0.50
4.32
4.38
27.31
0.37
2.92
2.98

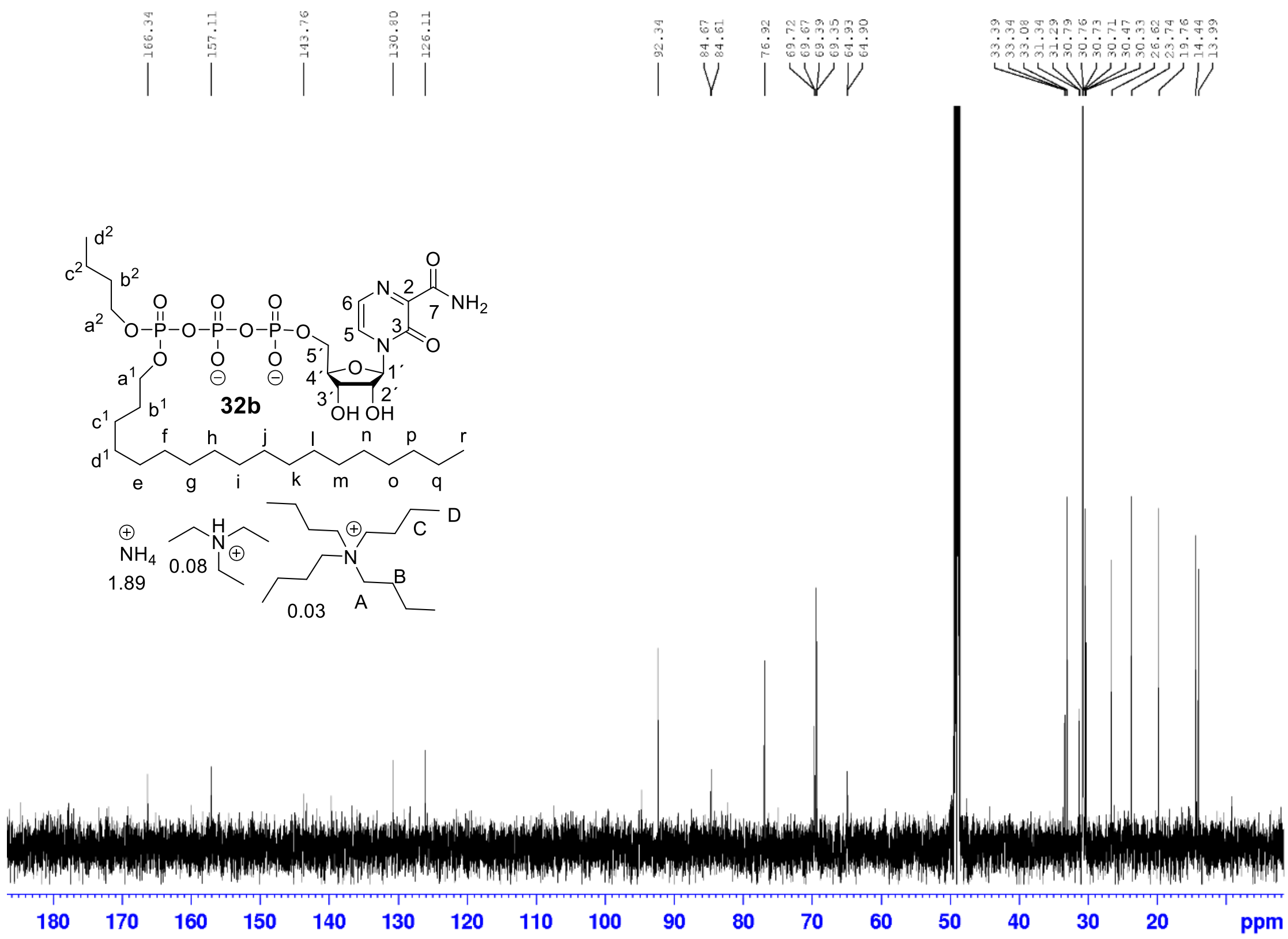
166.34
157.11
143.76
130.80
126.11
92.34
84.67
84.61
76.92
69.72
69.67
69.39
69.35
64.93
64.90
33.39
33.34
33.08
31.34
31.29
30.79
30.76
30.73
30.71
30.47
30.33
26.62
23.74
19.76
14.44
13.99
32b
NH4
1.89
0.08
0.03
A
B
C
D
ppm

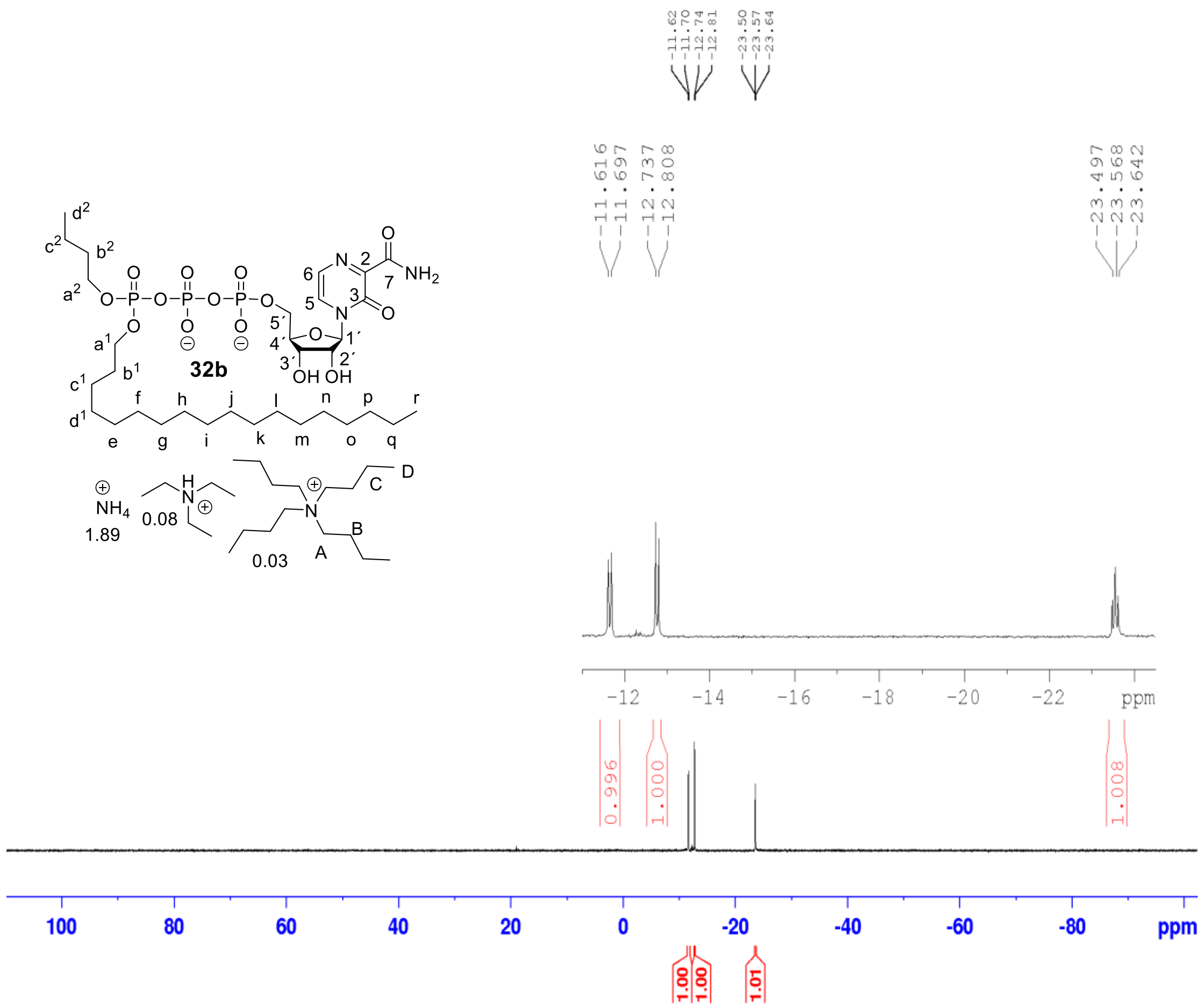
-11.62
-11.70
-12.74
-12.81
-23.50
-23.57
-23.64
-11.616
-11.697
-12.737
-12.808
-23.497
-23.568
-23.642
0.996
1.000
1.008
1.00
1.00
1.01
32b
NH4
1.89
0.08
0.03
ppm

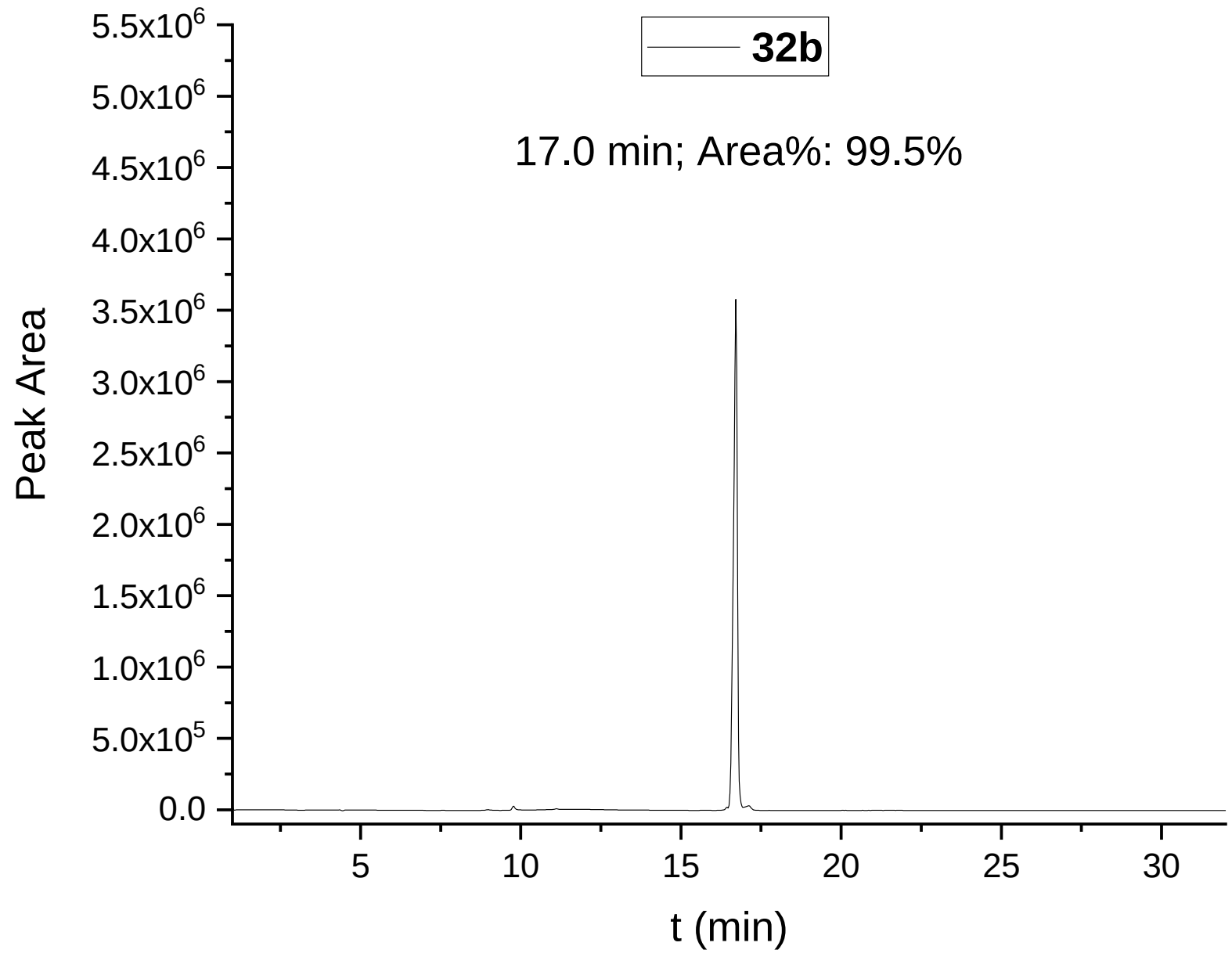
32b
17.0 min; Area%: 99.5%
Peak Area
t (min)

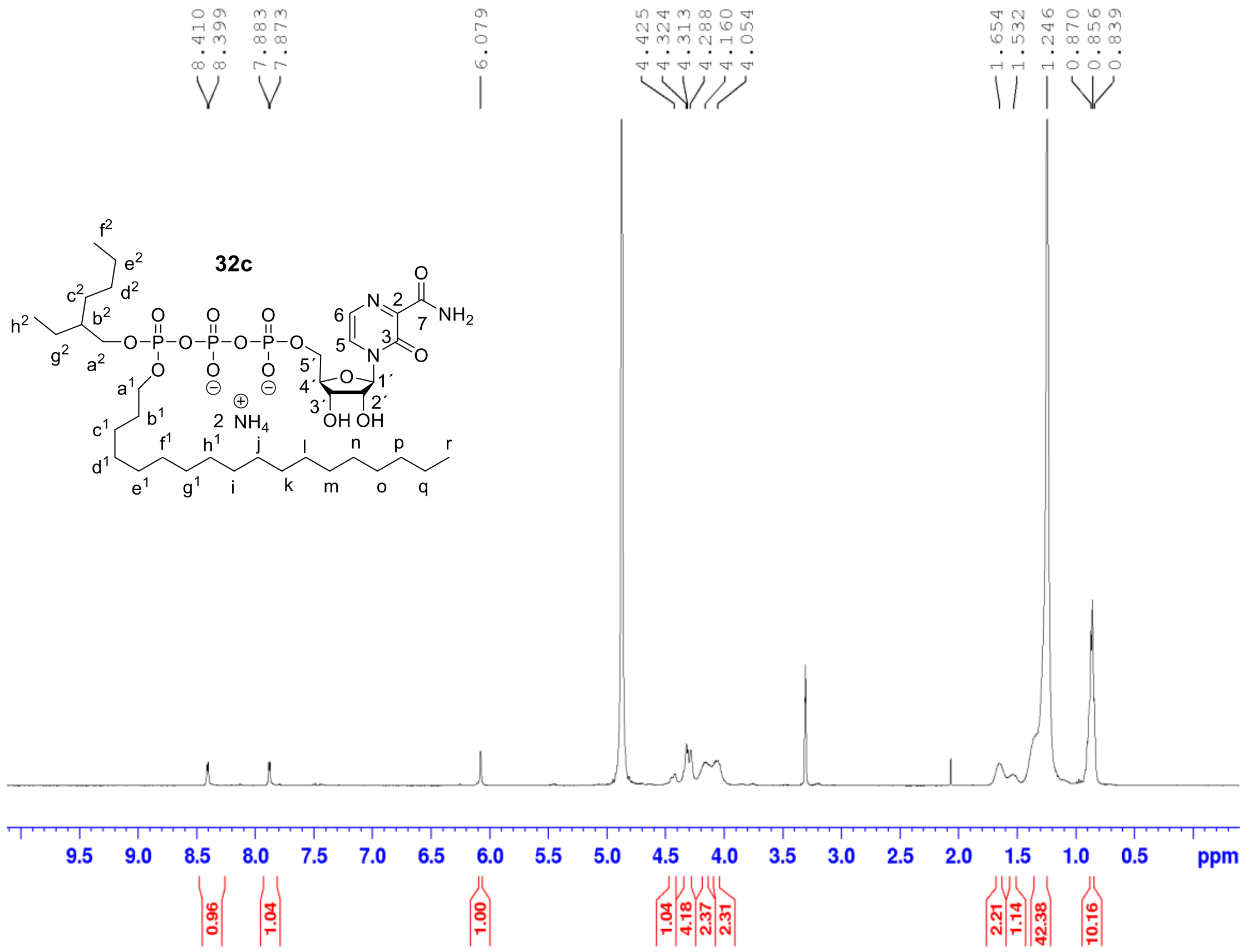
32c
8.410
8.399
7.883
7.873
6.079
4.425
4.324
4.313
4.288
4.160
4.054
1.654
1.532
1.246
0.870
0.856
0.839
ppm
0.96
1.04
1.00
1.04
4.18
2.37
2.31
2.21
1.14
42.38
10.16

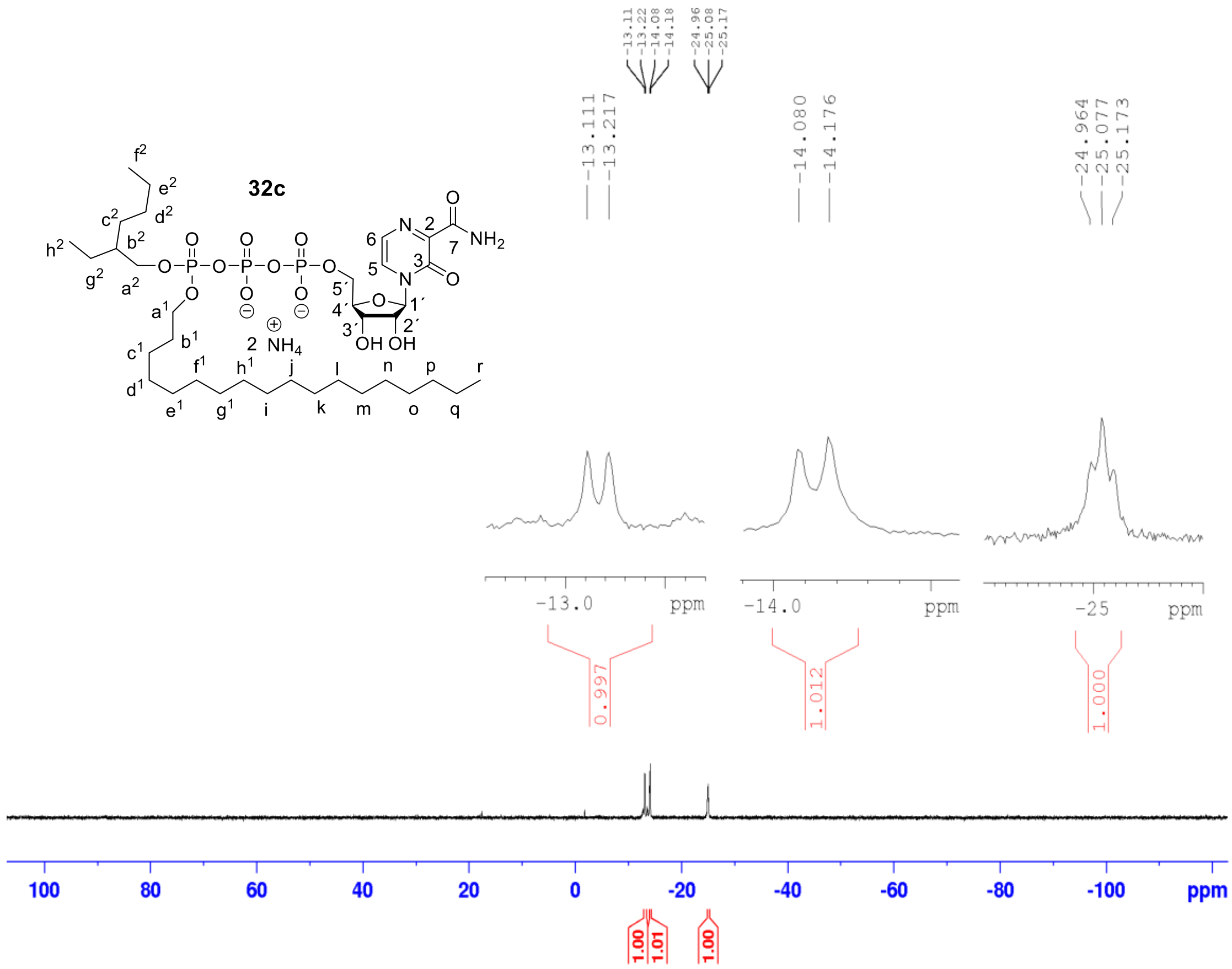
32c
NH2
2 NH4
-13.111
-13.217
-14.080
-14.176
-24.964
-25.077
-25.173
-13.0
-14.0
-25
ppm
0.997
1.012
1.000
1.00
1.01
1.00

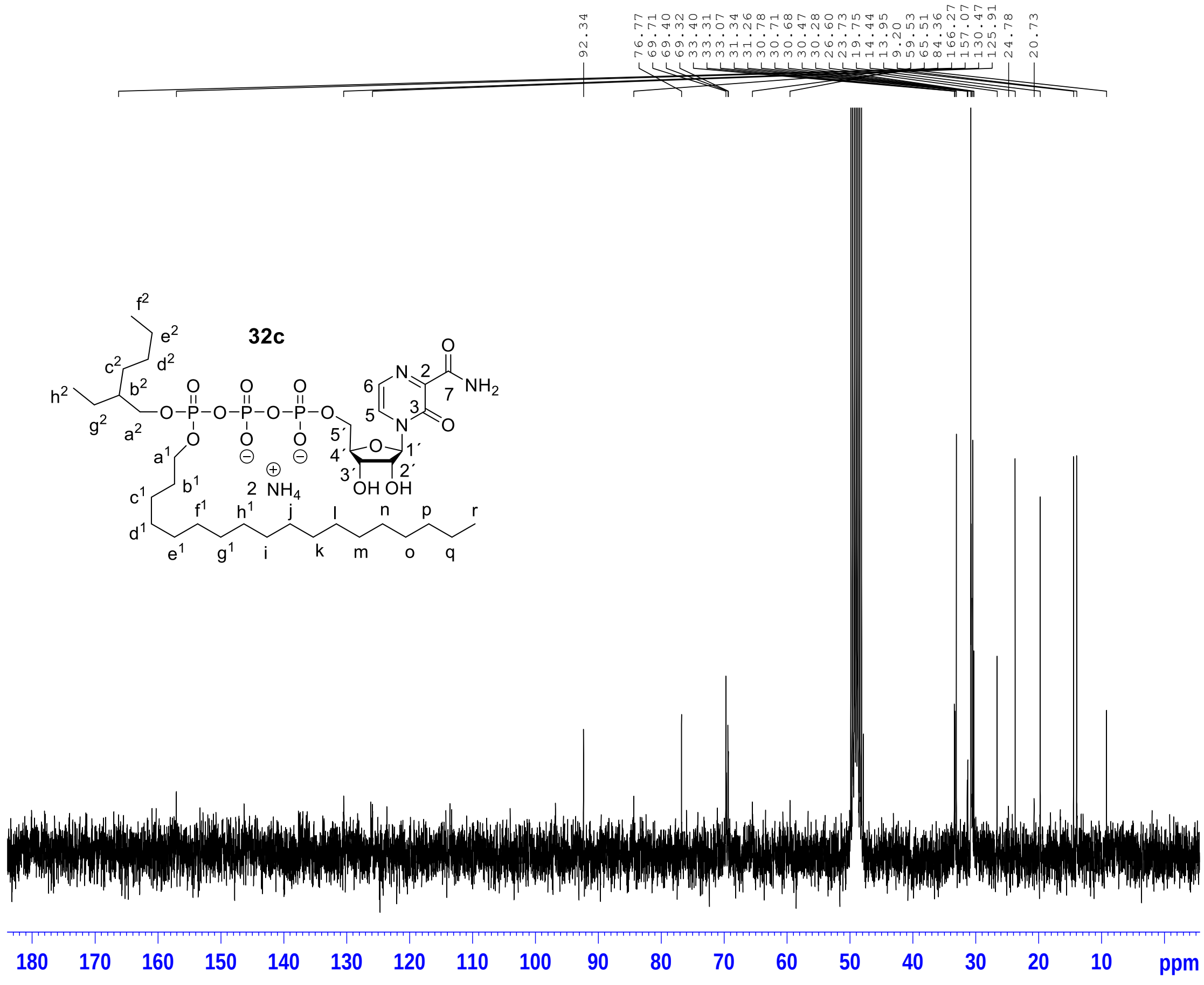
32c
NH2
2 NH4
92.34
76.77
69.71
69.40
69.32
33.40
33.07
31.34
31.26
30.78
30.71
30.68
30.47
30.28
26.60
23.73
19.75
14.44
13.95
9.20
59.53
65.51
84.36
166.27
157.07
130.47
125.91
24.78
20.73
ppm

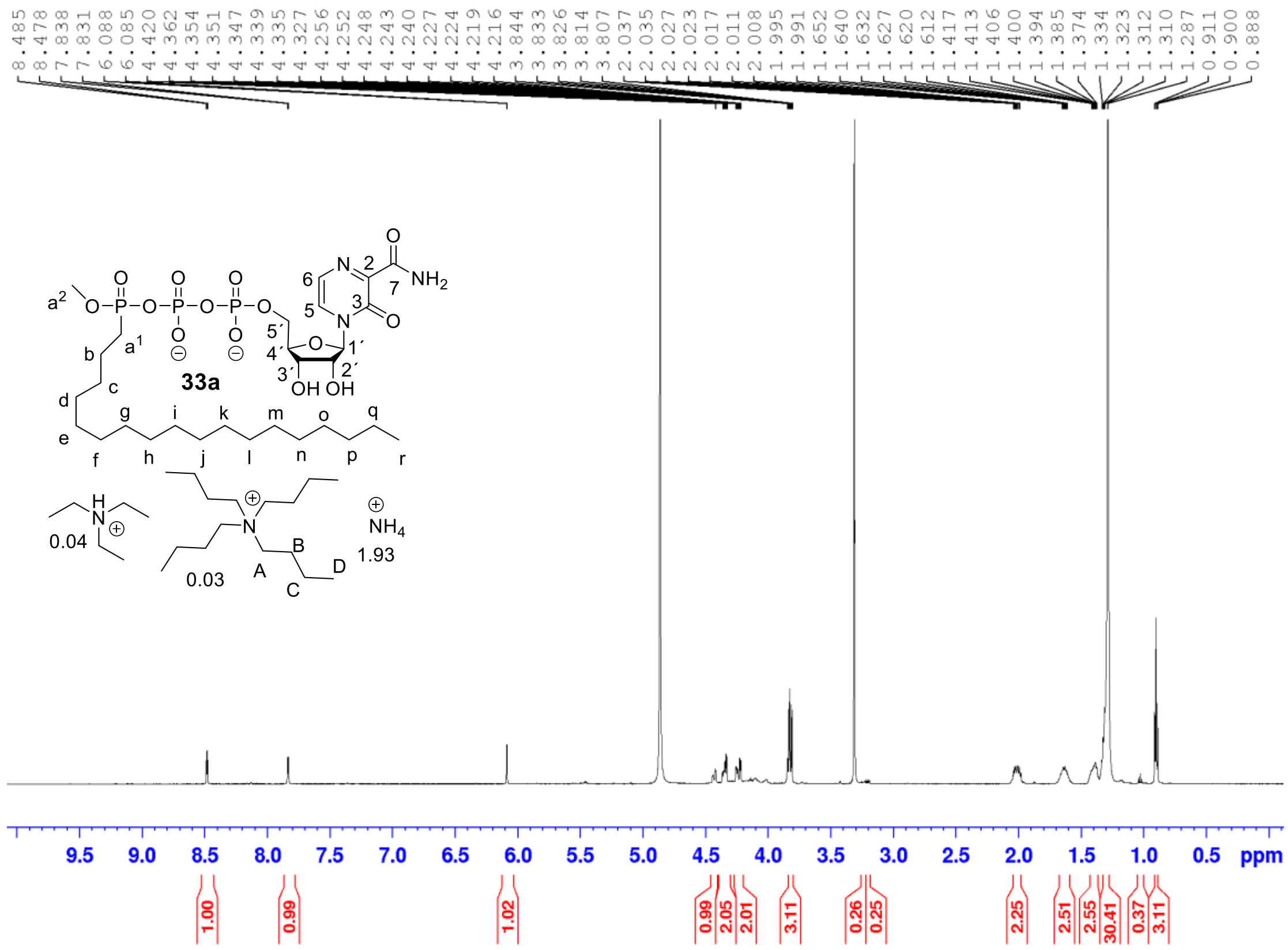
8.485 8.478 7.838 7.831 6.088 6.085 4.420 4.362 4.354 4.351 4.347 4.339 4.335 4.327 4.256 4.252 4.248 4.243 4.240 4.227 4.224 4.219 4.216 3.844 3.833 3.826 3.814 3.807 2.037 2.035 2.027 2.023 2.017 2.011 2.008 1.995 1.991 1.652 1.640 1.632 1.627 1.620 1.612 1.417 1.413 1.406 1.400 1.394 1.385 1.374 1.334 1.323 1.312 1.310 1.287 0.911 0.900 0.888
33a
0.04
0.03
1.93
9.5 9.0 8.5 8.0 7.5 7.0 6.5 6.0 5.5 5.0 4.5 4.0 3.5 3.0 2.5 2.0 1.5 1.0 0.5 ppm
1.00 0.99 1.02 0.99 2.05 2.01 3.11 0.26 0.25 2.25 2.51 2.55 30.41 0.37 3.11

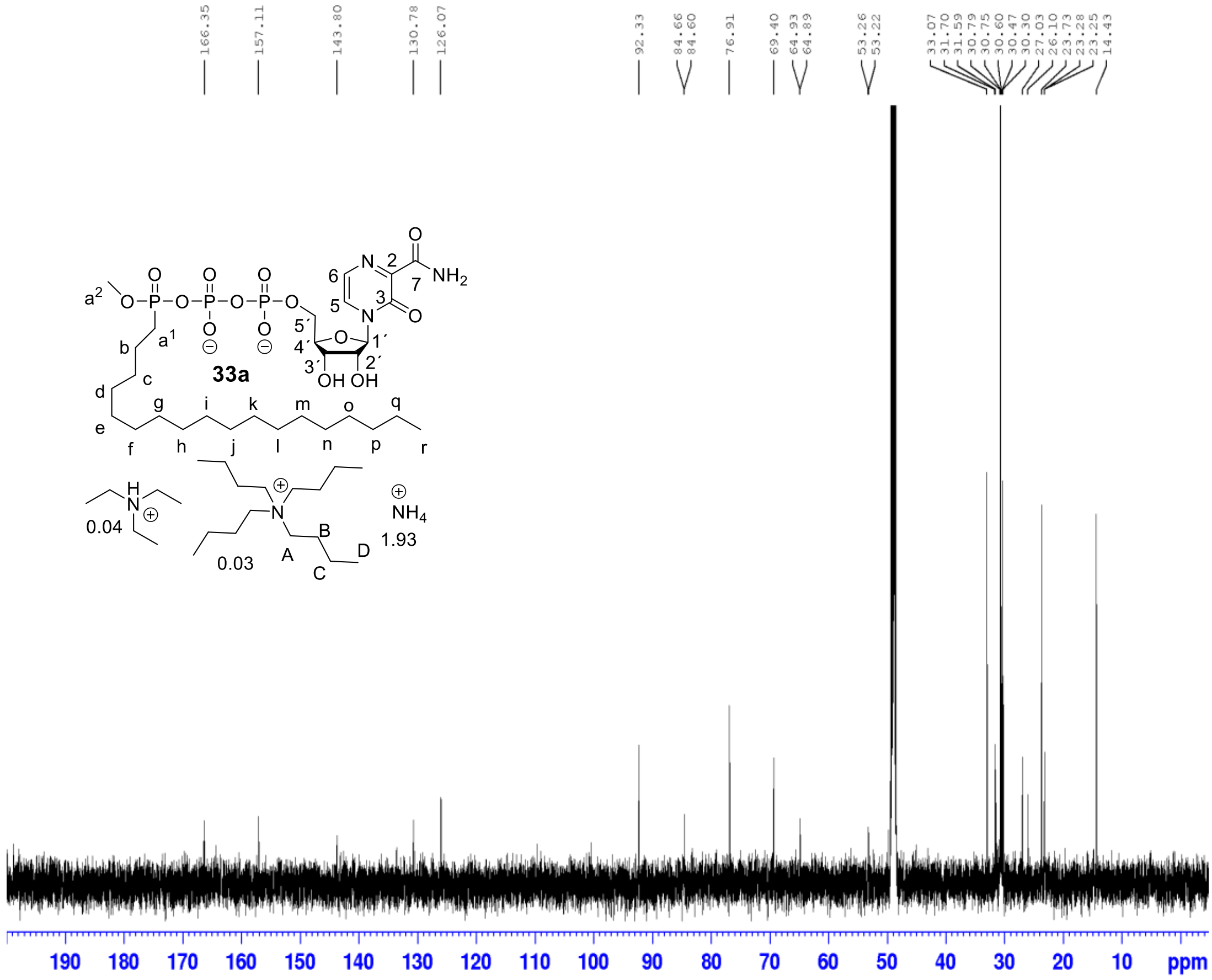
166.35 157.11 143.80 130.78 126.07 92.33 84.66 84.60 76.91 69.40 64.93 64.89 53.26 53.22 33.07 31.70 31.59 30.79 30.75 30.60 30.47 30.30 27.03 26.10 23.73 23.28 23.25 14.43
33a
0.04
0.03
1.93
190 180 170 160 150 140 130 120 110 100 90 80 70 60 50 40 30 20 10 ppm

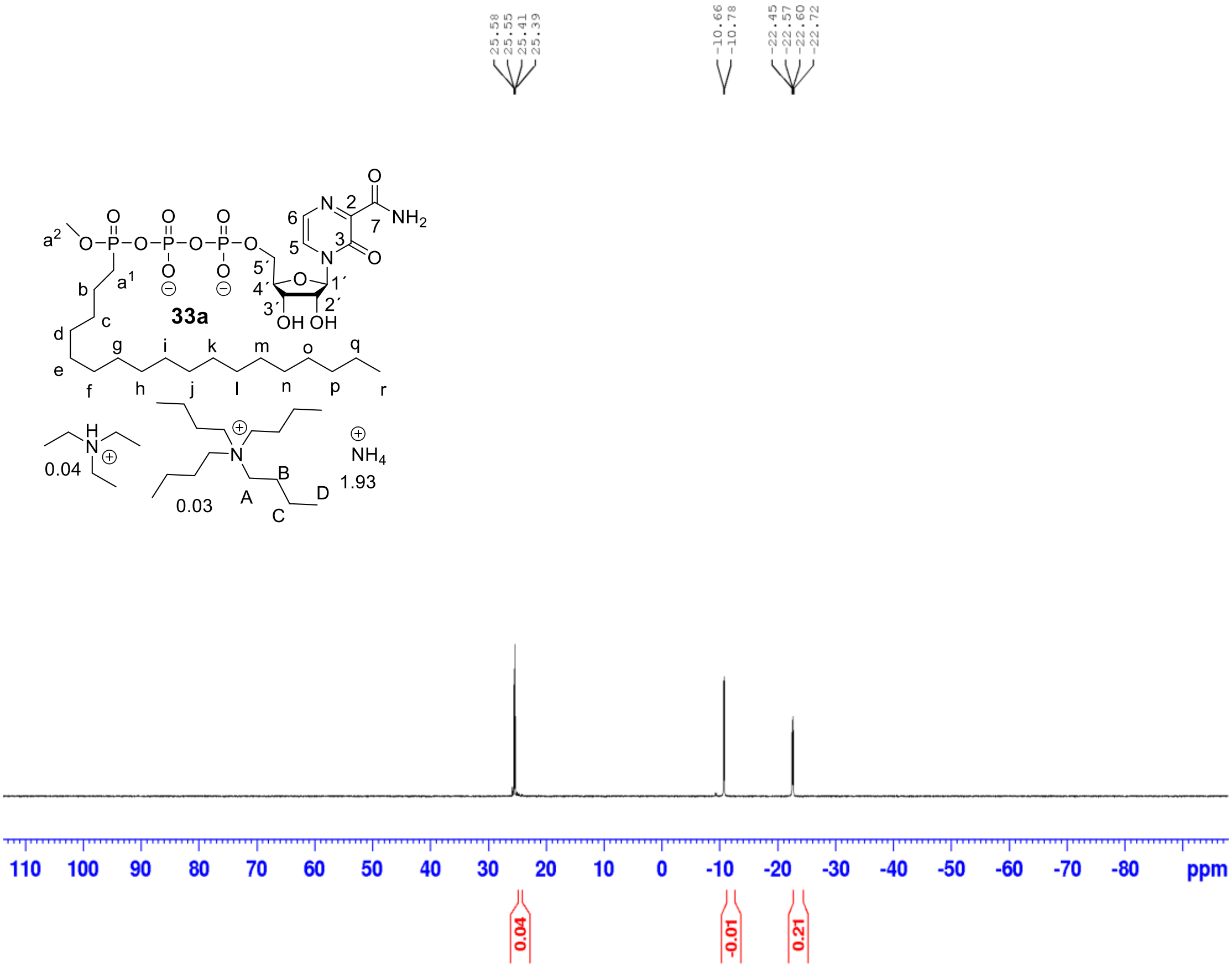
33a
0.04
0.03
1.93
25.58
25.55
25.41
25.39
-10.66
-10.78
-22.45
-22.57
-22.60
-22.72
110 100 90 80 70 60 50 40 30 20 10 0 -10 -20 -30 -40 -50 -60 -70 -80 ppm
0.04
-0.01
0.21

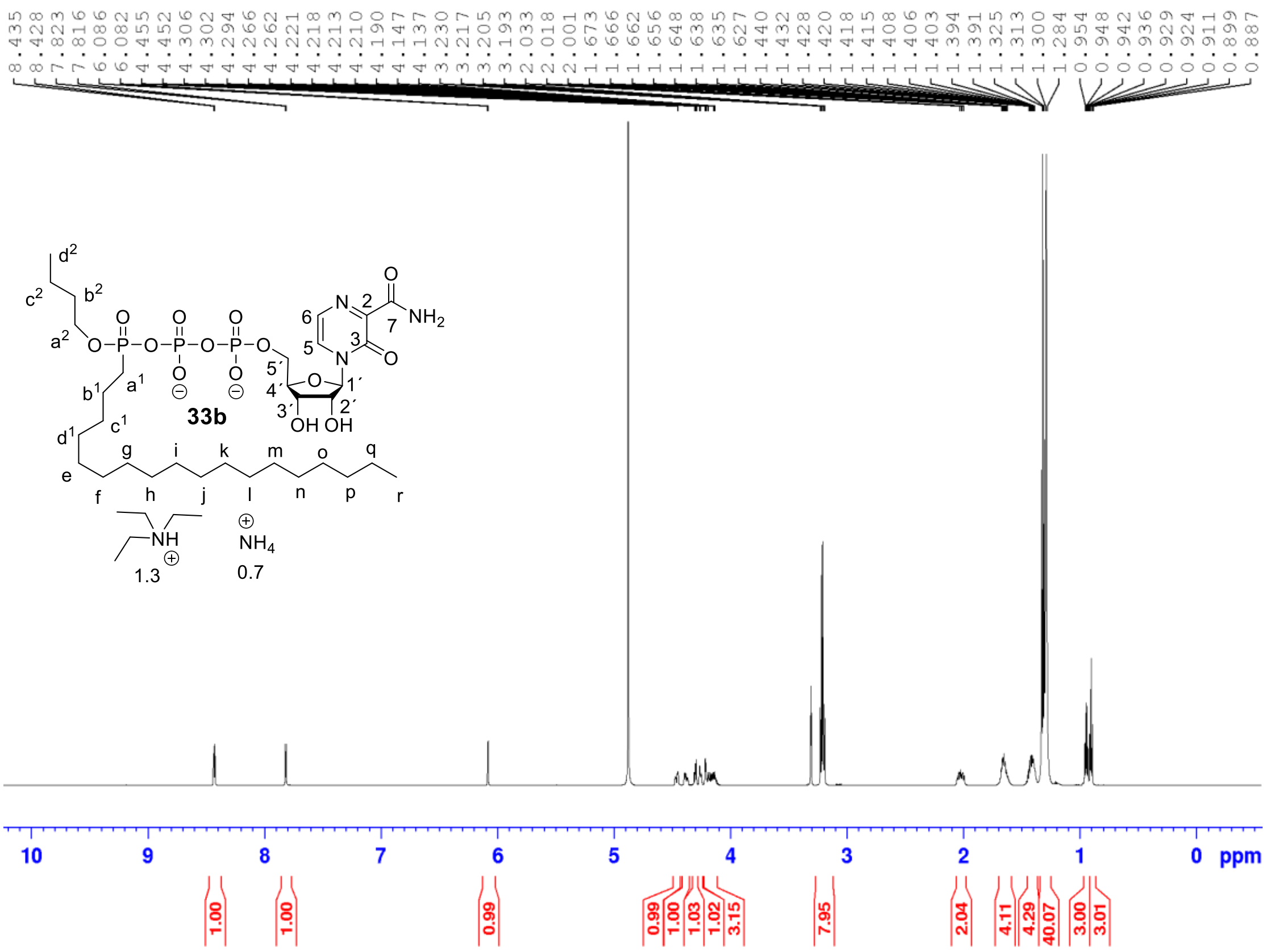
33b
1.3
0.7
8.435 8.428 7.823 7.816 6.086 6.082 4.455 4.452 4.306 4.302 4.294 4.266 4.262 4.221 4.218 4.213 4.210 4.190 4.147 4.137 3.230 3.217 3.205 3.193 2.033 2.018 2.001 1.673 1.666 1.662 1.656 1.648 1.638 1.635 1.627 1.440 1.432 1.428 1.420 1.418 1.415 1.408 1.406 1.403 1.394 1.391 1.325 1.313 1.300 1.284 0.954 0.948 0.942 0.936 0.929 0.924 0.911 0.899 0.887
10 9 8 7 6 5 4 3 2 1 0 ppm
1.00 1.00 0.99 0.99 1.00 1.03 1.02 3.15 7.95 2.04 4.11 4.29 40.07 3.00 3.01

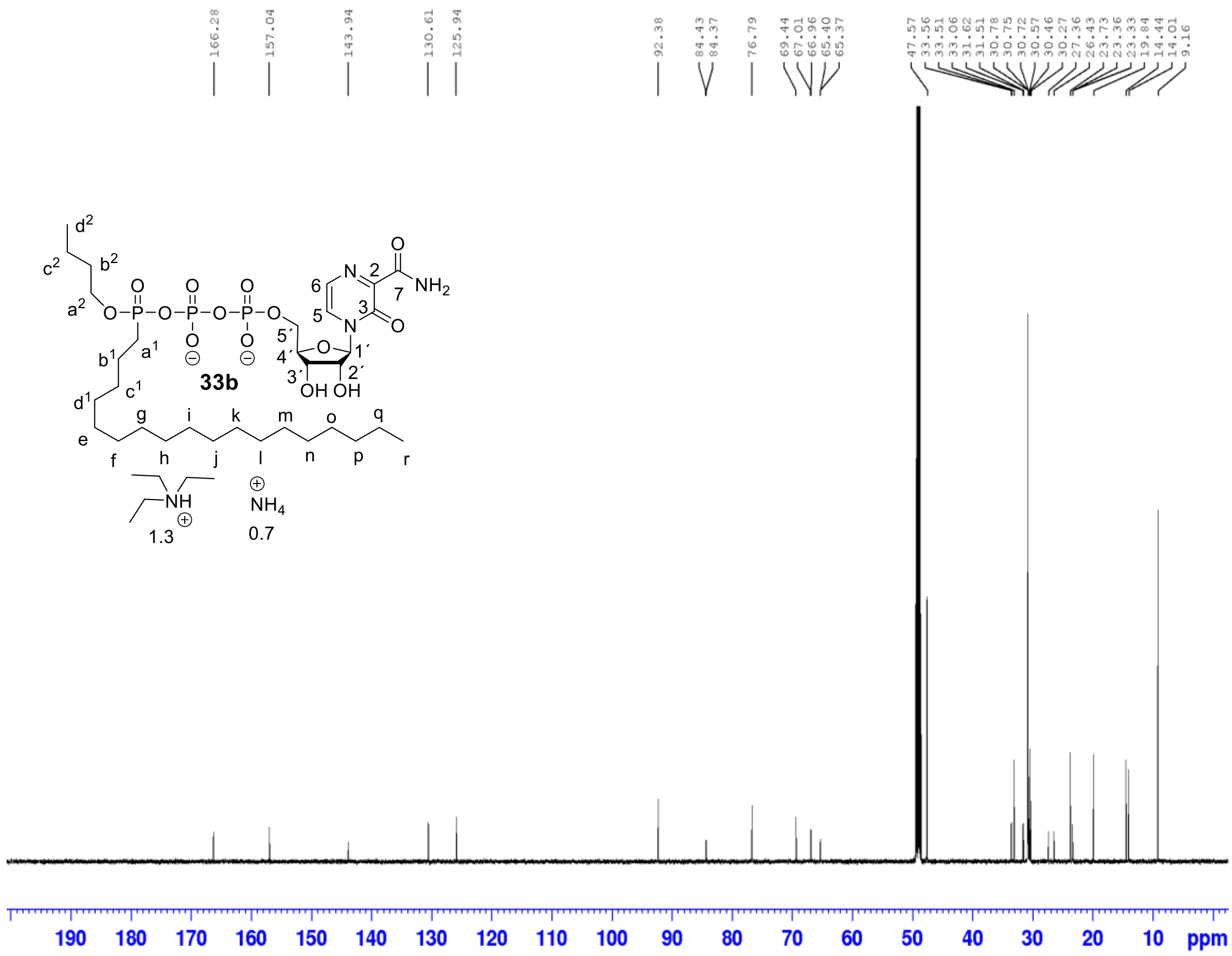
166.28
157.04
143.94
130.61
125.94
92.38
84.43
84.37
76.79
69.44
67.01
66.96
65.40
65.37
47.57
33.56
33.51
33.06
31.62
31.51
30.78
30.75
30.72
30.57
30.46
30.27
27.36
26.43
23.73
23.36
23.33
19.84
14.44
14.01
9.16
33b
1.3
0.7
ppm

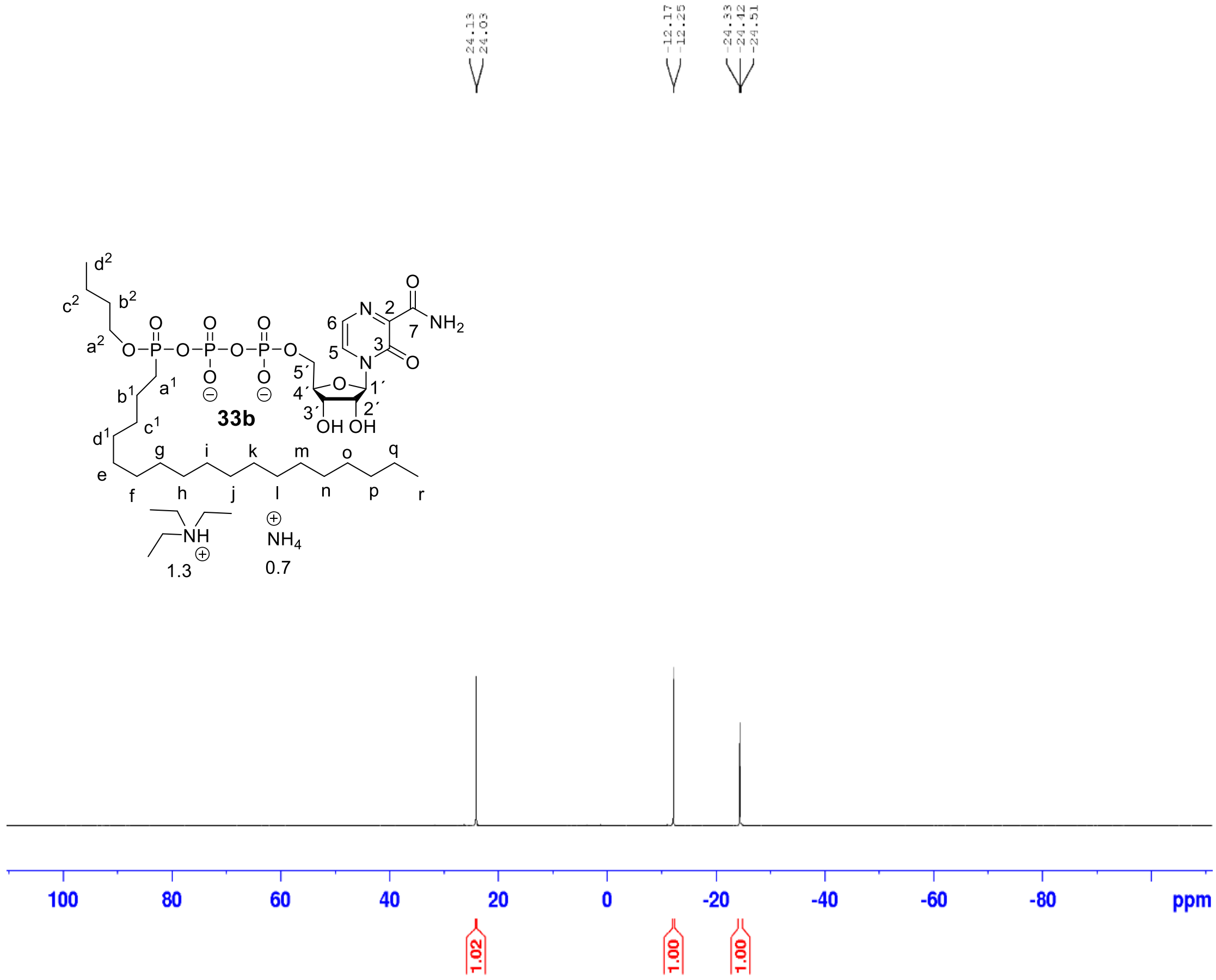
24.13
24.03
-12.17
-12.25
-24.33
-24.42
-24.51
33b
1.3
0.7
ppm
1.02
1.00
1.00

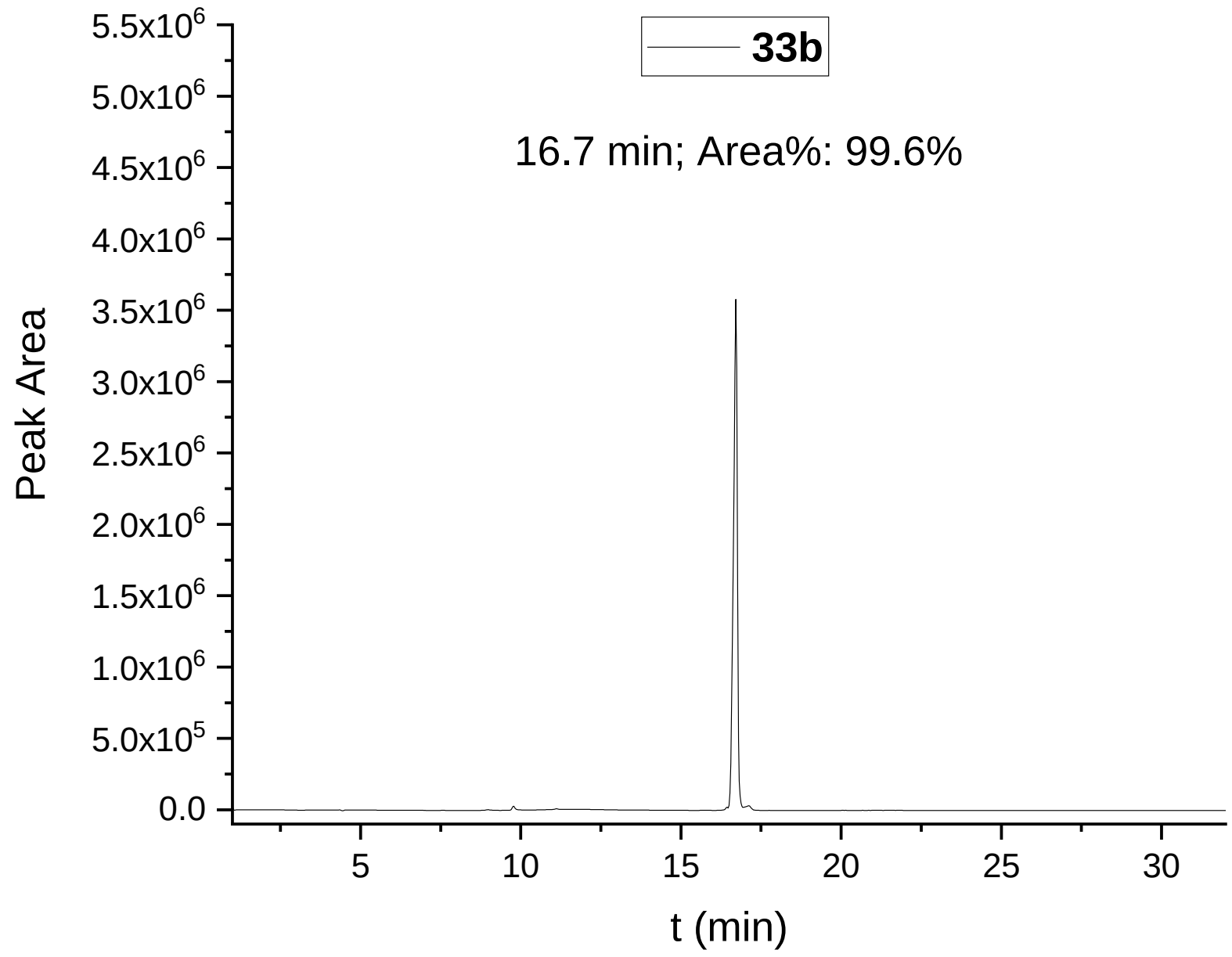
33b
16.7 min; Area%: 99.6%
Peak Area
t (min)

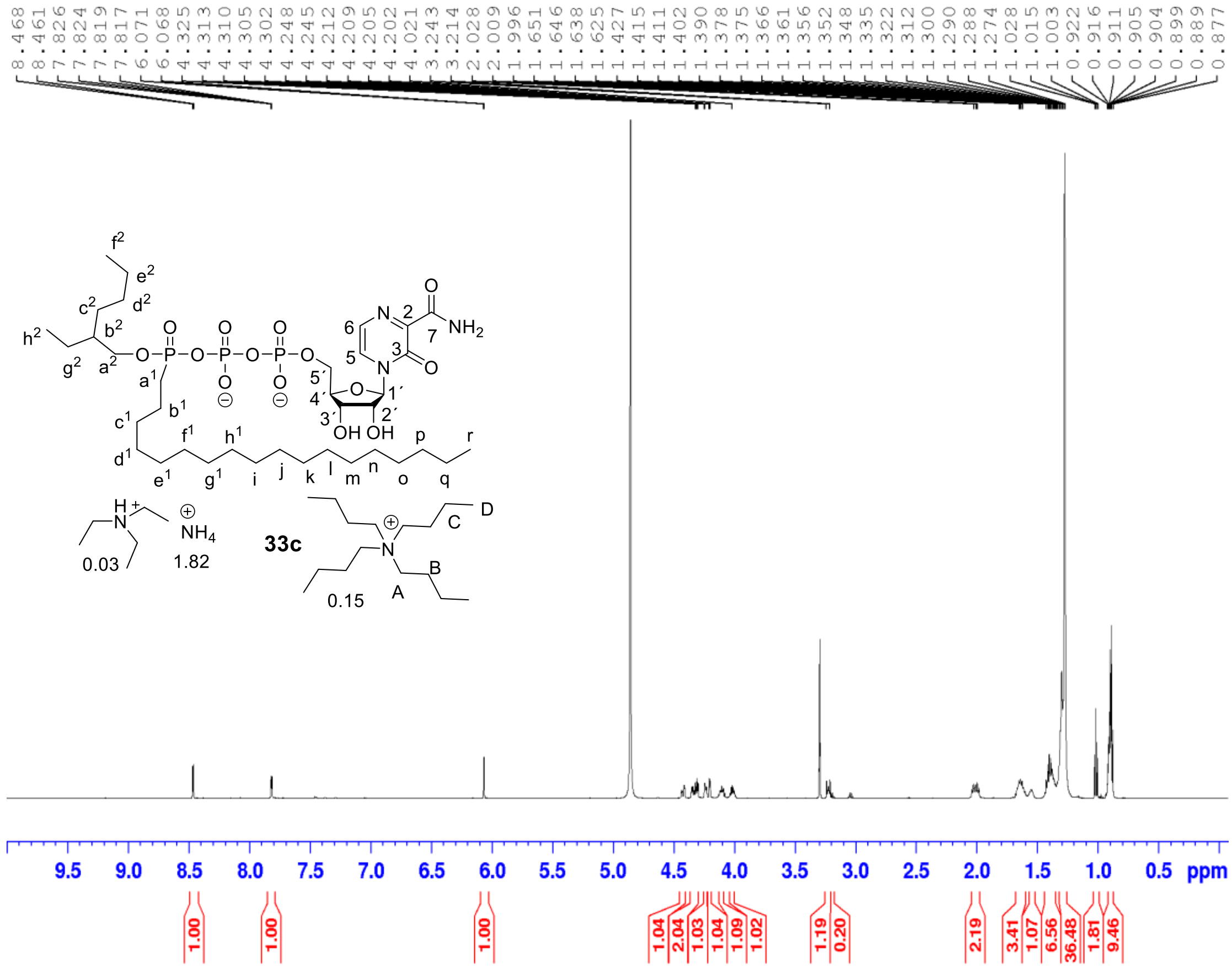
33c
0.03
1.82
0.15
ppm

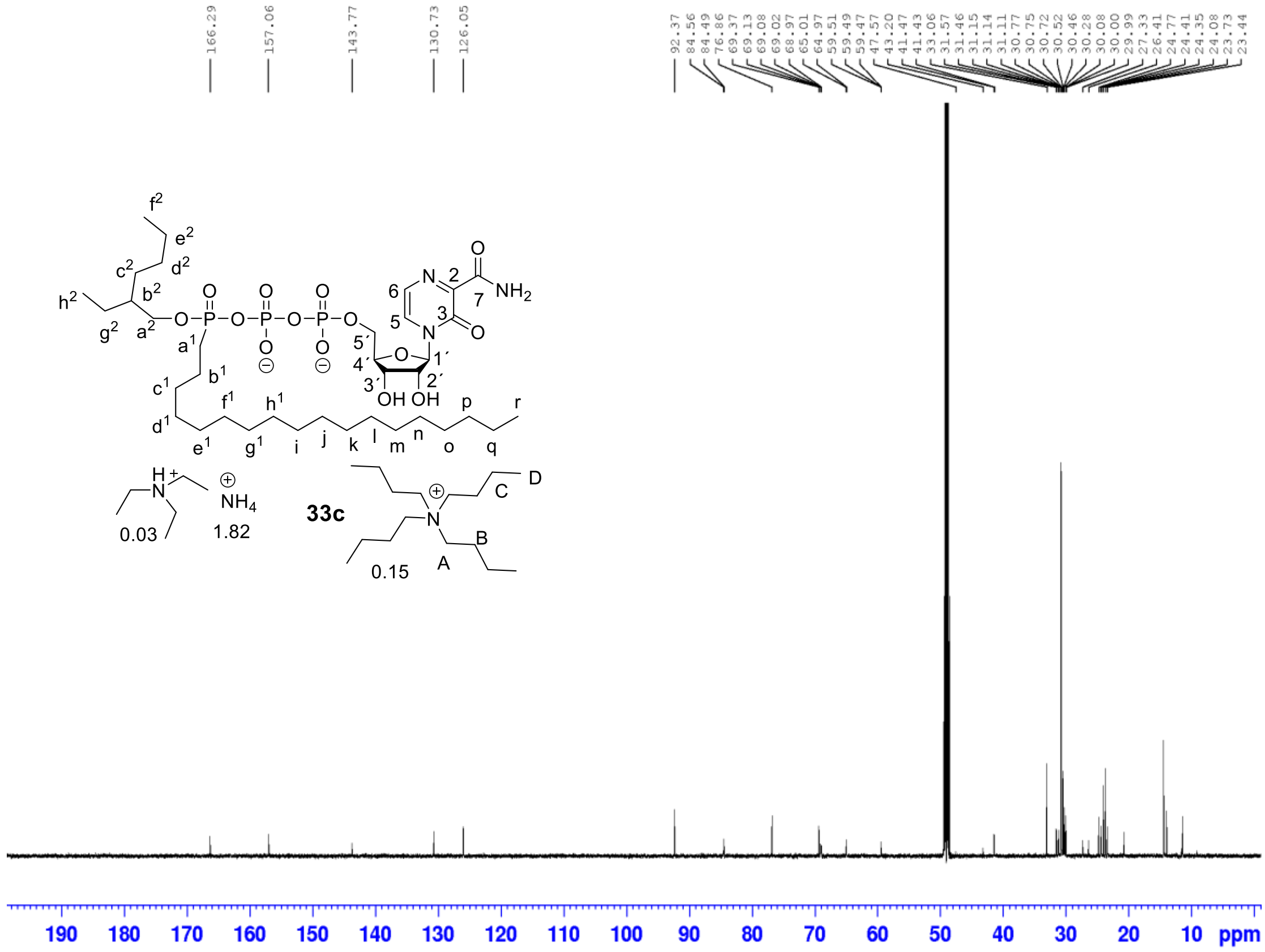
166.29
157.06
143.77
130.73
126.05
92.37
84.56
84.49
76.86
69.37
69.13
69.08
69.02
68.97
65.01
64.97
59.51
59.49
59.47
47.57
43.20
41.47
41.43
33.06
31.57
31.46
31.15
31.14
31.11
30.77
30.75
30.72
30.52
30.46
30.28
30.08
30.00
29.99
27.33
26.41
24.77
24.41
24.35
24.08
23.73
23.44
0.03
1.82
33c
0.15
190 180 170 160 150 140 130 120 110 100 90 80 70 60 50 40 30 20 10 ppm

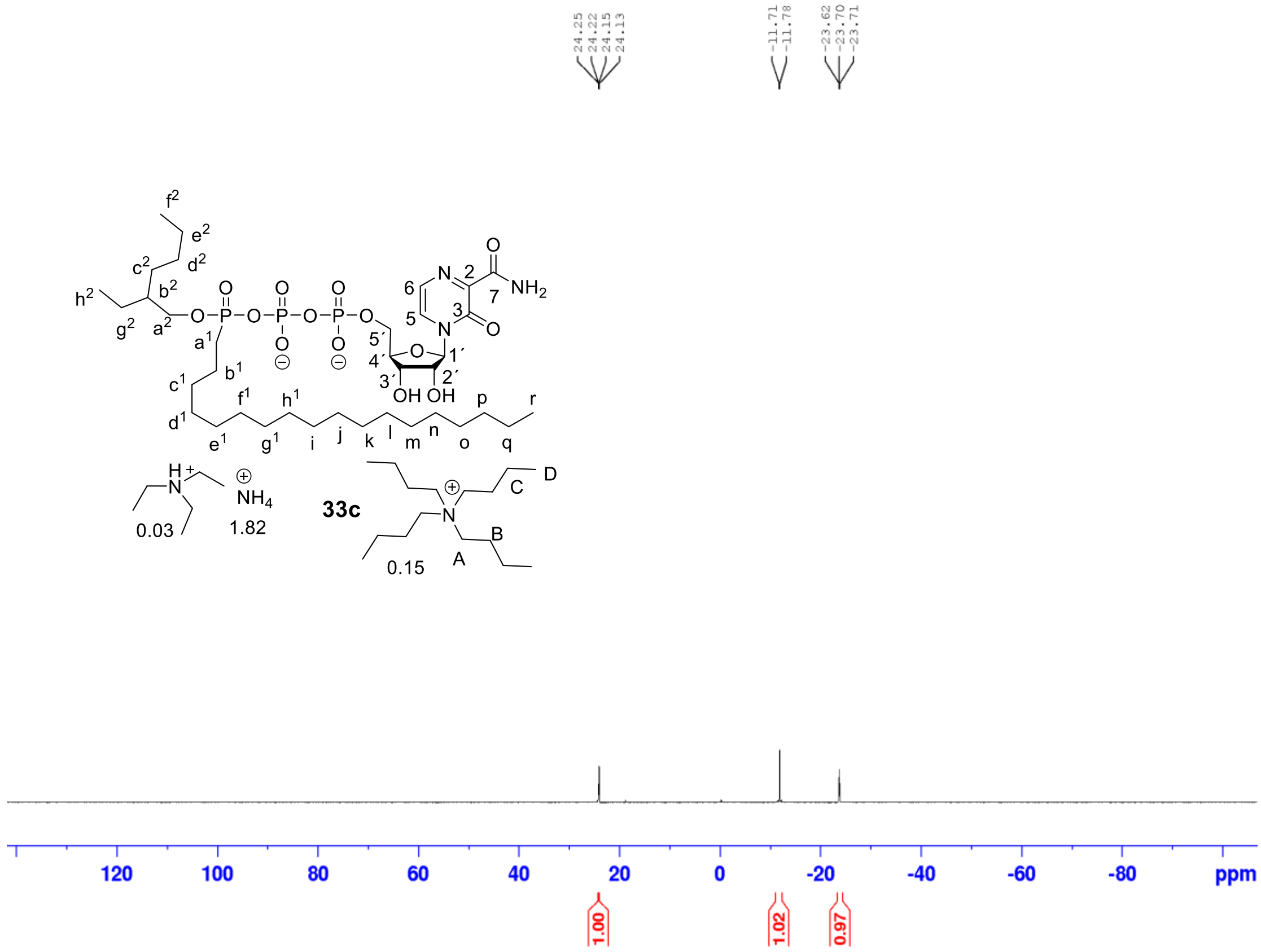
24.25
24.22
24.15
24.13
-11.71
-11.78
-23.62
-23.70
-23.71
0.03
1.82
33c
0.15
120 100 80 60 40 20 0 -20 -40 -60 -80 ppm
1.00
1.02
0.97

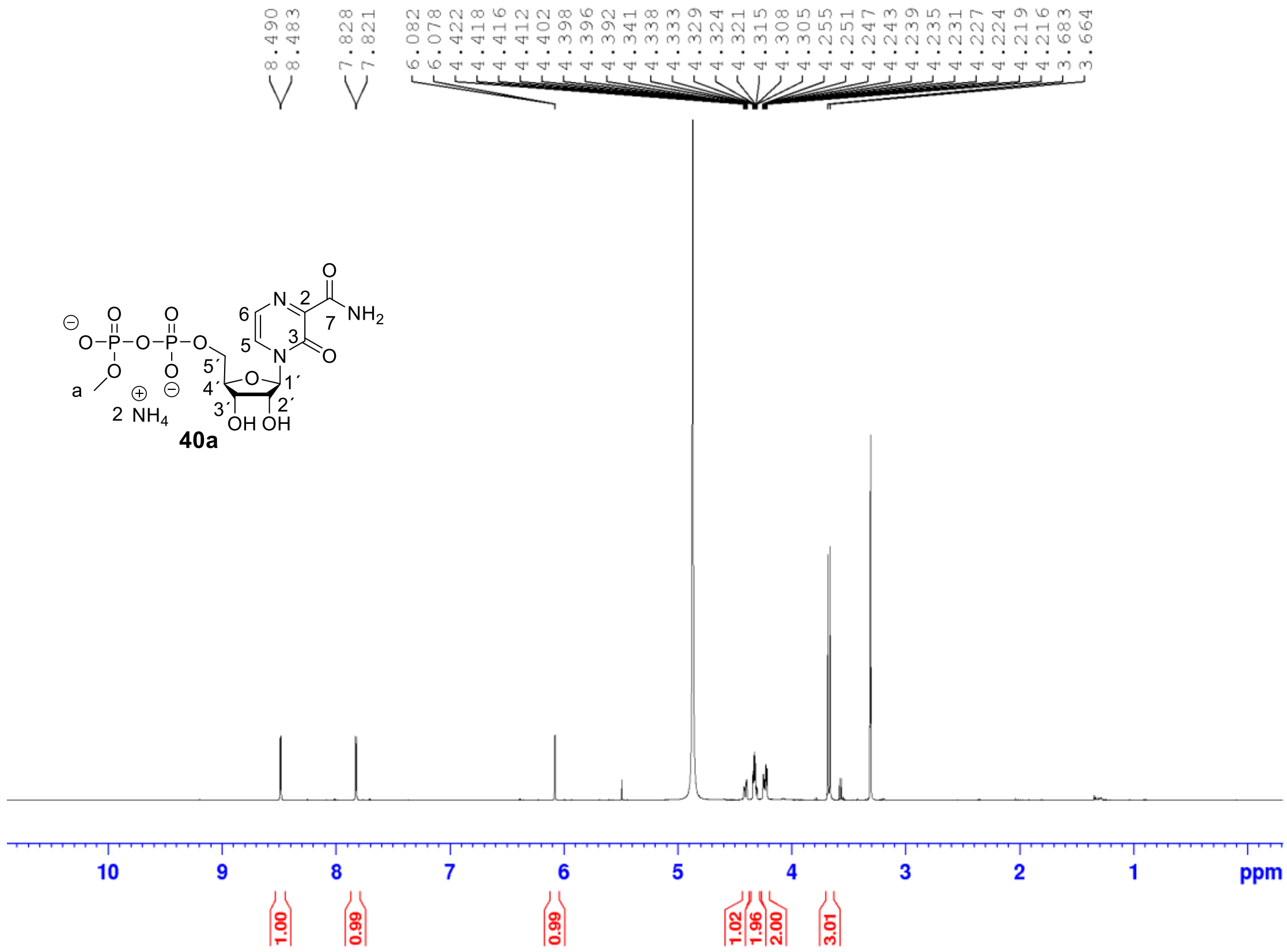
8.490
8.483
7.828
7.821
6.082
6.078
4.422
4.418
4.416
4.412
4.402
4.398
4.396
4.392
4.341
4.338
4.333
4.329
4.324
4.321
4.315
4.308
4.305
4.255
4.251
4.247
4.243
4.239
4.235
4.231
4.227
4.224
4.219
4.216
3.683
3.664
40a
ppm
1.00
0.99
0.99
1.02
1.96
2.00
3.01

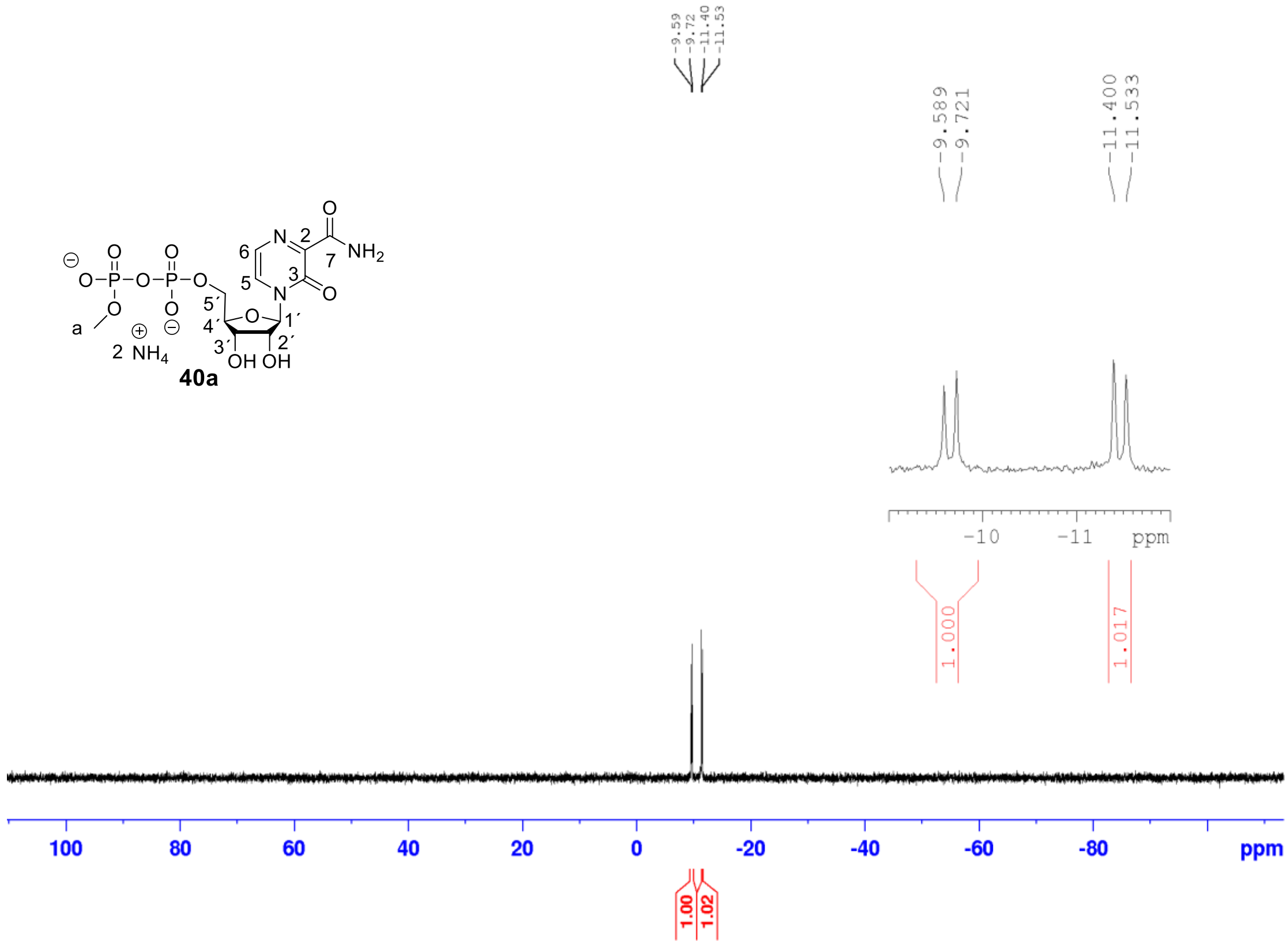
-9.59
-9.72
-11.40
-11.53
-9.589
-9.721
-11.400
-11.533
40a
-10
-11
ppm
1.000
1.017
ppm
1.00
1.02

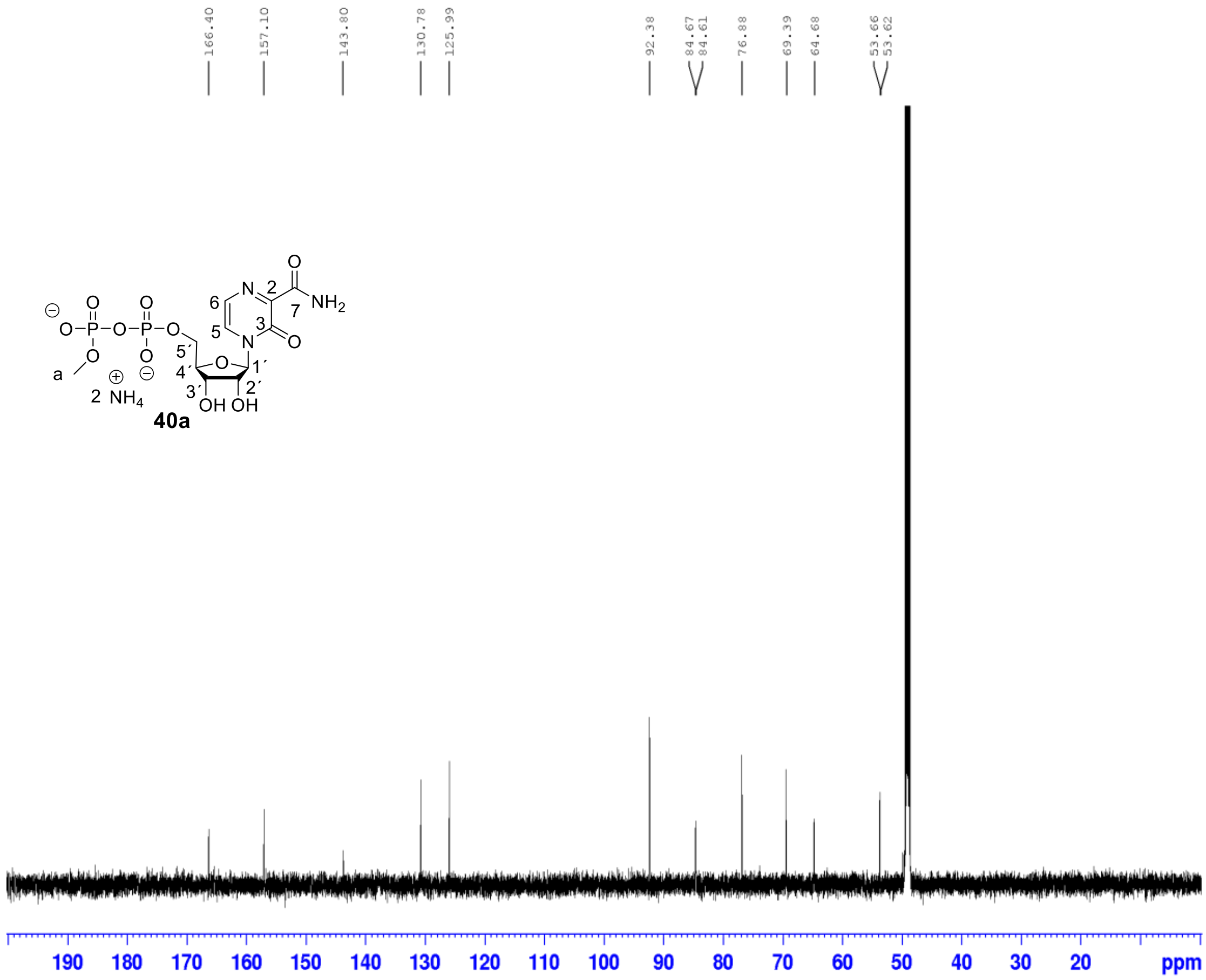
166.40
157.10
143.80
130.78
125.99
92.38
84.67
84.61
76.88
69.39
64.68
53.66
53.62
40a
190 180 170 160 150 140 130 120 110 100 90 80 70 60 50 40 30 20 ppm

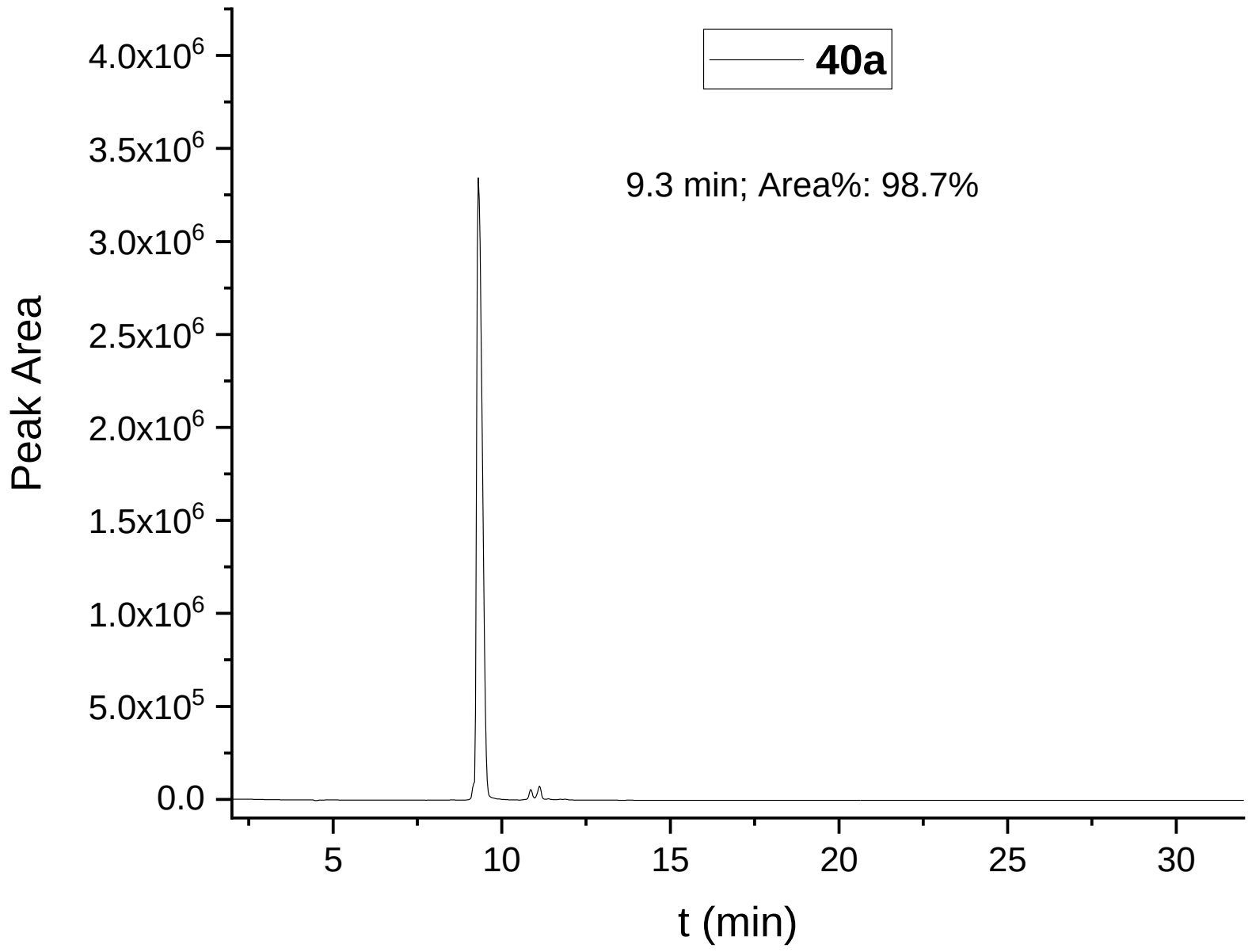
40a
9.3 min; Area%: 98.7%
Peak Area
t (min)

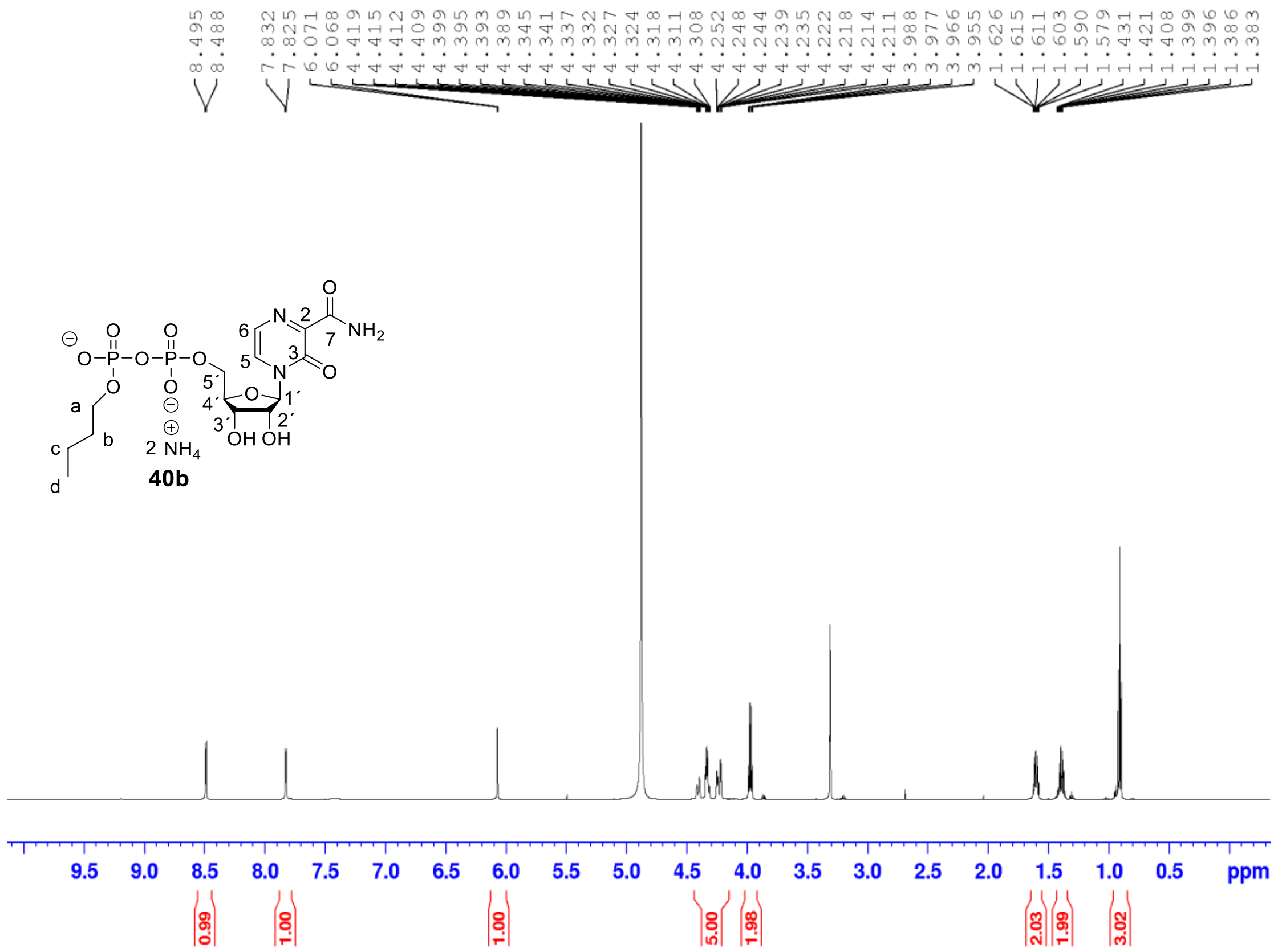
8.495
8.488
7.832
7.825
6.071
6.068
4.419
4.415
4.412
4.409
4.399
4.395
4.393
4.389
4.345
4.341
4.337
4.332
4.327
4.324
4.318
4.311
4.308
4.252
4.248
4.244
4.239
4.235
4.222
4.218
4.214
4.211
3.988
3.977
3.966
3.955
1.626
1.615
1.611
1.603
1.590
1.579
1.431
1.421
1.408
1.399
1.396
1.386
1.383
40b
2 NH4
9.5 9.0 8.5 8.0 7.5 7.0 6.5 6.0 5.5 5.0 4.5 4.0 3.5 3.0 2.5 2.0 1.5 1.0 0.5 ppm
0.99
1.00
1.00
5.00
1.98
2.03
1.99
3.02

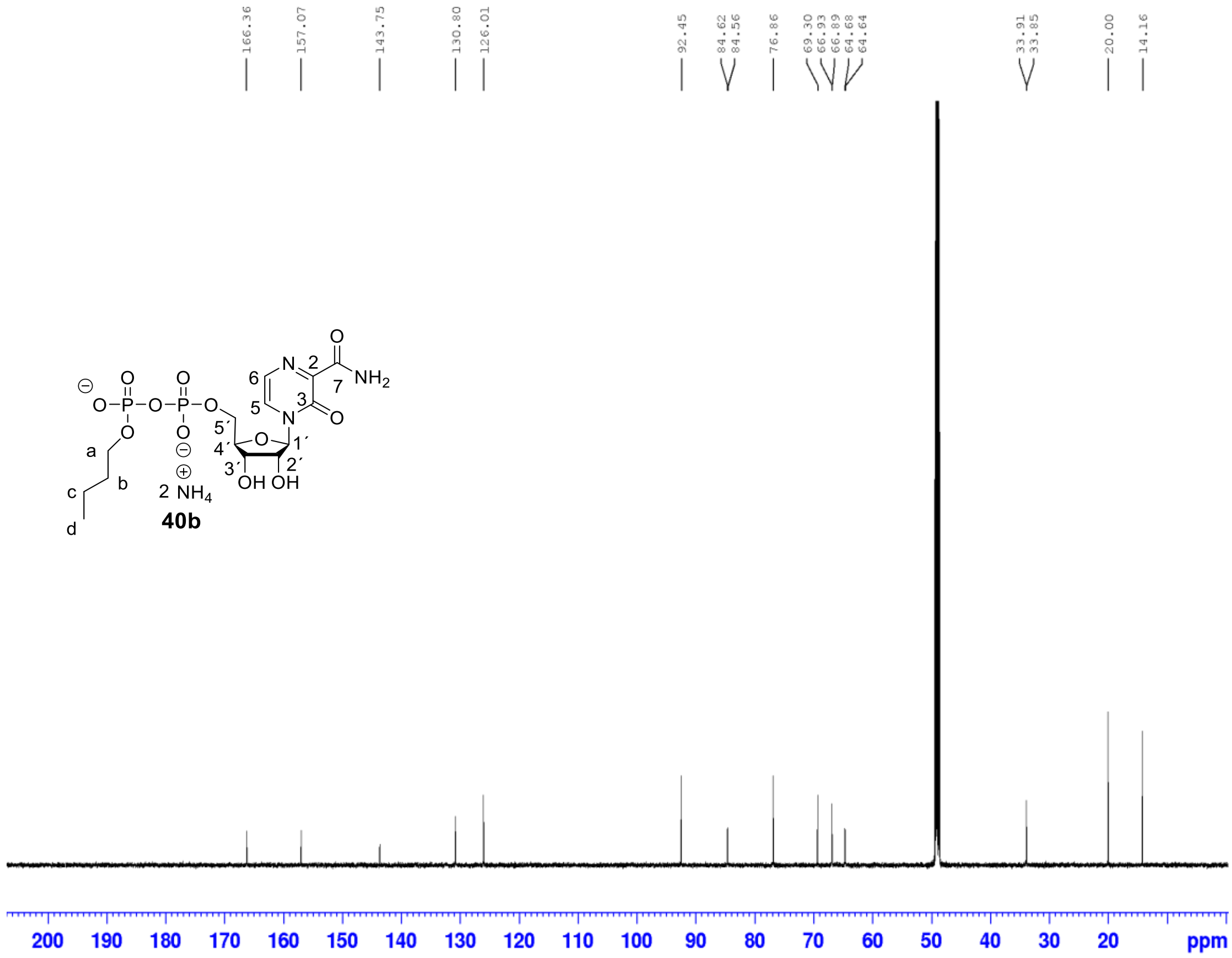
166.36
157.07
143.75
130.80
126.01
92.45
84.62
84.56
76.86
69.30
66.93
66.89
64.68
64.64
33.91
33.85
20.00
14.16
40b
2 NH4
200 190 180 170 160 150 140 130 120 110 100 90 80 70 60 50 40 30 20 ppm

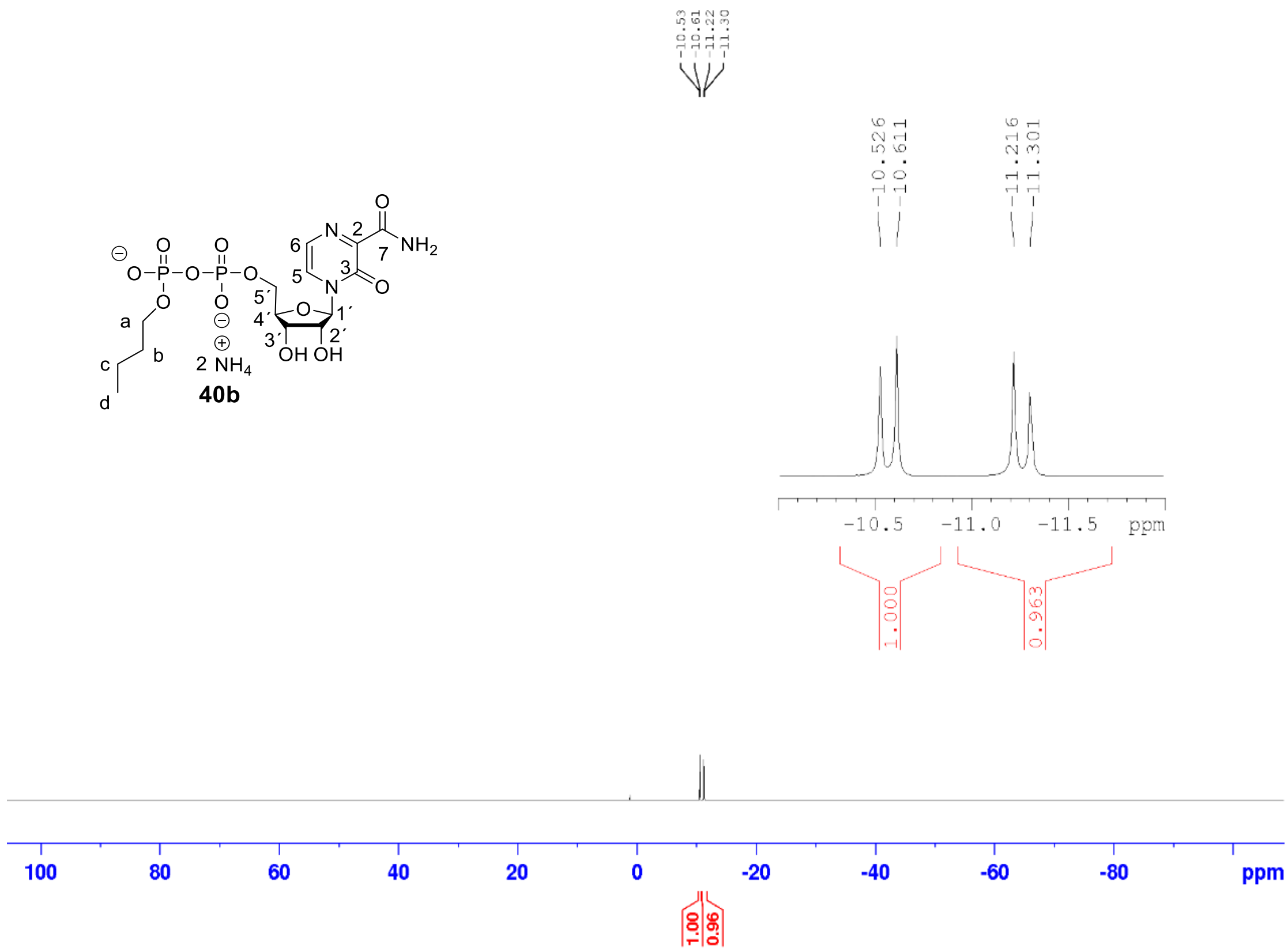
40b
ppm

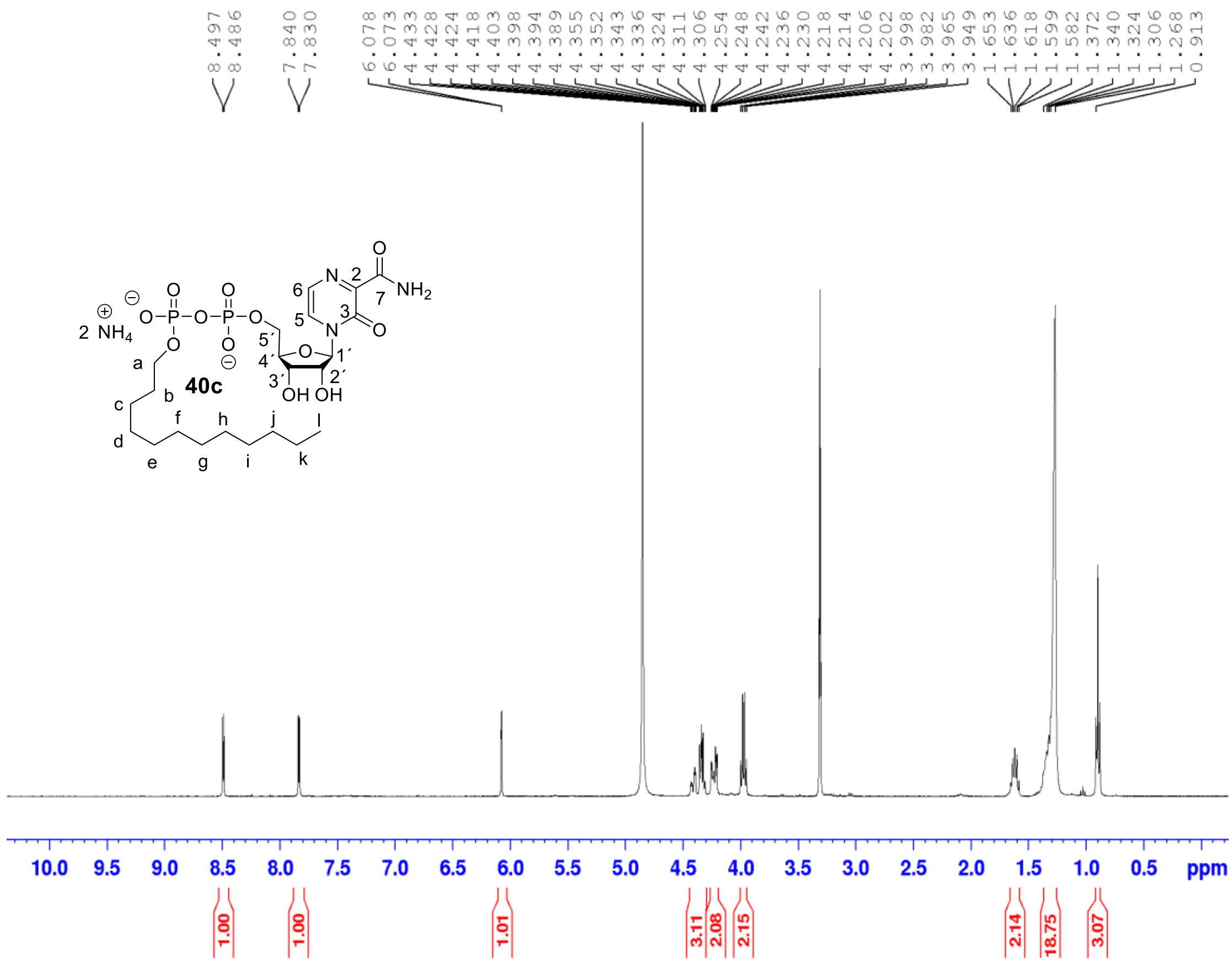
40c
ppm

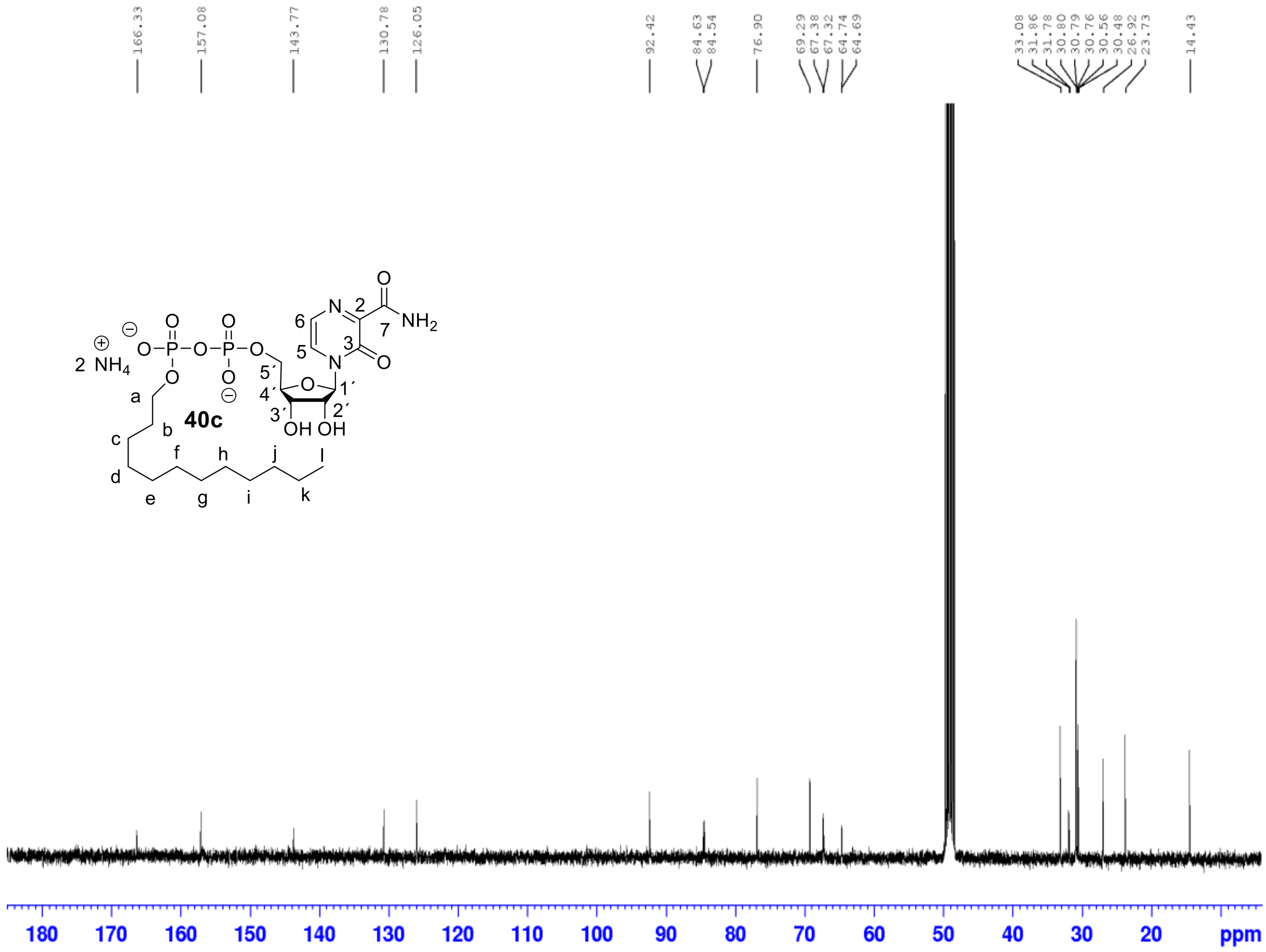
166.33
157.08
143.77
130.78
126.05
92.42
84.63
84.54
76.90
69.29
67.38
67.32
64.74
64.69
33.08
31.86
31.78
30.80
30.79
30.76
30.56
30.48
26.92
23.73
14.43
2 NH4
40c
OH OH
180 170 160 150 140 130 120 110 100 90 80 70 60 50 40 30 20 ppm

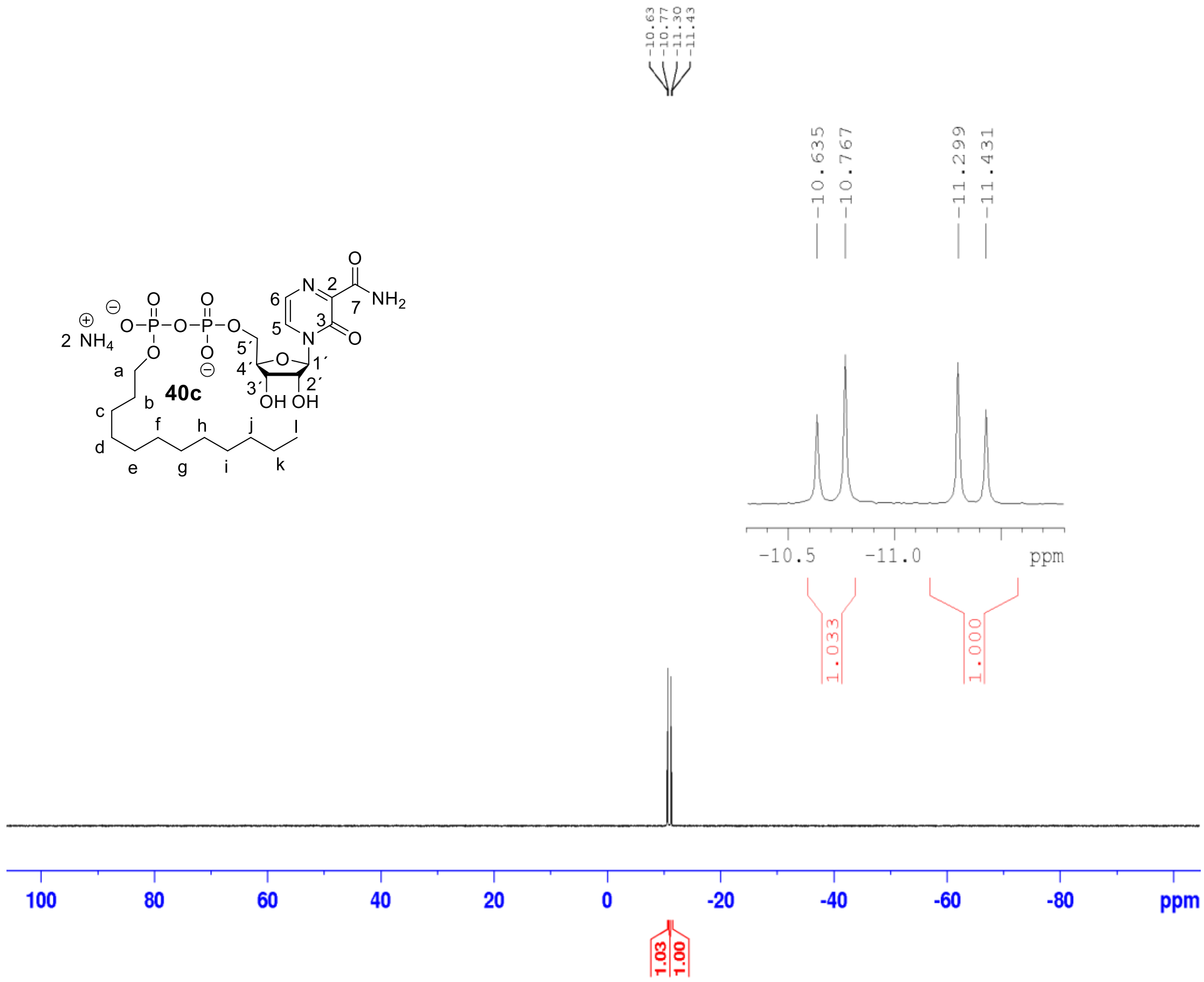
-10.63
-10.77
-11.30
-11.43
-10.635
-10.767
-11.299
-11.431
-10.5 -11.0 ppm
1.033
1.000
2 NH4
40c
OH OH
100 80 60 40 20 0 -20 -40 -60 -80 ppm
1.03
1.00

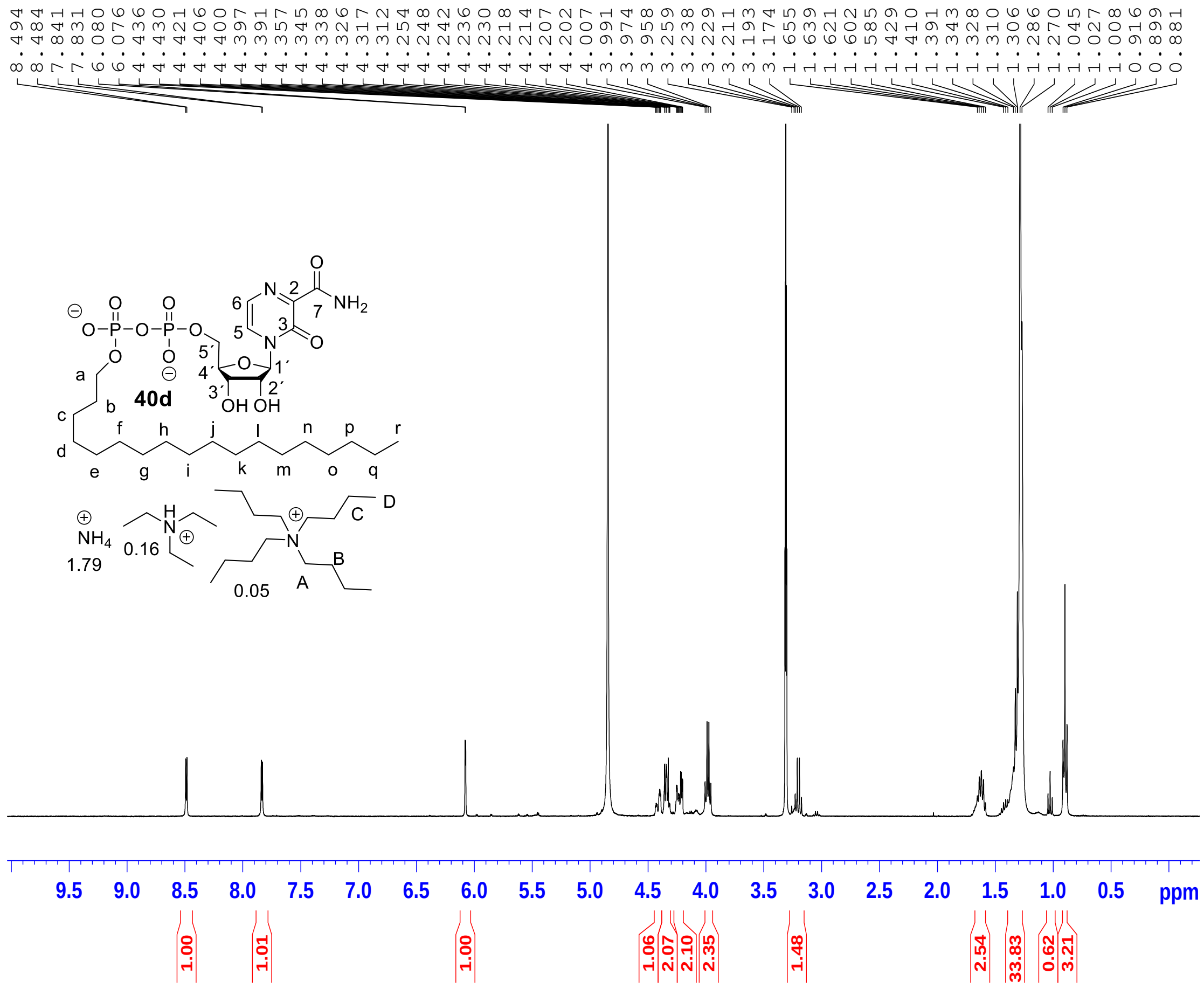

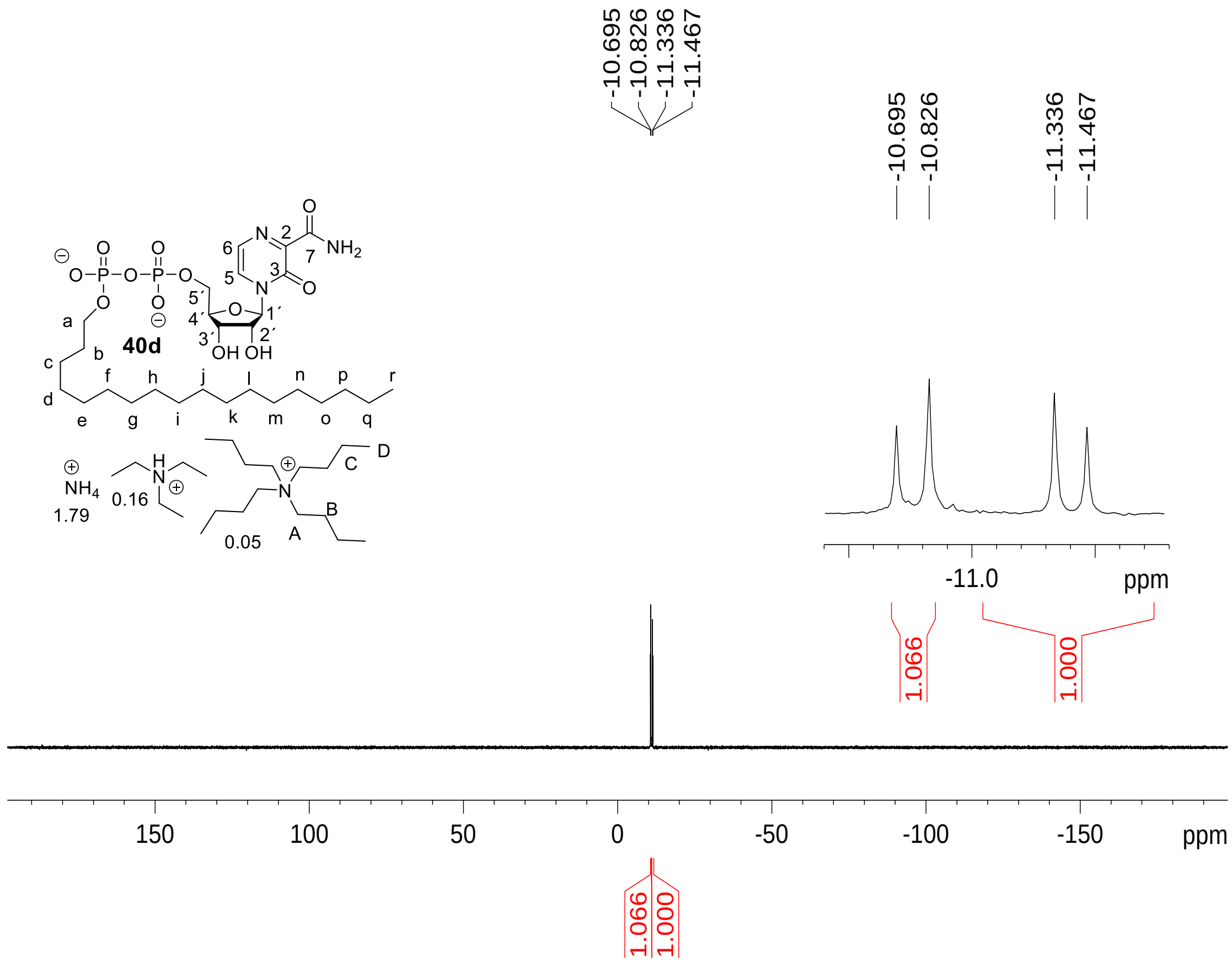

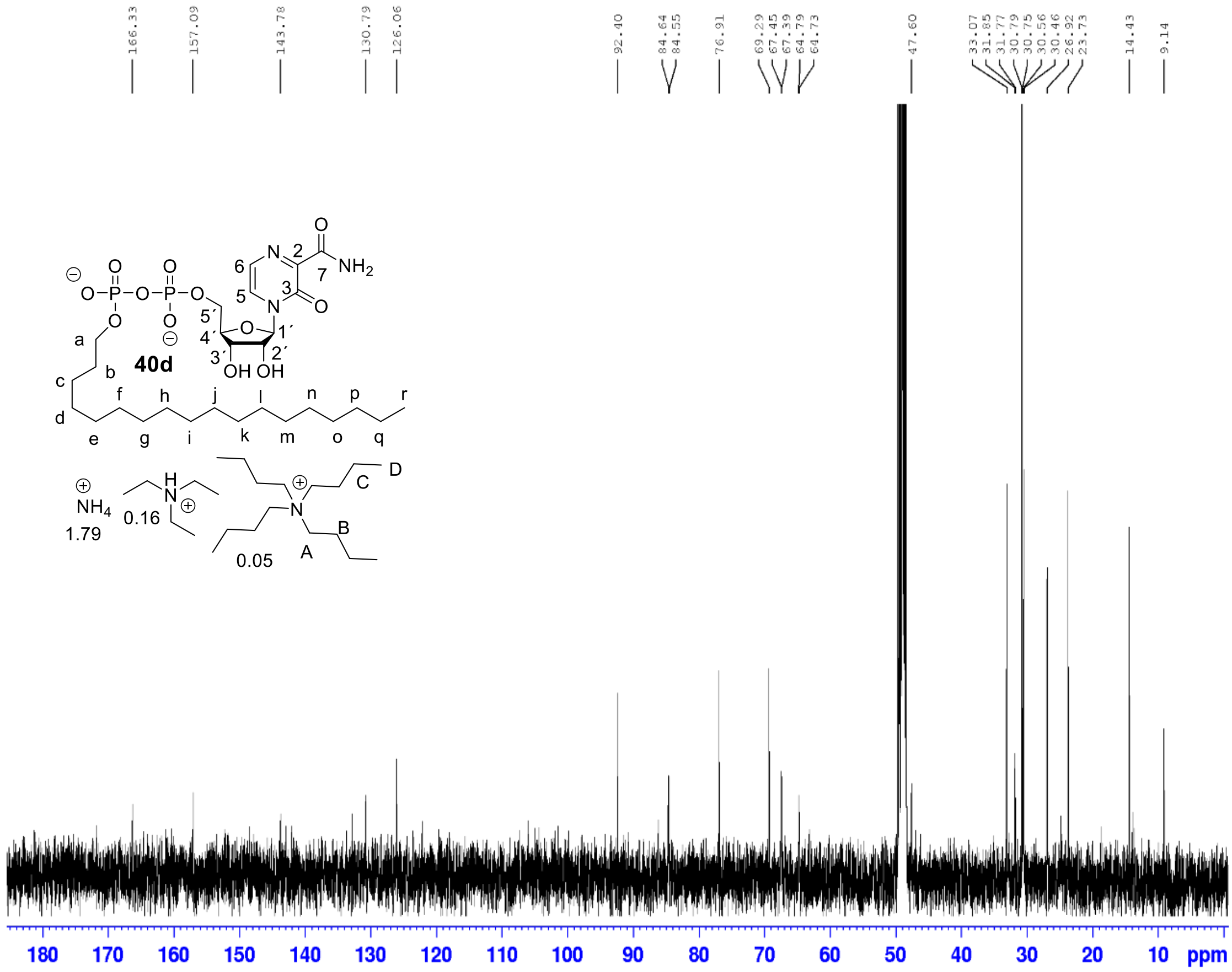
166.33
157.09
143.78
130.79
126.06
92.40
84.64
84.55
76.91
69.29
67.45
67.39
64.79
64.73
47.60
33.07
31.85
31.77
30.79
30.75
30.56
30.46
26.92
23.73
14.43
9.14
40d
NH4
1.79
0.16
0.05
180
170
160
150
140
130
120
110
100
90
80
70
60
50
40
30
20
10
ppm

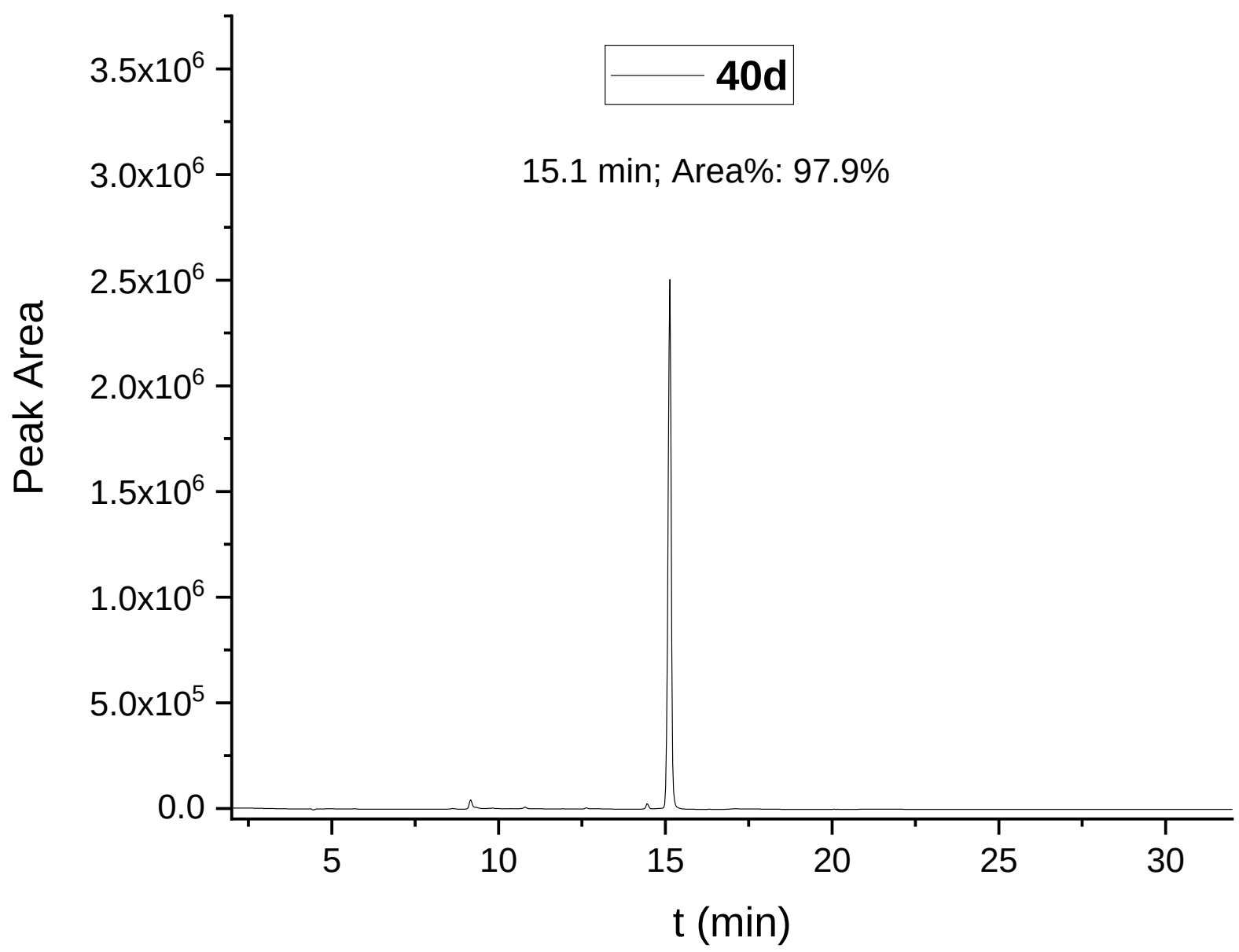
40d
15.1 min; Area%: 97.9%
Peak Area
t (min)

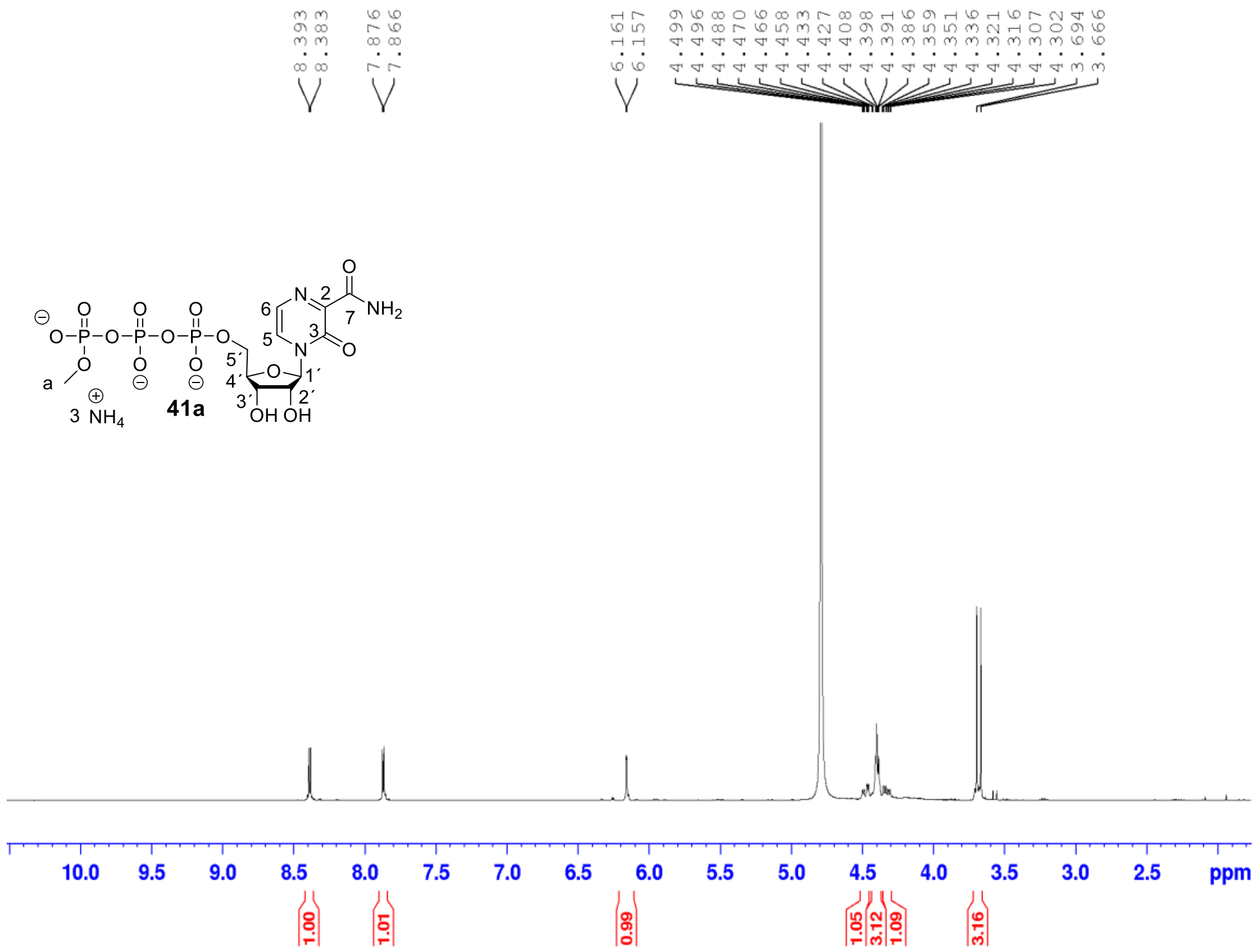

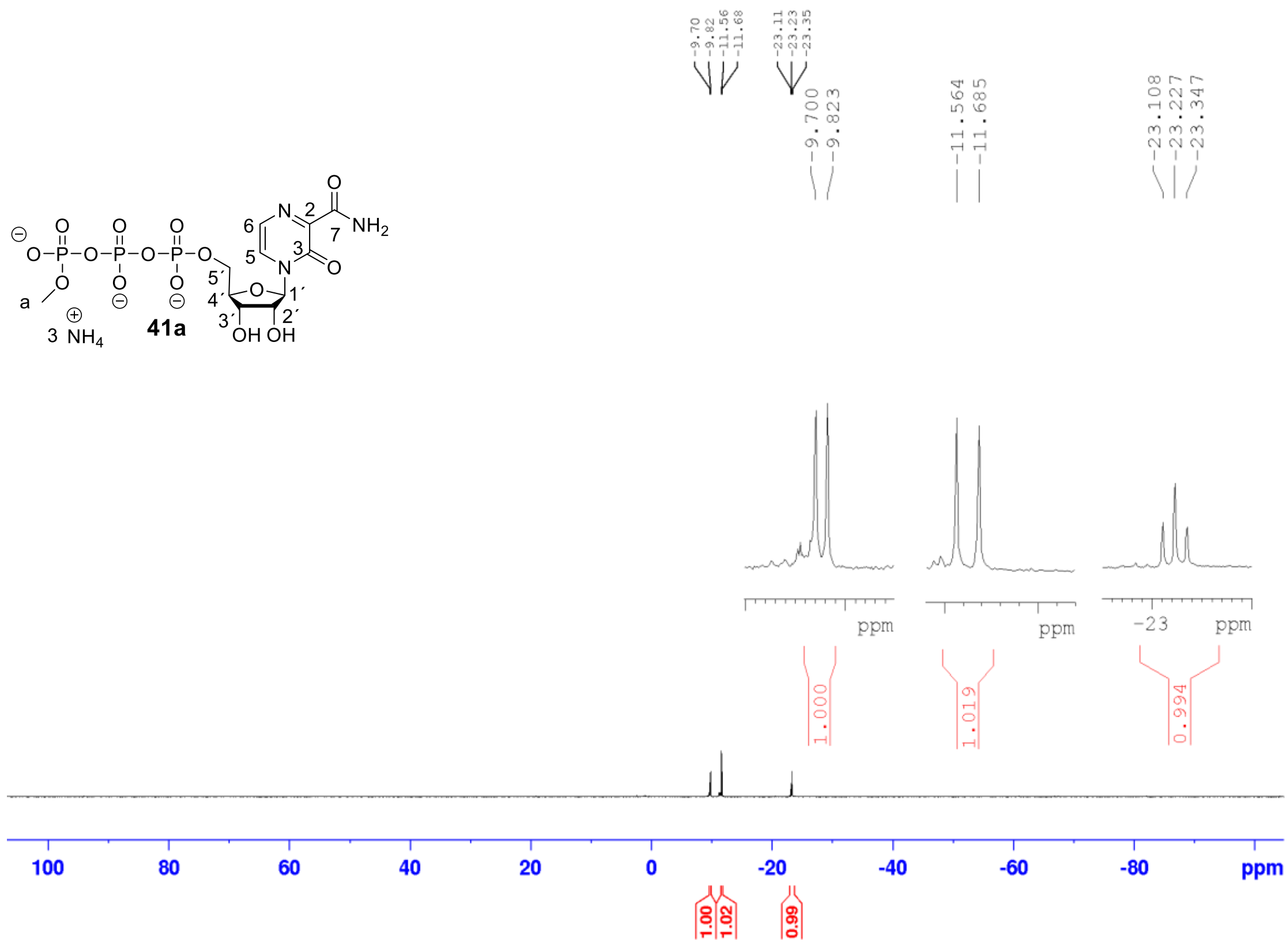

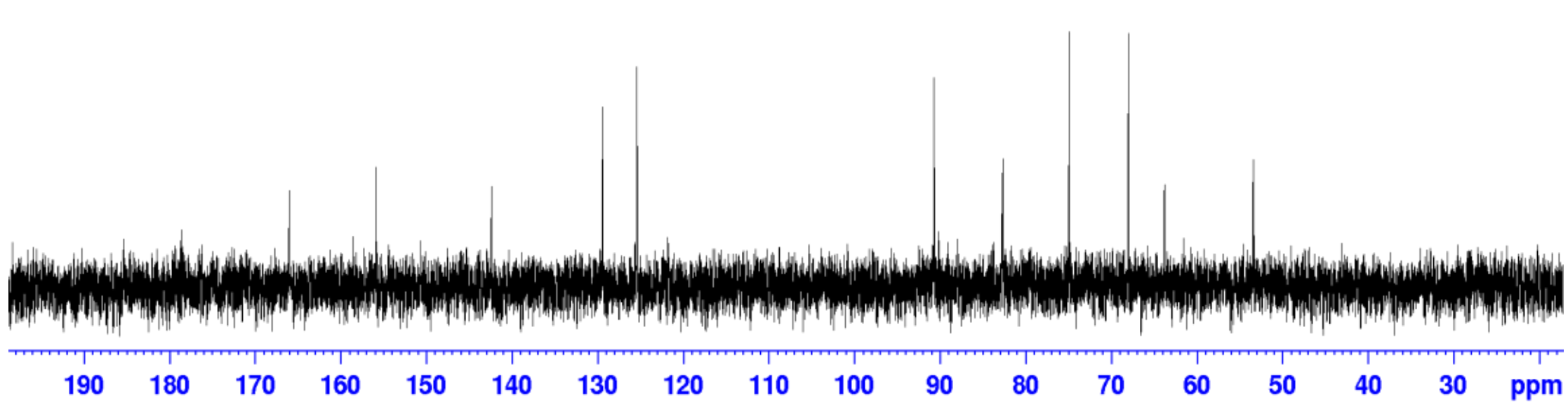
166.03
155.90
142.42
129.41
125.40
90.71
82.75
82.66
74.91
68.00
63.74
53.44
53.38
190
180
170
160
150
140
130
120
110
100
90
80
70
60
50
40
30
ppm

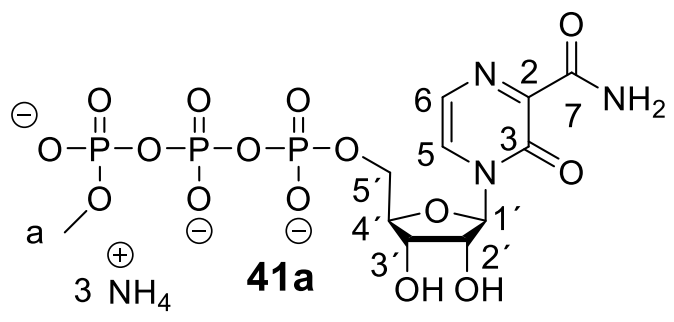
41a
3 NH4

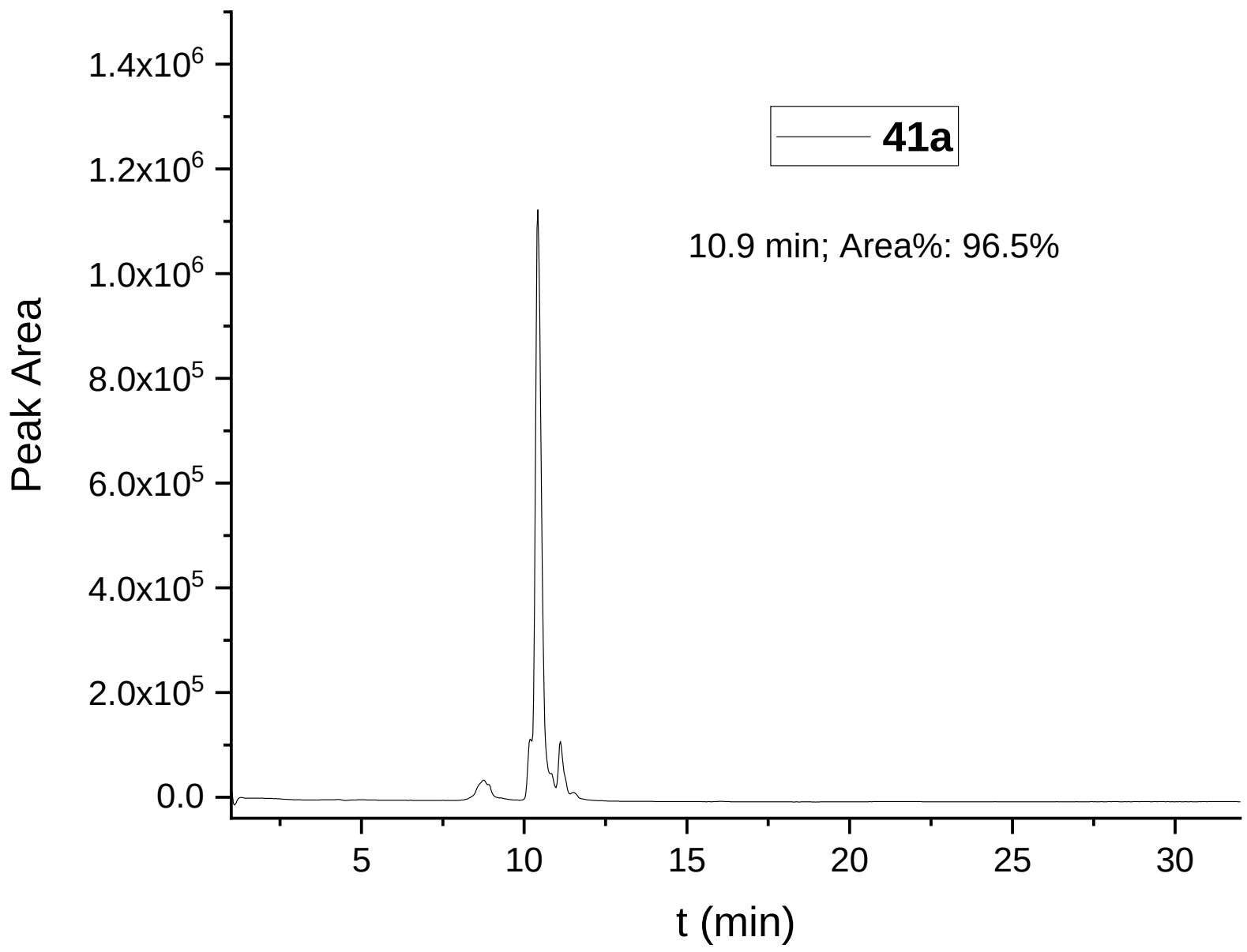
41a
10.9 min; Area%: 96.5%
Peak Area
t (min)

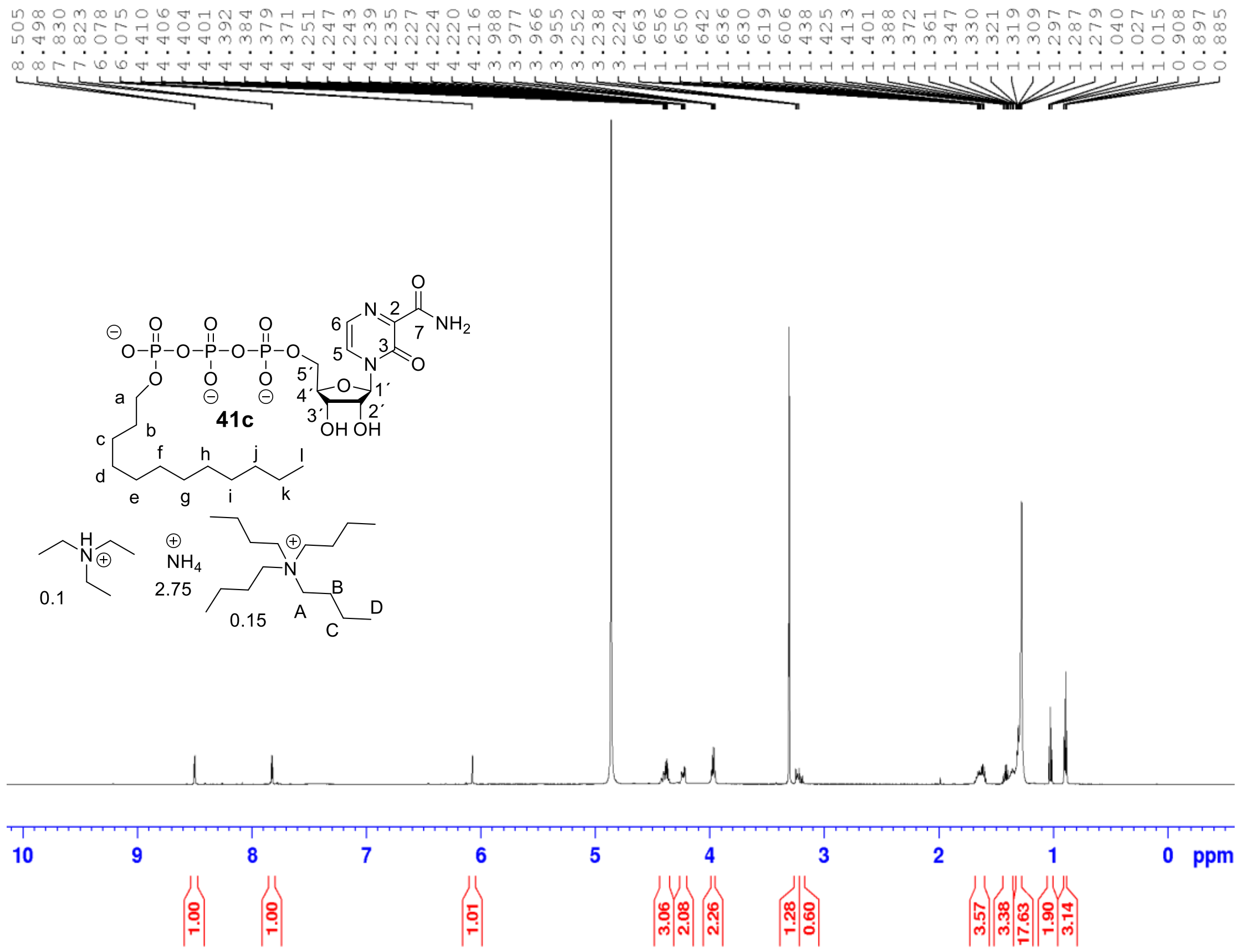
8.505
8.498
7.830
7.823
6.078
6.075
4.410
4.406
4.404
4.401
4.392
4.384
4.379
4.371
4.251
4.247
4.243
4.239
4.235
4.227
4.224
4.220
4.216
3.988
3.977
3.966
3.955
3.252
3.238
3.224
1.663
1.656
1.650
1.642
1.636
1.630
1.619
1.606
1.438
1.425
1.413
1.401
1.388
1.372
1.361
1.347
1.330
1.321
1.319
1.309
1.297
1.287
1.279
1.040
1.027
1.015
0.908
0.897
0.885
41c
0.1
2.75
0.15
ppm
1.00
1.00
1.01
3.06
2.08
2.26
1.28
0.60
3.57
3.38
17.63
1.90
3.14

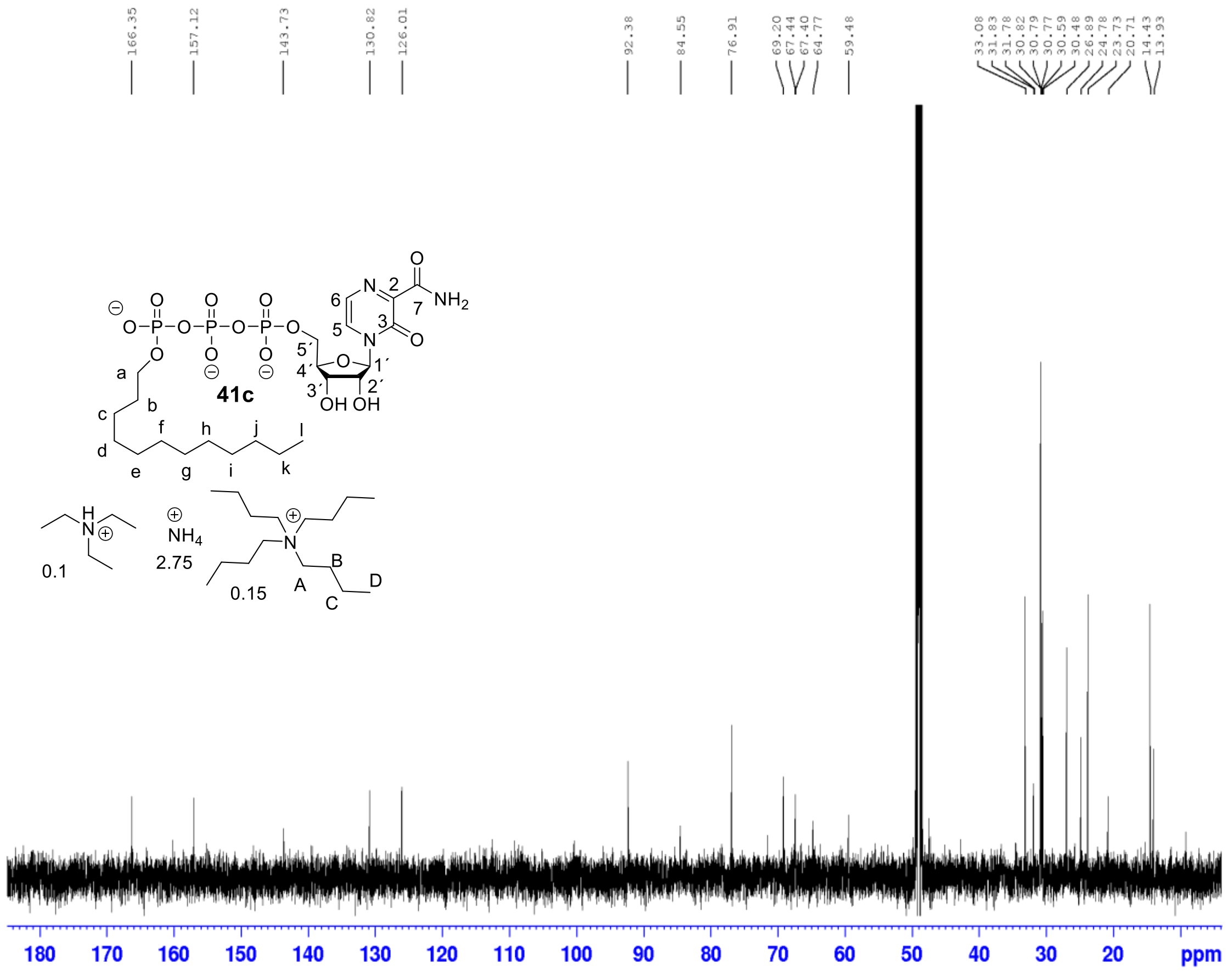
166.35
157.12
143.73
130.82
126.01
92.38
84.55
76.91
69.20
67.44
67.40
64.77
59.48
33.08
31.83
31.78
30.82
30.79
30.77
30.59
30.48
26.89
24.78
23.73
20.71
14.43
13.93
41c
0.1
2.75
0.15
ppm

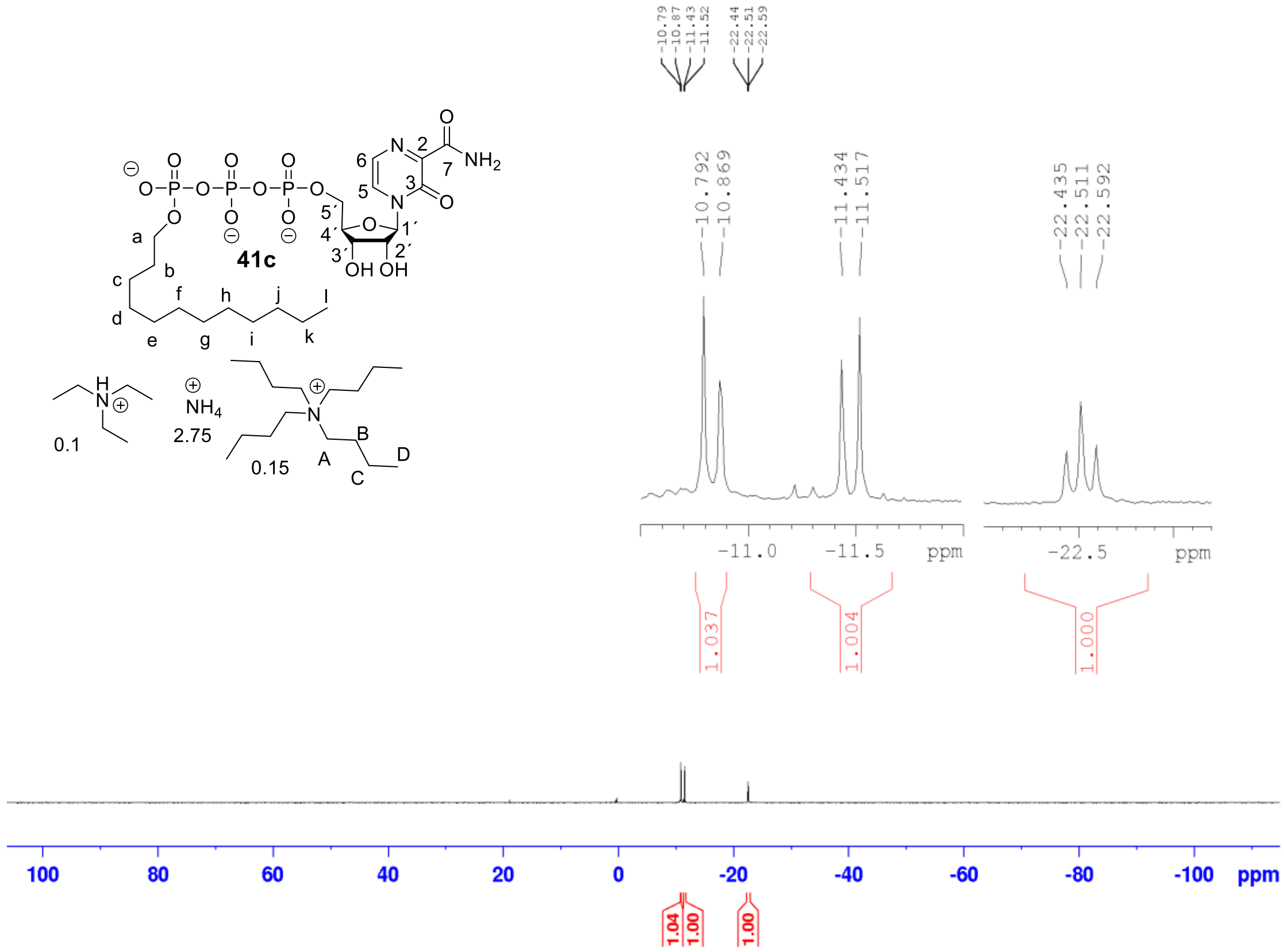
41c
0.1
2.75
0.15
-10.792
-10.869
-11.434
-11.517
-22.435
-22.511
-22.592
ppm
1.037
1.004
1.000
1.04
1.00
1.00

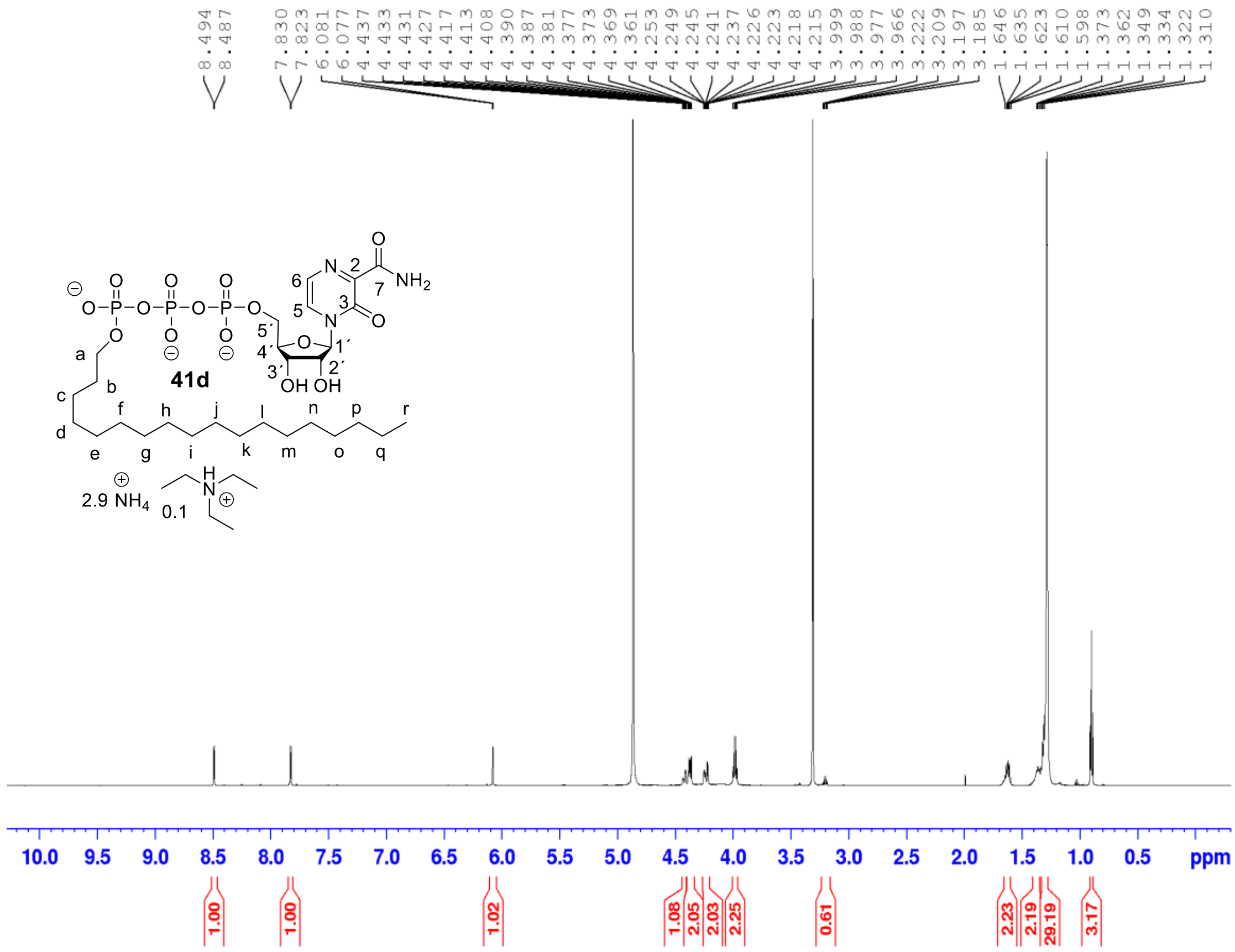
41d
2.9 NH4
0.1
ppm
1.00
1.00
1.02
1.08
2.05
2.03
2.25
0.61
2.23
2.19
29.19
3.17

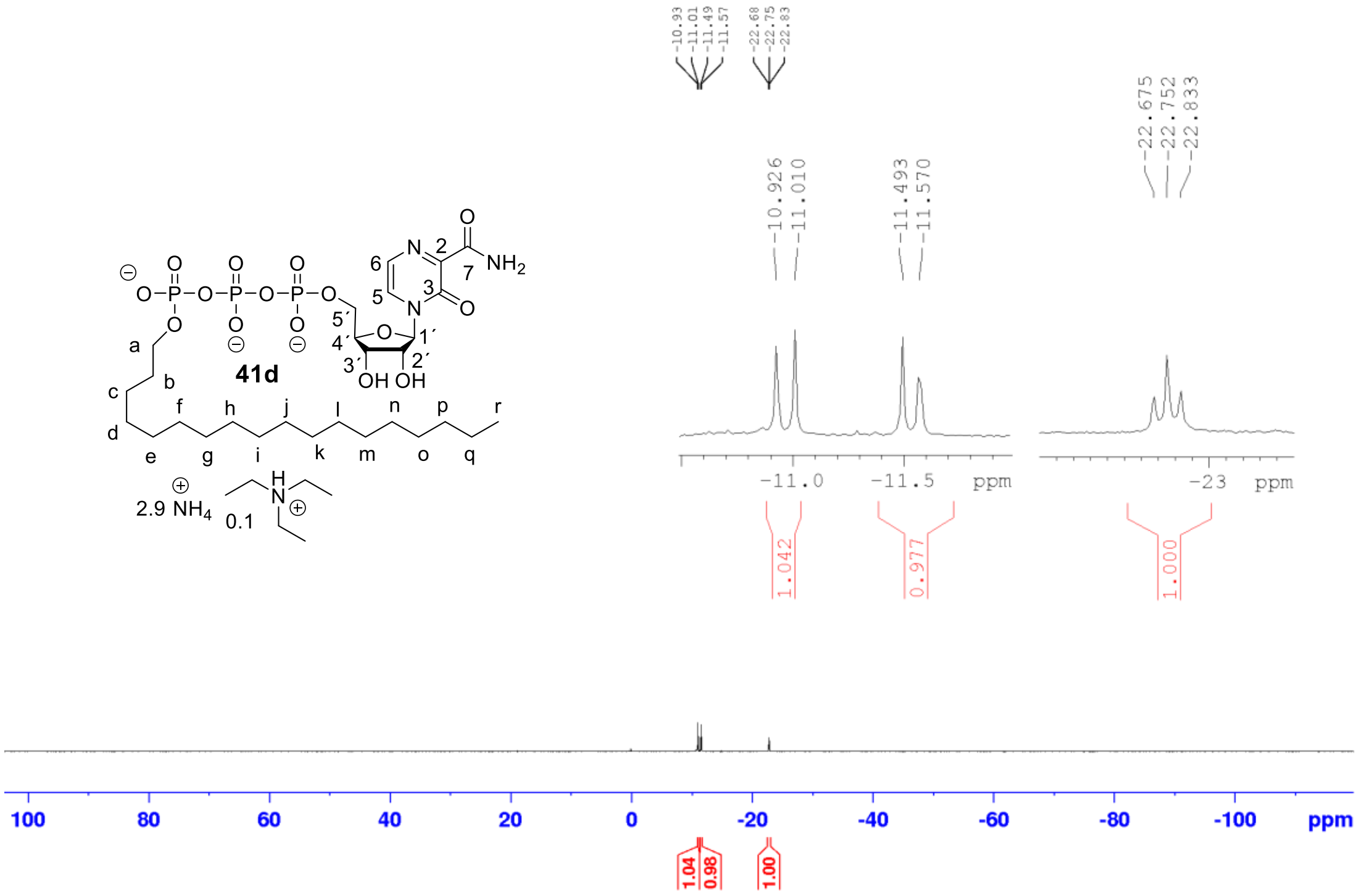
41d
2.9 NH4
0.1
-10.926
-11.010
-11.493
-11.570
-22.675
-22.752
-22.833
-11.0
-11.5
-23
ppm
1.042
0.977
1.000
100 80 60 40 20 0 -20 -40 -60 -80 -100 ppm
1.04
0.98
1.00

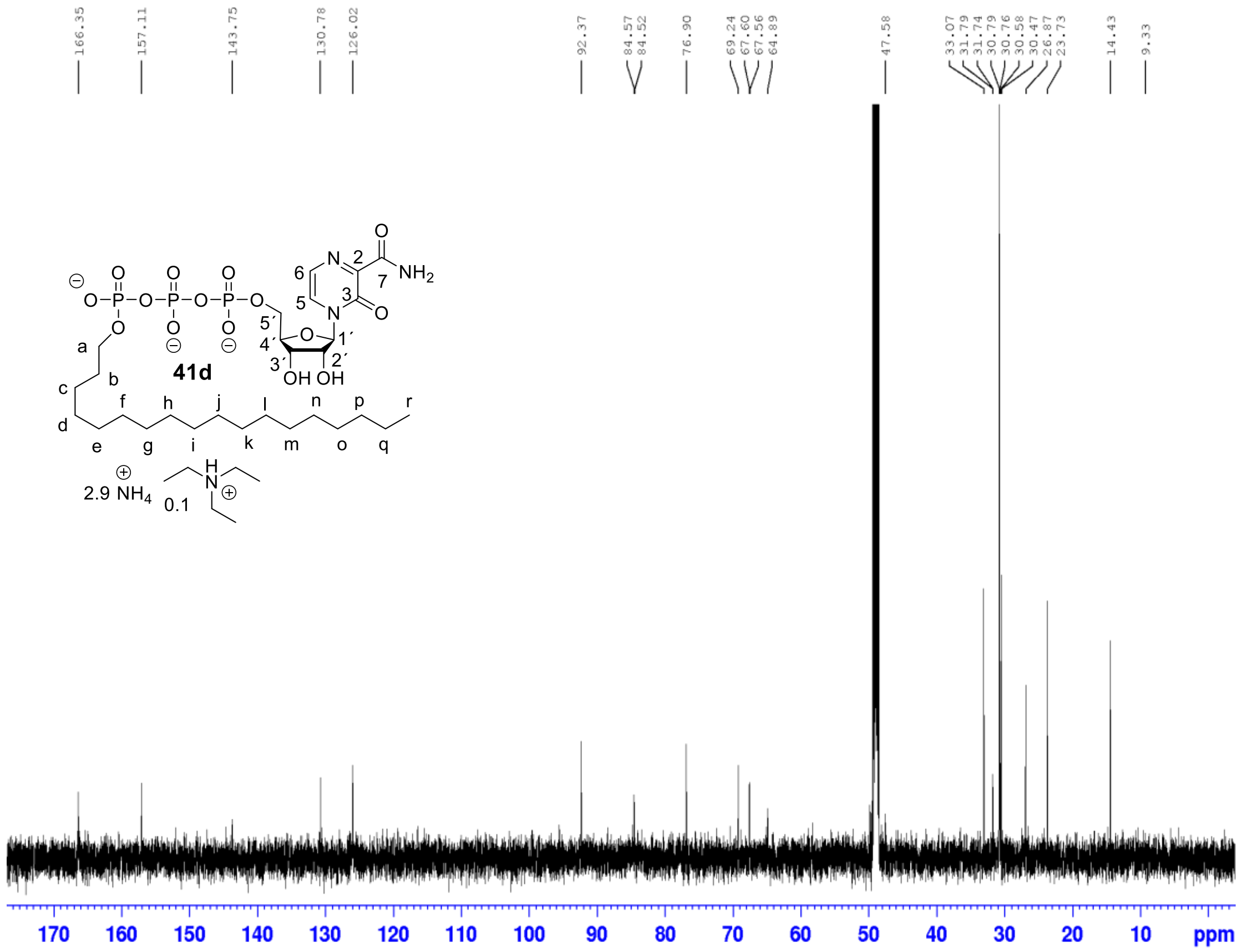
166.35
157.11
143.75
130.78
126.02
92.37
84.57
84.52
76.90
69.24
67.60
67.56
64.89
47.58
33.07
31.79
31.74
30.79
30.76
30.58
30.47
26.87
23.73
14.43
9.33
41d
2.9 NH4
0.1
170 160 150 140 130 120 110 100 90 80 70 60 50 40 30 20 10 ppm

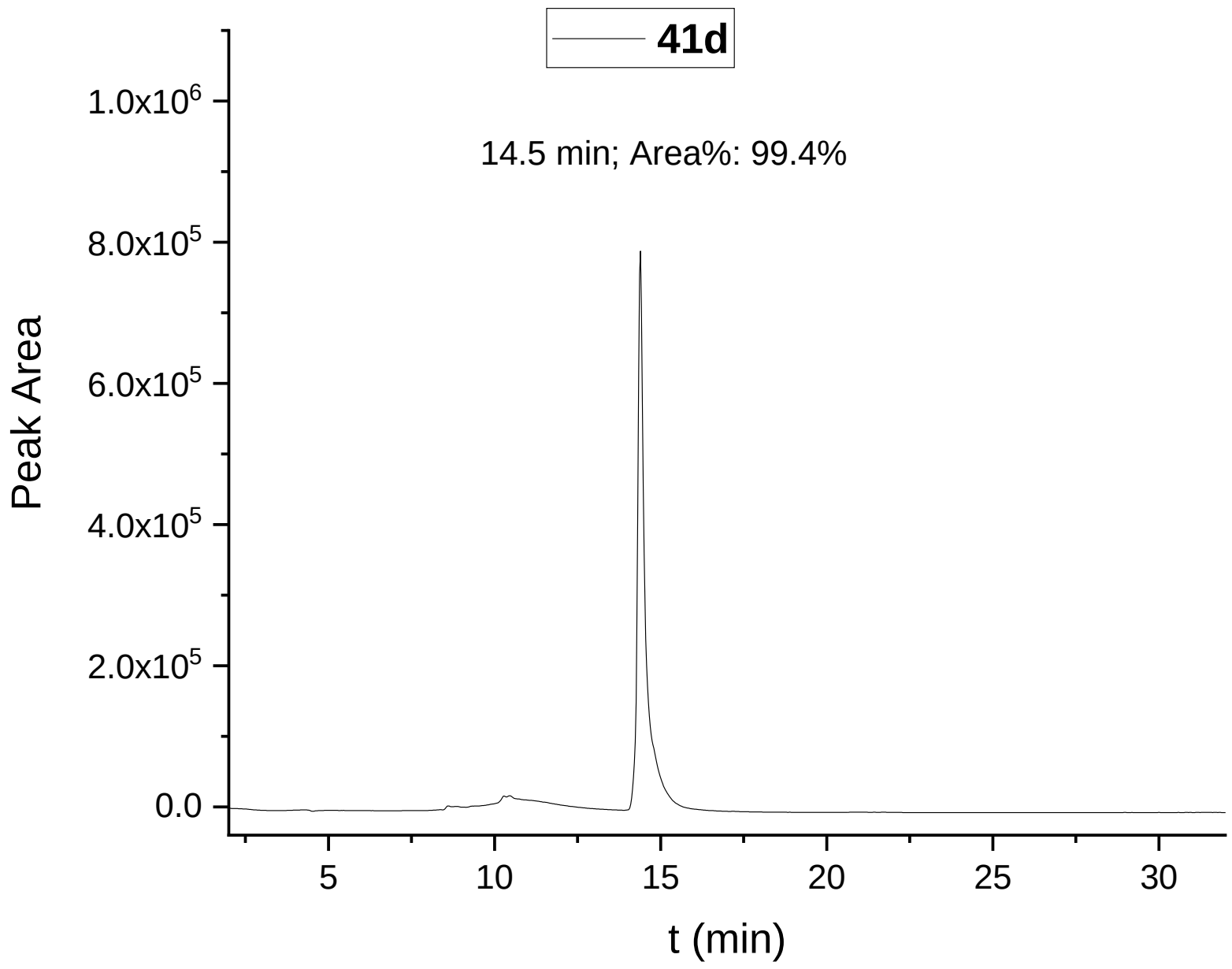
41d
14.5 min; Area%: 99.4%
Peak Area
t (min)

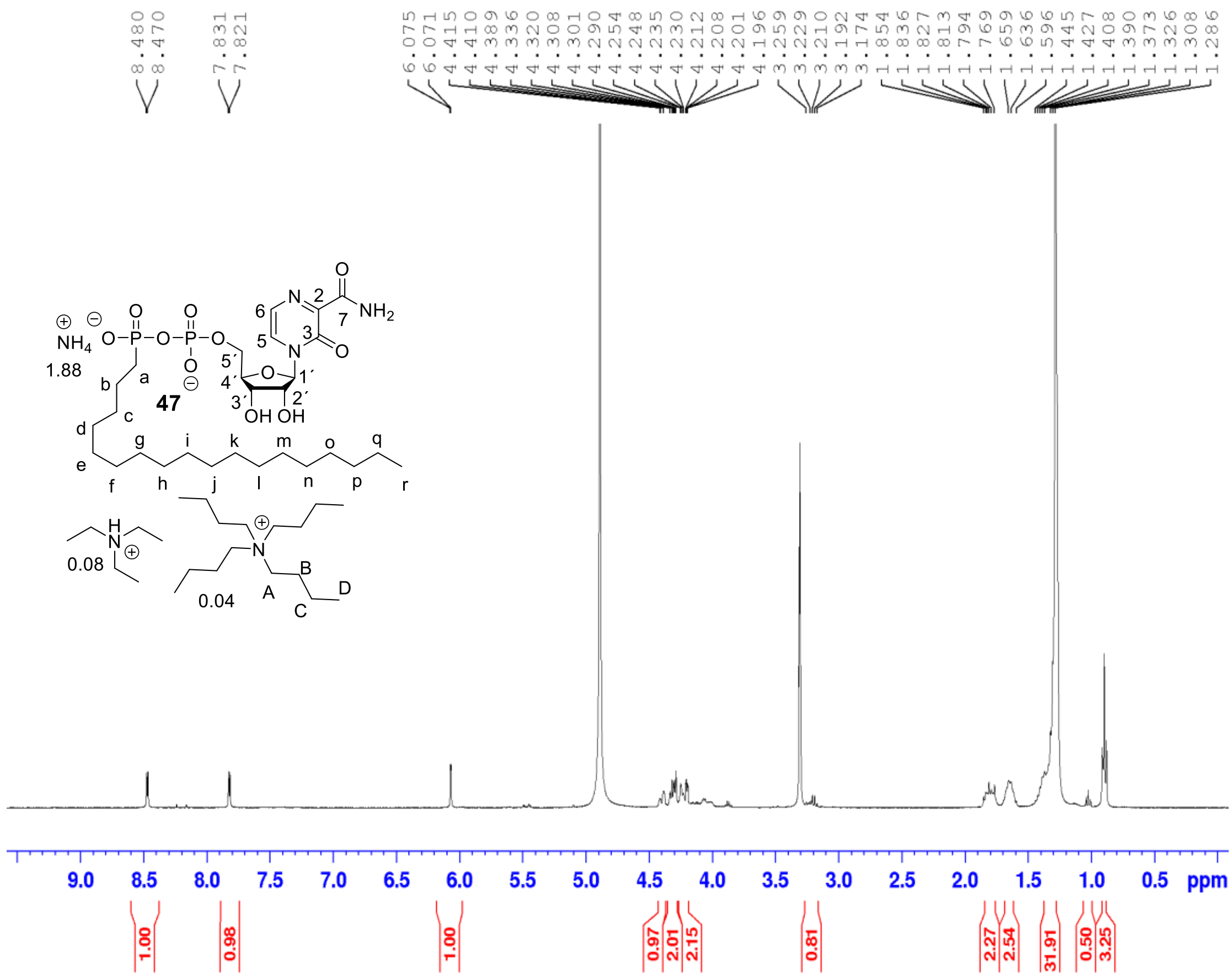
NH4
1.88
47
0.08
0.04
ppm

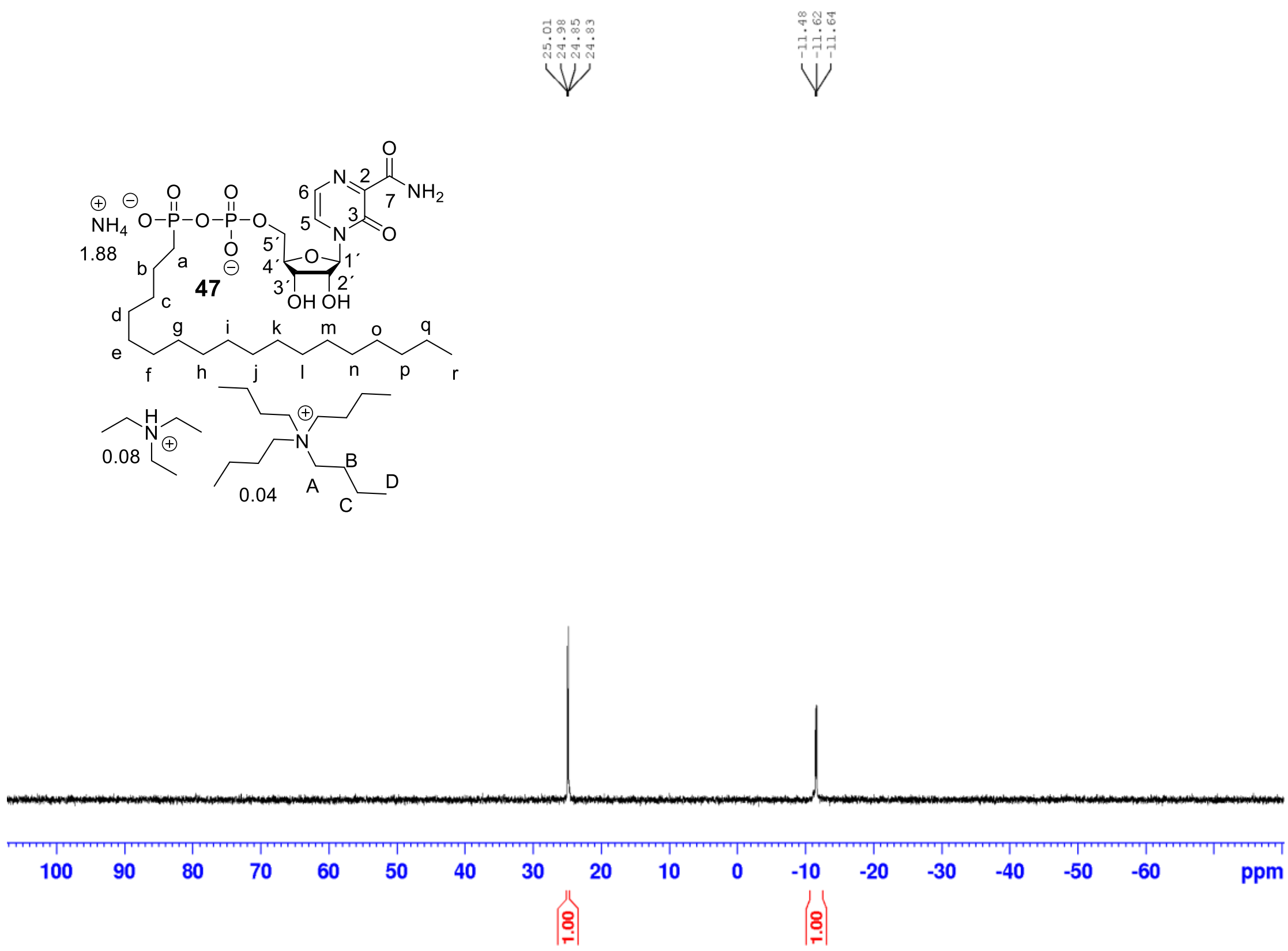
25.01
24.98
24.85
24.83
-11.48
-11.62
-11.64
47
1.88
0.08
0.04
ppm
1.00
1.00

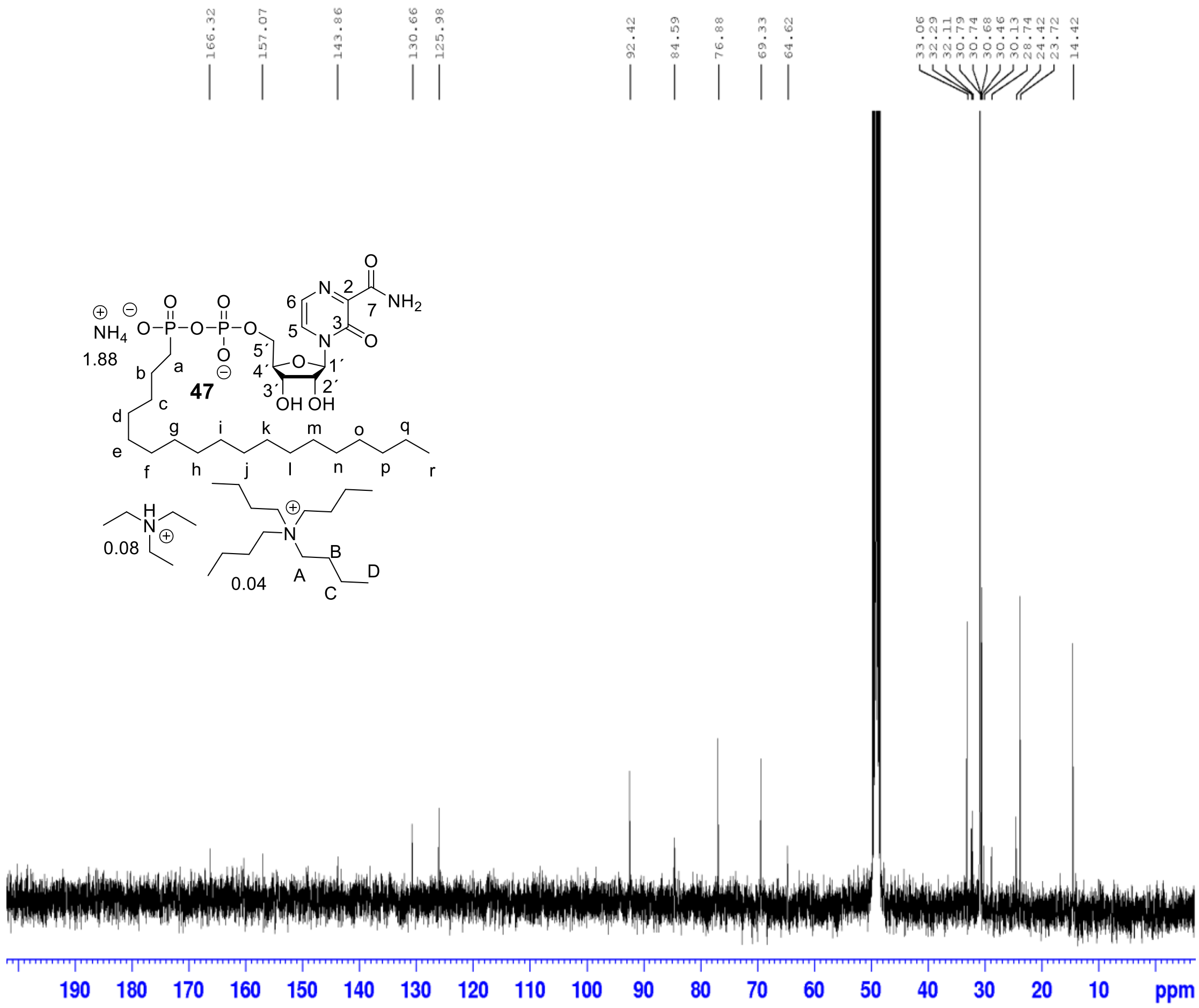
166.32
157.07
143.86
130.66
125.98
92.42
84.59
76.88
69.33
64.62
33.06
32.29
32.11
30.79
30.74
30.68
30.46
30.13
28.74
24.42
23.72
14.42
47
1.88
0.08
0.04
ppm

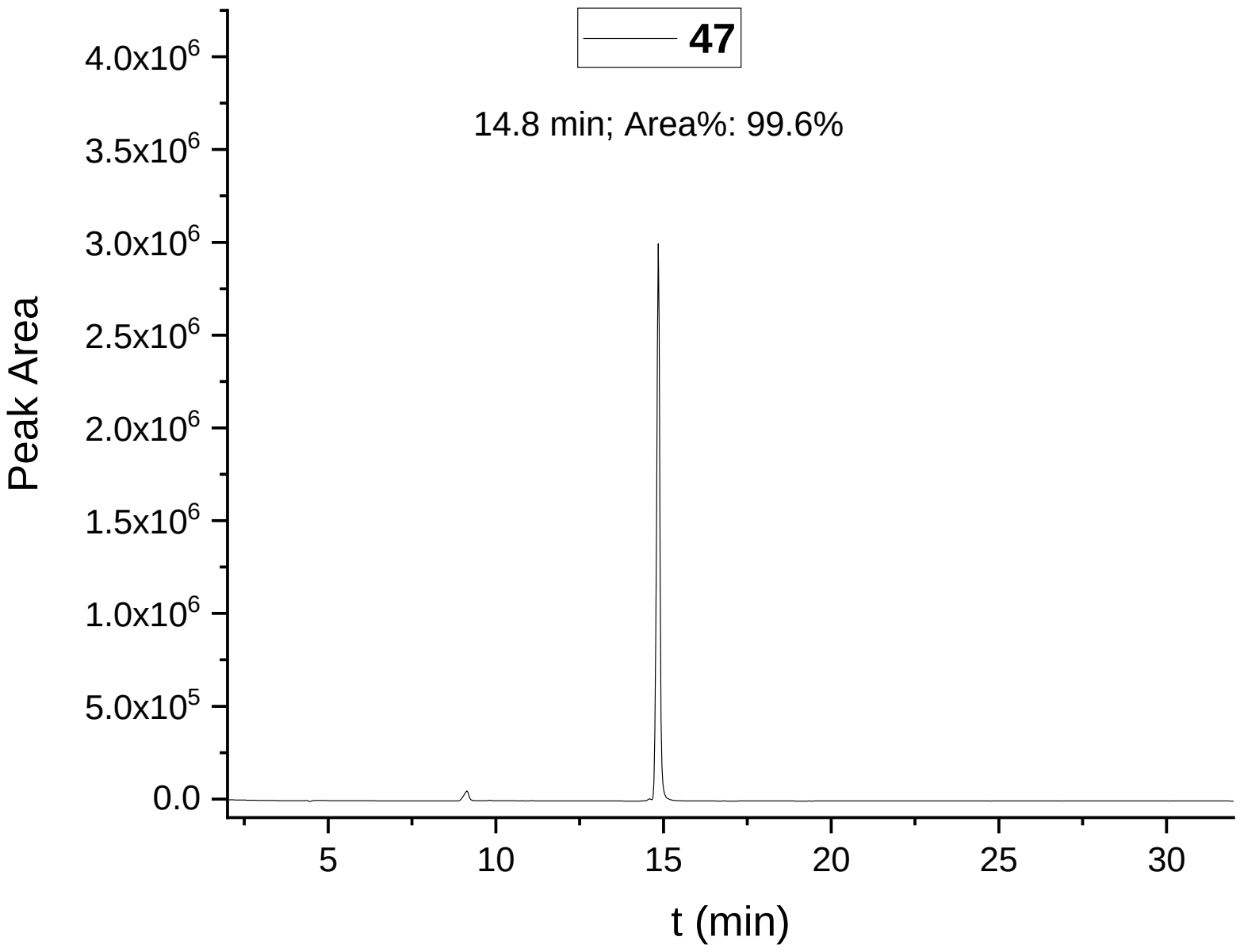
47
14.8 min; Area%: 99.6%
Peak Area
t (min)

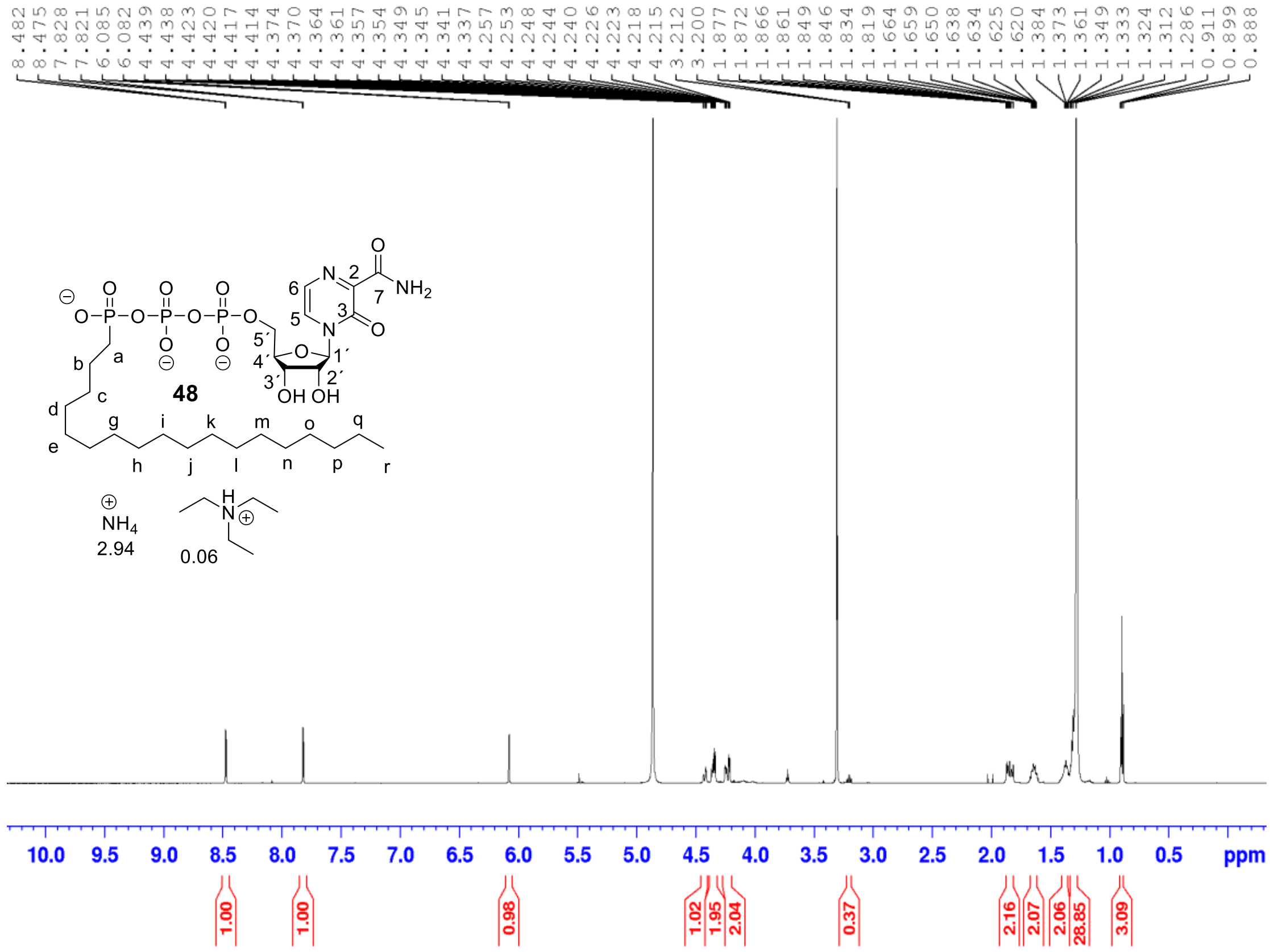
48
NH4
2.94
0.06
ppm

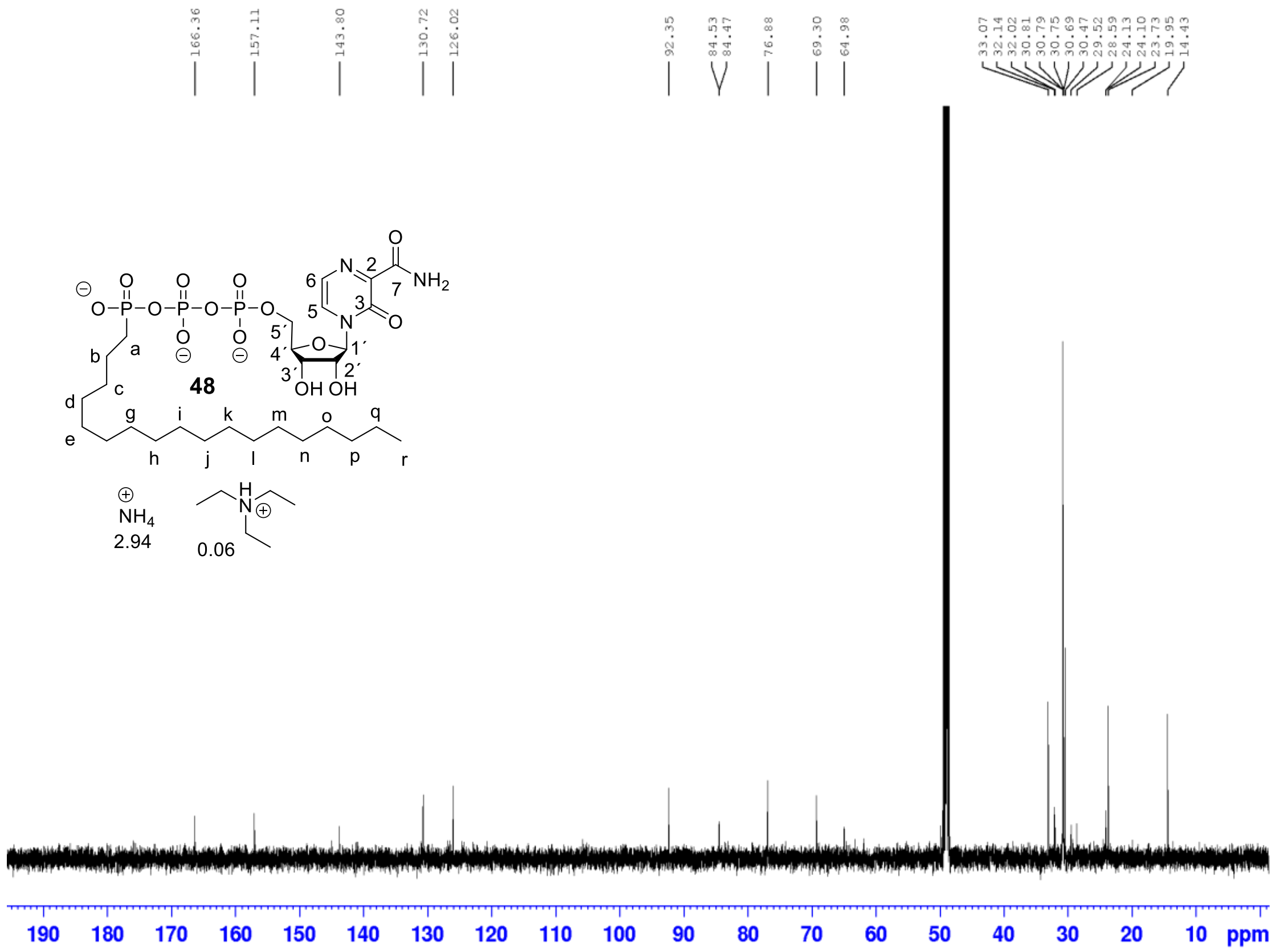
166.36
157.11
143.80
130.72
126.02
92.35
84.53
84.47
76.88
69.30
64.98
33.07
32.14
32.02
30.81
30.79
30.75
30.69
30.47
29.52
28.59
24.13
24.10
23.73
19.95
14.43
48
NH4
2.94
0.06
190 180 170 160 150 140 130 120 110 100 90 80 70 60 50 40 30 20 10 ppm

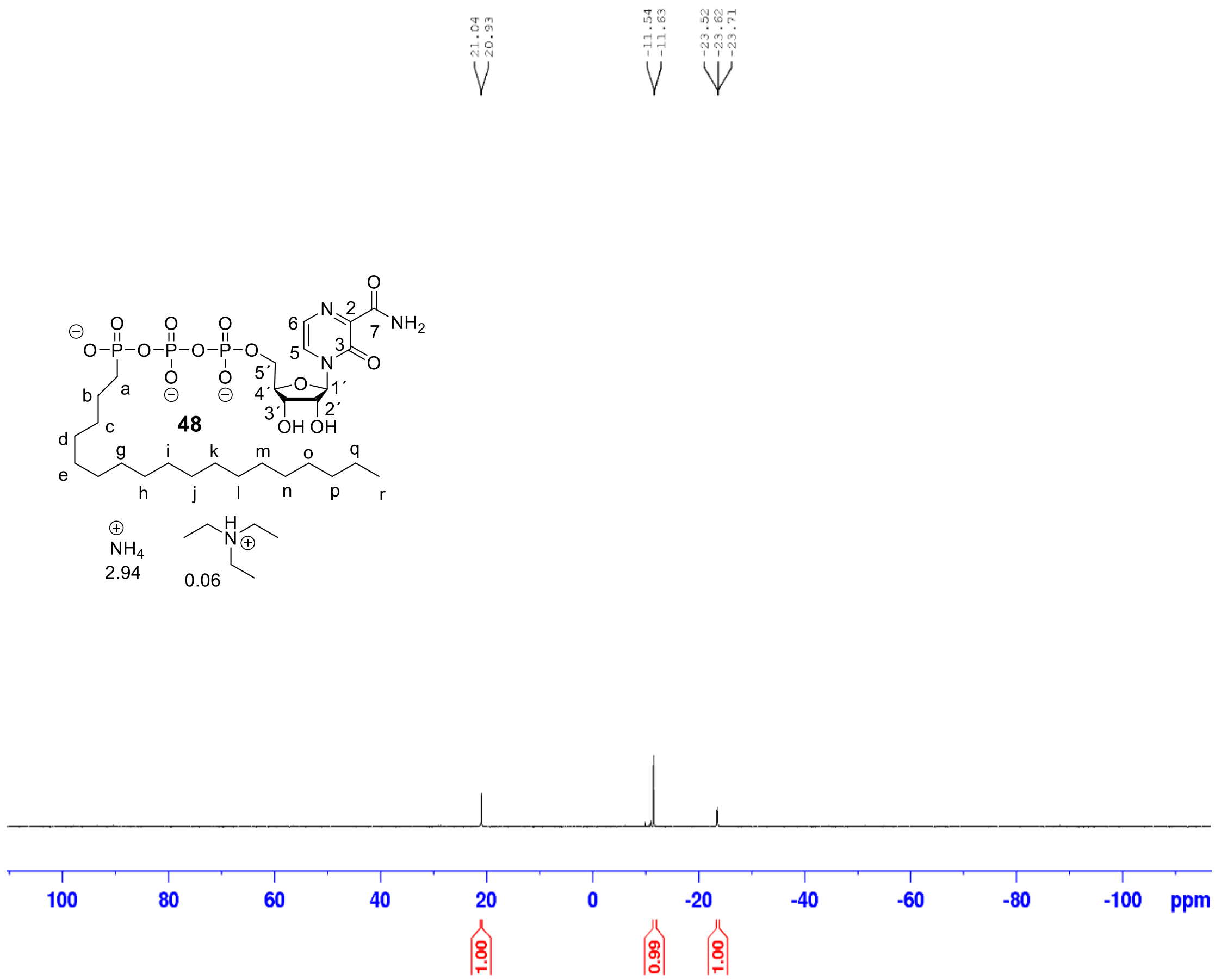
21.04
20.93
-11.54
-11.63
-23.52
-23.62
-23.71
48
NH4
2.94
0.06
100 80 60 40 20 0 -20 -40 -60 -80 -100 ppm
1.00
0.99
1.00

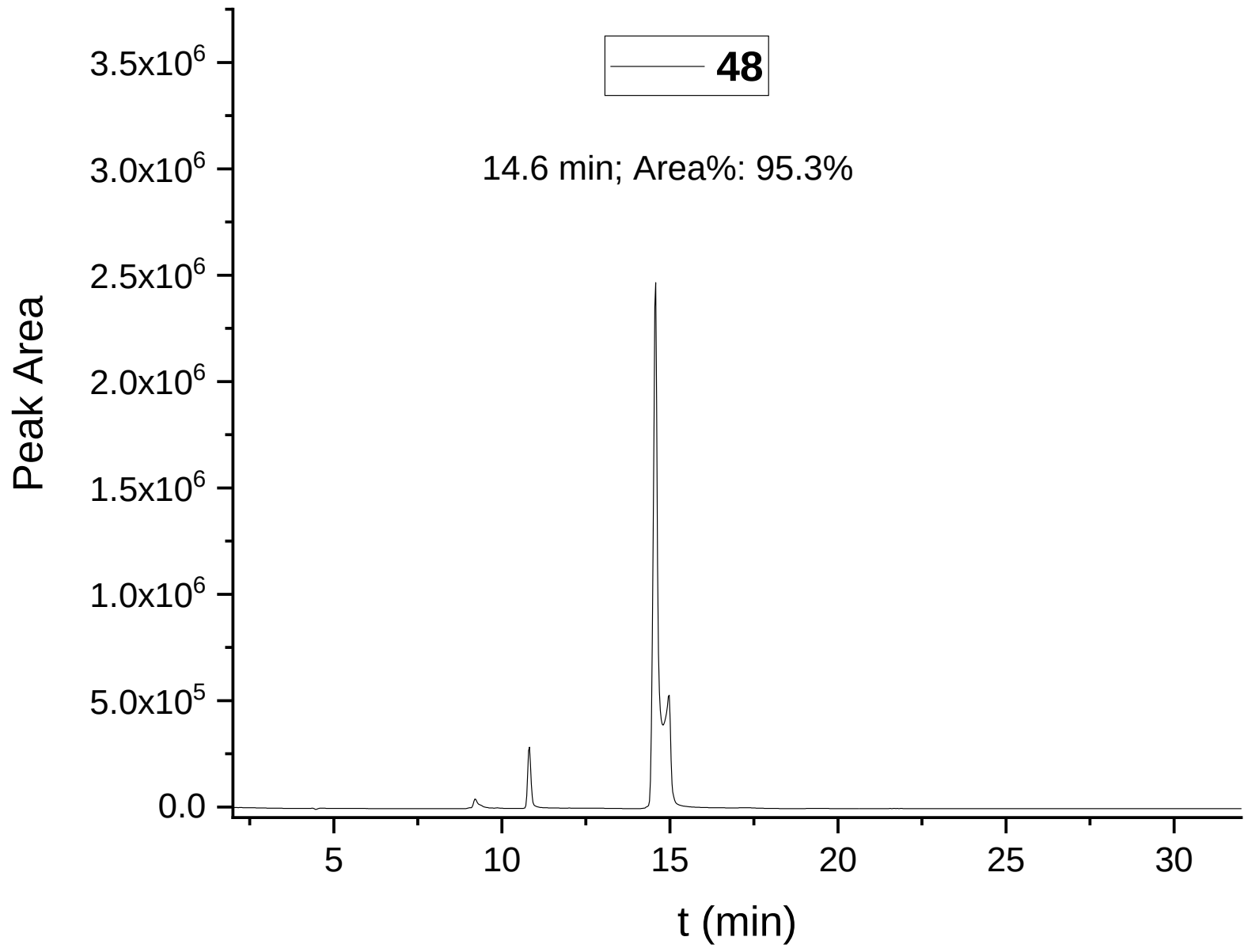

Reference

[1] S. Rimaux, A. Valdivia, J. Martín-López, V. Francesconi, C. Escriche, C. Mestdagh, R. Van Berwaer, L. Schurmans, K. Verleye, S. Noppen, Ó. Lozano, A. Stevaert, F. J. Luque, L. Naesens, S. Vázquez, *J. Med. Chem.* **2025**, *68*, 18491-18518.

[2] Ç. B. Apaydın, N. Cesur, A. Stevaert, L. Naesens, Z. Cesur, *Arch Pharm Chem Life Sci.* **2019**, *352*, e1800330.

[3] P. Vrijens, S. Noppen, T. Boogaerts, E. Vanstreels, R. Ronca, P. Chiodelli, M. Laporte, E. Vanderlinden, S. Liekens, A. Stevaert, L. Naesens, *J Gen Virol*. **2019**, *100*, 583-601.

[4] X. Jia, D. Schols, C. Meier, *J. Med. Chem.* **2023**, *66*, 12163-12184.

[5] C. Meier, L. Habel, F. Haller-Meier, A. Lomp, M. Herderich, R. Klocking, A. Meerbach, P. Wutzler, *Antiviral. Chem. Chemother.* **1998**, *9*, 389-402.

[6] A. Meerbach, R. Klöcking, C. Meier, A. Lomp, B. Helbig, P. Wutzler, *Antiviral Res.* **2000**, *45*, 69–77.

[7] Z. Szűcs, L. Naesens, A. Stevaert, E. Ostorházi, G. Batta, P. Herczegh, A. Borbás, *Pharmaceuticals* **2020**, *13*, 139.
